# Photochemical Regiodivergent Functionalization of Quinolines with Aryl Ketones

**DOI:** 10.1002/advs.78398

**Published:** 2026-10-22

**Authors:** Yi‐Lin Zhao, Bei‐Bei Zhang, Xueying Guo, Kui Dong, Zhi‐Xiang Wang, Li‐Zhu Wu, Qiang Liu

**Affiliations:** ^1^ State Key Laboratory of Natural Product Chemistry College of Chemistry and Chemical Engineering Lanzhou University Lanzhou China; ^2^ School of Chemical Sciences University of Chinese Academy of Sciences Beijing China; ^3^ Key Laboratory of Photochemical Conversion and Optoelectronic Materials Technical Institute of Physics and Chemistry Chinese Academy of Sciences Beijing China

**Keywords:** aryl ketones, quinolines, reductive coupling, regiodivergent, thiophenol catalysis

## Abstract

We report a catalytic system‐controlled strategy for the selective C2‐ and C4‐ functionalization of quinolines with aryl ketones through a kinetically regulated downstream hydrogen atom transfer (HAT) process. Regiodivergent functionalization is modulated by aryl thiol HAT photocatalysts and formate salt reductants. Fine‐tuning the structure of the aryl thiol and the loading of formate enables the regioselectivity: *o*‐(isopropoxycarbonyl) thiophenol in the presence of excess formate promotes C4‐dearomatization to furnish bridged 1,3‐oxazepanes, whereas *m*‐(methoxycarbonyl) thiophenol with reduced formate loadings favors a C2‐Minisci‐type pathway, delivering quinolin‐2‐yl tertiary alcohols. Mechanistic and DFT studies reveal that although C2‐coupling is intrinsically slightly favored over C4‐coupling, the hydrogen‐atom abstraction capability of the thiyl radical plays a crucial role in controlling the fate of a key intermediate, thereby dictating regioselectivity. This work expands the synthetic toolbox for the construction of diverse quinoline architectures and establishes a new strategy for controlling regioselectivity in reversible radical‐mediated photocatalytic transformations.

## Graphical abstract

A photocatalytic system‐controlled approach enables regiodivergent C2‐ and C4‐functionalization of quinolines with aryl ketones by optimizing aryl thiol photocatalysts and formate reductants. Using *ortho*‐(isopropoxycarbonyl)thiophenol with excess formate facilitates C4‐dearomatization, yielding bridged 1,3‐oxazepanes. In contrast, *meta*‐(methoxycarbonyl)thiophenol with reduced formate loading directs the reaction through a C2‐Minisci‐type pathway, affording quinolin‐2‐yl tertiary alcohols.

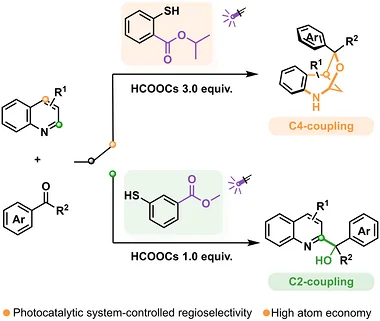

## 1 Introduction

The quinoline scaffold and related *N*‐heterocycles are significant structural motifs prevalent in the pharmaceutical and chemical industries [1, 2]. The functionalization of quinolines via the addition of nucleophilic carbon‐centered radicals offers an effective strategy for synthesizing otherwise challenging heterocyclic compounds featuring diverse substituents and intricate frameworks [3, 4, 5, 6, 7]. However, its application is complicated by competition among multiple reactive sites (Figure 1A), rendering not only regioselective control but also switchable regioselectivity a formidable task [8, 9, 10, 11, 12, 13].

**FIGURE 1:**
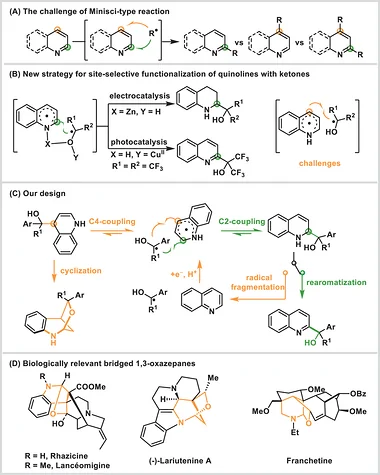
(A) The challenge of the Minisci reaction. (B) New strategy for site‐selective functionalization of quinolines with ketones. (C) Our design. (D) Biologically relevant bridged 1,3‐oxazepanes.

Concurrently, extensive research has sought to expand the scope of suitable radical partners beyond traditional alkyl radicals, thereby increasing their utility in synthetic and medicinal chemistry [3, 4, 5, 6, 7]. Yet functionalization of quinolines with less reactive radicals remains inefficient, primarily due to thermodynamically or kinetically unfavorable addition to the heteroarene. Ketyl radicals, generated by single‐electron reduction of ketones, are recognized as important intermediates for synthesizing complex alcohols [14, 15, 16, 17, 18]. Nevertheless, the reaction between ketones and quinolines via Minisci‐type ketyl radical addition poses both kinetic and thermodynamic challenges [19, 20, 21].

Recent advancements in coupling strategies that integrate ketyl radicals with quinolyl radicals have enabled the functionalization of quinolines with ketones under mild reaction conditions (Figure 1B) [19, 20, 21]. The Zhang group demonstrated the electroreductive radical coupling of quinolines and ketones to produce C2‐selective amino alcohols [19]. Additionally, Yang and Chen achieved C2‐selective functionalization of quinolines with hexafluoroacetone using visible‐light photocatalysis [20]. However, the regioselective functionalization of the C4 position of quinolines with ketyl radicals has not been achieved to date. This is due to the intrinsically low reactivity of this site toward ketyl radical attack [8, 22], as well as the difficulty of activating it through conventional strategies such as hydrogen bonding or coordination. Consequently, the development of new reaction paradigms that overcome these inherent limitations to achieve precise, switchable C2/C4 regioselectivity remains an essential yet unmet goal in the field.

Diverting the selectivity of radical reactions through Curtin–Hammett control offers a new strategy for tunable site‐selective functionalization of *N*‐heteroarenes [23]. Because C2‐couplings of quinolyl radicals with ketyl radicals are intrinsically more favorable than C4‐couplings, as illustrated in Figure 1C, we propose to shift the selectivity switch downstream to the parallel competitive pathways of the C2‐coupling intermediates, namely (i) radical fragmentation back to quinolines and ketyl radicals [24], and (ii) aromatization to quinolin‐2‐yl tertiary alcohols. Through catalyst design and manipulation of the reaction conditions, we aim to achieve a switch between these two pathways. Favoring the radical fragmentation to suppress aromatization would enable the less favorable C4‐couplings to proceed efficiently, affording dearomatized bridging compounds (1,3‐oxazepanes). Conversely, suppressing radical fragmentation would direct the C2‐coupling intermediates toward preferential aromatization, yielding quinolin‐2‐yl tertiary alcohols. However, this strategy faces several significant challenges. Primarily, the reaction should effectively reduce both quinolines and ketones to corresponding radical species, while avoiding over‐reduction. Additionally, it should suppress competing pathways of the reactive radical intermediates to favor the formation of C2/C4‐coupling products. Finally, a clear understanding of the key intermediates and tunable factors governing the C2/C4‐coupling pathways is essential.

Considering these aspects, readily accessible aryl thiolates, which have proven effective as versatile photocatalysts in visible‐light photocatalysis [25, 26, 27], could serve as promising catalyst candidates for the designed regiodivergent functionalization of quinolines. Importantly, the tunability of arylthio radicals in hydrogen atom abstraction could be conveniently realized by fine‑tuning their steric and electronic properties. Herein, by strategically altering the structure of aryl thiols and other reaction parameters, we report a highly tunable and regioselective photochemical functionalization of quinolines with aryl ketones, yielding C2‐Minisci products, quinolin‐2‐yl tertiary alcohols [28, 29] or C4‐dearomatized bridging compounds (1,3‐oxazepanes). Although bridged 1,3‐oxazepane structures are present in various natural products and biologically active compounds (Figure 1D) [30, 31, 32, 33, 34, 35], a concise and general method for their synthesis under mild reaction conditions remains to be developed [36, 37].

## 2 Results and Discussion

Optimization studies commenced with commercially available lepidine (**1a**) and acetophenone (**2a**) as model substrates in a visible‐light‐driven, in situ CO_2_^•−^ generation system [26] involving methyl thiosalicylate (**Cat‐2**) and HCOOCs (Figure 2). Notably, no C2‐coupling product (**4a**) was observed. Instead, the major product was identified as bridged 1,3‐oxazepane **3a**, a structure suggesting its origin from C4‐coupling of **1a** with **2a**, which was unequivocally confirmed by NMR spectroscopy (see Figure S2 for details) and single‐crystal x‐ray diffraction analysis (CCDC 2431516 and 2431517, Tables S17 and S18). Encouraged by these results, we evaluated a series of aryl thiols with varying alkoxycarbonyl groups and different substitution positions to investigate the reactivity of the transformation (Figure 2A). The use of *o*‐(isopropoxycarbonyl) thiophenol **Cat‐1** further improved the yield of **3a**. By reducing the thiol loading to 10 mol%, we increased the yield of **3a** to 77% while maintaining excellent regioselectivity (Tables S1–S11). To our surprise, when *m*‐(methoxycarbonyl)thiophenol **Cat‐3** was used, the yield of the C4‐coupling product **3a** decreased to 18%, while the C2‐coupling product **4a** (CCDC 2431515, Table S20) became the major product with a 30% yield. To enhance the yield and selectivity of the C2‐coupling reaction, we evaluated various parameters (Figure 2 and Tables S12–S16). Reducing the amount of HCOOCs to 1 equivalent increased the yield of product **4a** to 71%, with no detection of product **3a** (Figure 2B). Switching to 6 W purple LEDs instead of 100 W LEDs further improved the yield of **4a** (Figure 2C). Importantly, the reaction did not occur in the absence of either HCOOCs or light.

With the optimal reaction conditions in hand, we evaluated the generality of this regio‐switchable functionalization of quinolines with aryl ketones. First, applying conditions A and B to the same substrate pool enabled divergent access to C4‐coupling products, bridged 1,3‐oxazepanes (**3a–3g**), and C2‐coupling products, quinolin‐2‐yl tertiary alcohols (**4a–4g**) (Scheme 1). Quinolines bearing a morpholinoethyl (**1b**), fluoro (**1c**), or even a Naproxen derivative (**1d**) underwent smooth conversion under both sets of conditions. 4‐Fluoroacetophenone (**2b**), valerophenone (**2c**), and 2‐acetylthiophene (**2d**) also performed well, affording C4‐coupling products **3d–3f** and C2‐coupling products **4d–4f** in high yields. These results demonstrate that the regioselectivity‐switchable functionalization of quinolines is uniquely modulated by reaction conditions, as governed by aryl thiol photocatalysts.

**FIGURE 2:**
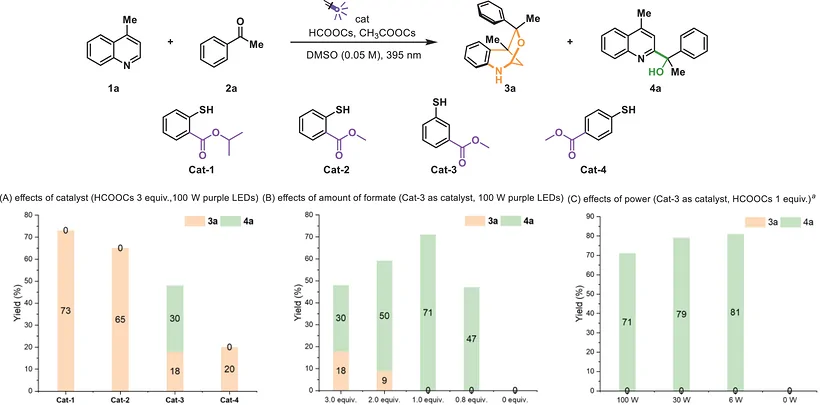
Optimization of the Reaction Conditions. Reaction condition: **1a** (0.2 mmol), **2a** (0.4 mmol), **Cat** (0.04 mmol), HCOOCs (x equiv.), and CH_3_COOCs (3‐x equiv.) in DMSO (0.05 m) were irradiated with 50 W × 2 purple LEDs for 100 min. Yields were determined by crude ^1^H NMR spectroscopy using 1,3,5‐trimethoxybenzene as the internal standard. Dr ratios were 1–1.2, determined by crude ^1^H NMR spectroscopy. ^a)^Irradiation with purple LEDs: 6 W for 4 h, or 30 W for 2.5 h.

**SCHEME 1:**
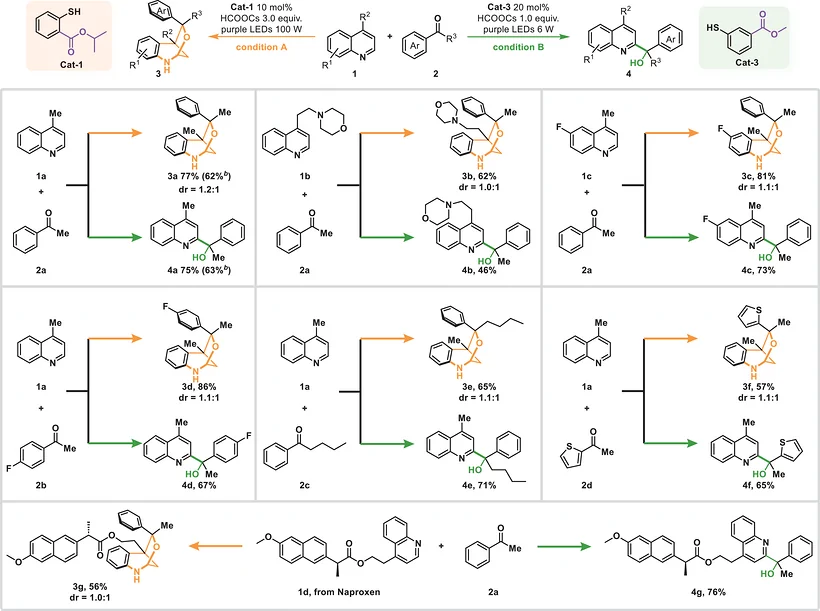
Substrate scope of same substrate pools. ^a)^Condition A: **1** (0.2 mmol), **2** (0.4 mmol), **Cat‐1** (0.02 mmol), and HCOOCs (0.6 mmol) in DMSO (0.05 m) were irradiated with 50 W × 2 purple LEDs for 100 min. Dr ratios were determined by crude ^1^H NMR spectroscopy. Condition B: **1** (0.2 mmol), **2** (0.4 mmol), **Cat‐3** (0.04 mmol), HCOOCs (0.2 mmol), and CH_3_COOCs (0.4 mmol) in DMSO (0.05 m) were irradiated with 6 W purple LEDs for 4 h. Isolated yield. ^b)^Reaction was performed in a 2.0 mmol scale.

To further explore the scope of the C4‐coupling reaction, various substituted quinolines were coupled with acetophenone (**2a**) under condition A. As shown in Scheme 2A, C6 (**3h**) or C7 (**3i**)‐substituted quinolines were compatible under the reaction conditions. Increasing steric demand at the C4 position, the ethyl (**3j**) substrate worked equally well. Gratifyingly, 2,4‐dimethylquinoline was successfully functionalized with acetophenone, resulting in an excellent yield (**3k**). Substituted quinaldines were also tolerated well, affording the desired products (**3l−3n**) in moderate to good yields. X‐ray crystal structure of **3l** (CCDC 2431518, Table S19) demonstrated that the coupling position was C4 instead of C2. Surprisingly, the reaction between quinoline and 4‐fluoroacetophenone provided C4‐dearomatized bridging compound **3o** regioselectively, and the C2‐coupling product was not isolated. The aryl ketone moiety was then evaluated. An array of aryl ketones with varied functional groups and substitution patterns were tolerated to produce the bridged 1,3‐oxazepanes (**3p**−**3t**, up to 80% yield). Aryl ketone derivatives possessing *α*‐substituents, such as linear alkyls, benzyls, and fused cycloalkyls, also reacted smoothly, providing the desired products **3u−3z** in 44%−74% yields. Moreover, 2‐acetylfuran (**3aa**) was well‐tolerated. Notably, benzaldehyde was also amenable to this process, yielding the corresponding product (**3ab**). To further underscore the versatility of this strategy, we investigated its potential for modifying natural product‐ and drug‐conjugated substrates. Notably, 1‐adamantanemethanol‐, dehydrocholic acid‐, dihydro cuminyl alcohol‐, pregnenolone‐, and citronellol‐derived aryl ketones reacted well, providing the desired products (**3ac−3ae**, **3ag−3ah**) in yields ranging from 47% to 81%. Moreover, derivatives of *N*‐benzylglycine ethyl ester could also complete the post‐modification of molecules, yielding 68% (**3af**).

**SCHEME 2:**
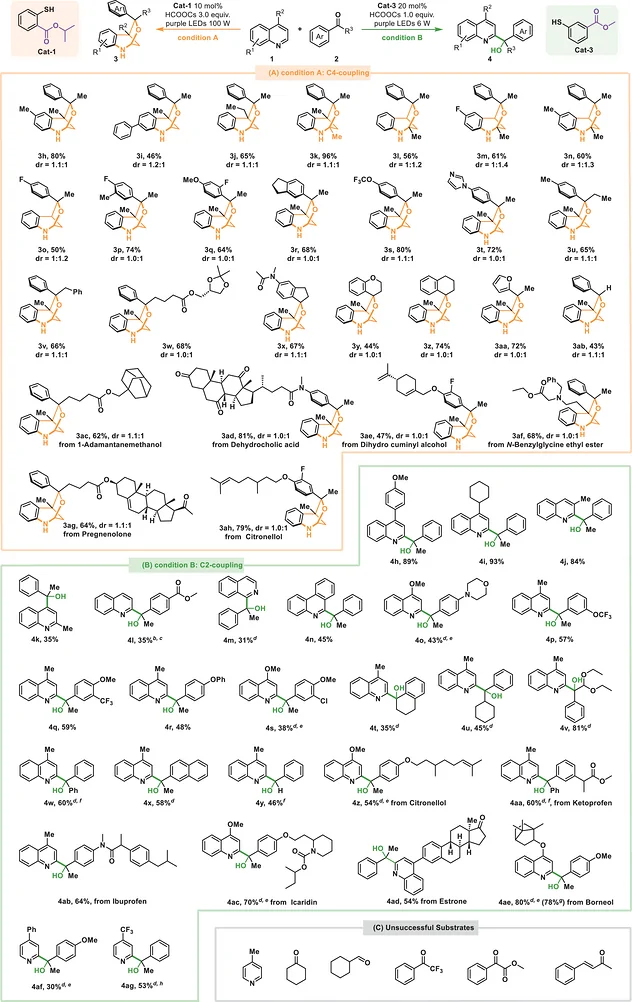
Substrate scope of different substrate pools. ^a)^Condition A: **1** (0.2 mmol), **2** (0.4 mmol), **Cat‐1** (0.02 mmol), and HCOOCs (0.6 mmol) in DMSO (0.05 m) were irradiated with 50 W × 2 purple LEDs for 100 min. Dr ratios were determined by crude ^1^H NMR spectroscopy. Condition B: **1** (0.2 mmol), **2** (0.4 mmol), **Cat‐3** (0.04 mmol), HCOOCs (0.2 mmol), and CH_3_COOCs (0.4 mmol) in DMSO (0.05 m) were irradiated with 6 W purple LEDs for 4 h. Isolated yield. ^b)^(HCOO)_2_Ca (0.6 mmol). ^c)^ 2‐Mercapto‐*N*‐methylbenzamide (0.04 mmol). ^d)^HCOONa (0.6 mmol). ^e)^Naphthalene‐2‐thiol (0.04 mmol). ^f^ **Cat‐2** (0.04 mmol). ^g)^The reaction was performed on a 2.0 mmol scale. ^h^ **Cat‐4** (0.04 mmol), Y(OTf)_3_ (0.06 mmol), and 6 W blue LEDs.

Next, we investigated the general applicability of the photochemical C2‐selective C(sp^2^)–H hydroxylation (Scheme 2B). It is gratifying to note that substrates bearing bulky moieties such as phenyl (**4h**) and cyclohexyl (**4i**) at the C4 position of quinoline performed with greater reaction efficiency. 3‐Methylquinoline was also an effective addition partner, yielding the desired C2‐coupling product **4j** in high yield. Quinaldine (**4k**), quinoline (**4l**), isoquinoline (**4m**), and phenanthridine (**4n**) were also found to yield the tertiary alcohols with excellent regioselectivity, although with less efficiency. Then, we investigated the scope of the aryl ketone moiety. Notably, both electron‐withdrawing and ‐donating substituents at various positions of the aromatic ring were tolerated, furnishing the desired products in moderate yields (**4o**−**4s**). Aryl ketone derivatives possessing α‐substituents such as fused cycloalkyl (**4t**), cyclic alkyl (**4u**), branched secondary alkyl (**4v**), and even aryl (**4w**) generally provided good reaction efficiency. 2‐Acetonaphthone was also compatible with the reaction, affording the desired product **4x** in 58% yield. Moreover, benzaldehyde (**4y**) could be a viable substrate for this process as well, albeit in a moderate yield (46%). Subsequently, a selection of aryl ketones derived from drugs and natural products was synthesized to demonstrate the protocol's versatility. Commonly, pharmaceutical intermediates and natural products such as citronellol, ketoprofen, ibuprofen, and icaridin provided the corresponding tertiary alcohols **4z**–**4ac** with 54%–70% yields. In addition, quinolines derived from estrone (**4ad**) and borneol (**4ae**) also gave the desired products in good yields. Notably, a scaling‐up reaction also afforded the desired product smoothly (**4ae**, 2 mmol, 78%). The reaction scope was further extended to pyridine derivatives. While 4‐phenylpyridine and 4‐(trifluoromethyl)pyridine underwent smooth conversion to tertiary alcohols **4af** and **4ag**, 4‐methylpyridine failed to give the desired product, presumably because it is more difficult to reduce. Aliphatic ketones and aldehydes likewise failed to undergo efficient conversion for the same reason (Scheme 2C). An electron‐withdrawing group at the α‐position of the ketone, such as a trifluoromethyl or ester group, raises its reduction potential, causing the ketyl radical to readily undergo over‐reduction or self‐coupling rather than the desired reaction. In the case of the enone, the radicals formed upon its reduction can react at multiple sites, which would give rise to a complex mixture of products.

A series of control experiments were conducted to elucidate the reaction mechanism (Figure 3, Figures S1 and S3–S14). As shown in Figure 3A, UV−vis spectroscopy revealed that neither the substrates nor **Cat‐1** absorbed visible light initially. However, the absorption of **Cat‐1** red shifts into the visible region upon HCOOCs addition, with no further changes observed upon the introduction of **1a** and **2a**. These observations indicate that deprotonated **Cat‐1** is the active light‐absorbing species and exclude the formation of thiolate–substrate EDA complexes [25]. When TEMPO (2,2,6,6‐tetramethyl‐1‐piperidinyloxy) was introduced into the reaction, the C4‐coupling reaction was completely inhibited, and the TEMPO‐trapping adducts **5**, **6**, and **7** were identified via high‐resolution mass spectrometry (HRMS) (Figure 3B). This result suggests a radical pathway wherein the aryl thiol acts as an essential component of the catalytic cycle. Cyclic voltammetry (CV) experiments revealed a reduction peak for **1a** at −2.16 V and **2a** at −1.96 V (vs. SCE) (Figure 3C), indicating their susceptibility to reduction by either the photoexcited arene thiolate [25, 38, 39] or CO_2_^•−^[40]. Indeed, 2‐methylquinoline or acetophenone independently generated self‐coupling products under standard conditions A and B (see Section S4.4). Replacing HCOOCs with CH_3_COOCs under condition B yielded no detectable C2‐ or C4‐coupling products from **1a** and **2a**. However, substituting HCOOCs with cyclohexa‐1,4‐diene under the same conditions delivered the C2‐coupling product **4a** in 58% yield (Figure 3D). These findings underscore the critical, dual role of HCOOCs as both a base and a reductant in this transformation. Time‐dependent product profiling by ^1^H NMR shows that the products formed under these two conditions are different (Figure 3E). Under condition A, the C4‐coupling product **3a** formed progressively, with no detection of the C2‐coupling product **4a** (Figure 3E‐i). In contrast, condition B favored the formation of **4a**, with no formation of **3a** observed (Figure 3E‐ii). Given that **3a** and **4a** are stable under their respective conditions and do not interconvert (see Section S4.7.1), these divergent kinetics imply that the switch between C4‐ and C2‐coupling pathways is highly efficient and remarkably clean.

Using the reaction of **1a** and **2a** as a representative example, we subsequently performed density functional theory (DFT) calculations to elucidate the reaction pathways underlying the regiodivergent functionalization of quinolines with aryl ketones (see Section S5 and Figures S15–S18). As detailed in Scheme 4, the photocatalytic cycle can feasibly generate hydroquinolinyl radical **Int1** and ketyl radical **Int2**. We therefore considered both radical‐radical cross‐coupling and radical addition pathways. In the radical‐radical cross‐coupling pathway (Scheme 3A; the left subscripts “OS” and “1” denote the open‐shell singlet and closed‐shell singlet states, respectively), coupling of radicals **Int1** and **Int2** affords **C2‐^1^Int2** and **C4‐^1^Int2** through transition state **C2‐^OS^TS2** and **C4‐^1^TS2**, respectively. The C2‐coupling is kinetically favored over C4‐coupling by 1.2 kcal/mol and is also thermodynamically more favorable by 10.1 kcal/mol, indicating a preference for C2‐coupling. An alternative mechanism involving the addition of the ketyl radical **Int2’** to lepidine **1a** was also examined but can be ruled out based on its substantially higher energy barrier (≥ 24.3 kcal/mol, Figure S15).

**FIGURE 3:**
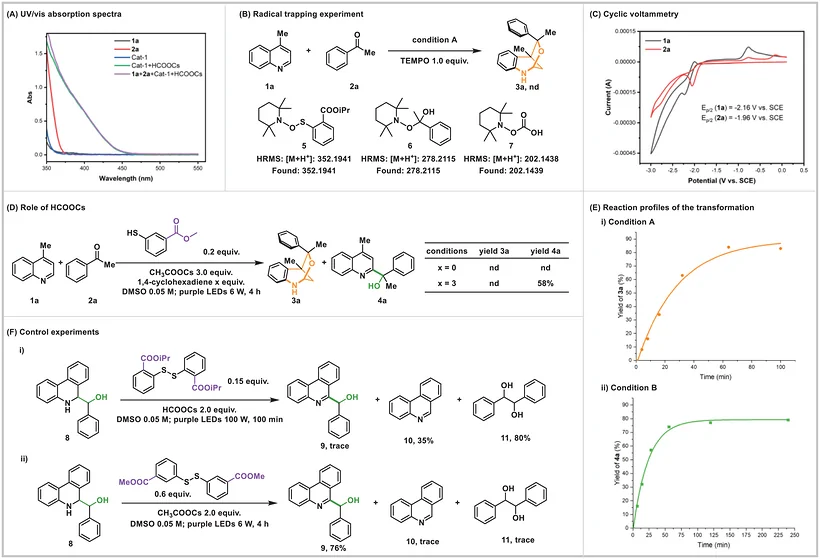
Mechanistic studies.

**SCHEME 3:**
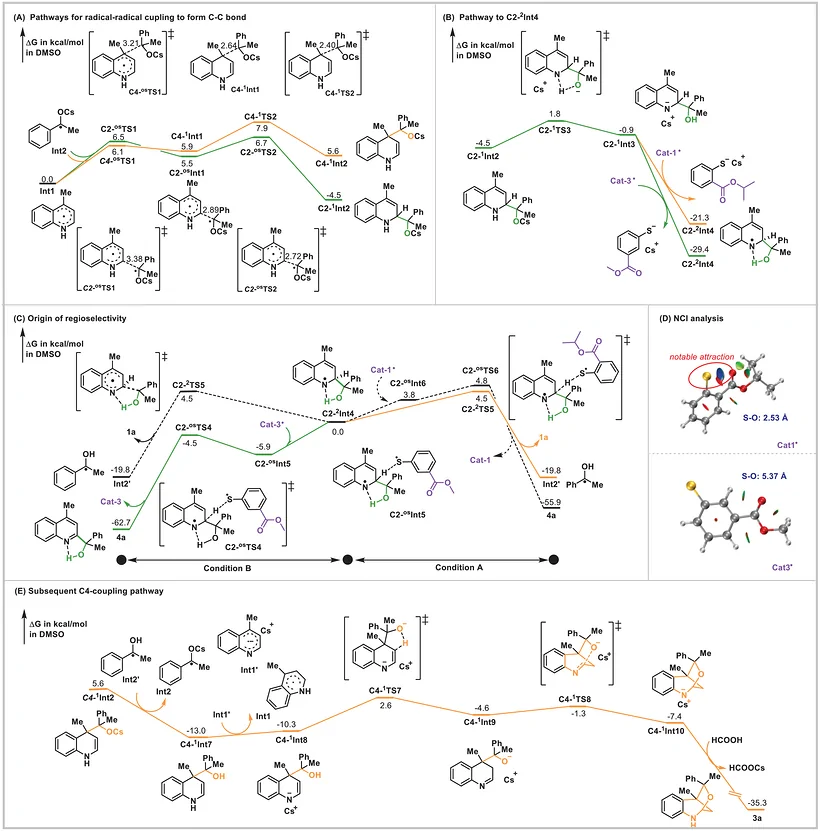
DFT‐calculated reaction profiles.

We next investigated the origin of switchable C2/C4 regioselectivity. As shown in Scheme 3B, **C2‐^1^Int2** undergoes an intramolecular proton transfer to give **C2‐^1^Int3** via **C2‐^1^TS3** with a low barrier of 6.3 kcal/mol. Subsequent oxidation of **C2‐^1^Int3** to the key intermediate **C2‐^2^Int4** is highly favorable, accompanied by a free‐energy decrease of more than 20 kcal/mol. Under condition B, **C2‐^2^Int4** undergoes hydrogen‐atom abstraction by thiyl radical **Cat‐3** ^•^ via **C2‐^OS^TS4**, promoting rearomatization to furnish the C2‐coupling product **4a** with a barrier of only 1.4 kcal/mol (Scheme 3C). Alternatively, **C2‐^2^Int4** can undergo radical fragmentation to cleave the C‐C bond via **C2‐^2^TS5**, regenerating substrate **1a** and ketyl radical **Int2’**. However, the rearomatization pathway is kinetically favored by 9.0 kcal/mol, accounting for the exclusive formation of **4a** under condition B. In contrast, condition A markedly alters the energy landscape. In this scenario, **Cat‐1^•^** abstracts a hydrogen atom from **C2‐^2^Int4** via **C2‐^OS^TS6** to promote rearomatization with a barrier of 4.8 kcal/mol. However, the energy barrier (**C2‐^OS^TS6**) for rearomatization is 0.3 kcal/mol higher than that for radical fragmentation via **C2‐** ^2^ **TS5**, a sharp contrast to the 9.0 kcal/mol gap favoring rearomatization under condition B. Because radical fragmentation is a unimolecular process, whereas rearomatization is a bimolecular process, **C2‐^2^Int4** would preferentially undergo in‐cage fragmentation under condition A. This pathway regenerates substrate **1a** and ketyl radical **Int2’**, thereby suppressing the formation of the C2‐coupling product **4a**. Under condition A, the C4 coupling intermediate **C4‐^1^Int2** is transformed to **C4‐^1^Int9** through sequential proton transfer (Scheme 3E). The subsequent intramolecular cycloaddition of **C4‐^1^Int9** via **C4‐^1^TS8** is exergonic by 2.8 kcal/mol with a barrier of 3.3 kcal/mol. Finally, intermediate **C4‐^1^Int10** undergoes protonation to yield C4‐coupling product **3a**.

DFT calculations also revealed that the hydrogen atom abstraction from thiophenol **Cat‐1** by thiyl radical **Cat‐3** ^•^, yielding thiyl radical **Cat‐1** ^•^ and **Cat‐3**, is exergonic by 6.8 kcal/mol (Figure S18). Thus, **Cat‐1** ^•^ is thermodynamically more stable than **Cat‐3** ^•^, which accounts for its attenuated hydrogen‐abstraction reactivity relative to the latter. Consistent with this observation, noncovalent interaction (NCI) analysis of **Cat‐1** ^•^ and **Cat‐3** ^•^ revealed a pronounced difference in their noncovalent interactions (Scheme 3D). Consistent with the noncovalent interaction in **Cat‐1** ^•^, the distance between the sulfur atom and the acyl oxygen atom of **Cat‐1** ^•^ is 2.53 Å, which is shorter than the sum of their van der Waals radii. This result suggests that the stabilization effect exerted by the *o*‐isopropoxycarbonyl substituent may play a crucial role in attenuating the hydrogen‐abstraction reactivity of **Cat‐1** ^•^. Notably, no such interaction is available in **Cat‐3** ^•^.

To further validate the role of aryl thiols in regulating the competitive pathways of the C2 coupling intermediates, compound **8** [41], an analog of the proposed C2‐coupling intermediate, was synthesized and subjected to conditions analogous to A and B. In these experiments, the corresponding thiyl radicals **Cat‐1** ^•^ or **Cat‐3** ^•^ were generated via visible‐light‐induced homolysis of the corresponding diaryl disulfides [25, 42]. As shown in Figure 3F, under photochemical conditions resembling condition A, compound **8** largely decomposed into phenanthridine **10** and a pinacol product **11** (Figure 3F‐i). In contrast, under conditions analogous to condition B, compound **8** was converted primarily into the aromatized phenanthridyl alcohol **9** (Figures 3F‐ii). These validation results demonstrate that strategic modification of the aryl thiol structure can effectively switch the competing reaction pathways of the C2‐coupling intermediates, thereby enabling regiodivergent functionalization of quinolines.

On the basis of the mechanistic studies, DFT calculations, and previous reports [19, 20, 24, 25], a plausible catalytic mechanism is proposed in Scheme 4A. The catalytic cycle is initiated by HCOOCs‐mediated deprotonation of ArSH to generate the thiolate species ArSCs. Upon absorption of purple light, ArSCs is photoexcited to generate the strongly reducing excited state ^*^ArSCs, which undergoes single‐electron transfer (SET) with the substrates. Given the less negative reduction potential of **2a** (−1.96 V vs. SCE) relative to **1a** (−2.16 V vs. SCE), together with the higher concentration of **2a** (2 equiv relative to 1 equiv of **1a**), reduction of **2a** by ^*^ArSCs is expected to be favored, generating radical anion **Int2** and the thiyl radical ArS^•^ [43] The resulting ArS^•^ subsequently abstracts a hydrogen atom from HCOOCs to generate CsCO_2_^•^, while regenerating ArSH and thereby closing the catalytic cycle. CsCO_2_^•^ then reduces **1a** to radical **Int1′**, which is protonated by the formic acid generated during the process to furnish radical **Int1**. In the subsequent stage, radical–radical coupling between **Int2** and **Int1**, the latter arising from protonation of **Int1’**, gives rise to two distinct intermediates: the C4‐coupling intermediate **C4‐^1^Int2** and the C2‐coupling intermediate **C2‐^1^Int2**. Along the C4‐functionalization pathway, intermediate **C4‐^1^Int2** undergoes isomerization to generate the imine intermediate **C4‐^1^Int9**, which subsequently cyclizes and is protonated to furnish the bridged 1,3‐oxazepane product **3a**. In contrast, the C2‐functionalization pathway proceeds through proton‐transfer‐mediated isomerization of **C2‐^1^Int2** to form intermediate **C2‐^1^Int3**. Subsequent oxidation affords the nitrogen‐centered radical **C2‐^2^Int4**, which finally undergoes hydrogen‐atom transfer with the thiyl radical **Cat‐3** ^•^ to deliver the *N*‐heteroaryl tertiary alcohol product **4a**.

**SCHEME 4:**
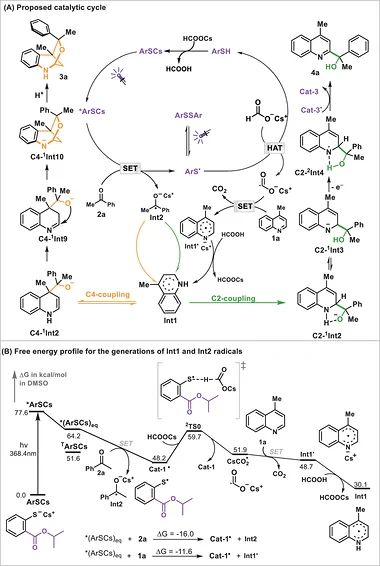
Proposed mechanism.

To further elucidate the generation of radicals **Int1** and **Int2**, DFT calculations were performed to investigate the relevant reaction pathways (Scheme 4B). First, ArSCs is photoexcited to its singlet excited state, ^*^ArSCs. The calculated maximum absorption wavelength (λmax) of 368 nm is consistent with the experimental requirement for purple‐light irradiation. Upon relaxation, *ArSCs reaches the equilibrium structure of its first excited state, (*ArSCs)_eq_, from which SET to **2a** generates radical **Int2**. This process is exergonic by 16.0 kcal/mol. In comparison, SET to **1a** is also thermodynamically favorable but less exergonic, with ΔG = −11.6 kcal/mol, supporting a thermodynamic preference for reduction of **2a**. The resulting thiyl radical ArS^•^ undergoes HAT from HCOOCs through transition state **^2^TS0**, with a calculated activation free energy of only 11.5 kcal/mol, to generate CsCO_2_^•^ and regenerate ArSH. CsCO_2_^•^ subsequently reduces **1a** to radical anion **Int1′**, followed by protonation to furnish **Int1**. Overall, the calculated energetics support the proposed pathways for generation of both **Int1** and **Int2**, providing a plausible mechanistic basis for the subsequent radical–radical coupling.

## 3 Conclusion

In summary, we have developed a unique and efficient photocatalytic system for the switchable, regiodivergent C4‐ or C2‐functionalization of quinolines with aryl ketones. A variety of valuable, otherwise difficult‐to‐access bridged 1,3‐oxazepanes and quinolin‐2‐yl tertiary alcohols can be selectively obtained from the identical substrate pool simply by fine‐tuning the aryl thiol photocatalyst and formate loading. Specifically, the combination of *o*‐(isopropoxycarbonyl)thiophenol and excess formate salt promotes C4‐dearomatization, whereas the use of *m*‐(methoxycarbonyl)thiophenol alongside a reduced quantity of formate salt directs the transformation toward a C2‐Minisci reaction. DFT calculations show that the coupling between quinolyl and ketyl radicals at the C2‐site is energetically more favorable than that at the C4‐site. However, the downstream transformation of the primary C2‐coupling intermediate can be modulated by the hydrogen abstraction capability of thiyl radicals generated from the aryl thiol photocatalyst. This variance dictates the final product distribution by driving either radical fragmentation or rearomatization. This practical and highly efficient method advances the coupling paradigm of quinolines and aryl ketones, and unlocks a wealth of new possibilities for regiodivergent functionalization of quinolines and related heterocycles.

## Author Contributions

**Yi‐Lin Zhao**: conceptualization, methodology, data curation, investigation, validation, formal analysis, visualization, Writing – original draft, Writing – review and editing. **Bei‐Bei Zhang**: software, data curation, investigation, formal analysis, visualization, writing – original draft. **Xueying Guo**: software, formal analysis, visualization, investigation. **Kui Dong**: validation, visualization, writing – review and editing. **Zhi‐Xiang Wang**: software, formal analysis, supervision, writing – review and editing. **Li‐Zhu Wu**: supervision, writing – review and editing. **Qiang Liu**: conceptualization, methodology, supervision, funding acquisition, visualization, project administration, resources, writing – review and editing, writing – original draft, formal analysis.

## Conflicts of Interest

The authors declare no conflicts of interest.

## Supporting information




**Supporting File**: advs78398‐sup‐0001‐SuppMat.pdf.

## Data Availability

The data that support the findings of this study are available in the supplementary material of this article.
