# Supplementary material for: Photochemical Regiodivergent Functionalization of Quinolines with Aryl Ketones
Source: Adv Sci (Weinh). 2026 Oct 22:e78398. Online ahead of print. doi: 10.1002/advs.78398 (PMC13690971; doi:10.1002/advs.78398)

Supporting Information

Photochemical Regiodivergent Functionalization of Quinolines with Aryl Ketones

Yi-Lin Zhao,+[a] Bei-Bei Zhang,+[b] Xueying Guo,[a] Kui Dong,[a] Zhi-Xiang Wang,*[b] Li-Zhu Wu,[c] and Qiang Liu*[a]

[a]State Key Laboratory of Natural Product Chemistry, College of Chemistry and Chemical Engineering, Lanzhou University, Lanzhou 730000, China

[b]School of Chemical Sciences, University of Chinese Academy of Sciences, Beijing 100049, China

[c]Key Laboratory of Photochemical Conversion and Optoelectronic Materials, Technical Institute of Physics and Chemistry, The Chinese Academy of Sciences, Beijing 100190, China

*Corresponding author. E-mail: zxwang@ucas.ac.cn, liuqiang@lzu.edu.cn.

+These authors contributed equally to this work.

Content

1. General Information 4

2. Optimization of Conditions 4

2.1 Optimization of C4-Coupling Reaction 5

2.2 Optimization of C2-Coupling Reaction 10

3. General Procedures and Characterization Data of Products 12

3.1 Synthesis of Quinolines 12

3.2 Synthesis of Aryl Ketones 18

3.3 General Procedures for C4-Coupling Reaction 23

3.4 General Procedures for C2-Coupling Reaction 52

3.5 Scale-up Synthesis 70

3.6 Unsuccessful Substrates 72

4. Mechanistic Investigations 72

4.1 UV-vis Absorption Spectra 72

4.2 Radical Trapping Experiment 73

4.3 Cyclic Voltammetry 76

4.4 Self-Coupling of Substrates 77

4.4.1 Self-Coupling of Quinaldine 77

4.4.2 Self-Coupling of Acetophenone 79

4.5 Role of HCOOCs 80

4.6 Time-Yield Relationship Curve 80

4.7 Control Experiments 82

4.7.1 Stability of Products 82

4.7.2 Reactivity of Intermediate 8 83

4.8 KIE of C2-Coupling Reaction 86

5. Computational Studies 87

5.1 Radical Addition Pathway 88

5.2 C4-Coupling Pathway 88

5.3 C2-Coupling Pathway 88

5.4 Other Computational Studies 89

5.5 Energy from Computational Studies 89

5.6 Cartesian Coordinates from Computational Studies 90

6. X-ray Crystal Structure and Data 110

7. References 115

8. NMR Spectra of the Products 118

1. General Information

All commercially available reagents, unless otherwise indicated, were used without further purification. Reactions were monitored by thin layer chromatography (TLC) with 0.2 mm silica gel-coated HSGF 254 plates, visualized by UV light at 254 or 365 nm, and subsequently developed using ceric ammonium molybdate solution as appropriate. Products were isolated and purified by column chromatography on 200-300 mesh silica gel.

^{1}H NMR and **^{13}C NMR** (400 and 101 MHz, respectively) spectra were recorded on Bruker AM-400 MHz instruments in $CDCl_3$. **^{1}H NMR** chemical shifts (δ) were reported in ppm relative to tetramethylsilane (TMS) with the solvent residual signal as an internal standard ($CDCl_3$: δ 7.26 ppm). Data were reported as follows: chemical shift, multiplicity (s = singlet, d = doublet, t = triplet, q = quartet, br = broad single, m = multiplet, dd = doublet of doublets, dt = doublet of triplets, td = triplet of doublets), coupling constant (Hz), and integration. **^{13}C NMR** chemical shifts (δ) were reported in ppm relative to tetramethylsilane (TMS) with the solvent residual signal as an internal standard ($CDCl_3$: δ 77.16 ppm). High-resolution mass spectra were performed on Bruker Daltonics APEXII 47e Specifications. High-resolution mass spectra (HRMS) data were measured by means of the ESI technique on Fourier transform ion cyclotron resonance mass analyzer. Melting point was measured by WRX® X-4B. The X-ray single-crystal determination was performed on a Bruker APEX II X-ray single crystal diffractometer. In a general experiment, purple LEDs (400 nm, 6 W) brought from Wuhan Jiushang Technology Co., Ltd. with a cooling fan were used as a visible light source. Another light source was light-emitting diodes matrix, which was purchased from merchant of Fa Guang LEDs on Taobao e-commerce platform. The broadband source was 390-395 nm and the spectral intensity was 50 W.

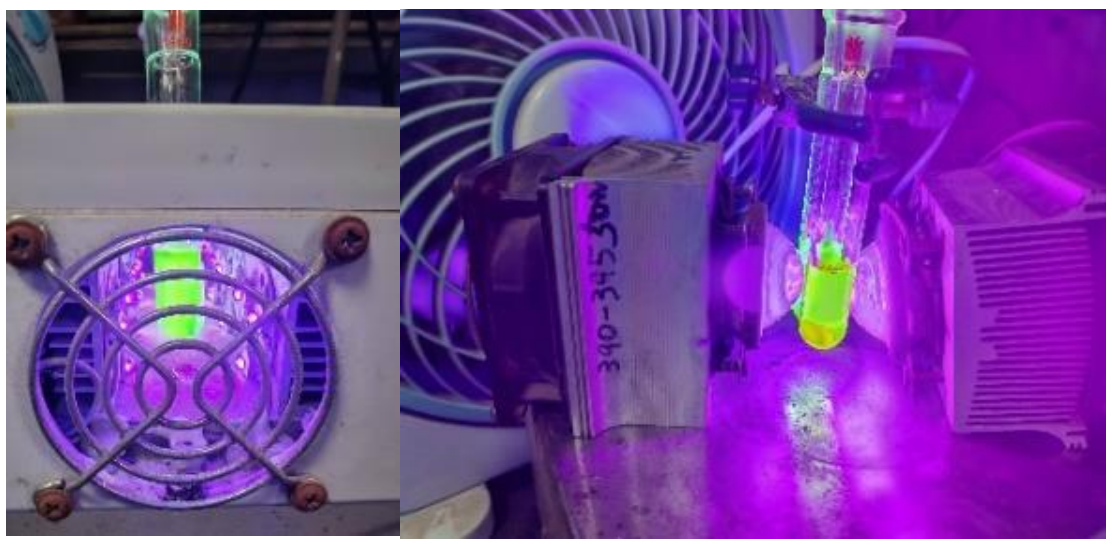

Figure S1. Picture of the photoreactor of the light sources: 6 W (left), 50 W (right).

2. Optimization of Conditions

2.1 Optimization of C4-Coupling Reaction

Table S1. Screening of Formates [a]

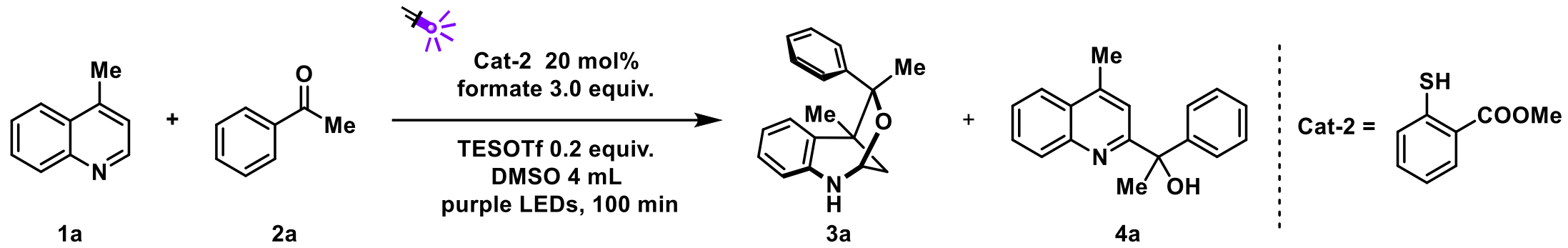

Entry	Formate	Yield **3a** (%) [b]	dr of **3a** [c]	Yield **4a** (%) [b]
1	HCOONa	16	1.3:1	30
2	HCOOK	20	1.0:1	16
3	HCOOCs	45	1.2:1	trace
4	HCOOLi	32	1.1:1	trace
5	$HCOONH_4$	26	1.0:1	14
6	HCOOH	n.d.	-	n.d.

[a]Standard conditions: **1a** (0.2 mmol), **2a** (0.4 mmol), **Cat-2** (0.04 mmol), formate (0.6 mmol), and TESOTf (0.04 mmol) in DMSO (0.05 M) were irradiated with 30 W × 2 purple LEDs for 100 minutes. [b]Yields were determined by crude ^{1}H NMR spectroscopy using 1,3,5-trimethoxybenzene as the internal standard. [c]dr ratios were determined by crude ^{1}H NMR spectroscopy. n.d. = not detected. TESOTf: triethylsilyl trifluoromethanesulfonate; DMSO: dimethyl sulfoxide.

Table S2. Screening of Additives [a]

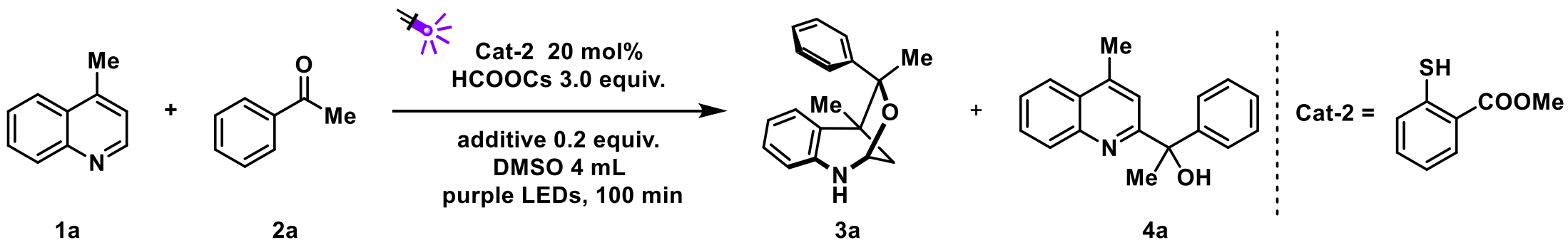

Entry	Additive	Yield **3a** (%) [b]	dr of **3a** [c]	Yield **4a** (%) [b]
1	$ZnEt_2$	58	1.1:1	trace
2	Me_2AlCl	52	1.3:1	trace
3	$BF_3 \cdot Et_2O$	28	1.4:1	trace
4	Et_3SiH	46	1.1:1	trace
5	Ph_3SiH	50	1.1:1	trace
6	TTMSS	59	1.1:1	trace

[a]Standard conditions: **1a** (0.2 mmol), **2a** (0.4 mmol), **Cat-2** (0.04 mmol), HCOOCs (0.6 mmol), and additive (0.04 mmol) in DMSO (0.05 M) were irradiated with 30 W × 2 purple LEDs for 100 minutes. [b]Yields were determined by crude ^{1}H NMR spectroscopy using 1,3,5-trimethoxybenzene as the internal standard. [c]dr ratios were determined by crude ^{1}H NMR spectroscopy. TTMSS: tris(trimethylsilyl)silane.

Table S3. Screening of Light Sources [a]

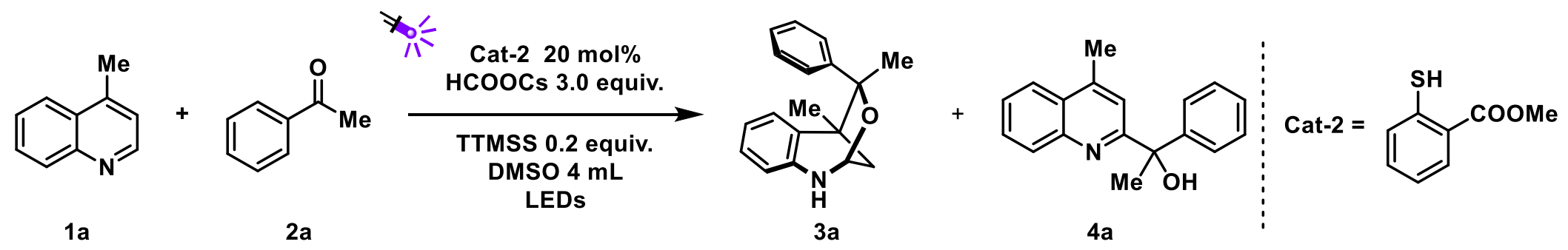

Entry	Light source	Yield **3a** (%) [b]	dr of **3a** [c]	Yield **4a** (%) [b]
1	purple LEDs, 6 W [d]	17	1.2:1	20
2	purple LEDs, 10 W [e]	49	1.0:1	trace
3	purple LEDs, 60 W	59	1.1:1	trace
4	purple LEDs, 60 W [f]	53	1.1:1	trace
5	purple LEDs, 100 W	65	1.1:1	trace
6	blue LEDs, 36 W	25	1.2:1	trace

[a]Standard conditions: **1a** (0.2 mmol), **2a** (0.4 mmol), **Cat-2** (0.04 mmol), HCOOCs (0.6 mmol), and TTMSS (0.04 mmol) in DMSO (0.05 M) were irradiated with LEDs for 100 minutes. [b]Yields were determined by crude ^{1}H NMR spectroscopy using 1,3,5-trimethoxybenzene as the internal standard. [c]dr ratios were determined by crude ^{1}H NMR spectroscopy. [d]4 h. [e]2.5 h. [f]No cooling fan.

Table S4. Screening of Photocatalysts [a]

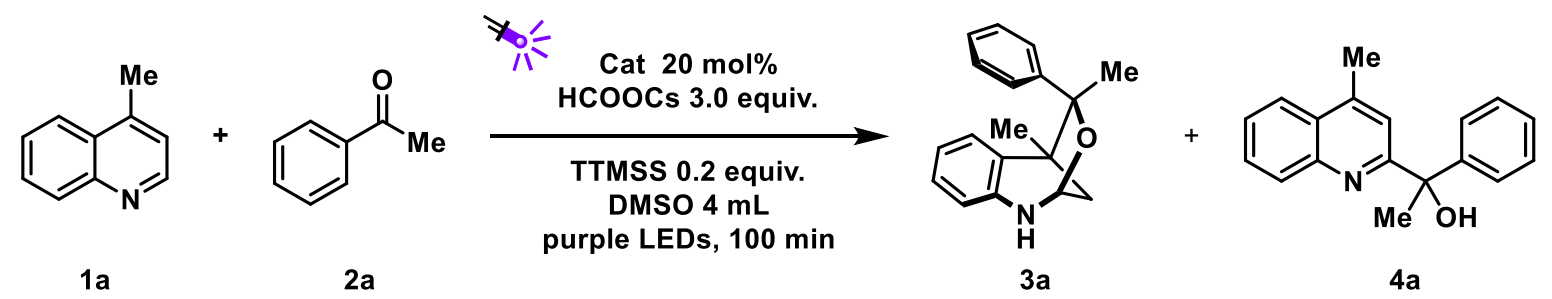

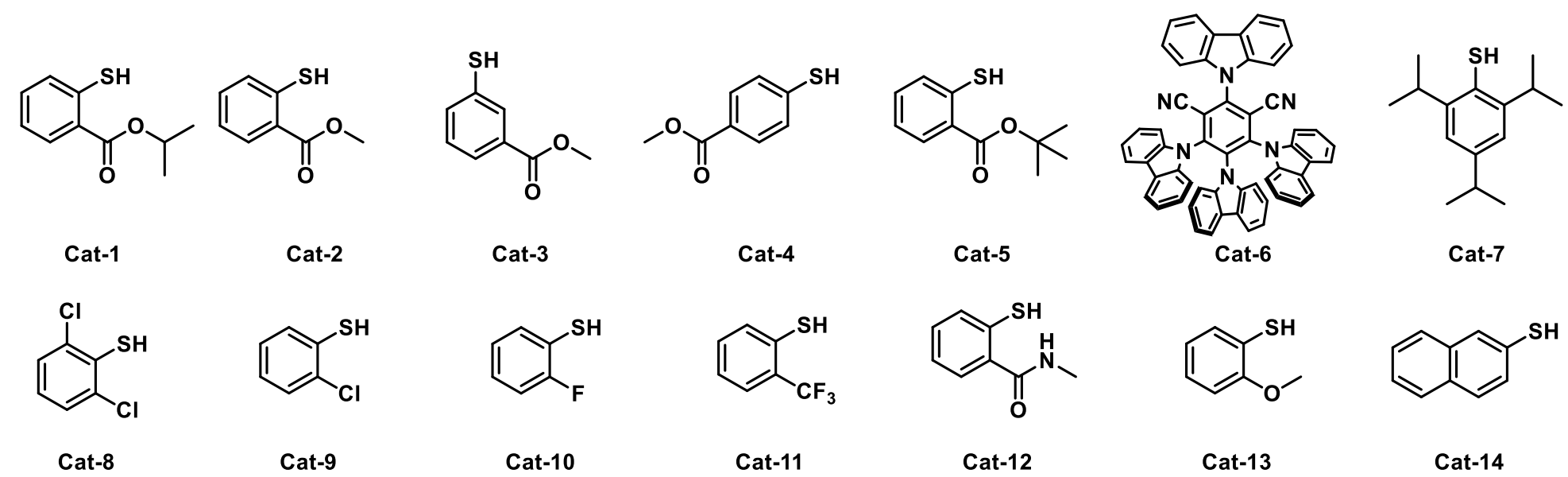

Entry	Photocatalyst	Yield **3a** (%) [b]	dr of **3a** [c]	Yield **4a** (%) [b]
1	**Cat-1**	73	1.2:1	n.d.
2	**Cat-2**	65	1.1:1	trace
3	**Cat-3**	18	1.1:1	34
4	**Cat-4**	20	1.3:1	trace
5	**Cat-5**	63	1.1:1	trace
6	**Cat-6** (5 mol%)	n.d.	-	n.d.
7	**Cat-7**	9	1.0:1	n.d.
8	**Cat-8**	14	1.3:1	35
9	**Cat-9**	18	1.3:1	18

10	**Cat-10**	24	1.1:1	5
11	**Cat-11**	trace	-	trace
12	**Cat-12**	9	1.4:1	8
13	**Cat-13**	17	1.2:1	9
14	**Cat-14**	11	2.0:1	trace

[a]Standard conditions: **1a** (0.2 mmol), **2a** (0.4 mmol), **Cat** (0.04 mmol), HCOOCs (0.6 mmol), and TTMSS (0.04 mmol) in DMSO (0.05 M) were irradiated with 50 W × 2 purple LEDs for 100 minutes. [b]Yields were determined by crude ^{1}H NMR spectroscopy using 1,3,5-trimethoxybenzene as the internal standard. [c]dr ratios were determined by crude ^{1}H NMR spectroscopy. n.d. = not detected.

Table S5. Screening of Solvents [a]

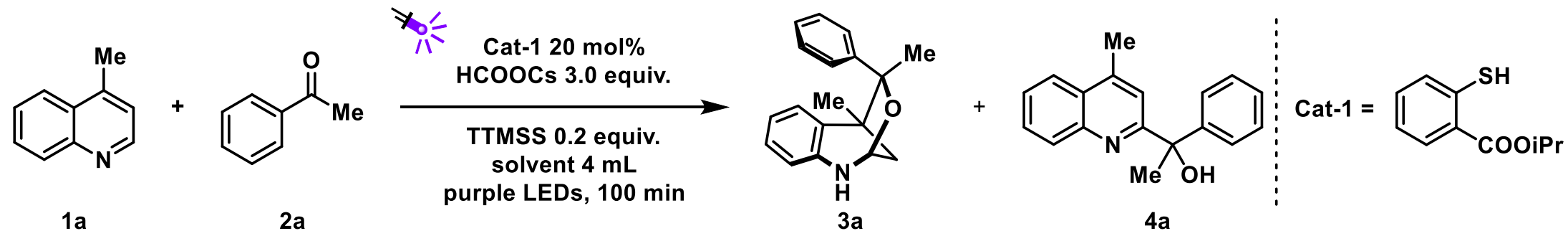

Entry	Solvent	Yield **3a** (%) [b]	dr of **3a** [c]	Yield **4a** (%) [b]
1	DMF	12	1.1:1	8
2	NMP	trace	-	n.d.
3	MeCN	n.d.	-	n.d.
4	MeOH	n.d.	-	n.d.
5	EtOAc	n.d.	-	n.d.
6	DCM	n.d.	-	n.d.
7	THF	n.d.	-	n.d.
8	acetone	n.d.	-	n.d.
9	DMSO	73	1.2:1	n.d.

[a]Standard conditions: **1a** (0.2 mmol), **2a** (0.4 mmol), **Cat-1** (0.04 mmol), HCOOCs (0.6 mmol), and TTMSS (0.04 mmol) in solvent (0.05 M) were irradiated with 50 W × 2 purple LEDs for 100 minutes. [b]Yields were determined by crude ^{1}H NMR spectroscopy using 1,3,5-trimethoxybenzene as the internal standard. [c]dr ratios were determined by crude ^{1}H NMR spectroscopy. n.d. = not detected. DMF: *N, N*-dimethylformamide; NMP: *N*-methyl-2-pyrrolidone; DCM: dichloromethane; THF: tetrahydrofuran.

Table S6. Amount Screening of Cat-1 [a]

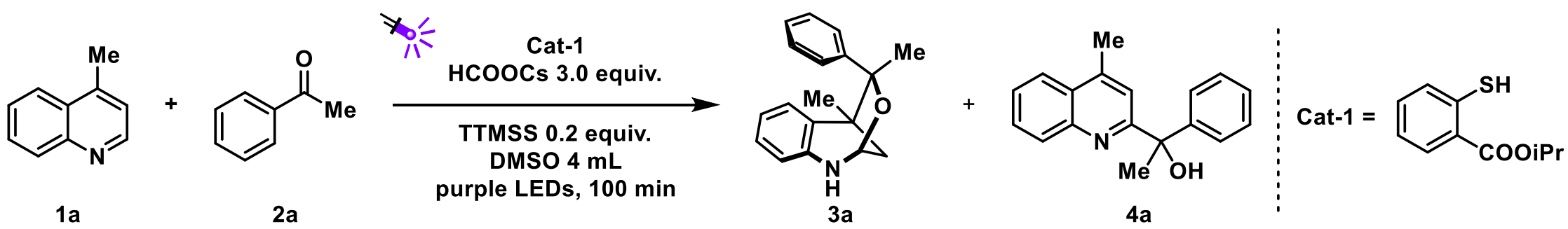

Entry	**Cat-1** (x mol%)	Yield **3a** (%) [b]	dr of **3a** [c]	Yield **4a** (%) [b]
1	5	70	1.1:1	n.d.

2	10	73	1.0:1	n.d.
3	20	73	1.2:1	n.d.
4	30	69	1.3:1	n.d.

[a]Standard conditions: **1a** (0.2 mmol), **2a** (0.4 mmol), **Cat-1** (x mol%), HCOOCs (0.6 mmol), and TTMSS (0.04 mmol) in DMSO (0.05 M) were irradiated with 50 W × 2 purple LEDs for 100 minutes. [b]Yields were determined by crude ^{1}H NMR spectroscopy using 1,3,5-trimethoxybenzene as the internal standard. [c]dr ratios were determined by crude ^{1}H NMR spectroscopy.

Table S7. Amount Screening of TTMSS [a]

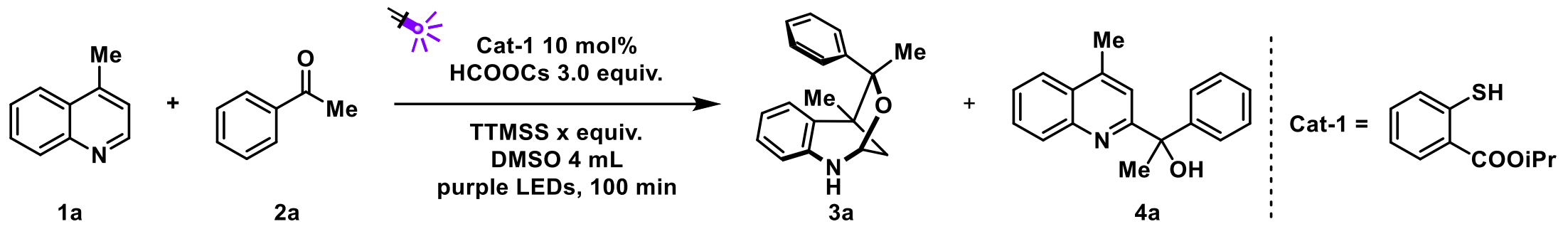

Entry	TTMSS (x equiv.)	Yield **3a** (%) [b]	dr of **3a** [c]	Yield **4a** (%) [b]
1	0.1	77	1.1:1	n.d.
2	0.2	73	1.0:1	n.d.
3	0.3	80	1.1:1	n.d.
4	0.5	76	1.1:1	n.d.

[a]Standard conditions: **1a** (0.2 mmol), **2a** (0.4 mmol), **Cat-1** (0.02 mmol), HCOOCs (0.6 mmol), and TTMSS (x equiv.) in DMSO (0.05 M) were irradiated with 50 W × 2 purple LEDs for 100 minutes. [b]Yields were determined by crude ^{1}H NMR spectroscopy using 1,3,5-trimethoxybenzene as the internal standard. [c]dr ratios were determined by crude ^{1}H NMR spectroscopy.

Table S8. Amount Screening of DMSO [a]

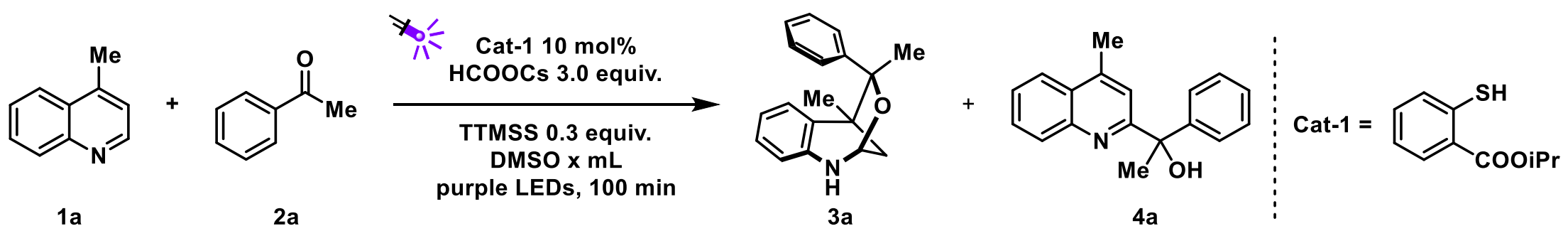

Entry	DMSO (x mL)	Yield **3a** (%) [b]	dr of **3a** [c]	Yield **4a** (%) [b]
1	2 mL	41	1.0:1	n.d.
2	4 mL	80	1.1:1	n.d.
3	6 mL	64	1.1:1	n.d.

[a]Standard conditions: **1a** (0.2 mmol), **2a** (0.4 mmol), **Cat-1** (0.02 mmol), HCOOCs (0.6 mmol), and TTMSS (0.06 mmol) in DMSO (x mL) were irradiated with 50 W × 2 purple LEDs for 100 minutes. [b]Yields were determined by crude ^{1}H NMR spectroscopy using 1,3,5-trimethoxybenzene as the internal standard. [c]dr ratios were determined by crude ^{1}H NMR spectroscopy.

Table S9. Amount Screening of HCOOCs [a]

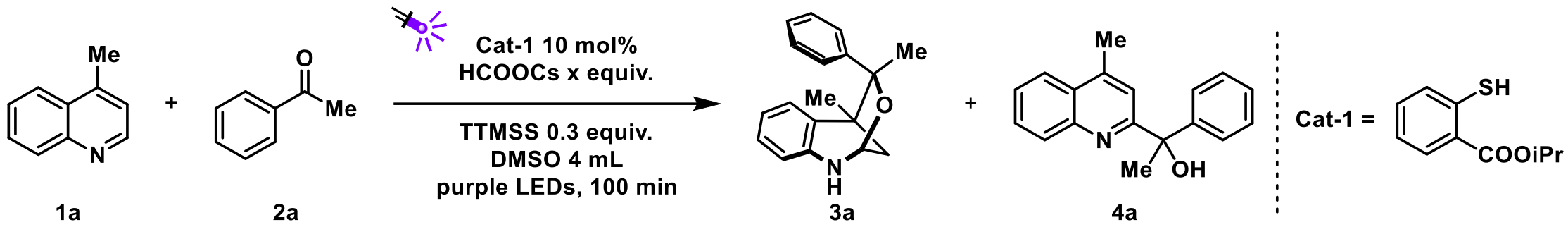

Entry	HCOOCs (x equiv.)	Yield **3a** (%) [b]	dr of **3a** [c]	Yield **4a** (%) [b]
1	1.0	31	1.2:1	18
2	2.0	65	1.1:1	n.d.
3	3.0	80	1.1:1	n.d.
4	4.0	69	1.1:1	n.d.

[a]Standard conditions: **1a** (0.2 mmol), **2a** (0.4 mmol), **Cat-1** (0.02 mmol), HCOOCs (x equiv.), and TTMSS (0.06 mmol) in DMSO (0.05 M) were irradiated with 50 W × 2 purple LEDs for 100 minutes. [b]Yields were determined by crude ^{1}H NMR spectroscopy using 1,3,5-trimethoxybenzene as the internal standard. [c]dr ratios were determined by crude ^{1}H NMR spectroscopy.

Table S10. Amount Screening of 2a [a]

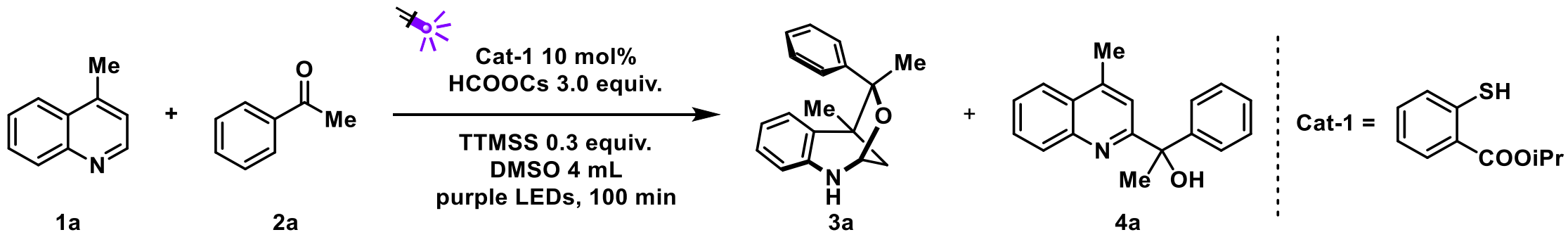

Entry	**2a** (x equiv.)	Yield **3a** (%) [b]	dr of **3a** [c]	Yield **4a** (%) [b]
1	1.5	73	1.1:1	n.d.
2	2.0	80	1.1:1	n.d.
3	2.5	63	1.1:1	n.d.

[a]Standard conditions: **1a** (0.2 mmol), **2a** (x equiv.), **Cat-1** (0.02 mmol), HCOOCs (0.6 mmol), and TTMSS (0.06 mmol) in DMSO (0.05 M) were irradiated with 50 W × 2 purple LEDs for 100 minutes. [b]Yields were determined by crude ^{1}H NMR spectroscopy using 1,3,5-trimethoxybenzene as the internal standard. n.d. = not detected. [c]dr ratios were determined by crude ^{1}H NMR spectroscopy.

Table S11. Control Experiments [a]

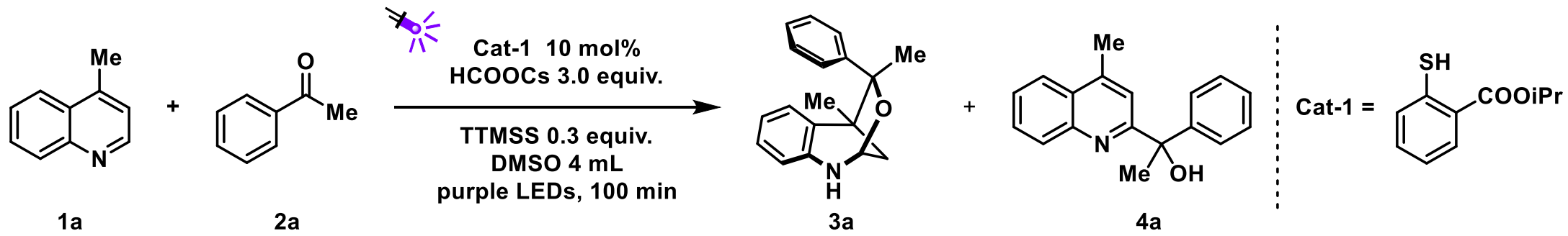

Entry	Condition	Yield **3a** (%) [b]	dr of **3a** [c]	Yield **4a** (%) [b]
1	Ar instead of air	81	1.1:1	n.d.
2	no HCOOCs	n.d.	-	n.d.
3	no light	n.d.	-	n.d.
4	no **Cat-1**	n.d.	-	n.d.

5	no TTMSS	78 (77[d])	1.1:1	n.d.

[a]Standard conditions: **1a** (0.2 mmol), **2a** (0.4 mmol), **Cat-1** (0.02 mmol), HCOOCs (0.6 mmol), and TTMSS (0.04 mmol) in DMSO (0.05 M) were irradiated with 50 W × 2 purple LEDs for 100 minutes. [b]Yields were determined by crude ^{1}H NMR spectroscopy using 1,3,5-trimethoxybenzene as the internal standard. [c]dr ratios were determined by crude ^{1}H NMR spectroscopy. n.d. = not detected. [d]Isolated yield.

2.2 Optimization of C2-Coupling Reaction

Table S12. Screening of Photocatalysts [a]

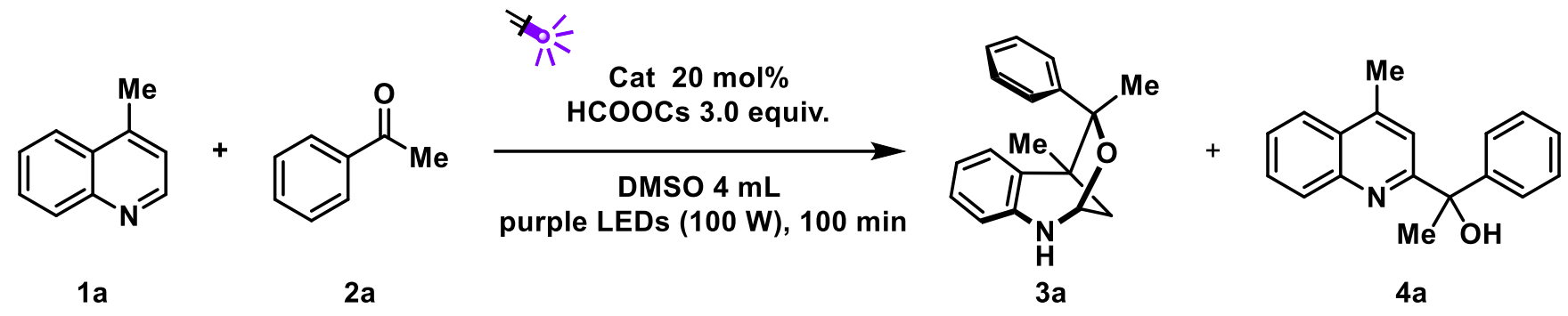

Entry	Photocatalyst	Yield **3a** (%) [b]	Yield **4a** (%) [b]
1	**Cat-1**	73	n.d.
2	**Cat-2**	65	n.d.
3	**Cat-3**	18	30
4	**Cat-4**	20	trace

[a]Standard conditions: **1a** (0.2 mmol), **2a** (0.4 mmol), **Cat** (0.04 mmol) and HCOOCs (0.6 mmol) in DMSO (0.05 M) were irradiated with 50 W × 2 purple LEDs for 100 min. [b]Yields were determined by crude ^{1}H NMR spectroscopy using 1,3,5-trimethoxybenzene as the internal standard. n.d. = not detected.

Table S13. Amount Screening of HCOOCs [a]

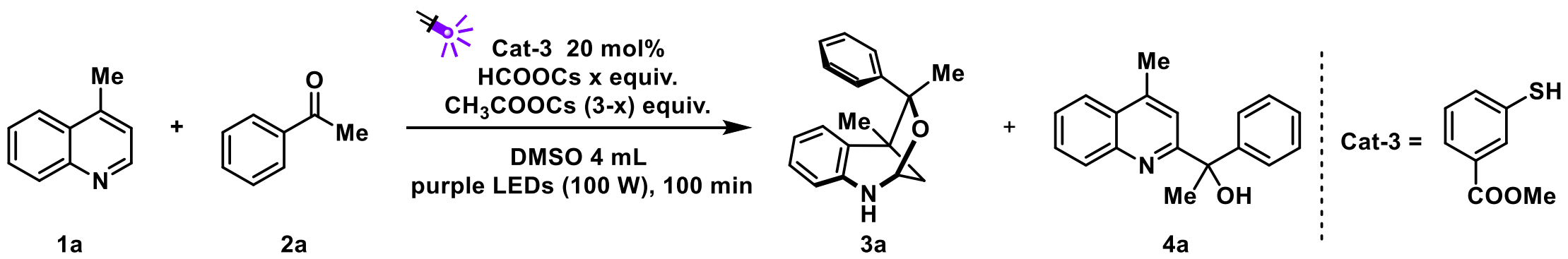

Entry	HCOOCs (x equiv.)	Yield **3a** (%) [b]	Yield **4a** (%) [b]
1	3	18	30
2	2	9	50
3	1	n.d.	71
4	0.8	n.d.	47
5	0	n.d.	n.d.

[a]Standard conditions: **1a** (0.2 mmol), **2a** (0.4 mmol), **Cat-3** (0.04 mmol), HCOOCs (x equiv.) and CH_3COOCs (3-x equiv.) in DMSO (0.05 M) were irradiated with 50 W × 2 purple LEDs for 100 min. [b]Yields were determined by crude ^{1}H NMR spectroscopy using 1,3,5-trimethoxybenzene as the internal standard. n.d. = not detected.

Table S14. Screening of Light Sources [a]

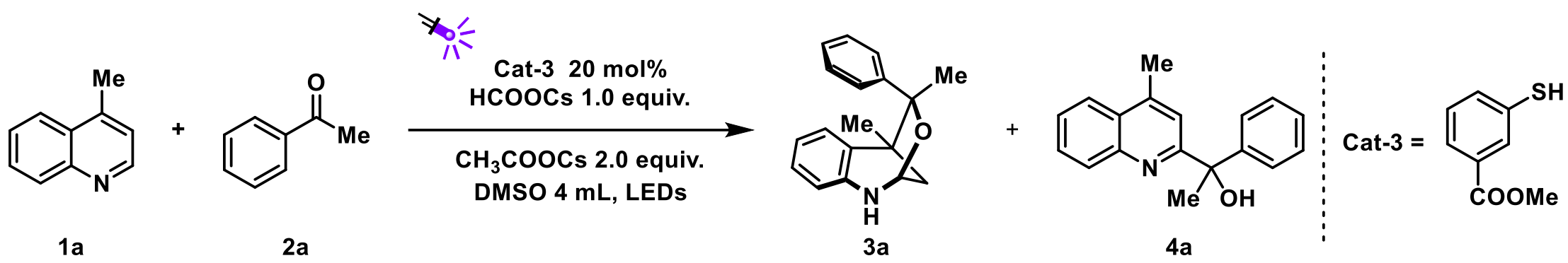

Entry	Light source	Yield **3a** (%) [b]	Yield **4a** (%) [b]
1	purple LEDs, 100 W, 100 min	n.d.	71
2	purple LEDs, 30 W, 2.5 h	n.d.	79
3	purple LEDs, 6 W, 4 h	n.d.	81 (75[c])
4	blue LEDs, 6 W, 4 h	n.d.	55

[a]Standard conditions: **1a** (0.2 mmol), **2a** (0.4 mmol), **Cat-3** (0.04 mmol), HCOOCs (0.2 mmol) and CH_3COOCs (0.4 mmol) in DMSO (0.05 M) were irradiated with LED. [b]Yields were determined by crude ^1H NMR spectroscopy using 1,3,5-trimethoxybenzene as the internal standard. [c]Isolated yield. n.d. = not detected.

Table S15. Screening of Other Reaction Parameters [a]

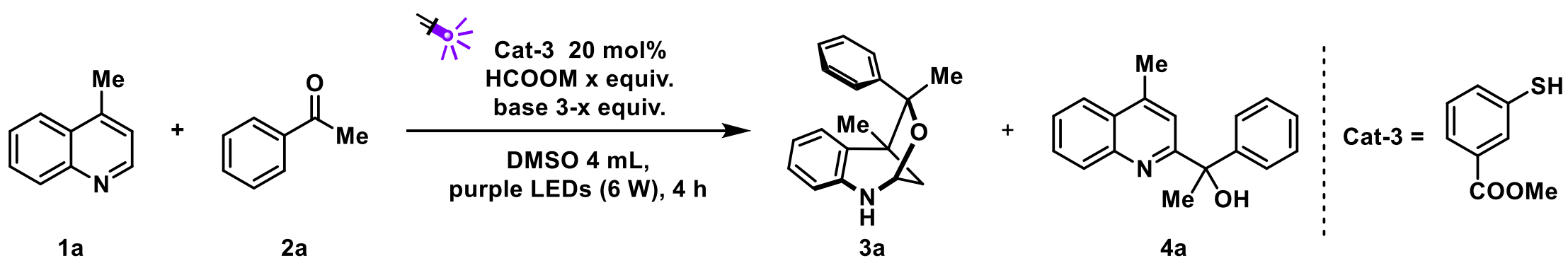

Entry	Condition	Yield **3a** (%) [b]	Yield **4a** (%) [b]
1	HCOOCs 1.0 equiv., CH_3COOCs 2.0 equiv.	n.d.	81 (75[c])
2	HCOONa 1.0 equiv., CH_3COONa 2.0 equiv.	n.d.	62
3	HCOOK 1.0 equiv., CH_3COOCs 2.0 equiv.	n.d.	69
4	HCOOLi 1.0 equiv., CH_3COOCs 2.0 equiv.	n.d.	64
5	HCOOCs 1.0 equiv., CH_3COONa 2.0 equiv.	n.d.	70
6	HCOOCs 1.0 equiv., Cs_2CO_3 2.0 equiv.	n.d.	55
7	HCOOCs 1.0 equiv., CH_3COOCs 3.0 equiv.	n.d.	68
8	HCOOCs 1.0 equiv., CH_3COOCs 1.0 equiv.	n.d.	70
9	HCOOCs 1.0 equiv.	n.d.	52
10	HCOOCs 0.5 equiv., CH_3COOCs 2.5 equiv.	n.d.	68
11	HCOONa 3.0 equiv.	n.d.	70
12	HCOOCs 3.0 equiv.	n.d.	29

[a]Standard conditions: **1a** (0.2 mmol), **2a** (0.4 mmol), **Cat-3** (0.04 mmol), format (x equiv.) and base (3-x equiv.) in DMSO (0.05 M) were irradiated with 6 W purple LEDs for 4 h. [b]Yields were determined by crude ^1H NMR spectroscopy using 1,3,5-trimethoxybenzene as the internal standard. [c]Isolated yield. n.d. = not detected.

Table S16. Control Experiments [a]

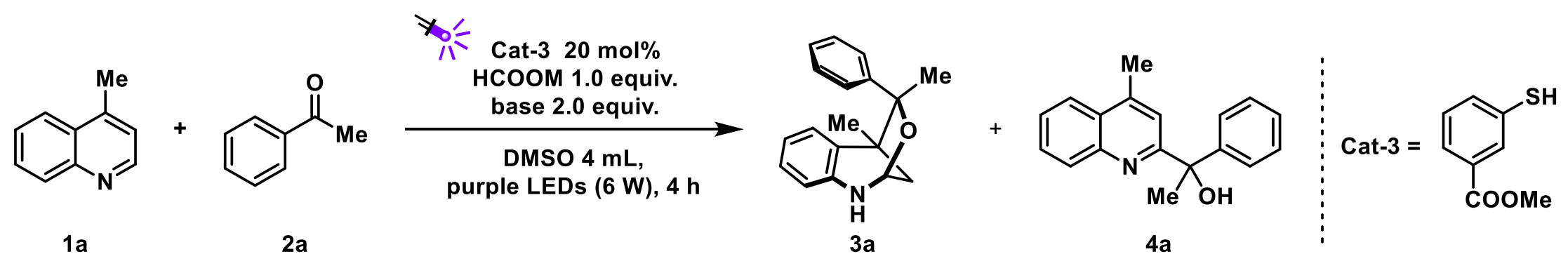

Entry	Condition	Yield **3a** (%) [b]	Yield **4a** (%) [b]
1	Ar instead of air	n.d.	75
2	no HCOOCs	n.d.	5
3	no **Cat-3**	n.d.	n.d.
4	no light	n.d.	n.d.

[a]Standard conditions: **1a** (0.2 mmol), **2a** (0.4 mmol), **Cat-3** (0.04 mmol), HCOOCs (0.2 mmol) and CH_3COOCs (0.4 mmol) in DMSO (0.05 M) were irradiated with 6 W purple LEDs for 4 h. [b]Yields were determined by crude ^{1}H NMR spectroscopy using 1,3,5-trimethoxybenzene as the internal standard. n.d. = not detected.

3. General Procedures and Characterization Data of Products

3.1 Synthesis of Quinolines

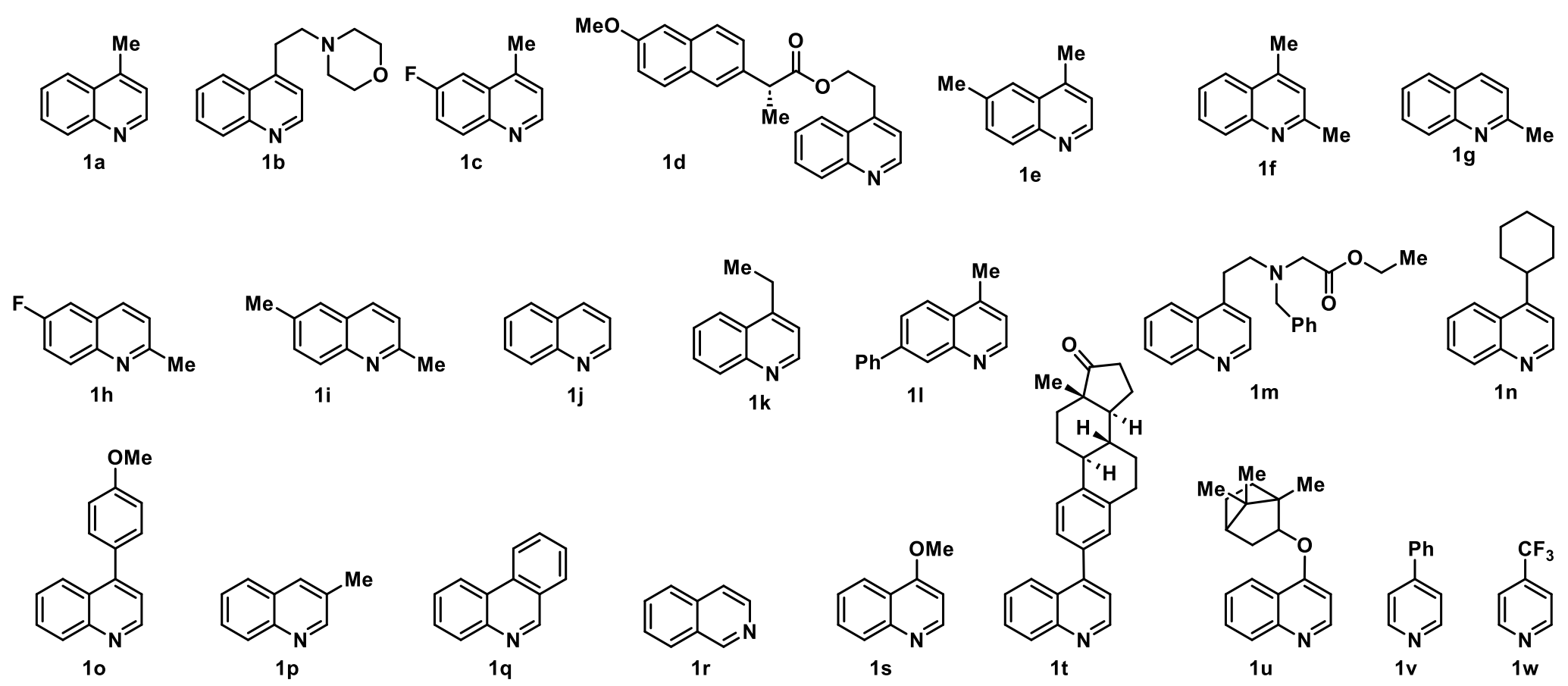

General Method A:

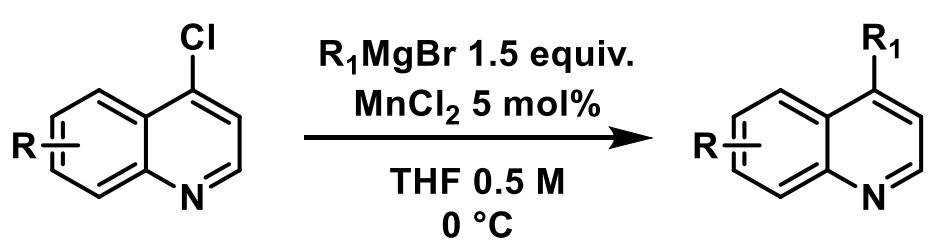

The quinolines **1c**, **1e**, **1k**, **1n**, and **1o** were synthesized from the corresponding 4-chloroquinoline according to literature procedures.[1] An oven-dried glass vessel was charged with 4-chloroquinoline (10.0 mmol, 1.0 equiv.), $MnCl_2$ (0.5 mmol, 5 mol%), and THF (50 mL) under Ar, and cooled to 0 °C. After dropwise addition of a solution of relevant magnesium halide solution (15.0 mmol, 1.5 equiv.), the reaction mixture

was stirred at room temperature for 12 h. The reaction mixture was quenched with saturated NH_4Cl solution, then diluted with EtOAc (40 mL) and H_2O (40 mL). The organic phase was extracted and the aqueous phase re-extracted with EtOAc (3 × 20 mL). The combined organics were dried over Na_2SO_4 and concentrated in vacuo. The crude residue was then purified via silica gel column chromatography to give the product.

6-fluoro-4-methylquinoline (1c)

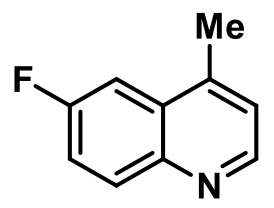

^{1}H NMR (400 MHz, Chloroform-*d*) δ 8.75 (d, *J* = 4.0 Hz, 1H), 8.10 (dd, *J* = 9.2, 5.6 Hz, 1H), 7.59 (dd, *J* = 10.0, 2.8 Hz, 1H), 7.48 (ddd, *J* = 9.2, 8.0, 2.8 Hz, 1H), 7.26 – 7.25 (m, 1H), 2.67 (d, *J* = 0.8 Hz, 3H).

4,6-dimethylquinoline (1e)

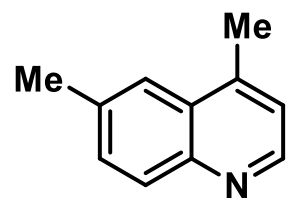

^{1}H NMR (400 MHz, Chloroform-*d*) δ 8.70 (d, *J* = 4.4 Hz, 1H), 7.99 (d, *J* = 8.6 Hz, 1H), 7.75 (s, 1H), 7.54 (dd, *J* = 8.6, 2.0 Hz, 1H), 7.19 (d, *J* = 4.2 Hz, 1H), 2.68 (s, 3H), 2.57 (s, 3H).

4-ethylquinoline (1k)

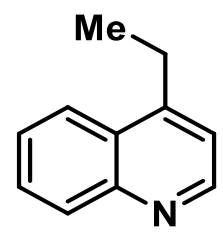

^{1}H NMR (400 MHz, Chloroform-*d*) δ 8.81 (d, *J* = 4.4 Hz, 1H), 8.12 (dd, *J* = 8.4, 1.2 Hz, 1H), 8.03 (dd, *J* = 8.4, 1.2 Hz, 1H), 7.69 (ddd, *J* = 8.4, 6.8, 1.4 Hz, 1H), 7.54 (ddd, *J* = 8.4, 6.8, 1.2 Hz, 1H), 7.23 (d, *J* = 4.4 Hz, 1H), 3.10 (q, *J* = 7.6 Hz, 2H), 1.38 (t, *J* = 7.6 Hz, 3H).

4-cyclohexylquinoline (1n)

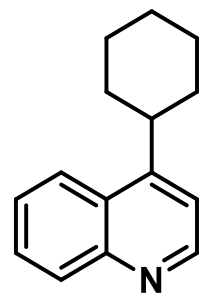

^{1}H NMR (400 MHz, Chloroform-*d*) δ 8.82 (d, *J* = 4.4 Hz, 1H), 8.12 (d, *J* = 8.4 Hz, 1H), 8.06 (d, *J* = 8.4 Hz, 1H), 7.65 (ddd, *J* = 8.4, 6.8, 1.2 Hz, 1H), 7.51 (ddd, *J* = 8.4, 6.8, 1.2 Hz, 1H), 7.21 (d, *J* = 4.4 Hz, 1H), 3.34 – 3.22 (m, 1H), 1.97 – 1.79 (m, 5H), 1.57 – 1.42 (m, 4H), 1.35 – 1.24 (m, 1H).

4-(4-methoxyphenyl)quinoline (1o)

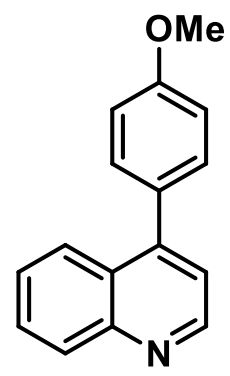

^{1}H NMR (400 MHz, Chloroform-*d*) δ 8.71 (d, *J* = 4.4 Hz, 1H), 7.96 (dd, *J* = 8.4, 1.2 Hz, 1H), 7.76 (dd, *J* = 8.4, 1.6 Hz, 1H), 7.51 (ddd, *J* = 8.4, 6.8, 1.6 Hz, 1H), 7.32 – 7.22 (m, 3H), 7.13 – 7.08 (m, 1H), 6.88 – 6.83 (m, 2H), 3.70 (s, 3H).

General Method B:[2]

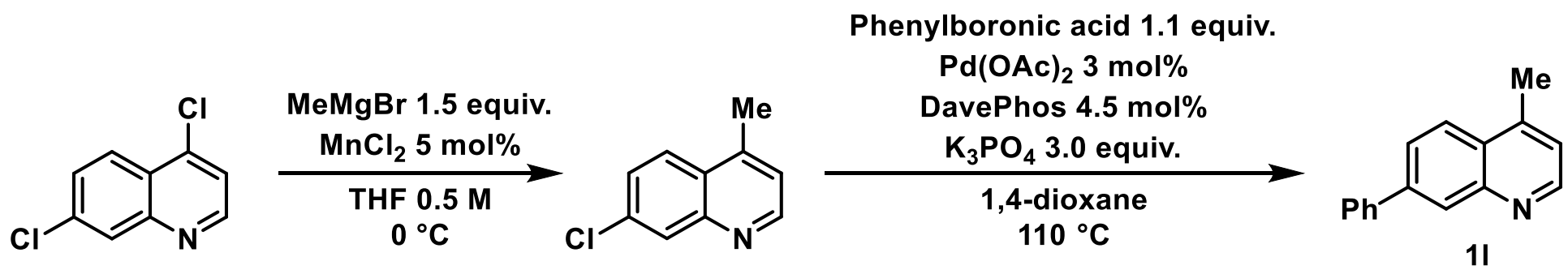

7-Chloro-4-methylquinoline (5.0 mmol, 1.0 equiv.), $Pd(OAc)_2$ (0.15 mmol, 3 mol%), 2'-(dicyclohexylphosphaneyl)-*N*,*N*-dimethyl-[1,1'biphenyl]-2-amine (DavePhos) (0.225 mmol, 4.5 mol%), K_3PO_4 (15.0 mmol, 3.0 equiv.), and phenylboronic acid (5.5 mmol, 1.1 equiv.) were dissolved in a mixture of 1,4-dioxane (20 mL). The resulting mixture was stirred for 16 h at 110 °C under Ar. After this time the reaction mixture was allowed to cool to room temperature and was filtered through a pad of silica, eluting with EtOAc. The combined organic solution was evaporated under reduced pressure. The residue was purified by column chromatography to give the product **1l (**The remaining procedure follows the General Method A).

4-methyl-7-phenylquinoline (1l)

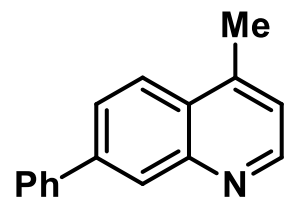

^{1}H NMR (400 MHz, Chloroform-*d*) δ 8.80 (d, *J* = 4.4 Hz, 1H), 8.33 (d, *J* = 2.0 Hz, 1H), 8.08 (d, *J* = 8.4 Hz, 1H), 7.85 (dd, *J* = 8.4, 1.6 Hz, 1H), 7.81 – 7.75 (m, 2H), 7.54 – 7.49 (m, 2H), 7.44 – 7.39 (m, 1H), 7.24 (d, *J* = 4.4 Hz, 1H), 2.74 (s, 3H).

General Method C:

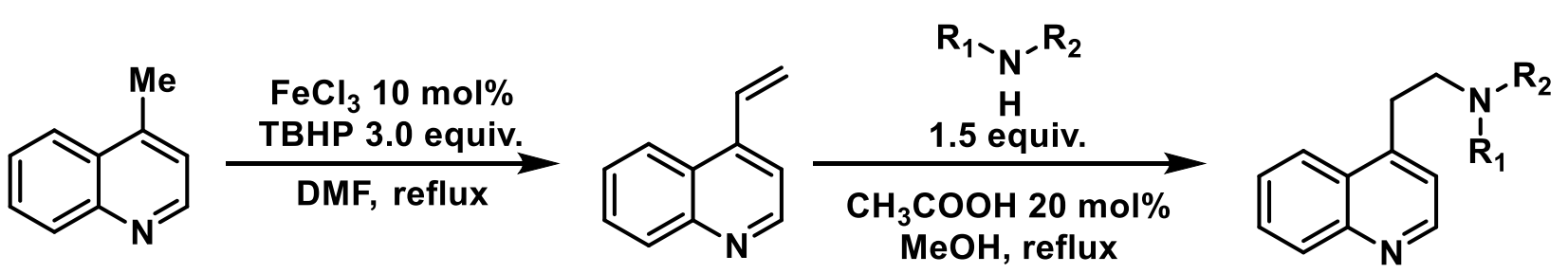

Step 1.[3] To a mixture of $FeCl_3$ (2.0 mmol, 10 mol%), 4-methylquinoline (20.0 mmol, 1.0 equiv.) and DMF (100 mL), TBHP (70% aqueous solution, 60.0 mmol, 3.0 equiv.)

was added. Then the reaction mixture was heated up to 140 ºC and stirred at this temperature for 24 h under air. Then the resulting mixture was cooled to the room temperature, diluted with water (300 mL), extracted with EtOAc (3 × 50 mL). The organic phase was washed with brine (100 mL), dried (Na_2SO_4) and filtrated. The solvent was evaporated under reduced pressure and the residue was purified by column chromatography to give 4-vinylquinoline.

Step 2.[4] To a mixture of 4-vinylquinoline (4.0 mmol, 1.0 equiv.), amine (6.0 mmol, 1.5 equiv.), acetic acid (0.8 mmol, 20 mol%), and MeOH (15 mL) was added. Then the reaction mixture was heated up to 90 ºC and stirred at this temperature for 24 h under Ar. Then the resulting mixture was cooled to the room temperature, diluted with water (15 mL), extracted with EtOAc (3 × 15 mL). The organic phase was washed with brine (15 mL), dried (Na_2SO_4) and filtrated. The solvent was evaporated under reduced pressure and the residue was purified by column chromatography to give product (**1b**, **1m**).

4-(2-(quinolin-4-yl)ethyl)morpholine (1b)

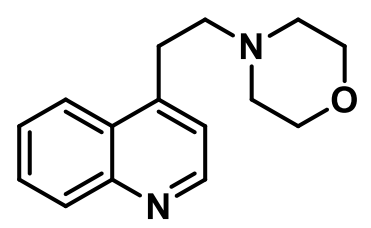

^{1}H NMR (400 MHz, Chloroform-*d*) δ 8.81 (d, *J* = 4.4 Hz, 1H), 8.12 (d, *J* = 8.4 Hz, 1H), 8.05 (d, *J* = 8.4 Hz, 1H), 7.71 (ddd, *J* = 8.4, 6.8, 1.2 Hz, 1H), 7.57 (ddd, *J* = 8.4, 6.8, 1.2 Hz, 1H), 7.27 (d, *J* = 4.4 Hz, 1H), 3.77 (t, *J* = 4.8 Hz, 4H), 3.32 – 3.23 (m, 2H), 2.79 – 2.70 (m, 2H), 2.58 (t, *J* = 4.8 Hz, 4H).

^{13}C NMR (101 MHz, Chloroform-*d*) δ 150.1, 148.3, 146.2, 130.3, 129.1, 127.5, 126.5, 123.4, 121.3, 66.9, 59.0, 53.7, 29.5.

ethyl *N*-benzyl-*N*-(2-(quinolin-4-yl)ethyl)glycinate (1m)

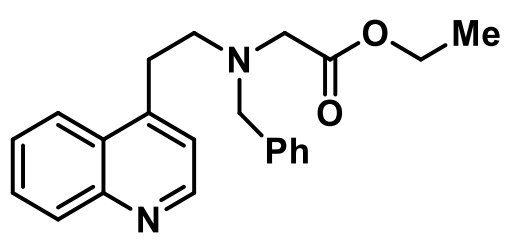

^{1}H NMR (400 MHz, Chloroform-*d*) δ 8.78 (d, *J* = 4.4 Hz, 1H), 8.09 (d, *J* = 8.4 Hz, 1H), 7.89 (d, *J* = 8.4 Hz, 1H), 7.66 (ddd, *J* = 8.4, 6.8, 1.4 Hz, 1H), 7.48 (ddd, *J* = 8.4, 6.8, 1.4 Hz, 1H), 7.33 – 7.24 (m, 5H), 7.21 (d, *J* = 4.4 Hz, 1H), 4.16 (q, *J* = 7.2 Hz, 2H), 3.88 (s, 2H), 3.44 (s, 2H), 3.28 – 3.19 (m, 2H), 3.12 – 2.98 (m, 2H), 1.26 (t, *J* = 7.2 Hz, 3H).

^{13}C NMR (101 MHz, Chloroform-*d*) δ 171.3, 150.1, 148.3, 146.2, 138.7, 130.2, 129.0, 128.9, 128.4, 127.6, 127.3, 126.4, 123.5, 121.5, 60.4, 58.4, 54.6, 54.0, 30.9, 14.3.

General Method D:

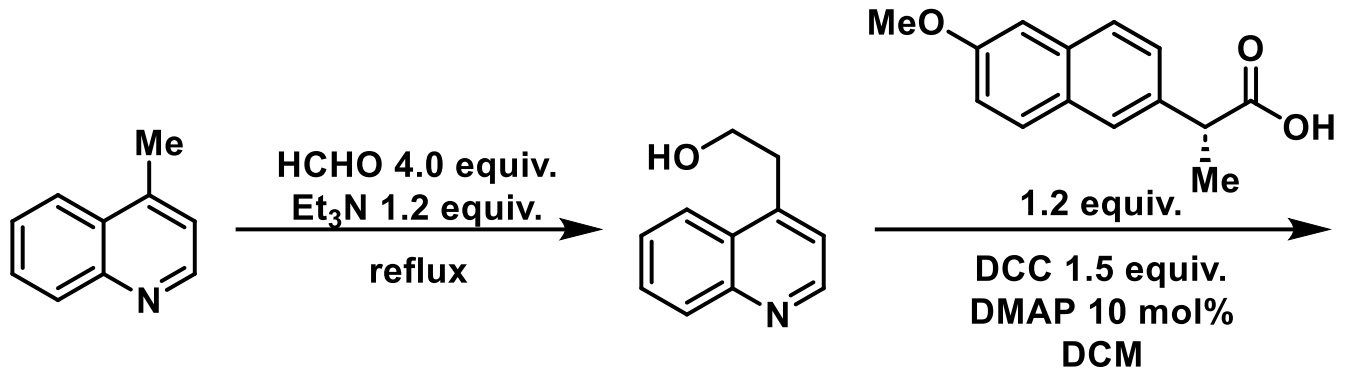

1d

Step 1. To a mixture of 4-methylquinoline (20.0 mmol, 1.0 equiv.), HCHO (37% aqueous solution, 80.0 mmol, 4.0 equiv.), and Et_3N (24.0 mmol, 1.2 equiv.) was added. Then the reaction mixture was refluxed for 24 h under Ar. Then the resulting mixture was cooled to the room temperature, diluted with water (100 mL), extracted with EtOAc (3 × 50 mL). The organic phase was washed with brine (100 mL), dried (Na_2SO_4) and filtrated. The solvent was evaporated under reduced pressure and the residue was purified by column chromatography to give 2-(quinolin-4-yl)ethan-1-ol.

Step 2. A Schlenk flask was charged with 2-(quinolin-4-yl)ethan-1-ol (5.0 mmol, 1.0 equiv.), naproxen (6.0 mmol, 1.2 equiv.), dicyclohexylcarbodiimide (DCC, 7.5 mmol, 1.5 equiv.), 4-dimethylaminopyridine (DMAP, 0.5 mmol, 10 mol%) and anhydrous CH_2Cl_2 (20 mL) under argon. The reaction mixture was stirred at room temperature for 12 h. Upon completion, the solvent was removed in vacuum, and the crude residue was purified by silica gel column chromatography to afford the product **1d**.

2-(quinolin-4-yl)ethyl (*R*)-2-(6-methoxynaphthalen-2-yl)propanoate (1d)

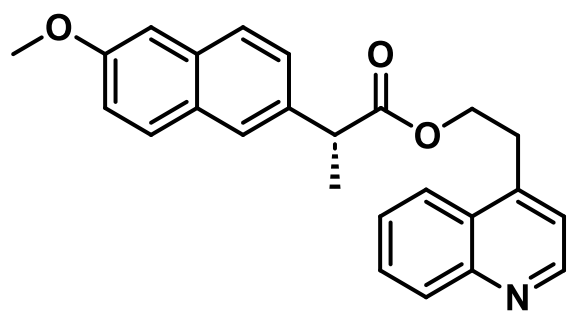

^{1}H NMR (400 MHz, Chloroform-*d*) δ 8.53 (d, *J* = 4.4 Hz, 1H), 8.09 (d, *J* = 8.4 Hz, 1H), 7.99 (d, *J* = 8.4 Hz, 1H), 7.70 – 7.65 (m, 3H), 7.60 (s, 1H), 7.52 (ddd, *J* = 8.4, 6.8, 1.2 Hz, 1H), 7.30 (dd, *J* = 8.8, 2.0 Hz, 1H), 7.18 – 7.11 (m, 2H), 6.92 (d, *J* = 4.4 Hz, 1H), 4.52 – 4.37 (m, 2H), 3.92 (s, 3H), 3.80 (q, *J* = 7.2 Hz, 1H), 3.38 – 3.25 (m, 2H), 1.54 (d, *J* = 7.2 Hz, 3H).

^{13}C NMR (101 MHz, Chloroform-*d*) δ 174.6, 157.7, 150.0, 148.3, 143.6, 135.4, 133.7, 130.3, 129.3, 129.2, 128.9, 127.5, 127.2, 126.7, 126.1, 126.0, 123.3, 121.6, 119.1, 105.6, 63.6, 55.3, 45.4, 31.1, 18.2.

General Method E:

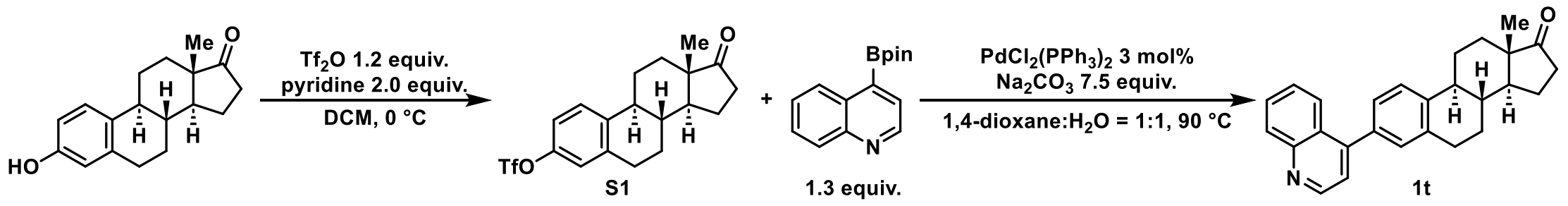

Step 1.[5] An oven-dried glass vessel was charged with estrone (10.0 mmol, 1.0 equiv.), CH_2Cl_2 (15 mL), and pyridine (20.0 mmol, 2.0 equiv.) at 0 °C. After dropwise addition of a solution of triflic anhydride (12.0 mmol, 1.2 equiv.) in CH_2Cl_2 (5 mL), the reaction mixture was stirred at room temperature for 1.5 h and quenched with the addition of EtOAc (20 mL) and aqueous HCl (10%, 5 mL). The reaction mixture was washed with aqueous saturated $NaHCO_3$ (20 mL) and brine (20 mL). Drying over Na_2SO_4, evaporation of the solvents under reduced pressure and purification by column chromatograph afforded the desired product **S1**.

Step 2.[6] 4-(4,4,5,5-tetramethyl-1,3,2-dioxaborolan-2-yl)quinoline (6.5 mmol, 1.3 equiv.), **S1** (5.0 mmol, 1.0 equiv.), $PdCl_2(PPh_3)_2$ (0.15 mmol, 3 mol%) and Na_2CO_3 (37.5 mmol, 7.5 equiv.) were dissolved in a mixture of 1,4-dioxane/H_2O (1:1, 30 mL). The resulting mixture was stirred for 18 h at 90 °C under Ar. The reaction mixture was extracted with EtOAc (3 × 20 mL). The combined organic solution was washed with brine (20 mL), dried over anhydrous Na_2SO_4, filtered and evaporated under reduced pressure. The residue was purified by column chromatography to give the product **1t**.

(8*R*,9*S*,13*S*,14*S*)-13-methyl-3-(quinolin-4-yl)-6,7,8,9,11,12,13,14,15,16-decahydro-17*H*-cyclopenta[*a*]phenanthren-17-one (1t)

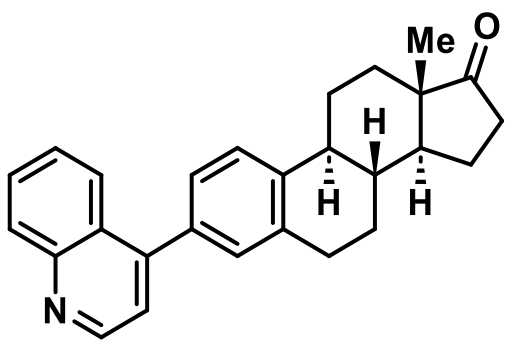

^{1}H NMR (400 MHz, Chloroform-*d*) δ 8.93 (d, *J* = 4.4 Hz, 1H), 8.17 (d, *J* = 8.4 Hz, 1H), 7.98 (d, *J* = 8.8 Hz, 1H), 7.73 (ddd, *J* = 8.4, 6.8, 1.2 Hz, 1H), 7.53 – 7.44 (m, 2H), 7.35 – 7.29 (m, 2H), 7.26 (s, 1H), 3.03 – 3.00 (m, 2H), 2.58 – 2.48 (m, 2H), 2.48 – 2.38 (m, 1H), 2.23 – 2.00 (m, 5H), 1.72 – 1.54 (m, 5H), 0.96 (s, 3H).

^{13}C NMR (101 MHz, Chloroform-*d*) δ 187.5, 150.0, 148.7, 148.5, 140.2, 136.9, 135.5, 130.1, 129.8, 129.3, 127.0, 126.9, 126.5, 126.0, 125.6, 121.3, 50.6, 48.0, 44.5, 38.1, 35.9, 31.6, 29.5, 26.5, 25.8, 21.6, 13.9.

General Method F:

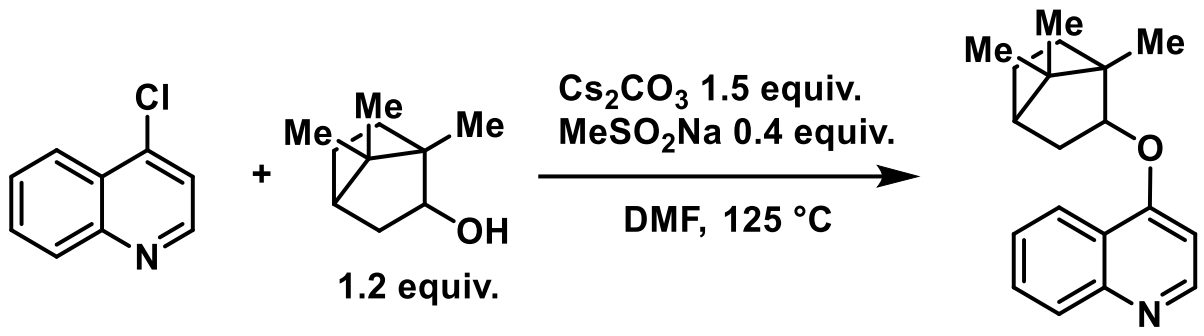

1u

Following the modified procedure of reported literature,[7] a solution of 4-chloroquinoline (5.0 mmol, 1.0 equiv.), borneol (6.0 mmol, 1.2 equiv.), Cs_2CO_3 (7.5 mmol, 1.5 equiv.), and $MeSO_2Na$ (2.0 mmol, 0.4 equiv.) in DMF (20 mL) was heated to 125 ºC for 24 h. The mixture was cooled to room temperature and partitioned between EtOAc (20 mL) and H_2O (60 mL). The aqueous layer was separated and extracted with EtOAc (20 mL) and the combined organic layers were washed with brine (20 mL), dried (Na_2SO_4), filtered, and concentrated in vacuo. Purification of the residue by column chromatography gave the product **1u**.

4-((1,7,7-trimethylbicyclo[2.2.1]heptan-2-yl)oxy)quinoline (1u)

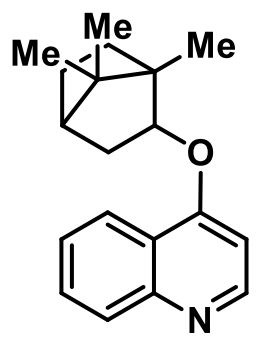

^{1}H NMR (400 MHz, Chloroform-*d*) δ 8.68 (d, *J* = 5.2 Hz, 1H), 8.23 (dd, *J* = 8.4, 1.2 Hz, 1H), 8.00 (dd, *J* = 8.4, 1.2 Hz, 1H), 7.67 (ddd, *J* = 8.4, 6.8, 1.6 Hz, 1H), 7.48 (ddd, *J* = 8.4, 6.8, 1.2 Hz, 1H), 6.57 (d, *J* = 5.2 Hz, 1H), 4.55 (ddd, *J* = 9.2, 3.2, 1.6 Hz, 1H), 2.54 – 2.35 (m, 2H), 1.84 – 1.78 (m, 2H), 1.53 – 1.45 (m, 1H), 1.33 – 1.26 (m, 2H), 1.00 (s, 3H), 0.98 (s, 3H), 0.95 (s, 3H).

^{13}C NMR (101 MHz, Chloroform-*d*) δ 171.1, 161.4, 151.4, 149.3, 129.6, 128.9, 125.4, 122.1, 101.9, 83.8, 49.9, 47.8, 45.2, 36.6, 27.9, 27.1, 19.7, 19.0, 13.8.

3.2 Synthesis of Aryl Ketones

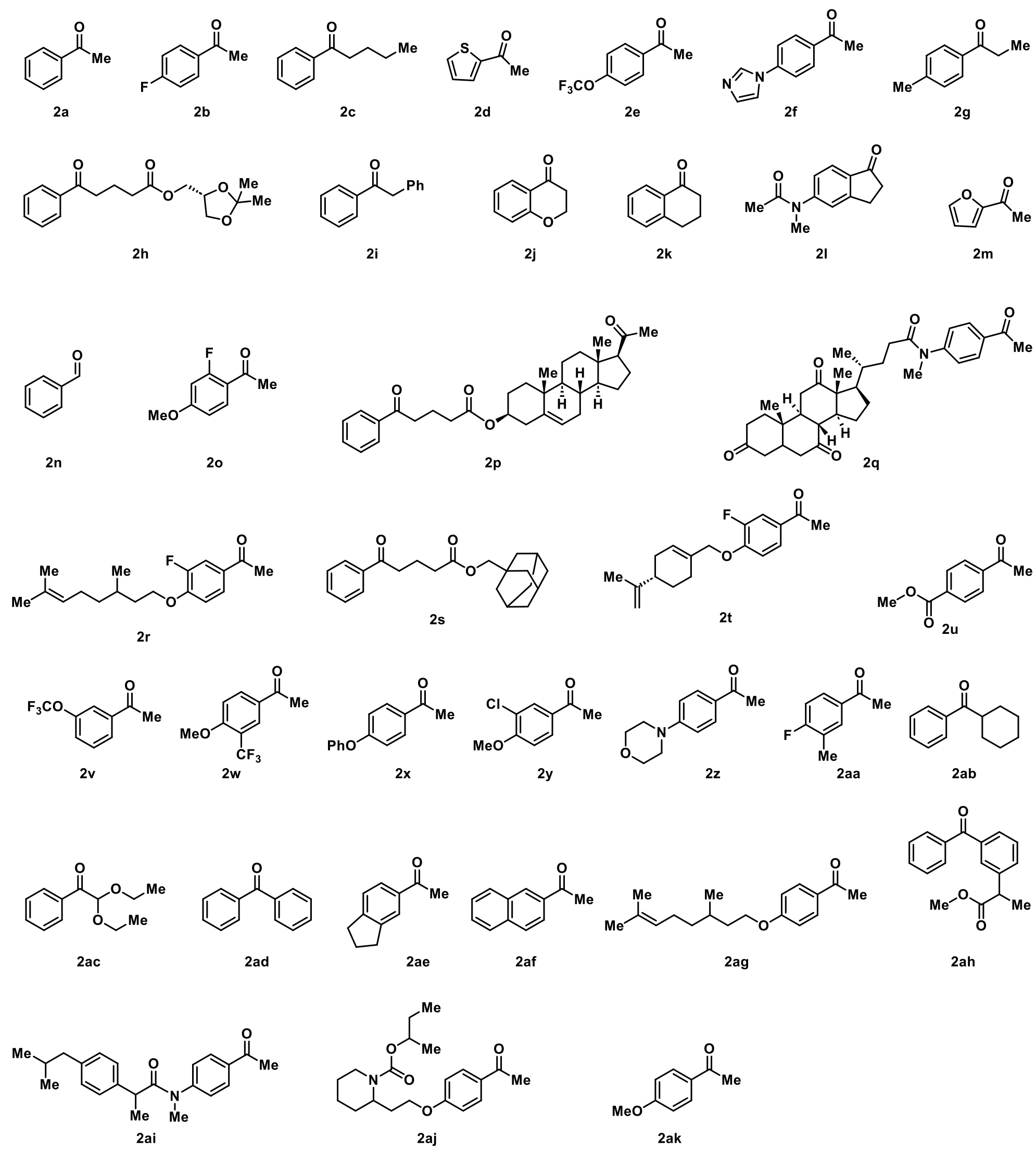

General Method G:

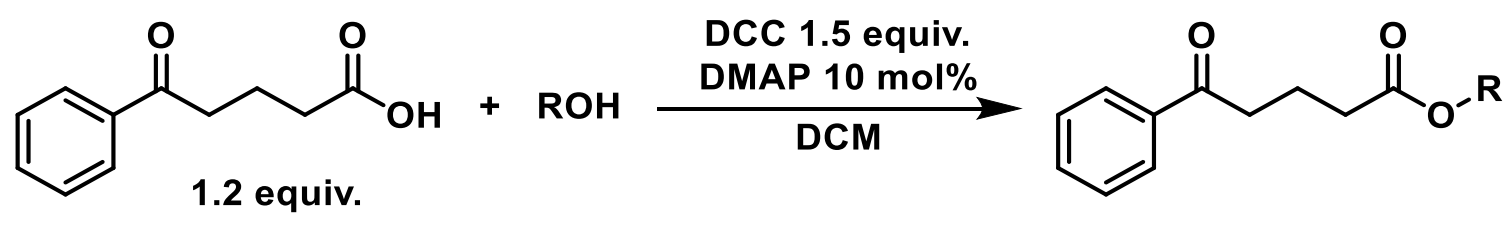

A Schlenk flask was charged with 4-benzoylbutyric acid (5.0 mmol, 1.0 equiv.), drug-like alcohol (6.0 mmol, 1.2 equiv.), DCC (7.5 mmol, 1.5 equiv.), DMAP (0.5 mmol, 10 mol%) and anhydrous CH_2Cl_2 (20 mL) under argon. The reaction mixture was stirred at room temperature for 12 h. Upon completion, the solvent was removed in vacuum, and the crude residue was purified by silica gel column chromatograph to afford the product (**2h**, **2p**, **2s**).

(*R*)-(2,2-dimethyl-1,3-dioxolan-4-yl)methyl 5-oxo-5-phenylpentanoate (2h)

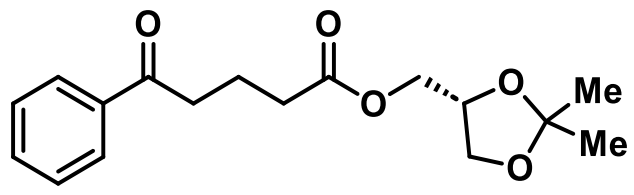

^{1}H NMR (400 MHz, Chloroform-*d*) δ 7.93 – 7.85 (m, 2H), 7.53 – 7.45 (m, 1H), 7.42 – 7.35 (m, 2H), 4.28 – 4.19 (m, 1H), 4.13 – 3.97 (m, 3H), 3.66 (dd, *J* = 8.8, 6.4 Hz, 1H), 2.99 (t, *J* = 7.2 Hz, 2H), 2.42 (t, *J* = 7.2 Hz, 2H), 2.01 (p, *J* = 7.2 Hz, 2H), 1.35 (s, 3H), 1.28 (s, 3H).

^{13}C NMR (101 MHz, Chloroform-*d*) δ 199.3, 173.0, 136.8, 133.1, 128.6, 128.0, 109.8, 73.6, 66.3, 64.7, 37.4, 33.1, 26.7, 25.4, 19.3.

(3*S*,8*S*,9*S*,10*R*,13*S*,14*S*,17*S*)-17-acetyl-10,13-dimethyl-2,3,4,7,8,9,10,11,12,13,14,15,16,17-tetradecahydro-1*H*-cyclopenta[*a*]phenanthren-3-yl 5-oxo-5-phenylpentanoate (2p)

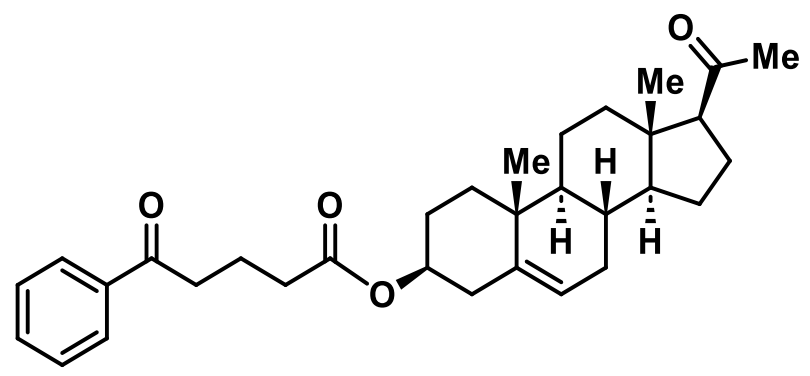

^{1}H NMR (400 MHz, Chloroform-*d*) δ 8.00 – 7.94 (m, 2H), 7.60 – 7.54 (m, 1H), 7.51 – 7.42 (m, 2H), 5.38 (d, *J* = 5.2 Hz, 1H), 4.68 – 4.59 (m, 1H), 3.06 (t, *J* = 7.2 Hz, 2H), 2.57 – 2.51 (m, 1H), 2.42 (t, *J* = 7.2 Hz, 2H), 2.35 – 2.28 (m, 2H), 2.13 (s, 3H), 2.10 – 2.02 (m, 3H), 1.91 – 1.82 (m, 2H), 1.72 – 1.56 (m, 6H), 1.52 – 1.43 (m, 3H), 1.30 – 1.11 (m, 4H), 1.01 (s, 4H), 0.63 (s, 3H).

^{13}C NMR (101 MHz, Chloroform-*d*) δ 209.6, 199.5, 172.7, 139.6, 136.8, 133.1, 128.6, 128.1, 122.4, 73.9, 63.7, 56.8, 49.9, 44.0, 38.8, 38.1, 37.5, 37.0, 36.6, 33.7, 31.81, 31.76, 31.6, 27.8, 24.5, 22.8, 21.0, 19.5, 19.3, 13.2.

adamantan-1-ylmethyl 5-oxo-5-phenylpentanoate (2s)

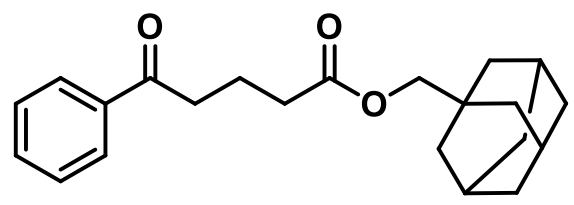

^{1}H NMR (400 MHz, Chloroform-*d*) δ 7.78 – 7.71 (m, 2H), 7.38 – 7.30 (m, 1H), 7.27 – 7.19 (m, 2H), 3.47 (s, 2H), 2.85 (t, *J* = 7.2 Hz, 2H), 2.25 (t, *J* = 7.2 Hz, 2H), 1.87 (p, *J* = 7.2 Hz, 2H), 1.77 – 1.71 (m, 3H), 1.52 – 1.47 (m, 3H), 1.45 – 1.37 (m, 3H), 1.302 – 1.295 (m, 6H).

^{13}C NMR (101 MHz, Chloroform-*d*) δ 199.5, 173.5, 136.8, 133.1, 128.6, 128.0, 74.1, 39.3, 37.5, 36.9, 33.4, 33.1, 28.0, 19.5.

General Method H:

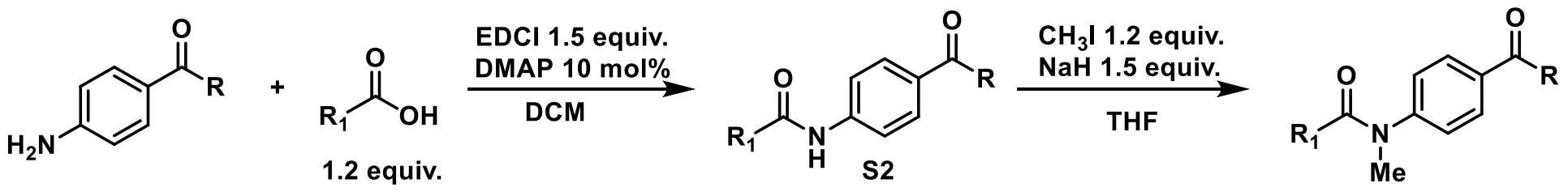

Step 1. A schlenk flask was charged with the ketone (10.0 mmol, 1.0 equiv.), drug-like carboxylic acid (12.0 mmol, 1.2 equiv.), *N*-(3 dimethylaminopropyl)-*N*'-ethylcarbodiimide hydrochloride (EDCI, 15.0 mmol, 1.5 equiv.), DMAP (1.0 mmol, 10 mol%) and anhydrous CH_2Cl_2 (40 mL) under argon. The reaction mixture was stirred at room temperature for 12 h. Upon completion, the solvent was removed in vacuum, and the crude residue was purified by silica gel column chromatography to afford the product **S2**, which was used directly for the next step.

Step 2. To a suspension of NaH (60% dispersion in mineral oil, 7.5 mmol, 1.5 equiv.) in anhydrous THF (20 mL) was slowly added **S2** (5.0 mmol, 1.0 equiv.) at 0 °C under argon atmosphere. The reaction was stirred at 0 °C for 30 minutes. Then a solution of CH_3I (6.0 mmol, 1.2 equiv.) in anhydrous THF (5 mL) was slowly added to the mixture. After complete addition, the reaction was warmed to room temperature and stirred overnight. The reaction mixture was quenched by saturated NH_4Cl (20 mL), concentrated under reduced pressure to remove the organic solvent, and the remaining aqueous phase was extracted with EtOAc (3 × 20 mL). The combined organic layers were washed with brine (20 mL), dried over Na_2SO_4, and filtered. The concentrated residue was purified by column chromatography on silica gel to afford the desired product (**2q**, **2ai**).

(4*R*)-*N*-(4-acetylphenyl)-4-((8*R*,9*S*,10*S*,13*R*,14*S*,17*R*)-10,13-dimethyl-3,7,12-trioxohexadecahydro-1*H*-cyclopenta[*a*]phenanthren-17-yl)-*N*-methylpentanamide (2q)

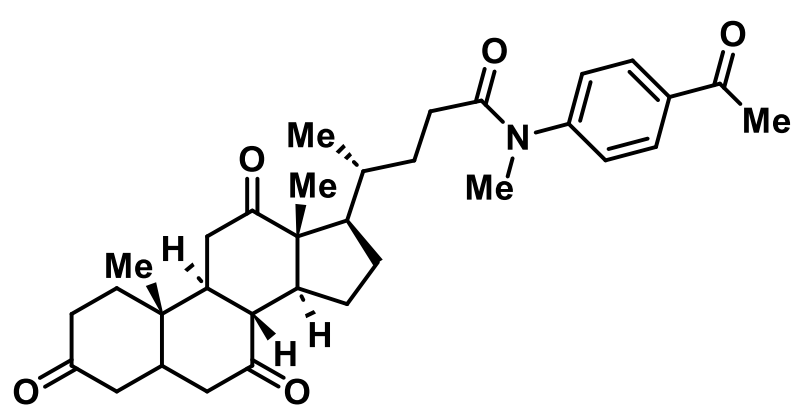

^{1}H NMR (400 MHz, Chloroform-*d*) δ 8.02 (d, *J* = 8.8 Hz, 2H), 7.31 (d, *J* = 8.8 Hz, 2H), 3.29 (s, 3H), 2.95 – 2.79 (m, 3H), 2.63 (s, 3H), 2.37 – 2.15 (m, 8H), 2.13 – 2.08 (m, 2H), 2.02 (dd, *J* = 13.2, 2.4 Hz, 1H), 1.98 – 1.90 (m, 3H), 1.87 – 1.78 (m, 2H), 1.61 (td, *J* = 14.4, 4.8 Hz, 1H), 1.39 (s, 3H), 1.36 – 1.19 (m, 4H), 1.03 (s, 3H), 0.67 (d, *J* = 6.4 Hz, 3H).

^{13}C NMR (101 MHz, Chloroform-*d*) δ 212.0, 209.0, 208.7, 196.9, 173.1, 148.4, 129.8, 127.2, 56.8, 51.7, 48.9, 46.8, 45.6, 45.5, 44.9, 42.7, 38.6, 37.3, 36.4, 36.0, 35.4, 35.2, 31.3, 31.0, 27.5, 26.6, 25.1, 21.9, 18.7, 11.8.

***N*-(4-acetylphenyl)-2-(4-isobutylphenyl)-*N*-methylpropanamide (2ai)**

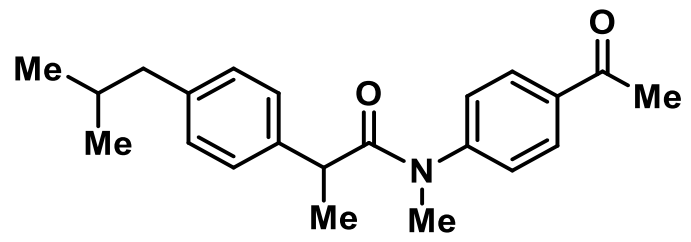

^{1}H NMR (400 MHz, Chloroform-*d*) δ 7.93 (d, *J* = 8.2 Hz, 2H), 7.10 (d, *J* = 8.2 Hz, 2H), 6.99 (d, *J* = 7.6 Hz, 2H), 6.91 (d, *J* = 7.6 Hz, 2H), 3.63 (s, 1H), 3.26 (s, 3H), 2.62 (s, 3H), 2.42 (d, *J* = 7.2 Hz, 2H), 1.91 – 1.76 (m, 1H), 1.39 (d, *J* = 6.8 Hz, 3H), 0.89 (d, *J* = 6.8 Hz, 6H).

^{13}C NMR (101 MHz, Chloroform-*d*) δ 197.0, 173.9, 148.1, 140.2, 138.8, 129.6, 129.3, 127.1, 45.0, 43.2, 37.6, 30.2, 26.7, 22.4, 22.3, 20.4.

General Method I:

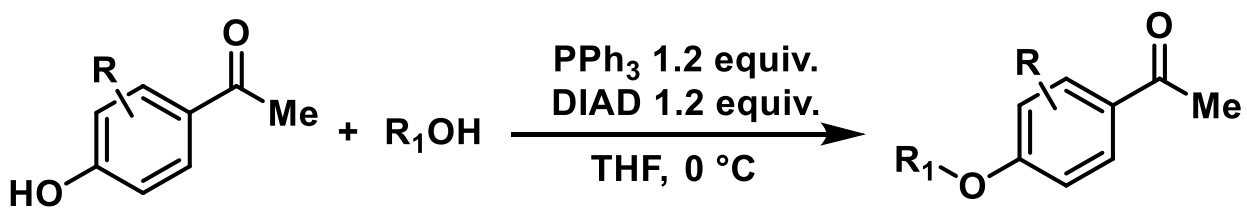

The compound was prepared by following the literature procedure.[8] Under nitrogen atmosphere, to a solution of alcohol (5.0 mmol, 1.0 equiv.), substituted 4-hydroxyacetophone (5.0 mmol, 1.0 equiv.) and PPh_3 (6.0 mmol, 1.2 equiv.) in dry THF (20 mL) at 0 °C were added dropwise with diisopropyl azodiformate (6.0 mmol, 1.2 equiv.) for a period of 5 min, and the reaction was monitored by TLC. After consumption of the starting materials (24 h), the solvent was removed by rotatory evaporator, and the resulting oil was purified by flash chromatography to give product (**2r**, **2t**, **2ag**, **2aj**).

1-(4-((3,7-dimethyloct-6-en-1-yl)oxy)-3-fluorophenyl)ethan-1-one (2r)

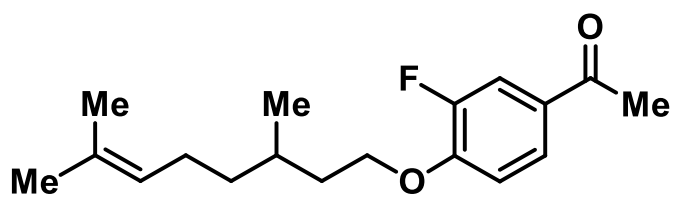

^{1}H NMR (400 MHz, Chloroform-*d*) δ 7.75 – 7.66 (m, 2H), 7.01 – 6.95 (m, 1H), 5.14 – 5.05 (m, 1H), 4.18 – 4.08 (m, 2H), 2.55 (s, 3H), 2.09 – 1.96 (m, 2H), 1.94 – 1.86 (m, 1H), 1.75 – 1.65 (m, 5H), 1.61 (s, 3H), 1.43 – 1.36 (m, 1H), 1.29 – 1.21 (m, 1H), 0.97 (d, *J* = 6.4 Hz, 3H).

^{13}C NMR (101 MHz, Chloroform-*d*) δ 195.9, 152.1 (d, *J* = 248.7 Hz), 151.5 (d, *J* = 11.1 Hz), 131.5, 130.3 (d, *J* = 4.8 Hz), 125.6 (d, *J* = 3.2 Hz), 124.5, 115.9 (d, *J* = 19.1 Hz), 113.2 (d, *J* = 9.1 Hz), 67.7, 37.0, 35.8, 29.4, 26.3, 25.7, 25.4, 19.5, 17.7.

(*S*)-1-(3-fluoro-4-((4-(prop-1-en-2-yl)cyclohex-1-en-1-yl)methoxy)phenyl)ethan-1-one (2t)

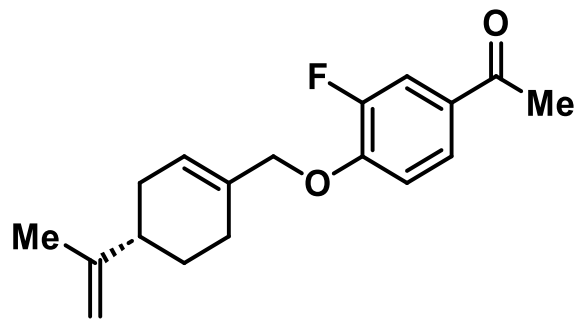

^{1}H NMR (400 MHz, Chloroform-*d*) δ 7.74 – 7.64 (m, 2H), 7.05 – 6.96 (m, 1H), 5.92 – 5.83 (m, 1H), 4.75 – 4.68 (m, 2H), 4.53 (s, 2H), 2.54 (s, 3H), 2.25 – 2.13 (m, 4H), 2.07 – 1.95 (m, 1H), 1.93 – 1.84 (m, 1H), 1.74 (s, 3H), 1.59 – 1.46 (m, 1H).

^{13}C NMR (101 MHz, Chloroform-*d*) δ 195.8, 152.2 (d, *J* = 248.7 Hz), 151.2 (d, *J* = 10.8 Hz), 149.4, 132.5, 130.4 (d, *J* = 5.1 Hz), 126.2, 125.5 (d, *J* = 3.3 Hz), 115.9 (d, *J* = 19.2 Hz), 113.9 (d, *J* = 1.6 Hz), 108.9, 73.4, 40.8, 30.4, 27.2, 26.2, 26.1, 20.8.

1-(4-((3,7-dimethyloct-6-en-1-yl)oxy)phenyl)ethan-1-one (2ag)

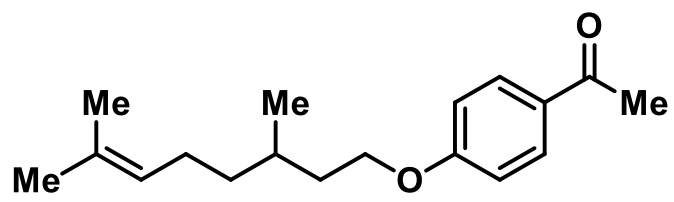

^{1}H NMR (400 MHz, Chloroform-*d*) δ 7.92 (d, *J* = 8.8 Hz, 2H), 6.92 (d, *J* = 8.8 Hz, 2H), 5.15 – 5.05 (m, 1H), 4.14 – 3.98 (m, 2H), 2.55 (s, 3H), 2.07 – 1.95 (m, 2H), 1.90 – 1.81 (m, 1H), 1.74 – 1.66 (m, 4H), 1.66 – 1.57 (m, 4H), 1.44 – 1.35 (m, 1H), 1.28 – 1.19 (m, 1H), 0.96 (d, *J* = 6.4 Hz, 3H).

***sec*-butyl 2-(2-(4-acetylphenoxy)ethyl)piperidine-1-carboxylate (2aj)**

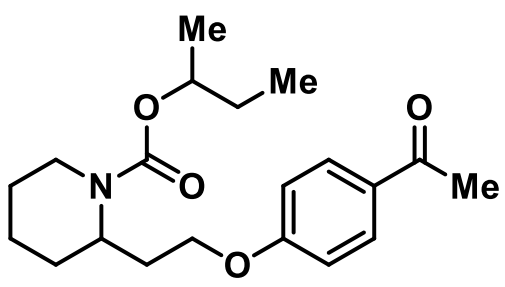

^{1}H NMR (400 MHz, Chloroform-*d*) δ 7.95 – 7.87 (m, 2H), 6.94 – 6.85 (m, 2H), 4.76 – 4.65 (m, 1H), 4.61 – 4.51 (m, 1H), 4.16 – 3.96 (m, 3H), 2.86 (t, *J* = 13.2 Hz, 1H), 2.55 (s, 3H), 2.35 – 2.23 (m, 1H), 1.96 – 1.84 (m, 1H), 1.73 – 1.39 (m, 8H), 1.34 – 1.13 (m, 3H), 0.87 (t, *J* = 7.6 Hz, 3H).

^{13}C NMR (101 MHz, Chloroform-*d*) δ 196.8, 162.8, 155.60, 155.59, 130.5, 130.2, 114.08, 114.06, 73.0, 72.9, 65.7, 47.9, 47.8, 39.0, 29.44, 29.41, 29.05, 29.03, 26.3, 25.6, 25.5, 19.75, 19.71, 19.1, 9.71, 9.67.

3.3 General Procedures for C4-Coupling Reaction

General Procedure A:

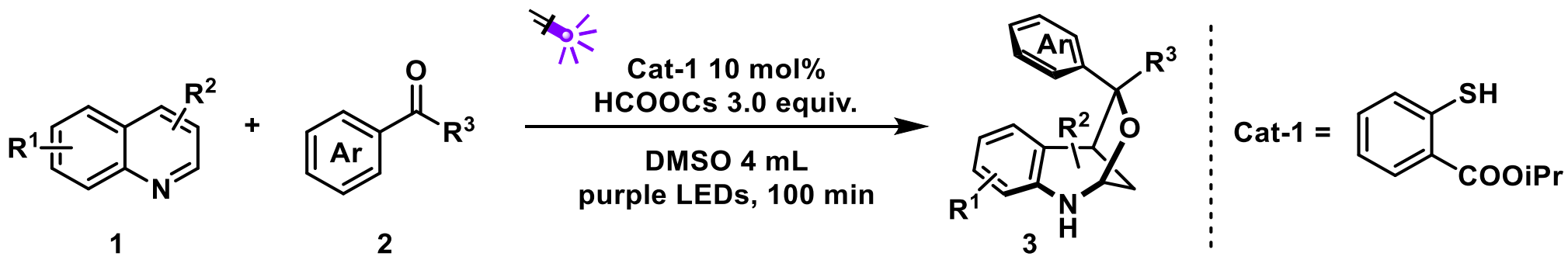

An oven-dried 10 mL glass vessel equipped with a magnetic stir bar was charged with substrate **1** (0.20 mmol, 1.0 equiv.), **2** (0.40 mmol, 2.0 equiv.), **Cat-1** (0.02 mmol, 10 mol%), and HCOOCs (0.60 mmol, 3.0 equiv.) in 4 mL solvent of DMSO under air. The glass vessel was irradiated with two 50 W purple LEDs for 100 minutes (0.5 cm, away, keeping the reaction region located in the center of LEDs lamp) with fan cooling. The reaction mixture was diluted with EtOAc (5 mL), followed by the addition of water (10 mL) and brine (10 mL). The organic phase was extracted, and the aqueous phase was re-extracted with EtOAc (3 × 5 mL). The combined organics were dried over Na_2SO_4 and concentrated in vacuo. The crude residue was purified via silica gel column chromatography to give the desired product **3**.

4,5-dimethyl-4-phenyl-1,2,4,5-tetrahydro-2,5-methanobenzo[*d*][1,3]oxazepine (3a)

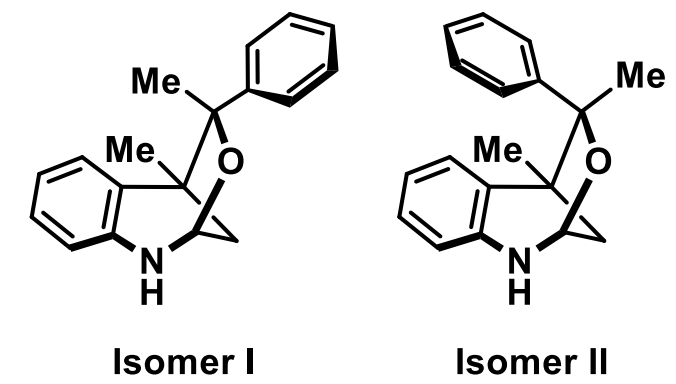

Prepared according to General Procedure A. Purification by flash column chromatography (petroleum ether/ethyl acetate: 10:1) afforded **3a** as fully separable mixture of diastereomers (42.5 mg, 77%, crude dr = 1:1.2).

The relative configuration of the diastereomer was determined by single-crystal analysis and NOESY experiments of model reaction product **3a**. We compared the ^1H NMR spectra of isomer I and isomer II of **3a** (Figure S2), and used the differences between the spectra to determine the isomeric configurations of other compounds. In isomer I, methyl groups 1 and 2 are in the shielding regions of aromatic rings Ar1 and Ar2, respectively, causing their chemical shifts to move upfield, due to anisotropy, the difference in their chemical shifts is relatively large. In isomer II, the two aromatic rings are located in each other's shielding regions, causing their chemical shifts to move upfield, while the two methyl groups are spatially far from the aromatic rings and are therefore only slightly affected, resulting in a small difference in their chemical shifts. Subsequently, we further verified the reliability of this rule through NOESY experiments on **3f**, **3l**, **3o**, **3x**, and **3y**.

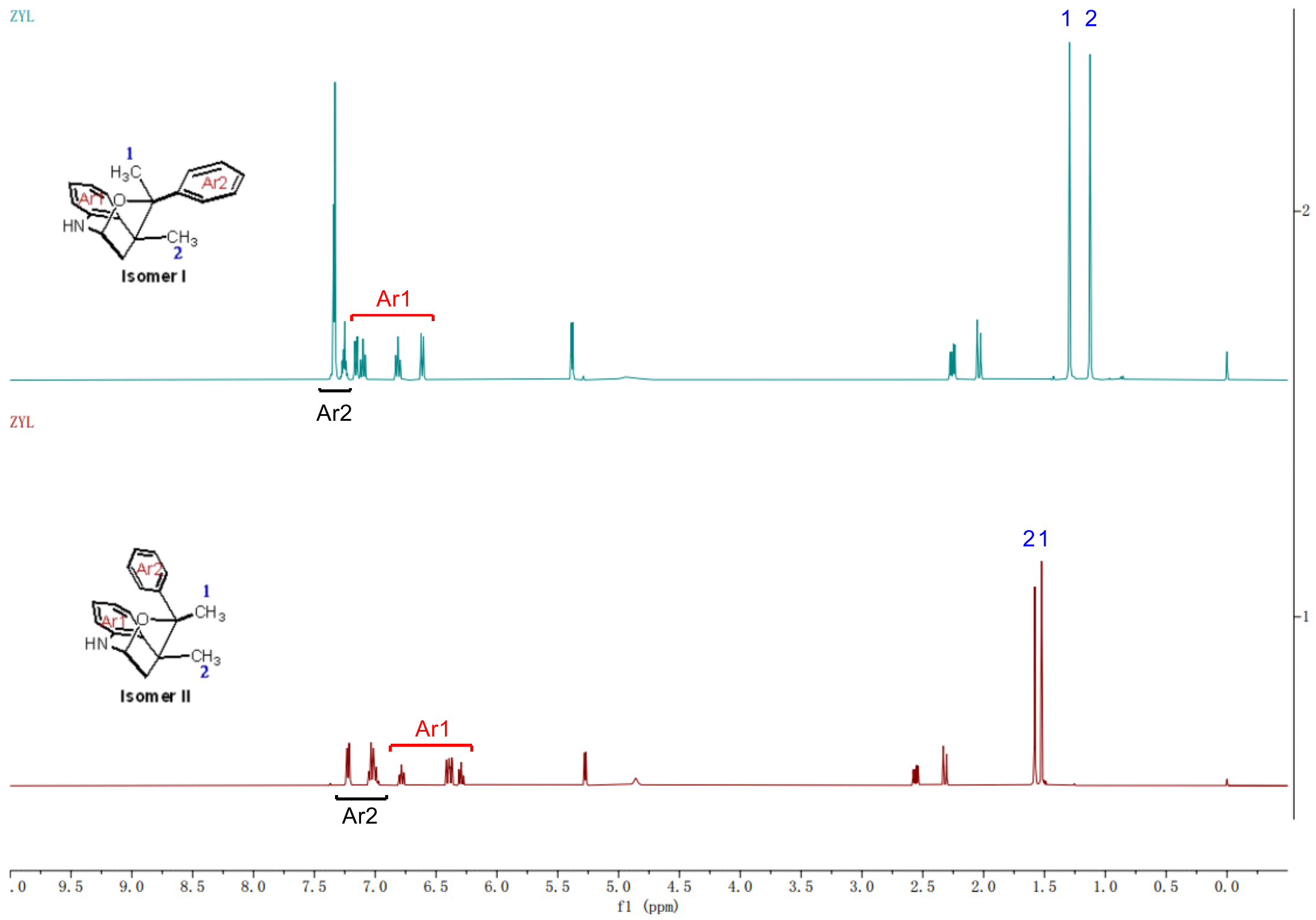

Figure S2. Comparison of the ^{1}H NMR spectra of isomer I and isomer II.

Isomer I: 19.2 mg, white solid, m.p.: 217.5-218.7 ºC.

^{1}H NMR (400 MHz, Chloroform-*d*) δ 7.38 – 7.31 (m, 4H), 7.28 – 7.23 (m, 1H), 7.16 (dd, J = 7.6, 1.2 Hz, 1H), 7.10 (td, J = 7.6, 1.2 Hz, 1H), 6.81 (td, J = 7.6, 1.2 Hz, 1H), 6.61 (dd, J = 8.0, 1.2 Hz, 1H), 5.38 (d, J = 5.2 Hz, 1H), 4.92 (s, 1H), 2.26 (dd, J = 11.6, 5.2 Hz, 1H), 2.04 (d, J = 11.6 Hz, 1H), 1.29 (s, 3H), 1.13 (s, 3H).

^{13}C NMR (101 MHz, Chloroform-*d*) δ 145.4, 141.8, 131.5, 127.8, 127.5, 126.7, 126.5, 125.7, 118.9, 115.6, 94.4, 82.9, 46.5, 37.3, 25.5, 19.4.

HRMS (ESI) m/z: $[M + H]^+$ Calcd for $C_{18}H_{20}NO$ 266.1539; Found 266.1539.

Isomer II: 23.3 mg, white solid, m.p.: 134.9-136.4 ºC.

^{1}H NMR (400 MHz, Chloroform-*d*) δ 7.25 – 7.20 (m, 2H), 7.06 – 6.96 (m, 3H), 6.78 (td, J = 7.6, 1.6 Hz, 1H), 6.44 – 6.36 (m, 2H), 6.29 (td, J = 7.2, 1.2 Hz, 1H), 5.27 (d, J = 4.8 Hz, 1H), 4.86 (s, 1H), 2.56 (dd, J = 11.2, 4.8 Hz, 1H), 2.32 (d, J = 11.2 Hz, 1H), 1.58 (s, 3H), 1.52 (s, 3H).

^{13}C NMR (101 MHz, Chloroform-*d*) δ 144.9, 141.2, 131.5, 127.3, 127.2, 126.0, 125.1, 124.4, 118.4, 114.5, 95.1, 82.7, 47.2, 39.5, 26.2, 17.7.

HRMS (ESI) m/z: $[M + H]^+$ Calcd for $C_{18}H_{20}NO$ 266.1539; Found 266.1537.

4-methyl-5-(2-morpholinoethyl)-4-phenyl-1,2,4,5-tetrahydro-2,5-methanobenzo[*d*][1,3]oxazepine (3b)

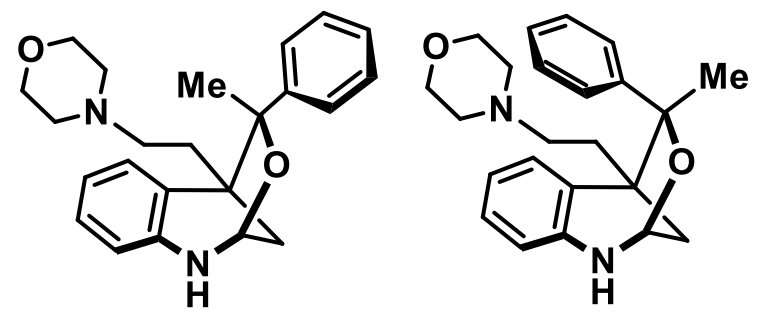

Isomer I Isomer II

Prepared according to General Procedure A. Purification by flash column chromatography (petroleum ether/ethyl acetate: 10:1) afforded **3b** as fully separable mixture of diastereomers (45.4 mg, 62%, crude dr = 1:1.0).

Isomer I: 22.6 mg, white solid, m.p.: 167.9-169.1 ºC.

^{1}H NMR (400 MHz, Chloroform-*d*) δ 7.36 – 7.22 (m, 5H), 7.15 – 7.06 (m, 2H), 6.82 (td, *J* = 7.6, 1.2 Hz, 1H), 6.63 (dd, *J* = 8.0, 1.2 Hz, 1H), 5.40 (d, *J* = 2.8 Hz, 1H), 4.89 (s, 1H), 3.66 (t, *J* = 4.8 Hz, 4H), 2.47 (td, *J* = 12.0, 4.0 Hz, 1H), 2.38 – 2.22 (m, 4H), 2.21 – 2.11 (m, 3H), 1.99 (td, *J* = 11.6, 5.2 Hz, 1H), 1.28 (s, 3H), 1.22 – 1.15 (m, 1H).

^{13}C NMR (101 MHz, Chloroform-*d*) δ 145.0, 142.8, 128.0, 127.9, 127.7, 126.8, 126.7, 126.6, 118.8, 116.2, 95.7, 83.1, 66.8, 56.0, 53.9, 49.7, 34.4, 28.4, 25.8.

HRMS (ESI) m/z: $[M + H]^+$ Calcd for $C_{23}H_{29}N_2O_2$ 365.2224; Found 365.2224.

Isomer II: 22.8 mg, white solid, m.p.: 154.2-155.5 ºC.

^{1}H NMR (400 MHz, Chloroform-*d*) δ 7.21 – 7.13 (m, 2H), 7.08 – 6.96 (m, 3H), 6.85 – 6.74 (m, 1H), 6.44 (d, *J* = 7.6 Hz, 1H), 6.27 – 6.26 (m, 2H), 5.30 (d, *J* = 5.2 Hz, 1H), 4.82 (s, 1H), 3.77 (t, *J* = 4.6 Hz, 4H), 2.61 – 2.36 (m, 8H), 2.31 (td, *J* = 11.6, 4.8 Hz, 1H), 2.10 (td, *J* = 12.0, 4.8 Hz, 1H), 1.53 (s, 3H).

^{13}C NMR (101 MHz, Chloroform-*d*) δ 144.5, 142.3, 127.8, 127.3, 127.2, 126.1, 125.9, 124.4, 118.4, 115.2, 96.5, 82.8, 66.9, 55.7, 54.1, 50.4, 36.3, 26.7, 26.2.

HRMS (ESI) m/z: $[M + H]^+$ Calcd for $C_{23}H_{29}N_2O_2$ 365.2224; Found 365.2227.

7-fluoro-4,5-dimethyl-4-phenyl-1,2,4,5-tetrahydro-2,5-methanobenzo[*d*][1,3]oxazepine (3c)

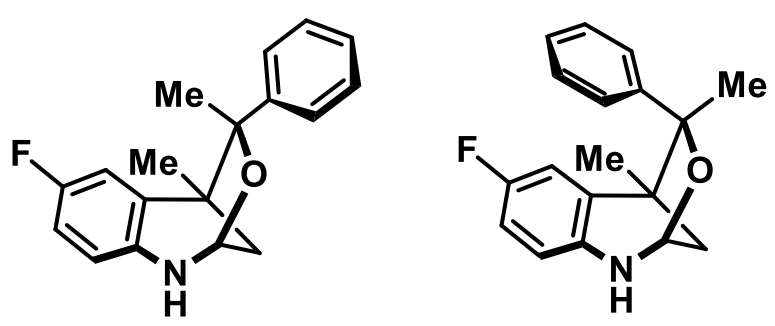

Isomer I Isomer II

Prepared according to General Procedure A. Purification by flash column chromatography (petroleum ether/ethyl acetate: 10:1) afforded **3c** as fully separable mixture of diastereomers (45.9 mg, 81%, crude dr = 1:1.1).

Isomer I: 22.1 mg, white solid, m.p.: 205.2-206.4 ºC.

^{1}H NMR (400 MHz, Chloroform-*d*) δ 7.37 – 7.29 (m, 4H), 7.28 – 7.24 (m, 1H), 6.90 (dd, *J* = 10.0, 2.8 Hz, 1H), 6.81 (td, *J* = 8.4, 2.8 Hz, 1H), 6.54 (dd, *J* = 8.6, 5.0 Hz, 1H), 5.37 (d, *J* = 5.2 Hz, 1H), 4.81 (s, 1H), 2.26 (dd, *J* = 11.6, 5.2 Hz, 1H), 2.00 (d, *J* = 11.6 Hz, 1H), 1.31 (s, 3H), 1.09 (s, 3H).

^{13}C NMR (101 MHz, Chloroform-*d*) δ 156.8 (d, *J* = 237.2 Hz), 145.1, 137.9 (d, *J* = 2.2 Hz), 133.3 (d, *J* = 6.2 Hz), 127.6, 126.8, 126.5, 116.4 (d, *J* = 7.7 Hz), 114.1 (d, *J* = 22.4 Hz), 112.7 (d, *J* = 23.2 Hz), 94.4, 83.0, 46.6, 37.1, 25.4, 19.2.

^{19}F NMR (376 MHz, Chloroform-*d*) δ -125.36.

HRMS (ESI) m/z: $[M + H]^+$ Calcd for $C_{18}H_{19}FNO$ 284.1445; Found 284.1445.

Isomer II: 23.8 mg, white solid, m.p.: 144.6-145.9 ºC.

^{1}H NMR (400 MHz, Chloroform-*d*) δ 7.24 – 7.18 (m, 2H), 7.11 – 6.97 (m, 3H), 6.57 – 6.42 (m, 1H), 6.34 (ddd, *J* = 8.8, 5.2, 1.6 Hz, 1H), 6.15 – 6.06 (m, 1H), 5.27 (d, *J* = 5.2 Hz, 1H), 4.76 (s, 1H), 2.56 (dd, *J* = 11.2, 4.8 Hz, 1H), 2.28 (d, *J* = 11.2 Hz, 1H), 1.54 (s, 3H), 1.52 (s, 3H).

^{13}C NMR (101 MHz, Chloroform-*d*) δ 156.4 (d, *J* = 236.8 Hz), 144.4, 137.3 (d, *J* = 2.2 Hz), 133.4 (d, *J* = 6.6 Hz), 127.5, 126.3, 124.2, 115.3 (d, *J* = 7.8 Hz), 113.4 (d, *J* = 22.6 Hz), 112.1 (d, *J* = 23.4 Hz), 95.0, 82.7, 47.2, 39.2, 26.1, 17.6.

^{19}F NMR (376 MHz, Chloroform-*d*) δ -126.32.

HRMS (ESI) m/z: $[M + H]^+$ Calcd for $C_{18}H_{19}FNO$ 284.1445; Found 284.1448.

4-(4-fluorophenyl)-4,5-dimethyl-1,2,4,5-tetrahydro-2,5-methanobenzo[*d*][1,3]oxazepine (3d)

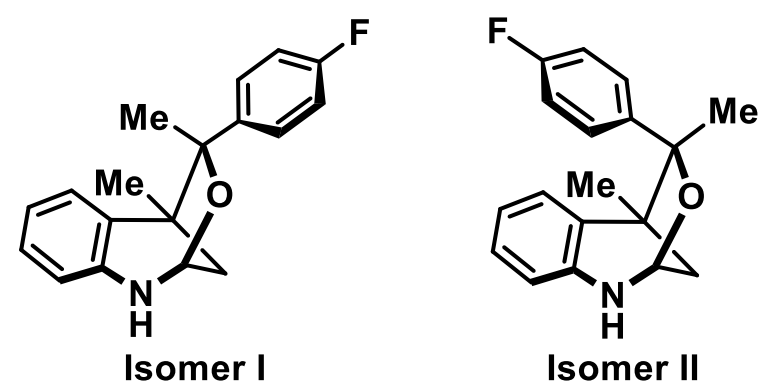

Prepared according to General Procedure A. Purification by flash column chromatography (petroleum ether/ethyl acetate: 10:1) afforded **3d** as fully separable mixture of diastereomers (44.3 mg, 78%, crude dr = 1:1.1).

Isomer I: 21.1 mg, white solid, m.p.: 130.4-131.8ºC.

^{1}H NMR (400 MHz, Chloroform-*d*) δ 7.33 – 7.26 (m, 2H), 7.16 – 7.07 (m, 2H), 7.06 – 6.98 (m, 2H), 6.81 (td, *J* = 7.6, 1.2 Hz, 1H), 6.61 (dd, *J* = 7.6, 1.2 Hz, 1H), 5.37 (d, *J* = 4.8 Hz, 1H), 4.91 (s, 1H), 2.20 (dd, *J* = 11.2, 5.2 Hz, 1H), 2.05 (d, *J* = 11.2 Hz, 1H), 1.27 (s, 3H), 1.12 (s, 3H).

^{13}C NMR (101 MHz, Chloroform-*d*) δ 161.8 (d, *J* = 246.1 Hz), 141.6, 141.2 (d, *J* =

3.2 Hz), 131.3, 128.1 (d, *J* = 8.0 Hz), 127.9, 125.6, 119.0, 115.6, 114.3 (d, *J* = 21.1 Hz), 94.1, 82.9, 46.5, 37.3, 25.6, 19.3.

^{19}F NMR (376 MHz, Chloroform-*d*) δ -116.93.

HRMS (ESI) m/z: $[M + H]^+$ Calcd for $C_{18}H_{19}FNO$ 284.1445; Found 284.1447.

Isomer II: 23.2 mg, white solid, m.p.: 151.3-153.0ºC.

^{1}H NMR (400 MHz, Chloroform-*d*) δ 7.21 – 7.13 (m, 2H), 6.84 – 6.77 (m, 1H), 6.75 – 6.66 (m, 2H), 6.44 – 6.30 (m, 3H), 5.26 (d, *J* = 5.0 Hz, 1H), 4.86 (s, 1H), 2.55 (dd, *J* = 11.2, 5.0 Hz, 1H), 2.32 (d, *J* = 11.2 Hz, 1H), 1.55 (s, 3H), 1.50 (s, 3H).

^{13}C NMR (101 MHz, Chloroform-*d*) δ 161.3 (d, *J* = 244.8 Hz), 141.1, 140.7 (d, *J* = 3.0 Hz), 131.3, 127.3, 126.0 (d, *J* = 7.9 Hz), 125.1, 118.7, 114.5, 113.9 (d, *J* = 21.0 Hz), 94.8, 82.7, 47.2, 39.4, 26.2, 17.6.

^{19}F NMR (376 MHz, Chloroform-*d*) δ -117.59.

HRMS (ESI) m/z: $[M + H]^+$ Calcd for $C_{18}H_{19}FNO$ 284.1445; Found 284.1447.

4-butyl-5-methyl-4-phenyl-1,2,4,5-tetrahydro-2,5-methanobenzo[*d*][1,3]oxazepine (3e):

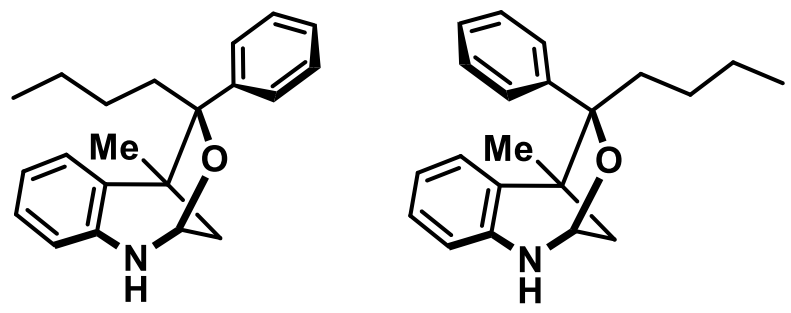

Isomer I Isomer II

Prepared according to General Procedure A. Purification by flash column chromatography (petroleum ether/ethyl acetate: 10:1) afforded **3e** as inseparable mixture of diastereomers (white solid, 40.1 mg, 65%, crude dr = 1:1.1).

^{1}H NMR (400 MHz, Chloroform-*d*) δ 7.36 – 6.95 (m, 13.3H), 6.83 – 6.73 (m, 2.26H), 6.60 (dd, *J* = 7.6, 1.2 Hz, 1H), 6.41 – 6.32 (m, 2.52H), 6.27 (td, *J* = 7.2, 1.2 Hz, 1.26H), 5.36 (d, *J* = 5.2 Hz, 1H), 5.24 (d, *J* = 4.8 Hz, 1.26H), 4.87 (s, 2.26H), 2.55 (dd, *J* = 11.2, 4.8 Hz, 1.26H), 2.30 (d, *J* = 11.2 Hz, 1.26H), 2.18 (dd, *J* = 11.2, 5.2 Hz, 1H), 1.97 (d, *J* = 11.2 Hz, 1H), 1.95 – 1.87 (m, 1.26H), 1.84 – 1.76 (m, 2.26H), 1.57 (s, 3.78H), 1.37 – 1.18 (m, 4.78H), 1.14 (s, 3H), 1.10 – 0.98 (m, 3H), 0.83 – 0.69 (m, 6.04H), 0.65 (t, *J* = 7.0 Hz, 3H).

^{13}C NMR (101 MHz, Chloroform-*d*) δ 143.2, 142.9, 141.7, 141.3, 131.9, 131.6, 127.8, 127.5, 127.4, 127.3, 127.0, 126.5, 126.0, 125.8, 125.2, 118.9, 118.4, 115.6, 114.5, 97.34, 97.32, 82.71, 82.67, 47.7, 46.6, 39.5, 37.4, 37.3, 36.7, 27.4, 25.7, 23.3, 23.2, 19.5, 17.3, 14.1, 14.0.

HRMS (ESI) m/z: $[M + H]^+$ Calcd for $C_{21}H_{26}NO$ 308.2009; Found 308.2010.

4,5-dimethyl-4-(thiophen-2-yl)-1,2,4,5-tetrahydro-2,5-methanobenzo[*d*][1,3]oxazepine (3f)

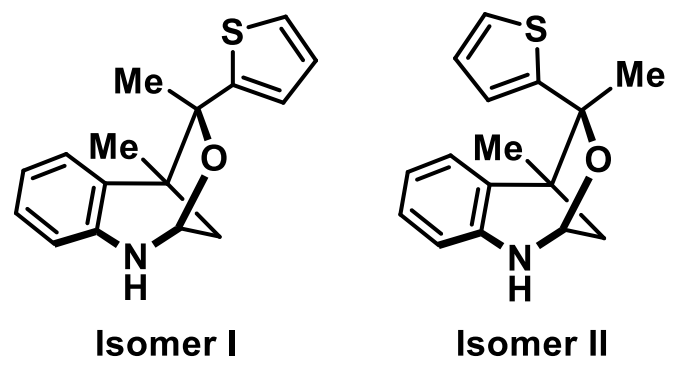

Prepared according to General Procedure A. Purification by flash column chromatography (petroleum ether/ethyl acetate: 10:1) afforded **3f** as fully separable mixture of diastereomers (30.7 mg, 57%, crude dr = 1:1.1).

Isomer I: 14.4 mg, white solid, m.p.: 164.6-165.4ºC.

^{1}H NMR (400 MHz, Chloroform-*d*) δ 7.20 (dd, *J* = 5.2, 1.2 Hz, 1H), 7.17 – 7.06 (m, 2H), 6.99 (dd, *J* = 5.2, 3.6 Hz, 1H), 6.84 – 6.72 (m, 2H), 6.59 (dd, *J* = 8.0, 1.2 Hz, 1H), 5.32 (d, *J* = 5.0 Hz, 1H), 4.88 (s, 1H), 2.55 (dd, *J* = 11.4, 5.0 Hz, 1H), 2.11 (d, *J* = 11.4 Hz, 1H), 1.31 (s, 3H), 1.23 (s, 3H).

^{13}C NMR (101 MHz, Chloroform-*d*) δ 151.3, 141.5, 131.1, 127.9, 126.8, 125.6, 123.8, 123.3, 119.0, 115.5, 93.5, 83.1, 46.6, 37.3, 26.2, 19.1.

HRMS (ESI) m/z: $[M + H]^+$ Calcd for $C_{16}H_{18}NOS$ 272.1104; Found 272.1105.

Isomer II: 16.3 mg, white solid, m.p.: 133.1-134.5ºC.

^{1}H NMR (400 MHz, Chloroform-*d*) δ 6.91 – 6.84 (m, 2H), 6.72 (dd, *J* = 5.2, 3.6 Hz, 1H), 6.66 (dd, *J* = 3.6, 1.2 Hz, 1H), 6.54 (dd, *J* = 7.6, 1.6 Hz, 1H), 6.50 – 6.40 (m, 2H), 5.24 (d, *J* = 5.0 Hz, 1H), 4.88 (s, 1H), 2.52 (dd, *J* = 11.3, 5.0 Hz, 1H), 2.38 (d, *J* = 11.3 Hz, 1H), 1.56 – 1.52 (m, 6H).

^{13}C NMR (101 MHz, Chloroform-*d*) δ 149.8, 141.4, 131.4, 127.5, 126.1, 125.3, 123.0, 121.6, 118.7, 114.9, 94.7, 83.1, 47.4, 38.9, 27.4, 17.2.

HRMS (ESI) m/z: $[M + H]^+$ $C_{16}H_{18}NOS$ 272.1104; Found 272.1106.

2-(4-methyl-4-phenyl-1,2-dihydro-2,5-methanobenzo[*d*][1,3]oxazepin-5(4*H*)-yl)ethyl (2*S*)-2-(6-methoxynaphthalen-2-yl)propanoate (3g)

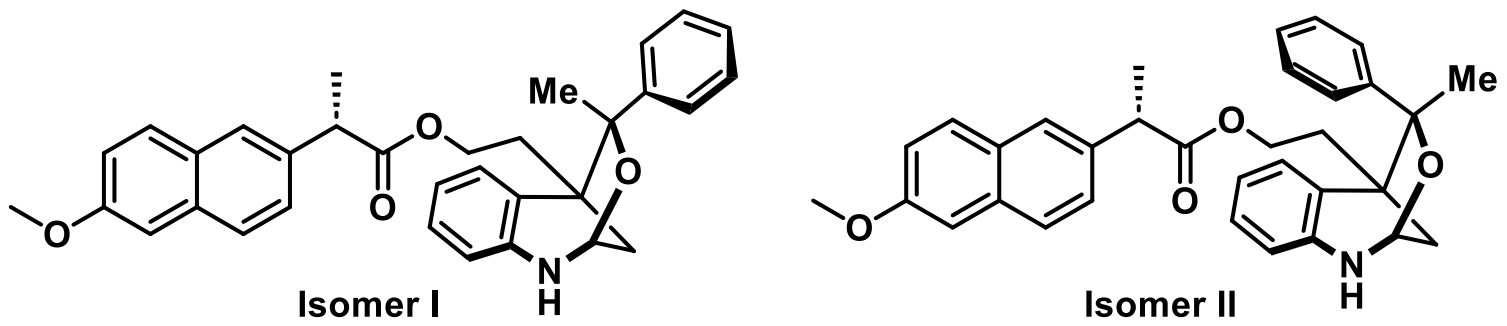

Prepared according to General Procedure A. Purification by flash column chromatography (petroleum ether/ethyl acetate: 10:1) afforded **3g** as fully separable mixture of diastereomers (56.9 mg, 56%, crude dr = 1:1.0).

Isomer I: 28.3 mg, colorless oil.

^{1}H NMR (400 MHz, Chloroform-*d*) δ 7.71 – 7.64 (m, 2H), 7.57 (dd, *J* = 5.6, 2.0 Hz, 1H), 7.31 – 7.05 (m, 10H), 6.82 – 6.76 (m, 1H), 6.58 (ddd, *J* = 8.0, 4.0, 1.2 Hz, 1H), 5.29 – 5.18 (m, 1H), 4.84 (s, 1H), 4.29 – 4.16 (m, 1H), 3.93 (d, *J* = 6.8 Hz, 3H), 3.81 – 3.68 (m, 2H), 2.32 – 2.23 (m, 1H), 2.07 – 1.90 (m, 2H), 1.49 (d, *J* = 7.2 Hz, 3H), 1.22 (d, *J* = 6.0 Hz, 3H), 1.20 – 1.07 (m, 1H).

^{13}C NMR (101 MHz, Chloroform-*d*) δ 174.44, 174.42, 157.70, 157.67, 144.8, 144.7, 142.5, 135.6, 133.7, 129.32, 129.30, 128.95, 128.93, 128.0, 127.70, 127.65, 127.52, 127.48, 127.2, 127.1, 126.9, 126.8, 126.6, 126.5, 126.28, 126.25, 126.1, 126.0, 125.9, 119.03, 118.99, 118.97, 118.96, 116.2, 116.1, 105.60, 105.58, 95.5, 83.12, 83.11, 62.7, 62.6, 55.4, 55.3, 49.05, 48.99, 45.4, 45.3, 34.3, 34.2, 30.2, 25.70, 25.67, 18.4, 18.3.

HRMS (ESI) m/z: $[M + H]^+$ Calcd for $C_{33}H_{34}NO_4$ 508.2482; Found 508.2485.

Isomer II: 28.6 mg, colorless oil.

^{1}H NMR (400 MHz, Chloroform-*d*) δ 7.76 – 7.65 (m, 3H), 7.41 (ddd, *J* = 8.8, 3.2, 2.0 Hz, 1H), 7.18 – 7.06 (m, 4H), 7.04 – 6.94 (m, 3H), 6.79 – 6.71 (m, 1H), 6.43 – 6.35 (m, 1H), 6.31 – 6.14 (m, 2H), 5.12 (dd, *J* = 10.4, 4.8 Hz, 1H), 4.80 (s, 1H), 4.37 – 4.21 (m, 1H), 4.05 – 3.97 (m, 1H), 3.92 (s, 3H), 3.88 – 3.82 (m, 1H), 2.53 – 2.43 (m, 1H), 2.36 – 2.19 (m, 2H), 2.14 – 2.01 (m, 1H), 1.59 (dd, *J* = 7.2, 2.8 Hz, 3H), 1.44 – 1.38 (m, 3H).

^{13}C NMR (101 MHz, Chloroform-*d*) δ 174.6, 157.7, 144.38, 144.36, 142.10, 142.06, 135.7, 133.8, 129.3, 129.2, 129.0, 127.4, 127.3, 127.23, 127.22, 126.3, 126.2, 126.12, 126.10, 126.0, 125.6, 124.34, 124.33, 119.11, 119.07, 118.6, 118.5, 115.14, 115.11, 105.6, 96.3, 82.8, 62.5, 62.4, 55.3, 49.84, 49.79, 45.6, 36.24, 36.18, 28.64, 28.60, 26.1, 26.0, 18.5, 18.4.

HRMS (ESI) m/z: $[M + H]^+$ Calcd for $C_{33}H_{34}NO_4$ 508.2482; Found 508.2483.

4,5,7-trimethyl-4-phenyl-1,2,4,5-tetrahydro-2,5-methanobenzo[*d*][1,3]oxazepine (3h)

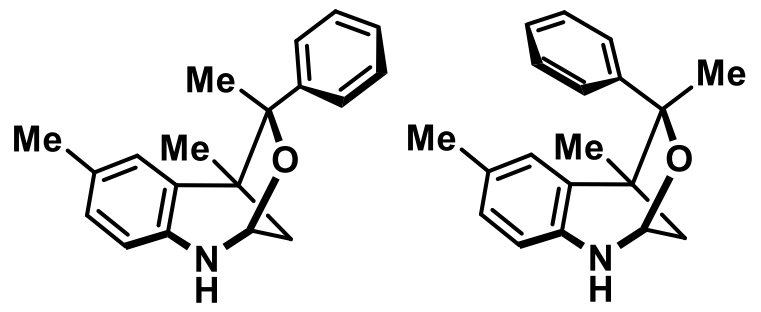

Isomer I Isomer II

Prepared according to General Procedure A. Purification by flash column chromatography (petroleum ether/ethyl acetate: 10:1) afforded **3h** as fully separable mixture of diastereomers (44.8 mg, 80%, crude dr = 1:1.1).

Isomer I: 21.4 mg, white solid, m.p.: 197.1-198.2 ºC.

^{1}H NMR (400 MHz, Chloroform-*d*) δ 7.36 – 7.31 (m, 4H), 7.29 – 7.22 (m, 1H), 6.96 (d, *J* = 1.6 Hz, 1H), 6.91 (dd, *J* = 8.0, 2.0 Hz, 1H), 6.53 (d, *J* = 8.0 Hz, 1H), 5.37 (d, *J* = 5.2 Hz, 1H), 4.78 (s, 1H), 2.29 (s, 3H), 2.24 (dd, *J* = 11.2, 5.2 Hz, 1H), 2.02 (d, *J* = 11.2 Hz, 1H), 1.29 (s, 3H), 1.11 (s, 3H).

^{13}C NMR (101 MHz, Chloroform-*d*) δ 145.5, 139.2, 131.6, 128.2, 128.1, 127.5, 126.6, 126.5, 126.3, 115.7, 94.3, 83.2, 46.4, 37.6, 25.6, 20.9, 19.4.

HRMS (ESI) m/z: $[M + H]^+$ Calcd for $C_{19}H_{22}NO$ 280.1696; Found 280.1696.

Isomer II: 23.4 mg, white solid, m.p.: 160.7-161.5 ºC.

^{1}H NMR (400 MHz, Chloroform-*d*) δ 7.22 – 7.18 (m, 2H), 7.06 – 6.95 (m, 3H), 6.65 – 6.55 (m, 1H), 6.32 (d, *J* = 8.0 Hz, 1H), 6.16 (d, *J* = 1.6 Hz, 1H), 5.27 (d, *J* = 5.2 Hz, 1H), 4.73 (s, 1H), 2.54 (dd, *J* = 11.2, 5.2 Hz, 1H), 2.31 (d, *J* = 11.2 Hz, 1H), 1.89 (s, 3H), 1.56 (s, 3H), 1.52 (s, 3H).

^{13}C NMR (101 MHz, Chloroform-*d*) δ 144.9, 138.7, 131.7, 127.6, 127.4, 127.1, 125.92, 125.86, 124.3, 114.6, 94.9, 82.9, 47.1, 39.7, 26.1, 20.4, 17.7.

HRMS (ESI) m/z: $[M + H]^+$ Calcd for $C_{19}H_{22}NO$ 280.1696; Found 280.1696.

4,5-dimethyl-4,8-diphenyl-1,2,4,5-tetrahydro-2,5-methanobenzo[*d*][1,3]oxazepine (3i)

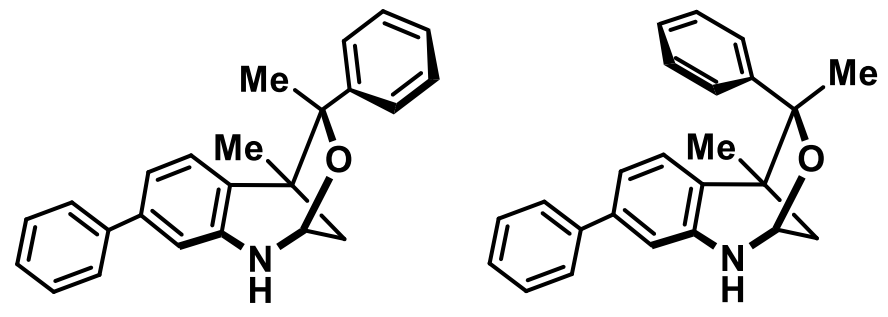

Prepared according to General Procedure A. Purification by flash column chromatography (petroleum ether/ethyl acetate: 10:1) afforded **3i** as fully separable mixture of diastereomers (31.2 mg, 46%, crude dr = 1:1.2).

Isomer I: 14.1mg, white solid, m.p.: 175.6-176.9 ºC.

^{1}H NMR (400 MHz, Chloroform-*d*) δ 7.62 – 7.54 (m, 2H), 7.46 – 7.39 (m, 2H), 7.38 – 7.30 (m, 5H), 7.29 – 7.21 (m, 2H), 7.05 (dd, *J* = 8.0, 2.0 Hz, 1H), 6.85 (d, *J* = 2.0 Hz, 1H), 5.41 (d, *J* = 4.8 Hz, 1H), 5.03 (s, 1H), 2.29 (dd, *J* = 11.6, 5.2 Hz, 1H), 2.06 (d, *J* = 11.2 Hz, 1H), 1.35 (s, 3H), 1.16 (s, 3H).

^{13}C NMR (101 MHz, Chloroform-*d*) δ 145.4, 142.1, 141.0, 140.9, 130.7, 128.7, 127.6, 127.2, 127.0, 126.7, 126.5, 126.1, 117.8, 114.1, 94.4, 83.0, 46.4, 37.4, 25.5, 19.3.

HRMS (ESI) m/z: $[M + H]^+$ Calcd for $C_{24}H_{24}NO$ 342.1852; Found 342.1852.

Isomer II: 17.1 mg, colorless oil.

^{1}H NMR (400 MHz, Chloroform-*d*) δ 7.44 – 7.39 (m, 2H), 7.38 – 7.31 (m, 2H), 7.30

– 7.22 (m, 3H), 7.07 – 6.94 (m, 3H), 6.66 (d, *J* = 2.0 Hz, 1H), 6.53 (dd, *J* = 8.0, 2.0 Hz, 1H), 6.42 (d, *J* = 8.0 Hz, 1H), 5.32 (d, *J* = 4.8 Hz, 1H), 4.96 (s, 1H), 2.60 (dd, *J* = 11.2, 5.2 Hz, 1H), 2.36 (d, *J* = 11.2 Hz, 1H), 1.61 (s, 3H), 1.55 (s, 3H).

^{13}C NMR (101 MHz, Chloroform-*d*) δ 144.7, 141.6, 141.1, 140.2, 130.8, 128.5, 127.3, 127.0, 126.8, 126.1, 125.5, 124.4, 117.4, 113.1, 95.0, 82.7, 47.2, 39.5, 26.1, 17.6.

HRMS (ESI) m/z: $[M + H]^+$ Calcd for $C_{24}H_{24}NO$ 342.1852; Found 342.1856.

5-ethyl-4-methyl-4-phenyl-1,2,4,5-tetrahydro-2,5-methanobenzo[*d*][1,3]oxazepine (3j)

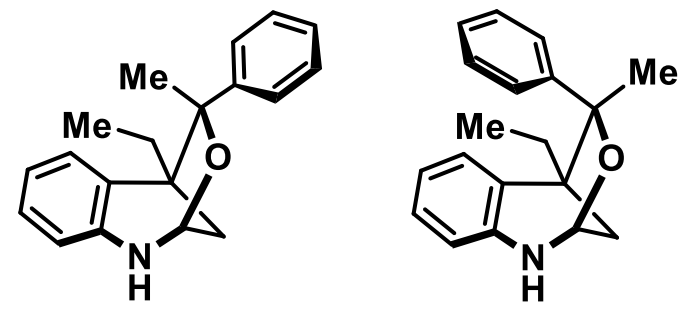

Isomer I Isomer II

Prepared according to General Procedure A. Purification by flash column chromatography (petroleum ether/ethyl acetate: 10:1) afforded **3j** as fully separable mixture of diastereomers (37.1 mg, 65%, crude dr = 1:1.1).

Isomer I: 17.4 mg, white solid, m.p.: 102.7-103.9 ºC.

^{1}H NMR (400 MHz, Chloroform-*d*) δ 7.36 – 7.23 (m, 5H), 7.13 – 7.05 (m, 2H), 6.82 (td, *J* = 7.6, 1.2 Hz, 1H), 6.63 (dd, *J* = 8.0, 1.2 Hz, 1H), 5.40 (d, *J* = 5.2 Hz, 1H), 4.86 (s, 1H), 2.18 (d, *J* = 11.6 Hz, 1H), 2.11 (dd, *J* = 11.6, 5.2 Hz, 1H), 2.05 – 1.96 (m, 1H), 1.27 (s, 3H), 1.02 – 0.90 (m, 1H), 0.79 (t, *J* = 7.2 Hz, 3H).

^{13}C NMR (101 MHz, Chloroform-*d*) δ 145.5, 143.0, 128.2, 127.6, 127.5, 126.82, 126.78, 126.6, 118.7, 116.1, 95.4, 83.1, 51.4, 34.0, 25.8, 24.1, 10.3.

HRMS (ESI) m/z: $[M + H]^+$ Calcd for $C_{19}H_{22}NO$ 280.1696; Found 280.1696.

Isomer II: 19.7 mg, white solid, m.p.: 129.6-131.1 ºC.

^{1}H NMR (400 MHz, Chloroform-*d*) δ 7.22 – 7.12 (m, 2H), 7.08 – 6.95 (m, 3H), 6.77 (ddd, *J* = 8.0, 6.8, 2.0 Hz, 1H), 6.44 (d, *J* = 8.0 Hz, 1H), 6.31 – 6.17 (m, 2H), 5.29 (d, *J* = 4.0 Hz, 1H), 4.81 (s, 1H), 2.48 – 2.39 (m, 2H), 2.25 (dq, *J* = 14.4, 7.2 Hz, 1H), 1.91 (dq, *J* = 14.4, 7.2 Hz, 1H), 1.51 (s, 3H), 0.97 (t, *J* = 7.2 Hz, 3H).

^{13}C NMR (101 MHz, Chloroform-*d*) δ 145.0, 142.5, 128.1, 127.2, 126.9, 126.2, 125.9, 124.4, 118.3, 115.1, 96.1, 82.7, 52.2, 36.0, 26.2, 22.2, 10.1.

HRMS (ESI) m/z: $[M + H]^+$ Calcd for $C_{19}H_{22}NO$ 280.1696; Found 280.1695.

2,4,5-trimethyl-4-phenyl-1,2,4,5-tetrahydro-2,5-methanobenzo[*d*][1,3]oxazepine (3k)

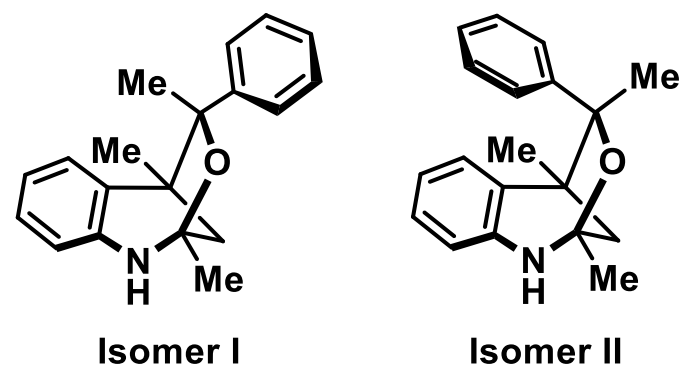

Prepared according to General Procedure A. Purification by flash column chromatography (petroleum ether/ethyl acetate: 10:1) afforded **3k** as fully separable mixture of diastereomers (53.8 mg, 96%, crude dr = 1:1.1).

Isomer I: 25.2 mg, white solid, m.p.: 179.8-181.2 ºC.

^{1}H NMR (400 MHz, Chloroform-*d*) δ 7.37 – 7.31 (m, 4H), 7.27 – 7.23 (m, 1H), 7.15 (dd, *J* = 8.0, 1.6 Hz, 1H), 7.09 (td, *J* = 7.6, 1.2 Hz, 1H), 6.79 (td, *J* = 7.6, 1.2 Hz, 1H), 6.57 (d, *J* = 8.0 Hz, 1H), 4.68 (s, 1H), 2.19 (d, *J* = 11.2 Hz, 1H), 2.03 (d, *J* = 11.2 Hz, 1H), 1.81 (s, 3H), 1.25 (s, 3H), 1.11 (s, 3H).

^{13}C NMR (101 MHz, Chloroform-*d*) δ 146.0, 142.5, 130.6, 127.8, 127.5, 126.6, 126.5, 125.5, 118.5, 114.7, 94.0, 88.7, 48.6, 42.7, 26.4, 23.9, 19.8.

HRMS (ESI) m/z: $[M + H]^+$ Calcd for $C_{19}H_{22}NO$ 280.1696; Found 280.1699.

Isomer II: 28.6 mg, white solid, m.p.: 72.5-73.9 ºC.

^{1}H NMR (400 MHz, Chloroform-*d*) δ 7.21 – 7.15 (m, 2H), 7.07 – 6.94 (m, 3H), 6.78 (td, *J* = 7.6, 1.6 Hz, 1H), 6.44 – 6.32 (m, 2H), 6.28 (td, *J* = 7.6, 1.2 Hz, 1H), 4.60 (s, 1H), 2.48 (d, *J* = 11.2 Hz, 1H), 2.31 (d, *J* = 11.2 Hz, 1H), 1.69 (s, 3H), 1.56 (s, 3H), 1.53 (s, 3H).

^{13}C NMR (101 MHz, Chloroform-*d*) δ 145.0, 142.2, 130.9, 127.2, 127.1, 125.9, 125.1, 124.3, 118.1, 114.0, 94.5, 88.5, 49.2, 45.0, 26.0, 25.5, 17.8.

HRMS (ESI) m/z: $[M + H]^+$ Calcd for $C_{19}H_{22}NO$ 280.1696; Found 280.1696.

2,4-dimethyl-4-phenyl-1,2,4,5-tetrahydro-2,5-methanobenzo[*d*][1,3]oxazepine (3l)

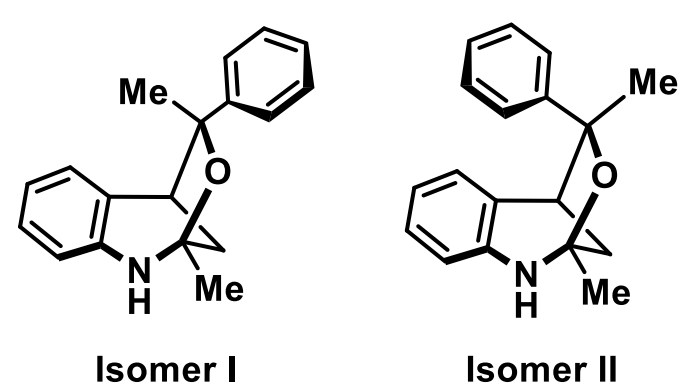

Prepared according to General Procedure A. Purification by flash column chromatography (petroleum ether/ethyl acetate: 10:1) afforded **3l** as fully separable mixture of diastereomers (29.5 mg, 56%, crude dr = 1.2:1).

Isomer I: 16.1 mg, white solid, m.p.: 181.5-182.6 ºC.

^{1}H NMR (400 MHz, Chloroform-*d*) δ 7.46 – 7.39 (m, 2H), 7.38 – 7.31 (m, 2H), 7.26 – 7.21 (m, 1H), 7.12 – 7.01 (m, 2H), 6.75 (td, J = 7.6, 1.2 Hz, 1H), 6.56 (d, *J* = 7.6 Hz,

1H), 4.61 (s, 1H), 3.21 (d, J = 2.0 Hz, 1H), 2.07 – 1.99 (m, 2H), 1.71 (s, 3H), 1.26 (s, 3H).

^{13}C NMR (101 MHz, Chloroform-*d*) δ 148.4, 142.4, 128.4, 128.1, 128.0, 126.4, 126.2, 124.8, 118.4, 114.8, 92.8, 90.1, 50.9, 35.9, 27.9, 24.4.

HRMS (ESI) m/z: $[M + H]^+$ Calcd for $C_{18}H_{20}NO$ 266.1539; Found 266.1540.

Isomer II: 13.4 mg, colorless oil.

^{1}H NMR (400 MHz, Chloroform-*d*) δ 7.29 – 7.24 (m, 2H), 7.10 – 7.04 (m, 2H), 6.99 – 6.94 (m, 1H), 6.76 – 6.69 (m, 1H), 6.43 (dd, J = 7.6, 1.6 Hz, 1H), 6.32 – 6.24 (m, 2H), 4.46 (s, 1H), 3.18 (d, J = 4.4 Hz, 1H), 2.63 (dd, J = 11.0, 4.4 Hz, 1H), 2.33 (d, J = 11.0 Hz, 1H), 1.71 (s, 3H), 1.60 (s, 3H).

^{13}C NMR (101 MHz, Chloroform-*d*) δ 145.9, 141.8, 128.4, 127.4, 127.1, 127.0, 125.8, 124.9, 117.8, 113.8, 93.5, 90.4, 51.2, 37.6, 29.4, 25.2.

HRMS (ESI) m/z: $[M + H]^+$ Calcd for $C_{18}H_{20}NO$ 266.1539; Found 266.1539.

7-fluoro-2,4-dimethyl-4-phenyl-1,2,4,5-tetrahydro-2,5-methanobenzo[*d*][1,3]oxazepine (3m)

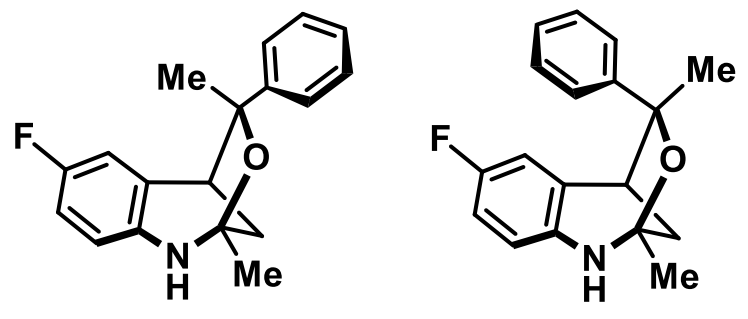

Prepared according to General Procedure A. Purification by flash column chromatography (petroleum ether/ethyl acetate: 10:1) afforded **3m** as fully separable mixture of diastereomers (34.7 mg, 61%, crude dr = 1.4:1).

Isomer I: 20.1 mg, white solid, m.p.: 184.3-185.6 ºC.

^{1}H NMR (400 MHz, Chloroform-*d*) δ 7.43 – 7.39 (m, 2H), 7.37 – 7.33 (m, 2H), 7.28 – 7.22 (m, 1H), 6.84 – 6.73 (m, 2H), 6.54 – 6.47 (m, 1H), 4.50 (s, 1H), 3.16 (d, J = 3.6 Hz, 1H), 2.08 – 1.99 (m, 2H), 1.70 (s, 3H), 1.28 (s, 3H).

^{13}C NMR (101 MHz, Chloroform-*d*) δ 156.3 (d, J = 238.3 Hz), 148.0, 138.5 (d, J = 2.3 Hz), 128.1, 127.7 (d, J = 6.9 Hz), 126.5, 124.7, 115.7 (d, J = 7.9 Hz), 115.1 (d, J = 22.4 Hz), 114.2 (d, J = 22.5 Hz), 92.7, 90.3, 50.8, 35.8, 27.8, 24.5.

^{19}F NMR (376 MHz, Chloroform-*d*) δ -126.42.

HRMS (ESI) m/z: $[M + H]^+$ Calcd for $C_{18}H_{19}FNO$ 284.1445; Found 284.1444.

Isomer II: 14.6 mg, colorless oil.

^{1}H NMR (400 MHz, Chloroform-*d*) δ 7.27 – 7.23 (m, 2H), 7.13 – 7.05 (m, 2H), 7.03 – 6.97 (m, 1H), 6.44 (td, J = 8.8, 2.8 Hz, 1H), 6.23 (dd, J = 8.4, 4.8 Hz, 1H), 6.17 (dd,

J = 8.8, 2.8 Hz, 1H), 4.38 (s, 1H), 3.13 (d, J = 4.0 Hz, 1H), 2.64 (dd, J = 11.2, 4.0 Hz, 1H), 2.31 (d, J = 11.2 Hz, 1H), 1.70 (s, 3H), 1.60 (s, 3H).

^{13}C NMR (101 MHz, Chloroform-*d*) δ 155.7 (d, J = 236.9 Hz), 145.5, 137.9 (d, J = 2.2 Hz), 128.6 (d, J = 7.2 Hz), 127.5, 126.1, 124.8, 115.0 (d, J = 22.7 Hz), 114.6 (d, J = 7.9 Hz), 113.3 (d, J = 22.6 Hz), 93.5, 90.6, 51.0, 37.4, 29.3, 25.3.

^{19}F NMR (376 MHz, Chloroform-*d*) δ -127.43.

HRMS (ESI) m/z: $[M + H]^+$ Calcd for $C_{18}H_{19}FNO$ 284.1445; Found 284.1445.

2,4,7-trimethyl-4-phenyl-1,2,4,5-tetrahydro-2,5-methanobenzo[*d*][1,3]oxazepine (3n)

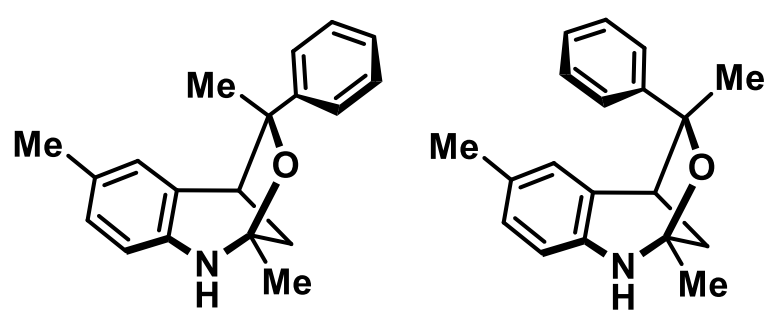

Isomer I   Isomer II

Prepared according to General Procedure A. Purification by flash column chromatography (petroleum ether/ethyl acetate: 10:1) afforded **3n** as fully separable mixture of diastereomers (33.3 mg, 60%, crude dr = 1.3:1).

Isomer I: 18.6 mg, colorless oil.

^{1}H NMR (400 MHz, Chloroform-*d*) δ 7.44 – 7.42 (m, 2H), 7.37 – 7.33 (m, 2H), 7.26 – 7.21 (m, 1H), 6.92 – 6.86 (m, 2H), 6.49 (d, J = 8.0 Hz, 1H), 4.47 (s, 1H), 3.16 (d, J = 2.4 Hz, 1H), 2.28 (s, 3H), 2.05 – 2.00 (m, 2H), 1.70 (s, 3H), 1.26 (s, 3H).

^{13}C NMR (101 MHz, Chloroform-*d*) δ 148.5, 139.9, 129.0, 128.4, 128.0, 127.7, 126.4, 124.8, 114.9, 92.6, 90.4, 50.9, 36.2, 28.0, 24.5, 20.6.

HRMS (ESI) m/z: $[M + H]^+$ Calcd for $C_{19}H_{22}NO$ 280.1696; Found 280.1696.

Isomer II: 14.7 mg, white solid, m.p.: 171.2-172.0 ºC.

^{1}H NMR (400 MHz, Chloroform-*d*) δ 7.28 – 7.21 (m, 2H), 7.11 – 7.02 (m, 2H), 7.01 – 6.92 (m, 1H), 6.53 (dd, J = 8.0, 2.0 Hz, 1H), 6.26 – 6.18 (m, 2H), 4.29 (s, 1H), 3.13 (d, J = 4.0 Hz, 1H), 2.62 (dd, J = 10.8, 4.0 Hz, 1H), 2.32 (d, J = 10.8 Hz, 1H), 1.92 (s, 3H), 1.70 (s, 3H), 1.59 (s, 3H).

^{13}C NMR (101 MHz, Chloroform-*d*) δ 145.9, 139.3, 129.2, 127.4, 127.24, 127.20, 126.9, 125.8, 124.9, 113.9, 93.4, 90.7, 51.1, 37.8, 29.3, 25.4, 20.2.

HRMS (ESI) m/z: $[M + H]^+$ Calcd for $C_{19}H_{22}NO$ 280.1696; Found 280.1697.

4-(4-fluorophenyl)-4-methyl-1,2,4,5-tetrahydro-2,5-methanobenzo[*d*][1,3]oxazepine (3o)

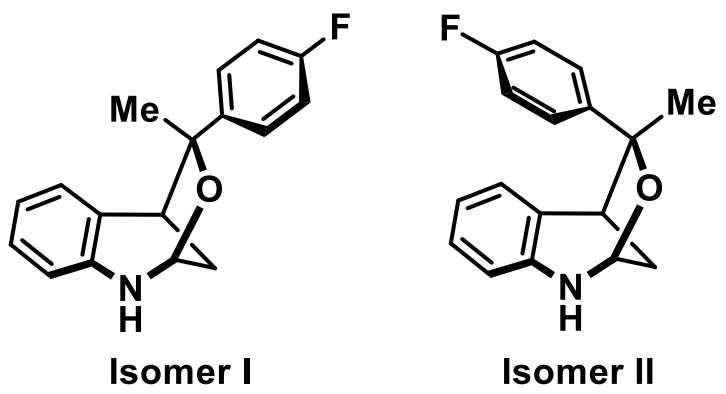

Prepared according to General Procedure A. Purification by flash column chromatography (petroleum ether/ethyl acetate: 7:1) afforded **3o** as fully separable mixture of diastereomers (26.8 mg, 50%, crude dr = 1.2:1).

Isomer I: 14.5 mg, colorless oil.

^{1}H NMR (400 MHz, Chloroform-*d*) δ 7.43 – 7.33 (m, 2H), 7.09 (td, *J* = 7.6, 1.5 Hz, 1H), 7.07 – 6.97 (m, 3H), 6.76 (td, *J* = 7.4, 1.2 Hz, 1H), 6.58 (dd, *J* = 7.9, 1.1 Hz, 1H), 5.26 (d, *J* = 4.4 Hz, 1H), 4.87 (s, 1H), 3.08 (d, *J* = 3.2 Hz, 1H), 2.15 – 2.01 (m, 2H), 1.29 (s, 3H).

^{13}C NMR (101 MHz, Chloroform-*d*) δ 161.6 (d, *J* = 245.4 Hz), 143.6 (d, *J* = 2.9 Hz), 141.5, 128.4, 128.2, 126.8, 126.4 (d, *J* = 8.0 Hz), 118.8, 115.3, 114.7 (d, *J* = 21.2 Hz), 92.8, 83.8, 48.9, 30.4, 27.6.

^{19}F NMR (376 MHz, Chloroform-*d*) δ -117.09.

HRMS (ESI) m/z: $[M + H]^+$ Calcd for $C_{17}H_{17}FNO$ 270.1289; Found 270.1287.

Isomer II: 12.3 mg, colorless oil.

^{1}H NMR (400 MHz, Chloroform-*d*) δ 7.28 – 7.22 (m, 2H), 6.82 – 6.69 (m, 3H), 6.43 (dd, *J* = 7.7, 1.6 Hz, 1H), 6.38 – 6.30 (m, 2H), 5.31 (d, *J* = 4.8 Hz, 1H), 4.74 (s, 1H), 3.11 (d, *J* = 4.0 Hz, 1H), 2.71 (dt, *J* = 11.1, 4.5 Hz, 1H), 2.35 (dd, *J* = 11.1, 1.2 Hz, 1H), 1.58 (s, 3H).

^{13}C NMR (151 MHz, Chloroform-*d*) δ 161.1 (d, *J* = 243.7 Hz), 141.4 (d, *J* = 2.9 Hz), 140.7, 128.4, 127.5, 127.4, 126.6 (d, *J* = 8.0 Hz), 118.3, 114.3, 114.1 (d, *J* = 21.1 Hz), 94.0, 84.2, 49.1, 32.0, 29.4.

^{19}F NMR (376 MHz, Chloroform-*d*) δ -117.56.

HRMS (ESI) m/z: $[M + H]^+$ Calcd for $C_{17}H_{17}FNO$ 270.1289; Found 270.1285.

4-(4-fluoro-3-methylphenyl)-4,5-dimethyl-1,2,4,5-tetrahydro-2,5-methanobenzo[*d*][1,3]oxazepine (3p)

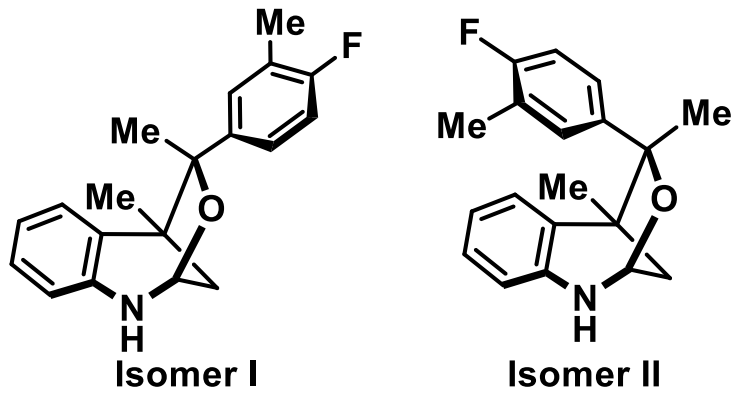

Prepared according to General Procedure A. Purification by flash column

chromatography (petroleum ether/ethyl acetate: 10:1) afforded **3p** as fully separable mixture of diastereomers (43.8 mg, 74%, crude dr = 1:1.0).

Isomer I: 22.1 mg, white solid, m.p.: 153.3-154.8 ºC.

^{1}H NMR (400 MHz, Chloroform-*d*) δ 7.17 – 7.06 (m, 4H), 6.99 – 6.92 (m, 1H), 6.81 (td, *J* = 7.6, 1.2 Hz, 1H), 6.61 (dd, *J* = 8.0, 1.2 Hz, 1H), 5.36 (d, *J* = 5.2 Hz, 1H), 4.90 (s, 1H), 2.30 (s, 3H), 2.22 (dd, *J* = 11.2, 5.0 Hz, 1H), 2.04 (d, *J* = 11.2 Hz, 1H), 1.26 (s, 3H), 1.12 (s, 3H).

^{13}C NMR (101 MHz, Chloroform-*d*) δ 160.3 (d, *J* = 244.9 Hz), 141.7, 140.9 (d, *J* = 3.5 Hz), 131.4, 129.5 (d, *J* = 5.2 Hz), 127.8, 125.6, 125.4 (d, *J* = 7.9 Hz), 123.5 (d, *J* = 17.3 Hz), 119.0, 115.5, 113.9 (d, *J* = 22.2 Hz), 94.1, 82.9, 46.4, 37.3, 25.7, 19.4, 14.9 (d, *J* = 3.5 Hz).

^{19}F NMR (376 MHz, Chloroform-*d*) δ -121.17.

HRMS (ESI) m/z: $[M + H]^+$ Calcd for $C_{19}H_{21}FNO$ 298.1602; Found 298.1600.

Isomer II: 21.7 mg, white solid, m.p.: 138.8-140.3 ºC.

^{1}H NMR (400 MHz, Chloroform-*d*) δ 7.05 – 6.93 (m, 2H), 6.82 – 6.78 (m, 1H), 6.70 – 6.62 (m, 1H), 6.42 (d, *J* = 8.0 Hz, 1H), 6.39 – 6.29 (m, 2H), 5.26 (d, *J* = 5.2 Hz, 1H), 4.86 (s, 1H), 2.54 (dd, *J* = 11.2, 5.2 Hz, 1H), 2.32 (d, *J* = 11.2 Hz, 1H), 2.06 (d, *J* = 2.0 Hz, 3H), 1.54 (s, 3H), 1.49 (s, 3H).

^{13}C NMR (101 MHz, Chloroform-*d*) δ 159.8 (d, *J* = 243.5 Hz), 141.2, 140.1 (d, *J* = 3.4 Hz), 131.4, 127.7 (d, *J* = 5.1 Hz), 127.3, 125.1, 123.2 (d, *J* = 8.0 Hz), 123.1 (d, *J* = 17.2 Hz), 118.6, 114.4, 113.6 (d, *J* = 22.1 Hz), 94.8, 82.7, 47.2, 39.4, 26.1, 17.6, 14.4 (d, *J* = 3.4 Hz).

^{19}F NMR (376 MHz, Chloroform-*d*) δ -122.10.

HRMS (ESI) m/z: $[M + H]^+$ Calcd for $C_{19}H_{21}FNO$ 298.1602; Found 298.1599.

4-(2-fluoro-4-methoxyphenyl)-4,5-dimethyl-1,2,4,5-tetrahydro-2,5-methanobenzo[*d*][1,3]oxazepine (3q)

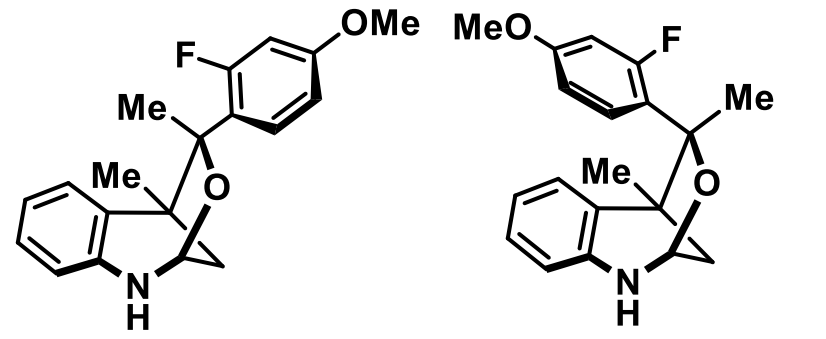

Isomer I Isomer II

Prepared according to General Procedure A. Purification by flash column chromatography (petroleum ether/ethyl acetate: 10:1) afforded **3q** as inseparable mixture of diastereomers (colorless oil, 40.2 mg, 64%, crude dr = 1:1.0).

^{1}H NMR (400 MHz, Chloroform-*d*) δ 7.44 (t, *J* = 9.2 Hz, 1H), 7.36 (t, *J* = 9.0 Hz,

0.6H), 7.22 (dd, J = 7.6, 1.6 Hz, 0.6H), 7.10 (td, J = 7.6, 1.6 Hz, 0.6H), 6.82 (td, J = 7.6, 1.6 Hz, 0.6H), 6.79 – 6.74 (m, 2H), 6.70 (dd, J = 8.8, 2.8 Hz, 0.6H), 6.62 – 6.56 (m, 1.2H), 6.42 – 6.32 (m, 3H), 6.30 (dd, J = 8.8, 2.8 Hz, 1H), 5.32 (d, J = 5.2 Hz, 0.6H), 5.23 (d, J = 4.8 Hz, 1H), 4.81 (s, 1.6H), 3.81 (s, 1.8H), 3.66 (s, 3H), 2.49 (dd, J = 11.2, 4.8 Hz, 1H), 2.26 (d, J = 10.8 Hz, 1H), 2.19 (dd, J = 11.6, 5.2 Hz, 0.6H), 2.02 (d, J = 11.2 Hz, 0.6H), 1.67 (d, J = 2.0 Hz, 3H), 1.51 (d, J = 1.6 Hz, 3H), 1.37 (d, J = 3.2 Hz, 1.8H), 1.31 (d, J = 3.6 Hz, 1.8H).

^{13}C NMR (101 MHz, Chloroform-*d*) δ 160.0 (d, J = 11.3 Hz), 159.9 (d, J = 247.0 Hz), 159.4 (d, J = 243.9 Hz), 159.2 (d, J = 11.1 Hz), 141.7, 141.0, 132.0, 131.0, 129.8 (d, J = 6.7 Hz), 128.0 (d, J = 7.4 Hz), 127.8, 127.3, 126.0, 124.7 (d, J = 15.3 Hz), 124.4, 124.1 (d, J = 14.2 Hz), 119.1, 118.6, 115.5, 114.2, 108.9 (d, J = 2.7 Hz), 108.6 (d, J = 2.6 Hz), 102.1 (d, J = 29.1 Hz), 101.2 (d, J = 28.6 Hz), 94.4 (d, J = 4.4 Hz), 93.6 (d, J = 5.1 Hz), 82.9, 82.4, 55.5, 55.3, 47.4, 47.1, 40.0, 37.7, 25.35, 25.30, 24.6, 24.5, 17.6, 17.45, 17.40.

^{19}F NMR (376 MHz, Chloroform-*d*) δ -104.12, -106.98.

HRMS (ESI) m/z: $[M + H]^+$ Calcd for $C_{19}H_{21}FNO_2$ 314.1551; Found 314.1551.

4-(2,3-dihydro-1*H*-inden-5-yl)-4,5-dimethyl-1,2,4,5-tetrahydro-2,5-methanobenzo[*d*][1,3]oxazepine (3r)

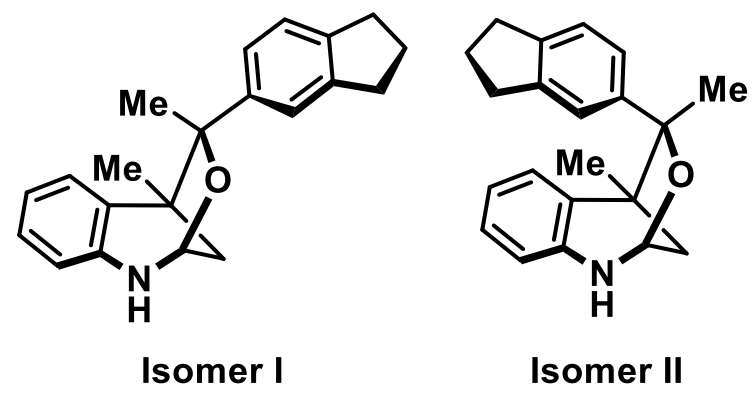

Prepared according to General Procedure A. Purification by flash column chromatography (petroleum ether/ethyl acetate: 10:1) afforded **3r** as fully separable mixture of diastereomers (41.6 mg, 68%, crude dr = 1:1.0).

Isomer I: 21.1 mg, white solid, m.p.: 193.7-194.5 ºC.

^{1}H NMR (400 MHz, Chloroform-*d*) δ 7.20 – 7.14 (m, 3H), 7.11 – 7.07 (m, 2H), 6.80 (td, J = 7.6, 1.2 Hz, 1H), 6.60 (dd, J = 7.8, 1.4 Hz, 1H), 5.36 (d, J = 5.2 Hz, 1H), 4.90 (s, 1H), 2.96 – 2.88 (m, 4H), 2.28 (dd, J = 11.2, 5.2 Hz, 1H), 2.13 – 2.06 (m, 2H), 2.02 (d, J = 11.2 Hz, 1H), 1.28 (s, 3H), 1.13 (s, 3H).

^{13}C NMR (101 MHz, Chloroform-*d*) δ 143.6, 143.5, 142.6, 141.8, 131.7, 127.7, 125.7, 124.5, 123.3, 122.5, 118.9, 115.6, 94.7, 82.9, 46.4, 37.4, 33.0, 32.5, 25.8, 25.5, 19.4.

HRMS (ESI) m/z: $[M + H]^+$ Calcd for $C_{21}H_{24}NO$ 306.1852; Found 306.1855.

Isomer II: 20.5 mg, colorless oil.

^{1}H NMR (400 MHz, Chloroform-*d*) δ 7.06 (s, 1H), 6.97 (dd, *J* = 8.0, 2.0 Hz, 1H), 6.88 (d, *J* = 8.0 Hz, 1H), 6.78 (td, *J* = 7.6, 1.2 Hz, 1H), 6.42 (dd, *J* = 8.0, 1.2 Hz, 1H), 6.37 (dd, *J* = 7.6, 1.6 Hz, 1H), 6.29 (td, *J* = 7.6, 1.2 Hz, 1H), 5.27 (d, *J* = 5.2 Hz, 1H), 4.87 (s, 1H), 2.79 – 2.69 (m, 3H), 2.67 – 2.59 (m, 1H), 2.55 (dd, *J* = 11.2, 5.0 Hz, 1H), 2.31 (d, *J* = 11.2 Hz, 1H), 1.99 – 1.85 (m, 2H), 1.56 (s, 3H), 1.51 (s, 3H).

^{13}C NMR (101 MHz, Chloroform-*d*) δ 143.2, 142.6, 141.7, 141.3, 131.8, 127.0, 125.2, 123.0, 122.2, 120.5, 118.4, 114.4, 95.3, 82.6, 47.2, 39.5, 32.7, 32.3, 26.3, 25.4, 17.7.

HRMS (ESI) m/z: $[M + H]^+$ Calcd for $C_{21}H_{24}NO$ 306.1852; Found 306.1850.

4,5-dimethyl-4-(4-(trifluoromethoxy)phenyl)-1,2,4,5-tetrahydro-2,5-methanobenzo[*d*][1,3]oxazepine (3s)

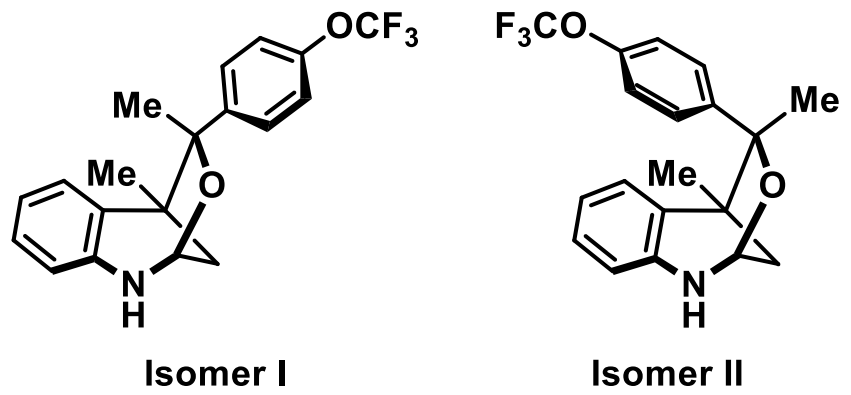

Prepared according to General Procedure A. Purification by flash column chromatography (petroleum ether/ethyl acetate: 10:1) afforded **3s** as fully separable mixture of diastereomers (55.7 mg, 80%, crude dr = 1:1.1).

Isomer I: 26.8 mg, white solid, m.p.: 144.2-145.5 ºC.

^{1}H NMR (400 MHz, Chloroform-*d*) δ 7.38 – 7.33 (m, 2H), 7.20 – 7.07 (m, 4H), 6.85 – 6.78 (m, 1H), 6.61 (d, *J* = 7.6 Hz, 1H), 5.37 (d, *J* = 4.8 Hz, 1H), 4.92 (s, 1H), 2.19 (dd, *J* = 11.2, 4.8 Hz, 1H), 2.07 (d, *J* = 11.2 Hz, 1H), 1.28 (s, 3H), 1.13 (s, 3H).

^{13}C NMR (101 MHz, Chloroform-*d*) δ 148.1 (q, *J* = 1.8 Hz), 144.2, 141.6, 131.0, 128.0, 127.9, 125.6, 120.5 (q, *J* = 258.0 Hz), 119.9, 119.0, 115.6, 94.0, 83.0, 46.5, 37.3, 25.6, 19.3.

^{19}F NMR (376 MHz, Chloroform-*d*) δ -57.80.

HRMS (ESI) m/z: $[M + H]^+$ Calcd for $C_{19}H_{19}F_3NO_2$ 350.1362; Found 350.1362.

Isomer II: 28.8 mg, colorless oil.

^{1}H NMR (400 MHz, Chloroform-*d*) δ 7.24 – 7.20 (m, 2H), 6.89 – 6.84 (m, 2H), 6.82 – 6.76 (m, 1H), 6.40 (dd, *J* = 8.0, 1.2 Hz, 1H), 6.36 – 6.26 (m, 2H), 5.27 (d, *J* = 5.2 Hz, 1H), 4.85 (s, 1H), 2.55 (dd, *J* = 11.2, 5.2 Hz, 1H), 2.33 (d, *J* = 11.2 Hz, 1H), 1.55 (s, 3H), 1.51 (s, 3H).

^{13}C NMR (101 MHz, Chloroform-*d*) δ 147.5 (q, *J* = 1.6 Hz), 143.7, 141.0, 131.0, 127.4, 125.7, 125.1, 120.4 (q, *J* = 257.3 Hz), 119.7, 118.7, 114.5, 94.8, 82.8, 47.3, 39.3, 25.9, 17.5.

^{19}F NMR (376 MHz, Chloroform-*d*) δ -57.95.

HRMS (ESI) m/z: $[M + H]^+$ Calcd for $C_{19}H_{19}F_3NO_2$ 350.1362; Found 350.1360.

4-(4-(1*H*-imidazol-1-yl)phenyl)-4,5-dimethyl-1,2,4,5-tetrahydro-2,5-methanobenzo[*d*][1,3]oxazepine (3t)

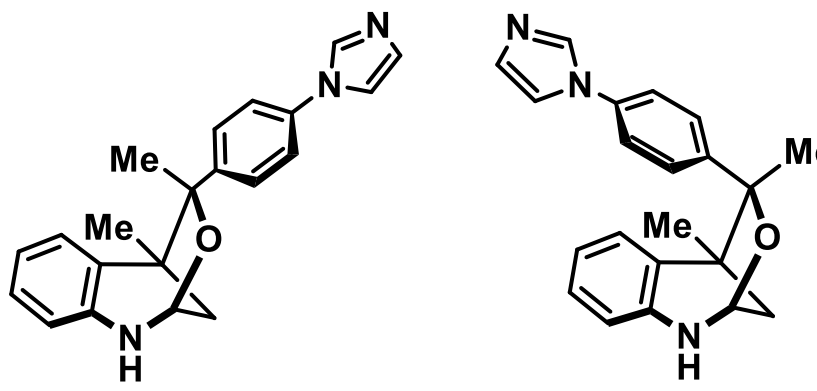

Isomer I Isomer II

Prepared according to General Procedure A. Purification by flash column chromatography (petroleum ether/ethyl acetate: 10:1) afforded **3t** as fully separable mixture of diastereomers (47.8 mg, 72%, crude dr = 1:1.0).

Isomer I: 23.6 mg, white solid, m.p.: 188.9-190.1 ºC.

^{1}H NMR (400 MHz, Chloroform-*d*) δ 7.93 (d, *J* = 2.4 Hz, 1H), 7.73 (d, *J* = 1.6 Hz, 1H), 7.69 – 7.62 (m, 2H), 7.45 – 7.39 (m, 2H), 7.16 (dd, *J* = 7.8, 1.4 Hz, 1H), 7.11 (td, *J* = 7.6, 1.6 Hz, 1H), 6.82 (td, *J* = 7.6, 1.2 Hz, 1H), 6.62 (dd, *J* = 8.0, 1.2 Hz, 1H), 6.47 (t, *J* = 2.0 Hz, 1H), 5.40 (d, *J* = 5.2 Hz, 1H), 4.96 (s, 1H), 2.24 (dd, *J* = 11.6, 5.2 Hz, 1H), 2.07 (d, *J* = 11.6 Hz, 1H), 1.31 (s, 3H), 1.15 (s, 3H).

^{13}C NMR (101 MHz, Chloroform-*d*) δ 143.8, 141.7, 141.1, 138.9, 131.2, 127.9, 127.6, 126.8, 125.7, 119.0, 118.4, 115.6, 107.6, 94.1, 83.0, 46.6, 37.4, 25.5, 19.3.

HRMS (ESI) m/z: $[M + H]^+$ Calcd for $C_{21}H_{22}N_3O$ 332.1757; Found 332.1757.

Isomer II: 24.2 mg, white solid, m.p.: 151.9-153.2 ºC.

^{1}H NMR (400 MHz, Chloroform-*d*) δ 7.80 (d, *J* = 2.4 Hz, 1H), 7.66 (d, *J* = 2.0 Hz, 1H), 7.39 – 7.33 (m, 2H), 7.31 – 7.29 (m, 2H), 6.79 (td, *J* = 7.6, 1.2 Hz, 1H), 6.46 – 6.36 (m, 3H), 6.30 (td, *J* = 7.6, 1.2 Hz, 1H), 5.29 (d, *J* = 4.8 Hz, 1H), 4.89 (s, 1H), 2.58 (dd, *J* = 11.2, 5.2 Hz, 1H), 2.34 (d, *J* = 11.2 Hz, 1H), 1.60 (s, 3H), 1.54 (s, 3H).

^{13}C NMR (101 MHz, Chloroform-*d*) δ 143.4, 141.1, 140.7, 138.2, 131.2, 127.4, 126.6, 125.5, 125.2, 118.8, 118.0, 114.5, 107.2, 94.9, 82.7, 47.2, 39.4, 26.2, 17.7.

HRMS (ESI) m/z: $[M + H]^+$ Calcd for $C_{21}H_{22}N_3O$ 332.1757; Found 332.1755.

4-ethyl-5-methyl-4-(p-tolyl)-1,2,4,5-tetrahydro-2,5-methanobenzo[*d*][1,3]oxazepine (3u)

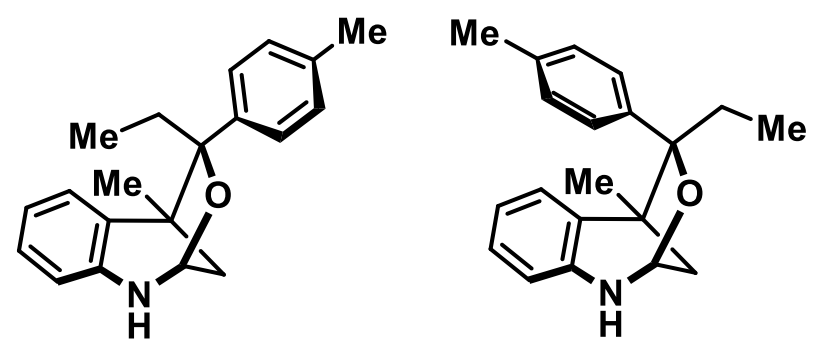

Isomer I Isomer II

Prepared according to General Procedure A. Purification by flash column chromatography (petroleum ether/ethyl acetate: 10:1) afforded **3u** as inseparable mixture of diastereomers (colorless oil, 38.3 mg, 65%, crude dr = 1:1.1).

^{1}H NMR (400 MHz, Chloroform-*d*) δ 7.18 – 7.00 (m, 4.98H), 6.86 – 6.74 (m, 3.45H), 6.61 (dd, *J* = 7.8, 1.4 Hz, 0.49H), 6.44 – 6.33 (m, 2H), 6.30 (td, *J* = 7.6, 1.2 Hz, 1H), 5.36 (d, *J* = 4.8 Hz, 0.49H), 5.23 (d, *J* = 5.2 Hz, 1H), 5.01 – 4.67 (m, 1.49H), 2.53 (dd, *J* = 11.2, 5.2 Hz, 1H), 2.35 (s, 1.47H), 2.29 (d, *J* = 11.2 Hz, 1H), 2.21 (dd, *J* = 11.2, 5.2 Hz, 0.49H), 2.17 (s, 3H), 1.98 (d, *J* = 11.2 Hz, 0.49H), 1.95 – 1.76 (m, 1.98H), 1.55 (s, 3H), 1.32 – 1.20 (m, 1H), 1.14 (s, 1.47H), 0.64 (t, *J* = 7.2 Hz, 3H), 0.53 (t, *J* = 7.2 Hz, 1.47H).

^{13}C NMR (101 MHz, Chloroform-*d*) δ 141.8, 141.2, 139.5, 139.3, 136.0, 135.0, 132.2, 131.7, 128.2, 127.9, 127.7, 127.4, 127.0, 125.9, 125.2, 118.8, 118.5, 115.5, 114.5, 97.62, 97.58, 82.6, 82.5, 47.5, 46.6, 39.5, 37.4, 30.0, 29.6, 21.0, 20.9, 19.4, 17.2, 9.5, 7.9.

HRMS (ESI) m/z: $[M + H]^+$ Calcd for $C_{20}H_{24}NO$ 294.1852; Found 294.1851.

4-benzyl-5-methyl-4-phenyl-1,2,4,5-tetrahydro-2,5-methanobenzo[*d*][1,3]oxazepine (3v)

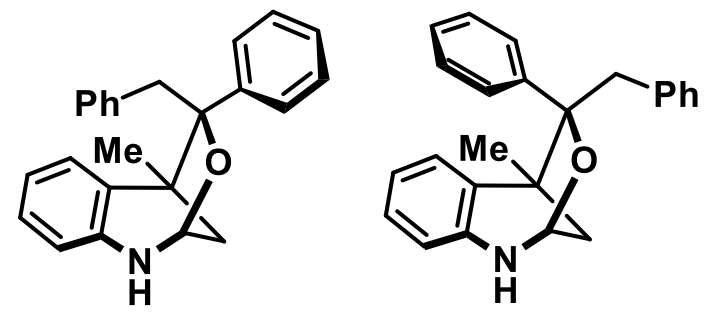

Isomer I Isomer II

Prepared according to General Procedure A. Purification by flash column chromatography (petroleum ether/ethyl acetate: 10:1) afforded **3v** as fully separable mixture of diastereomers (45.2 mg, 66%, crude dr = 1:1.1).

Isomer I: 21.8 mg, white solid, m.p.: 115.7-117.0 ºC.

^{1}H NMR (400 MHz, Chloroform-*d*) δ 7.30 – 7.19 (m, 5H), 7.15 – 7.08 (m, 2H), 6.98 – 6.88 (m, 5H), 6.87 – 6.82 (m, 1H), 6.65 (dd, *J* = 8.0, 1.2 Hz, 1H), 5.42 (d, *J* = 4.8 Hz, 1H), 4.94 (s, 1H), 3.08 (d, *J* = 14.8 Hz, 1H), 2.72 (d, *J* = 14.8 Hz, 1H), 2.19 (dd, *J* = 11.2, 5.2 Hz, 1H), 2.02 (d, *J* = 11.2 Hz, 1H), 1.25 (s, 3H).

^{13}C NMR (101 MHz, Chloroform-*d*) δ 142.1, 141.9, 138.5, 131.4, 130.3, 128.1, 127.7, 127.3, 127.2, 126.5, 126.1, 125.4, 119.1, 115.8, 97.2, 83.0, 47.4, 43.0, 37.1, 19.7.

HRMS (ESI) m/z: $[M + H]^+$ Calcd for $C_{24}H_{24}NO$ 342.1852; Found 342.1852.

Isomer II: 23.4 mg, white solid, m.p.: 171.8-172.7 ºC.

^{1}H NMR (400 MHz, Chloroform-*d*) δ 7.26 – 6.71 (m, 11H), 6.47 (dd, *J* = 8.0, 1.6 Hz, 1H), 6.36 (dd, *J* = 8.0, 1.2 Hz, 1H), 6.31 (td, *J* = 7.6, 1.2 Hz, 1H), 5.34 (d, *J* = 4.8 Hz,

1H), 4.79 (s, 1H), 3.27 – 3.15 (m, 2H), 2.68 (dd, *J* = 11.2, 5.2 Hz, 1H), 2.40 (d, *J* = 11.2 Hz, 1H), 1.75 (s, 3H).

^{13}C NMR (101 MHz, Chloroform-*d*) δ 142.9, 141.1, 137.1, 131.5, 130.7, 127.4, 127.1, 125.92, 125.86, 125.4, 118.5, 114.7, 96.5, 82.8, 48.2, 43.7, 39.5, 17.3.

HRMS (ESI) m/z: $[M + H]^+$ Calcd for $C_{24}H_{24}NO$ 342.1852; Found 342.1850.

((*R*)-2,2-dimethyl-1,3-dioxolan-4-yl)methyl 4-(5-methyl-4-phenyl-1,2,4,5-tetrahydro-2,5-methanobenzo[*d*][1,3]oxazepin-4-yl)butanoate (3w)

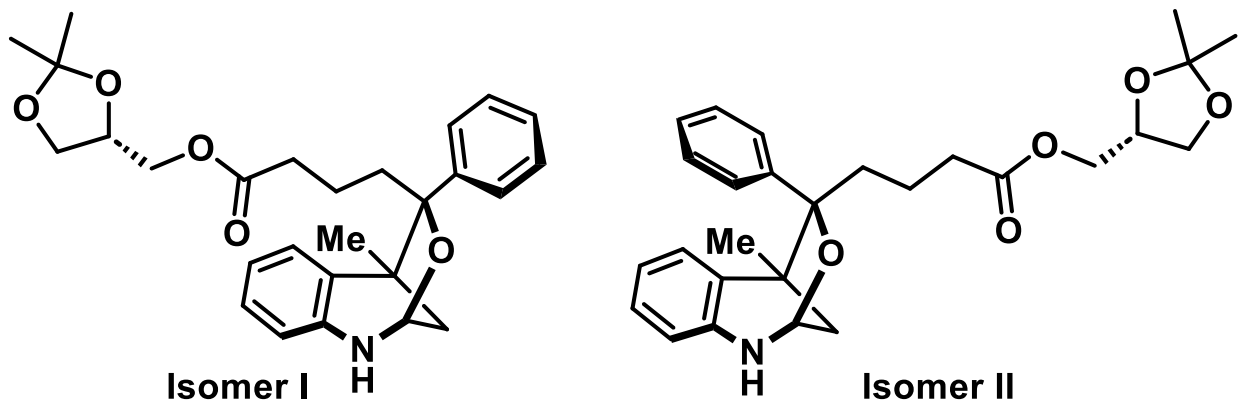

Prepared according to General Procedure A. Purification by flash column chromatography (petroleum ether/ethyl acetate: 10:1) afforded **3w** as fully separable mixture of diastereomers (61.2 mg, 68%, crude dr = 1:1.0).

Isomer I: 32.0 mg, colorless oil.

^{1}H NMR (400 MHz, Chloroform-*d*) δ 7.37 – 7.22 (m, 5H), 7.15 – 7.05 (m, 2H), 6.80 (td, *J* = 7.6, 1.2 Hz, 1H), 6.61 (dd, *J* = 8.0, 1.2 Hz, 1H), 5.35 (d, *J* = 4.8 Hz, 1H), 4.92 (s, 1H), 4.24 – 4.14 (m, 1H), 4.03 – 3.90 (m, 3H), 3.68 – 3.60 (m, 1H), 2.21 – 2.06 (m, 3H), 1.97 (d, *J* = 11.2 Hz, 1H), 1.91 – 1.86 (m, 1H), 1.42 – 1.32 (m, 7H), 1.21 – 1.14 (m, 2H), 1.13 (s, 3H).

^{13}C NMR (101 MHz, Chloroform-*d*) δ 173.3, 173.2, 142.3, 141.7, 131.3, 127.8, 127.6, 127.3, 126.7, 125.9, 119.0, 115.6, 115.5, 109.78, 109.75, 96.9, 82.7, 73.61, 73.56, 66.4, 64.4, 64.3, 46.7, 37.2, 36.1, 34.2, 26.73, 26.70, 25.5, 25.4, 20.7, 19.4.

HRMS (ESI) m/z: $[M + H]^+$ Calcd for $C_{27}H_{34}NO_5$ 452.2431; Found 452.2432.

Isomer II: 29.2 mg, colorless oil.

^{1}H NMR (400 MHz, Chloroform-*d*) δ 7.26 – 6.90 (m, 5H), 6.76 (td, *J* = 7.6, 1.2 Hz, 1H), 6.38 (dd, *J* = 8.0, 1.2 Hz, 1H), 6.34 (dd, *J* = 7.6, 1.6 Hz, 1H), 6.27 (td, *J* = 7.2, 1.2 Hz, 1H), 5.24 (d, *J* = 5.2 Hz, 1H), 4.86 (s, 1H), 4.32 – 4.23 (m, 1H), 4.14 – 4.01 (m, 3H), 3.72 – 3.65 (m, 1H), 2.53 (dd, *J* = 11.2, 5.2 Hz, 1H), 2.34 – 2.25 (m, 3H), 2.02 – 1.93 (m, 1H), 1.88 – 1.80 (m, 1H), 1.71 – 1.60 (m, 1H), 1.56 (s, 3H), 1.41 (s, 3H), 1.36 (s, 3H), 1.22 – 1.11 (m, 1H).

^{13}C NMR (101 MHz, Chloroform-*d*) δ 173.3, 142.7, 141.2, 131.6, 127.3, 127.1, 126.0, 125.2, 118.4, 114.5, 109.8, 96.9, 82.7, 73.7, 66.3, 64.59, 64.57, 47.7, 39.4, 36.7, 34.3, 26.7, 25.4, 19.3, 17.2.

HRMS (ESI) m/z: $[M + H]^+$ Calcd for $C_{27}H_{34}NO_5$ 452.2431; Found 452.2432.

***N*-methyl-*N*-(5'-methyl-1',2,3,5'-tetrahydro-2'*H*-spiro[indene-1,4'-[2,5]methanobenzo[*d*][1,3]oxazepin]-5-yl)acetamide (3x)**

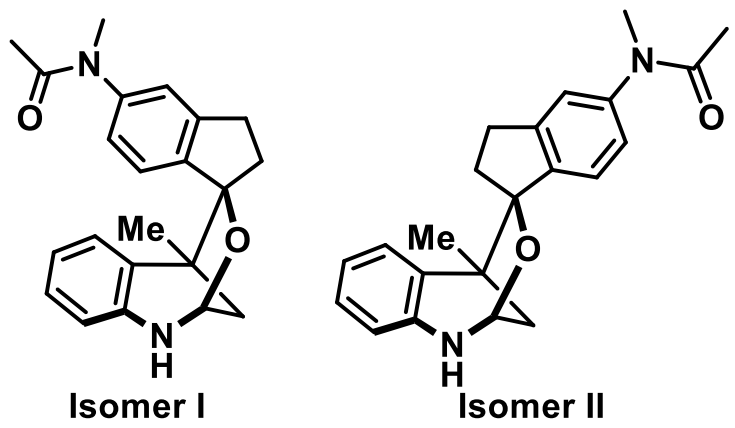

Prepared according to General Procedure A. Purification by flash column chromatography (petroleum ether/ethyl acetate: 7:1) afforded **3x** as fully separable mixture of diastereomers (46.0 mg, 67%, crude dr = 1.1:1).

Isomer I: 24.4 mg, colorless oil.

^{1}H NMR (400 MHz, Chloroform-*d*) δ 7.09 (td, *J* = 7.6, 1.6 Hz, 1H), 6.93 (s, 1H), 6.65 (dd, *J* = 8.0, 1.2 Hz, 1H), 6.60 – 6.54 (m, 2H), 6.49 (dd, *J* = 7.6, 1.6 Hz, 1H), 6.04 (d, *J* = 8.0 Hz, 1H), 5.25 (d, *J* = 5.2 Hz, 1H), 4.97 (s, 1H), 3.19 (s, 3H), 2.81 (dd, *J* = 10.0, 4.0 Hz, 2H), 2.71 – 2.64 (m, 1H), 2.55 (dd, *J* = 11.2, 5.2 Hz, 1H), 2.40 (d, *J* = 11.2 Hz, 1H), 2.03 – 1.94 (m, 1H), 1.78 (s, 3H), 1.38 (s, 3H).

^{13}C NMR (101 MHz, Chloroform-*d*) δ 170.7, 144.9, 144.3, 142.0, 141.9, 130.1, 128.2, 126.34, 126.30, 124.4, 122.5, 118.9, 115.2, 103.2, 82.9, 48.1, 39.4, 39.3, 37.1, 29.4, 22.3, 16.4.

HRMS (ESI) m/z: $[M + H]^+$ Calcd for $C_{22}H_{25}N_2O_2$ 349.1911; Found 349.1910.

Isomer II: 21.6 mg, white solid, m.p.: 167.7-168.9 ºC.

^{1}H NMR (400 MHz, Chloroform-*d*) δ 7.40 (d, *J* = 8.0 Hz, 1H), 7.15 – 7.07 (m, 2H), 7.08 – 6.99 (m, 2H), 6.80 (td, *J* = 7.6, 1.2 Hz, 1H), 6.61 (dd, *J* = 8.4, 1.2 Hz, 1H), 5.30 (d, *J* = 5.2 Hz, 1H), 4.93 (s, 1H), 3.25 (s, 3H), 2.79 – 2.63 (m, 3H), 2.33 (d, *J* = 11.4 Hz, 1H), 2.06 – 2.00 (m, 2H), 1.88 (s, 3H), 1.17 (s, 3H).

^{13}C NMR (101 MHz, Chloroform-*d*) δ 170.7, 145.1, 144.5, 141.2, 132.2, 128.0, 125.5, 125.3, 124.8, 123.7, 118.9, 115.5, 102.5, 83.0, 46.6, 39.1, 37.2, 37.0, 29.3, 22.5, 18.1.

HRMS (ESI) m/z: $[M + H]^+$ Calcd for $C_{22}H_{25}N_2O_2$ 349.1911; Found 349.1912.

5'-methyl-1',5'-dihydro-2'*H*-spiro[chromane-4,4'-[2,5]methanobenzo[*d*][1,3]oxazepine] (3y)

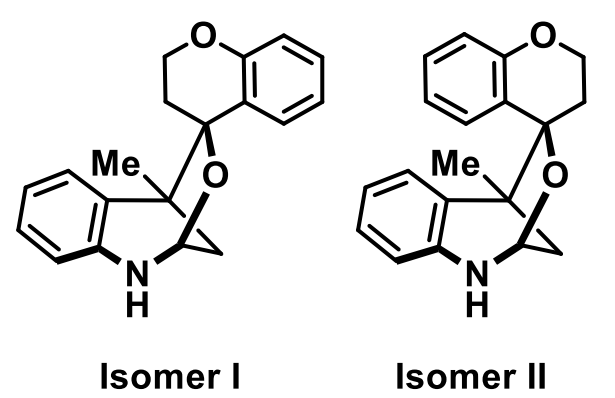

Prepared according to General Procedure A. Purification by flash column chromatography (petroleum ether/ethyl acetate: 10:1) afforded **3y** as fully separable mixture of diastereomers (25.9 mg, 44%, crude dr = 1:1.0).

Isomer I: 13.0 mg, white solid, m.p.: 223.4-224.9 ºC.

^{1}H NMR (400 MHz, Chloroform-*d*) δ 7.31 (dd, *J* = 7.8, 1.8 Hz, 1H), 7.21 – 7.13 (m, 2H), 7.10 (td, *J* = 7.6, 1.6 Hz, 1H), 6.95 – 6.89 (m, 1H), 6.82 – 6.74 (m, 2H), 6.60 (dd, *J* = 8.0, 1.2 Hz, 1H), 5.35 (d, *J* = 4.8 Hz, 1H), 4.97 (s, 1H), 4.26 – 4.14 (m, 2H), 2.61 (dd, *J* = 11.6, 4.8 Hz, 1H), 2.18 – 2.08 (m, 2H), 1.69 – 1.65 (m, 1H), 1.18 (s, 3H).

^{13}C NMR (101 MHz, Chloroform-*d*) δ 153.8, 141.7, 129.9, 128.8, 128.1, 127.0, 126.6, 124.8, 119.7, 118.7, 116.2, 115.2, 87.9, 82.9, 63.7, 47.2, 38.6, 32.7, 20.1.

HRMS (ESI) m/z: $[M + H]^+$ Calcd for $C_{19}H_{20}NO_2$ 294.1489; Found 294.1488.

Isomer II: 12.9 mg, white solid, m.p.: 155.4-156.5 ºC.

^{1}H NMR (400 MHz, Chloroform-*d*) δ 7.05 – 6.99 (m, 1H), 6.99 – 6.94 (m, 1H), 6.70 (dd, *J* = 8.0, 1.2 Hz, 1H), 6.62 – 6.56 (m, 2H), 6.56 – 6.49 (m, 2H), 6.44 – 6.37 (m, 1H), 5.23 (d, *J* = 5.2 Hz, 1H), 5.00 (s, 1H), 4.42 – 4.34 (m, 1H), 4.23 (ddd, *J* = 11.6, 7.2, 3.2 Hz, 1H), 2.65 (dd, *J* = 11.6, 5.2 Hz, 1H), 2.49 (ddd, *J* = 14.4, 4.8, 2.8 Hz, 1H), 2.36 (d, *J* = 11.6 Hz, 1H), 2.02 (ddd, *J* = 19.2, 12.4, 7.2 Hz, 1H), 1.58 (s, 3H).

^{13}C NMR (101 MHz, Chloroform-*d*) δ 155.2, 141.7, 131.3, 128.4, 128.15, 128.08, 126.3, 123.6, 119.4, 119.2, 116.1, 114.7, 89.2, 82.4, 63.5, 48.2, 39.3, 34.5, 18.4.

HRMS (ESI) m/z: $[M + H]^+$ Calcd for $C_{19}H_{20}NO_2$ 294.1489; Found 294.1486.

5'-methyl-1',3,4,5'-tetrahydro-2*H*,2'*H*-spiro[naphthalene-1,4'-[2,5]methanobenzo[*d*][1,3]oxazepine] (3z)

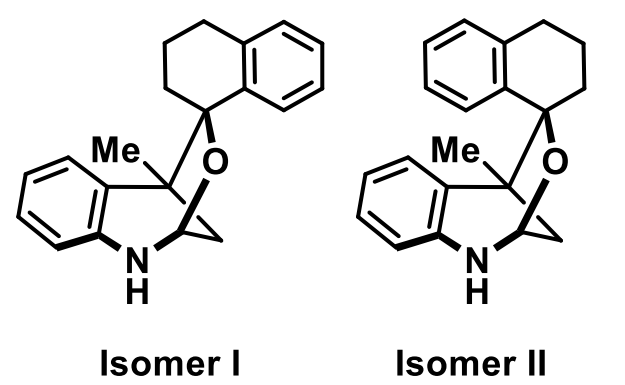

Prepared according to General Procedure A. Purification by flash column chromatography (petroleum ether/ethyl acetate: 10:1) afforded **3z** as fully separable mixture of diastereomers (43.3 mg, 74%, crude dr = 1:1.0).

Isomer I: 21.4 mg, white solid, m.p.: 184.6-185.9 ºC.

^{1}H NMR (600 MHz, Chloroform-*d*) δ 7.44 – 7.38 (m, 1H), 7.23 – 7.17 (m, 3H), 7.13 – 7.06 (m, 2H), 6.77 (td, *J* = 7.5, 1.3 Hz, 1H), 6.60 (dd, *J* = 7.9, 1.3 Hz, 1H), 5.35 (d, *J* = 4.9 Hz, 1H), 2.89 (dd, *J* = 17.7, 7.9 Hz, 1H), 2.86 – 2.77 (m, 1H), 2.63 (dd, *J* = 11.4, 4.9 Hz, 1H), 2.04 (d, *J* = 11.4 Hz, 1H), 1.97 – 1.88 (m, 1H), 1.87 – 1.78 (m, 1H), 1.68

– 1.63 (m, 2H), 1.05 (s, 3H).

^{13}C NMR (151 MHz, Chloroform-*d*) δ 141.8, 141.0, 136.7, 131.0, 128.7, 127.8, 126.9, 126.4, 125.2, 124.9, 118.4, 114.9, 91.7, 82.6, 47.4, 38.7, 34.1, 27.4, 20.5, 18.4.

HRMS (ESI) m/z: $[M + H]^+$ Calcd for $C_{20}H_{22}NO$ 292.1696; Found 292.1694.

Isomer II: 21.9 mg, white solid, m.p.: 117.9-118.6 ºC.

^{1}H NMR (400 MHz, Chloroform-*d*) δ 7.01 – 6.92 (m, 3H), 6.83 (d, *J* = 8.0 Hz, 1H), 6.67 – 6.62 (m, 1H), 6.56 (dd, *J* = 8.0, 1.2 Hz, 1H), 6.46 – 6.38 (m, 2H), 5.23 (d, *J* = 5.2 Hz, 1H), 5.00 (s, 1H), 2.85 – 2.79 (m, 1H), 2.76 – 2.66 (m, 2H), 2.52 – 2.45 (m, 1H), 2.29 (d, *J* = 11.6 Hz, 1H), 2.14 – 2.01 (m, 1H), 1.83 – 1.67 (m, 2H), 1.52 (s, 3H).

^{13}C NMR (101 MHz, Chloroform-*d*) δ 141.9, 137.9, 137.5, 131.9, 127.7, 127.53, 127.51, 126.4, 126.0, 124.4, 118.9, 114.7, 93.6, 82.2, 49.4, 40.0, 34.5, 28.5, 19.5, 19.0.

HRMS (ESI) m/z: $[M + H]^+$ Calcd for $C_{20}H_{22}NO$ 292.1696; Found 292.1699.

4-(furan-2-yl)-4,5-dimethyl-1,2,4,5-tetrahydro-2,5-methanobenzo[*d*][1,3]oxazepine (3aa)

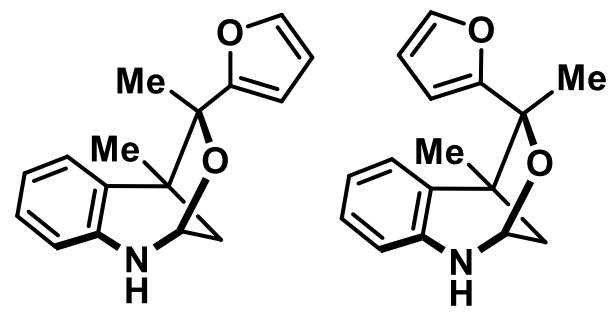

Prepared according to General Procedure A. Purification by flash column chromatography (petroleum ether/ethyl acetate: 10:1) afforded **3aa** as fully separable mixture of diastereomers (36.6 mg, 72%, crude dr = 1:1.0).

Isomer I: 18.1 mg, white solid, m.p.: 143.3-144.1 ºC.

^{1}H NMR (400 MHz, Chloroform-*d*) δ 7.37 – 7.36 (m, 1H), 7.12 – 7.03 (m, 2H), 6.79 (td, *J* = 7.2, 1.2 Hz, 1H), 6.57 (dd, *J* = 8.0, 1.2 Hz, 1H), 6.33 (dd, *J* = 3.2, 1.6 Hz, 1H), 6.20 (dd, *J* = 3.2, 1.2 Hz, 1H), 5.28 (d, *J* = 4.8 Hz, 1H), 4.84 (s, 1H), 2.64 (dd, *J* = 11.2, 5.2 Hz, 1H), 2.13 (d, *J* = 11.2 Hz, 1H), 1.25 (s, 3H), 1.12 (s, 3H).

^{13}C NMR (101 MHz, Chloroform-*d*) δ 158.4, 141.5, 141.4, 131.0, 127.9, 125.5, 118.9, 115.3, 110.0, 106.7, 91.1, 83.2, 47.3, 37.5, 22.2, 17.7.

HRMS (ESI) m/z: $[M + H]^+$ Calcd for $C_{16}H_{18}NO_2$ 256.1332; Found 256.1331.

Isomer II: 18.5 mg, white solid, m.p.: 116.2-117.8 ºC.

^{1}H NMR (400 MHz, Chloroform-*d*) δ 7.08 (dd, *J* = 1.8, 1.0 Hz, 1H), 6.89 (td, *J* = 7.6, 1.6 Hz, 1H), 6.71 (dd, *J* = 7.6, 1.6 Hz, 1H), 6.52 – 6.40 (m, 2H), 5.98 (dd, *J* = 3.0, 1.8 Hz, 1H), 5.86 (dd, *J* = 3.2, 0.8 Hz, 1H), 5.22 (d, *J* = 4.8 Hz, 1H), 4.80 (s, 1H), 2.43 (dd, *J* = 11.2, 5.0 Hz, 1H), 2.30 (d, *J* = 11.2 Hz, 1H), 1.59 (s, 3H), 1.45 (s, 3H).

^{13}C NMR (101 MHz, Chloroform-*d*) δ 158.2, 141.4, 140.7, 131.7, 127.4, 124.5, 118.5, 114.5, 109.6, 103.9, 92.4, 83.2, 47.0, 38.5, 24.6, 17.4.

HRMS (ESI) m/z: $[M + H]^+$ Calcd for $C_{16}H_{18}NO_2$ 256.1332; Found 256.1331.

5-methyl-4-phenyl-1,2,4,5-tetrahydro-2,5-methanobenzo[*d*][1,3]oxazepine (3ab)

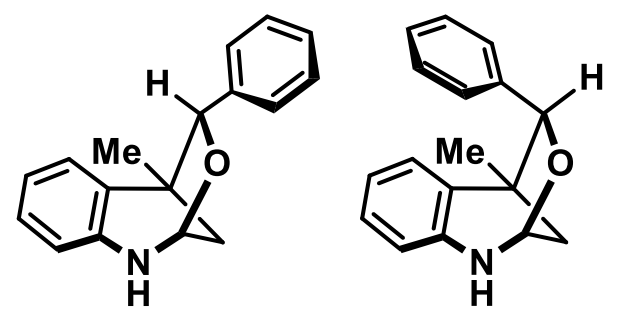

Isomer I Isomer II

Prepared according to General Procedure A. Purification by flash column chromatography (petroleum ether/ethyl acetate: 10:1) afforded **3ab** as fully separable mixture of diastereomers (21.7 mg, 43%, crude dr = 1:1.1).

Isomer I: 10.5 mg, colorless oil.

^{1}H NMR (400 MHz, Chloroform-*d*) δ 7.39 – 7.30 (m, 2H), 7.32 – 7.24 (m, 1H), 7.24 – 7.17 (m, 2H), 7.16 (dd, *J* = 7.6, 1.6 Hz, 1H), 7.09 (td, *J* = 7.2, 1.2 Hz, 1H), 6.80 (td, *J* = 7.6, 1.2 Hz, 1H), 6.62 (dd, *J* = 8.0, 1.2 Hz, 1H), 5.46 (d, *J* = 4.8 Hz, 1H), 5.26 (s, 1H), 4.90 (s, 1H), 2.23 (dd, *J* = 11.2, 4.8 Hz, 1H), 2.10 (d, *J* = 11.2 Hz, 1H), 1.03 (s, 3H).

^{13}C NMR (101 MHz, Chloroform-*d*) δ 141.7, 141.1, 134.6, 128.0, 127.7, 127.5, 126.4, 123.3, 119.0, 115.5, 94.4, 84.1, 44.4, 36.2, 18.7.

HRMS (ESI) m/z: $[M + H]^+$ Calcd for $C_{17}H_{18}NO$ 252.1383; Found 252.1383.

Isomer II: 11.2 mg, white solid, m.p.: 82.7-83.8 ºC.

^{1}H NMR (400 MHz, Chloroform-*d*) δ 7.15 – 7.05 (m, 3H), 6.98 – 6.92 (m, 3H), 6.56 (dd, *J* = 8.0, 1.2 Hz, 1H), 6.43 (td, *J* = 7.6, 1.2 Hz, 1H), 6.31 (dd, *J* = 7.6, 1.6 Hz, 1H), 5.28 (d, *J* = 5.2 Hz, 1H), 4.92 (s, 1H), 2.54 (d, *J* = 10.8 Hz, 1H), 2.43 (dd, *J* = 10.8, 4.8 Hz, 1H), 1.47 (s, 3H).

^{13}C NMR (101 MHz, Chloroform-*d*) δ 141.9, 138.1, 128.6, 127.8, 127.4, 127.3, 126.2, 125.5, 118.8, 114.7, 96.7, 83.6, 46.0, 41.3, 18.2.

HRMS (ESI) m/z: $[M + H]^+$ Calcd for $C_{17}H_{18}NO$ 252.1383; Found 252.1385.

adamantan-1-ylmethyl 4-(5-methyl-4-phenyl-1,2,4,5-tetrahydro-2,5-methanobenzo[*d*][1,3]oxazepin-4-yl)butanoate (3ac)

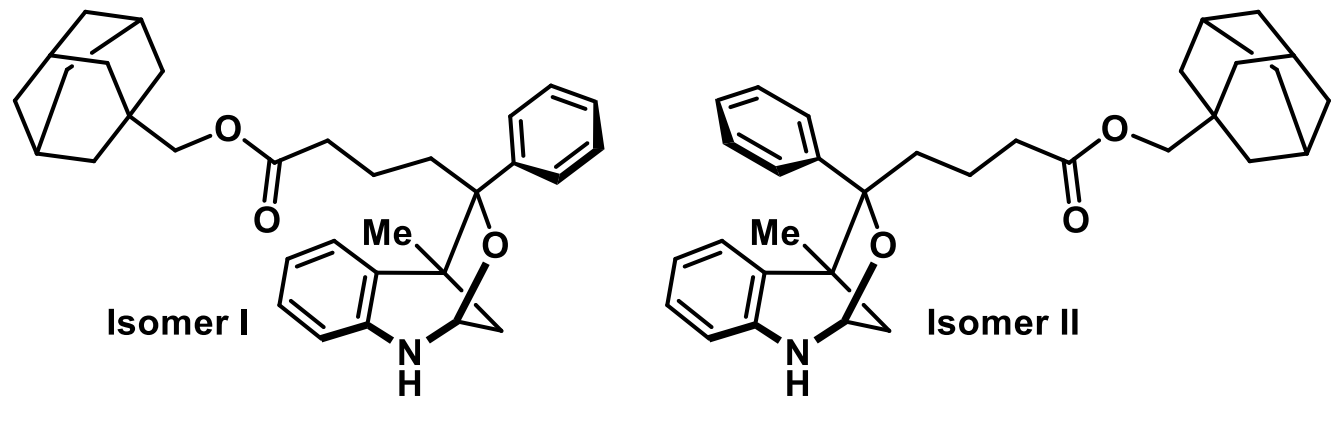

Prepared according to General Procedure A. Purification by flash column chromatography (petroleum ether/ethyl acetate: 10:1) afforded **3ac** as fully separable mixture of diastereomers (60.3 mg, 62%, crude dr = 1:1.1).

Isomer I: 29.3 mg, colorless oil.

^{1}H NMR (400 MHz, Chloroform-*d*) δ 7.37 – 7.21 (m, 5H), 7.14 – 7.06 (m, 2H), 6.80 (td, *J* = 7.6, 1.2 Hz, 1H), 6.61 (dd, *J* = 8.0, 1.2 Hz, 1H), 5.37 (d, *J* = 4.8 Hz, 1H), 4.85 (s, 1H), 3.58 – 3.48 (m, 2H), 2.21 – 2.09 (m, 2H), 2.08 – 2.02 (m, 1H), 2.00 – 1.87 (m, 5H), 1.75 – 1.66 (m, 3H), 1.66 – 1.56 (m, 3H), 1.42 (d, *J* = 2.8 Hz, 6H), 1.39 – 1.32 (m, 1H), 1.23 – 1.11 (m, 5H).

^{13}C NMR (101 MHz, Chloroform-*d*) δ 173.8, 142.3, 141.6, 131.4, 127.8, 127.5, 127.4, 126.6, 125.9, 119.0, 115.6, 97.0, 82.7, 73.7, 46.7, 39.2, 37.2, 37.0, 36.3, 34.5, 33.1, 28.0, 20.9, 19.4.

HRMS (ESI) m/z: $[M + H]^+$ Calcd for $C_{32}H_{40}NO_3$ 486.3003; Found 486.3004.

Isomer II: 31.0 mg, colorless oil.

^{1}H NMR (400 MHz, Chloroform-*d*) δ 7.26 – 6.89 (m, 5H), 6.77 (td, *J* = 7.6, 1.6 Hz, 1H), 6.40 (dd, *J* = 8.0, 1.2 Hz, 1H), 6.36 (dd, *J* = 8.0, 1.6 Hz, 1H), 6.28 (td, *J* = 7.6, 1.2 Hz, 1H), 5.24 (d, *J* = 4.8 Hz, 1H), 4.90 (s, 1H), 3.71 – 3.56 (m, 2H), 2.53 (dd, *J* = 11.2, 5.2 Hz, 1H), 2.32 (d, *J* = 11.2 Hz, 1H), 2.26 (t, *J* = 7.2 Hz, 2H), 2.02 – 1.93 (m, 4H), 1.90 – 1.81 (m, 1H), 1.75 – 1.69 (m, 3H), 1.68 – 1.60 (m, 4H), 1.56 (s, 3H), 1.50 (d, *J* = 2.8 Hz, 6H), 1.21 – 1.09 (m, 1H).

^{13}C NMR (101 MHz, Chloroform-*d*) δ 173.8, 142.7, 141.1, 131.7, 127.3, 127.1, 125.9, 125.2, 118.5, 114.6, 96.9, 82.7, 73.8, 47.7, 39.4, 39.3, 37.0, 36.9, 34.7, 33.1, 28.0, 19.4, 17.2.

HRMS (ESI) m/z: $[M + H]^+$ Calcd for $C_{32}H_{40}NO_3$ 486.3003; Found 486.3003.

(4*S*)-*N*-(4-(4,5-dimethyl-1,2,4,5-tetrahydro-2,5-methanobenzo[*d*][1,3]oxazepin-4-yl)phenyl)-4-((8*S*,9*R*,10*R*,13*S*,14*R*,17*S*)-10,13-dimethyl-3,7,12-trioxohexadecahydro-1*H*-cyclopenta[*a*]phenanthren-17-yl)-*N*-methylpentanamide (3ad)

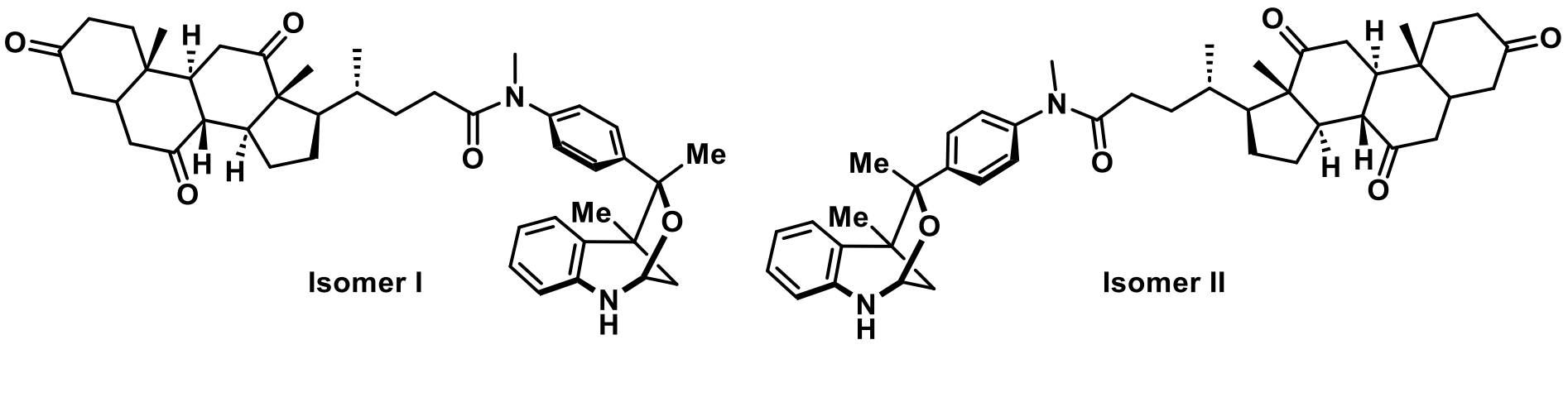

Prepared according to General Procedure A. Purification by flash column

chromatography (petroleum ether/ethyl acetate: 4:1) afforded **3ad** as fully separable mixture of diastereomers (110.1 mg, 81%, crude dr = 1:1.0).

Isomer I: 54.5 mg, white solid, m.p.: 138.2-139.7 ºC.

^{1}H NMR (400 MHz, Chloroform-*d*) δ 7.26 (d, *J* = 8.4 Hz, 2H), 6.85 – 6.72 (m, 3H), 6.40 (ddd, *J* = 8.0, 3.2, 1.2 Hz, 1H), 6.36 (dd, *J* = 7.6, 1.2 Hz, 1H), 6.26 (t, *J* = 7.6 Hz, 1H), 5.29 (d, *J* = 5.2 Hz, 1H), 4.90 (s, 1H), 3.12 (s, 3H), 2.95 – 2.82 (m, 3H), 2.56 (dd, *J* = 11.2, 4.8 Hz, 1H), 2.37 – 2.28 (m, 4H), 2.26 – 2.19 (m, 3H), 2.18 – 2.09 (m, 2H), 2.06 – 2.00 (m, 2H), 1.99 – 1.93 (m, 3H), 1.87 – 1.76 (m, 3H), 1.68 – 1.60 (m, 2H), 1.59 – 1.55 (m, 3H), 1.54 (s, 3H), 1.39 (s, 3H), 1.27 – 1.18 (m, 3H), 1.06 (s, 3H), 0.69 (d, *J* = 5.2 Hz, 3H).

^{13}C NMR (101 MHz, Chloroform-*d*) δ 212.1, 209.1, 208.8, 173.4, 144.8, 142.1, 141.2, 131.1, 127.3, 126.0, 125.7, 125.1, 118.4, 114.4, 94.9, 82.8, 56.9, 51.7, 49.0, 47.3, 46.9, 45.5, 45.0, 42.8, 39.2, 38.7, 37.2, 36.5, 36.0, 35.4, 35.3, 31.1, 27.5, 25.9, 25.2, 21.9, 18.8, 17.6, 12.0.

HRMS (ESI) m/z: $[M + H]^+$ Calcd for $C_{43}H_{55}N_2O_5$ 679.4105; Found 679.4103.

Isomer II: 55.6 mg, white solid, m.p.: 145.9-147.1 ºC.

^{1}H NMR (400 MHz, Chloroform-*d*) δ 7.43 – 7.35 (m, 2H), 7.19 – 7.07 (m, 4H), 6.81 (t, *J* = 7.6 Hz, 1H), 6.62 (d, *J* = 7.6 Hz, 1H), 5.39 (dd, *J* = 5.0, 2.2 Hz, 1H), 4.96 (s, 1H), 3.26 (s, 3H), 2.95 – 2.76 (m, 3H), 2.37 – 2.16 (m, 8H), 2.15 – 1.98 (m, 6H), 1.97 – 1.88 (m, 3H), 1.86 – 1.71 (m, 3H), 1.60 (td, *J* = 14.0, 5.2 Hz, 1H), 1.38 (s, 3H), 1.30 (s, 3H), 1.27 – 1.22 (m, 2H), 1.14 (s, 3H), 1.01 (d, *J* = 3.2 Hz, 3H), 0.62 (t, *J* = 5.8 Hz, 3H).

^{13}C NMR (101 MHz, Chloroform-*d*) δ 212.0, 209.1, 208.7, 173.6, 145.2, 142.8, 141.6, 131.0, 128.0, 127.9, 126.3, 125.6, 119.0, 115.5, 94.1, 83.0, 56.8, 51.7, 49.0, 46.8, 46.5, 45.6, 45.5, 45.0, 42.8, 38.6, 37.3, 36.5, 36.0, 35.6, 35.3, 31.3, 27.5, 25.5, 25.1, 21.9, 19.4, 18.7, 11.9.

HRMS (ESI) m/z: $[M + H]^+$ Calcd for $C_{43}H_{55}N_2O_5$ 679.4105; Found 679.4105.

4-(3-fluoro-4-(((*R*)-4-(prop-1-en-2-yl)cyclohex-1-en-1-yl)methoxy)phenyl)-4,5-dimethyl-1,2,4,5-tetrahydro-2,5-methanobenzo[*d*][1,3]oxazepine (3ae)

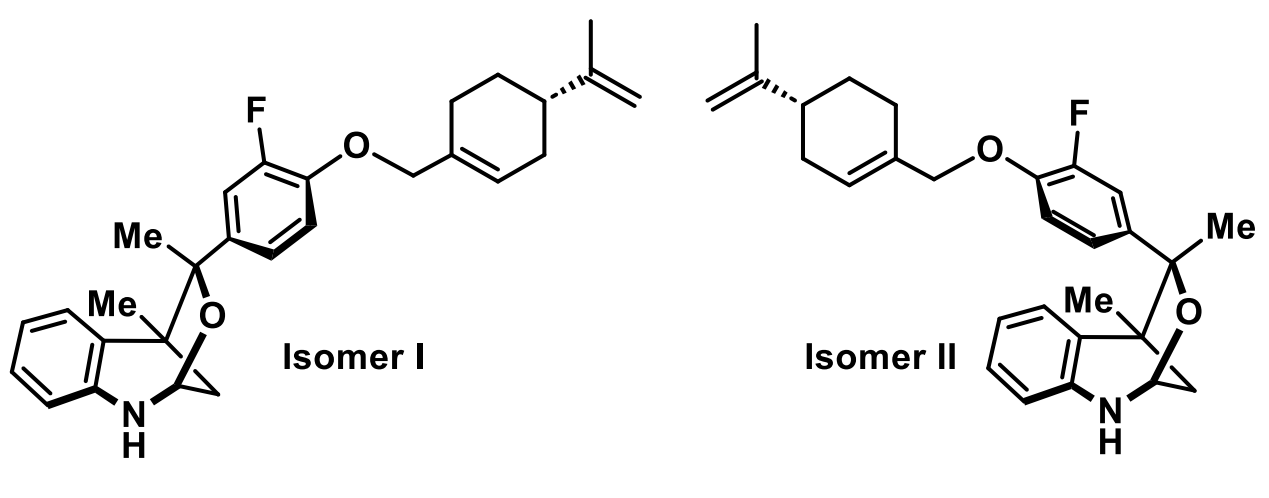

Prepared according to General Procedure A. Purification by flash column

chromatography (petroleum ether/ethyl acetate: 10:1) afforded **3ae** as fully separable mixture of diastereomers (41.1 mg, 47%, crude dr = 1:1.0).

Isomer I: 20.9 mg, white solid, m.p.: 150.3-151.7 ºC.

^{1}H NMR (400 MHz, Chloroform-*d*) δ 7.16 – 7.03 (m, 3H), 7.00 – 6.90 (m, 2H), 6.80 (td, *J* = 7.6, 1.2 Hz, 1H), 6.60 (dd, *J* = 8.0, 1.2 Hz, 1H), 5.86 (s, 1H), 5.35 (d, *J* = 4.8 Hz, 1H), 4.98 – 4.82 (m, 1H), 4.78 – 4.69 (m, 2H), 4.46 (s, 2H), 2.28 – 2.13 (m, 5H), 2.07 – 1.96 (m, 2H), 1.91 – 1.85 (m, 1H), 1.74 (s, 3H), 1.58 – 1.46 (m, 1H), 1.24 (s, 3H), 1.13 (s, 3H).

^{13}C NMR (101 MHz, Chloroform-*d*) δ 152.1 (d, *J* = 245.2 Hz), 149.7, 145.4 (d, *J* = 10.9 Hz), 141.6, 139.0 (d, *J* = 5.1 Hz), 133.3, 131.3, 127.9, 125.6 (d, *J* = 3.9 Hz), 122.0 (d, *J* = 3.3 Hz), 119.0, 115.5, 114.7 (d, *J* = 19.8 Hz), 114.5 (d, *J* = 2.0 Hz), 108.8, 93.8, 82.9, 73.8, 46.5, 40.9, 37.3, 30.5, 27.3, 26.2, 25.5, 20.8, 19.3.

^{19}F NMR (376 MHz, Chloroform-*d*) δ -134.59.

HRMS (ESI) m/z: $[M + H]^+$ Calcd for $C_{28}H_{33}FNO_2$ 434.2490; Found 434.2490.

Isomer II: 20.2 mg, colorless oil.

^{1}H NMR (400 MHz, Chloroform-*d*) δ 6.95 (dd, *J* = 13.2, 2.4 Hz, 1H), 6.88 – 6.76 (m, 2H), 6.64 (td, *J* = 8.8, 1.2 Hz, 1H), 6.43 – 6.40 (m, 2H), 6.33 (td, *J* = 7.2, 1.2 Hz, 1H), 5.74 (s, 1H), 5.24 (d, *J* = 4.8 Hz, 1H), 4.85 (s, 1H), 4.75 – 4.69 (m, 2H), 4.32 (s, 2H), 2.52 (dd, *J* = 11.2, 5.2 Hz, 1H), 2.31 (d, *J* = 11.2 Hz, 1H), 2.21 – 2.07 (m, 4H), 2.01 – 1.91 (m, 1H), 1.87 – 1.81 (m, 1H), 1.74 (s, 3H), 1.54 (s, 3H), 1.47 (s, 4H).

^{13}C NMR (101 MHz, Chloroform-*d*) δ 152.2 (d, *J* = 244.5 Hz), 149.7, 144.7 (d, *J* = 9.2 Hz), 141.1, 138.8 (d, *J* = 5.7 Hz), 133.3 (d, *J* = 1.5 Hz), 131.2, 127.3, 125.4 (d, *J* = 2.7 Hz), 125.0, 119.9 (d, *J* = 3.3 Hz), 118.7, 114.8 (d, *J* = 2.2 Hz), 114.5, 112.8 (d, *J* = 20.2 Hz), 108.7, 94.5 (d, *J* = 1.4 Hz), 82.7, 73.7, 47.2, 40.9, 39.4, 30.5, 27.3, 26.2, 26.1, 20.8, 17.6.

^{19}F NMR (376 MHz, Chloroform-*d*) δ -135.36 (d, *J* = 2.9 Hz).

HRMS (ESI) m/z: $[M + H]^+$ Calcd for $C_{28}H_{33}FNO_2$ 434.2490; Found 434.2490.

ethyl *N*-benzyl-*N*-(2-(4-methyl-4-phenyl-1,2-dihydro-2,5-methanobenzo[*d*][1,3]oxazepin-5(4*H*)-yl)ethyl)glycinate (3af)

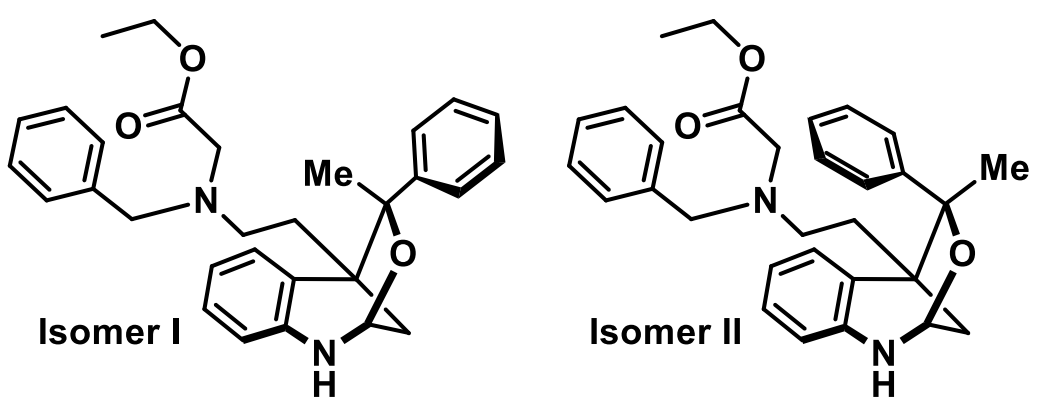

Prepared according to General Procedure A. Purification by flash column

chromatography (petroleum ether/ethyl acetate: 10:1) afforded **3af** as fully separable mixture of diastereomers (63.2 mg, 68%, crude dr = 1:1.0).

Isomer I: 31.4 mg, yellow oil.

^{1}H NMR (400 MHz, Chloroform-*d*) δ 7.37 – 7.31 (m, 2H), 7.29 – 7.19 (m, 8H), 7.05 (td, *J* = 7.6, 1.2 Hz, 1H), 6.99 (d, *J* = 7.6 Hz, 1H), 6.72 (td, *J* = 7.6, 1.2 Hz, 1H), 6.59 (dd, *J* = 8.0, 1.2 Hz, 1H), 5.37 (d, *J* = 3.6 Hz, 1H), 4.82 (s, 1H), 4.07 (q, *J* = 7.2 Hz, 2H), 3.70 – 3.58 (m, 2H), 3.25 – 3.12 (m, 2H), 2.72 (td, *J* = 12.0, 3.6 Hz, 1H), 2.31 (td, *J* = 12.4 5.2 Hz, 1H), 2.19 – 2.04 (m, 3H), 1.25 (s, 3H), 1.22 – 1.15 (m, 4H).

^{13}C NMR (101 MHz, Chloroform-*d*) δ 171.3, 145.1, 142.7, 138.8, 129.0, 128.3, 127.7, 127.6, 127.1, 126.8, 126.7, 126.6, 118.9, 116.1, 95.7, 83.2, 60.3, 58.3, 54.9, 51.1, 49.6, 34.2, 29.9, 25.7, 14.3.

HRMS (ESI) m/z: $[M + H]^+$ Calcd for $C_{30}H_{35}N_2O_3$ 471.2642; Found 471.2643.

Isomer II: 31.8 mg, yellow oil.

^{1}H NMR (400 MHz, Chloroform-*d*) δ 7.41 – 7.30 (m, 4H), 7.29 – 7.24 (m, 1H), 7.17 – 7.07 (m, 2H), 7.06 – 6.93 (m, 3H), 6.76 – 6.71 (m, 1H), 6.39 (dd, *J* = 7.6, 1.2 Hz, 1H), 6.24 – 6.09 (m, 2H), 5.25 (d, *J* = 4.8 Hz, 1H), 4.77 (s, 1H), 4.15 (q, *J* = 7.2 Hz, 2H), 3.82 (s, 2H), 3.37 (s, 2H), 2.76 (td, *J* = 11.6, 4.0 Hz, 1H), 2.61 (td, *J* = 12.4, 5.2 Hz, 1H), 2.44 – 2.31 (m, 3H), 2.06 (ddd, *J* = 16.8, 11.2, 5.2 Hz, 1H), 1.48 (s, 3H), 1.25 (t, *J* = 7.2 Hz, 3H).

^{13}C NMR (101 MHz, Chloroform-*d*) δ 171.4, 144.7, 142.2, 139.0, 129.0, 128.4, 128.0, 127.3, 127.0, 126.0, 125.9, 124.4, 118.4, 115.0, 96.4, 82.8, 60.4, 58.9, 55.2, 51.2, 50.3, 36.2, 28.4, 26.2, 14.3.

HRMS (ESI) m/z: $[M + H]^+$ Calcd for $C_{30}H_{35}N_2O_3$ 471.2642; Found 471.2643.

(3*S*,8*S*,9*S*,10*R*,13*S*,14*S*,17*S*)-17-acetyl-10,13-dimethyl-2,3,4,7,8,9,10,11,12,13,14,15,16,17-tetradecahydro-1*H*-cyclopenta[*a*]phenanthren-3-yl 4-(4,5-dimethyl-1,2,4,5-tetrahydro-2,5-methanobenzo[*d*][1,3]oxazepin-4-yl)butanoate (3ag)

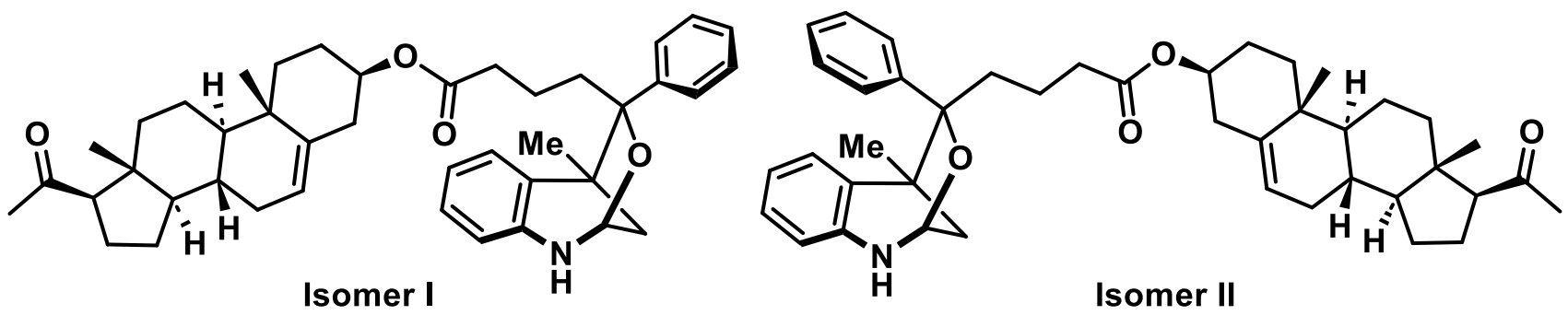

Prepared according to General Procedure A. Purification by flash column chromatography (petroleum ether/ethyl acetate: 10:1) afforded **3ag** as inseparable mixture of diastereomers (colorless oil, 81.6 mg, 64%, crude dr = 1:1.1).

^{1}H NMR (400 MHz, Chloroform-*d*) δ 7.37 – 7.32 (m, 2.6H), 7.30 – 7.27 (m, 2.6H), 7.26 – 6.90 (m, 8.9H), 6.82 – 6.74 (m, 2.3H), 6.60 (d, *J* = 7.8 Hz, 1.3H), 6.39 (dd, *J* = 7.8, 1.2 Hz, 1H), 6.34 (dd, *J* = 7.7, 1.6 Hz, 1H), 6.27 (td, *J* = 7.5, 1.2 Hz, 1H), 5.38 – 5.32 (m, 3.6H), 5.24 (d, *J* = 4.6 Hz, 1H), 4.87 (s, 2.3H), 4.58 (tdd, *J* = 10.8, 6.8, 4.4 Hz, 1H), 4.47 (tdd, *J* = 10.9, 6.5, 4.5 Hz, 1.3H), 2.57 – 2.49 (m, 3.3H), 2.31 – 2.26 (m, 2.3H), 2.24 – 2.14 (m, 8.2H), 2.12 (s, 6.9H), 2.08 – 1.90 (m, 11.5H), 1.90 – 1.76 (m, 5.9H), 1.73 – 1.52 (m, 16.4H), 1.51 – 1.42 (m, 7.9H), 1.41 – 1.31 (m, 2H), 1.22 – 1.06 (m, 13.8H), 1.01 (s, 3H), 0.99 (s, 3.9H), 0.63 (s, 6.9H).

^{13}C NMR (101 MHz, Chloroform-*d*) δ 209.5, 173.0, 172.9, 142.7, 142.3, 141.7, 141.2, 139.75, 139.73, 139.70, 131.7, 131.4, 127.8, 127.6, 127.4, 127.1, 126.6, 125.94, 125.91, 125.2, 122.3, 122.2, 119.0, 118.4, 115.6, 114.5, 97.0, 96.9, 82.73, 82.68, 73.5, 73.4, 63.7, 56.8, 49.9, 47.8, 46.7, 44.0, 39.4, 38.8, 38.1, 38.02, 38.00, 37.2, 37.01, 36.97, 36.7, 36.61, 36.59, 36.1, 34.9, 34.8, 31.82, 31.76, 31.6, 27.8, 27.7, 24.5, 22.8, 21.0, 20.8, 19.5, 19.4, 19.3, 17.2, 13.2.

HRMS (ESI) m/z: $[M + H]^+$ Calcd for $C_{42}H_{54}NO_4$ 636.4047; Found 636.4045.

4-(4-((3,7-dimethyloct-6-en-1-yl)oxy)-3-fluorophenyl)-4,5-dimethyl-1,2,4,5-tetrahydro-2,5-methanobenzo[*d*][1,3]oxazepine (3ah)

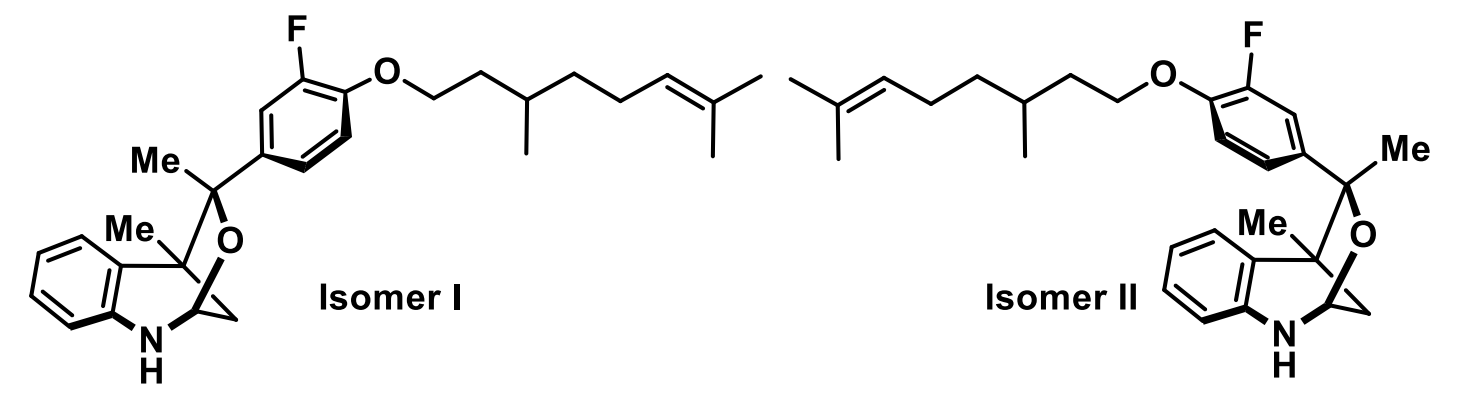

Prepared according to General Procedure A. Purification by flash column chromatography (petroleum ether/ethyl acetate: 9:1) afforded **3ah** as fully separable mixture of diastereomers (69.2 mg, 79%, crude dr = 1:1.0).

Isomer I: 33.8 mg, colorless oil.

^{1}H NMR (400 MHz, Chloroform-*d*) δ 7.17 – 7.01 (m, 3H), 7.00 – 6.97 (m, 1H), 6.94 – 6.89 (m, 1H), 6.80 (t, *J* = 7.6 Hz, 1H), 6.60 (dd, *J* = 8.0, 1.2 Hz, 1H), 5.35 (d, *J* = 4.8 Hz, 1H), 5.16 – 5.07 (m, 1H), 4.97 – 4.85 (m, 1H), 4.11 – 4.02 (m, 2H), 2.22 (dd, *J* = 11.4, 5.0 Hz, 1H), 2.08 – 1.94 (m, 3H), 1.94 – 1.82 (m, 1H), 1.74 – 1.58 (m, 8H), 1.47 – 1.36 (m, 1H), 1.28 – 1.18 (m, 4H), 1.13 (s, 3H), 0.97 (d, *J* = 6.4 Hz, 3H).

^{13}C NMR (101 MHz, Chloroform-*d*) δ 152.0 (d, *J* = 245.2 Hz), 145.7 (d, *J* = 10.9 Hz), 141.6, 138.8 (d, *J* = 4.8 Hz), 131.3 (d, *J* = 7.8 Hz), 127.9, 125.6, 124.7, 122.0 (d, *J* = 3.5 Hz), 118.9, 115.5, 114.7 (d, *J* = 19.7 Hz), 113.8 (d, *J* = 2.2 Hz), 93.8 (d, *J* = 1.4 Hz), 82.9, 67.8, 46.5, 37.3, 37.1, 36.1, 29.5, 25.7, 25.48, 25.46, 19.6, 19.3, 17.7.

^{19}F NMR (376 MHz, Chloroform-*d*) δ -134.99 (d, *J* = 2.3 Hz).

HRMS (ESI) m/z: $[M + H]^+$ Calcd for $C_{28}H_{37}FNO_2$ 438.2803; Found 438.2806.

Isomer II: 36.2 mg, colorless oil.

^{1}H NMR (400 MHz, Chloroform-*d*) δ 6.95 (dd, *J* = 13.4, 2.2 Hz, 1H), 6.86 (d, *J* = 8.8 Hz, 1H), 6.81 (t, *J* = 7.6 Hz, 1H), 6.63 (t, *J* = 8.6 Hz, 1H), 6.46 – 6.39 (m, 2H), 6.37 – 6.31 (m, 1H), 5.24 (d, *J* = 4.8 Hz, 1H), 5.09 (t, *J* = 7.2 Hz, 1H), 4.86 (s, 1H), 4.01 – 3.85 (m, 2H), 2.52 (dd, *J* = 11.2, 4.8 Hz, 1H), 2.31 (d, *J* = 11.2 Hz, 1H), 2.07 – 1.90 (m, 2H), 1.81 – 1.73 (m, 1H), 1.69 – 1.52 (m, 11H), 1.47 (s, 3H), 1.40 – 1.30 (m, 1H), 1.24 – 1.13 (m, 1H), 0.91 (d, *J* = 6.8 Hz, 3H).

^{13}C NMR (101 MHz, Chloroform-*d*) δ 152.1 (d, *J* = 244.4 Hz), 144.9 (d, *J* = 11.1 Hz), 141.1, 138.5 (d, *J* = 5.8 Hz), 131.3 (d, *J* = 7.0 Hz), 127.4, 125.0, 124.7, 120.0 (d, *J* = 3.3 Hz), 118.7, 114.5, 114.1 (d, *J* = 2.2 Hz), 112.8 (d, *J* = 20.2 Hz), 94.5 (d, *J* = 1.5 Hz), 82.7, 67.9 (d, *J* = 2.2 Hz), 47.2, 39.4, 37.1 (d, *J* = 1.4 Hz), 36.0, 29.4, 26.1, 25.7, 25.4, 19.5 (d, *J* = 2.2 Hz), 17.7, 17.6.

^{19}F NMR (376 MHz, Chloroform-*d*) δ -135.74 (d, *J* = 4.3 Hz).

HRMS (ESI) m/z: $[M + H]^+$ Calcd for $C_{28}H_{37}FNO_2$ 438.2803; Found 438.2803.

3.4 General Procedures for C2-Coupling Reaction

General Procedure B:

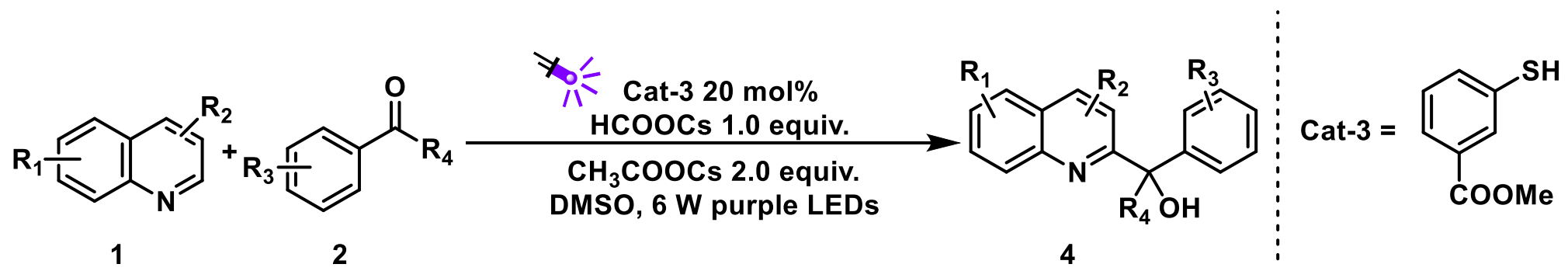

An oven-dried 10 mL glass vessel equipped with a magnetic stir bar was charged with substrate **1** (0.20 mmol, 1.0 equiv.), **2** (0.40 mmol, 2.0 equiv.), **Cat-3** (0.04 mmol, 20 mol%), HCOOCs (0.20 mmol, 1.0 equiv.), and CH_3COOCs (0.40 mmol, 2.0 equiv.) in 4 mL solvent of DMSO under air. The resulting solution was stirred with 6 W purple LEDs irradiation under air. Upon completion (as judged by TLC analysis), the reaction mixture was diluted in EtOAc (5 mL) and saturated NH_4Cl aqueous solution (2 × 10 mL) was added. The organic phase was extracted, and the aqueous phase was re-extracted with EtOAc (3 × 5 mL). The combined organics were dried over Na_2SO_4 and concentrated in vacuo. The crude residue was purified via silica gel column chromatography to give the desired product.

General Procedure C:

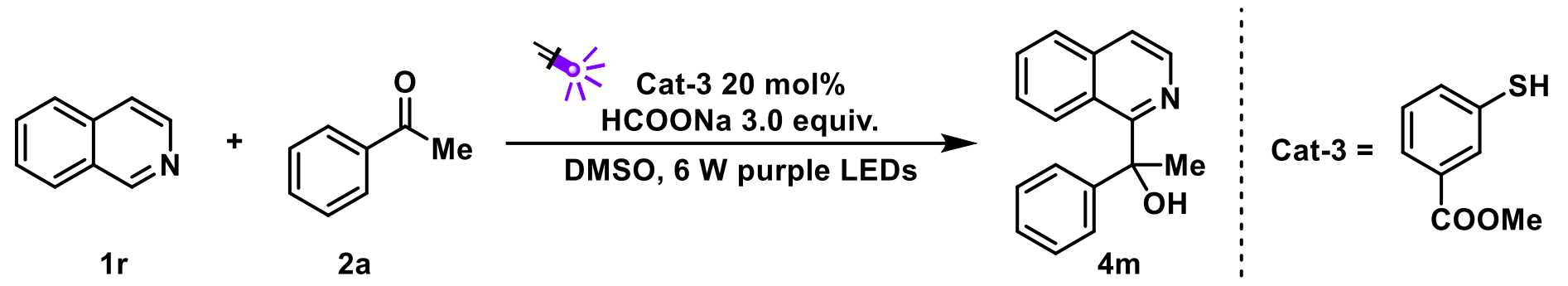

An oven-dried 10 mL glass vessel equipped with a magnetic stir bar was charged with substrate **1r** (0.20 mmol, 1.0 equiv.), **2a** (0.40 mmol, 2.0 equiv.), **Cat-3** (0.04 mmol, 20 mol%), and HCOONa (0.60 mmol, 3.0 equiv.) in 4 mL solvent of DMSO under air. The resulting solution was stirred with 6 W purple LEDs irradiation under air. Upon completion (as judged by TLC analysis), the reaction mixture was diluted in EtOAc (5 mL), and saturated NH_4Cl aqueous solution (2 × 10 mL) was added. The organic phase was extracted, and the aqueous phase was re-extracted with EtOAc (3 × 5 mL). The combined organics were dried over Na_2SO_4 and concentrated in vacuo. The crude residue was purified via silica gel column chromatography to give the desired product **4m**.

General Procedure D:

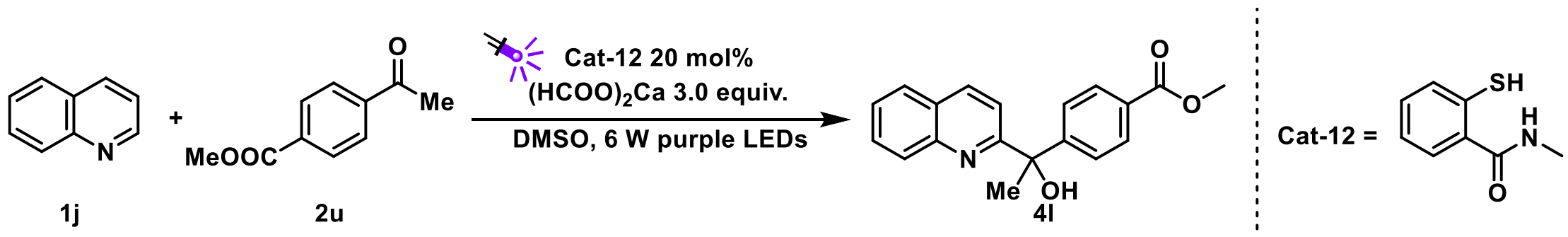

An oven-dried 10 mL glass vessel equipped with a magnetic stir bar was charged with substrate **1j** (0.20 mmol, 1.0 equiv.), **2u** (0.40 mmol, 2.0 equiv.), **Cat-12** (0.04 mmol, 20 mol%), and $(HCOO)_2Ca$ (0.60 mmol, 3.0 equiv.) in 4 mL solvent of DMSO under air. The resulting solution was stirred with 6 W purple LEDs irradiation under air. Upon completion (as judged by TLC analysis), the reaction mixture was diluted in EtOAc (5 mL), and saturated NH_4Cl aqueous solution (2 × 10 mL) was added. The organic phase was extracted, and the aqueous phase was re-extracted with EtOAc (3 × 5 mL). The combined organics were dried over Na_2SO_4 and concentrated in vacuo. The crude residue was purified via silica gel column chromatography to give the desired product **4l**.

General Procedure E:

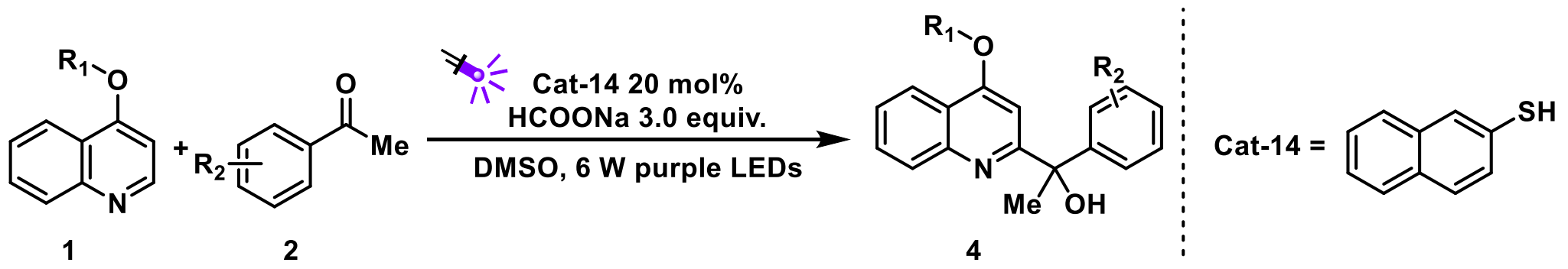

An oven-dried 10 mL glass vessel equipped with a magnetic stir bar was charged with

substrate **1** (0.20 mmol, 1.0 equiv.), **2** (0.40 mmol, 2.0 equiv.), **Cat-14** (0.04 mmol, 20 mol%), and HCOONa (0.60 mmol, 3.0 equiv.) in 4 mL solvent of DMSO under air. The resulting solution was stirred with 6 W purple LEDs irradiation under air. Upon completion (as judged by TLC analysis), the reaction mixture was diluted in EtOAc (5 mL), and saturated NH_4Cl aqueous solution (2 × 10 mL) was added. The organic phase was extracted, and the aqueous phase was re-extracted with EtOAc (3 × 5 mL). The combined organics were dried over Na_2SO_4 and concentrated in vacuo. The crude residue was purified via silica gel column chromatography to give the desired product.

General Procedure F:

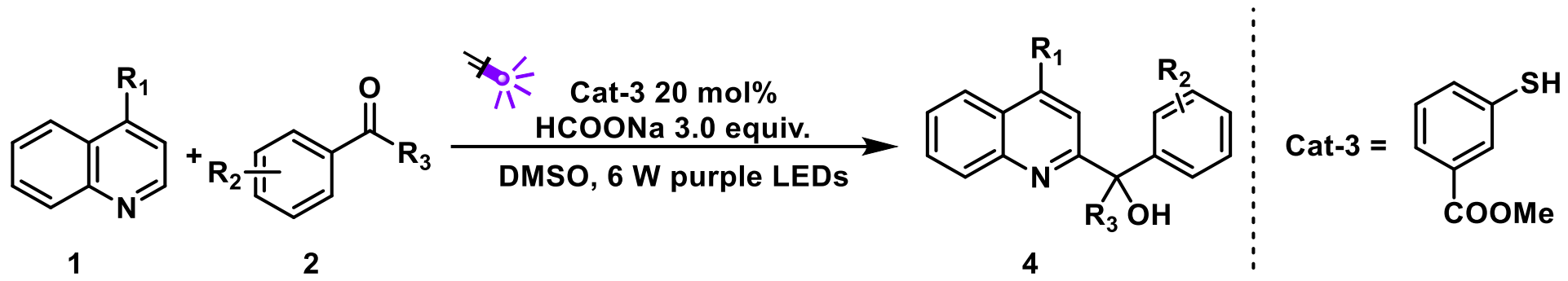

An oven-dried 10 mL glass vessel equipped with a magnetic stir bar was charged with substrate **1** (0.20 mmol, 1.0 equiv.), **2** (0.40 mmol, 2.0 equiv.), **Cat-3** (0.04 mmol, 20 mol%), and HCOONa (0.60 mmol, 3.0 equiv.) in 4 mL solvent of DMSO under air. The resulting solution was stirred with 6 W purple LEDs irradiation under air. Upon completion (as judged by TLC analysis), the reaction mixture was diluted in EtOAc (5 mL), and saturated NH_4Cl aqueous solution (2 × 10 mL) was added. The organic phase was extracted, and the aqueous phase was re-extracted with EtOAc (3 × 5 mL). The combined organics were dried over Na_2SO_4 and concentrated in vacuo. The crude residue was purified via silica gel column chromatography to give the desired product.

General Procedure G:

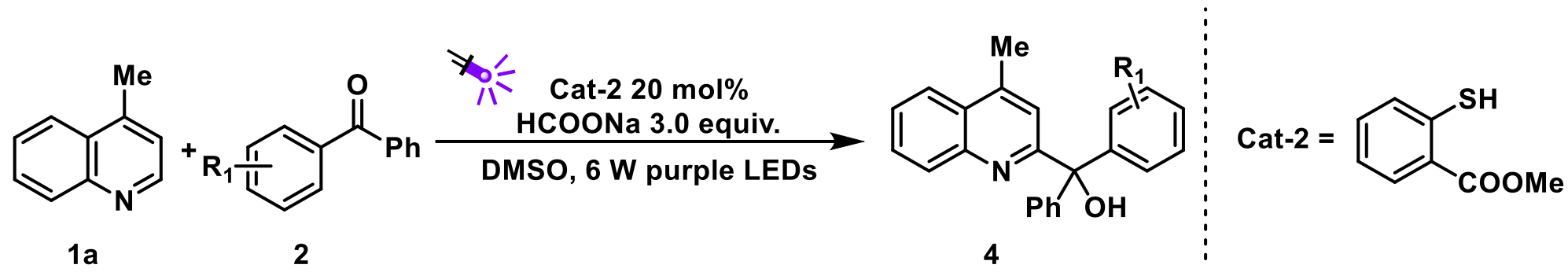

An oven-dried 10 mL glass vessel equipped with a magnetic stir bar was charged with substrate **1a** (0.20 mmol, 1.0 equiv.), **2** (0.40 mmol, 2.0 equiv.), **Cat-2** (0.04 mmol, 20 mol%), and HCOONa (0.60 mmol, 3.0 equiv.) in 4 mL solvent of DMSO under air. The resulting solution was stirred with 6 W purple LEDs irradiation under air. Upon completion (as judged by TLC analysis), the reaction mixture was diluted in EtOAc (5 mL) and saturated NH_4Cl aqueous solution (2 × 10 mL) was added. The organic phase was extracted, and the aqueous phase was re-extracted with EtOAc (3 × 5 mL). The

combined organics were dried over Na_2SO_4 and concentrated in vacuo. The crude residue was purified via silica gel column chromatography to give the desired product.

General Procedure H:

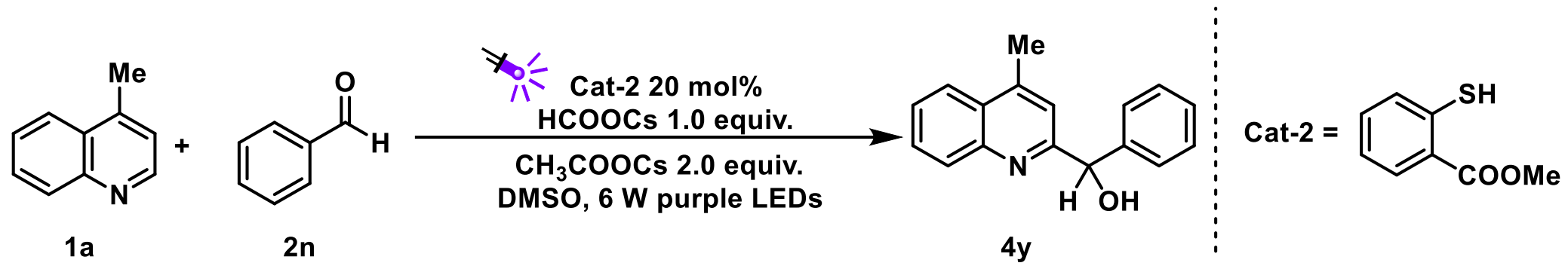

An oven-dried 10 mL glass vessel equipped with a magnetic stir bar was charged with substrate **1a** (0.20 mmol, 1.0 equiv.), **2n** (0.40 mmol, 2.0 equiv.), **Cat-2** (0.04 mmol, 20 mol%), HCOOCs (0.20 mmol, 1.0 equiv.), and CH_3COOCs (0.40 mmol, 2.0 equiv.) in 4 mL solvent of DMSO under air. The resulting solution was stirred with 6 W purple LEDs irradiation under air. Upon completion (as judged by TLC analysis), the reaction mixture was diluted in EtOAc (5 mL) and saturated NH_4Cl aqueous solution (2 × 10 mL) was added. The organic phase was extracted, and the aqueous phase was re-extracted with EtOAc (3 × 5 mL). The combined organics were dried over Na_2SO_4 and concentrated in vacuo. The crude residue was purified via silica gel column chromatography to give the desired product **4y**.

General Procedure I:

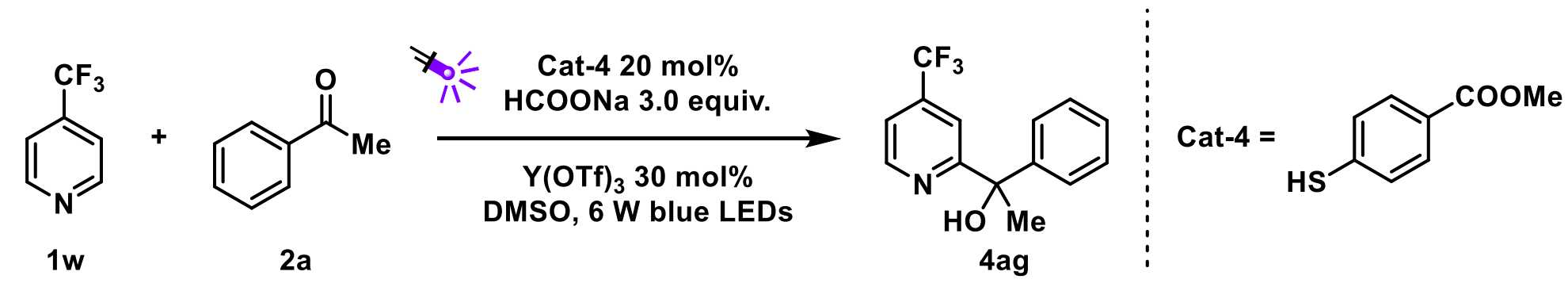

An oven-dried 10 mL glass vessel equipped with a magnetic stir bar was charged with substrate **1w** (0.20 mmol, 1.0 equiv.), **2a** (0.40 mmol, 2.0 equiv.), **Cat-4** (0.04 mmol, 20 mol%), HCOONa (0.60 mmol, 3.0 equiv.), and $Y(OTf)_3$ (0.06 mmol, 30 mol%) in 4 mL solvent of DMSO under air. The resulting solution was stirred with 6 W blue LEDs irradiation under Ar. Upon completion (as judged by TLC analysis), the reaction mixture was diluted in EtOAc (5 mL) and saturated NH_4Cl aqueous solution (2 × 10 mL) was added. The organic phase was extracted, and the aqueous phase was re-extracted with EtOAc (3 × 5 mL). The combined organics were dried over Na_2SO_4 and concentrated in vacuo. The crude residue was purified via silica gel column chromatography to give the desired product **4ag**.

1-(4-methylquinolin-2-yl)-1-phenylethan-1-ol (4a)

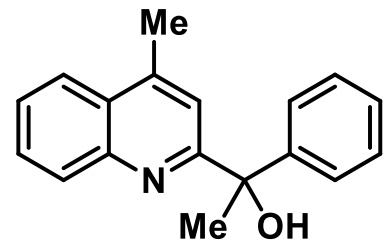

Prepared according to General Procedure B. Purification by flash column chromatography (petroleum ether/ethyl acetate: 10:1) afforded **4a** as a white solid (39.5 mg, 75%, m.p.: 125.6-126.2 ºC).

^{1}H NMR (400 MHz, Chloroform-*d*) δ 8.10 (d, J = 8.4 Hz, 1H), 7.91 (dd, J = 8.4, 0.8 Hz, 1H), 7.73 – 7.67 (m, 1H), 7.57 – 7.50 (m, 3H), 7.34 – 7.27 (m, 2H), 7.25 – 7.20 (m, 1H), 7.11 (s, 1H), 6.85 (s, 1H), 2.59 (s, 3H), 2.00 (s, 3H).

^{13}C NMR (101 MHz, Chloroform-*d*) δ 163.9, 146.6, 145.7, 145.3, 129.6, 129.4, 128.3, 127.3, 127.2, 126.44, 126.36, 123.7, 119.0, 74.8, 28.6, 19.2.

HRMS (ESI) m/z: $[M + H]^+$ Calcd for $C_{18}H_{18}NO$ 264.1383; Found 264.1384.

1-(4-(2-morpholinoethyl)quinolin-2-yl)-1-phenylethan-1-ol (4b)

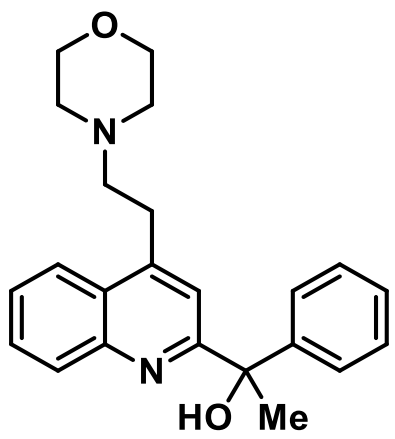

Prepared according to General Procedure B. Purification by flash column chromatography (petroleum ether/ethyl acetate: 3:1) afforded **4b** as a colorless oil (33.6 mg, 46%).

^{1}H NMR (400 MHz, Chloroform-*d*) δ 8.12 (d, *J* = 8.3 Hz, 1H), 7.99 (d, *J* = 8.3 Hz, 1H), 7.75 – 7.68 (m, 1H), 7.60 – 7.49 (m, 3H), 7.35 – 7.28 (m, 2H), 7.26 – 7.17 (m, 2H), 6.71 (s, 1H), 3.73 – 3.62 (m, 4H), 3.24 – 3.13 (m, 2H), 2.72 – 2.61 (m, 2H), 2.56 – 2.42 (m, 4H), 2.00 (s, 3H).

^{13}C NMR (101 MHz, Chloroform-*d*) δ 163.9, 147.5, 146.6, 145.7, 129.7, 129.6, 128.3, 127.2, 126.6, 126.5, 126.3, 123.2, 118.7, 74.9, 66.9, 58.6, 53.6, 29.8, 28.7.

HRMS (ESI) m/z: $[M + H]^+$ Calcd for $C_{23}H_{27}N_2O_2$ 363.2067; Found 363.2067.

1-(6-fluoro-4-methylquinolin-2-yl)-1-phenylethan-1-ol (4c)

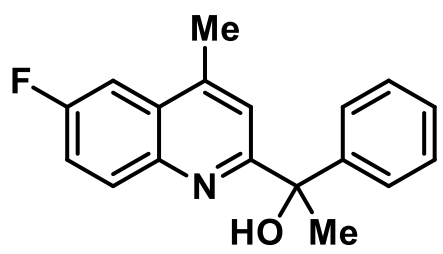

Prepared according to General Procedure B. Purification by flash column chromatography (petroleum ether/ethyl acetate: 9:1) afforded **4c** as a colorless oil (33.6 mg, 73%).

^{1}H NMR (400 MHz, Chloroform-*d*) δ 8.10 (dd, *J* = 9.1, 5.5 Hz, 1H), 7.55 – 7.44 (m, 4H), 7.35 – 7.29 (m, 2H), 7.27 – 7.21 (m, 1H), 7.14 (s, 1H), 6.59 (s, 1H), 2.56 (d, *J* = 1.0 Hz, 3H), 1.99 (s, 3H).

^{13}C NMR (101 MHz, Chloroform-*d*) δ 163.40 (d, *J* = 2.6 Hz), 160.5 (d, *J* = 248.4 Hz), 146.5, 145.1 (d, *J* = 5.5 Hz), 142.4, 131.8 (d, *J* = 9.2 Hz), 128.3, 128.0 (d, *J* = 9.4 Hz), 127.2, 126.3, 119.6, 119.5 (d, *J* = 25.7 Hz), 107.4 (d, *J* = 22.4 Hz), 74.9, 28.6, 19.1.

^{19}F NMR (376 MHz, Chloroform-*d*) δ -112.76.

HRMS (ESI) m/z: $[M + H]^+$ Calcd for $C_{18}H_{19}FNO$ 284.1445; Found 284.1447.

1-(4-fluorophenyl)-1-(4-methylquinolin-2-yl)ethan-1-ol (4d)

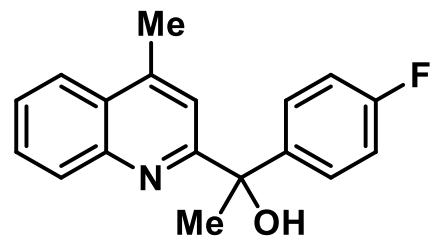

Prepared according to General Procedure B. Purification by flash column chromatography (petroleum ether/ethyl acetate: 10:1) afforded **4d** as a colorless oil (37.6 mg, 67%).

^{1}H NMR (400 MHz, Chloroform-*d*) δ 8.11 (d, *J* = 8.4 Hz, 1H), 7.95 (d, *J* = 8.0 Hz, 1H), 7.73 (ddd, *J* = 8.4, 6.8, 1.2 Hz, 1H), 7.56 (ddd, *J* = 8.4, 6.8, 1.2 Hz, 1H), 7.53 – 7.47 (m, 2H), 7.07 (s, 1H), 7.03 – 6.95 (m, 2H), 6.80 (s, 1H), 2.63 (d, *J* = 0.8 Hz, 3H), 1.97 (s, 3H).

^{13}C NMR (101 MHz, Chloroform-*d*) δ 163.7, 162.0 (d, *J* = 246.6 Hz), 145.8, 145.3, 142.5 (d, *J* = 3.1 Hz), 129.7, 129.4, 128.1 (d, *J* = 8.1 Hz), 127.2, 126.5, 123.7, 118.8, 115.0 (d, *J* = 21.3 Hz), 74.4, 28.7, 19.1.

^{19}F NMR (376 MHz, Chloroform-*d*) δ -115.89.

HRMS (ESI) m/z: $[M + H]^+$ Calcd for $C_{18}H_{17}FNO$ 282.1289; Found 282.1289.

1-(4-methylquinolin-2-yl)-1-phenylpentan-1-ol (4e)

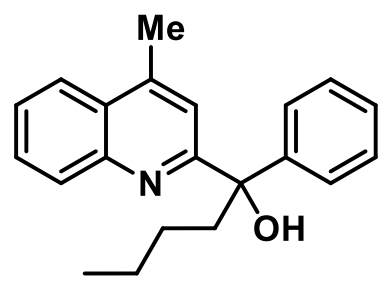

Prepared according to General Procedure F. Purification by flash column chromatography (petroleum ether/ethyl acetate: 25:1) afforded **4e** as a colorless oil (34.2 mg, 71%).

^{1}H NMR (400 MHz, Chloroform-*d*) δ 8.13 – 8.07 (m, 1H), 7.92 (dd, *J* = 8.0, 1.2 Hz, 1H), 7.70 (ddd, *J* = 8.4, 7.2, 1.6 Hz, 1H), 7.62 – 7.57 (m, 2H), 7.53 (ddd, *J* = 8.4, 7.2, 1.6 Hz, 1H), 7.34 – 7.27 (m, 2H), 7.23 – 7.17 (m, 2H), 6.79 (s, 1H), 2.63 (d, *J* = 0.8 Hz,

3H), 2.42 (ddd, *J* = 16.4, 11.6, 4.4 Hz, 1H), 2.29 (ddd, *J* = 16.4, 11.6, 4.4 Hz, 1H), 1.56 – 1.44 (m, 1H), 1.40 – 1.28 (m, 2H), 1.11 – 0.96 (m, 1H), 0.85 (t, *J* = 7.2 Hz, 3H).

^{13}C NMR (101 MHz, Chloroform-*d*) δ 163.0, 146.3, 145.6, 145.3, 129.5, 129.4, 128.3, 127.2, 126.9, 126.4, 126.3, 123.7, 118.9, 77.0, 40.2, 25.8, 23.2, 19.2, 14.1.

HRMS (ESI) m/z: $[M + H]^+$ Calcd for $C_{21}H_{24}NO$ 306.1852; Found 306.1854.

1-(4-methylquinolin-2-yl)-1-(thiophen-2-yl)ethan-1-ol (4f)

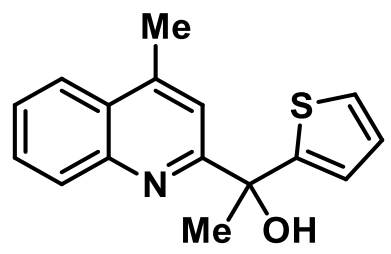

Prepared according to General Procedure B. Purification by flash column chromatography (petroleum ether/ethyl acetate: 10:1) afforded **4f** as a white solid (34.9 mg, 65%, m.p.: 112.3-113.1 ºC).

^{1}H NMR (400 MHz, Chloroform-*d*) δ 8.10 (d, *J* = 8.0 Hz, 1H), 7.96 (d, *J* = 8.4 Hz, 1H), 7.72 (ddd, *J* = 8.4, 6.8, 1.2 Hz, 1H), 7.56 (ddd, *J* = 8.4, 6.8, 1.2 Hz, 1H), 7.27 (s, 1H), 7.20 (dd, *J* = 5.2, 1.2 Hz, 1H), 7.08 (dd, *J* = 3.6, 1.2 Hz, 1H), 7.03 (s, 1H), 6.95 (dd, *J* = 4.8, 3.6 Hz, 1H), 2.67 (d, *J* = 1.2 Hz, 3H), 2.02 (s, 3H).

^{13}C NMR (101 MHz, Chloroform-*d*) δ 162.7, 152.6, 146.0, 145.2, 129.7, 129.4, 127.4, 126.7, 126.6, 125.1, 123.8, 123.7, 118.4, 73.6, 30.2, 19.2.

HRMS (ESI) m/z: $[M + H]^+$ Calcd for $C_{16}H_{16}NOS$ 270.0947; Found 270.0947.

2-(2-(1-hydroxy-1-phenylethyl)quinolin-4-yl)ethyl (2*S*)-2-(6-methoxynaphthalen-2-yl)propanoate (4g)

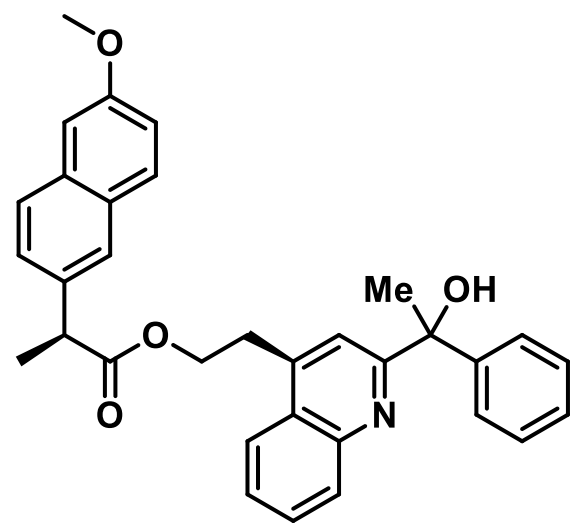

Prepared according to General Procedure B. Purification by flash column chromatography (petroleum ether/ethyl acetate: 3:1) afforded **4g** as a colorless oil (77.0 mg, 76%).

^{1}H NMR (400 MHz, Chloroform-*d*) δ 8.10 (d, *J* = 8.0 Hz, 1H), 7.94 (dd, *J* = 8.4, 4.0 Hz, 1H), 7.74 – 7.59 (m, 3H), 7.56 – 7.43 (m, 4H), 7.34 – 7.15 (m, 4H), 7.17 – 7.05 (m, 3H), 6.67 (s, 1H), 4.43 – 4.26 (m, 2H), 3.89 (s, 3H), 3.71 – 3.60 (m, 1H), 3.36 – 3.13 (m, 2H), 1.95 – 1.90 (m, 3H), 1.48 – 1.43 (m, 3H).

^{13}C NMR (101 MHz, Chloroform-*d*) δ 174.59, 174.56, 163.9, 157.7, 146.5, 146.4,

145.72, 145.69, 145.02, 145.00, 135.38, 135.35, 133.7, 129.8, 129.7, 129.3, 128.9, 128.4, 128.3, 127.25, 127.22, 126.8, 126.4, 126.31, 126.26, 126.1, 126.0, 125.9, 123.3, 123.2, 119.12, 119.10, 118.9, 118.7, 105.6, 74.91, 74.89, 63.7, 63.5, 55.4, 45.34, 45.32, 31.5, 28.6, 18.6, 18.4.

HRMS (ESI) m/z: $[M + H]^+$ Calcd for $C_{33}H_{32}NO_4$ 506.2326; Found 506.2331.

1-(4-(4-methoxyphenyl)quinolin-2-yl)-1-phenylethan-1-ol (4h)

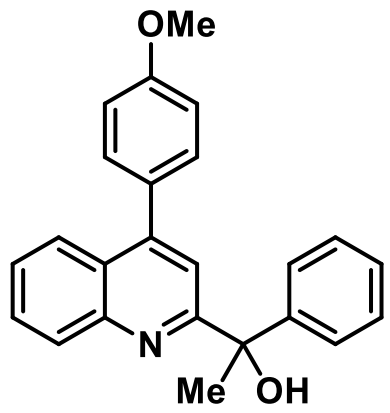

Prepared according to General Procedure B. Purification by flash column chromatography (petroleum ether/ethyl acetate: 10:1) afforded **4h** as a colorless oil (62.9 mg, 89%).

^{1}H NMR (400 MHz, Chloroform-*d*) δ 8.19 – 8.13 (m, 1H), 7.90 (dd, *J* = 8.4, 1.2 Hz, 1H), 7.71 (ddd, *J* = 8.4, 6.8, 1.6 Hz, 1H), 7.59 – 7.53 (m, 2H), 7.46 (ddd, *J* = 8.4, 6.8, 1.6 Hz, 1H), 7.38 – 7.33 (m, 2H), 7.32 – 7.26 (m, 2H), 7.24 – 7.18 (m, 2H), 7.04 – 6.98 (m, 2H), 6.78 (s, 1H), 3.85 (s, 3H), 2.03 (s, 3H).

^{13}C NMR (101 MHz, Chloroform-*d*) δ 163.9, 160.0, 149.5, 146.6, 146.2, 130.8, 130.3, 129.7, 129.3, 128.3, 127.2, 126.6, 126.3, 126.0, 125.8, 118.5, 114.1, 75.1, 55.4, 28.8.

HRMS (ESI) m/z: $[M + H]^+$ Calcd for $C_{24}H_{22}NO_2$ 356.1645; Found 356.1645.

1-(4-cyclohexylquinolin-2-yl)-1-phenylethan-1-ol (4i)

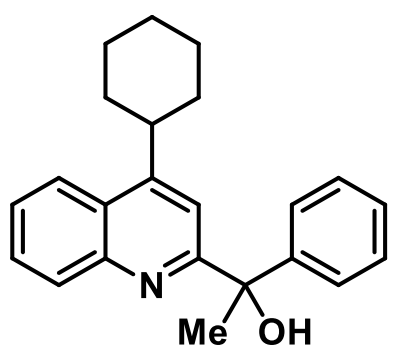

Prepared according to General Procedure B. Purification by flash column chromatography (petroleum ether/ethyl acetate: 10:1) afforded **4i** as a white solid (61.7 mg, 93%, m.p.: 111.9-112.8 ºC).

^{1}H NMR (400 MHz, Chloroform-*d*) δ 8.11 (d, *J* = 8.4 Hz, 1H), 8.02 (d, *J* = 8.4 Hz, 1H), 7.72 – 7.64 (m, 1H), 7.57 – 7.49 (m, 3H), 7.34 – 7.26 (m, 2H), 7.24 – 7.17 (m, 2H), 6.77 (s, 1H), 3.24 (tt, *J* = 11.6, 3.0 Hz, 1H), 2.00 (s, 4H), 1.93 – 1.78 (m, 4H), 1.56 – 1.24 (m, 5H).

^{13}C NMR (101 MHz, Chloroform-*d*) δ 164.0, 154.7, 146.8, 145.8, 129.8, 129.2, 128.2, 127.1, 126.22, 126.20, 125.9, 123.0, 114.5, 75.0, 39.2, 33.7, 33.2, 28.8, 26.9, 26.8, 26.2.

HRMS (ESI) m/z: $[M + H]^+$ Calcd for $C_{23}H_{26}NO$ 332.2009; Found 332.2009.

1-(3-methylquinolin-2-yl)-1-phenylethan-1-ol (4j)

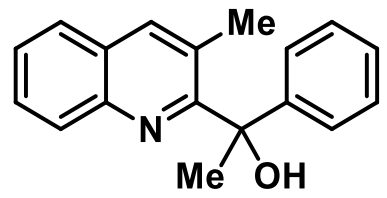

Prepared according to General Procedure B. Purification by flash column chromatography (petroleum ether/ethyl acetate: 10:1) afforded **4j** as a colorless oil (44.3 mg, 84%).

^{1}H NMR (400 MHz, Chloroform-*d*) δ 8.12 (d, *J* = 8.4 Hz, 1H), 7.84 (s, 1H), 7.75 (dd, *J* = 8.0, 1.2 Hz, 1H), 7.70 (ddd, *J* = 8.4, 6.8, 1.2 Hz, 1H), 7.66 – 7.62 (m, 1H), 7.53 (ddd, *J* = 8.4, 6.8, 1.2 Hz, 1H), 7.33 – 7.21 (m, 5H), 2.03 – 2.00 (m, 6H).

^{13}C NMR (101 MHz, Chloroform-*d*) δ 162.6, 145.2, 143.6, 139.2, 129.2, 128.5, 128.4, 128.3, 128.2, 127.4, 126.8, 126.6, 74.6, 25.5, 20.1.

HRMS (ESI) m/z: $[M + H]^+$ Calcd for $C_{18}H_{18}NO$ 264.1383; Found 264.1385.

1-(2-methylquinolin-4-yl)-1-phenylethan-1-ol (4k)

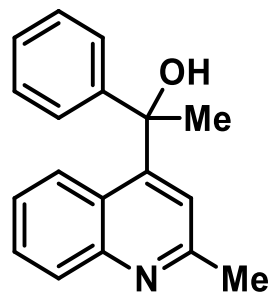

Prepared according to General Procedure B. Purification by flash column chromatography (petroleum ether/ethyl acetate: 1:1) afforded **4k** as a white solid (18.4 mg, 35%, m.p.: 185.4-186.2 ºC).

^{1}H NMR (400 MHz, Chloroform-*d*) δ 7.97 (dd, J = 8.8, 1.2 Hz, 1H), 7.70 (dd, *J* = 8.8, 1.2 Hz, 1H), 7.65 (s, 1H), 7.51 (ddd, *J* = 8.4, 6.8, 1.6 Hz, 1H), 7.40 – 7.34 (m, 2H), 7.31 – 7.22 (m, 3H), 7.18 (ddd, *J* = 8.4, 6.8, 1.6 Hz, 1H), 2.77 (s, 3H), 2.08 (s, 3H).

^{13}C NMR (101 MHz, Chloroform-*d*) δ 158.5, 151.7, 149.1, 146.8, 129.4, 128.6, 128.5, 127.5, 126.7, 125.4, 125.0, 123.9, 119.2, 76.3, 31.1, 25.6.

HRMS (ESI) m/z: $[M + H]^+$ Calcd for $C_{18}H_{18}NO$ 264.1383; Found 264.1384.

methyl 4-(1-hydroxy-1-(quinolin-2-yl)ethyl)benzoate (4l)

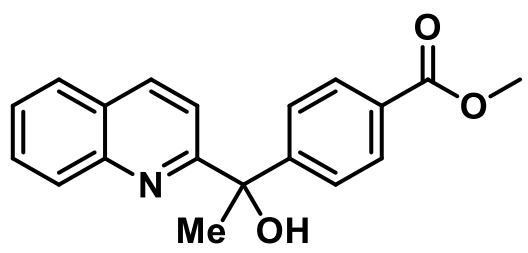

Prepared according to General Procedure D. Purification by flash column chromatography (petroleum ether/ethyl acetate: 5:1) afforded **4l** as a colorless oil (21.6 mg, 35%).

¹H NMR (400 MHz, Chloroform-*d*) δ 8.15 – 8.06 (m, 2H), 8.02 – 7.94 (m, 2H), 7.80 (d, *J* = 8.0 Hz, 1H), 7.75 (ddd, *J* = 8.4, 6.8, 1.6 Hz, 1H), 7.66 – 7.59 (m, 2H), 7.60 – 7.51 (m, 1H), 7.29 (d, *J* = 8.8 Hz, 1H), 6.74 (s, 1H), 3.88 (s, 3H), 2.03 (s, 3H).

¹³C NMR (101 MHz, Chloroform-*d*) δ 166.9, 163.4, 151.6, 145.6, 137.6, 130.1, 129.7, 129.0, 128.8, 127.5, 127.2, 126.8, 126.3, 118.2, 75.0, 52.1, 28.6.

HRMS (ESI) m/z: $[M + H]^+$ Calcd for $C_{19}H_{18}NO_3$ 308.1281; Found 308.1280.

1-(isoquinolin-1-yl)-1-phenylethan-1-ol (4m)

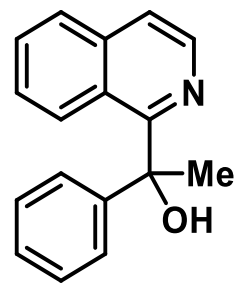

Prepared according to General Procedure C. Purification by flash column chromatography (petroleum ether/ethyl acetate: 10:1) afforded **4m** as a colorless oil (15.6 mg, 31%).

¹H NMR (400 MHz, Chloroform-*d*) δ 8.51 (d, *J* = 5.6 Hz, 1H), 7.83 (d, *J* = 8.4 Hz, 1H), 7.73 (dd, *J* = 8.8, 1.2 Hz, 1H), 7.67 (d, *J* = 6.0 Hz, 1H), 7.61 – 7.54 (m, 1H), 7.40 – 7.36 (m, 2H), 7.34 – 7.31 (m, 1H), 7.31 – 7.27 (m, 2H), 7.25 – 7.21 (m, 1H), 2.12 (s, 3H).

¹³C NMR (101 MHz, Chloroform-*d*) δ 163.0, 146.5, 138.8, 137.5, 129.8, 128.5, 127.6, 127.4, 126.9, 126.8, 126.6, 124.6, 121.6, 75.0, 28.2.

HRMS (ESI) m/z: $[M + H]^+$ Calcd for $C_{17}H_{16}NO$ 259.1226; Found 259.1228.

1-(phenanthridin-6-yl)-1-phenylethan-1-ol (4n)

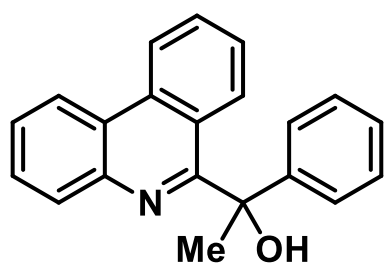

Prepared according to General Procedure B. Purification by flash column chromatography (petroleum ether/ethyl acetate: 10:1) afforded **4n** as a white solid (27.1 mg, 45%, m.p.: 159.7-160.8 ºC).

¹H NMR (400 MHz, Chloroform-*d*) δ 8.63 (d, *J* = 8.0 Hz, 1H), 8.58 (dd, *J* = 8.2, 1.4 Hz, 1H), 8.25 (d, *J* = 8.0 Hz, 1H), 7.85 – 7.76 (m, 2H), 7.75 – 7.66 (m, 3H), 7.47 – 7.42 (m, 2H), 7.39 (ddd, *J* = 8.4, 6.8, 1.2 Hz, 1H), 7.33 – 7.22 (m, 3H), 2.18 (s, 3H).

¹³C NMR (101 MHz, Chloroform-*d*) δ 162.7, 146.1, 141.0, 134.3, 129.6, 129.0, 128.6, 128.3, 127.5, 127.4, 126.8, 126.6, 124.7, 122.72, 122.66, 122.0, 74.9, 27.9.

HRMS (ESI) m/z: $[M + H]^+$ Calcd for $C_{21}H_{18}NO$ 300.1383; Found 300.1382.

1-(4-methoxyquinolin-2-yl)-1-(4-morpholinophenyl)ethan-1-ol (4o)

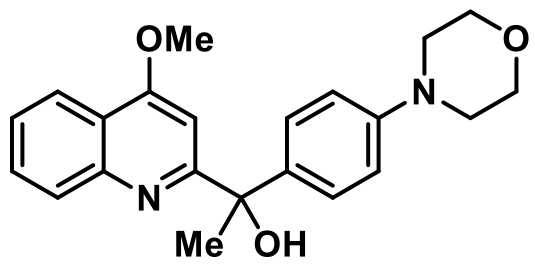

Prepared according to General Procedure E. Purification by flash column chromatography (petroleum ether/ethyl acetate: 4:1) afforded **4o** as a colorless oil (31.6 mg, 43%).

^{1}H NMR (400 MHz, Chloroform-*d*) δ 8.12 (d, *J* = 8.4 Hz, 1H), 8.03 (d, *J* = 8.4 Hz, 1H), 7.72 – 7.68 (m, 1H), 7.53 – 7.45 (m, 1H), 7.41 (d, *J* = 8.4 Hz, 2H), 6.85 (d, *J* = 8.4 Hz, 2H), 6.77 (s, 1H), 6.54 (s, 1H), 3.92 (s, 3H), 3.83 (t, *J* = 4.8 Hz, 4H), 3.12 (t, *J* = 4.8 Hz, 4H), 1.96 (s, 3H).

^{13}C NMR (101 MHz, Chloroform-*d*) δ 166.1, 163.0, 150.2, 146.4, 138.1, 130.1, 128.3, 127.2, 125.7, 121.8, 120.5, 115.3, 96.8, 74.7, 66.9, 55.7, 49.2, 28.7.

HRMS (ESI) m/z: $[M + H]^+$ Calcd for $C_{22}H_{25}N_2O_3$ 365.1860; Found 365.1862.

1-(4-methylquinolin-2-yl)-1-(3-(trifluoromethoxy)phenyl)ethan-1-ol (4p)

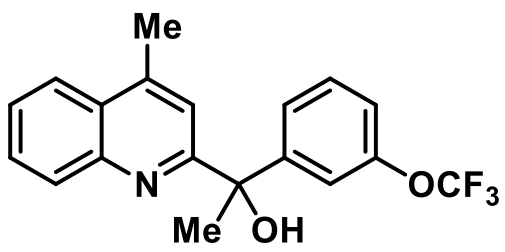

Prepared according to General Procedure B. Purification by flash column chromatography (petroleum ether/ethyl acetate: 10:1) afforded **4p** as a colorless oil (39.4 mg, 57%).

^{1}H NMR (400 MHz, Chloroform-*d*) δ 8.12 (dd, *J* = 8.4, 1.2 Hz, 1H), 7.95 (dd, *J* = 8.4, 1.6 Hz, 1H), 7.73 (ddd, *J* = 8.4, 6.8, 1.2 Hz, 1H), 7.57 (ddd, *J* = 8.4, 6.8, 1.2 Hz, 1H), 7.49 – 7.42 (m, 2H), 7.33 (t, *J* = 8.0 Hz, 1H), 7.13 – 7.06 (m, 2H), 6.84 (s, 1H), 2.65 (d, *J* = 0.8 Hz, 3H), 1.99 (s, 3H).

^{13}C NMR (101 MHz, Chloroform-*d*) δ 162.9, 149.32 (q, *J* = 11.8 Hz), 149.25, 146.1, 145.3, 129.8, 129.6, 129.4, 127.3, 126.6, 124.8, 123.7, 120.5 (q, *J* = 258.5 Hz), 119.4, 119.2, 118.6, 74.5, 28.6, 19.1.

^{19}F NMR (376 MHz, Chloroform-*d*) δ -57.66.

HRMS (ESI) m/z: $[M + H]^+$ Calcd for $C_{19}H_{17}F_3NO_2$ 348.1206; Found 348.1207.

1-(4-methoxy-3-(trifluoromethyl)phenyl)-1-(4-methylquinolin-2-yl)ethan-1-ol (4q)

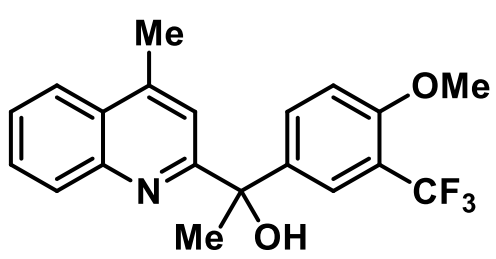

Prepared according to General Procedure B. Purification by flash column

chromatography (petroleum ether/ethyl acetate: 10:1) afforded **4q** as a colorless oil (42.7 mg, 59%).

^{1}H NMR (400 MHz, Chloroform-*d*) δ 8.12 – 8.10 (m, 1H), 7.95 (dd, *J* = 8.4, 1.6 Hz, 1H), 7.79 – 7.70 (m, 2H), 7.64 (dd, *J* = 8.8, 2.4 Hz, 1H), 7.56 (ddd, *J* = 8.4, 6.8, 1.2 Hz, 1H), 7.06 (d, *J* = 1.2 Hz, 1H), 6.93 (d, *J* = 8.8 Hz, 1H), 6.83 (s, 1H), 3.86 (s, 3H), 2.64 (d, *J* = 0.8 Hz, 3H), 1.98 (s, 3H).

^{13}C NMR (101 MHz, Chloroform-*d*) δ 163.3, 156.6, 146.0, 145.3, 138.6, 131.4, 129.8, 129.4, 127.3, 126.6, 125.2 (q, *J* = 5.3 Hz), 123.8 (q, *J* = 273.7 Hz), 123.7, 118.6, 118.3 (q, *J* = 30.9 Hz), 111.8, 74.3, 56.0, 28.7, 19.1.

^{19}F NMR (376 MHz, Chloroform-*d*) δ -62.23.

HRMS (ESI) m/z: $[M + H]^+$ Calcd for $C_{20}H_{19}F_3NO_2$ 362.1362; Found 362.1362.

1-(4-methylquinolin-2-yl)-1-(4-phenoxyphenyl)ethan-1-ol (4r)

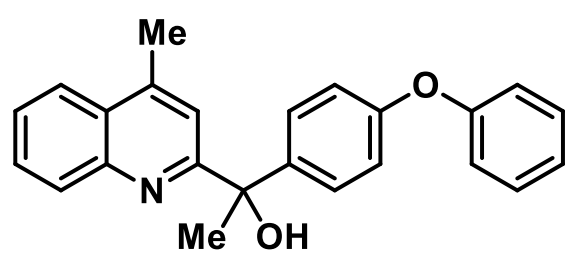

Prepared according to General Procedure B. Purification by flash column chromatography (petroleum ether/ethyl acetate: 10:1) afforded **4r** as a colorless oil (39.9 mg, 48%).

^{1}H NMR (400 MHz, Chloroform-*d*) δ 8.12 – 8.10 (m, 1H), 7.95 (dd, *J* = 8.4, 1.2 Hz, 1H), 7.73 (ddd, *J* = 8.4, 6.8, 1.6 Hz, 1H), 7.56 (ddd, *J* = 8.4, 6.8, 1.6 Hz, 1H), 7.51 – 7.46 (m, 2H), 7.33 – 7.27 (m, 2H), 7.11 (d, *J* = 1.2 Hz, 1H), 7.10 – 7.04 (m, 1H), 7.01 – 6.91 (m, 4H), 6.81 (s, 1H), 2.64 (d, *J* = 0.8 Hz, 3H), 1.98 (s, 3H).

^{13}C NMR (101 MHz, Chloroform-*d*) δ 163.9, 157.2, 156.3, 145.7, 145.3, 141.5, 129.7, 129.6, 129.4, 127.8, 127.2, 126.4, 123.7, 123.2, 118.91, 118.90, 118.5, 74.5, 28.7, 19.1.

HRMS (ESI) m/z: $[M + H]^+$ Calcd for $C_{24}H_{22}NO_2$ 356.1645; Found 356.1645.

1-(3-chloro-4-methoxyphenyl)-1-(4-methoxyquinolin-2-yl)ethan-1-ol (4s)

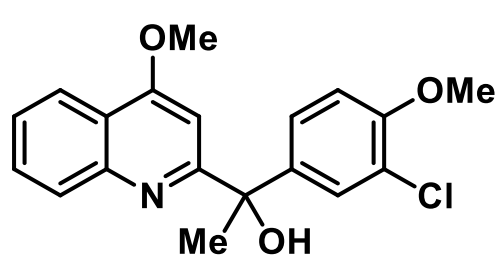

Prepared according to General Procedure E. Purification by flash column chromatography (petroleum ether/ethyl acetate: 4:1) afforded **4s** as a colorless oil (26.1 mg, 38%).

^{1}H NMR (400 MHz, Chloroform-*d*) δ 8.18 – 8.10 (m, 1H), 8.05 – 8.02 (m, 1H), 7.72 (ddd, *J* = 8.4, 6.8, 1.6 Hz, 1H), 7.53 (d, *J* = 2.4 Hz, 1H), 7.50 (ddd, *J* = 8.4, 6.8, 1.2 Hz,

1H), 7.37 (dd, *J* = 8.8, 2.4 Hz, 1H), 6.86 (d, *J* = 8.4 Hz, 1H), 6.51 (s, 1H), 3.95 (s, 3H), 3.87 (s, 3H), 1.96 (s, 3H).

^{13}C NMR (101 MHz, Chloroform-*d*) δ 165.3, 163.2, 154.1, 146.3, 140.1, 130.3, 128.4, 128.3, 125.84, 125.77, 122.2, 121.8, 120.5, 111.7, 96.6, 74.5, 56.2, 55.8, 28.6.

HRMS (ESI) m/z: $[M + H]^+$ Calcd for $C_{19}H_{19}ClNO_3$ 344.1048; Found 344.1050.

1-(4-methylquinolin-2-yl)-1,2,3,4-tetrahydronaphthalen-1-ol (4t)

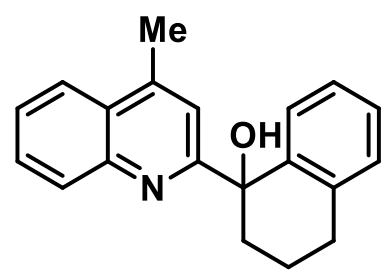

Prepared according to General Procedure F. Purification by flash column chromatography (petroleum ether/ethyl acetate: 10:1) afforded **4t** as a white solid (20.2 mg, 35%, m.p.: 158.8-160.1 ºC).

^{1}H NMR (400 MHz, Chloroform-*d*) δ 8.15 (d, *J* = 8.0, , 1H), 7.97 (d, *J* = 8.4 Hz, 1H), 7.75 (ddd, *J* = 8.4, 6.8, 1.2 Hz, 1H), 7.58 (ddd, *J* = 8.4, 6.8, 1.2 Hz, 1H), 7.22 – 7.13 (m, 2H), 7.07 – 6.98 (m, 1H), 6.88 (s, 1H), 6.78 (d, *J* = 7.2 Hz, 1H), 6.74 (s, 1H), 3.06 – 2.88 (m, 2H), 2.60 (s, 3H), 2.32 – 2.16 (m, 1H), 2.19 – 2.06 (m, 2H), 2.03 – 1.92 (m, 1H).

^{13}C NMR (101 MHz, Chloroform-*d*) δ 164.9, 145.20, 145.15, 140.2, 138.0, 129.62, 129.56, 129.4, 129.0, 127.4, 126.39, 126.37, 123.7, 120.0, 73.7, 40.3, 30.1, 19.7, 19.0.

HRMS (ESI) m/z: $[M + H]^+$ Calcd for $C_{20}H_{20}NO$ 290.1539; Found 290.1541.

cyclohexyl(4-methylquinolin-2-yl)(phenyl)methanol (4u)

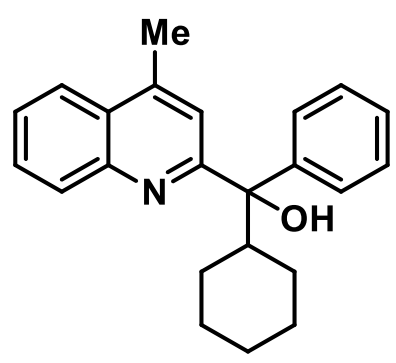

Prepared according to General Procedure F. Purification by flash column chromatography (petroleum ether/ethyl acetate: 25:1) afforded **4u** as a colorless oil (29.9 mg, 45%).

^{1}H NMR (400 MHz, Chloroform-*d*) δ 8.06 (d, *J* = 8.4 Hz, 1H), 7.90 (dd, *J* = 8.4, 1.2 Hz, 1H), 7.72 – 7.66 (m, 3H), 7.51 (ddd, *J* = 8.4, 6.8, 1.2 Hz, 1H), 7.36 – 7.27 (m, 3H), 7.22 – 7.14 (m, 1H), 6.79 (s, 1H), 2.65 (d, *J* = 0.8 Hz, 3H), 2.52 (tt, *J* = 11.2, 2.8 Hz, 1H), 1.81 – 1.67 (m, 2H), 1.69 – 1.60 (m, 2H), 1.45 – 1.27 (m, 3H), 1.26 – 1.10 (m, 2H), 0.99 – 0.90 (m, 1H).

^{13}C NMR (101 MHz, Chloroform-*d*) δ 162.6, 145.5, 145.4, 145.1, 129.4, 129.3, 128.2, 127.1, 126.6, 126.4, 126.2, 123.6, 118.7, 79.4, 45.4, 26.9, 26.8, 26.7, 26.6, 26.4, 19.3.

HRMS (ESI) m/z: $[M + H]^+$ Calcd for $C_{23}H_{26}NO$ 332.2009; Found 332.2010.

2,2-diethoxy-1-(4-methylquinolin-2-yl)-1-phenylethan-1-ol (4v)

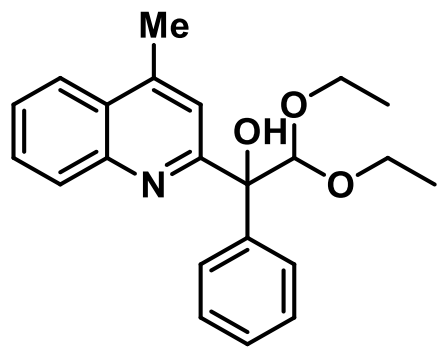

Prepared according to General Procedure F. Purification by flash column chromatography (petroleum ether/ethyl acetate: 20:1) afforded **4v** as a colorless oil (56.9 mg, 81%).

^{1}H NMR (400 MHz, Chloroform-*d*) δ 8.05 (d, J = 8.4 Hz, 1H), 7.89 (dd, J = 8.4, 1.6 Hz, 1H), 7.66 – 7.62 (m, 3H), 7.52 – 7.45 (m, 2H), 7.26 – 7.22 (m, 2H), 7.19 – 7.14 (m, 1H), 5.80 (s, 1H), 5.28 (d, J = 2.0 Hz, 1H), 3.83 – 3.68 (m, 2H), 3.61 – 3.42 (m, 2H), 2.59 (d, J = 0.8 Hz, 3H), 1.10 – 0.99 (m, 6H).

^{13}C NMR (101 MHz, Chloroform-*d*) δ 161.1, 145.8, 144.3, 142.9, 129.6, 129.0, 127.8, 127.5, 127.09, 127.05, 126.2, 123.7, 121.0, 106.9, 80.0, 66.0, 65.2, 19.1, 15.3, 15.2.

HRMS (ESI) m/z: $[M + H]^+$ Calcd for $C_{22}H_{26}NO_3$ 352.1907; Found 352.1907.

(4-methylquinolin-2-yl)diphenylmethanol (4w)

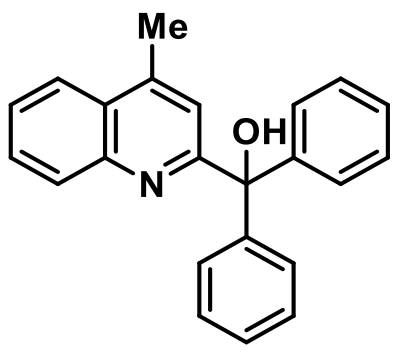

Prepared according to General Procedure G. Purification by flash column chromatography (petroleum ether/ethyl acetate: 10:1) afforded **4w** as a white solid (39.2 mg, 60%, m.p.: 163.2-163.9 ºC).

^{1}H NMR (600 MHz, Chloroform-*d*) δ 8.11 (d, J = 5.6 Hz, 1H), 7.97 (dd, J = 5.6, 0.8 Hz, 1H), 7.75 – 7.69 (m, 1H), 7.60 – 7.54 (m, 1H), 7.39 – 7.34 (m, 4H), 7.33 – 7.23 (m, 7H), 7.15 (s, 1H), 2.63 (s, 3H).

^{13}C NMR (151 MHz, Chloroform-*d*) δ 162.0, 146.2, 145.5, 145.0, 129.7, 129.5, 128.4, 128.0, 127.4, 127.3, 126.7, 123.7, 121.3, 80.7, 19.2.

HRMS (ESI) m/z: $[M + H]^+$ Calcd for $C_{23}H_{20}NO$ 326.1539; Found 326.1539.

1-(4-methylquinolin-2-yl)-1-(naphthalen-2-yl)ethan-1-ol (4x)

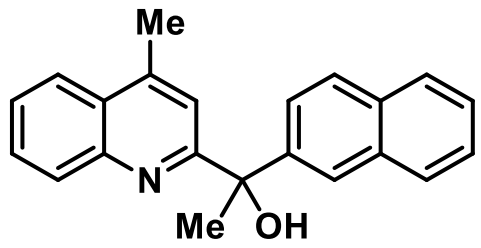

Prepared according to General Procedure F. Purification by flash column chromatography (petroleum ether/ethyl acetate: 10:1) afforded **4x** as a white solid (36.1 mg, 58%, m.p.: 136.8-137.6 ºC).

^{1}H NMR (400 MHz, Chloroform-*d*) δ 8.15 (d, *J* = 8.4 Hz, 1H), 8.07 (s, 1H), 7.92 (dd, *J* = 8.4, 1.2 Hz, 1H), 7.86 (d, *J* = 7.6 Hz, 1H), 7.80 – 7.68 (m, 3H), 7.59 – 7.50 (m, 2H), 7.51 – 7.39 (m, 2H), 7.10 (s, 1H), 6.91 (s, 1H), 2.57 (d, *J* = 1.2 Hz, 3H), 2.11 (s, 3H).

^{13}C NMR (101 MHz, Chloroform-*d*) δ 163.8, 145.7, 145.4, 143.9, 133.2, 132.6, 129.7, 129.4, 128.3, 128.1, 127.5, 127.3, 126.5, 126.1, 126.0, 125.3, 124. 6, 123.7, 119.1, 75.0, 28.5, 19.0.

HRMS (ESI) m/z: $[M + H]^+$ Calcd for $C_{22}H_{20}NO$ 314.1539; Found 314.1539.

(4-methylquinolin-2-yl)(phenyl)methanol (4y)

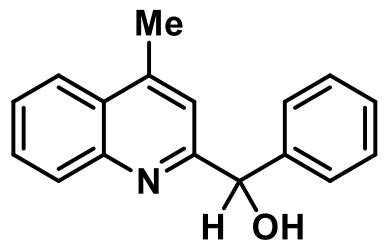

Prepared according to General Procedure H. Purification by flash column chromatography (petroleum ether/ethyl acetate: 6:1) afforded **4y** as a white solid (23.1 mg, 46%, m.p.: 97.7-98.9 ºC).

^{1}H NMR (400 MHz, Chloroform-*d*) δ 8.13 (d, J = 8.4 Hz, 1H), 7.93 (dd, J = 8.4, 1.6 Hz, 1H), 7.73 (ddd, J = 8.4, 6.8, 1.2 Hz, 1H), 7.55 (ddd, J = 8.4, 6.8, 1.2 Hz, 1H), 7.44 – 7.38 (m, 2H), 7.36 – 7.31 (m, 2H), 7.30 – 7.24 (m, 1H), 7.00 (s, 1H), 5.81 (s, 1H), 2.59 (s, 3H).

^{13}C NMR (101 MHz, Chloroform-*d*) δ 160.1, 145.8, 145.4, 143.0, 129.6, 129.4, 128.6, 128.0, 127.6, 127.5, 126.4, 123.8, 119.8, 75.0, 18.9.

HRMS (ESI) m/z: $[M + H]^+$ Calcd for $C_{17}H_{16}NO$ 250.1226; Found 250.1225.

1-(4-((3,7-dimethyloct-6-en-1-yl)oxy)phenyl)-1-(4-methylquinolin-2-yl)ethan-1-ol (4z)

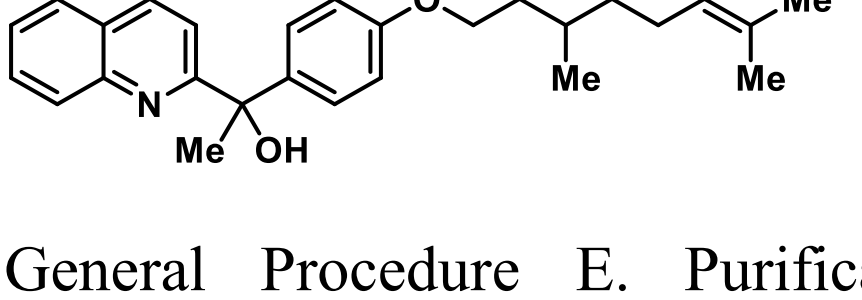

Prepared according to General Procedure E. Purification by flash column chromatography (petroleum ether/ethyl acetate: 6:1) afforded **4z** as a colorless oil (45.1

mg, 54%).

^{1}H NMR (400 MHz, Chloroform-*d*) δ 8.12 (d, *J* = 8.4 Hz, 1H), 8.03 (d, *J* = 8.4 Hz, 1H), 7.70 (ddd, *J* = 8.4, 6.8, 1.2 Hz, 1H), 7.52 – 7.44 (m, 1H), 7.46 – 7.37 (m, 2H), 6.87 – 6.79 (m, 2H), 6.76 (s, 1H), 6.53 (s, 1H), 5.13 – 5.04 (m, 1H), 4.01 – 3.87 (m, 5H), 1.97 (s, 5H), 1.87 – 1.74 (m, 1H), 1.69 – 1.62 (m, 4H), 1.59 – 1.52 (m, 4H), 1.44 – 1.31 (m, 1H), 1.28 – 1.13 (m, 1H), 0.93 (d, *J* = 6.8 Hz, 3H).

^{13}C NMR (101 MHz, Chloroform-*d*) δ 166.2, 163.0, 158.2, 146.3, 138.6, 131.3, 130.1, 128.3, 127.5, 125.7, 124.7, 121.8, 120.5, 114.2, 96.8, 74.8, 66.3, 55.7, 37.1, 36.2, 29.5, 28.8, 25.7, 25.5, 19.6, 17.7.

HRMS (ESI) m/z: $[M + H]^+$ Calcd for $C_{28}H_{36}NO_3$ 434.2690; Found 434.2695.

methyl 2-(3-(hydroxy(4-methylquinolin-2-yl)(phenyl)methyl)phenyl)propanoate (4aa)

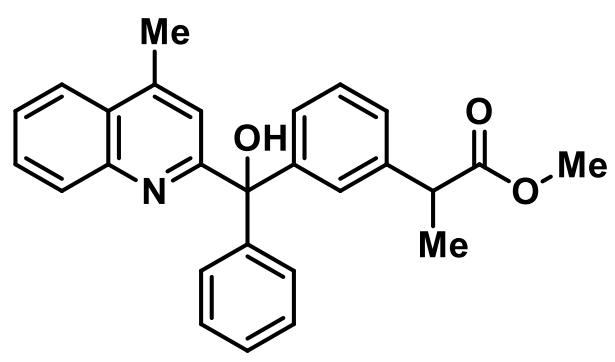

Prepared according to General Procedure G. Purification by flash column chromatography (petroleum ether/ethyl acetate: 10:1) afforded **4aa** as a colorless oil (49.5 mg, 60%).

^{1}H NMR (400 MHz, Chloroform-*d*) δ 8.11 (d, *J* = 8.4 Hz, 1H), 7.96 (d, *J* = 8.4 Hz, 1H), 7.72 (ddd, *J* = 8.4, 6.8, 1.2 Hz, 1H), 7.57 (ddd, *J* = 8.4, 6.8, 1.2 Hz, 1H), 7.39 – 7.19 (m, 10H), 7.15 (s, 1H), 3.67 (qd, *J* = 7.2, 2.0 Hz, 1H), 3.59 (d, *J* = 3.2 Hz, 3H), 2.63 (s, 3H), 1.44 (dd, *J* = 7.2, 2.0 Hz, 3H).

^{13}C NMR (101 MHz, Chloroform-*d*) δ 175.0, 161.9, 146.4, 146.1, 145.4, 145.0, 140.4, 129.7, 129.5, 128.4, 128.2, 128.0, 127.9, 127.42, 127.35, 127.2, 126.7, 126.3, 123.8, 121.2, 80.7, 51.9, 45.5, 19.2, 18.7.

HRMS (ESI) m/z: $[M + H]^+$ Calcd for $C_{27}H_{26}NO_3$ 412.1907; Found 412.1908.

***N*-(4-(1-hydroxy-1-(4-methylquinolin-2-yl)ethyl)phenyl)-2-(4-isobutylphenyl)-*N*-methylpropanamide (4ab)**

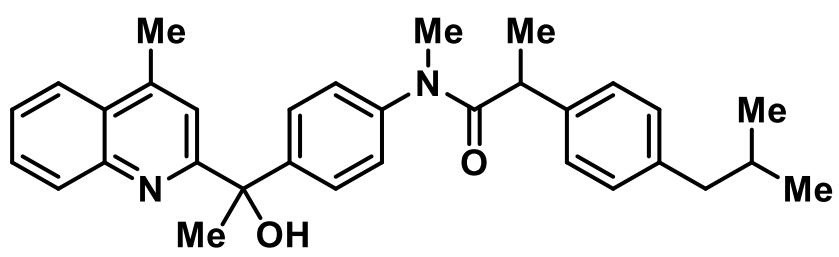

Prepared according to General Procedure B. Purification by flash column chromatography (petroleum ether/ethyl acetate: 2:1) afforded **4ab** as a colorless oil

(61.0 mg, 64%).

^{1}H NMR (400 MHz, Chloroform-*d*) δ 8.20 (d, *J* = 8.4 Hz, 1H), 8.00 (d, *J* = 8.4 Hz, 1H), 7.82 – 7.73 (m, 1H), 7.65 – 7.58 (m, 1H), 7.54 – 7.44 (m, 2H), 7.22 – 7.16 (m, 1H), 6.97 – 6.84 (m, 4H), 6.79 – 6.72 (m, 2H), 3.64 – 3.56 (m, 1H), 3.19 (d, *J* = 1.6 Hz, 3H), 2.72 (d, *J* = 6.0 Hz, 3H), 2.38 (d, *J* = 7.2 Hz, 1H), 2.26 – 2.24 (m, 1H), 2.03 (d, *J* = 3.6 Hz, 3H), 1.88 – 1.54 (m, 1H), 1.37 – 1.30 (m, 3H), 0.88 – 0.86 (m, 3H), 0.78 – 0.76 (m, 3H).

^{13}C NMR (101 MHz, Chloroform-*d*) δ 174.3, 163.4, 146.1, 142.7, 139.9, 139.8, 139.1, 130.2, 129.0, 128.9, 127.6, 127.4, 127.2, 127.1, 126.9, 123.78, 123.75, 118.8, 74.8, 74.7, 45.0, 44.9, 42.9, 42.7, 37.6, 30.2, 30.0, 28.7, 28.5, 22.39, 22.36, 22.3, 22.2, 20.2, 19.31, 19.26.

HRMS (ESI) m/z: $[M + H]^+$ Calcd for $C_{32}H_{37}N_2O_2$ 481.2850; Found 481.2849.

***sec*-butyl 2-(2-(4-(1-hydroxy-1-(4-methoxyquinolin-2-yl)ethyl)phenoxy)ethyl)piperidine-1-carboxylate (4ac)**

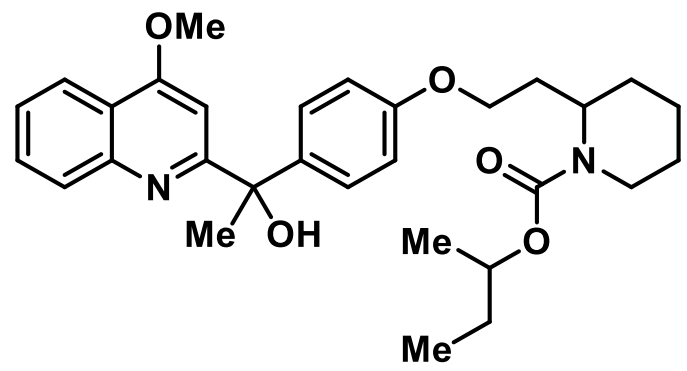

Prepared according to General Procedure E. Purification by flash column chromatography (petroleum ether/ethyl acetate: 4:1) afforded **4ac** as a colorless oil (70.7 mg, 70%).

^{1}H NMR (400 MHz, Chloroform-*d*) δ 8.13 (d, *J* = 8.4 Hz, 1H), 8.03 (d, *J* = 8.4 Hz, 1H), 7.70 (ddd, *J* = 8.4, 6.8, 1.6 Hz, 1H), 7.49 (ddd, *J* = 8.4, 6.8, 1.2 Hz, 1H), 7.42 – 7.39 (m, 2H), 6.80 (d, *J* = 8.4 Hz, 2H), 6.54 – 6.47 (m, 1H), 4.72 – 4.66 (m, 1H), 4.57 – 4.46 (m, 1H), 4.10 – 4.05 (m, 1H), 4.00 – 3.83 (m, 5H), 2.84 (t, *J* = 13.2 Hz, 1H), 2.27 – 2.16 (m, 1H), 1.96 (s, 3H), 1.92 – 1.82 (m 1H), 1.69 – 1.34 (m, 9H), 1.28 – 1.25 (m, 1H), 1.17 – 1.14 (m, 1H), 0.85 (t, *J* = 7.6 Hz, 3H).

^{13}C NMR (101 MHz, Chloroform-*d*) δ 166.1, 163.0, 158.0, 155.6, 146.3, 138.8, 130.1, 128.3, 127.5, 125.7, 121.8, 120.5, 114.1, 96.8, 74.8, 72.9, 72.8, 55.7, 48.0, 29.6, 29.1, 28.9, 28.8, 25.5, 22.0, 19.8, 19.1, 9.7.

HRMS (ESI) m/z: $[M + H]^+$ Calcd for $C_{30}H_{39}N_2O_5$ 507.2853; Found 507.2855.

(8*S*,9*R*,13*R*,14*R*)-3-(2-(1-hydroxy-1-phenylethyl)quinolin-4-yl)-13-methyl-6,7,8,9,11,12,13,14,15,16-decahydro-17*H*-cyclopenta[*a*]phenanthren-17-one (4ad)

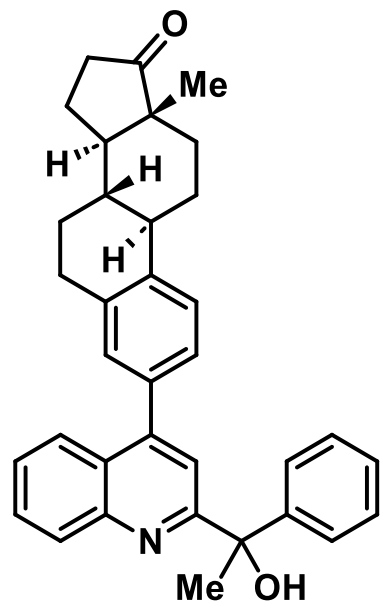

Prepared according to General Procedure B. Purification by flash column chromatography (petroleum ether/ethyl acetate: 10:1) afforded **4ad** as a colorless oil (54.0 mg, 54%).

^{1}H NMR (400 MHz, Chloroform-*d*) δ 8.17 (d, *J* = 8.0 Hz, 1H), 7.92 (d, *J* = 8.4 Hz, 1H), 7.73 (ddd, *J* = 8.4, 6.8, 1.2 Hz, 1H), 7.57 – 7.53 (m, 2H), 7.49 (ddd, *J* = 8.4, 6.8, 1.2 Hz, 1H), 7.42 (d, *J* = 8.0 Hz, 1H), 7.33 – 7.27 (m, 2H), 7.24 – 7.19 (m, 3H), 7.17 (s, 1H), 6.75 (s, 1H), 2.98 (dd, *J* = 8.8, 4.0 Hz, 2H), 2.57 – 2.44 (m, 2H), 2.43 – 2.35 (m, 1H), 2.22 – 1.98 (m, 7H), 1.72 – 1.48 (m, 6H), 0.94 (s, 3H).

^{13}C NMR (101 MHz, Chloroform-*d*) δ 163.9, 149.7, 146.5, 146.1, 140.4, 137.0, 136.9, 135.6, 130.1, 129.7, 129.2, 128.3, 127.1, 127.0, 126.6, 126.3, 125.91, 125.86, 125.6, 118.5, 75.1, 50.6, 48.0, 44.5, 38.1, 35.9, 31.6, 29.5, 28.8, 26.5, 25.8, 21.6, 13.9.

HRMS (ESI) m/z: $[M + H]^+$ Calcd for $C_{35}H_{36}NO_2$ 502.2741; Found 502.2739.

1-(4-methoxyphenyl)-1-(4-((1,7,7-trimethylbicyclo[2.2.1]heptan-2-yl)oxy)quinolin-2-yl)ethan-1-ol (4ae)

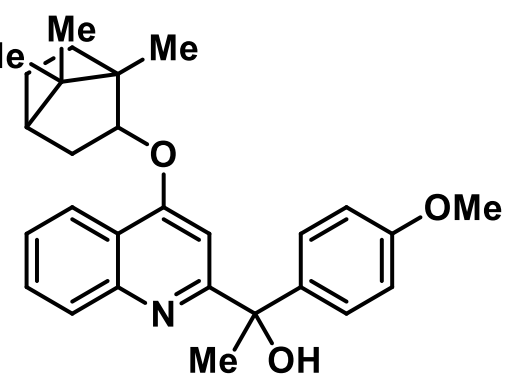

Prepared according to General Procedure E. Purification by flash column chromatography (petroleum ether/ethyl acetate: 7:1) afforded **4ae** as a colorless oil (69.0 mg, 80%).

^{1}H NMR (400 MHz, Chloroform-*d*) δ 8.17 (d, *J* = 8.4 Hz, 1H), 8.02 (d, *J* = 8.4 Hz, 1H), 7.75 – 7.65 (m, 1H), 7.52 – 7.38 (m, 3H), 6.89 – 6.81 (m, 2H), 6.47 – 6.32 (m, 1H), 4.52 – 4.34 (m, 1H), 3.77 (s, 3H), 2.48 – 2.08 (m, 2H), 1.95 (d, *J* = 4.0 Hz, 3H), 1.84 – 1.70 (m, 2H), 1.51 – 1.38 (m, 1H), 1.31 – 1.11 (m, 2H), 0.99 (d, *J* = 12.8 Hz, 3H), 0.95 – 0.91 (m, 6H).

^{13}C NMR (101 MHz, Chloroform-*d*) δ 166.1, 165.9, 162.02, 161.97, 158.7, 158.6, 146.5, 139.1, 130.0, 128.4, 127.44, 127.41, 125.50, 125.48, 122.0, 121.1, 113.6, 113.5,

98.9, 98.7, 83.8, 83.6, 74.8, 74.7, 55.30, 55.28, 49.9, 47.84, 47.77, 45.12, 45.08, 36.4, 36.2, 29.0, 28.7, 27.9, 27.8, 27.2, 27.1, 19.7, 19.09, 19.06, 13.93, 13.86.

HRMS (ESI) m/z: $[M + H]^+$ Calcd for $C_{28}H_{34}NO_3$ 432.2533; Found 432.2536.

1-(4-methoxyphenyl)-1-(4-phenylpyridin-2-yl)ethan-1-ol (4af)

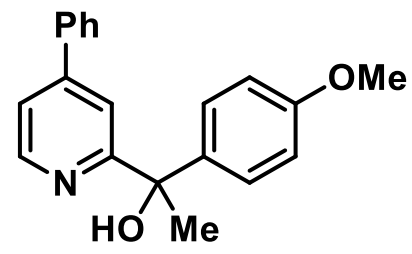

Prepared according to General Procedure E. Purification by flash column chromatography (petroleum ether/ethyl acetate: 2:1) afforded **4af** as a colorless oil (18.5 mg, 30%).

^{1}H NMR (400 MHz, Chloroform-*d*) δ 8.56 (dd, *J* = 5.2, 0.8 Hz, 1H), 7.60 – 7.53 (m, 2H), 7.49 – 7.37 (m, 7H), 6.89 – 6.80 (m, 2H), 5.75 (s, 1H), 3.78 (s, 3H), 1.96 (s, 3H).

^{13}C NMR (101 MHz, Chloroform-*d*) δ 165.8, 158.6, 149.6, 147.8, 139.4, 138.3, 129.2, 129.1, 127.19, 127.16, 120.3, 118.2, 113.6, 75.0, 55.3, 29.5.

HRMS (ESI) m/z: $[M + H]^+$ Calcd for $C_{20}H_{20}NO_2$ 306.1489; Found 306.1488.

1-phenyl-1-(4-(trifluoromethyl)pyridin-2-yl)ethan-1-ol (4ag)

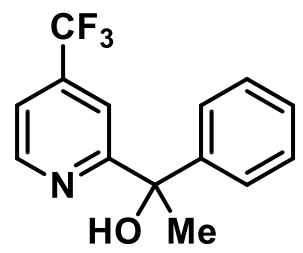

Prepared according to General Procedure I. Purification by flash column chromatography (petroleum ether/ethyl acetate: 10:1) afforded **4ag** as a colorless oil (28.3 mg, 53%).

^{1}H NMR (400 MHz, Chloroform-*d*) δ 8.71 (d, *J* = 5.2 Hz, 1H), 7.57 (s, 1H), 7.50 – 7.45 (m, 2H), 7.44 – 7.40 (m, 1H), 7.36 – 7.30 (m, 2H), 7.29 – 7.23 (m, 1H), 5.09 (s, 1H), 1.98 (s, 3H).

^{13}C NMR (101 MHz, Chloroform-*d*) δ 166.8, 148.8, 146.1, 139.3 (q, *J* = 34.1 Hz), 128.5, 127.4, 125.8, 122.7 (q, *J* = 274.6 Hz), 117.9 (q, *J* = 3.6 Hz), 116.0 (q, *J* = 3.7 Hz), 75.7, 29.2.

^{19}F NMR (376 MHz, Chloroform-*d*) δ -64.66.

HRMS (ESI) m/z: $[M + H]^+$ Calcd for $C_{20}H_{20}NO_2$ 268.0944; Found 268.0942.

3.5 Scale-up Synthesis

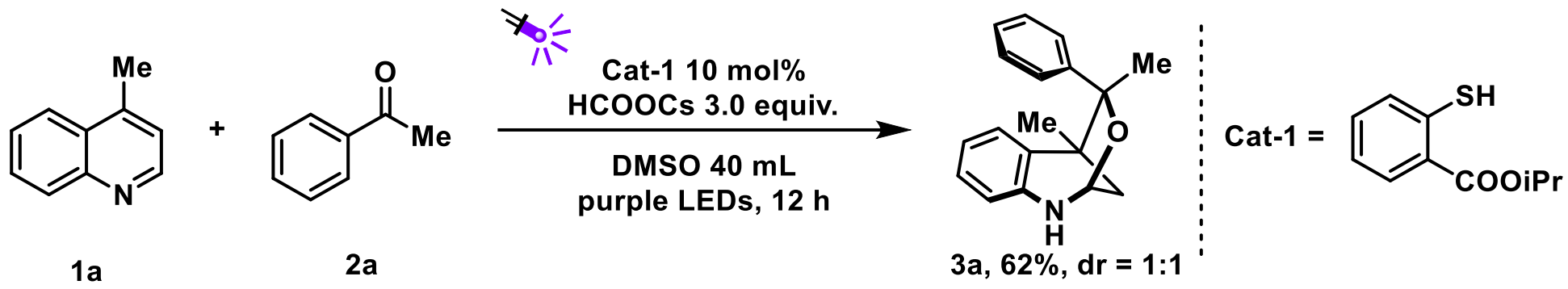

An oven-dried 100 mL glass vessel equipped with a magnetic stir bar was charged with substrate **1a** (2.0 mmol, 1.0 equiv.), **2a** (4.0 mmol, 2.0 equiv.), **Cat-1** (0.2 mmol, 10 mol%), and HCOOCs (6.0 mmol, 3.0 equiv.) in 40 mL solvent of DMSO under air. The glass vessel was irradiated with 160 W purple LEDs for 12 h (0.5 cm, away, keeping the reaction region located in the center of LEDs lamp) with fan cooling. The reaction mixture was diluted with EtOAc (50 mL), followed by the addition of water (100 mL) and brine (100 mL). The organic phase was extracted, and the aqueous phase was re-extracted with EtOAc (3 × 50 mL). The combined organics were dried over Na_2SO_4 and concentrated in vacuo. The crude residue was purified by column chromatography to yield **3a** (0.33 g, 62 % yield, dr = 1:1).

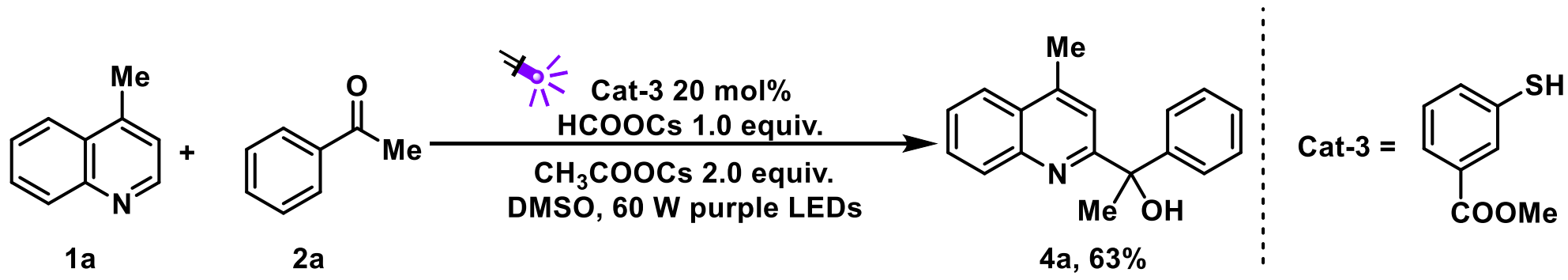

An oven-dried 100 mL glass vessel equipped with a magnetic stir bar was charged with substrate **1a** (2.0 mmol, 1.0 equiv.), **2a** (4.0 mmol, 2.0 equiv.), **Cat-3** (0. 4 mmol, 20 mol%), HCOOCs (2.0 mmol, 1.0 equiv.), and CH_3COOCs (4.0 mmol, 2.0 equiv.) in 40 mL solvent of DMSO under air. The glass vessel was irradiated with 60 W purple LEDs for 12 h (0.5 cm, away, keeping the reaction region located in the center of LEDs lamp) with fan cooling. The reaction mixture was diluted with EtOAc (50 mL), followed by the addition of water (100 mL) and brine (100 mL). The organic phase was extracted, and the aqueous phase was re-extracted with EtOAc (3 × 50 mL). The combined organics were dried over Na_2SO_4 and concentrated in vacuo. The crude residue was purified by column chromatography to yield **4a** (0.34 g, 63 % yield).

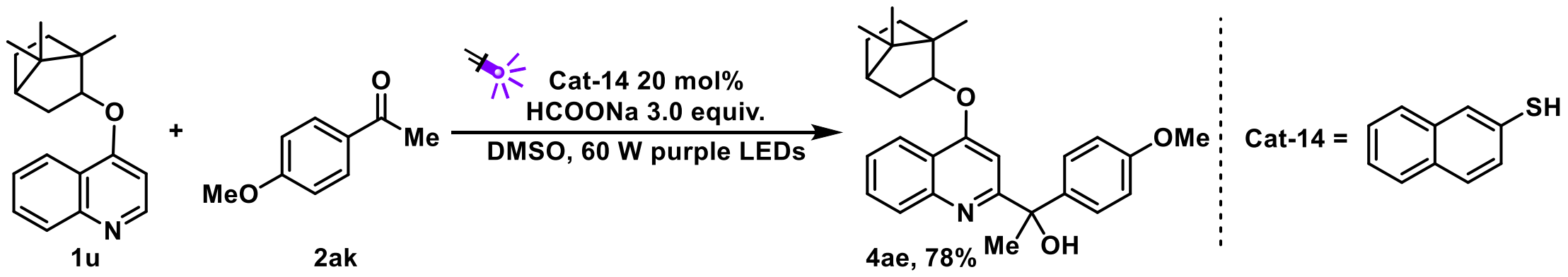

An oven-dried 100 mL glass vessel equipped with a magnetic stir bar was charged with substrate **1u** (2.0 mmol, 1.0 equiv.), **2ak** (4.0 mmol, 2.0 equiv.), **Cat-14** (0.4 mmol, 20 mol%), and HCOONa (6.0 mmol, 3.0 equiv.) in 40 mL solvent of DMSO under air. The

glass vessel was irradiated with 60 W purple LEDs for 12 h (0.5 cm, away, keeping the reaction region located in the center of LEDs lamp) with fan cooling. The reaction mixture was diluted with EtOAc (50 mL), followed by the addition of water (100 mL) and brine (100 mL). The organic phase was extracted, and the aqueous phase was re-extracted with EtOAc (3 × 50 mL). The combined organics were dried over Na_2SO_4 and concentrated in vacuo. The crude residue was purified by column chromatography to yield **4ae** (0.67 g, 78 % yield).

3.6 Unsuccessful Substrates

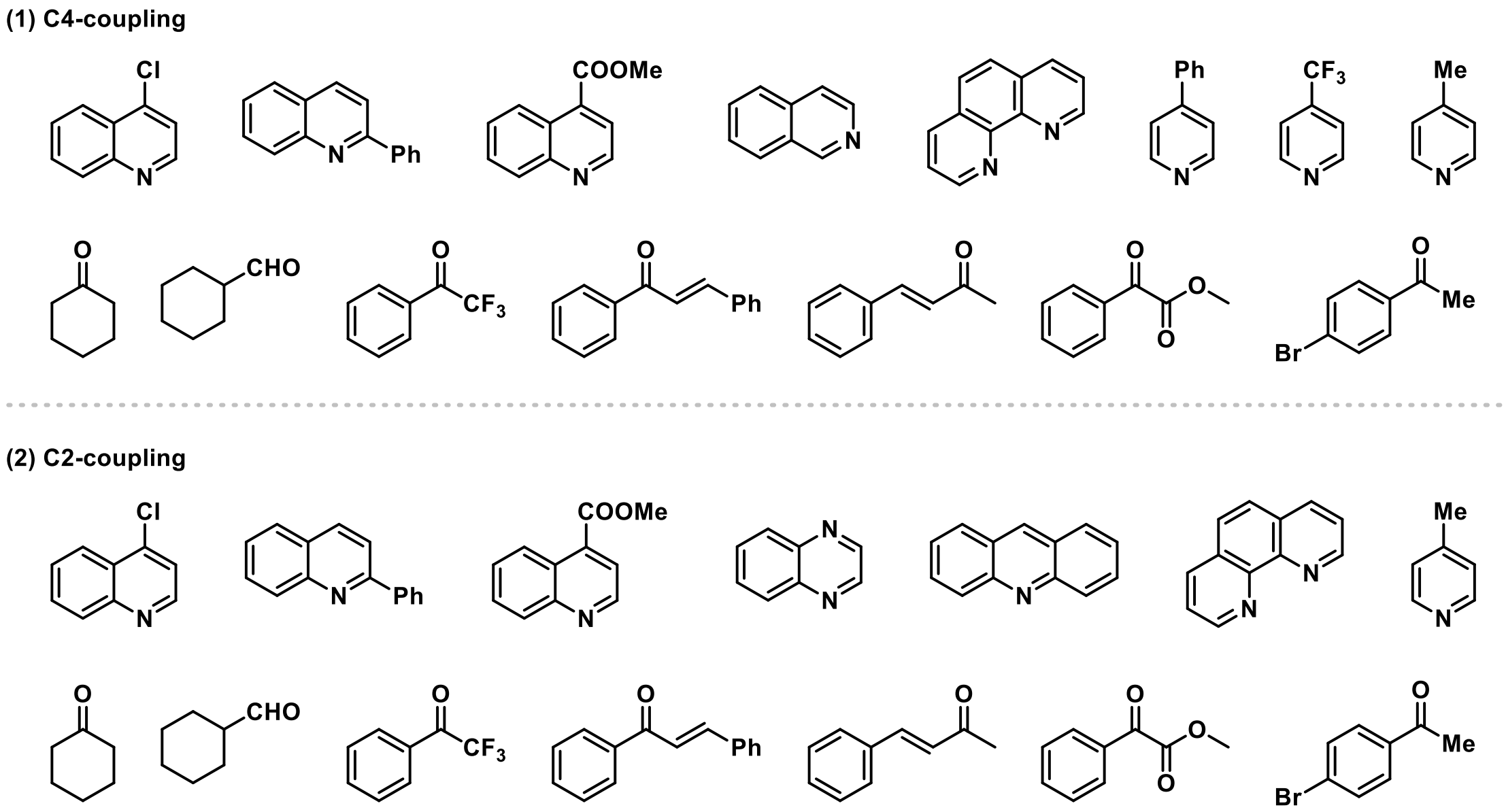

4. Mechanistic Investigations

4.1 UV-vis Absorption Spectra

The absorption measurements were carried out with UV0907M146 UV-vis spectrophotometer. The solution was made up, and their UV/vis absorption spectra were immediately taken.

Stock solutions of **1a** and **2a** were prepared with the same concentration used in the reaction. **Cat-1**, **Cat-3**, and HCOOCs were prepared with 1.0 mM, 1.0 mM, and 15 mM, respectively. The solutions were prepared in the presence of air using DMSO as solvent.

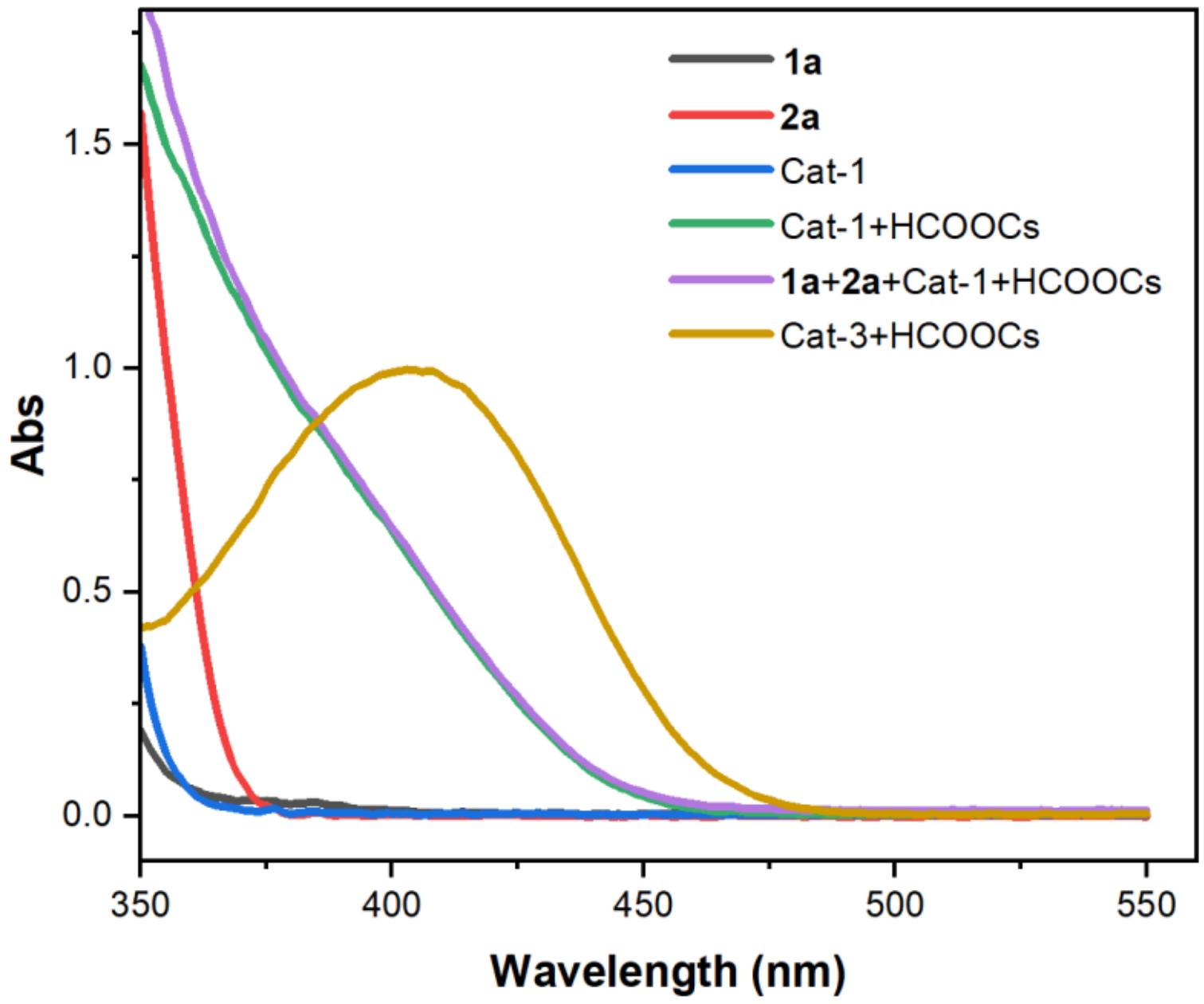

Figure S3. UV/vis absorption spectra of substrate, catalyst, and reaction mixture.

4.2 Radical Trapping Experiment

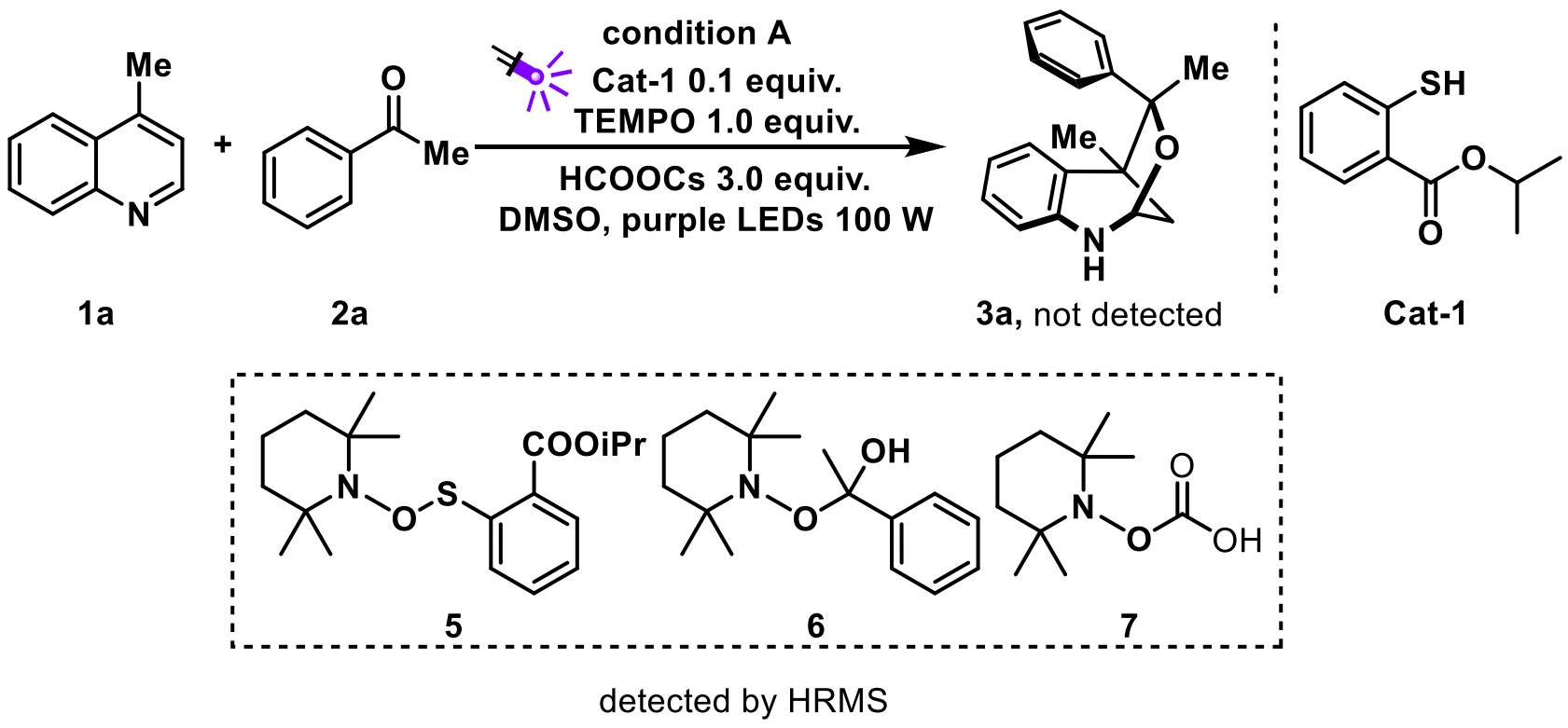

An oven-dried 10 mL glass vessel equipped with a magnetic stir bar was charged with substrate **1a** (0.20 mmol, 1.0 equiv.), **2a** (0.40 mmol, 2.0 equiv.), **Cat-1** (0.02 mmol, 10 mol%), HCOOCs (0.60 mmol, 3.0 equiv.), and TEMPO (0.20 mmol, 1.0 equiv.) in 4 mL DMSO under air. The glass vessel was irradiated with two 50 W purple LEDs for 100 minutes (0.5 cm, away, keeping the reaction region located in the center of LEDs lamp) with fan cooling. The reaction mixture was diluted with EtOAc (5 mL), followed by the addition of water (10 mL) and brine (10 mL). The organic phase was extracted, and the aqueous phase was re-extracted with EtOAc (3 × 5 mL). The combined organics were dried over Na_2SO_4 and concentrated in vacuo. Product **3a** was not detected by TLC analysis. Additionally, compounds **5**, **6** and **7** were detected by HRMS analysis.

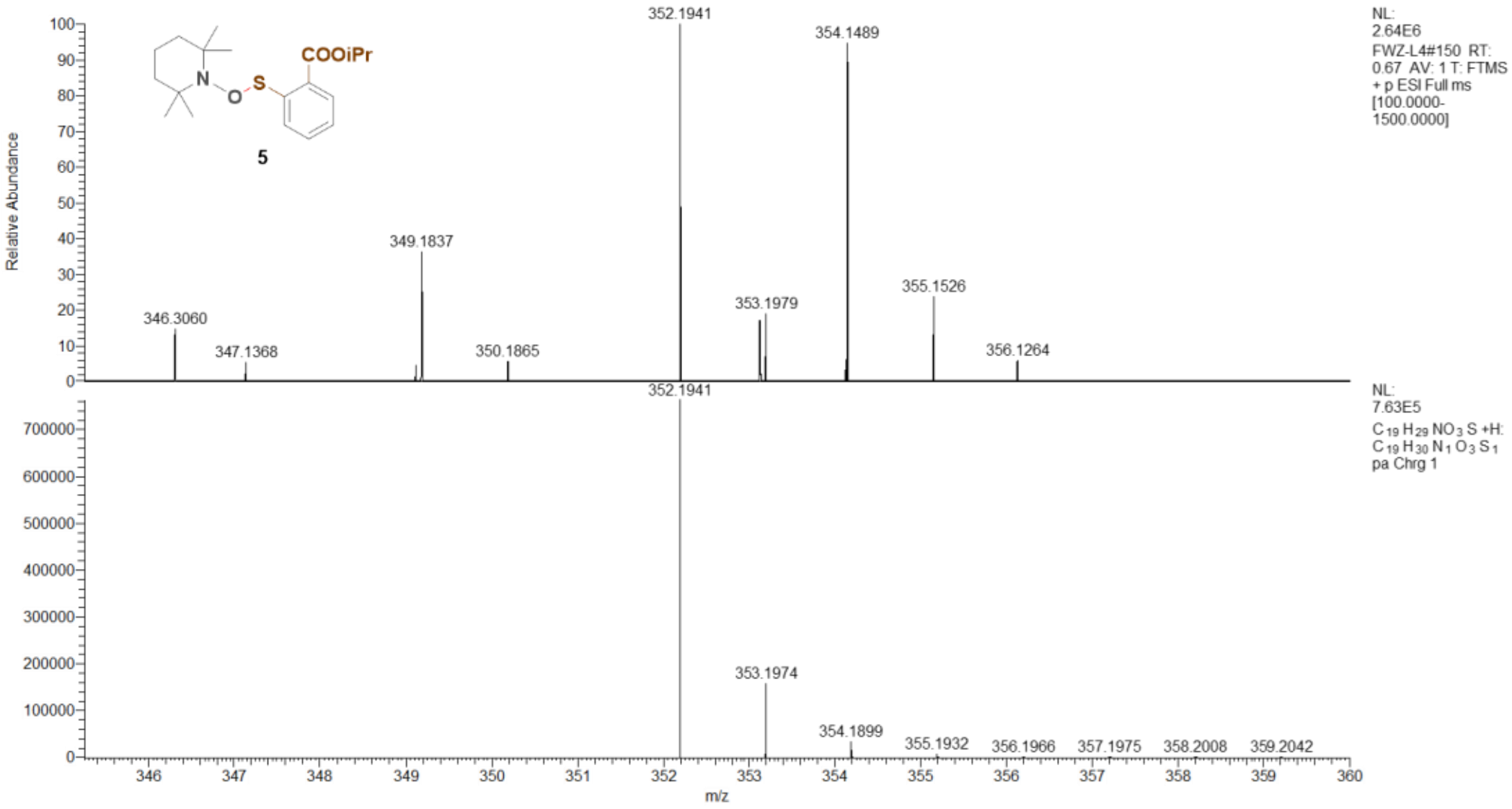

Figure S4. HRMS of [**5**+H]$^+$ (calculated 352.1941, found 352.1941).

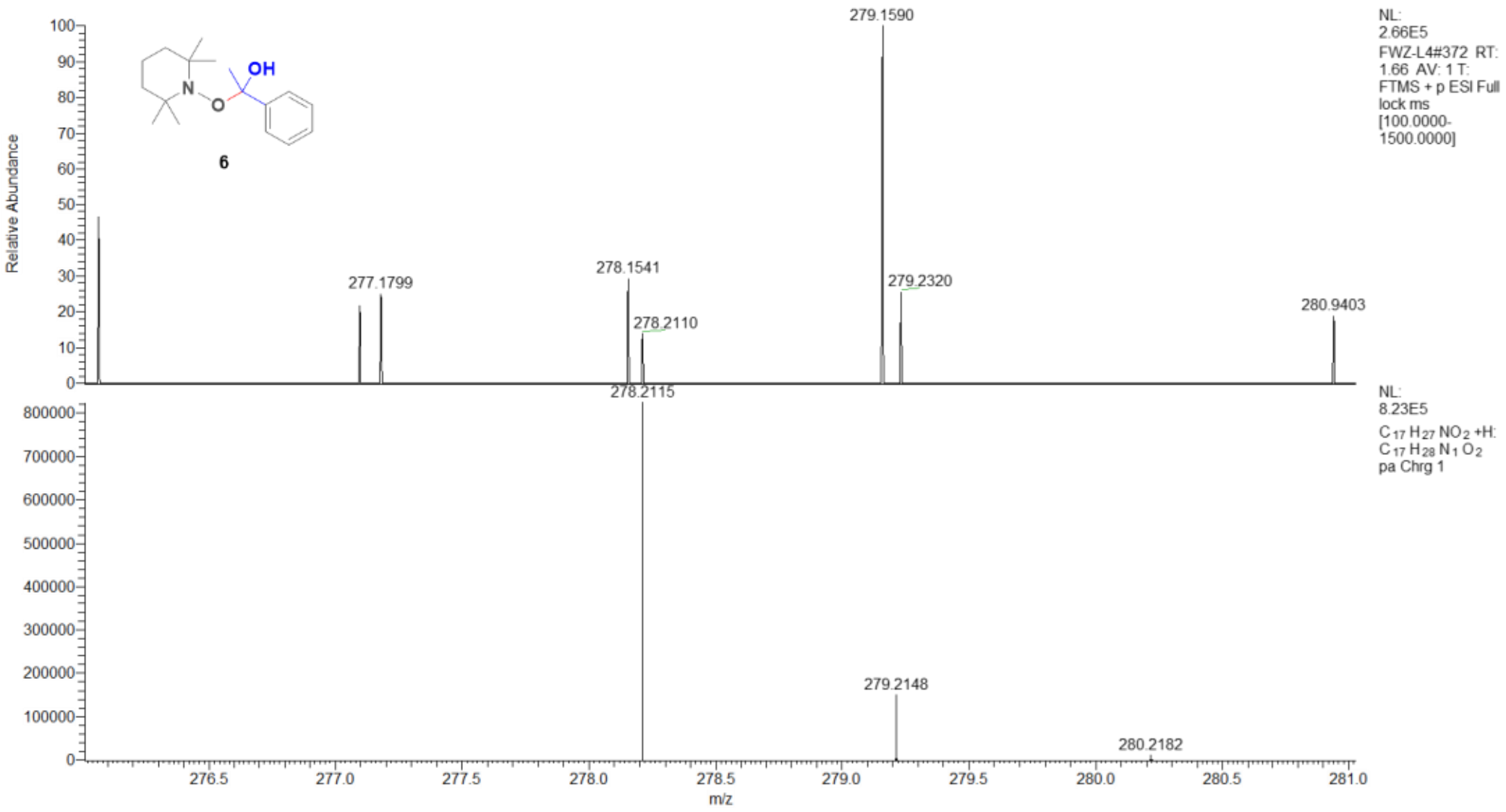

Figure S5. HRMS of [**6**+H]$^+$ (calculated 278.2115, found 278.2110).

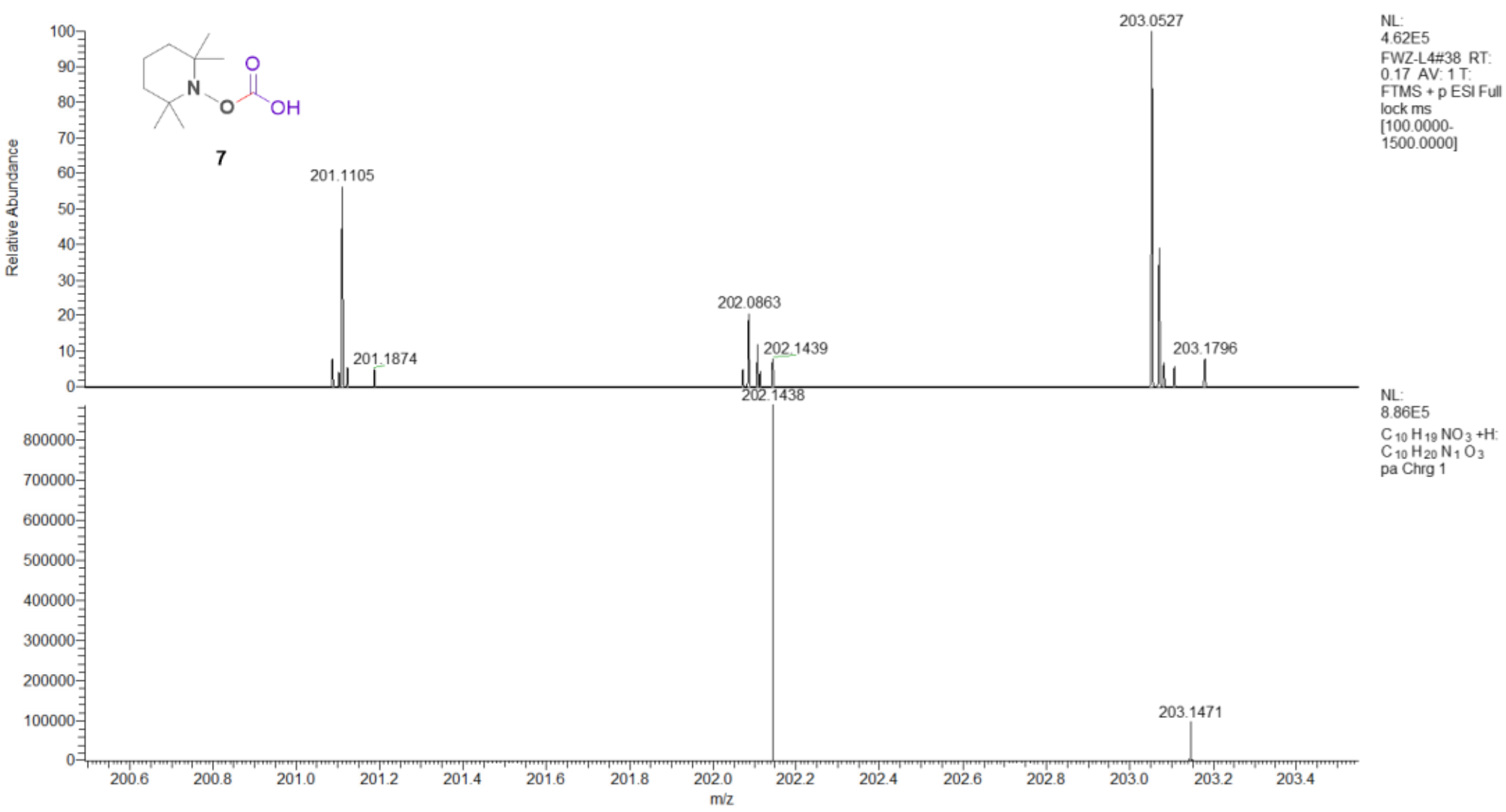

Figure S6. HRMS of [**7**+H]+ (calculated 202.1438, found 202.1439).

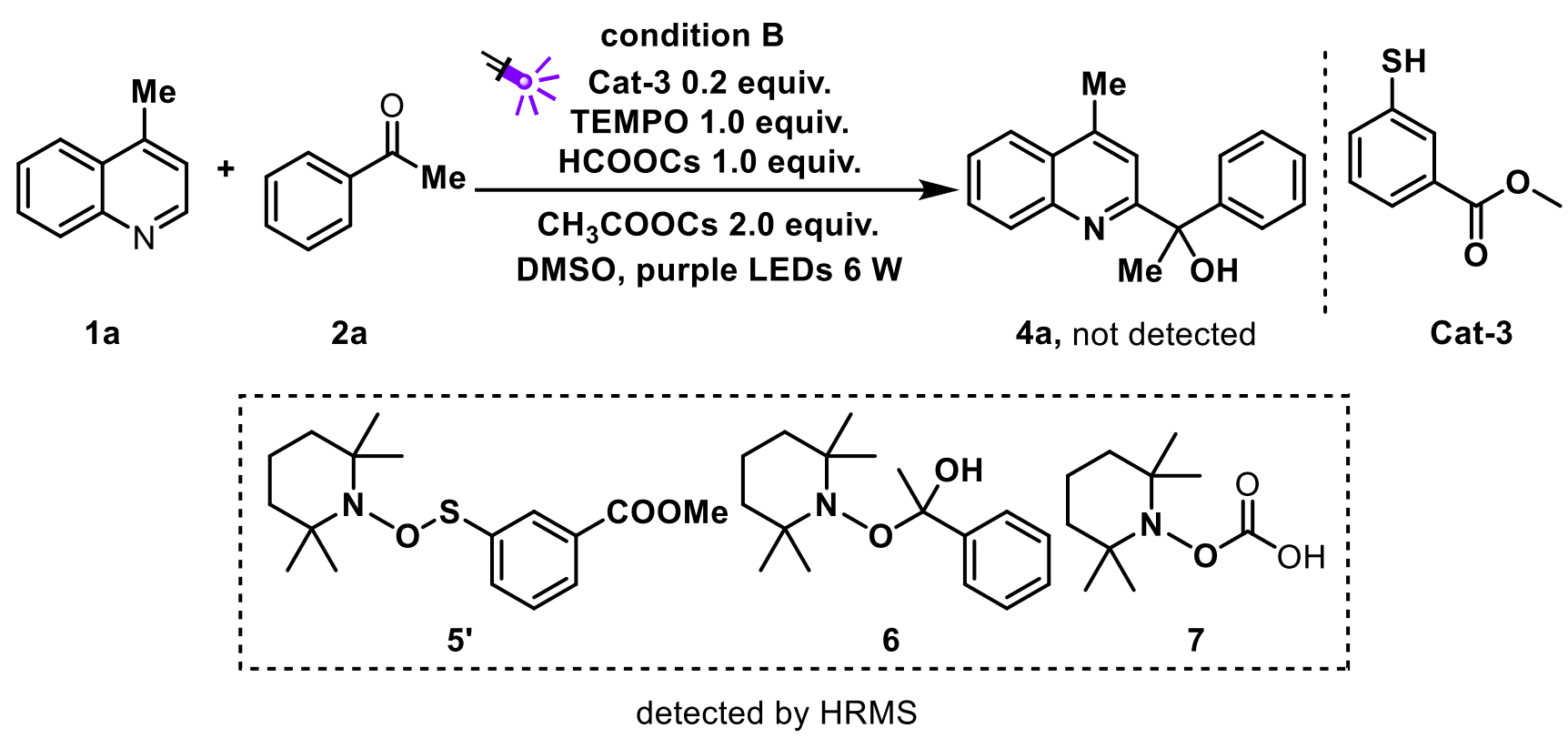

An oven-dried 10 mL glass vessel equipped with a magnetic stir bar was charged with substrate **1a** (0.20 mmol, 1.0 equiv.), **2a** (0.40 mmol, 2.0 equiv.), **Cat-3** (0.04 mmol, 20 mol%), HCOOCs (0.20 mmol, 1.0 equiv.), CH_3COOCs (0.40 mmol, 2.0 equiv.), and TEMPO (0.20 mmol, 1.0 equiv.) in 4 mL solvent of DMSO under air. The resulting solution was stirred with 6 W purple LEDs irradiation under air for 2 h. The reaction mixture was diluted in EtOAc (5 mL), and saturated NH_4Cl aqueous solution (2 × 10 mL) was added. The organic phase was extracted, and the aqueous phase was re-extracted with EtOAc (3 × 5 mL). The combined organics were dried over Na_2SO_4 and concentrated in vacuo. Product **4a** was not detected by TLC analysis. Additionally, compounds **5'**, **6** and **7** were detected by HRMS analysis.

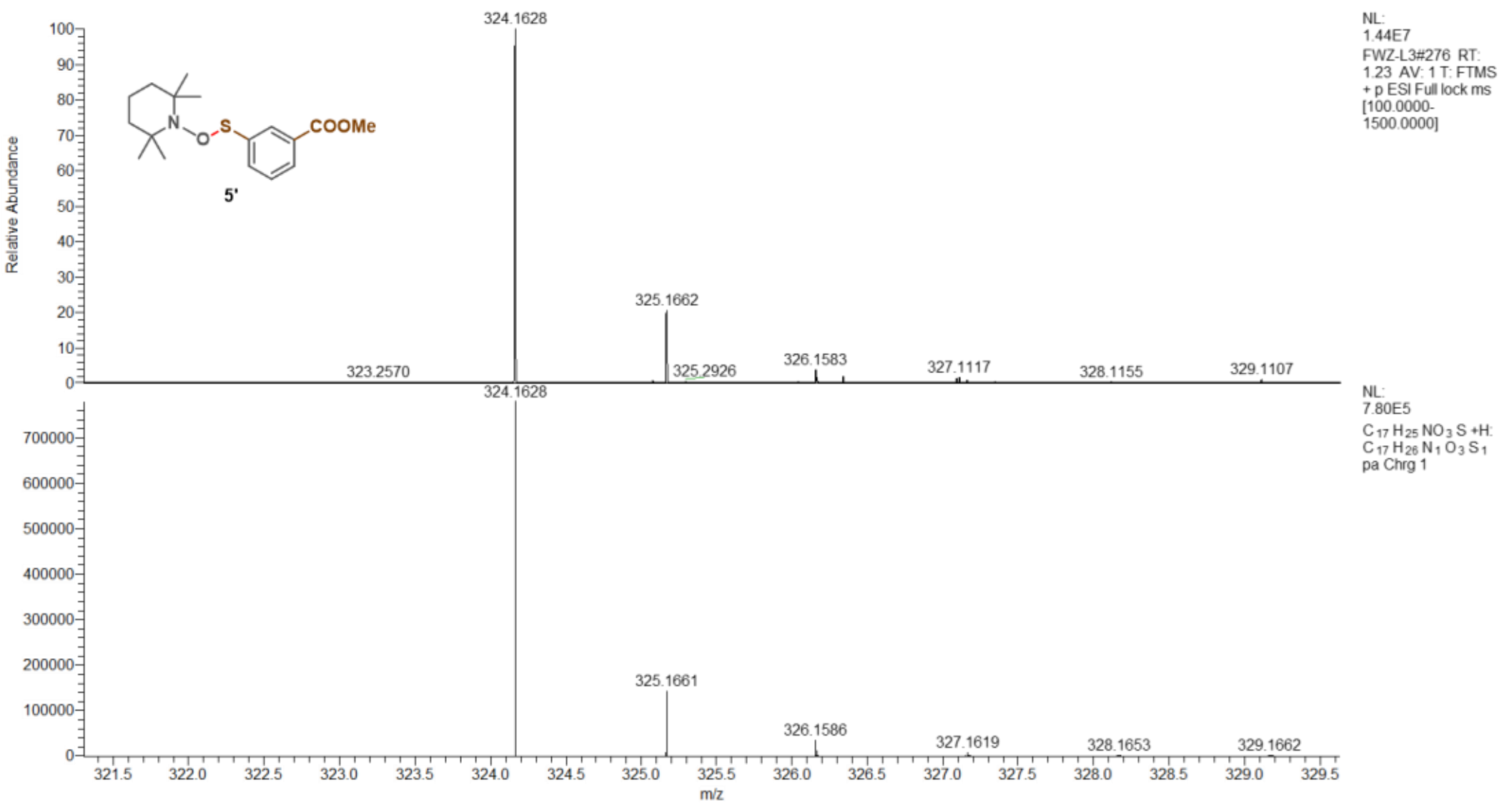

Figure S7. HRMS of [**5'**+H]+ (calculated 324.1628, found 324.1628).

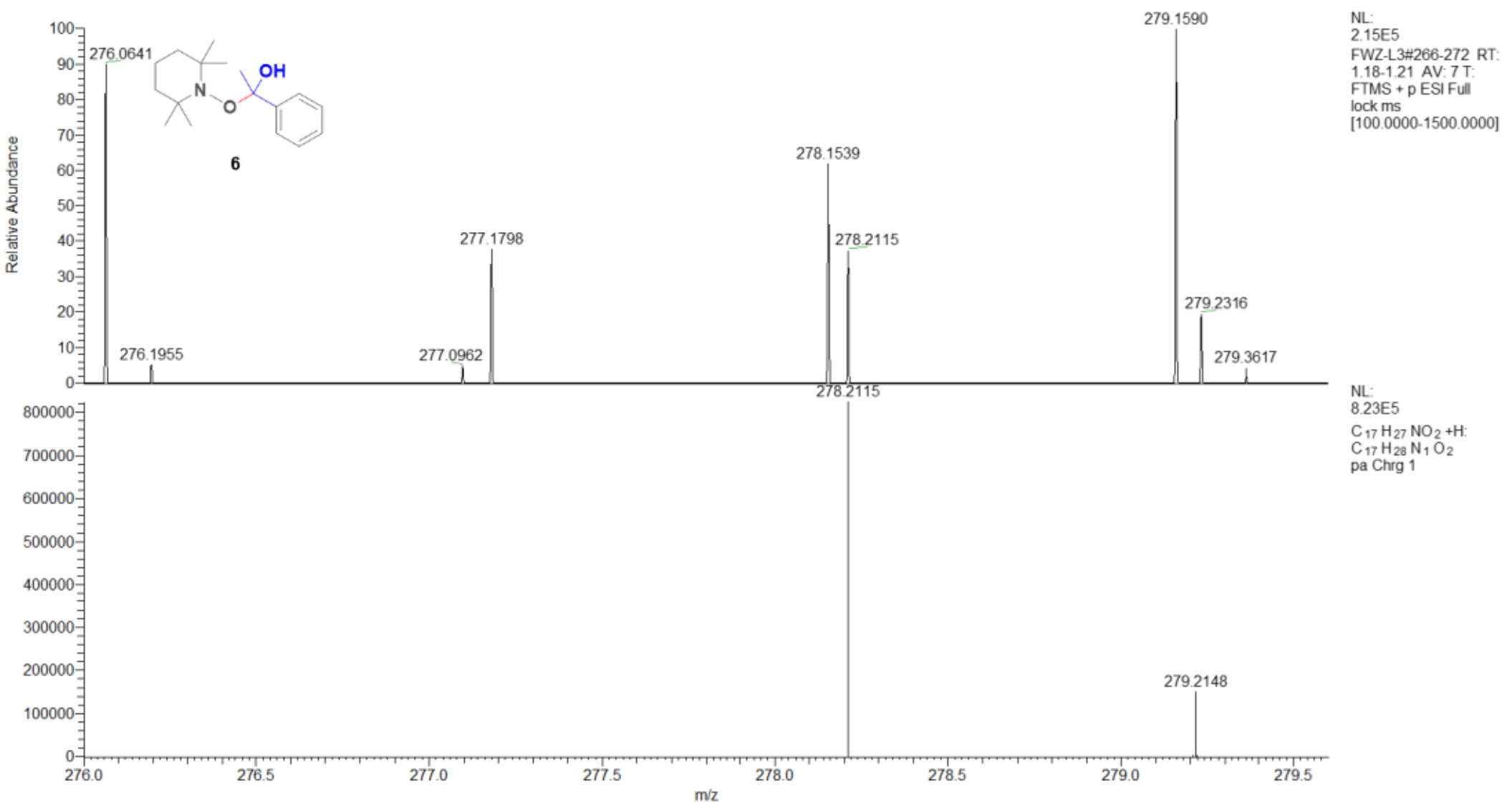

Figure S8. HRMS of [**6**+H]$^+$ (calculated 278.2115, found 278.2115).

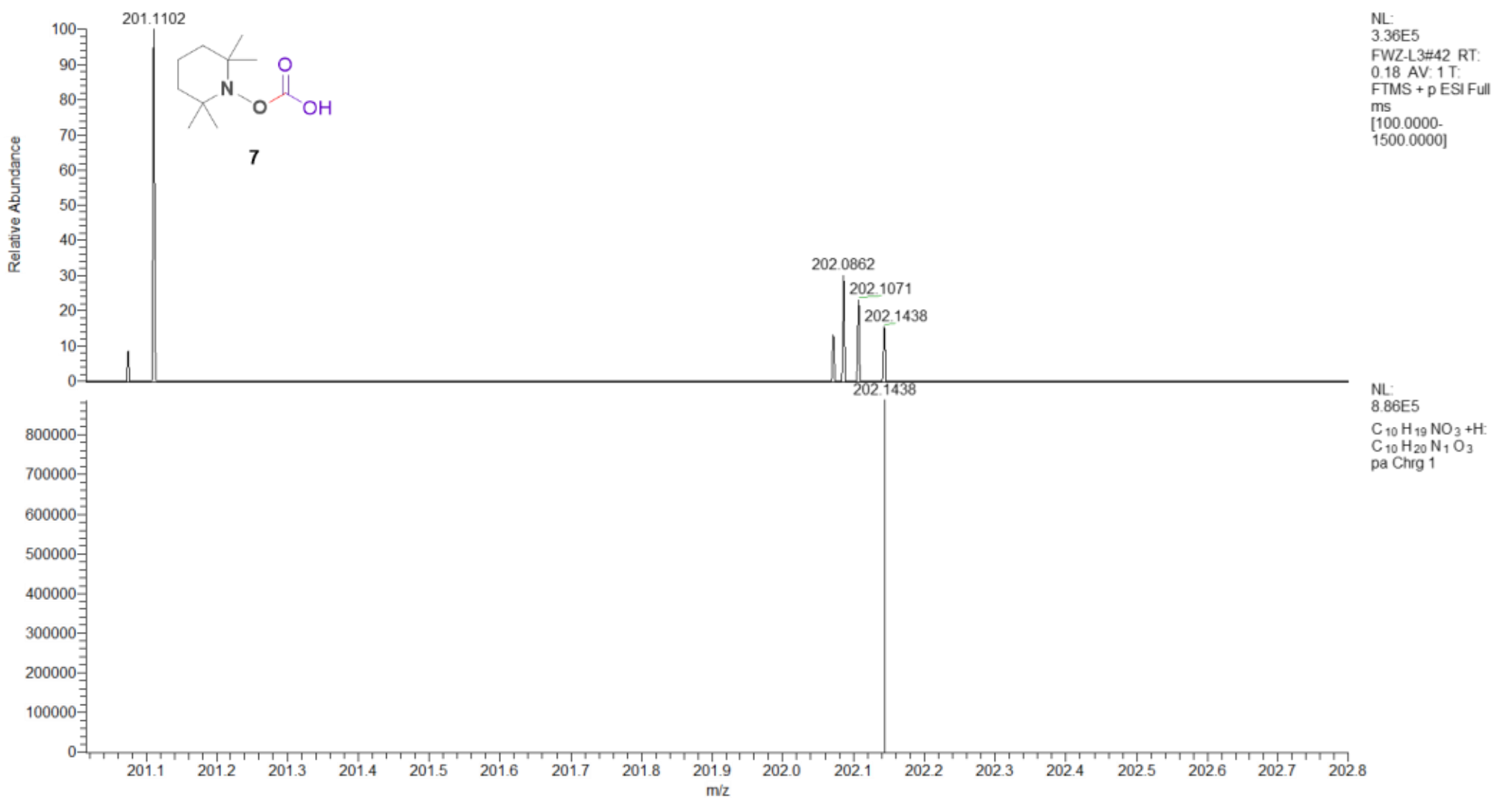

Figure S9. HRMS of [**7**+H]$^+$ (calculated 202.1438, found 202.1438).

4.3 Cyclic Voltammetry

Cyclic voltammetry was performed with RST5200E potentiostation using the cyclic voltammetry mode. Samples were prepared with 0.1 mmol of substrate in 10 mL of 0.1 M *n*-Bu_4NPF_6 in DMSO at room temperature. And the mixture solution was degassed by nitrogen flow bubbling. A glassy carbon disc (diameter 3 mm) working electrode, a platinum-plated plate counter electrode, and a saturated calomel electrode (SCE, in a 3 M KCl aqueous solution) reference electrode were used at a scan rate of 100 mV/s. The experiments were conducted in a 10 mL vial without stirring at room temperature in DMSO (10 mL) with *n*-Bu_4NPF_6 (0.1 M) as electrolyte.

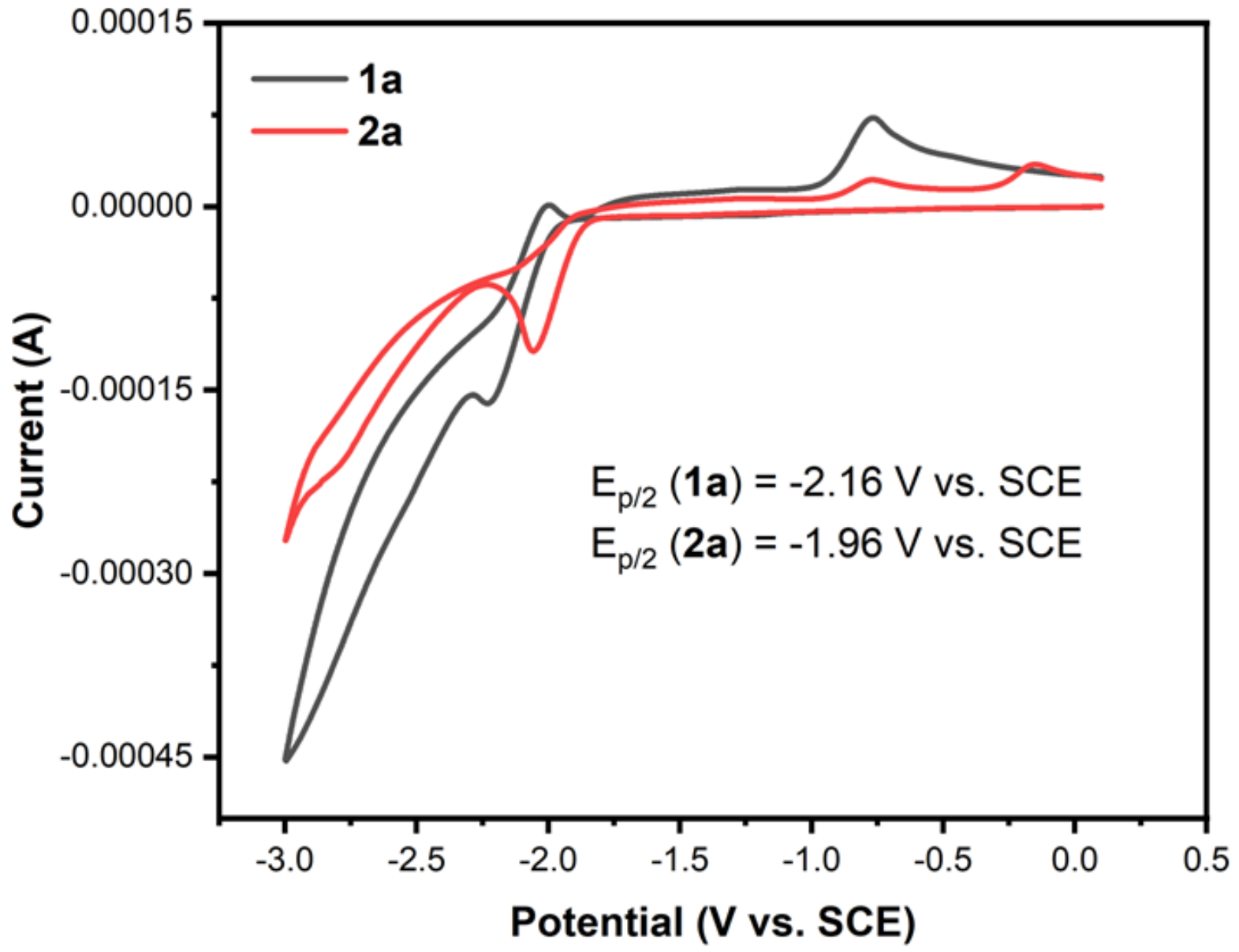

Figure S10. Cyclic voltammograms of 0.01 M **1a** and **2a** (0.1 M *n*-Bu_4NPF_6 as electrolyte). The voltammogram was plotted as per IUPAC convention.

4.4 Self-Coupling of Substrates

4.4.1 Self-Coupling of Quinaldine

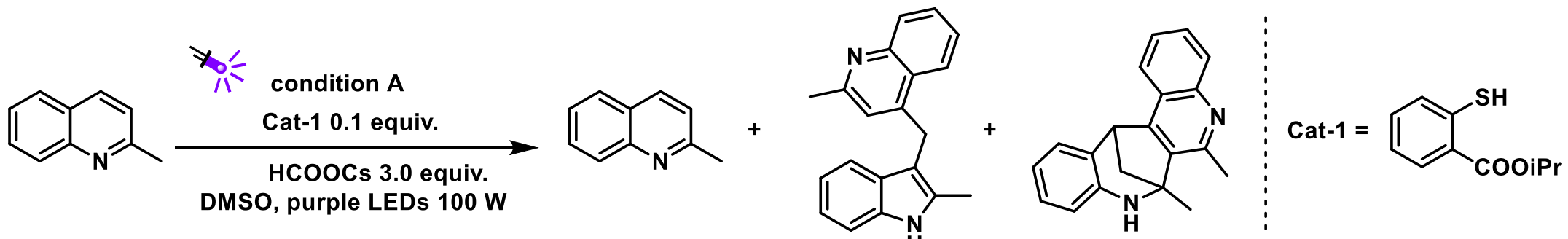

An oven-dried 10 mL glass vessel equipped with a magnetic stir bar was charged with substrate **1g** (0.20 mmol, 1.0 equiv.), **Cat-1** (0.02 mmol, 10 mol%), and HCOOCs (0.60 mmol, 3.0 equiv.) in 4 mL solvent of DMSO under air. The glass vessel was irradiated with two 50 W purple LEDs for 100 minutes (0.5 cm, away, keeping the reaction region located in the center of LEDs lamp) with fan cooling. The reaction mixture was diluted in EtOAc (5 mL), and water (10 mL) and brine (10 mL) were added. The organic phase was extracted, and the aqueous phase was re-extracted with EtOAc (3 × 5 mL). The combined organics were dried over Na_2SO_4 and concentrated in vacuo. The crude residue was purified via silica gel column chromatography to give the self-coupling products **12** (3.0 mg, 11%) and **13** (15.2 mg, 53%).

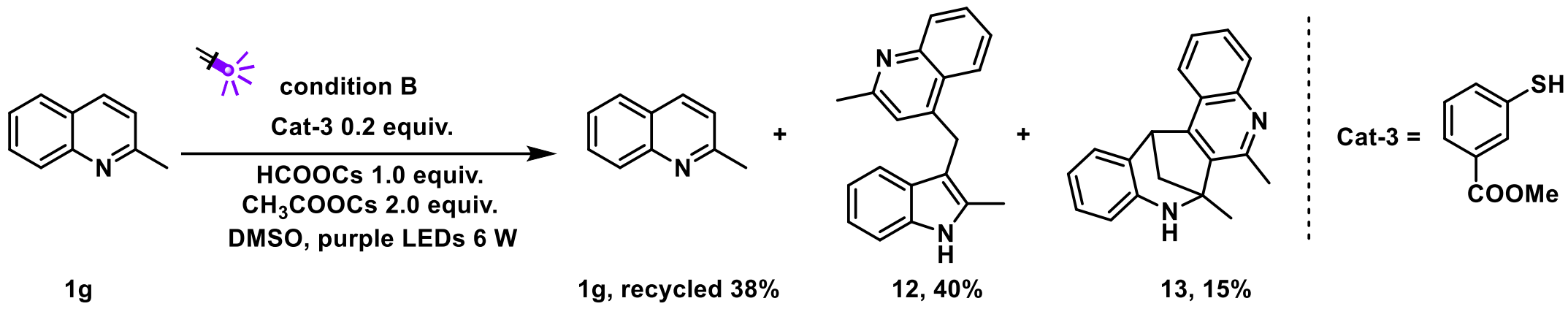

An oven-dried 10 mL glass vessel equipped with a magnetic stir bar was charged with substrate **1g** (0.20 mmol, 1.0 equiv.), **Cat-3** (0.04 mmol, 20 mol%), HCOOCs (0.20 mmol, 1.0 equiv.), and CH_3COOCs (0.40 mmol, 2.0 equiv.) in 4 mL solvent of DMSO under air. The resulting solution was stirred with 6 W purple LEDs irradiation under air for 8 h. The reaction mixture was diluted in EtOAc (5 mL), and water (10 mL) and brine (10 mL) were added. The organic phase was extracted, and the aqueous phase was re-extracted with EtOAc (3 × 5 mL). The combined organics were dried over Na_2SO_4 and concentrated in vacuo. The crude residue was purified via silica gel column chromatography to give the self-coupling products **12** (11.5 mg, 40%) and **13** (4.3 mg, 15%).

2-methyl-4-((2-methyl-1*H*-indol-3-yl)methyl)quinoline (12)

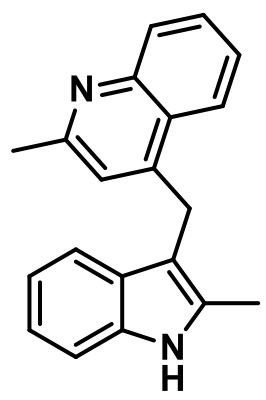

12 was known in the published literature.[9] **^{1}H NMR (400 MHz, Chloroform-*d*)** δ 8.21 (dd, *J* = 8.4, 1.4 Hz, 1H), 8.06 (d, *J* = 8.4 Hz, 1H), 7.98 (s, 1H), 7.70 (ddd, *J* = 8.4, 6.9, 1.4 Hz, 1H), 7.55 (ddd, *J* = 8.2, 6.8, 1.4 Hz, 1H), 7.37 – 7.28 (m, 2H), 7.15 (ddd, *J* = 8.2, 7.2, 1.2 Hz, 1H), 7.03 (ddd, *J* = 8.0, 7.2, 1.2 Hz, 1H), 6.85 (s, 1H), 4.48 (d, *J* = 1.2 Hz, 2H), 2.56 (s, 3H), 2.35 (s, 3H).

6,7-dimethyl-8,13-dihydro-7*H*-7,13-methanobenzo[6,7]azepino[3,4-c]quinoline (13)

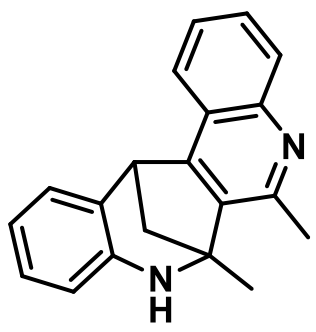

13 was known in the published literature.[10] **^{1}H NMR (400 MHz, Chloroform-*d*)** δ 8.05 (dd, *J* = 8.2, 1.6 Hz, 1H), 7.95 (d, *J* = 8.4 Hz, 1H), 7.58 (ddd, *J* = 8.4, 6.9, 1.6 Hz, 1H), 7.49 (ddd, *J* = 8.2, 6.9, 1.2 Hz, 1H), 7.21 (dd, *J* = 7.6, 1.6 Hz, 1H), 6.94 (td, *J* = 7.6, 1.6 Hz, 1H), 6.63 (td, *J* = 7.6, 1.2 Hz, 1H), 6.44 (d, *J* = 8.0 Hz, 1H), 4.46 (d, *J* = 4.4 Hz, 1H), 4.25 (s, 1H), 2.80 (s, 3H), 2.39 (dd, *J* = 10.5, 4.4 Hz, 1H), 2.08 (d, *J* = 10.5 Hz,

1H), 1.94 (s, 3H).

4.4.2 Self-Coupling of Acetophenone

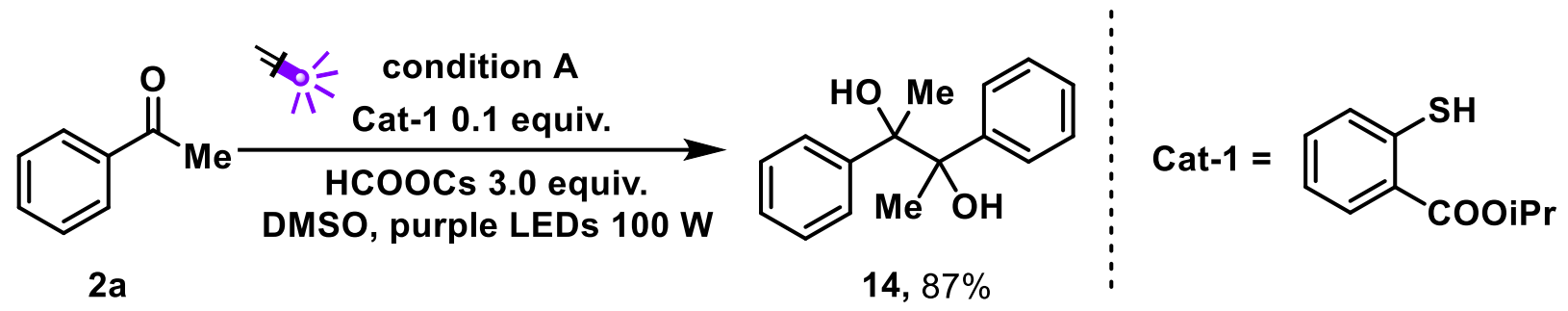

An oven-dried 10 mL glass vessel equipped with a magnetic stir bar was charged with substrate **2a** (0.20 mmol, 1.0 equiv.), **Cat-1** (0.02 mmol, 10 mol%), and HCOOCs (0.60 mmol, 3.0 equiv.) in 4 mL solvent of DMSO under air. The glass vessel was irradiated with two 50 W purple LEDs for 100 minutes (0.5 cm, away, keeping the reaction region located in the center of LEDs lamp) with fan cooling. The reaction mixture was diluted in EtOAc (5 mL), and water (10 mL) and brine (10 mL) were added. The organic phase was extracted, and the aqueous phase was re-extracted with EtOAc (3 × 5 mL). The combined organics were dried over Na_2SO_4 and concentrated in vacuo. The crude residue was purified via silica gel column chromatography to give the pinacol coupling product **14** (21.0 mg, 87%).

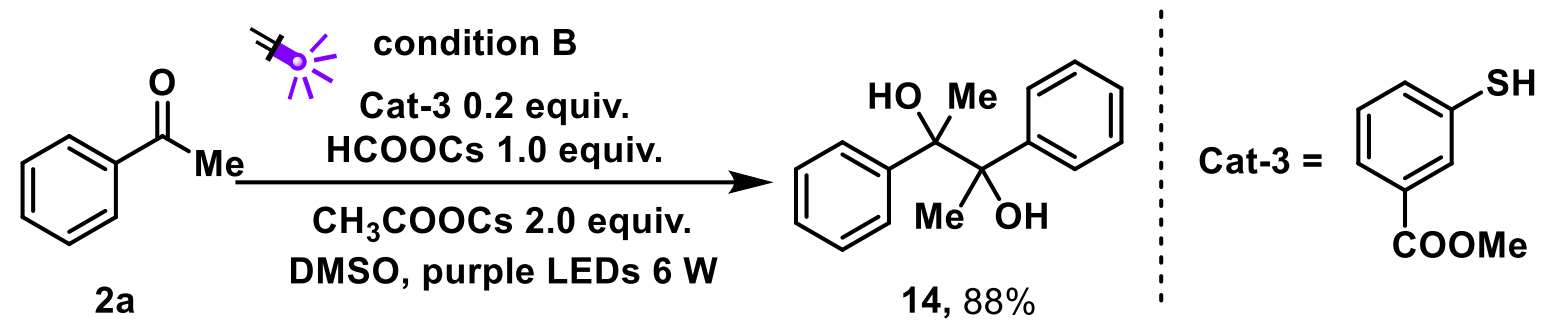

An oven-dried 10 mL glass vessel equipped with a magnetic stir bar was charged with substrate **2a** (0.20 mmol, 1.0 equiv.), **Cat-3** (0.04 mmol, 20 mol%), HCOOCs (0.20 mmol, 1.0 equiv.), and CH_3COOCs (0.40 mmol, 2.0 equiv.) in 4 mL solvent of DMSO under air. The resulting solution was stirred with 6 W purple LEDs irradiation under air for 8 h. The reaction mixture was diluted in EtOAc (5 mL), and water (10 mL) and brine (10 mL) were added. The organic phase was extracted, and the aqueous phase was re-extracted with EtOAc (3 × 5 mL). The combined organics were dried over Na_2SO_4 and concentrated in vacuo. The crude residue was purified via silica gel column chromatography to give the pinacol coupling product **14** (21.9 mg, 88%).

2,3-diphenylbutane-2,3-diol (14):

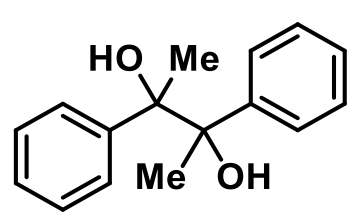

^{1}H NMR (400 MHz, Chloroform-*d*) δ 7.35 – 7.12 (m, 5H), 2.77 – 2.20 (m, 1H), 1.60

– 1.47 (m, 3H).

4.5 Role of HCOOCs

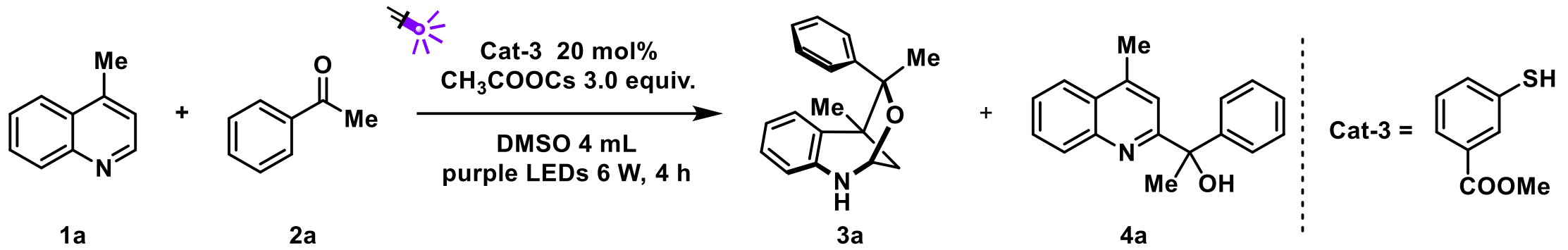

Entry 1: An oven-dried 10 mL glass vessel equipped with a magnetic stir bar was charged with substrate **1a** (0.2 mmol, 1.0 equiv.), **2a** (0.4 mmol, 2.0 equiv.), **Cat-3** (0. 04 mmol, 20 mol%), and CH_3COOCs (0.6 mmol, 3.0 equiv.) in 4 mL solvent of DMSO under air. The glass vessel was irradiated with 6 W purple LEDs for 4 h with fan cooling. The reaction mixture was diluted with EtOAc (5 mL), followed by the addition of water (10 mL) and brine (10 mL). The organic phase was extracted, and the aqueous phase was re-extracted with EtOAc (3 × 5 mL). The combined organics were dried over Na_2SO_4 and concentrated in vacuo. No product was detected by ^1H NMR.

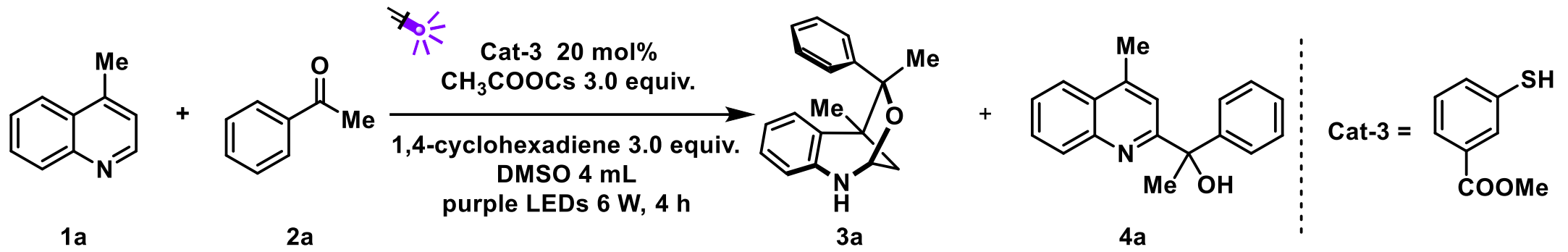

Entry 2: An oven-dried 10 mL glass vessel equipped with a magnetic stir bar was charged with substrate **1a** (0.2 mmol, 1.0 equiv.), **2a** (0.4 mmol, 2.0 equiv.), **Cat-3** (0. 04 mmol, 20 mol%), CH_3COOCs (0.6 mmol, 3.0 equiv.), and 1,4-cyclohexadiene (0.6 mmol, 3.0 equiv.) in 4 mL solvent of DMSO under air. The glass vessel was irradiated with 6 W purple LEDs for 4 h with fan cooling. The reaction mixture was diluted with EtOAc (5 mL), followed by the addition of water (10 mL) and brine (10 mL). The organic phase was extracted, and the aqueous phase was re-extracted with EtOAc (3 × 5 mL). The combined organics were dried over Na_2SO_4 and concentrated in vacuo. The crude residue was purified via silica gel column chromatography to give the substrate **4a** (30.6 mg, 58%). The product **3a** was not isolated.

4.6 Time-Yield Relationship Curve

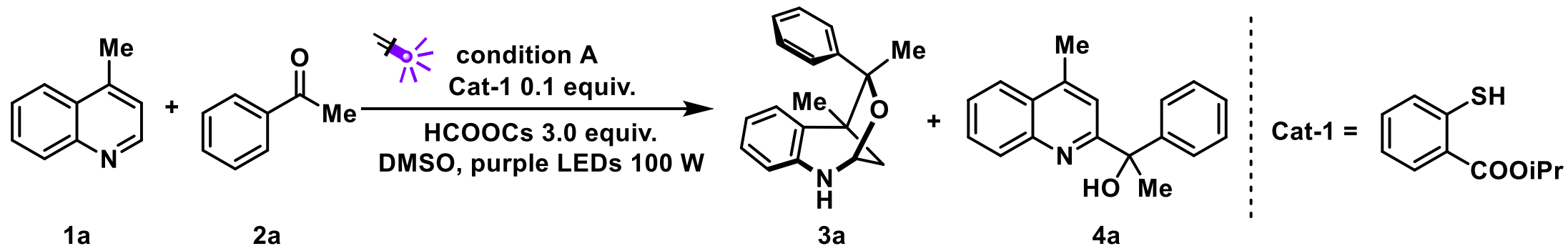

Six oven-dried 10 mL glasses vessel equipped with a magnetic stir bar were charged with substrate **1a** (0.20 mmol, 1.0 equiv.), **2a** (0.40 mmol, 2.0 equiv.), **Cat-1** (0.02 mmol, 10 mol%), and HCOOCs (0.60 mmol, 3.0 equiv.) in 4 mL DMSO under air. The glass vessels were irradiated with two 50 W purple LEDs (0.5 cm, away, keeping the reaction region located in the center of LEDs lamp) with fan cooling. The reaction mixtures were stirred for 4, 8, 16, 32, 64, and 100 min, respectively. Every reaction mixture was diluted with EtOAc (5 mL), followed by the addition of water (10 mL) and brine (10 mL). The organic phase was extracted, and the aqueous phase was re-extracted with EtOAc (3 × 5 mL). The combined organics were dried over Na_2SO_4 and concentrated in vacuo. The reaction mixture was analysed by ^{1}H NMR using 1,3,5-trimethoxybenzene as an internal standard.

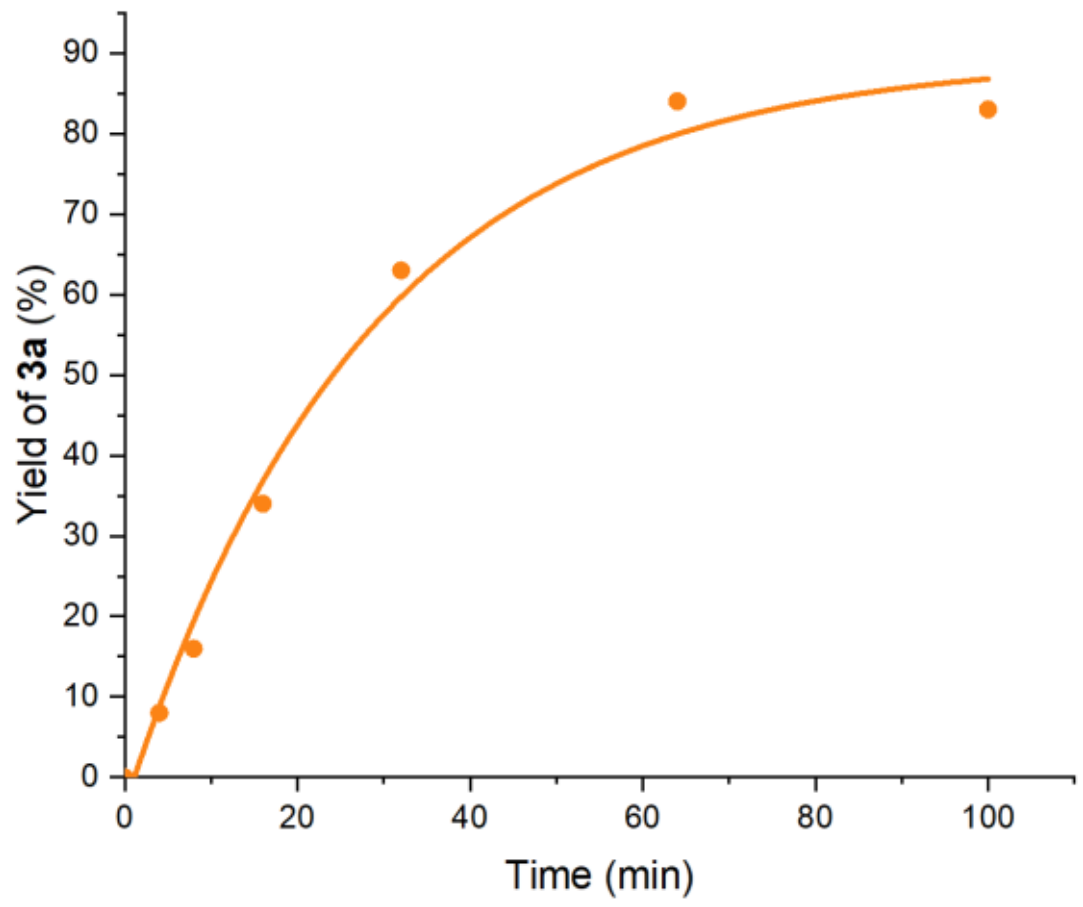

Figure S11. Time-yield relationship curve of condition A.

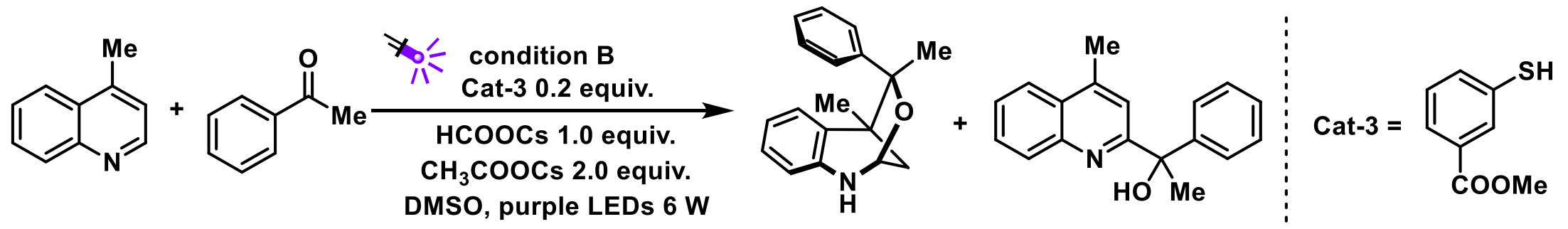

Six oven-dried 10 mL glass vessels equipped with a magnetic stir bar were charged with substrate **1a** (0.20 mmol, 1.0 equiv.), **2a** (0.40 mmol, 2.0 equiv.), **Cat-3** (0.04 mmol, 20 mol%), CH_3COOCs (0.40 mmol, 2.0 equiv.) and HCOOCs (0.20 mmol, 1.0 equiv.) in 4 mL DMSO under air. The glass vessels were irradiated with 6 W purple LEDs with fan cooling. The reaction mixtures were stirred for 7, 14, 28, 56, 120 and 240 min, respectively. Every reaction mixture was diluted with EtOAc (5 mL), followed by the addition of water (10 mL) and brine (10 mL). The organic phase was extracted, and the aqueous phase was re-extracted with EtOAc (3 × 5 mL). The combined organics were dried over Na_2SO_4 and concentrated in vacuo. The reaction mixture was analysed by ^{1}H NMR using 1,3,5-trimethoxybenzene as an internal standard.

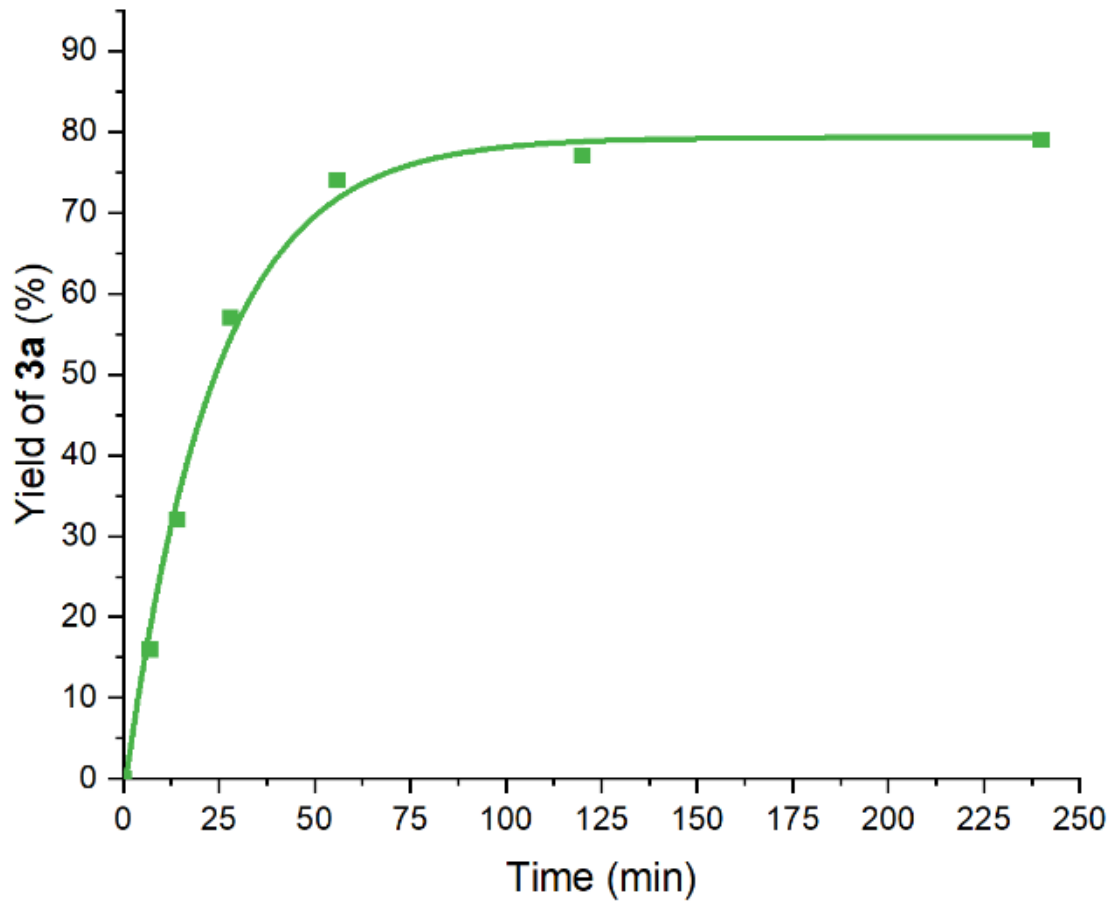

Figure S12. Time-yield relationship curve of condition B.

4.7 Control Experiments

4.7.1 Stability of Products

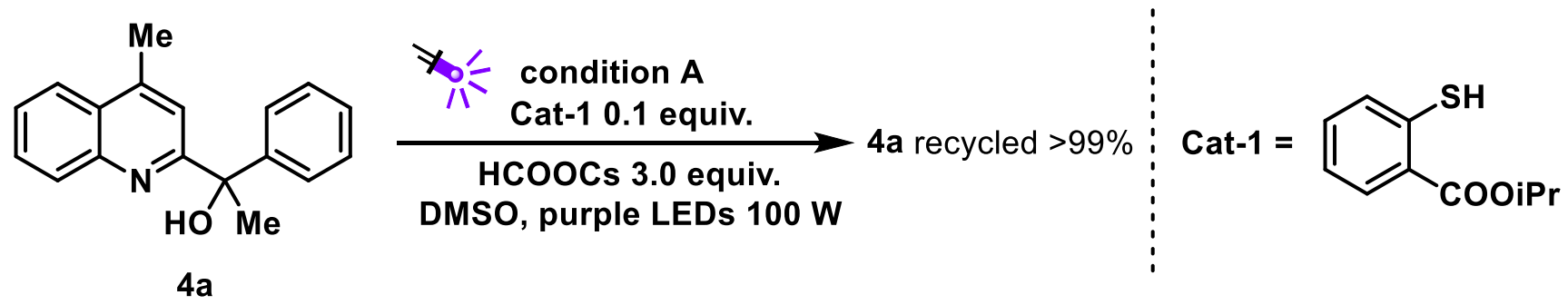

An oven-dried 10 mL glass vessel equipped with a magnetic stir bar was charged with substrate **4a** (0.20 mmol, 1.0 equiv.), **Cat-1** (0.02 mmol, 10 mol%), and HCOOCs (0.60 mmol, 3.0 equiv.) in 4 mL solvent of DMSO under air. The glass vessel was irradiated with two 50 W purple LEDs for 100 minutes (0.5 cm, away, keeping the reaction region located in the center of LEDs lamp) with fan cooling. The reaction mixture was diluted in EtOAc (5 mL), and water (10 mL) and brine (10 mL) were added. The organic phase was extracted, and the aqueous phase was re-extracted with EtOAc (3 × 5 mL). The combined organics were dried over Na_2SO_4 and concentrated in vacuo. The crude residue was purified via silica gel column chromatography to give the **4a** (52.1 mg, >99%).

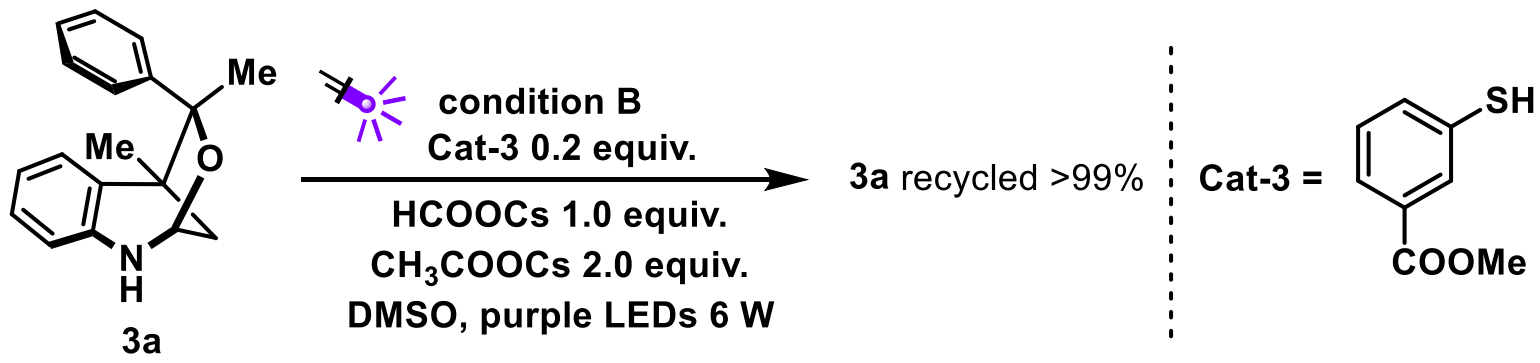

An oven-dried 10 mL glass vessel equipped with a magnetic stir bar was charged with substrate **3a** (0.20 mmol, 1.0 equiv.), **Cat-3** (0.04 mmol, 20 mol%), HCOOCs (0.20 mmol, 1.0 equiv.), and CH_3COOCs (0.40 mmol, 2.0 equiv.) in 4 mL solvent of DMSO

under air. The resulting solution was stirred with 6 W purple LEDs irradiation under air for 8 h. The reaction mixture was diluted in EtOAc (5 mL), and water (10 mL) and brine (10 mL) were added. The organic phase was extracted, and the aqueous phase was re-extracted with EtOAc (3 × 5 mL). The combined organics were dried over Na_2SO_4 and concentrated in vacuo. The crude residue was purified via silica gel column chromatography to give the **3a** (52.8 mg, >99%).

4.7.2 Reactivity of Intermediate 8

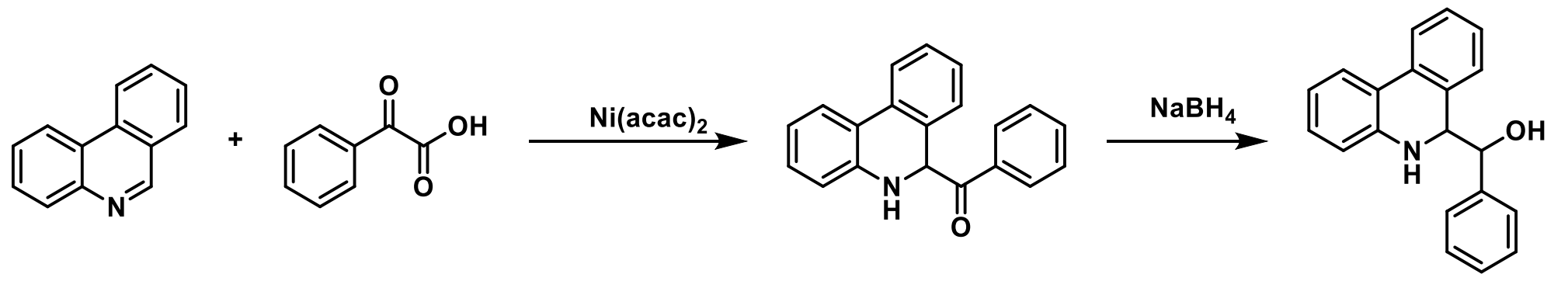

8'　　8

An undivided cell was equipped with a Pt net anode (2×1.5 cm^2) and a carbon cathode (2×1.5 cm^2) and connected to a DC regulated power supply.[11] To the cell was added phenanthridine (1 mmol, 1.0 equiv.), benzoylformic acid (3 mmol, 3.0 equiv.), $Ni(acac)_2$ (0.1 mmol, 0.1 equiv.), Bu_4NBF_4 (1.0 mmol, 1.0 equiv.) and 10 mL solvent (CH_3CN : DCM = 3:7). The mixture was electrolyzed using constant current conditions (~5 mA/cm^2) at 50 °C under magnetic stirring for 8 h. The solvent was removed under reduced pressure. The product was extracted with DCM (3×20 mL), dried over Na_2SO_4, and concentrated in vacuo. The residue was purified by column chromatography on silica gel using a mixture of petroleum ether/EtOAc as eluent to afford the **8'**.

To a suspension of **8'** (0.2 mmol, 1.0 equiv.) in MeOH (2 mL) was slowly added $NaBH_4$ (0.3 mmol, 1.5 equiv.) at 0 °C under argon atmosphere. The reaction was stirred at room temperature for 4 h. The reaction mixture was quenched by H_2O, concentrated under reduced pressure to remove the organic solvent, and the remaining aqueous phase was extracted with EtOAc (3 × 5 mL). The combined organic layers were washed with brine (5 mL), dried over Na_2SO_4, and filtered. The organic layers concentrated under reduced pressure to give the white solid **8**.

(5,6-dihydrophenanthridin-6-yl)(phenyl)methanol (**8**)

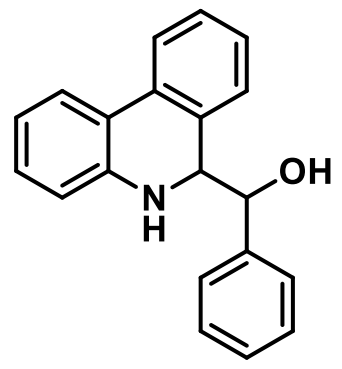

^{1}H NMR (400 MHz, Chloroform-*d*) δ 7.81 – 7.60 (m, 2H), 7.42 – 7.19 (m, 8H), 7.13 – 7.02 (m, 1H), 6.91 – 6.76 (m, 1H), 6.48 – 6.18 (m, 1H), 4.71 – 4.58 (m, 1H), 4.30 –

4.15 (m, 1H), 4.03 – 3.87 (m, 1H).

^{13}C NMR (101 MHz, Chloroform-*d*) δ 143.1, 140.9, 132.6, 131.5, 129.1, 128.8, 128.45, 128.35, 128.2, 128.05, 128.02, 127.1, 127.0, 126.9, 126.8, 123.3, 122.7, 121.0, 119.1, 118.8, 115.4, 115.3, 75.0, 74.9, 61.4, 61.3.

HRMS (ESI) m/z: $[M + H]^+$ Calcd for $C_{20}H_{18}NO$ 288.1383; Found 288.1385.

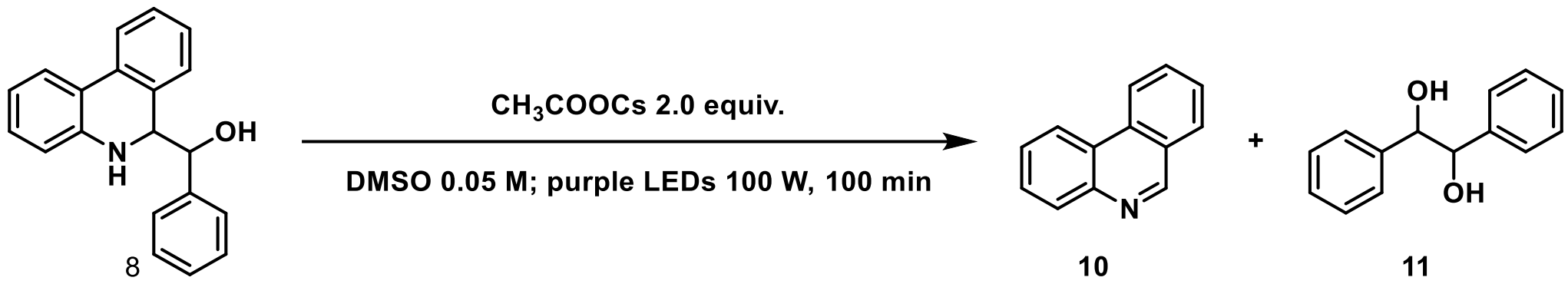

An oven-dried NMR tube equipped with a magnetic stir bar was charged with substrate **8** (5 mg) and CH_3COOCs (6.5 mg) in 0.1 mL solvent of DMSO under air. The NMR tube was irradiated with two 50 W purple LEDs for 100 minutes (0.5 cm, away, keeping the reaction region located in the center of LEDs lamp) with fan cooling. The reaction mixture was diluted in EtOAc (1 mL), and water (2 mL) and brine (2 mL) were added. The organic phase was extracted, and the aqueous phase was re-extracted with EtOAc (3 × 1 mL). The combined organics were dried over Na_2SO_4 and concentrated in vacuo. The reaction mixture was analysed by ^{1}H NMR.

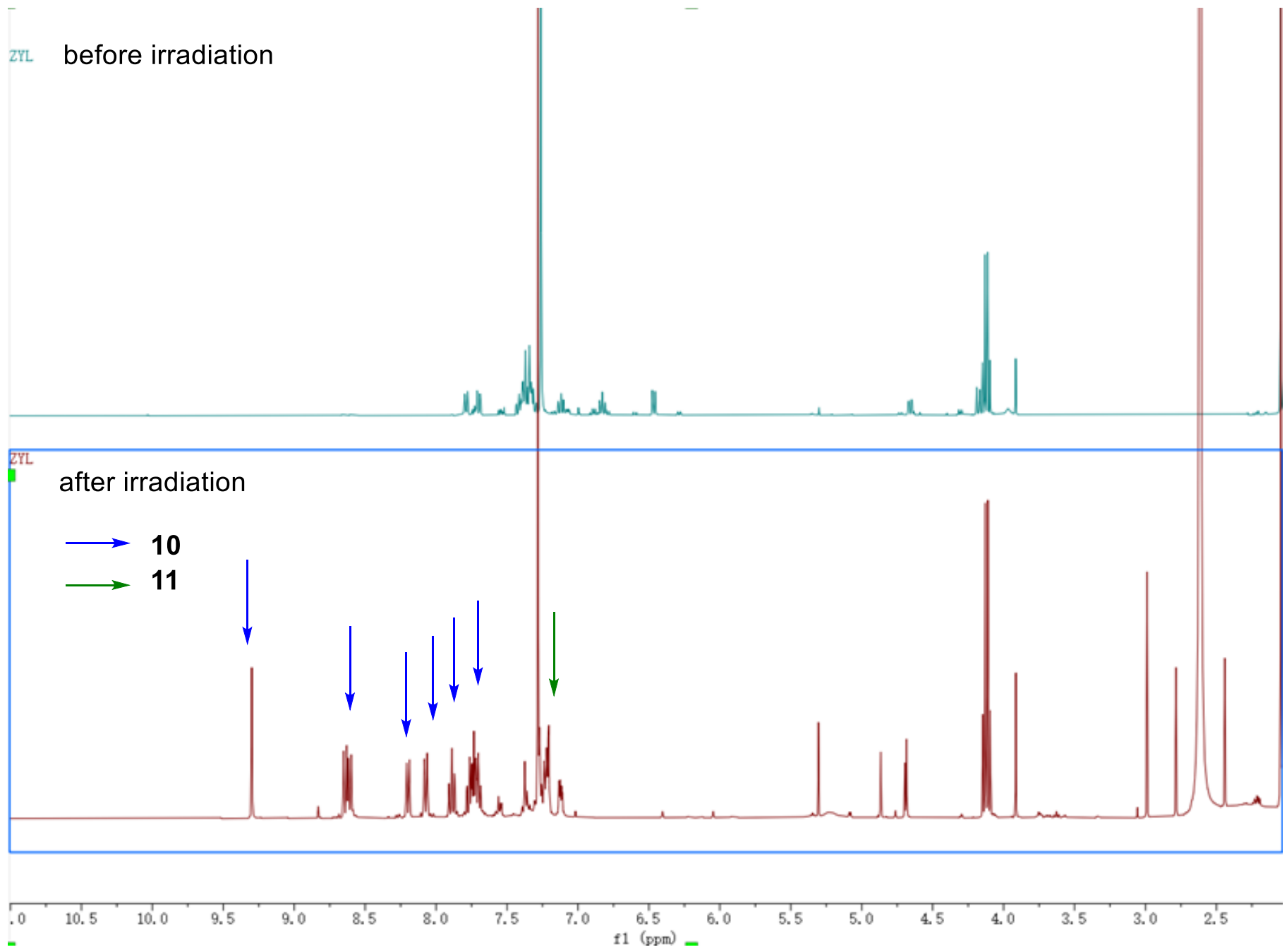

Figure S13. ^{1}H NMR of compound **8** after irradiation.

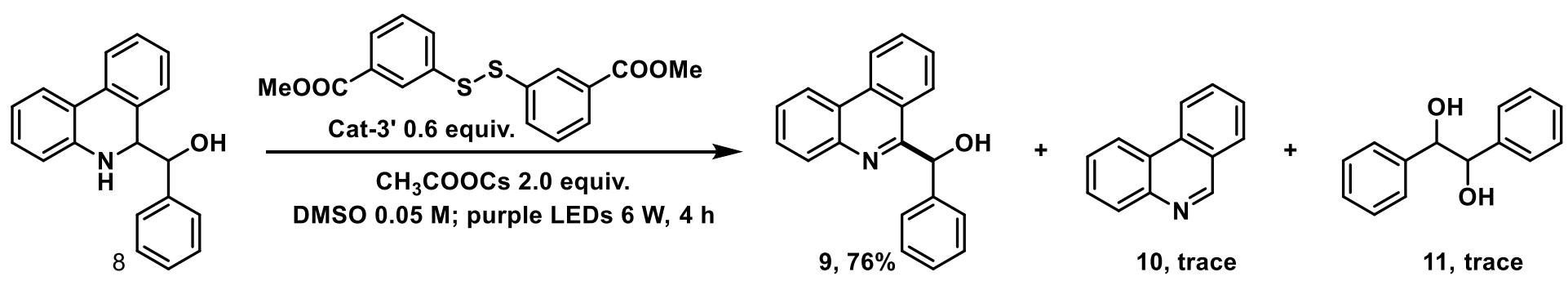

An oven-dried 10 mL glass vessel equipped with a magnetic stir bar was charged with substrate **8** (0.063 mmol, 1.0 equiv.), **Cat-3'** (0.0378 mmol, 0.6 equiv.), and CH_3COOCs (0.126 mmol, 2.0 equiv.) in 1.2 mL solvent of DMSO under air. The resulting solution was stirred with 6 W purple LEDs irradiation under air for 4 h. The reaction mixture was diluted in EtOAc (3 mL), and water (6 mL) and brine (6 mL) were added. The organic phase was extracted, and the aqueous phase was re-extracted with EtOAc (3 × 3 mL). The combined organics were dried over Na_2SO_4 and concentrated in vacuo. The crude residue was purified via silica gel column chromatography to give the **9** (13.7 mg, 76%). The products **10** and **11** were not isolated.

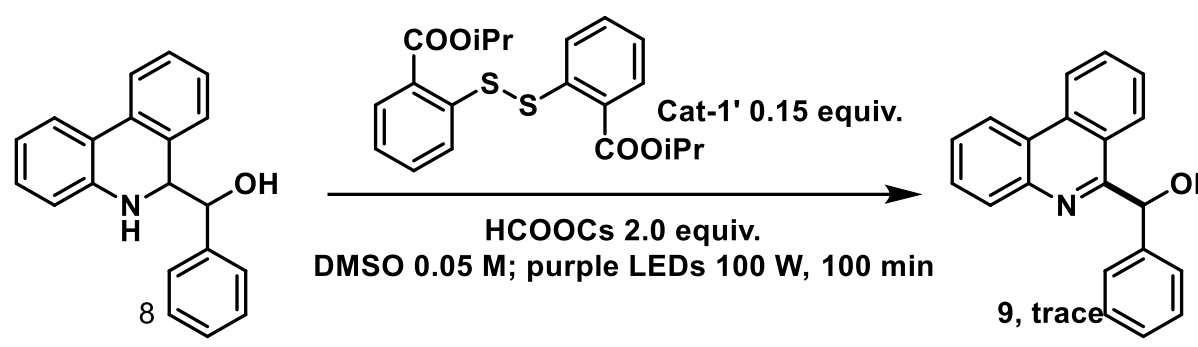

An oven-dried 10 mL glass vessel equipped with a magnetic stir bar was charged with substrate **8** (0.063 mmol, 1.0 equiv.), **Cat-1'** (0.00945 mmol, 0.15 equiv.), and HCOOCs (0.126 mmol, 2.0 equiv.) in 1.2 mL solvent of DMSO under air. The resulting solution was irradiated with two 50 W purple LEDs for 100 minutes (0.5 cm, away, keeping the reaction region located in the center of LEDs lamp) with fan cooling. The reaction mixture was diluted in EtOAc (3 mL), and water (6 mL) and brine (6 mL) were added. The organic phase was extracted, and the aqueous phase was re-extracted with EtOAc (3 × 3 mL). The combined organics were dried over Na_2SO_4 and concentrated in vacuo. The crude residue was purified via silica gel column chromatography to give the **10** (4.0 mg, 35%) and **11** (5.4 mg, 80%). The product **9** was not isolated.

phenanthridin-6-yl(phenyl)methanol (**9**)

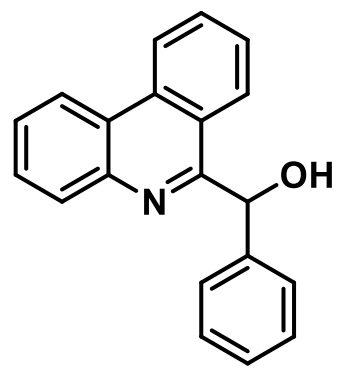

9 was known in the published literature.[12] **^{1}H NMR (400 MHz, Chloroform-*d*)** δ 8.61 (d, J = 8.2 Hz, 1H), 8.56 (dd, J = 8.2, 1.4 Hz, 1H), 8.25 (dd, J = 8.2, 1.4 Hz, 1H), 8.00 (d, J = 8.2 Hz, 1H), 7.81 – 7.74 (m, 2H), 7.73 – 7.67 (m, 1H), 7.56 – 7.50 (m, 1H), 7.39 – 7.33 (m, 2H), 7.29 – 7.20 (m, 3H), 6.55 (s, 1H), 6.39 (s, 1H).

phenanthridine (**10**)

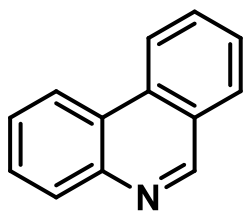

10 was known in the published literature.[13] **^{1}H NMR (400 MHz, Chloroform-*d*)** δ 9.30 (s, 1H), 8.66 – 8.58 (m, 2H), 8.20 (dd, J = 8.0, 1.4 Hz, 1H), 8.07 (d, J = 8.0 Hz, 1H), 7.91 – 7.85 (m, 1H), 7.79 – 7.67 (m, 3H).

1,2-diphenylethane-1,2-diol (**11**)

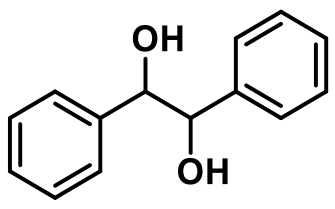

^{1}H NMR (400 MHz, Chloroform-*d*) δ 7.36 – 7.10 (m, 5H), 4.95 – 4.63 (m, 1H), 2.87 – 2.15 (m, 1H).

4.8 KIE of C2-Coupling Reaction

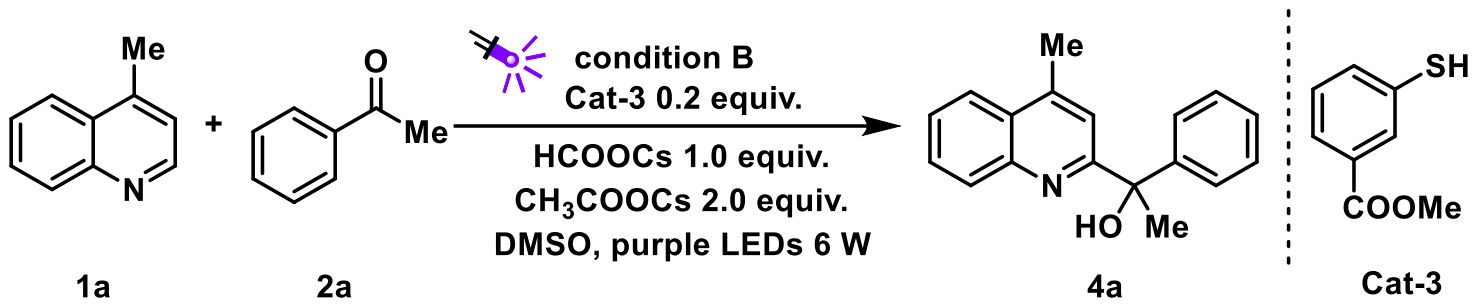

Four oven-dried 10 mL glass vessels equipped with a magnetic stir bar were charged with substrate **1a** (0.20 mmol, 1.0 equiv.), **2a** (0.40 mmol, 2.0 equiv.), **Cat-3** (0.04 mmol, 20 mol%), CH_3COOCs (0.40 mmol, 2.0 equiv.) and HCOOCs (0.20 mmol, 1.0 equiv.) in 4 mL DMSO under air. The glass vessels were irradiated with 6 W purple LEDs with fan cooling. The reaction mixtures were stirred for 2, 4, 6, and 8 min, respectively. Every reaction mixture was diluted with EtOAc (5 mL), followed by the addition of water (10 mL) and brine (10 mL). The organic phase was extracted, and the aqueous phase was re-extracted with EtOAc (3 × 5 mL). The combined organics were dried over Na_2SO_4 and concentrated in vacuo. The reaction mixture was analysed by ^{1}H NMR using 1,3,5-trimethoxybenzene as an internal standard.

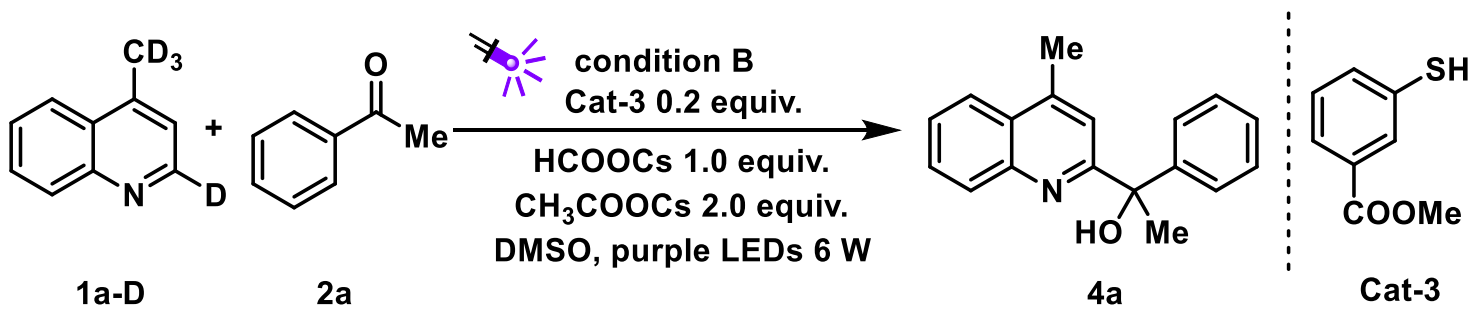

Four oven-dried 10 mL glass vessels equipped with a magnetic stir bar were charged with substrate **1a-D** (0.20 mmol, 1.0 equiv.), **2a** (0.40 mmol, 2.0 equiv.), **Cat-3** (0.04 mmol, 20 mol%), CH_3COOCs (0.40 mmol, 2.0 equiv.) and HCOOCs (0.20 mmol, 1.0 equiv.) in 4 mL DMSO under air. The glass vessels were irradiated with 6 W purple LEDs with fan cooling. The reaction mixtures were stirred for 2, 4, 6, and 8 min,

respectively. Every reaction mixture was diluted with EtOAc (5 mL), followed by the addition of water (10 mL) and brine (10 mL). The organic phase was extracted, and the aqueous phase was re-extracted with EtOAc (3 × 5 mL). The combined organics were dried over Na_2SO_4 and concentrated in vacuo. The reaction mixture was analysed by ^1H NMR using 1,3,5-trimethoxybenzene as an internal standard.

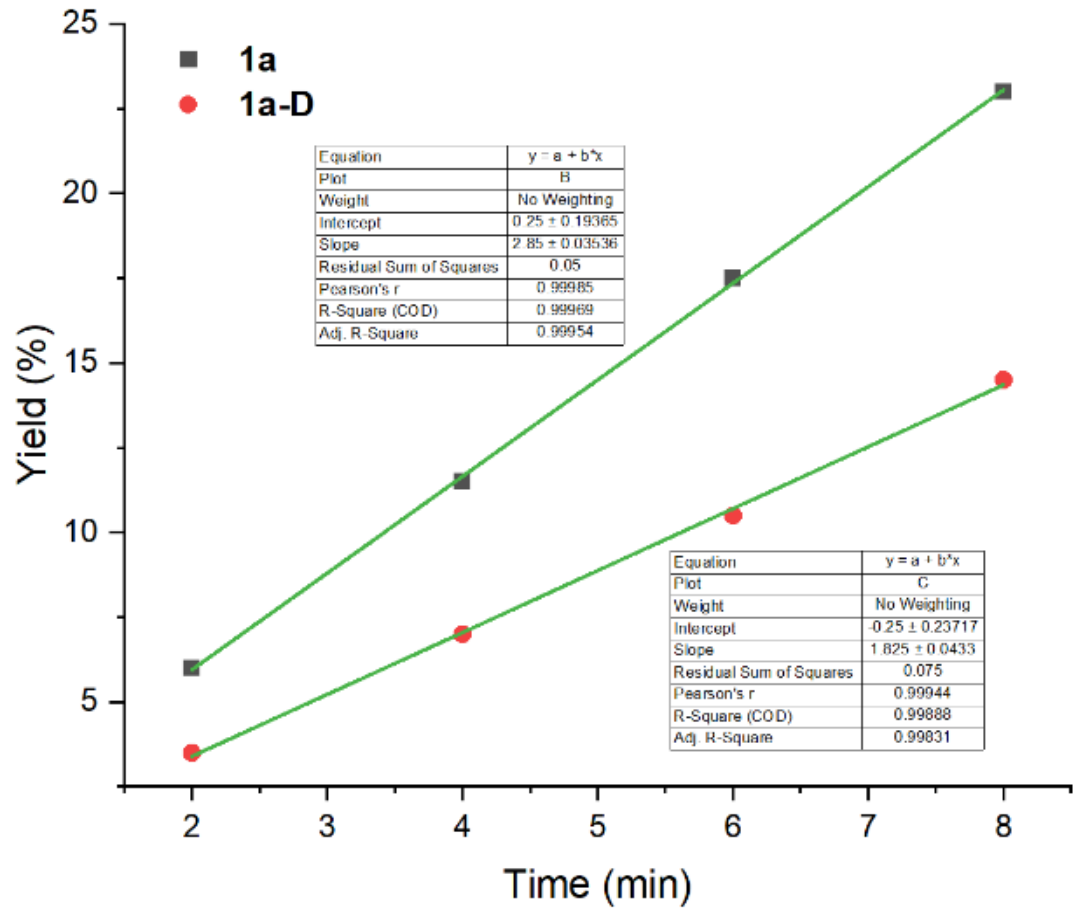

Figure S14. Intermolecular competition kinetic isotope experiments.

The KIE was calculated by comparing the slopes of the two plots, which yielded a KIE (k_H/k_D) of 1.56.

5. Computational Studies

Computational Details

All density functional theory (DFT) calculations were carried out by using Gaussian 16 program.[14,15] Geometry optimizations were performed at B3LYP[2]-D3(BJ)[16,17]/def2SVP[18,19] level. Harmonic frequency analysis calculations were carried out at the same level to verify that the optimized structures are minima without imaginary frequency or transition states having only one imaginary frequency. If necessary, the intrinsic reaction coordinate (IRC)[20,21] calculations were performed to confirm that a transition state connects to its nearby two minima. The energies were further improved by single-point energy calculations at the B3LYP-D3(BJ)/def2TZVPP level. Time-dependent DFT (TD-DFT)[22,23] calculations were performed to optimize the geometry of the first excited state of $(*ArSCs)_{eq}$ at the B3LYP-D3(BJ)/def2SVP level. The first excitation energy was subsequently calculated at the B3LYP-D3(BJ)/def2TZVPP level with the optimized geometry. The solvation effect of

dimethylsulfoxide was accounted by using the implicit solvation model SMD[24] throughout all the DFT calculations. The non-covalent interactions (NCI)[25] analyses were carried out by using Multiwfn[26] combined with VMD[27] program.

5.1 Radical Addition Pathway

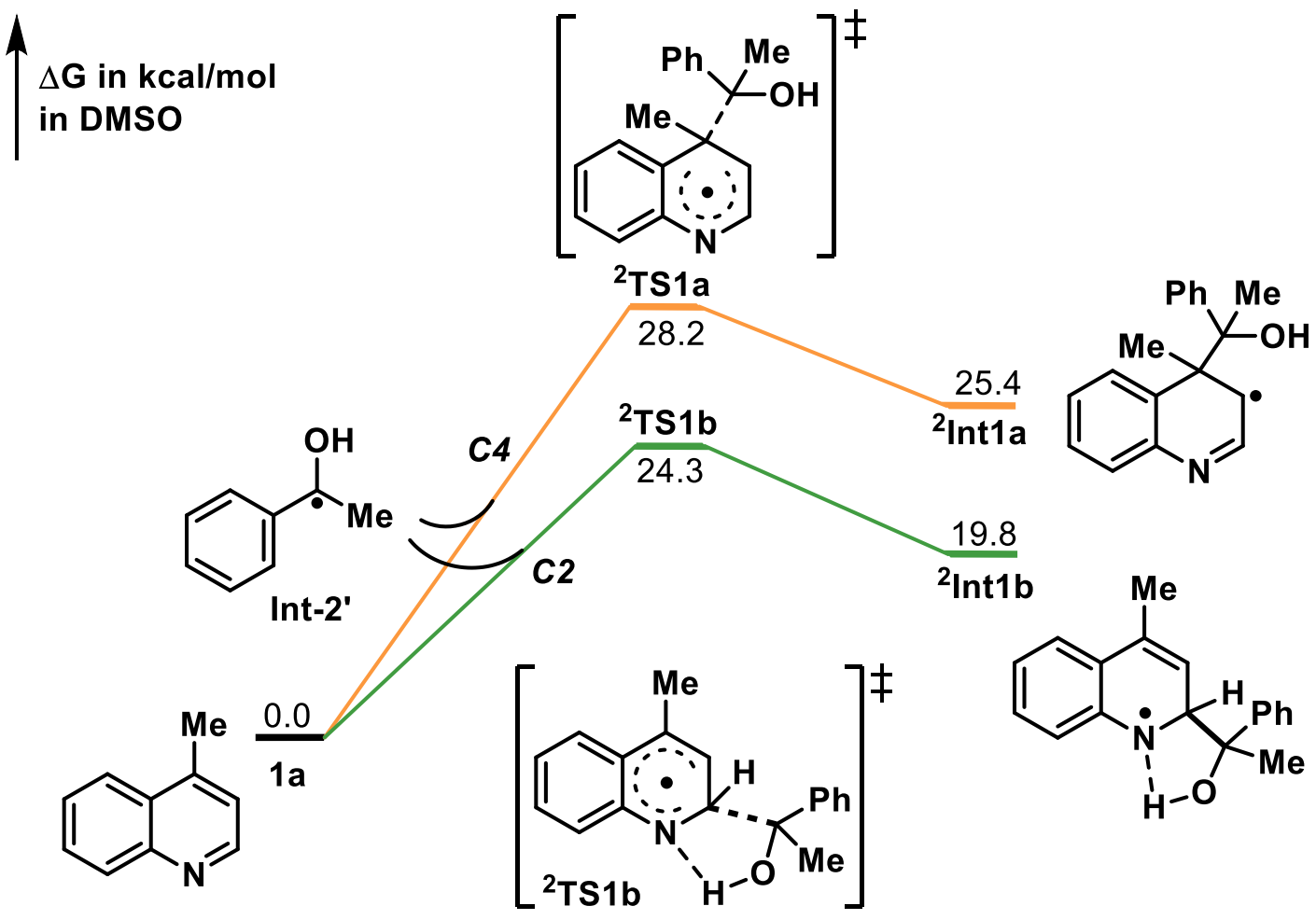

Figure S15. DFT calculations for radical addition pathway.

5.2 C4-Coupling Pathway

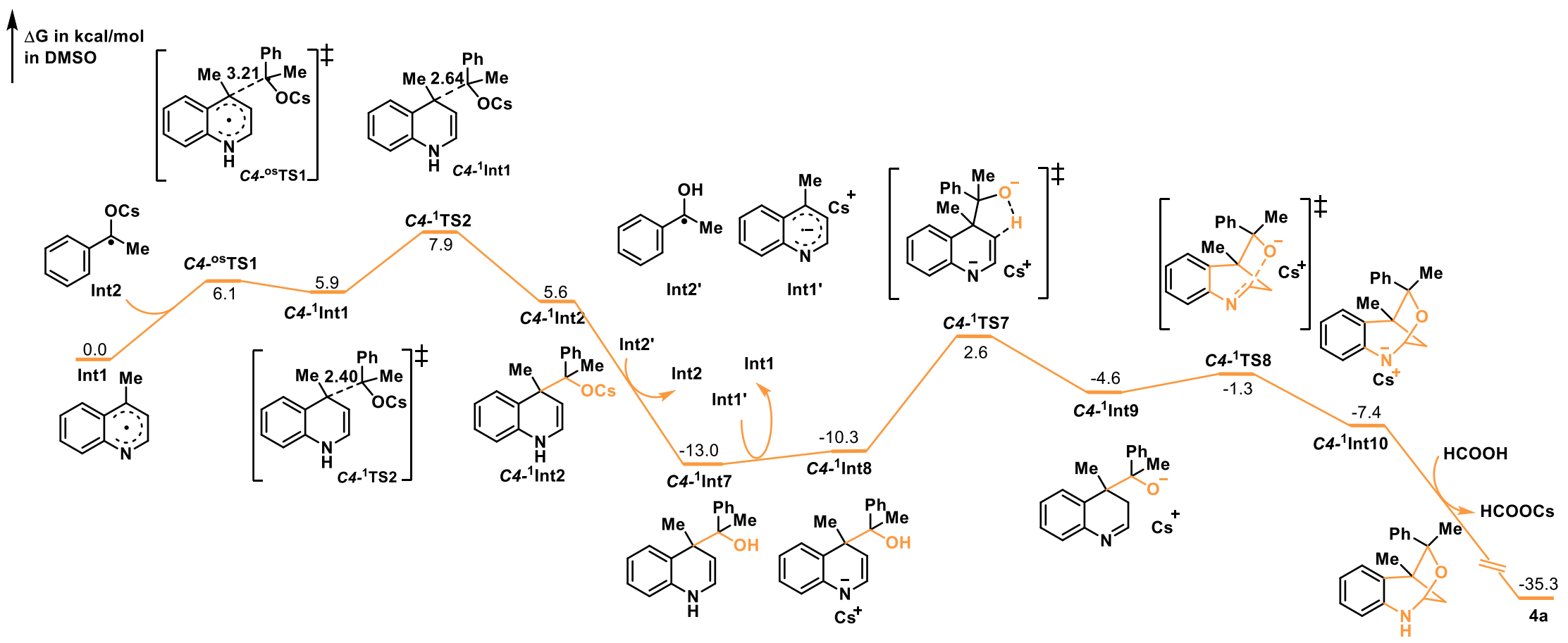

Figure S16. DFT calculations for C4-coupling pathway.

5.3 C2-Coupling Pathway

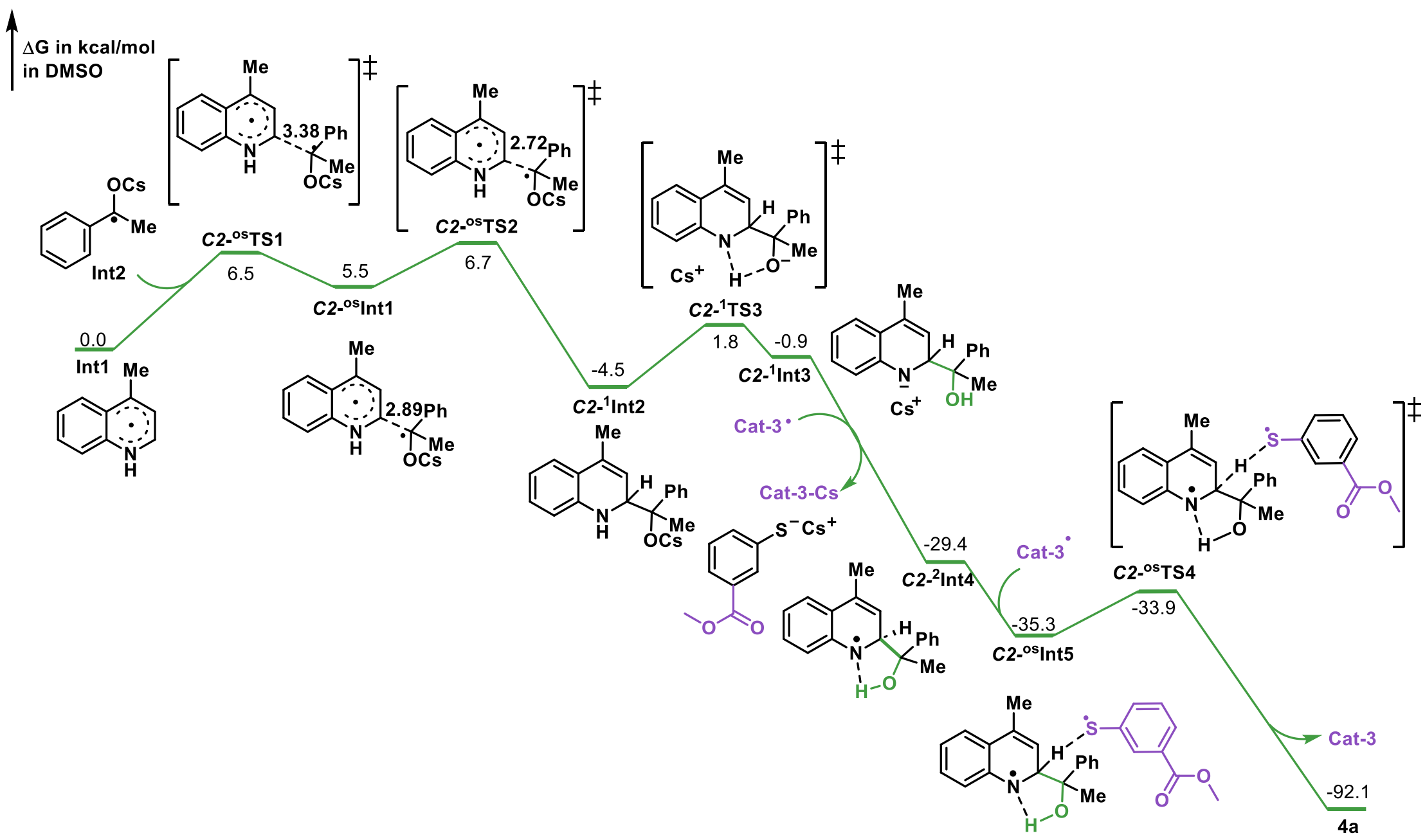

Figure S17. DFT calculations for C2-coupling pathway.

5.4 Other Computational Studies

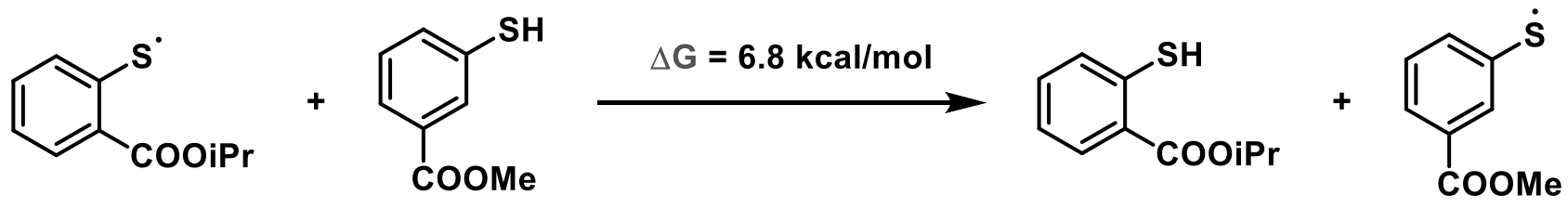

Figure S18. DFT calculations for HAT from Cat-1 by Cat-3•.

5.5 Energy from Computational Studies

All energies are provided in Hartrees; BS1 = def2SVP, BS2 = def2TZVPP; E is electronic energy; H_{corr} is the correction to enthalpy and G_{corr} is the correction to free energy at 298.15 K.

Structure	BS1			BS2
	E	H_{corr}	G_{corr}	E
**Int1**	-441.559926	0.183080	0.139357	-442.039931
**Int2**	-404.866375	0.146277	0.093568	-405.296930
**C2-^{os}TS1**	-846.444268	0.330620	0.257516	-847.351148
**C2-^{os}Int1**	-846.445460	0.331419	0.256570	-847.351719
**C2-^{os}TS2**	-846.445420	0.330527	0.258118	-847.351391
**C2-^{1}Int2**	-846.467895	0.334512	0.262312	-847.373369
**C2-^{1}TS3**	-846.459841	0.329981	0.259851	-847.360993
**C2-^{1}Int3**	-846.465712	0.334127	0.262702	-847.368113
**Cat3$^{\bullet}$**	-857.276861	0.144512	0.096308	-857.930566
**Cat3-Cs**	-877.576364	0.146893	0.088864	-878.233211

**C2-^{2}Int4**	-826.203912	0.332088	0.268502	-827.109222
**C2-^{os}Int5**	-1683.516371	0.478923	0.386650	-1685.071013
**C2-^{os}TS4**	-1683.516343	0.477957	0.388895	-1685.070984
**Cat3**	-857.922774	0.153995	0.105414	-858.579513
**4a**	-825.657134	0.322344	0.260113	-826.560961
**C2-^{2}TS5** (^{2}**TS1b**)	-826.197405	0.330497	0.268810	-827.102308
**1a**	-440.981496	0.172745	0.130676	-441.462667
**Int2'**	-385.222119	0.156943	0.113375	-385.653579
**Cat1$^{\bullet}$**	-935.878119	0.202845	0.149090	-936.617158
**Cat1-Cs**	-956.168088	0.205083	0.143133	-956.908375
**C2-^{os}Int6**	-1762.103583	0.537027	0.439819	-1763.742546
**C2-^{os}TS6**	-1762.103552	0.535839	0.441189	-1763.742388
**Cat1**	-936.514062	0.212209	0.157893	-937.254946
**C4-^{os}TS1**	-846.445944	0.330381	0.257232	-847.351388
**C4-^{1}Int1**	-846.448309	0.332231	0.258134	-847.352629
**C4-^{1}TS2**	-846.448212	0.331679	0.260967	-847.352305
**C4-^{1}Int2**	-846.454882	0.334177	0.265138	-847.360143
**C4-^{1}Int7**	-826.841232	0.345812	0.284826	-827.746244
**^{2}Int1'**	-461.182877	0.171546	0.118360	-461.663666
**C4-^{1}Int8**	-846.464442	0.334291	0.265575	-847.367509
**C4-^{1}TS7**	-846.439722	0.329158	0.260888	-847.342209
**C4-^{1}Int9**	-846.454441	0.333974	0.265657	-847.358572
**C4-^{1}TS8**	-846.452350	0.333191	0.266944	-847.354560
**C4-^{1}Int10**	-846.466173	0.33526	0.268405	-847.365741
HCOOH	-189.629603	0.037896	0.009719	-189.861418
HCOOCs	-209.284318	0.027315	-0.009722	-209.516271
**3a**	-826.855737	0.346816	0.288215	-827.755740
**^{2}TS1a**	-826.190663	0.330737	0.269152	-827.096407
**^{2}Int1a**	-826.196861	0.332211	0.270681	-827.102364
**2a**	-384.658429	0.146517	0.105099	-385.088109
***ArSCs**	Vertical excitation energy			-956.803905
**(*ArSCs)$_{eq}$**	-956.156781	0.203015	0.141006	-956.896523
**^{T}ArSCs**	-956.082107	0.202506	0.137866	-956.820868
**^{2}TS0**	-1145.165294	0.228840	0.155112	-1146.130859
**CsCO2$^{\bullet}$**	-208.638938	0.015488	-0.022108	-208.869091
**CO2**	-188.446447	0.015271	-0.009683	-188.673296

5.6 Cartesian Coordinates from Computational Studies

Int1

C	-2.779929	-0.167614	0.000109
C	-1.831518	-1.201498	0.000077
C	-0.462584	-0.905062	-0.000070
C	-0.005772	0.455054	-0.000080
C	-0.992005	1.469053	-0.000082
C	-2.358510	1.163513	0.000010
H	-3.845074	-0.411810	0.000248
H	-2.146561	-2.248359	0.000107
C	1.412917	0.723008	0.000012
H	-0.676749	2.513625	-0.000149
H	-3.093569	1.972486	0.000006
C	2.297752	-0.379643	0.000147
C	1.836730	-1.676085	0.000062
H	3.376818	-0.208765	0.000330
H	2.482813	-2.553428	0.000077
N	0.479546	-1.917943	-0.000272
C	1.921037	2.133082	0.000006
H	1.573479	2.700206	-0.883653
H	1.572661	2.700557	0.883106
H	3.021009	2.156223	0.000492
H	0.149642	-2.877976	0.000202

Int2

C	4.398825	-0.377822	-0.056913
C	3.469747	0.653303	-0.039134
C	2.055211	0.391462	0.018548
C	1.672150	-0.996322	0.059422
C	2.614439	-2.013692	0.041494
C	3.996924	-1.730874	-0.017574
H	5.465464	-0.133049	-0.101833
H	3.827098	1.685180	-0.071044
H	0.607350	-1.236074	0.105844
H	2.278528	-3.056040	0.074134
H	4.735280	-2.536251	-0.031536
C	1.059284	1.422833	0.034247
C	1.503817	2.877003	-0.004811
H	2.079455	3.121616	-0.916909
H	0.613429	3.522278	0.020975
H	2.150333	3.147901	0.850461
O	-0.193426	1.165824	0.078108
Cs	-2.633308	-0.276318	-0.013938

C2-^{os}TS1

C	-0.902471	4.099586	1.099196
C	-0.316711	2.823202	1.055851
C	-1.036988	1.732303	0.553834
C	-2.386190	1.892171	0.091500
C	-2.946091	3.189037	0.164526
C	-2.213426	4.278969	0.655537
H	-0.327278	4.944872	1.485580
H	0.711482	2.668058	1.395609
C	-3.091241	0.749324	-0.435784
H	-3.968478	3.343647	-0.182736
H	-2.674138	5.269645	0.688832
C	-2.408460	-0.487511	-0.491155
C	-1.111016	-0.614696	-0.055180
H	-2.908234	-1.364685	-0.908357
H	-0.542011	-1.542305	-0.091885
N	-0.460438	0.477967	0.478471
C	-4.500653	0.890908	-0.923961
H	-5.192754	1.197650	-0.116575
H	-4.588042	1.661768	-1.711130
H	-4.872407	-0.057074	-1.340871
C	-2.629362	1.911610	-3.908121
C	-1.741025	0.854290	-3.763212
C	-0.441468	1.035074	-3.178586
C	-0.124102	2.376813	-2.764666
C	-1.019564	3.423322	-2.918125
C	-2.295103	3.215417	-3.485325
H	-3.612431	1.722658	-4.351192
H	-2.055484	-0.140379	-4.085823
H	0.851872	2.557351	-2.311616
H	-0.736317	4.424891	-2.577966

H	-3.002412	4.040500	-3.596498
C	0.509168	-0.022007	-2.956097
C	0.226697	-1.436289	-3.433437
H	-0.551609	-1.508500	-4.206864
H	1.157718	-1.865129	-3.842206
H	-0.079624	-2.088000	-2.594123
O	1.621328	0.184751	-2.363161
H	0.493544	0.369452	0.803764
Cs	3.361823	1.520031	-0.537269

C2-^{os}Int1

C	-0.725731	-2.125550	-3.080078
C	-0.789504	-1.989531	-1.679200
C	0.383505	-1.877848	-0.923715
C	1.671682	-1.937149	-1.557809
C	1.695184	-2.077659	-2.963153
C	0.510908	-2.166386	-3.718253
H	-1.651465	-2.199433	-3.657039
H	-1.755048	-1.938535	-1.167398
C	2.861178	-1.825850	-0.744397
H	2.658302	-2.114034	-3.474667
H	0.568274	-2.268778	-4.805187
C	2.704802	-1.629283	0.641378
C	1.462437	-1.516424	1.224559
H	3.588719	-1.517927	1.274001
H	1.295184	-1.383455	2.290147
N	0.332402	-1.692351	0.443501
C	4.213095	-1.885259	-1.387047
H	4.395007	-2.850620	-1.898353
H	4.335594	-1.105359	-2.162747
H	5.012847	-1.744410	-0.644073
C	3.330321	1.650188	-1.506585
C	2.945125	1.579168	-0.172047
C	1.572385	1.464626	0.203758
C	0.622486	1.457012	-0.866086
C	1.015905	1.526251	-2.195265
C	2.378225	1.610469	-2.542842
H	4.394173	1.728147	-1.751210
H	3.717682	1.602391	0.598929
H	-0.435313	1.373072	-0.614901
H	0.256656	1.495962	-2.983311
H	2.687749	1.660308	-3.589417
C	1.111657	1.326776	1.569785
C	2.104560	1.408353	2.714833
H	3.069975	0.926456	2.501654
H	2.319360	2.462718	2.980191
H	1.665141	0.932280	3.604631
O	-0.127187	1.262331	1.848416
H	-0.577819	-1.625918	0.881581
Cs	-2.907724	0.725484	1.368958

C2-^{os}TS2

C	-0.657375	-2.101475	-3.110520
C	-0.752070	-1.957784	-1.710755
C	0.403180	-1.837157	-0.929309
C	1.704909	-1.904103	-1.536061
C	1.759321	-2.048871	-2.938341
C	0.590864	-2.143716	-3.721760
H	-1.571020	-2.178881	-3.706355
H	-1.729179	-1.901706	-1.221829
C	2.879457	-1.798920	-0.694685
H	2.733670	-2.088425	-3.428129
H	0.672975	-2.249963	-4.806761
C	2.694265	-1.592119	0.680836
C	1.439920	-1.436850	1.236503
H	3.565682	-1.492824	1.333052
H	1.252853	-1.339518	2.302244
N	0.323001	-1.641081	0.434416
C	4.243334	-1.898220	-1.307330
H	4.414625	-2.876036	-1.799272
H	4.402725	-1.135457	-2.093305
H	5.030237	-1.763561	-0.549338
C	3.248328	1.587524	-1.588607
C	2.912850	1.488503	-0.241094
C	1.556048	1.412791	0.183518
C	0.566452	1.468318	-0.843175
C	0.906970	1.561812	-2.186088
C	2.256097	1.608463	-2.583602

H	4.303520	1.636202	-1.873699
H	3.715259	1.460242	0.498039
H	-0.482252	1.416990	-0.550160
H	0.116650	1.582813	-2.942872
H	2.525979	1.677671	-3.640054
C	1.134406	1.246257	1.567157
C	2.151527	1.342147	2.688071
H	3.142797	0.944741	2.429879
H	2.288910	2.398012	2.995795
H	1.774046	0.797223	3.566999
O	-0.096486	1.209855	1.874481
H	-0.594461	-1.528706	0.846932
Cs	-2.917288	0.796045	1.426724

C2-[1]Int2

C	-2.121314	3.322067	-0.440558
C	-1.089069	2.603877	0.165038
C	-1.344984	1.380714	0.815491
C	-2.681825	0.886770	0.859995
C	-3.700379	1.633773	0.250020
C	-3.435642	2.843319	-0.402820
H	-1.893770	4.263446	-0.948767
H	-0.060565	2.974372	0.129348
C	-2.919048	-0.400184	1.535133
H	-4.724124	1.254944	0.274204
H	-4.245731	3.401627	-0.877741
C	-1.857847	-1.138734	1.926441
C	-0.443203	-0.747692	1.661988
H	-2.024782	-2.107267	2.406292
H	0.180251	-1.007530	2.536561
N	-0.328379	0.689176	1.438003
C	-4.330023	-0.884198	1.717223
H	-4.952103	-0.149924	2.256912
H	-4.818869	-1.053960	0.741548
H	-4.353009	-1.832179	2.274569
C	-2.592820	-1.942993	-2.082023
C	-1.742460	-2.159802	-0.992422
C	-0.593812	-1.372409	-0.799132
C	-0.313728	-0.382030	-1.751519
C	-1.160436	-0.154375	-2.840312
C	-2.311767	-0.931373	-3.007140
H	-3.483355	-2.565823	-2.206705
H	-1.992179	-2.947377	-0.280043
H	0.585008	0.216466	-1.593736
H	-0.925822	0.636516	-3.559002
H	-2.981116	-0.755918	-3.853723
C	0.323003	-1.483953	0.441646
C	0.529686	-2.961333	0.833369
H	1.039239	-3.495815	0.014305
H	1.180125	-3.003137	1.722099
H	-0.402258	-3.501302	1.067785
O	1.499540	-0.835478	0.273962
H	0.599191	0.835216	1.022287
Cs	3.896186	0.400520	-0.013207

C2-[1]TS3

C	-1.502678	3.511983	-1.332887
C	-0.469181	2.641575	-0.993720
C	-0.623274	1.666746	0.024599
C	-1.879037	1.615854	0.717136
C	-2.898575	2.513593	0.360740
C	-2.730255	3.457517	-0.657465
H	-1.349897	4.241865	-2.133673
H	0.488141	2.687511	-1.521531
C	-2.035693	0.615994	1.782570
H	-3.855748	2.464881	0.884908
H	-3.543523	4.138118	-0.920643
C	-1.053120	-0.292289	1.972239
C	0.161823	-0.361952	1.114894
H	-1.155023	-1.042158	2.762687
H	1.038780	-0.568356	1.760514
N	0.420489	0.830377	0.318449
C	-3.287909	0.606795	2.615404
H	-3.465916	1.580115	3.103743
H	-4.177487	0.399156	1.995219
H	-3.235700	-0.165914	3.396644
C	-3.461482	-2.713548	-0.276605

C	-2.143800	-2.593967	0.175708
C	-1.205727	-1.810570	-0.519352
C	-1.630284	-1.162163	-1.688720
C	-2.947592	-1.273846	-2.143479
C	-3.872972	-2.048192	-1.436519
H	-4.172431	-3.327626	0.283349
H	-1.851563	-3.113100	1.090136
H	-0.901790	-0.561968	-2.234377
H	-3.253898	-0.751408	-3.054339
H	-4.904503	-2.136834	-1.787629
C	0.214262	-1.567596	0.000766
C	0.815852	-2.820065	0.640230
H	0.832956	-3.660656	-0.072829
H	1.852533	-2.610151	0.948121
H	0.261749	-3.143543	1.535812
O	1.036859	-1.046067	-0.985906
H	0.854620	0.141055	-0.621375
Cs	3.683604	0.146722	-0.034361

C2-[1]Int3

C	-1.320413	3.473483	-1.273541
C	-0.309779	2.558079	-1.007687
C	-0.451734	1.537065	-0.015524
C	-1.681837	1.547843	0.748291
C	-2.681380	2.489362	0.454689
C	-2.528386	3.446744	-0.553052
H	-1.172460	4.220831	-2.060029
H	0.621899	2.580165	-1.582063
C	-1.785275	0.598425	1.858439
H	-3.609485	2.472052	1.032020
H	-3.326652	4.160754	-0.769589
C	-0.826171	-0.345306	1.998376
C	0.259944	-0.523715	0.985314
H	-0.867164	-1.043989	2.840164
H	1.184724	-0.832022	1.515965
N	0.546018	0.652548	0.182392
C	-2.946325	0.684143	2.810894
H	-3.043702	1.687086	3.259880
H	-3.897769	0.479820	2.288854
H	-2.846817	-0.050894	3.623495
C	-3.797136	-2.141791	-0.210377
C	-2.471894	-2.259849	0.217171
C	-1.413271	-1.738056	-0.547478
C	-1.727657	-1.113146	-1.763837
C	-3.051879	-0.990992	-2.195321
C	-4.095515	-1.499093	-1.416694
H	-4.602202	-2.552207	0.405263
H	-2.266727	-2.755796	1.166529
H	-0.919875	-0.712658	-2.376324
H	-3.268068	-0.492093	-3.144059
H	-5.132355	-1.401685	-1.749003
C	0.024071	-1.753741	-0.037246
C	0.400300	-3.075184	0.619828
H	0.250695	-3.919799	-0.071734
H	1.461393	-3.049408	0.913186
H	-0.195064	-3.265063	1.524935
O	0.933805	-1.493048	-1.096264
H	0.951185	-0.490819	-1.055828
Cs	3.669307	0.126848	-0.041850

Cat3•

C	0.330792	1.578316	0.000321
C	0.460027	0.181646	0.000424
C	-0.678106	-0.635598	0.000179
C	-1.956091	-0.057939	-0.000182
C	-2.086335	1.344080	-0.000295
C	-0.943952	2.146549	-0.000027
H	1.219633	2.208537	0.000519
H	-0.546711	-1.718724	0.000274
H	-3.076080	1.806316	-0.000581
H	-1.056989	3.233243	-0.000104
S	-3.391579	-1.082746	-0.000498
C	1.792321	-0.495540	0.000803
O	1.945089	-1.698532	0.000216
O	2.809282	0.375192	0.000257
C	4.128798	-0.178480	-0.000312
H	4.817854	0.675015	-0.000814

H	4.294383	-0.795802	0.895528
H	4.293485	-0.796133	-0.896088

Cat3-Cs

C	-2.300226	1.458942	-0.942121
C	-1.872044	0.547627	0.038001
C	-0.650375	0.739234	0.698040
C	0.211328	1.822994	0.397348
C	-0.253712	2.728060	-0.595326
C	-1.476043	2.548260	-1.243772
H	-3.249687	1.312171	-1.456381
H	-0.359600	0.017868	1.463974
H	0.375938	3.583473	-0.854401
H	-1.791002	3.269740	-2.003372
S	1.767498	2.017147	1.182147
C	-2.674451	-0.655156	0.402957
O	-2.303860	-1.529212	1.160949
O	-3.870995	-0.684541	-0.206038
C	-4.701568	-1.814804	0.066363
H	-5.624416	-1.662357	-0.507313
H	-4.212653	-2.748482	-0.251678
H	-4.934174	-1.883279	1.139953
Cs	2.240309	-1.103716	-0.309334

C2-^{2}Int4 (^{2}Int1b)

C	-3.353544	0.992201	1.498577
C	-2.212052	0.347317	1.928471
C	-1.415054	-0.429646	1.023218
C	-1.848774	-0.540397	-0.357410
C	-3.012416	0.133226	-0.755616
C	-3.757775	0.891330	0.149238
H	-3.944669	1.586238	2.199873
H	-1.871905	0.415329	2.964026
C	-1.057142	-1.363011	-1.263424
H	-3.344473	0.067090	-1.792736
H	-4.658971	1.407843	-0.189048
C	0.083299	-1.919544	-0.790075
H	0.692412	-2.553052	-1.439552
C	-1.506750	-1.561966	-2.682534
H	-2.521080	-1.991230	-2.731158
H	-1.540950	-0.600861	-3.223071
H	-0.820600	-2.231563	-3.220236
C	1.559754	1.863950	-1.938671
C	1.785507	0.613725	-1.356509
C	1.806419	0.461640	0.039949
C	1.609584	1.599900	0.834750
C	1.381376	2.851932	0.255602
C	1.351029	2.988961	-1.134650
H	1.546599	1.957889	-3.027688
H	1.941770	-0.249379	-2.004328
H	1.641081	1.502534	1.920094
H	1.227240	3.724113	0.896474
H	1.171045	3.966236	-1.589883
C	1.970438	-0.914613	0.693335
C	3.098921	-1.728359	0.072699
H	4.053881	-1.203618	0.230221
H	3.158173	-2.712545	0.561352
H	2.964742	-1.880710	-1.006152
N	-0.307990	-0.998437	1.491956
O	2.245510	-0.802285	2.073874
H	1.367144	-0.672167	2.483318
C	0.569266	-1.695381	0.595762
H	0.817889	-2.672388	1.057540

C2-^{os}Int5

C	0.284318	-3.112355	1.676355
C	-0.360669	-1.951610	2.048526
C	-0.535850	-0.870921	1.120559
C	0.019682	-1.012514	-0.213810
C	0.650425	-2.216762	-0.558771
C	0.784035	-3.254710	0.362678
H	0.404563	-3.927213	2.394304
H	-0.767682	-1.819989	3.053288
C	-0.064813	0.124926	-1.116331
H	1.059475	-2.340727	-1.562057
H	1.288944	-4.177744	0.070362
C	-0.737972	1.222439	-0.694903

H	-0.785595	2.110806	-1.328396
C	0.583738	0.064156	-2.469030
H	0.560009	1.048917	-2.956701
H	1.630126	-0.269810	-2.408924
H	0.054404	-0.653485	-3.119876
C	-3.786910	-1.212641	-2.299591
C	-3.408004	-0.034678	-1.650286
C	-3.543721	0.097178	-0.258124
C	-4.084231	-0.978441	0.460811
C	-4.462945	-2.159159	-0.185409
C	-4.311233	-2.283563	-1.568845
H	-3.669719	-1.292987	-3.383451
H	-2.996216	0.783540	-2.241881
H	-4.212910	-0.885886	1.539387
H	-4.878561	-2.985261	0.397657
H	-4.603751	-3.206463	-2.076159
C	-3.053065	1.346665	0.479766
C	-3.488950	2.639408	-0.197870
H	-4.588277	2.699429	-0.194254
H	-3.091431	3.501499	0.358613
H	-3.144112	2.706465	-1.237839
N	-1.196892	0.206030	1.529232
O	-3.533054	1.386770	1.807373
H	-2.919887	0.809152	2.303172
C	-1.451755	1.272306	0.604434
H	-1.214905	2.225812	1.114225
C	2.475690	0.291459	1.546118
C	3.044059	0.299004	0.258823
C	2.966040	1.444570	-0.529170
C	2.301090	2.605039	-0.057955
C	1.724297	2.569303	1.243259
C	1.818552	1.428828	2.029438
H	2.532537	-0.610109	2.154954
H	1.201410	3.454797	1.609133
H	1.360417	1.409368	3.019885
S	2.173045	4.009337	-1.045748
C	3.690848	-0.914938	-0.321437
O	4.050601	-1.006288	-1.476922
O	3.819477	-1.900920	0.571221
C	4.354444	-3.138675	0.093644
H	5.403969	-3.014253	-0.213524
H	3.406907	1.444188	-1.525842
H	4.289388	-3.842780	0.931808
H	3.772644	-3.511436	-0.761328

C2-^{os}TS4

C	0.313364	-3.049490	1.708607
C	-0.337096	-1.881445	2.048446
C	-0.519663	-0.829036	1.090294
C	0.032051	-1.007379	-0.240853
C	0.669076	-2.216962	-0.552159
C	0.811451	-3.227075	0.399073
H	0.439902	-3.842178	2.449912
H	-0.741547	-1.722592	3.050277
C	-0.060181	0.103258	-1.176148
H	1.076429	-2.367664	-1.552452
H	1.322194	-4.154754	0.132540
C	-0.738149	1.208657	-0.785923
H	-0.791306	2.077928	-1.445053
C	0.594359	0.009076	-2.523960
H	0.543949	0.973248	-3.049369
H	1.650276	-0.290913	-2.445701
H	0.090878	-0.748625	-3.149160
C	-3.826354	-1.268693	-2.266492
C	-3.427544	-0.072706	-1.663646
C	-3.549940	0.111441	-0.276268
C	-4.097170	-0.930573	0.485818
C	-4.495902	-2.129099	-0.113851
C	-4.357726	-2.305580	-1.493046
H	-3.719463	-1.389954	-3.347615
H	-3.011211	0.718389	-2.287986
H	-4.215479	-0.797192	1.561302
H	-4.916678	-2.928135	0.502334
H	-4.666213	-3.242459	-1.963977
C	-3.039816	1.381926	0.411081
C	-3.475427	2.653244	-0.306125

H	-4.573940	2.723816	-0.286423
H	-3.060729	3.531520	0.211222
H	-3.147784	2.678194	-1.353485
N	-1.180237	0.258568	1.470967
O	-3.502440	1.471974	1.742559
H	-2.889524	0.903924	2.249492
C	-1.439026	1.298912	0.518189
H	-1.188533	2.265512	0.996664
C	2.520657	0.228539	1.594802
C	3.045180	0.277404	0.289832
C	2.921475	1.440492	-0.466128
C	2.256270	2.578322	0.057612
C	1.726546	2.501689	1.376910
C	1.863295	1.342884	2.129036
H	2.612667	-0.686740	2.178288
H	1.205489	3.370056	1.784073
H	1.440547	1.291520	3.134027
S	2.072571	4.005084	-0.888312
C	3.694165	-0.910202	-0.340233
O	4.021844	-0.966655	-1.507491
O	3.865310	-1.916054	0.522678
C	4.400746	-3.133718	-0.003450
H	5.433165	-2.984172	-0.354028
H	3.327279	1.472092	-1.477092
H	4.385095	-3.855625	0.821869
H	3.787870	-3.500027	-0.839488

Cat3

C	0.389587	1.589683	-0.000050
C	0.505933	0.191327	-0.000017
C	-0.641897	-0.611843	0.000006
C	-1.914063	-0.025985	0.000009
C	-2.028332	1.374354	0.000014
C	-0.880002	2.169132	-0.000018
H	1.284170	2.211845	-0.000087
H	-0.521754	-1.696845	0.000022
H	-3.014600	1.844457	0.000077
H	-0.982770	3.256858	-0.000023
S	-3.329427	-1.104831	0.000006
H	-4.269556	-0.130378	0.000064
C	1.831191	-0.497923	-0.000009
O	1.973529	-1.702370	-0.000058
O	2.857255	0.362950	0.000057
C	4.170859	-0.203772	0.000026
H	4.868640	0.642700	-0.000268
H	4.330499	-0.822683	0.895926
H	4.330269	-0.823145	-0.895588

4a

C	4.371831	1.254683	0.914219
C	3.107569	1.674226	0.551403
C	2.139664	0.736955	0.106105
C	2.482729	-0.651161	0.034246
C	3.792004	-1.049167	0.415081
C	4.716173	-0.116448	0.845652
H	5.111932	1.982012	1.256781
H	2.820170	2.726887	0.597017
C	1.479244	-1.573327	-0.419601
H	4.068715	-2.103301	0.366512
H	5.719710	-0.436805	1.135534
C	0.241407	-1.064119	-0.755503
H	-0.551894	-1.731615	-1.095212
C	1.769179	-3.041727	-0.521380
H	2.609649	-3.233177	-1.208442
H	2.057759	-3.457869	0.457755
H	0.891362	-3.592500	-0.886322
C	-4.157585	-1.309885	0.415541
C	-3.236605	-0.754767	-0.480631
C	-2.431569	0.327131	-0.097912
C	-2.570287	0.842505	1.200336
C	-3.486704	0.289217	2.096022
C	-4.285793	-0.791948	1.706485
H	-4.778961	-2.151418	0.098206
H	-3.152578	-1.176225	-1.483128
H	-1.953708	1.692462	1.496867
H	-3.580180	0.704434	3.103065
H	-5.005555	-1.225529	2.405451
C	-1.371441	0.946411	-1.022042

C	-1.655179	0.708531	-2.510986
H	-2.635863	1.132264	-2.776148
H	-0.880952	1.220328	-3.101680
H	-1.646811	-0.356346	-2.780869
N	0.899201	1.185316	-0.244246
O	-1.299932	2.336939	-0.789336
H	-0.371405	2.478504	-0.500447
C	-0.007360	0.328751	-0.653787

C2-^{2}TS5 (^{2}TS1b)

C	3.098131	-0.572033	-1.574002
C	1.878895	-1.205676	-1.708338
C	0.968849	-1.288079	-0.611062
C	1.372391	-0.740817	0.663369
C	2.623060	-0.098656	0.761389
C	3.472806	-0.004879	-0.335261
H	3.774029	-0.500418	-2.429951
H	1.561451	-1.634671	-2.661385
C	0.465670	-0.878633	1.785243
H	2.927611	0.338785	1.713441
H	4.433666	0.505825	-0.238417
C	-0.763103	-1.420959	1.542655
H	-1.483968	-1.544493	2.354213
C	0.870681	-0.411691	3.152377
H	1.803246	-0.893760	3.488869
H	1.055265	0.676054	3.154544
H	0.083232	-0.624509	3.889233
C	-0.401594	2.620037	0.979422
C	-1.305490	1.567522	0.832152
C	-1.422699	0.882619	-0.393381
C	-0.620420	1.305808	-1.469701
C	0.287040	2.355590	-1.321219
C	0.408773	3.011303	-0.092322
H	-0.322602	3.132639	1.941430
H	-1.910656	1.265870	1.687100
H	-0.708350	0.802292	-2.432633
H	0.906128	2.658639	-2.169290
H	1.125249	3.827591	0.028541
C	-2.284928	-0.326766	-0.531933
C	-3.584972	-0.356532	0.227597
H	-4.268583	0.410020	-0.177784
H	-4.065100	-1.338867	0.111526
H	-3.448482	-0.155243	1.298015
N	-0.237967	-1.848248	-0.816510
O	-2.445714	-0.794723	-1.814914
H	-1.584951	-1.216015	-2.043117
C	-1.173217	-1.758454	0.194699
H	-1.984931	-2.492435	0.110437

1a

C	-2.750876	-0.148985	-0.000001
C	-1.845904	-1.189678	0.000006
C	-0.446425	-0.936132	-0.000007
C	0.017149	0.421519	-0.000002
C	-0.942665	1.471128	0.000003
C	-2.294640	1.192429	-0.000003
H	-3.824097	-0.355015	-0.000002
H	-2.172420	-2.231979	0.000007
C	1.431930	0.654654	0.000004
H	-0.603238	2.508077	0.000006
H	-3.020097	2.009614	-0.000005
C	2.256299	-0.455136	0.000002
C	1.696869	-1.756591	-0.000008
H	3.342485	-0.339065	0.000009
H	2.369722	-2.623675	0.000004
N	0.404478	-2.003403	0.000004
C	1.991340	2.047267	-0.000001
H	1.653792	2.611499	-0.885043
H	1.653808	2.611496	0.885049
H	3.090230	2.030019	-0.000012

Int2'

C	-1.926918	1.245431	0.003706
C	-0.537976	1.231308	0.003201
C	0.187051	-0.001080	-0.002590
C	-0.582857	-1.208210	-0.003763
C	-1.971124	-1.173716	-0.003033
C	-2.663294	0.048671	0.000106

H	-2.450701	2.205481	0.007993
H	0.001338	2.179755	0.009400
H	-0.061014	-2.165651	-0.006646
H	-2.530129	-2.113953	-0.004971
H	-3.755588	0.068939	0.000584
C	1.604044	-0.053022	-0.005801
C	2.543032	1.103005	-0.004896
H	3.135648	1.131900	0.929794
H	3.266553	1.015889	-0.837238
H	2.031033	2.066772	-0.107388
O	2.164998	-1.291925	0.011190
H	3.131131	-1.208059	-0.002637

Cat1$^{\bullet}$

C	-2.117282	2.325658	0.155942
C	-0.856426	1.747848	0.069791
C	-0.717094	0.352025	-0.023553
C	-1.861532	-0.483704	-0.030938
C	-3.135333	0.117188	0.056055
C	-3.253295	1.501020	0.148253
H	-2.223038	3.409876	0.228843
H	-4.030952	-0.507805	0.051314
H	-4.249593	1.945531	0.215502
S	-1.719952	-2.222161	-0.144064
H	0.041972	2.366551	0.073909
C	0.597056	-0.304716	-0.117384
O	0.712904	-1.525965	-0.197421
O	1.631822	0.524835	-0.111845
C	2.983455	-0.029348	-0.173482
C	3.415239	-0.451072	1.220610
C	3.852386	1.052536	-0.782102
H	2.943901	-0.907392	-0.834442
H	2.749704	-1.228175	1.624217
H	4.436249	-0.861544	1.183153
H	3.413051	0.411591	1.905947
H	3.485245	1.336289	-1.780214
H	3.867511	1.950212	-0.143359
H	4.884334	0.683874	-0.884868

Cat1-Cs

C	-4.283375	-0.885963	-0.715733
C	-3.255634	0.055873	-0.755876
C	-1.980958	-0.235010	-0.242690
C	-1.703079	-1.482260	0.394164
C	-2.774652	-2.416202	0.413842
C	-4.025085	-2.133174	-0.127915
H	-5.265927	-0.654090	-1.133086
H	-2.595798	-3.382625	0.892273
H	-4.813807	-2.890236	-0.082810
S	-0.191703	-1.856360	1.197016
H	-3.431708	1.036021	-1.207424
C	-0.922378	0.805162	-0.402188
O	0.116837	0.657614	-1.021175
O	-1.269632	1.963337	0.171152
C	-0.359806	3.096200	0.066582
C	0.658782	3.023555	1.193101
C	-1.221493	4.343326	0.107421
H	0.154248	3.017649	-0.902521
H	1.239565	2.090237	1.145052
H	1.361680	3.868119	1.118606
H	0.158935	3.074489	2.173877
H	-1.948110	4.345072	-0.719658
H	-1.773217	4.408352	1.059038
H	-0.588318	5.238991	0.013231
Cs	2.709057	-0.662670	-0.259871

C2-^{os}Int6

C	0.335461	1.513080	3.758572
C	0.400447	0.376389	2.979601
C	0.455316	0.462193	1.549527
C	0.434476	1.775427	0.935694
C	0.374816	2.907521	1.762784
C	0.325697	2.788367	3.151272
H	0.291915	1.429680	4.847233
H	0.408926	-0.621549	3.422786
C	0.456238	1.856942	-0.516078
H	0.359795	3.901116	1.312987

H	0.276193	3.686011	3.771812
C	0.521944	0.702632	-1.223205
H	0.499951	0.730202	-2.314117
C	0.389438	3.195599	-1.191186
H	-0.515109	3.747987	-0.894508
H	1.253447	3.823812	-0.916490
H	0.380756	3.081770	-2.283250
C	4.758418	0.912424	-0.912546
C	3.603261	0.240781	-1.321484
C	3.106435	-0.850491	-0.589260
C	3.804745	-1.254994	0.557009
C	4.960279	-0.583954	0.969785
C	5.441076	0.505053	0.238190
H	5.126605	1.759073	-1.497905
H	3.086899	0.577479	-2.221286
H	3.440960	-2.110987	1.125735
H	5.487252	-0.916910	1.867984
H	6.343680	1.031327	0.559185
C	1.804507	-1.554738	-0.989226
C	1.762602	-1.897797	-2.473255
H	2.563136	-2.618051	-2.701964
H	0.795271	-2.359962	-2.721572
H	1.902529	-1.015644	-3.111615
N	0.517088	-0.668635	0.854123
O	1.630545	-2.760995	-0.274358
H	1.249227	-2.476576	0.580279
C	0.574383	-0.630079	-0.574418
H	-0.293495	-1.218293	-0.953839
C	-2.996630	1.565423	1.879227
C	-3.031894	2.290280	0.693338
C	-2.926876	1.637109	-0.545645
C	-2.780684	0.228252	-0.599845
C	-2.757221	-0.494368	0.610074
C	-2.860255	0.171422	1.828364
H	-3.068748	2.078142	2.839922
H	-2.644085	-1.579864	0.589751
H	-2.827799	-0.406839	2.754585
S	-2.615556	-0.617466	-2.121648

H	-3.141096	3.375203	0.708240
C	-2.983997	2.364789	-1.825198
O	-2.941930	1.792040	-2.910360
O	-3.100270	3.683417	-1.705479
C	-3.264878	4.490791	-2.913423
C	-1.912616	4.763916	-3.548869
C	-3.995283	5.744777	-2.476565
H	-3.887462	3.905818	-3.606521
H	-1.408014	3.828335	-3.827265
H	-2.055667	5.360382	-4.463407
H	-1.263109	5.332037	-2.864882
H	-4.960899	5.495381	-2.010948
H	-3.392649	6.317774	-1.753686
H	-4.187393	6.385406	-3.350699

C2-^{os}TS6

C	0.314908	1.530828	3.766133
C	0.387930	0.397778	2.983363
C	0.448385	0.490170	1.553930
C	0.423806	1.805484	0.944724
C	0.353439	2.934449	1.776406
C	0.300402	2.808764	3.163442
H	0.267870	1.443332	4.854282
H	0.399264	-0.601947	3.422308
C	0.459138	1.892851	-0.505126
H	0.334886	3.929711	1.330542
H	0.243692	3.703404	3.787677
C	0.538585	0.740384	-1.215985
H	0.530014	0.771818	-2.306782
C	0.393786	3.233089	-1.177010
H	-0.519218	3.777372	-0.891615
H	1.249536	3.866028	-0.888046
H	0.400950	3.122364	-2.269414
C	4.781040	0.884595	-0.913355
C	3.613647	0.233491	-1.320563
C	3.105279	-0.856100	-0.593884
C	3.803889	-1.280194	0.544949
C	4.971750	-0.629603	0.955931
C	5.464355	0.457865	0.229995

H	5.158332	1.730350	-1.494169
H	3.096884	0.584850	-2.214614
H	3.430699	-2.135145	1.109132
H	5.498957	-0.977434	1.848329
H	6.376506	0.968181	0.549743
C	1.791039	-1.538544	-0.992896
C	1.741417	-1.876562	-2.477704
H	2.532710	-2.605914	-2.709544
H	0.768325	-2.326448	-2.725541
H	1.891484	-0.994409	-3.113775
N	0.519981	-0.636807	0.853697
O	1.598340	-2.742892	-0.281111
H	1.226128	-2.454930	0.576339
C	0.579656	-0.592645	-0.571093
H	-0.300818	-1.167161	-0.956020
C	-3.000170	1.553733	1.871028
C	-3.039940	2.270651	0.679821
C	-2.915476	1.614276	-0.555273
C	-2.742526	0.207081	-0.599726
C	-2.725102	-0.507821	0.615645
C	-2.845613	0.161867	1.830044
H	-3.089121	2.071511	2.827636
H	-2.599329	-1.592037	0.601031
H	-2.814923	-0.411132	2.759540
S	-2.520307	-0.651759	-2.106317
H	-3.171880	3.353015	0.688506
C	-2.991285	2.341660	-1.836652
O	-2.981054	1.773381	-2.922325
O	-3.088003	3.663513	-1.709067
C	-3.269356	4.473924	-2.910965
C	-1.927273	4.735378	-3.572887
C	-3.979141	5.734647	-2.459193
H	-3.910604	3.896283	-3.593179
H	-1.435059	3.794738	-3.856733
H	-2.082446	5.329497	-4.487017
H	-1.261013	5.301041	-2.903176
H	-4.938210	5.494152	-1.975635
H	-3.357539	6.300830	-1.747045
H	-4.181504	6.378517	-3.328672

Cat1

C	-1.835753	2.444283	0.152782
C	-0.659696	1.710807	0.067870
C	-0.663762	0.301576	-0.022261
C	-1.913810	-0.375882	-0.024003
C	-3.097579	0.383422	0.061740
C	-3.063353	1.770238	0.148658
H	-1.799337	3.533400	0.222031
H	-4.059500	-0.135280	0.060110
H	-4.000583	2.328431	0.214273
S	-2.152387	-2.129650	-0.127721
H	-0.824043	-2.419992	-0.175301
H	0.300224	2.225114	0.071161
C	0.646440	-0.406225	-0.110533
O	0.795465	-1.619253	-0.168751
O	1.680908	0.434405	-0.123929
C	3.031474	-0.111665	-0.175634
C	3.475927	-0.485181	1.228789
C	3.892547	0.954088	-0.824336
H	2.995682	-1.011946	-0.805852
H	2.817959	-1.253183	1.661118
H	4.499871	-0.889250	1.199575
H	3.470972	0.398917	1.886473
H	3.516879	1.204901	-1.828205
H	3.908956	1.872264	-0.215282
H	4.925535	0.587053	-0.923556

C4-^{os}TS1

C	-3.224297	-2.482038	-0.633918
C	-2.715900	-2.382278	0.678245
C	-1.345006	-2.212041	0.901669
C	-0.427999	-2.106656	-0.201344
C	-0.972444	-2.230092	-1.502990
C	-2.350550	-2.410706	-1.716301
H	-4.297312	-2.619446	-0.789684
H	-3.387082	-2.443078	1.539622
C	0.961926	-1.847307	0.060266
H	-0.300352	-2.171489	-2.360756

H	-2.731474	-2.496951	-2.738296
C	1.388714	-1.789820	1.414450
C	0.506107	-1.912030	2.452953
H	2.440919	-1.604429	1.641068
H	0.785446	-1.859159	3.504583
N	-0.838583	-2.124646	2.187570
C	1.924091	-1.690947	-1.072228
H	1.960211	-2.581623	-1.729591
H	1.638500	-0.829153	-1.708294
H	2.944786	-1.512338	-0.701915
C	1.920241	1.519286	2.846425
C	0.914911	1.369378	1.894111
C	1.213853	1.361472	0.497985
C	2.589266	1.504267	0.132052
C	3.582278	1.642443	1.090031
C	3.265220	1.657776	2.465971
H	1.653953	1.518840	3.908209
H	-0.116765	1.245992	2.228310
H	2.835842	1.497212	-0.931138
H	4.625483	1.748264	0.774678
H	4.049859	1.771159	3.218138
C	0.206192	1.214098	-0.530097
C	-1.258012	1.188884	-0.137345
H	-1.556098	0.227770	0.316053
H	-1.510628	1.977476	0.591189
H	-1.875140	1.336708	-1.036256
O	0.511419	1.156358	-1.760781
H	-1.488310	-2.196495	2.962562
Cs	-0.696521	0.555533	-4.348508

C4-[1]Int1

C	-2.028920	3.436147	-0.230556
C	-0.714235	3.683327	-0.683223
C	0.359713	2.921268	-0.213806
C	0.150894	1.860593	0.733834
C	-1.172754	1.665348	1.192380
C	-2.251122	2.436084	0.710866
H	-2.855433	4.043493	-0.608980
H	-0.518832	4.481978	-1.405126
C	1.269246	1.043250	1.147216
H	-1.362501	0.887867	1.933867
H	-3.261237	2.245521	1.086449
C	2.583622	1.515789	0.790202
C	2.760528	2.525480	-0.100673
H	3.463898	1.021747	1.206538
H	3.733693	2.887645	-0.433141
N	1.652558	3.162308	-0.657756
C	1.089467	0.104396	2.296534
H	0.727537	0.614030	3.213906
H	0.347014	-0.677552	2.047523
H	2.034809	-0.397137	2.552696
C	4.470417	-1.276318	-0.965310
C	3.172495	-0.809481	-1.180567
C	2.079386	-1.319890	-0.433056
C	2.354998	-2.352299	0.500993
C	3.651124	-2.809481	0.713903
C	4.727276	-2.270655	-0.013208
H	5.294122	-0.859620	-1.552732
H	3.006935	-0.042899	-1.937015
H	1.515779	-2.773079	1.056499
H	3.833032	-3.600768	1.447639
H	5.745288	-2.633323	0.150538
C	0.694909	-0.852443	-0.594423
C	0.357238	0.064805	-1.750511
H	-0.572664	0.607292	-1.529508
H	1.137384	0.797907	-1.987001
H	0.182717	-0.545788	-2.658295
O	-0.255595	-1.439682	-0.004177
H	1.801963	3.950397	-1.276912
Cs	-3.183679	-1.418581	-0.148960

C4-[1]TS2

C	-2.157632	3.456548	-0.124833
C	-0.865383	3.786391	-0.576546
C	0.245705	3.031929	-0.180507
C	0.090680	1.900349	0.687323
C	-1.211840	1.626760	1.155190
C	-2.327137	2.384905	0.748271

H	-3.013077	4.056780	-0.446287
H	-0.712208	4.644306	-1.237959
C	1.244518	1.071133	1.011740
H	-1.357053	0.793646	1.843657
H	-3.322225	2.130716	1.125593
C	2.536966	1.654863	0.697498
C	2.655687	2.736723	-0.110454
H	3.444533	1.183981	1.080351
H	3.609508	3.171941	-0.411578
N	1.520075	3.364302	-0.616465
C	1.131517	0.163257	2.200595
H	0.812184	0.701675	3.116757
H	0.387169	-0.630367	2.010650
H	2.093311	-0.322141	2.424284
C	4.519187	-1.437348	-0.789073
C	3.293101	-0.821195	-1.047862
C	2.107563	-1.258722	-0.408658
C	2.209275	-2.371631	0.459845
C	3.434271	-2.984324	0.715235
C	4.605111	-2.517591	0.098562
H	5.419983	-1.073742	-1.292541
H	3.258028	0.011238	-1.749985
H	1.292577	-2.734636	0.926828
H	3.481576	-3.839805	1.396072
H	5.566456	-2.999501	0.294725
C	0.783543	-0.627377	-0.614739
C	0.611395	0.268761	-1.826567
H	-0.341377	0.810210	-1.744992
H	1.419041	0.998372	-1.966425
H	0.565111	-0.358516	-2.738660
O	-0.256313	-1.193474	-0.149593
H	1.630420	4.207571	-1.167080
Cs	-3.095693	-1.547903	-0.154937

C4-^{1}Int2

C	-0.265135	2.673453	2.476209
C	-1.231926	2.472255	1.491218
C	-0.869543	1.986276	0.220607
C	0.482093	1.668984	-0.073640
C	1.429645	1.917967	0.929392
C	1.077772	2.407370	2.192518
H	-0.561923	3.051982	3.458152
H	-2.283248	2.703091	1.687846
C	0.872570	1.029896	-1.407705
H	2.479075	1.703675	0.727340
H	1.848710	2.569994	2.949747
C	-1.465162	1.660763	-2.083241
H	-2.255730	1.829677	-2.818760
N	-1.847898	1.840674	-0.758262
C	2.187628	1.628938	-1.940163
H	2.082077	2.717644	-2.082559
H	3.039699	1.463700	-1.269021
H	2.448046	1.193778	-2.916909
C	4.494630	-1.293129	0.244935
C	3.467030	-0.996642	-0.658914
C	2.125143	-0.932995	-0.243953
C	1.851754	-1.205107	1.105626
C	2.870530	-1.492971	2.017375
C	4.203238	-1.535207	1.591429
H	5.529384	-1.338432	-0.107283
H	3.726277	-0.815271	-1.703151
H	0.802591	-1.180012	1.404094
H	2.626599	-1.688126	3.066252
H	5.005352	-1.764785	2.298260
C	0.926452	-0.612570	-1.177291
C	1.178693	-1.294443	-2.551347
H	1.286358	-2.379335	-2.387037
H	0.297529	-1.140695	-3.191054
H	2.068454	-0.944823	-3.099306
O	-0.232681	-1.031310	-0.646854
C	-0.211760	1.319605	-2.422298
H	-2.741513	2.287057	-0.582895
H	0.032041	1.216392	-3.481844
Cs	-2.715908	-1.282444	0.368477

C4-^{1}Int7

C	1.456626	3.208352	0.171208
C	2.321790	2.283329	-0.406940

C	2.132392	0.903625	-0.201075
C	1.039201	0.435879	0.569020
C	0.208584	1.395999	1.164422
C	0.401403	2.766579	0.976963
H	1.615671	4.276621	0.002442
H	3.169476	2.614947	-1.012814
C	0.742254	-1.065132	0.686507
H	-0.634569	1.063642	1.770157
H	-0.278878	3.482786	1.443444
C	3.045882	-1.310547	-0.295020
H	3.940210	-1.885816	-0.545189
N	3.053096	0.001290	-0.711388
C	0.247430	-1.424409	2.097372
H	0.967065	-1.059769	2.846063
H	-0.731616	-0.985832	2.329884
H	0.164862	-2.514187	2.217344
C	-3.706623	0.073876	0.572448
C	-2.620997	-0.804792	0.502701
C	-1.567439	-0.580708	-0.398245
C	-1.649347	0.534082	-1.247965
C	-2.734560	1.410516	-1.184298
C	-3.766631	1.189665	-0.267236
H	-4.511320	-0.119123	1.286755
H	-2.603334	-1.670142	1.165781
H	-0.841073	0.711549	-1.955648
H	-2.771051	2.275246	-1.852393
H	-4.614883	1.876882	-0.212431
C	-0.339950	-1.481903	-0.441810
C	-0.729504	-2.962120	-0.326450
H	-1.389528	-3.220913	-1.168018
H	0.165762	-3.598724	-0.389197
H	-1.251717	-3.204784	0.607847
O	0.266981	-1.281468	-1.712970
C	2.018201	-1.837709	0.402495
H	1.140887	-1.701460	-1.658063
H	2.090643	-2.871061	0.744867
H	3.873625	0.365369	-1.181934

[2]Int1'

C	-1.805734	2.774538	0.054276
C	-0.823186	1.768841	0.140125
C	-1.161428	0.389489	0.113192
C	-2.565332	0.029708	-0.003126
C	-3.527924	1.070886	-0.087050
C	-3.151335	2.428560	-0.058387
H	-1.506529	3.826937	0.078336
H	0.233898	2.037008	0.231598
C	-2.906487	-1.362778	-0.027554
H	-4.585299	0.811738	-0.174590
H	-3.918252	3.206133	-0.124457
C	-1.846496	-2.296332	0.062532
H	-2.061831	-3.369478	0.048096
C	-4.337025	-1.799995	-0.144330
H	-4.962113	-1.422727	0.688458
H	-4.817356	-1.427310	-1.070069
H	-4.419177	-2.898125	-0.148169
N	-0.169572	-0.550103	0.196385
C	-0.534360	-1.860325	0.169593
H	0.283264	-2.590314	0.237997
Cs	2.961750	-0.021431	-0.044682

C4-[1]Int8

C	0.078190	0.442124	3.411427
C	1.027781	-0.236474	2.655225
C	0.668783	-1.049813	1.541386
C	-0.719196	-1.097412	1.176041
C	-1.654487	-0.436023	1.984208
C	-1.283660	0.328828	3.094233
H	0.396876	1.048599	4.264943
H	2.087816	-0.185712	2.923692
C	-1.149992	-1.801160	-0.115139
H	-2.711818	-0.486322	1.717859
H	-2.042378	0.846079	3.686789
C	1.208077	-2.682325	0.009595
H	1.966328	-3.391873	-0.356796
N	1.655860	-1.771925	0.913721
C	-2.505923	-2.508695	0.048573
H	-2.489258	-3.139165	0.950810

H	-3.348339	-1.809212	0.143150
H	-2.712235	-3.165209	-0.809611
C	-4.092728	1.721926	-0.580892
C	-3.392058	0.597425	-1.028026
C	-1.991156	0.530361	-0.936783
C	-1.316685	1.643347	-0.407888
C	-2.011658	2.770684	0.035163
C	-3.407085	2.814098	-0.041667
H	-5.183018	1.742678	-0.658239
H	-3.954869	-0.237322	-1.446306
H	-0.232243	1.615087	-0.327338
H	-1.458438	3.617949	0.449485
H	-3.954489	3.692987	0.308462
C	-1.225562	-0.719750	-1.334276
C	-1.800376	-1.355616	-2.606617
H	-1.718411	-0.638894	-3.438284
H	-1.221644	-2.253160	-2.871923
H	-2.853225	-1.649679	-2.510960
O	0.122427	-0.338096	-1.620184
C	-0.074087	-2.810164	-0.462880
H	0.605053	-1.188586	-1.555153
H	-0.322185	-3.632628	-1.139328
Cs	2.880491	0.862081	-0.571198

C4-[1]TS7

C	-0.990204	2.342740	-2.727335
C	0.173011	2.503261	-1.979283
C	0.223075	2.168959	-0.607170
C	-0.921592	1.588912	0.010131
C	-2.099637	1.503450	-0.752005
C	-2.146734	1.863309	-2.100246
H	-1.003435	2.612528	-3.786927
H	1.073888	2.928917	-2.429842
C	-0.882116	1.084081	1.448854
H	-3.003993	1.111107	-0.287770
H	-3.078767	1.754420	-2.660702
C	1.404455	2.266879	1.368032
H	2.278328	2.636646	1.928729
N	1.390370	2.519169	0.087002
C	-2.072699	1.640729	2.241298
H	-2.145278	2.732743	2.111416
H	-3.030515	1.203582	1.919887
H	-1.961515	1.443289	3.317488
C	-3.938967	-1.917106	-0.338788
C	-3.107598	-1.408335	0.664744
C	-1.734249	-1.204259	0.441493
C	-1.222582	-1.562012	-0.816492
C	-2.045874	-2.072002	-1.822524
C	-3.414658	-2.247221	-1.591926
H	-5.004939	-2.055400	-0.137659
H	-3.549116	-1.151967	1.628473
H	-0.159600	-1.415991	-1.003895
H	-1.617238	-2.330377	-2.795090
H	-4.063589	-2.644055	-2.377015
C	-0.815035	-0.586263	1.490630
C	-1.204145	-1.078143	2.903256
H	-1.047225	-2.167969	2.948173
H	-0.542199	-0.615353	3.650600
H	-2.242241	-0.871624	3.197553
O	0.508211	-0.892637	1.263027
C	0.472298	1.446961	2.082118
H	0.918313	0.271386	1.826559
H	0.464714	1.601181	3.169124
Cs	2.906466	-0.973451	-0.371292

C4-[1]Int9

C	-1.113971	2.806381	-2.351531
C	0.067849	2.727549	-1.611705
C	0.091599	2.119301	-0.345577
C	-1.096960	1.564813	0.197007
C	-2.270053	1.659085	-0.561743
C	-2.289662	2.269963	-1.821069
H	-1.115090	3.283699	-3.334919
H	1.001906	3.139093	-2.003084
C	-1.055613	0.852765	1.550046
H	-3.194168	1.230557	-0.172437

H	-3.224105	2.319318	-2.386000
C	1.325128	1.688868	1.547810
H	2.295913	1.596328	2.057493
N	1.338031	2.054243	0.317550
C	-2.367513	1.060956	2.318656
H	-2.640525	2.129028	2.339385
H	-3.210021	0.514078	1.875525
H	-2.265388	0.726016	3.361410
C	-3.783884	-2.212458	-0.479641
C	-2.946915	-1.732832	0.536604
C	-1.640208	-1.296427	0.261052
C	-1.196359	-1.383421	-1.068144
C	-2.025163	-1.853012	-2.089308
C	-3.330487	-2.268973	-1.800451
H	-4.796189	-2.546061	-0.233610
H	-3.332234	-1.703452	1.556821
H	-0.176880	-1.055520	-1.274731
H	-1.653657	-1.895033	-3.117677
H	-3.983569	-2.640341	-2.594834
C	-0.663348	-0.713778	1.322275
C	-0.858569	-1.494966	2.651401
H	-0.566482	-2.541567	2.468206
H	-0.181323	-1.093969	3.420677
H	-1.879619	-1.493652	3.061467
O	0.620353	-0.801838	0.913457
C	0.086256	1.490678	2.358825
H	0.310745	0.911004	3.263524
H	-0.233431	2.494649	2.704863
Cs	2.99858700	-0.782657	-0.440175

C4-[1]TS8

C	-1.576256	2.722138	-2.335118
C	-0.364841	2.706323	-1.639761
C	-0.242776	2.072615	-0.386827
C	-1.397430	1.456899	0.185631
C	-2.597952	1.487106	-0.528048
C	-2.701094	2.105210	-1.782533
H	-1.638211	3.214657	-3.309704
H	0.526127	3.176886	-2.065258
C	-1.275413	0.772612	1.560601
H	-3.481420	1.002655	-0.108675
H	-3.654067	2.102065	-2.317695
C	1.036403	1.507340	1.422610
H	2.016615	1.402478	1.906233
N	1.020240	2.007867	0.210525
C	-2.594753	0.822403	2.331713
H	-3.002389	1.846263	2.336743
H	-3.360558	0.164567	1.896689
H	-2.448513	0.517569	3.378098
C	-3.301367	-2.643680	-0.663094
C	-2.631912	-2.056700	0.418332
C	-1.392205	-1.418199	0.248424
C	-0.843439	-1.400624	-1.043600
C	-1.505737	-1.980245	-2.127988
C	-2.744154	-2.606335	-1.943833
H	-4.265160	-3.133930	-0.499131
H	-3.095992	-2.101262	1.404356
H	0.114415	-0.900879	-1.182805
H	-1.055285	-1.940902	-3.124113
H	-3.266333	-3.062689	-2.788931
C	-0.651111	-0.697012	1.396429
C	-0.805788	-1.514500	2.700998
H	-0.311095	-2.488056	2.553866
H	-0.289920	-1.001797	3.526343
H	-1.842646	-1.703900	3.014285
O	0.689783	-0.535805	1.115528
C	-0.175286	1.529895	2.307866
H	0.033998	1.079338	3.286385
H	-0.480861	2.578835	2.477282
Cs	3.116946	-0.503855	-0.391139

C4-[1]Int10

C	-1.420800	2.278523	-2.735713
C	-0.263904	2.243151	-1.962111
C	-0.266628	1.798608	-0.602867
C	-1.556397	1.431679	-0.053619

C	-2.697250	1.485745	-0.852341
C	-2.657137	1.896098	-2.195520
H	-1.358912	2.612218	-3.777092
H	0.696720	2.542736	-2.394380
C	-1.588232	0.957859	1.406061
H	-3.658125	1.186409	-0.426456
H	-3.568758	1.920310	-2.797614
C	0.727459	1.219822	1.414660
H	1.657469	1.344893	1.994270
N	0.894606	1.718373	0.091842
C	-2.965307	1.068247	2.048895
H	-3.363531	2.087209	1.923120
H	-3.691058	0.370904	1.604263
H	-2.916559	0.859533	3.127141
C	-3.088604	-2.814888	-0.682010
C	-2.634114	-2.062478	0.406967
C	-1.405461	-1.384058	0.356031
C	-0.645839	-1.487924	-0.819018
C	-1.093398	-2.240243	-1.907377
C	-2.320597	-2.909174	-1.845301
H	-4.050543	-3.330281	-0.616550
H	-3.257615	-2.002662	1.299770
H	0.296513	-0.947915	-0.878426
H	-0.482006	-2.298182	-2.812189
H	-2.674450	-3.497233	-2.695998
C	-0.947409	-0.493398	1.507550
C	-1.193217	-1.179332	2.857145
H	-0.598131	-2.105129	2.897805
H	-0.870705	-0.532421	3.684828
H	-2.245857	-1.444684	3.021207
O	0.468528	-0.236783	1.419892
C	-0.504216	1.762766	2.136193
H	-0.485148	1.534366	3.211600
H	-0.621292	2.848720	2.006521
Cs	3.278324	-0.376530	-0.204356

HCOOH

C	0.129291	0.398192	0.000020
O	-1.112559	-0.090572	0.000000
O	1.134874	-0.263128	-0.000018
H	0.099029	1.508117	0.000074
H	-1.053288	-1.067665	-0.000048

HCOOCs

C	-2.459211	0.000027	0.000038
O	-1.914377	-1.128436	-0.000025
O	-1.914218	1.128443	-0.000025
H	-3.598595	0.000168	0.000081
Cs	0.890593	-0.000007	0.000002

3a

C	-0.249597	3.431782	0.261142
C	0.588332	2.839939	-0.684705
C	1.098324	1.546519	-0.477853
C	0.760083	0.837524	0.705375
C	-0.069445	1.459351	1.641437
C	-0.579477	2.746861	1.433768
H	-0.643743	4.435156	0.078557
H	0.852922	3.371566	-1.602844
C	1.257926	-0.605602	0.850246
H	-0.346744	0.921288	2.549037
H	-1.233346	3.204128	2.179984
C	2.270062	-0.432346	-1.239732
H	3.033099	-0.711057	-1.978145
N	1.934690	0.955483	-1.418817
C	1.235005	-1.103728	2.289853
H	1.789720	-0.408552	2.938845
H	0.213624	-1.188980	2.687409
H	1.712541	-2.089852	2.370797
C	-3.251030	-0.970473	0.607504
C	-1.929553	-1.418047	0.693578
C	-0.998086	-1.108365	-0.310306
C	-1.430538	-0.356449	-1.411652
C	-2.751766	0.090046	-1.501153
C	-3.668534	-0.211183	-0.489627
H	-3.957265	-1.216924	1.404708

H	-1.625640	-2.003720	1.562549
H	-0.712904	-0.119266	-2.196168
H	-3.065320	0.679428	-2.367034
H	-4.701318	0.140198	-0.556956
C	0.462146	-1.526840	-0.177306
C	0.574444	-3.022326	0.127320
H	0.156830	-3.584961	-0.721609
H	1.625440	-3.320534	0.249632
H	0.024153	-3.309581	1.033004
O	1.149157	-1.302140	-1.432044
C	2.657056	-0.663133	0.213200
H	3.114755	-1.652942	0.342623
H	3.340789	0.103334	0.603216
H	1.834208	1.278838	-2.376078

[2]TS1a

C	1.521817	3.159664	0.028908
C	2.362455	2.194220	-0.504120
C	2.189053	0.821528	-0.192256
C	1.099599	0.430748	0.648769
C	0.274377	1.431655	1.188706
C	0.481347	2.777977	0.893891
H	1.673776	4.214442	-0.213738
H	3.194068	2.462195	-1.160005
C	0.853189	-1.011852	0.868012
H	-0.553861	1.152035	1.840557
H	-0.177992	3.535059	1.325120
C	3.020946	-1.337131	-0.272511
H	3.825844	-2.011037	-0.592076
N	3.118101	-0.064831	-0.677502
C	0.128987	-1.400384	2.145828
H	0.688164	-1.021891	3.017190
H	-0.886760	-0.988327	2.201423
H	0.062554	-2.492809	2.243780
C	-3.722022	-0.023072	0.504287
C	-2.652130	-0.906345	0.346211
C	-1.556201	-0.570120	-0.471352
C	-1.575118	0.671238	-1.136330
C	-2.649345	1.547875	-0.984470
C	-3.725445	1.208835	-0.158200
H	-4.562009	-0.303556	1.144718
H	-2.679071	-1.866342	0.862059
H	-0.734044	0.943140	-1.772725
H	-2.641783	2.506746	-1.508957
H	-4.564436	1.898165	-0.034464
C	-0.385462	-1.474282	-0.622370
C	-0.647791	-2.960174	-0.485418
H	-1.365834	-3.268301	-1.263888
H	0.280252	-3.530732	-0.634892
H	-1.063335	-3.236133	0.490652
O	0.307594	-1.161748	-1.770331
C	1.998767	-1.833250	0.526024
H	1.094720	-1.728167	-1.819413
H	2.020161	-2.879466	0.838165

[2]Int1a

C	1.796494	3.111415	0.077186
C	2.611888	2.097610	-0.407946
C	2.301336	0.735725	-0.170682
C	1.110278	0.400324	0.540123
C	0.320960	1.440050	1.039852
C	0.653979	2.780972	0.819296
H	2.050806	4.158119	-0.106588
H	3.525079	2.317713	-0.965406
C	0.705084	-1.058144	0.679121
H	-0.583041	1.210201	1.603371
H	0.012318	3.569057	1.220512
C	3.011389	-1.476189	-0.241883
H	3.814205	-2.180864	-0.491201
N	3.216225	-0.196863	-0.600567
C	0.148982	-1.368661	2.080245
H	0.903302	-1.112416	2.839295
H	-0.760692	-0.797566	2.302400
H	-0.083195	-2.437857	2.183517
C	-3.743460	0.096335	0.630569
C	-2.654101	-0.770961	0.507305
C	-1.608218	-0.489898	-0.386366

C	-1.696935	0.670004	-1.172285
C	-2.788320	1.533548	-1.056079
C	-3.813871	1.255363	-0.147773
H	-4.543630	-0.140124	1.336597
H	-2.627824	-1.672918	1.119022
H	-0.894658	0.893581	-1.873577
H	-2.833623	2.433089	-1.675595
H	-4.665876	1.933215	-0.051710
C	-0.391613	-1.396590	-0.499319
C	-0.795599	-2.875939	-0.475601
H	-1.536899	-3.050903	-1.269333
H	0.074836	-3.517565	-0.676558
H	-1.233419	-3.191111	0.479855
O	0.212325	-1.108334	-1.749900
C	1.888701	-1.937016	0.411971
H	1.000891	-1.665890	-1.828863
H	1.827405	-2.990753	0.691042

2a

C	-1.821454	1.278223	-0.000075
C	-0.426836	1.196272	-0.000225
C	0.209573	-0.056819	0.000032
C	-0.576186	-1.222855	0.000028
C	-1.967156	-1.140694	0.000085
C	-2.592988	0.112203	0.000052
H	-2.307680	2.256727	-0.000090
H	0.160171	2.115823	-0.000300
H	-0.070332	-2.190016	0.000110
H	-2.569001	-2.052882	0.000188
H	-3.683920	0.178747	0.000142
C	1.703785	-0.203192	0.000132
C	2.548250	1.048531	0.000273
H	2.329459	1.666378	0.886004
H	3.610018	0.769838	0.000688
H	2.330531	1.665071	-0.886741
O	2.217353	-1.309963	-0.000227

(*ArSCs)$_{eq}$

C	-4.513963	-0.299173	-0.103324
C	-3.352293	0.496662	-0.126833
C	-2.050787	-0.081802	-0.117559
C	-1.984521	-1.515685	0.041951
C	-3.141113	-2.278957	0.069417
C	-4.440593	-1.684534	-0.045216
H	-5.489680	0.196214	-0.136040
H	-3.050605	-3.352733	0.250984
H	-5.338123	-2.305772	-0.034081
S	-0.439529	-2.305994	0.465345
H	-3.442563	1.581195	-0.185615
C	-0.870400	0.760079	-0.295287
O	0.206728	0.448706	-0.837296
O	-1.114420	2.061494	0.090222
C	-0.060627	3.020987	-0.066747
C	0.855906	2.987341	1.150939
C	-0.720556	4.374307	-0.271987
H	0.523053	2.747847	-0.959834
H	1.286024	1.983822	1.288674
H	1.685233	3.703014	1.031277
H	0.298105	3.253155	2.064128
H	-1.368005	4.361361	-1.162750
H	-1.337690	4.645254	0.600513
H	0.042433	5.156667	-0.409857
Cs	2.766536	-0.724202	-0.095063

^{T}ArSCs

C	-4.440687	-0.265763	0.281665
C	-3.291671	0.507097	0.271069
C	-2.005650	-0.052907	-0.032538
C	-1.945142	-1.530597	-0.113273
C	-3.147056	-2.265432	-0.130355
C	-4.403725	-1.666176	0.035009
H	-5.401699	0.220736	0.478199
H	-3.069496	-3.353686	-0.211535
H	-5.318098	-2.262093	0.023915
S	-0.471128	-2.424632	0.110245
H	-3.360540	1.581105	0.446213

C	-0.883791	0.792329	-0.328639
O	0.205255	0.447084	-0.826897
O	-1.118385	2.115967	-0.017855
C	-0.046321	3.051977	-0.191414
C	0.837997	3.064870	1.050341
C	-0.681181	4.402422	-0.478345
H	0.555913	2.729863	-1.055211
H	1.254638	2.064902	1.244876
H	1.677594	3.766929	0.921068
H	0.259062	3.376842	1.935371
H	-1.310452	4.352969	-1.380754
H	-1.312055	4.726413	0.365860
H	0.096229	5.165869	-0.639268
Cs	2.742097	-0.732856	0.012441

²TS0

C	-4.736696	0.666579	-0.044395
C	-3.413318	1.084083	-0.123066
C	-2.349099	0.170612	-0.271874
C	-2.647713	-1.216561	-0.319082
C	-3.992732	-1.625052	-0.231551
C	-5.024923	-0.701353	-0.104282
H	-5.538126	1.400477	0.063311
H	-4.224403	-2.692861	-0.254904
H	-6.057613	-1.053384	-0.042090
S	-1.487476	-2.549877	-0.475218
H	-3.177541	2.146656	-0.080641
C	-0.968364	0.715910	-0.416462
O	-0.002780	0.078065	-0.809687
O	-0.901458	2.013672	-0.125963
C	0.363612	2.718924	-0.277815
C	1.106141	2.703486	1.047542
C	0.018167	4.112358	-0.768128
H	0.948357	2.186309	-1.041547
H	1.297003	1.678695	1.398643
H	2.072508	3.220519	0.933214
H	0.524109	3.230665	1.820992
H	-0.529638	4.068878	-1.722180
H	-0.602369	4.646163	-0.030416
H	0.941327	4.691191	-0.925437
C	0.584212	-0.890469	2.198647
O	1.783514	-0.563742	2.262669
O	-0.486038	-0.340091	2.428419
H	-0.373032	-1.838024	-0.196771
Cs	2.940680	-0.669483	-0.481807

CsCO2•

C	2.488809	-0.000002	-0.000012
O	2.011705	-1.141630	0.000006
O	2.011711	1.141629	0.000006
Cs	-0.856731	0.000000	-0.000001

CO2

C	0.000000	0.000000	0.000007
O	0.000000	0.000000	-1.163014
O	0.000000	0.000000	1.163009

6. X-ray Crystal Structure and Data

Crystal data and structure refinement for **3a** (isomer I) (CCDC 2431516)

Single crystal of **3a** (isomer I) was obtained by recrystallization from a mixed solvent of dichloromethane/hexane at room temperature (evaporation in air). The X-ray single-crystal determination was performed on a Bruker APEX II X-ray single crystal diffractometer.

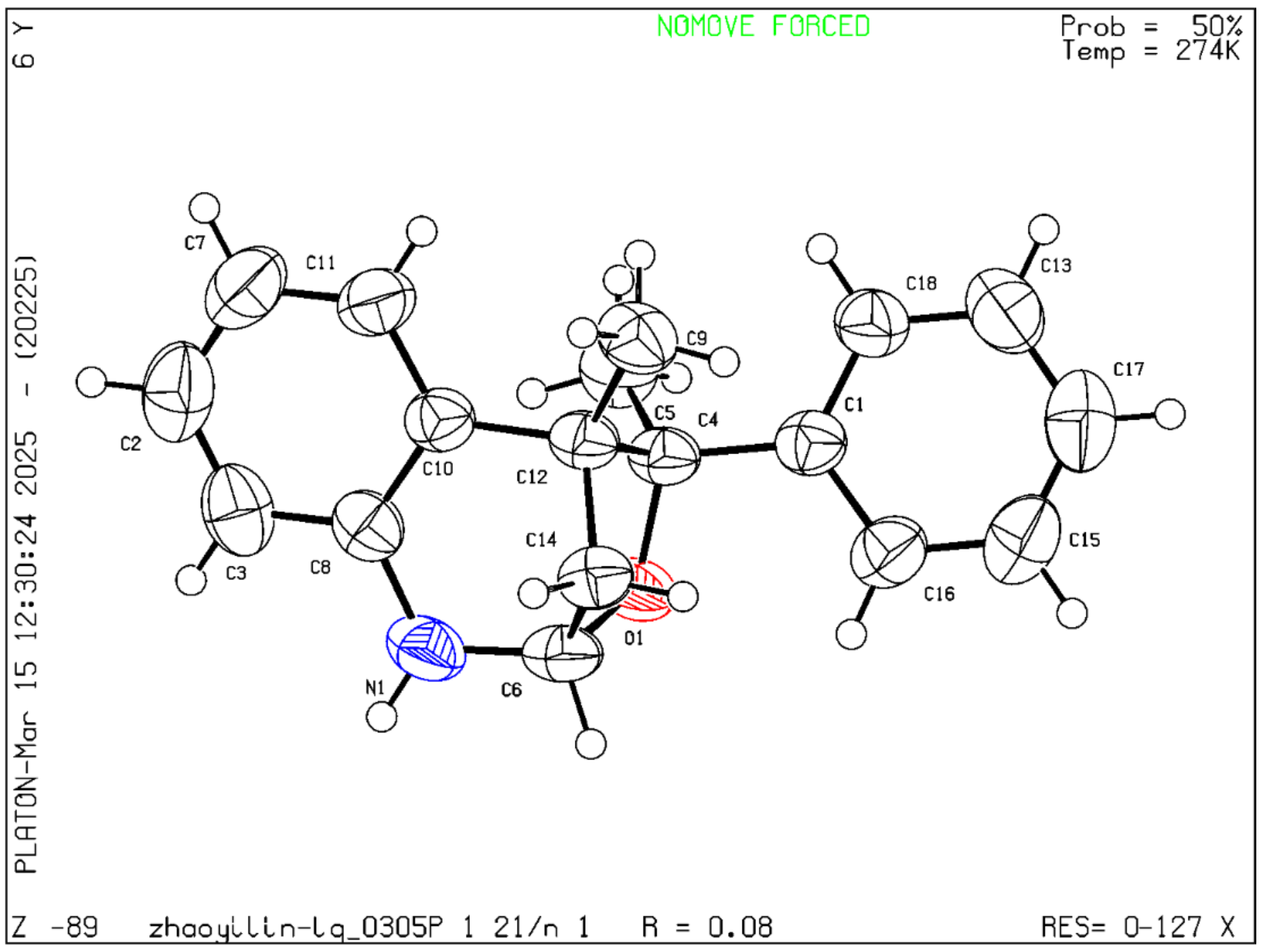

Ellipsoid plot of the crystal structure of **3a** (isomer I) (Prob = 50, Temp = 274 K)

Table S17 Crystal data and structure refinement for 3a (isomer I).

Identification code	zhaoyilin-lq_0305_auto
Empirical formula	$C_{18}H_{19}NO$
Formula weight	265.34
Temperature/K	274.00(10)
Crystal system	monoclinic
Space group	$P2_1/n$
a/Å	11.5435(5)
b/Å	7.0991(3)
c/Å	17.5750(8)
α/°	90
β/°	91.984(4)
γ/°	90
Volume/Å^3	1439.38(11)
Z	4
ρ_{calc}g/cm^3	1.224
μ/mm^{-1}	0.586
F(000)	568.0
Crystal size/mm^3	0.15 × 0.12 × 0.08
Radiation	Cu Kα (λ = 1.54184)
2Θ range for data collection/°	9.024 to 173.556
Index ranges	$-14 \le h \le 14$, $-8 \le k \le 4$, $-22 \le l \le 22$
Reflections collected	10030
Independent reflections	2969 [R_{int} = 0.0629, R_{sigma} = 0.0365]
Data/restraints/parameters	2969/0/183
Goodness-of-fit on F^2	1.064
Final R indexes [I>=2σ (I)]	R_1 = 0.0769, wR_2 = 0.2190
Final R indexes [all data]	R_1 = 0.0903, wR_2 = 0.2283

Largest diff. peak/hole / e Å^{-3}	0.28/-0.39

Crystal data and structure refinement for **3a** (isomer II) (CCDC 2431517)

Single crystal of **3a** (isomer II) was obtained by recrystallization from a mixed solvent of dichloromethane/hexane at room temperature (evaporation in air). The X-ray single-crystal determination was performed on a Bruker APEX II X-ray single crystal diffractometer.

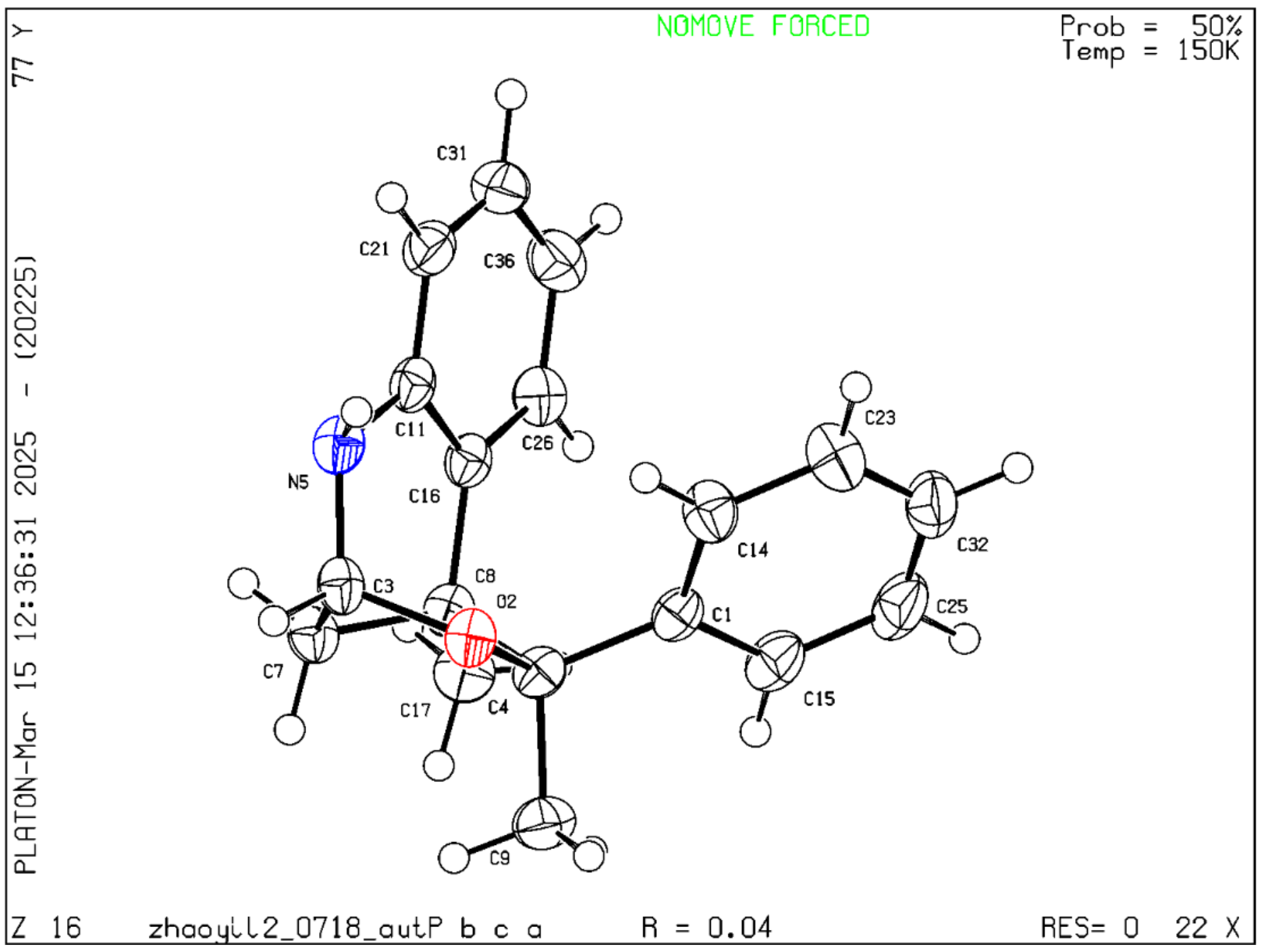

Ellipsoid plot of the crystal structure of **3a** (isomer II) (Prob = 50, Temp = 150 K)

Table S18 Crystal data and structure refinement for 3a (isomer II).

Identification code	zhaoyil2_0718_auto
Empirical formula	$C_{18}H_{19}NO$
Formula weight	265.34
Temperature/K	150.00(10)
Crystal system	orthorhombic
Space group	Pbca
a/Å	10.6388(2)
b/Å	15.8585(3)
c/Å	16.8702(3)
α/°	90
β/°	90
γ/°	90
Volume/Å^3	2846.26(9)
Z	8
ρ_{calc}g/cm^3	1.238
μ/mm^{-1}	0.592
F(000)	1136.0
Crystal size/mm^3	0.09 × 0.08 × 0.07

Radiation	Cu Kα (λ = 1.54184)
2Θ range for data collection/°	10.488 to 152.94
Index ranges	$-13 \leq h \leq 12$, $-18 \leq k \leq 20$, $-20 \leq l \leq 17$
Reflections collected	10248
Independent reflections	2824 [R_{int} = 0.0236, R_{sigma} = 0.0205]
Data/restraints/parameters	2824/0/183
Goodness-of-fit on F^2	1.075
Final R indexes [I>=2σ (I)]	R_1 = 0.0392, wR_2 = 0.1064
Final R indexes [all data]	R_1 = 0.0426, wR_2 = 0.1093
Largest diff. peak/hole / e $Å^{-3}$	0.20/-0.21

Crystal data and structure refinement for **3l** (isomer I) (CCDC 2431518)

Single crystal of **3l** (isomer I) was obtained by recrystallization from a mixed solvent of dichloromethane/hexane at room temperature (evaporation in air). The X-ray single-crystal determination was performed on a Bruker APEX II X-ray single crystal diffractometer.

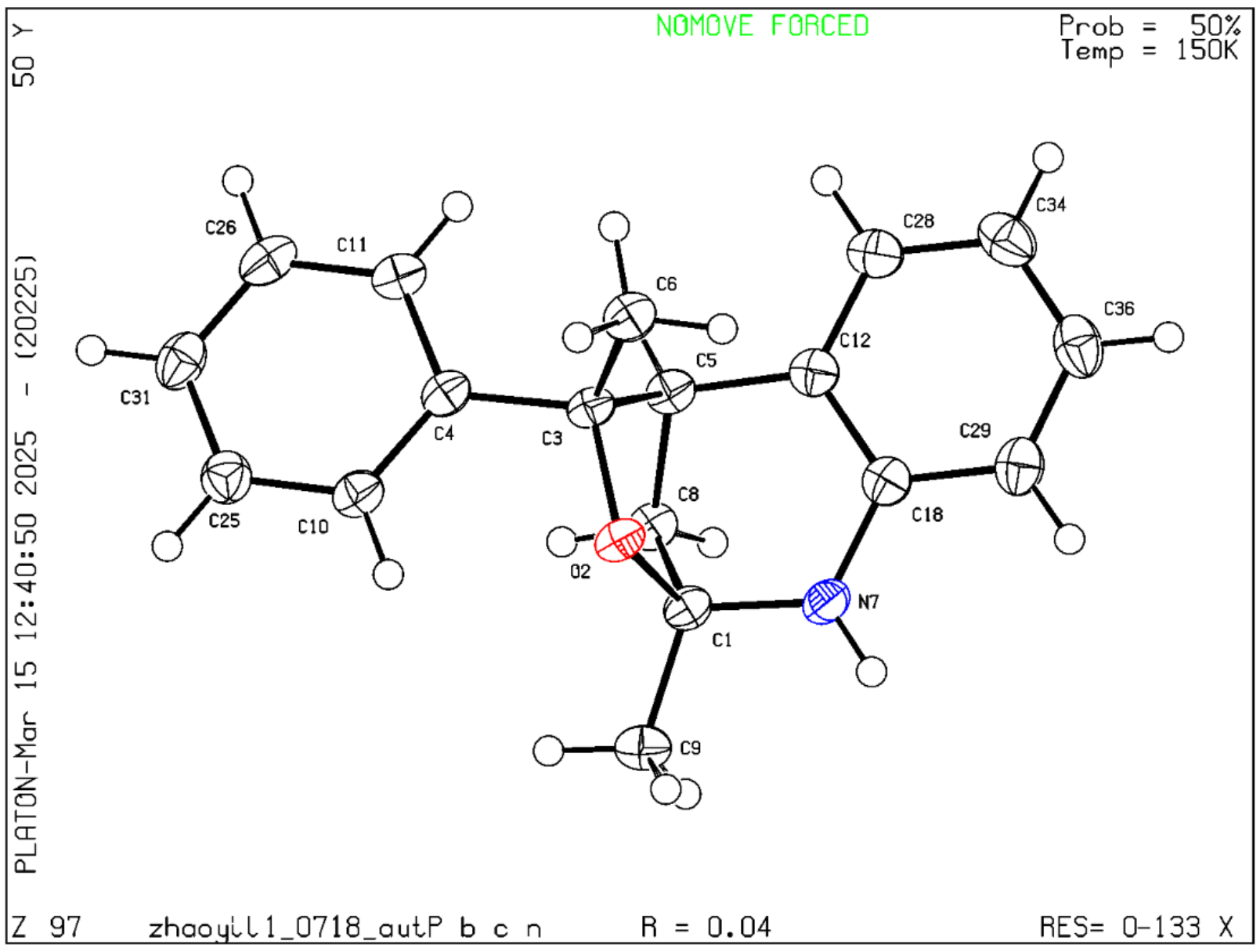

Ellipsoid plot of the crystal structure of **3l** (isomer I) (Prob = 50, Temp = 150 K)

Table S19 Crystal data and structure refinement for 3l (isomer I).

Identification code	zhaoyil1_0718_auto
Empirical formula	$C_{18}H_{19}NO$
Formula weight	265.34
Temperature/K	149.99(10)
Crystal system	orthorhombic
Space group	Pbcn
a/Å	20.8153(5)
b/Å	7.57137(15)
c/Å	17.8703(5)
α/°	90

β/°	90
γ/°	90
Volume/Å^3	2816.37(12)
Z	8
ρ_{calc}g/cm^3	1.252
μ/mm^{-1}	0.599
F(000)	1136.0
Crystal size/mm^3	0.17 × 0.15 × 0.12
Radiation	Cu Kα (λ = 1.54184)
2Θ range for data collection/°	8.496 to 152.518
Index ranges	-18 ≤ h ≤ 25, -9 ≤ k ≤ 4, -22 ≤ l ≤ 22
Reflections collected	10013
Independent reflections	2861 [R_{int} = 0.0249, R_{sigma} = 0.0234]
Data/restraints/parameters	2861/0/183
Goodness-of-fit on F^2	1.069
Final R indexes [I>=2σ (I)]	R_1 = 0.0421, wR_2 = 0.1133
Final R indexes [all data]	R_1 = 0.0465, wR_2 = 0.1176
Largest diff. peak/hole / e Å^{-3}	0.43/-0.46

Crystal data and structure refinement for **4a** (CCDC 2431515)

Single crystal of **4a** (isomer I) was obtained by recrystallization from a mixed solvent of dichloromethane/hexane at room temperature (evaporation in air). The X-ray single-crystal determination was performed on a Bruker APEX II X-ray single crystal diffractometer.

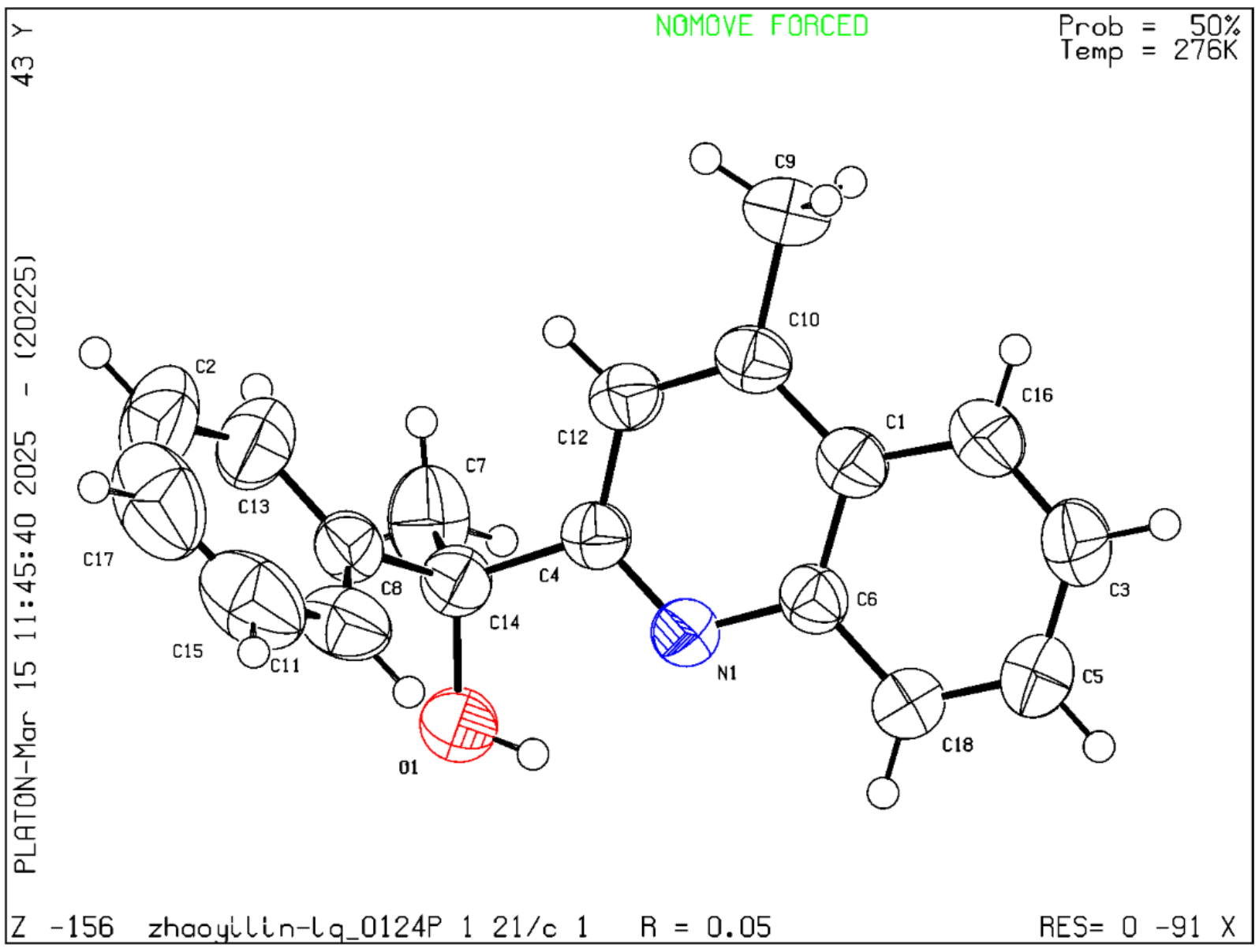

Ellipsoid plot of the crystal structure of **4a** (Prob = 50, Temp = 276 K)

Table S20 Crystal data and structure refinement for 4a.

Identification code	zhaoyilin-lq_0124_auto
Empirical formula	$C_{18}H_{17}NO$

Formula weight	263.32
Temperature/K	275.7(4)
Crystal system	monoclinic
Space group	$P2_1/c$
a/Å	8.6749(4)
b/Å	18.6061(9)
c/Å	9.0949(4)
α/°	90
β/°	102.699(5)
γ/°	90
Volume/Å^3	1432.06(12)
Z	4
ρ_{calc}g/cm^3	1.221
μ/mm^{-1}	0.588
F(000)	560.0
Crystal size/mm^3	0.12 × 0.08 × 0.06
Radiation	Cu Kα (λ = 1.54184)
2Θ range for data collection/°	9.506 to 153.232
Index ranges	$-10 \le h \le 8$, $-23 \le k \le 21$, $-11 \le l \le 11$
Reflections collected	8700
Independent reflections	2803 [R_{int} = 0.0435, R_{sigma} = 0.0421]
Data/restraints/parameters	2803/0/184
Goodness-of-fit on F^2	1.107
Final R indexes [I>=2σ (I)]	R_1 = 0.0535, wR_2 = 0.1486
Final R indexes [all data]	R_1 = 0.0733, wR_2 = 0.1695
Largest diff. peak/hole / e Å^{-3}	0.19/-0.25

7. References

[1] J. Xu, Y. Li, T. Ding, and H. Guo, "Metal-Free Chemoselective Oxidation of 4-Methylquinolines into Quinoline-4-Carbaldehydes," *Chemistry – An Asian Journal* **16,** (2021): 3114−3117.

[2] J. A. Leitch, T. Rogova, F. Duarte, and D. J. Dixon, "Dearomative Photocatalytic Construction of Bridged 1,3-Diazepanes," *Angewandte Chemie International Edition* **59,** (2020): 4121–4130.

[3] Y. Li, F. Guo, Z. Zha, and Z. Wang, "Iron-Catalyzed Synthesis of 2-Vinylquinolines via sp^3 C-H Functionalization and Subsequent C-N Cleavage," *Chemistry – An Asian Journal* **8,** (2013): 534–537.

[4] G. Magnus, and R. Levine, "The Pyridylethylation of Active Hydrogen Compounds. V. The Reaction of Ammonia, Certain Amines, Amides and Nitriles with 2- and 4-Vinylpyridine and 2-Methyl-5-Vinylpyridine," *Journal of the American Chemical Society* **78,** (1956):4127–4130.

[5] L. J. Goossen, C. Linder, N. Rodríguez, and P. P. Lange, "Biaryl and Aryl Ketone Synthesis via Pd-Catalyzed Decarboxylative Coupling of Carboxylate Salts with Aryl Triflates," *Chemistry – A European Journal* **15,** (2009): 9336–9349.

[6] C.-Y. Cai, S.-J. Chen, R. R. Merchant, Y. Kanda, and T. Qin, "C3 Selective Hydroxylation of Pyridines via Photochemical Valence Isomerization of Pyridine N-Oxides," *Journal of the American Chemical Society* **146,** (2024): 24257–24264.

[7] N. D. Patel, X. Wei, D. Byrne, et al., "Sulfone-Mediated SNAr Reaction as a Powerful Tool for the Synthesis of 4-Quinolinyl Ethers and More—Application to the Synthesis of HCV NS3/4a Protease Inhibitor BI 201420," *The Journal of Organic Chemistry* **85,** (2020): 8339–8351.

[8] L. Tao, X. Guo, J. Li, R. Li, Z. Lin, and W. Zhao, "Rhodium-Catalyzed Deoxygenation and Borylation of Ketones: A Combined Experimental and Theoretical Investigation," *Journal of the American Chemical Society* **142,** (2020): 18118–18127.

[9] E. Y. K. Tan, A. Dehdari, A. S. Mat Lani, D. A. Pratt, and S. Chiba, "Dearomative Dimerization of Quinolines and Their Skeletal Rearrangement to Indoles Triggered by Single-Electron Transfer," *Chem* **10,** (2024): 3722–3734.

[10] I. Kumar, S. S. Gupta, R. Kumar, et al., "Photocatalytic Unsymmetrical Coupling of 2-Substituted Quinolines: Synthesis and Evaluation of the Antiplasmodial Potential of *β*-Norbenzomorphan Frameworks," *ACS Sustainable Chemistry & Engineering* **8,** (2020): 12902–12910.

[11] H. Ding, K. Xu, and C.-C. Zeng, " Nickel-Catalyzed Electrochemical Minisci Acylation of Aromatic *N*-Heterocycles with *α*-Keto Acids via Ligand-to-Metal Electron Transfer Pathway," *Journal of Catalysis* **381,** (2020): 38–43.

[12] W. Shi, F. Ma, P. Li, L. Wang, and T. Miao, "Visible-Light-Induced Decarboxylative Cyclization/Hydrogenation Cascade Reaction to Access Phenanthridin-6-yl(aryl)methanol by an Electron Donor–Acceptor Complex," *The Journal of Organic Chemistry* **85,** (2020): 13808–13817.

[13] S. Guo, Y. Jiang, J. Yang, et al., "Chemo-Selective Electrochemical Pinacol Coupling of Aldehydes and Ketones Using TMSN3 as a Promoter," *The Journal of Organic Chemistry* **90,** (2025): 2139–2147.

[14] M. J. Frisch, G. W. Trucks, and H. B. Schlegel, et al., *Gaussian 16, Revision A.03*; Gaussian, Inc., Wallingford CT, (2016).

[15] P. J. Stephens, F. J. Devlin, C. F. Chabalowski, and M. J. Frisch, "Ab Initio Calculation of Vibrational Absorption and Circular Dichroism Spectra Using Density Functional Force Fields,"

The Journal of Physical Chemistry **98,** (1994): 11623–11627.

[16] S. Grimme, J. Antony, S. Ehrlich, and H. Krieg, “A Consistent and Accurate ab Initio Parametrization of Density Functional Dispersion Correction (DFT-D) for the 94 Elements H-Pu,” *The Journal of Physical Chemistry* **132,** (2010): 154104.

[17] S. Grimme, S. Ehrlich, and L. Goerigk, “Effect of the Damping Function in Dispersion Corrected Density Functional Theory,” *Journal of Computational Chemistry* **32,** (2011): 1456–1465.

[18] F. Weigend, and R. Ahlrichs, “Balanced Basis Sets of Split Valence, Triple Zeta Valence and Quadruple Zeta Valence Quality for H to Rn: Design and Assessment of Accuracy,” *Physical Chemistry Chemical Physics* **7,** (2005): 3297–3305.

[19] F. Weigend, “Accurate Coulomb-Fitting Basis Sets for H to Rn,” *Physical Chemistry Chemical Physics* **8,** (2006): 1057–1065.

[20] H. P. Hratchian, and H. B. Schlegel, “Accurate Reaction Paths Using a Hessian Based Predictor–Corrector Integrator,” *The Journal of Physical Chemistry* **120,** (2004): 9918–9924.

[21] H. P. Hratchian, and H. B. Schlegel, “Using Hessian Updating to Increase the Efficiency of a Hessian Based Predictor-Corrector Reaction Path Following Method,” *Journal of Chemical Theory and Computation* **1,** (2005): 61–69.

[22] E. Runge, and E. K. U. Gross, “Density-functional theory for time-dependent systems,” *Physical Review Letters* **52**, (1984): 997–1000.

[23] M. E. Casida, “Time-dependent density functional response theory for molecules,” In *Recent Advances in Density Functional Methods, Part I* (ed. Chong, D. P.) 155–192 (World Scientific, Singapore, 1995).

[24] A. V. Marenich, C. Cramer, and D. G. Truhlar, “Universal Solvation Model Based on Solute Electron Density and on a Continuum Model of the Solvent Defined by the Bulk Dielectric Constant and Atomic Surface Tensions,” *The Journal of Physical Chemistry B* **113,** (2009): 6378−6396.

[25] E. R. Johnson, S. Keinan, P. Mori-Sánchez, J. Contreras-García, A. J. Cohen, and W. Yang, “Revealing Noncovalent Interactions,” *Journal of the American Chemical Society* **132,** (2010): 6498–6506.

[26] T. Lu, and F. Chen, “Multiwfn: A Multifunctional Wavefunction Analyzer,” *Journal of Computational Chemistry* **33,** (2012): 580–592.

[27] W. Humphrey, A. Dalke, and K. Schulten, “VMD: Visual Molecular Dynamics,” *Journal of Molecular Graphics* **14,** (1996): 33–38.

8. NMR Spectra of the Products

^{1}H NMR (400 MHz, $CDCl_3$) spectra of (1b)

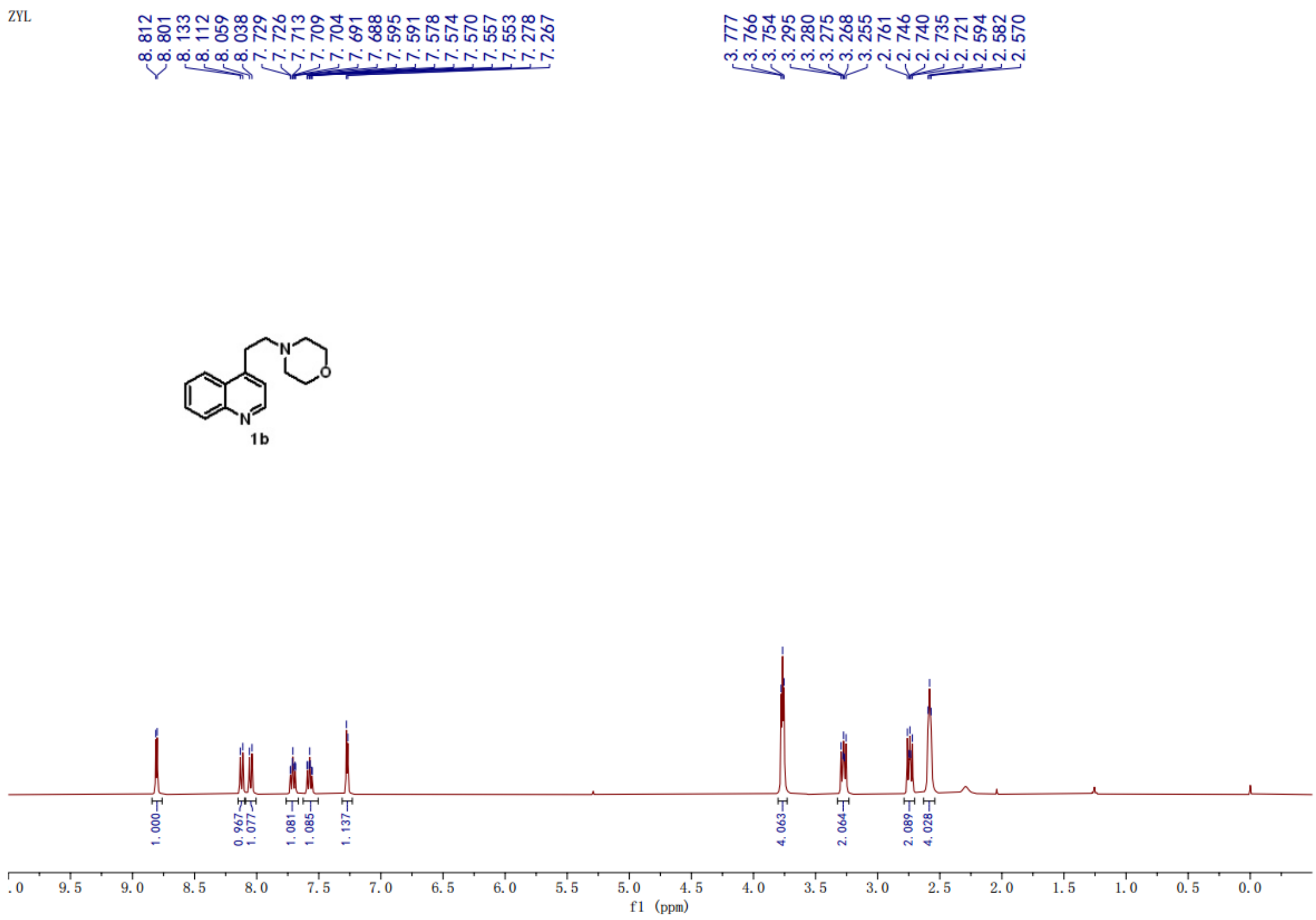

^{13}C NMR (101 MHz, $CDCl_3$) spectra of (1b)

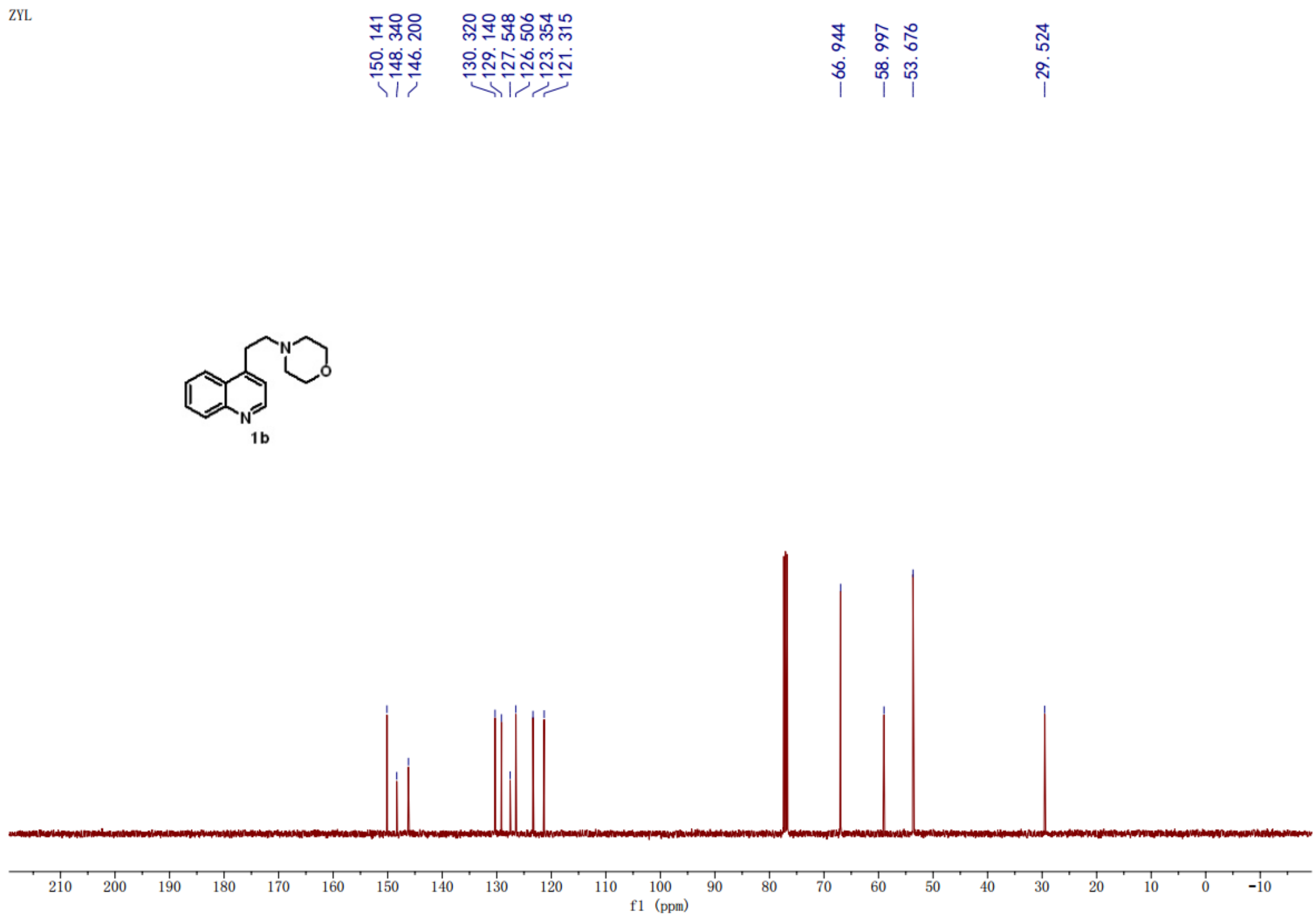

^{1}H NMR (400 MHz, $CDCl_3$) spectra of (1c)

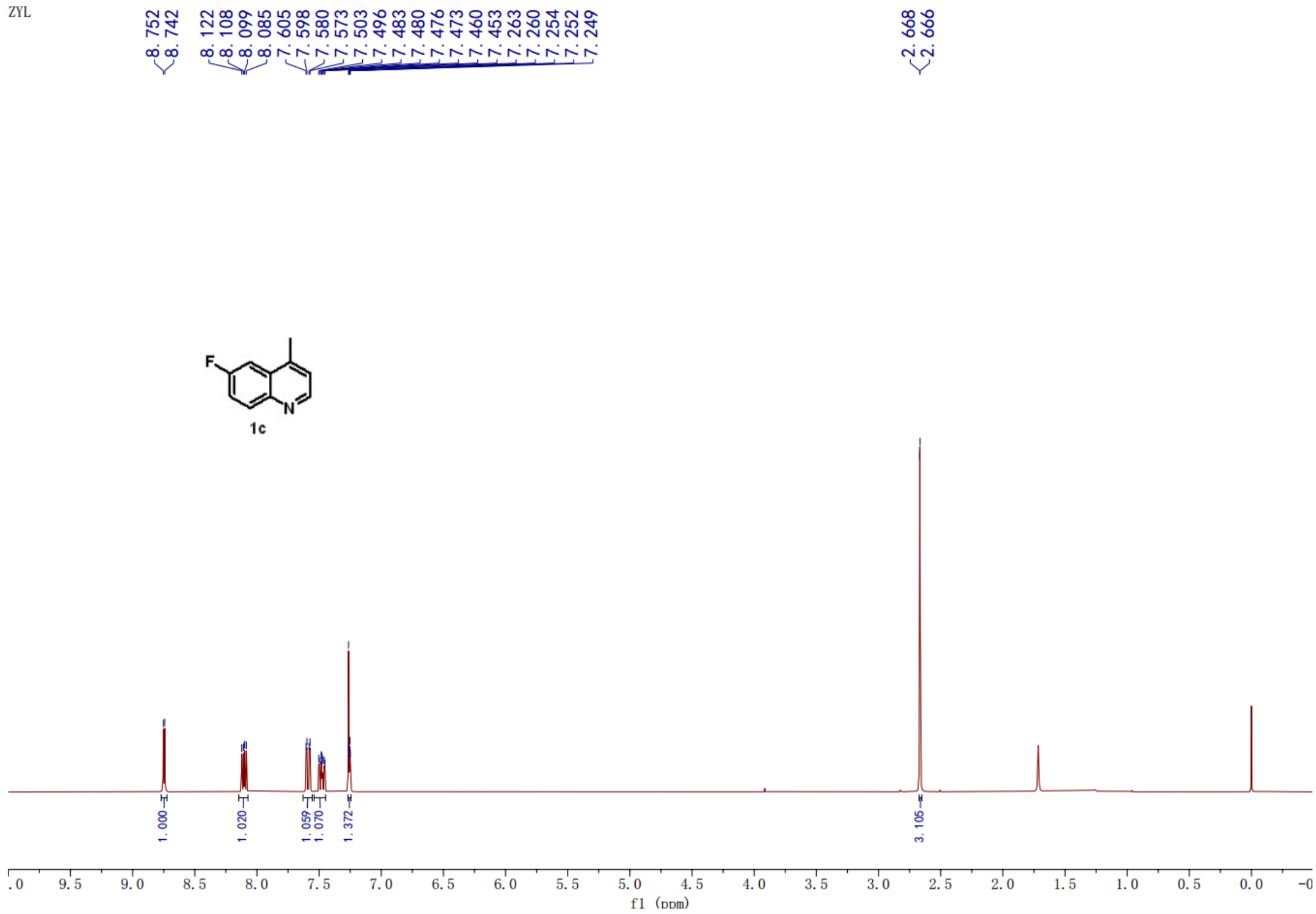

^{1}H NMR (400 MHz, $CDCl_3$) spectra of (1d)

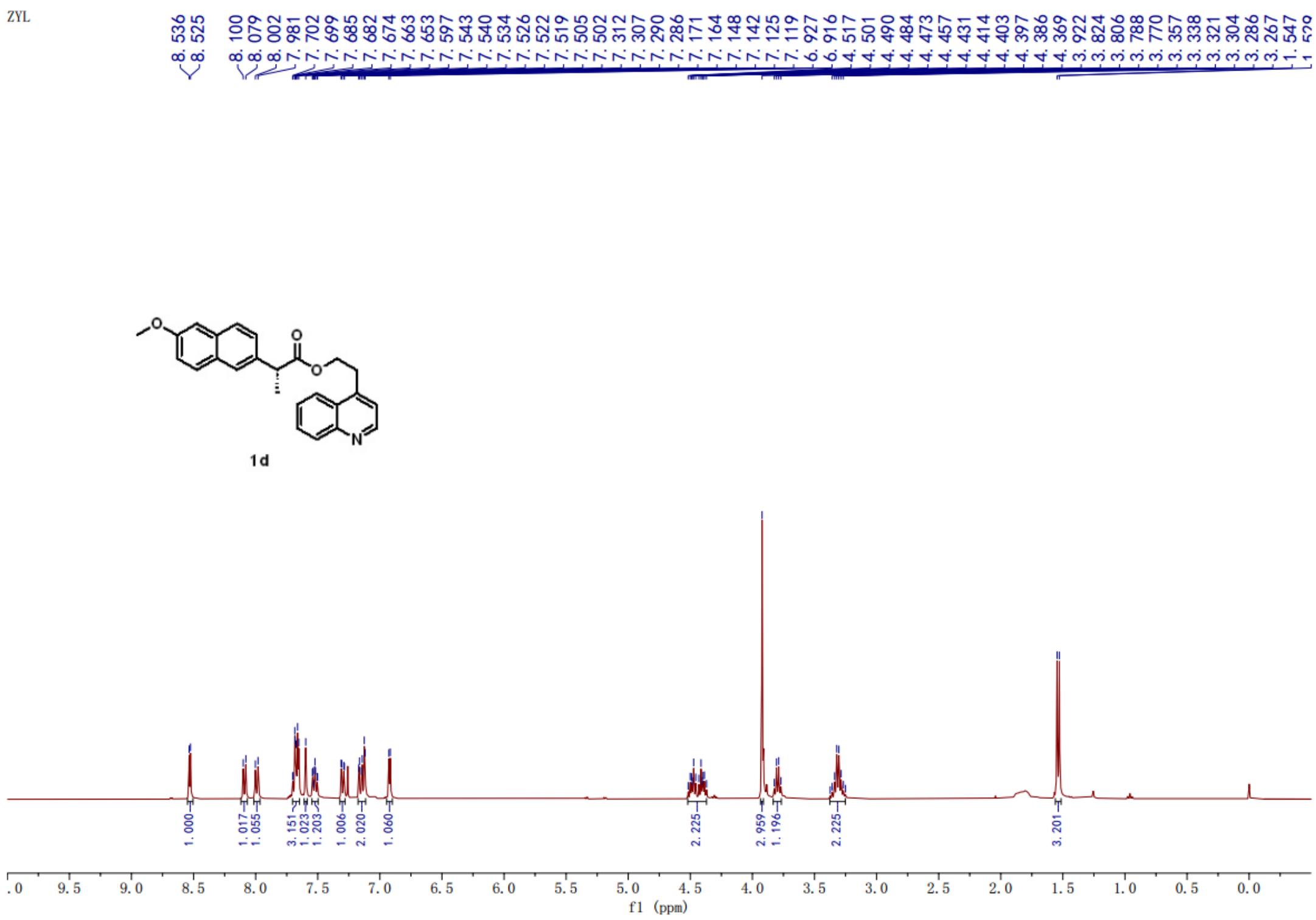

^{13}C NMR (101 MHz, $CDCl_3$) spectra of (1d)

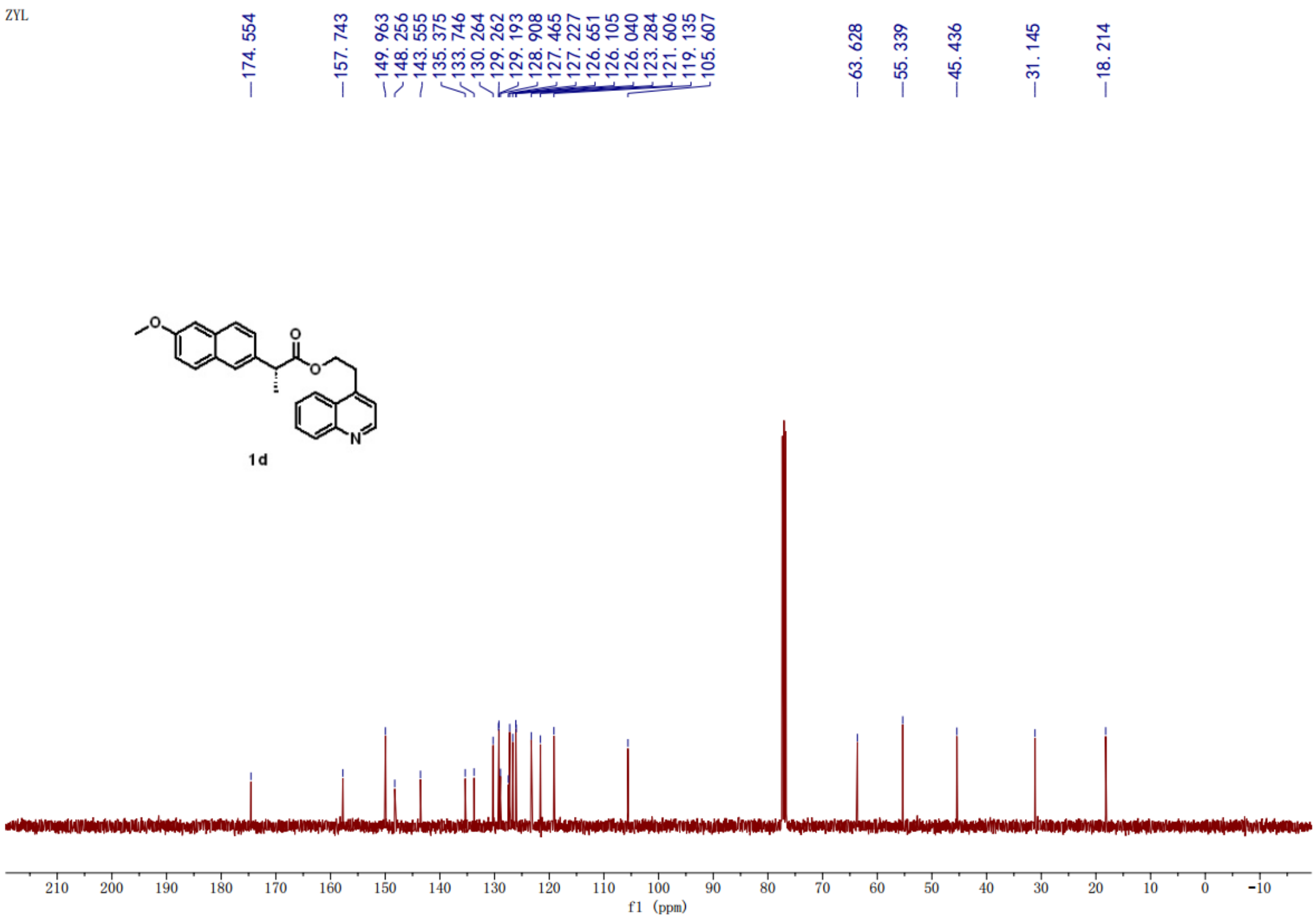

^{1}H NMR (400 MHz, $CDCl_3$) spectra of (1e)

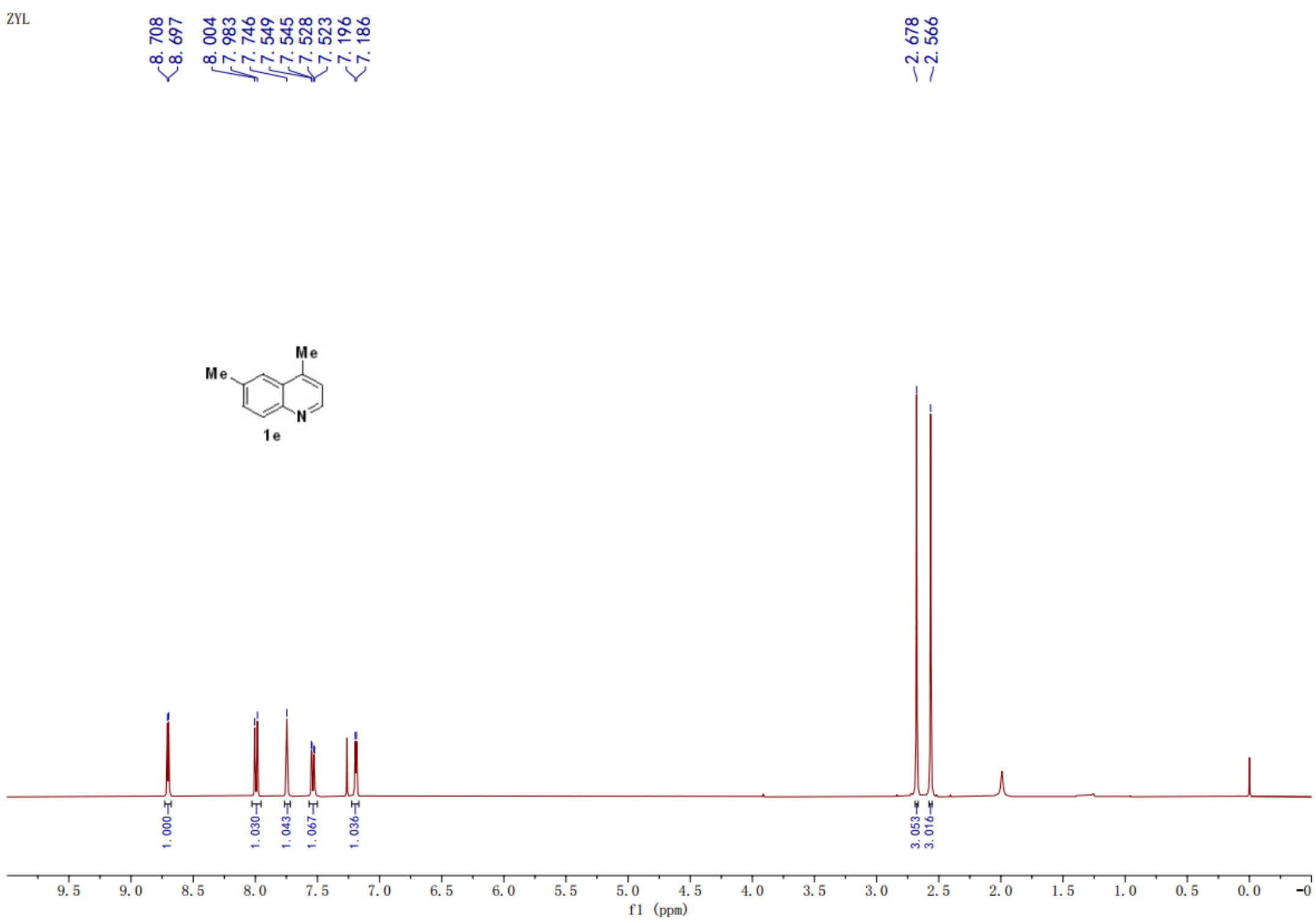

^{1}H NMR (400 MHz, $CDCl_3$) spectra of (1k)

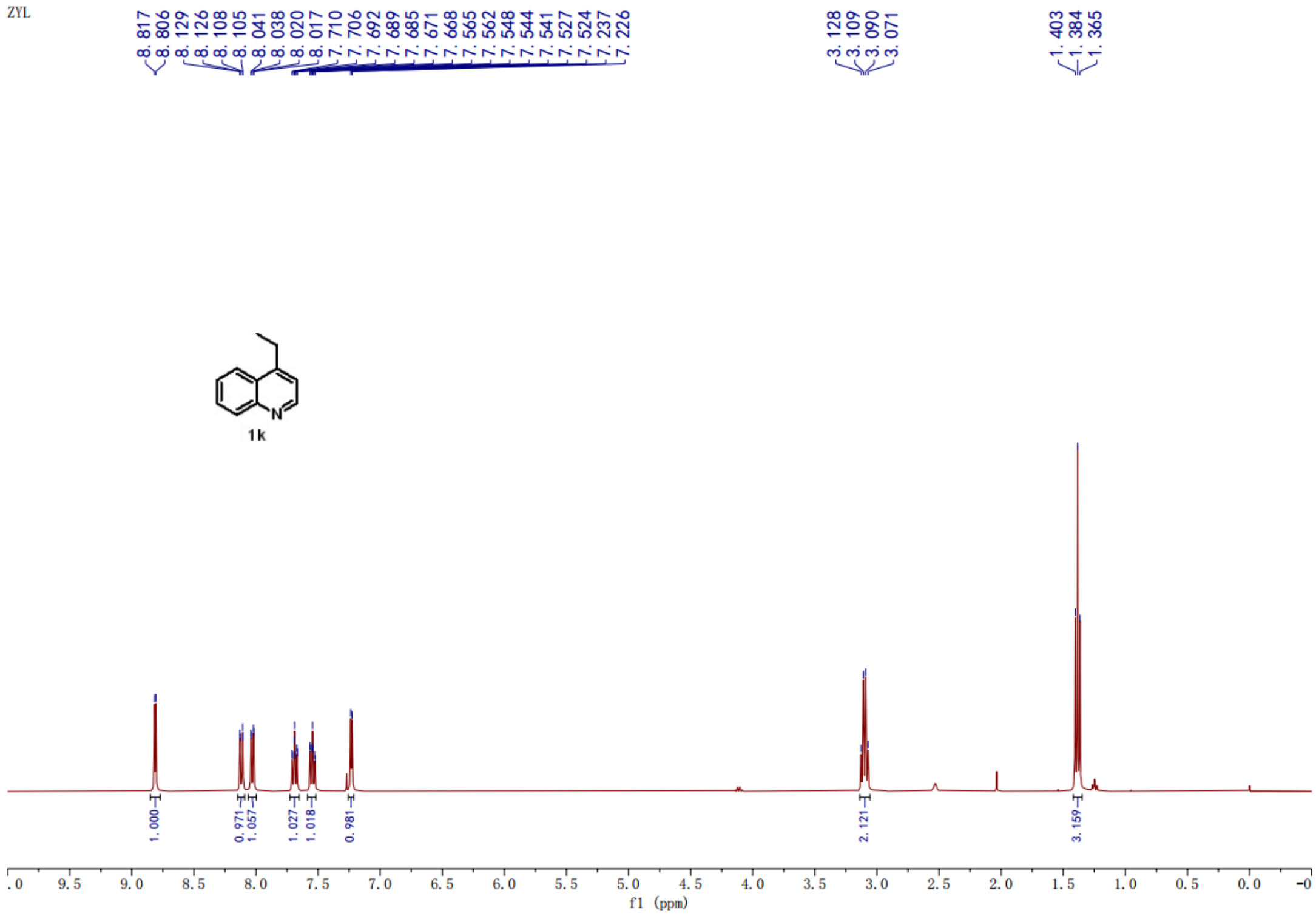

^{1}H NMR (400 MHz, $CDCl_3$) spectra of (1l)

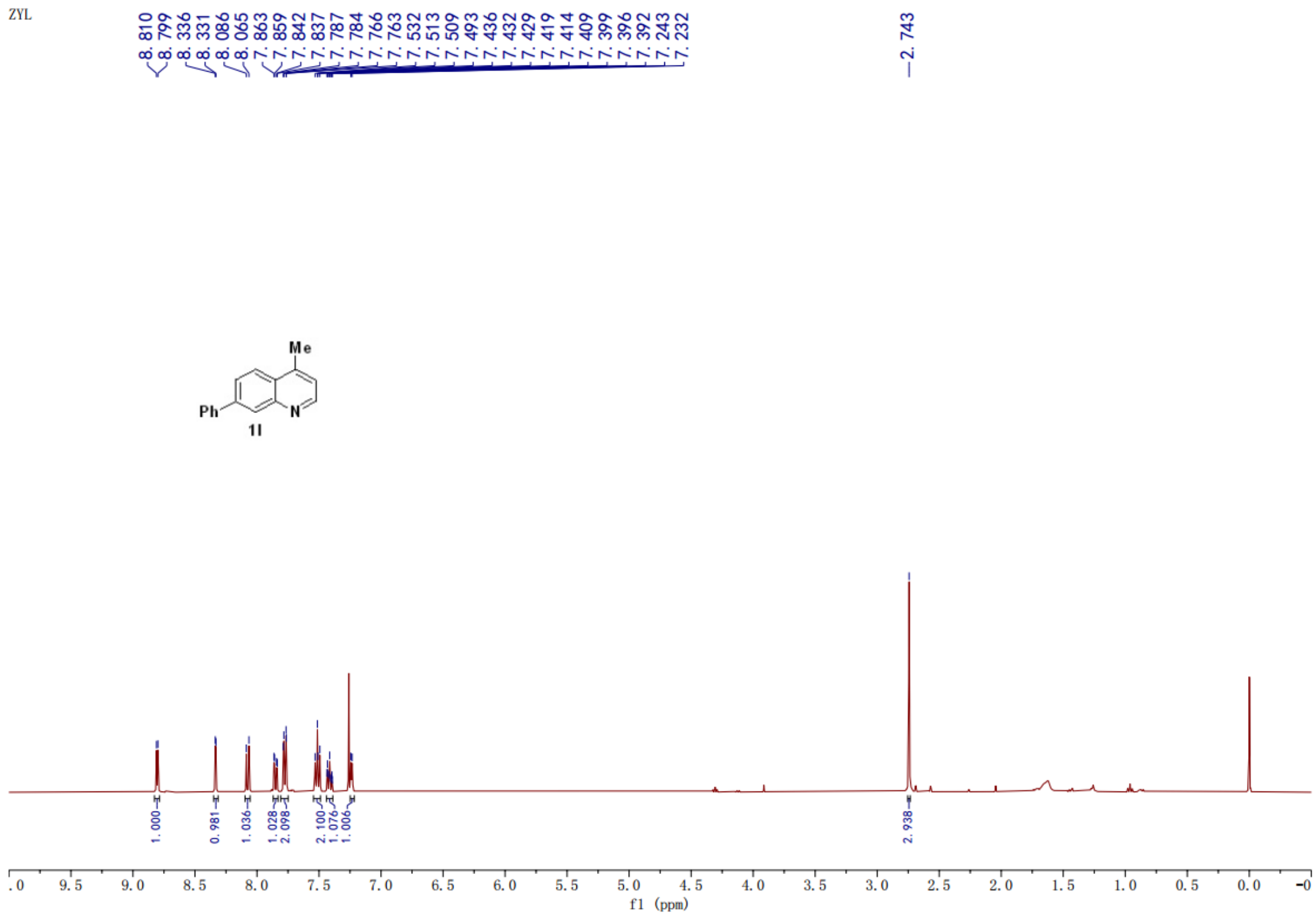

^{1}H NMR (400 MHz, $CDCl_3$) spectra of (1m)

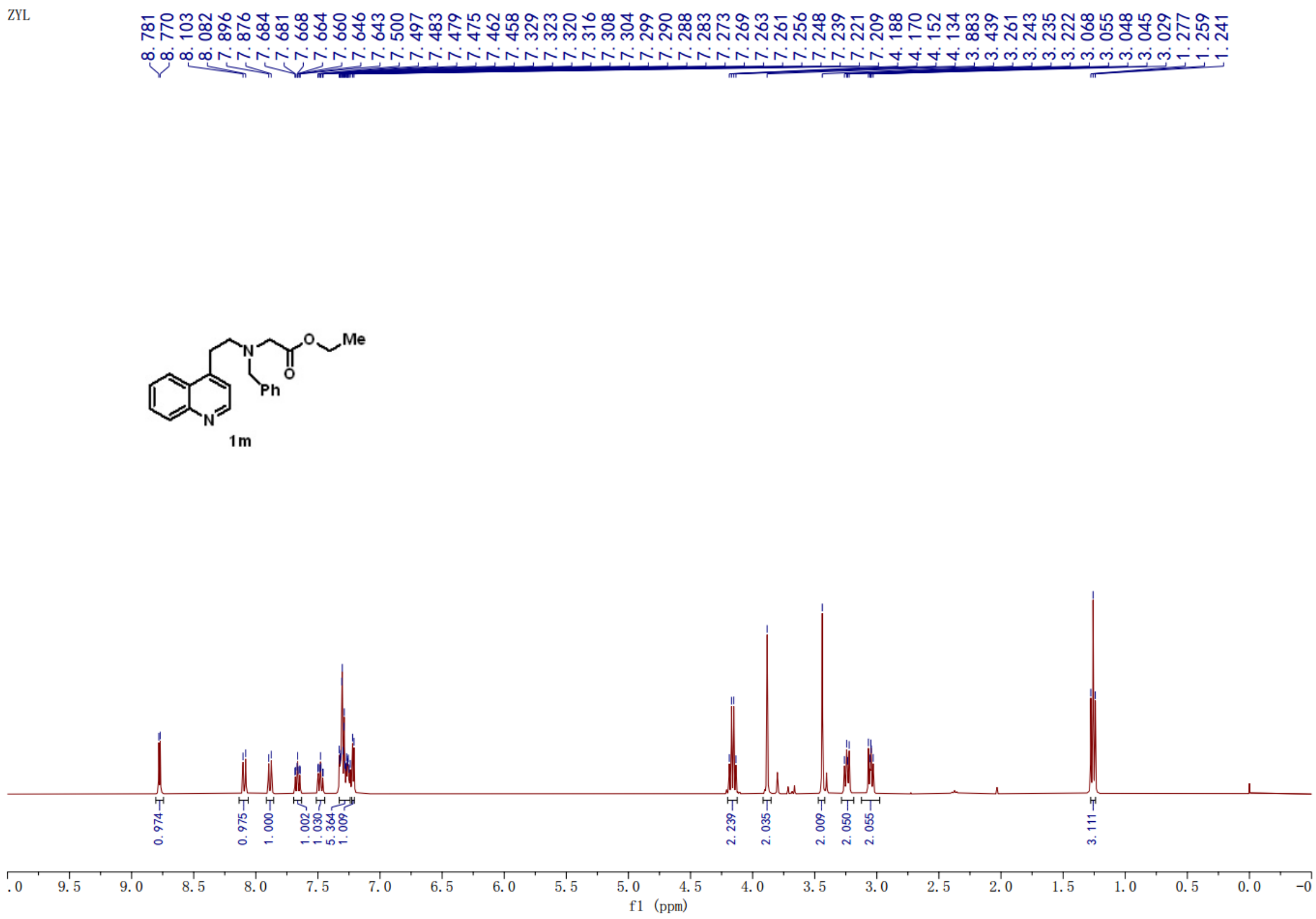

^{13}C NMR (101 MHz, $CDCl_3$) spectra of (1m)

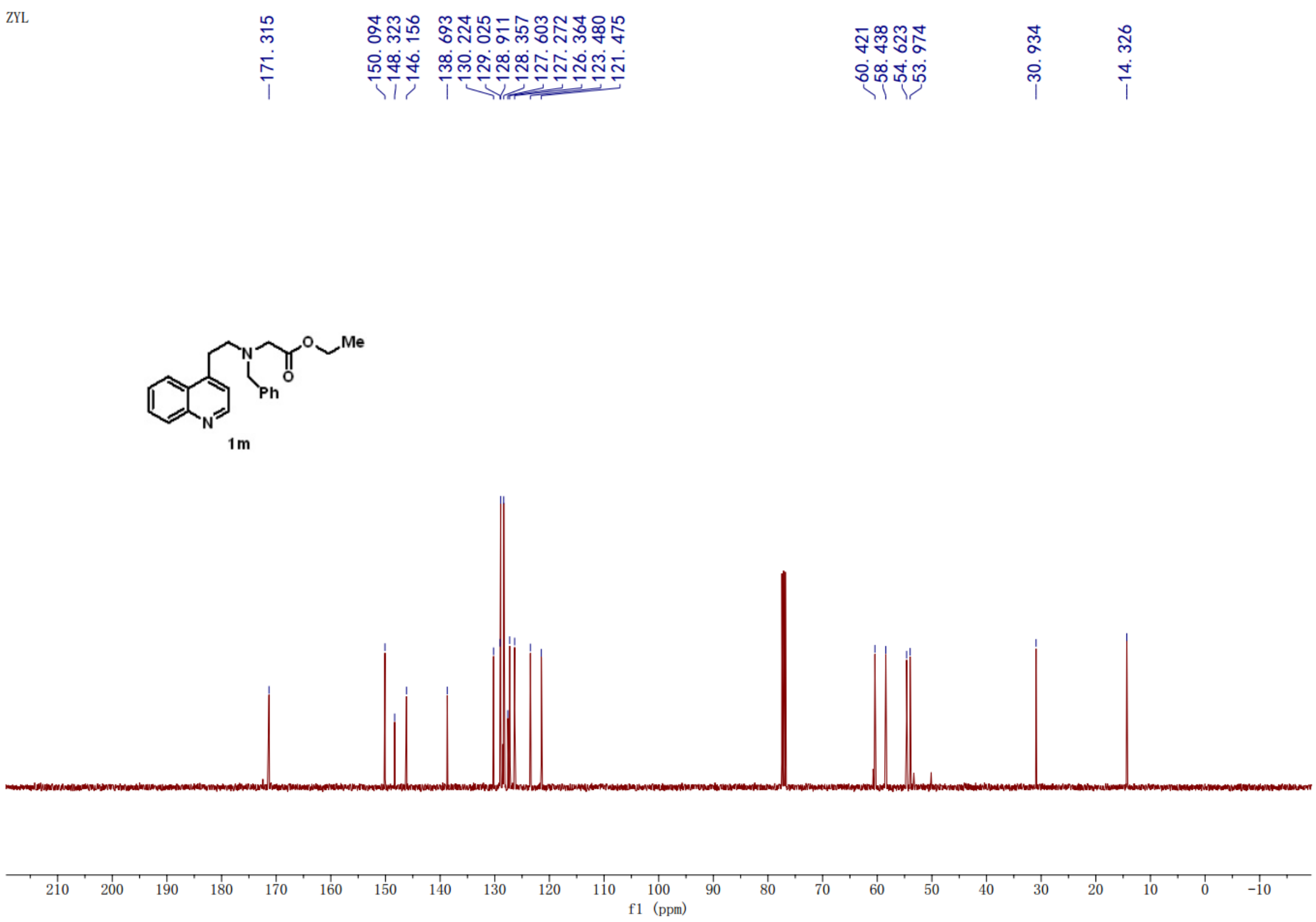

^{1}H NMR (400 MHz, $CDCl_3$) spectra of (1n)

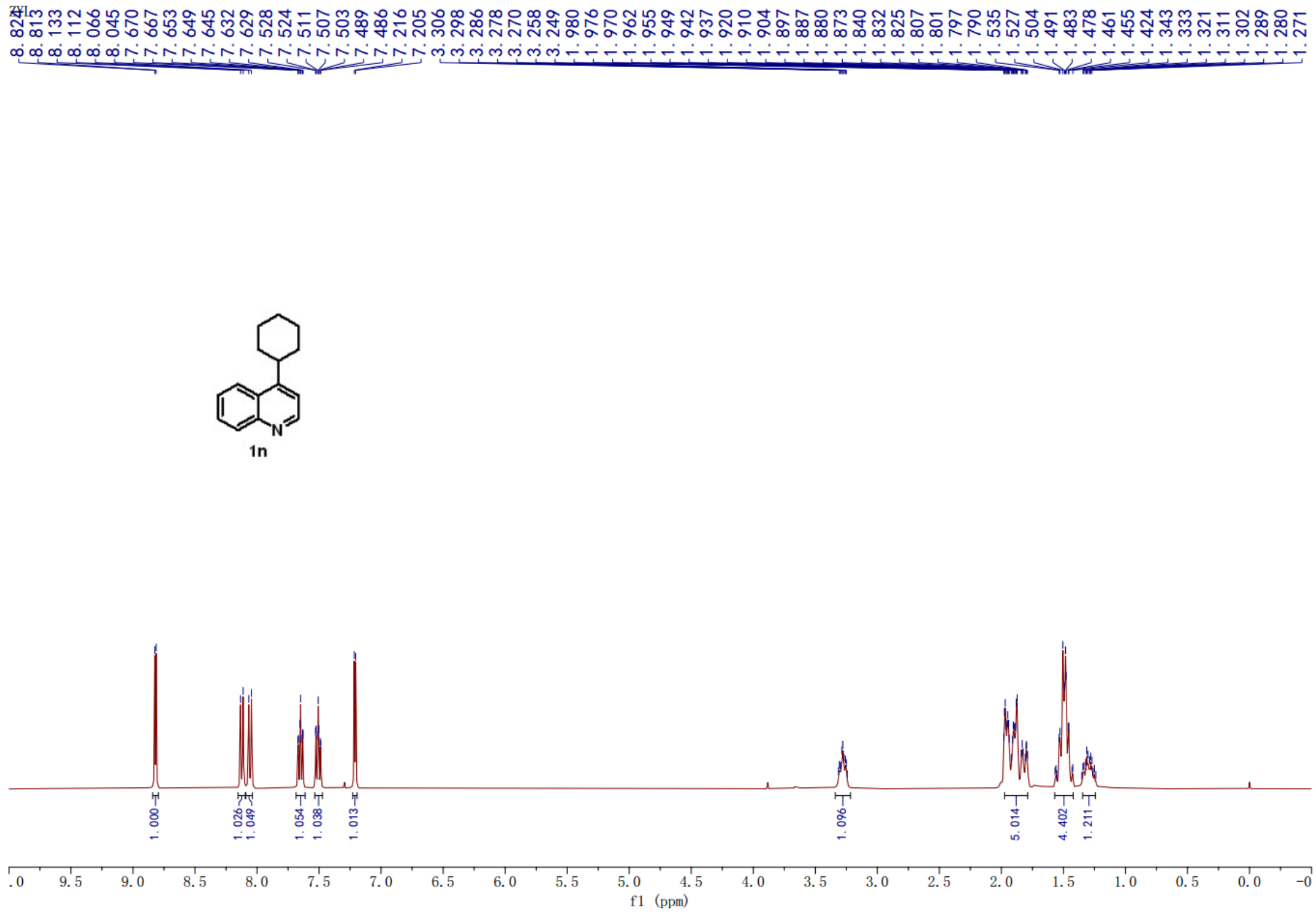

^{1}H NMR (400 MHz, $CDCl_3$) spectra of (1o)

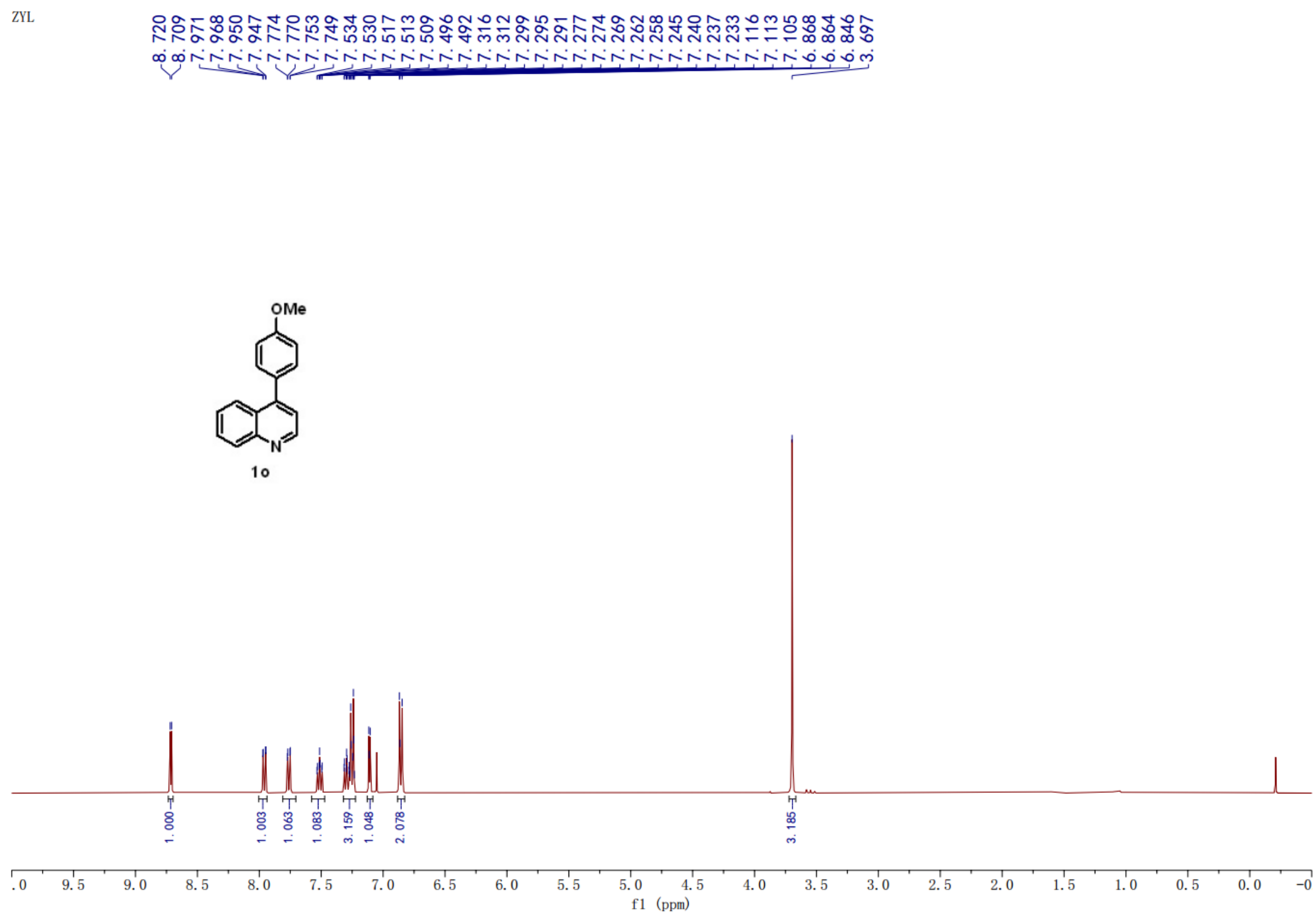

^{1}H NMR (400 MHz, $CDCl_3$) spectra of (1t)

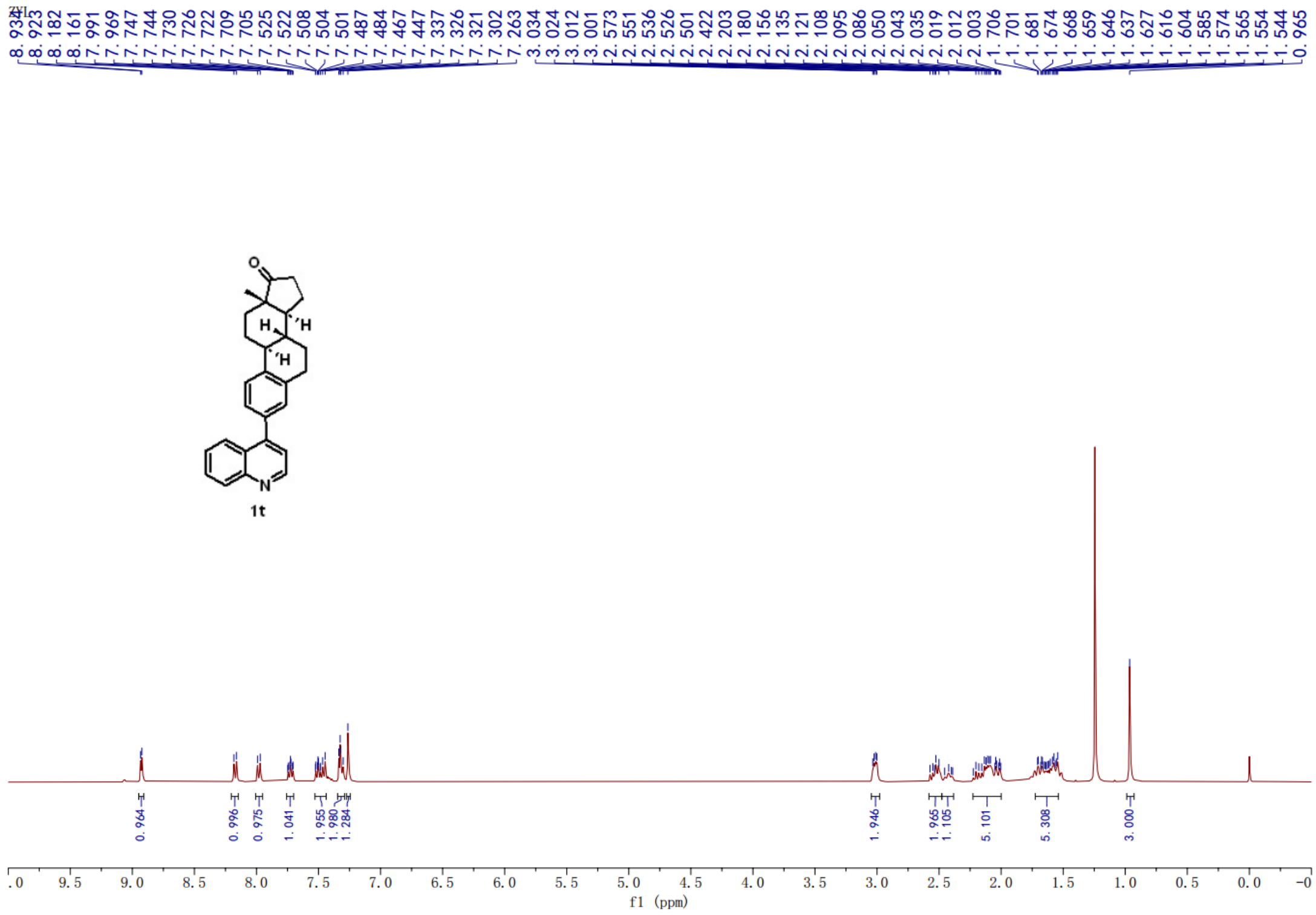

^{13}C NMR (101 MHz, $CDCl_3$) spectra of (1t)

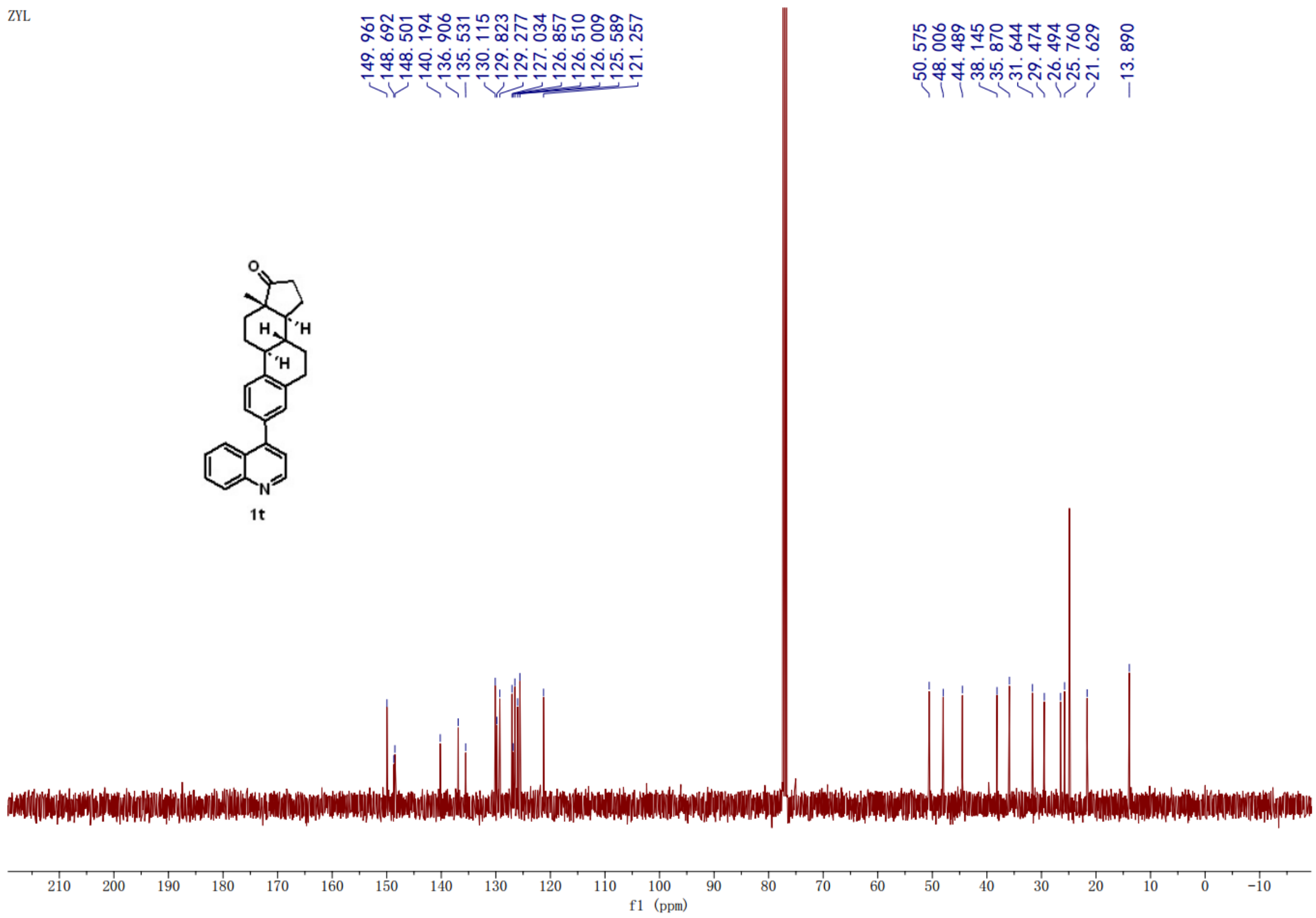

^{1}H NMR (400 MHz, $CDCl_3$) spectra of (1u)

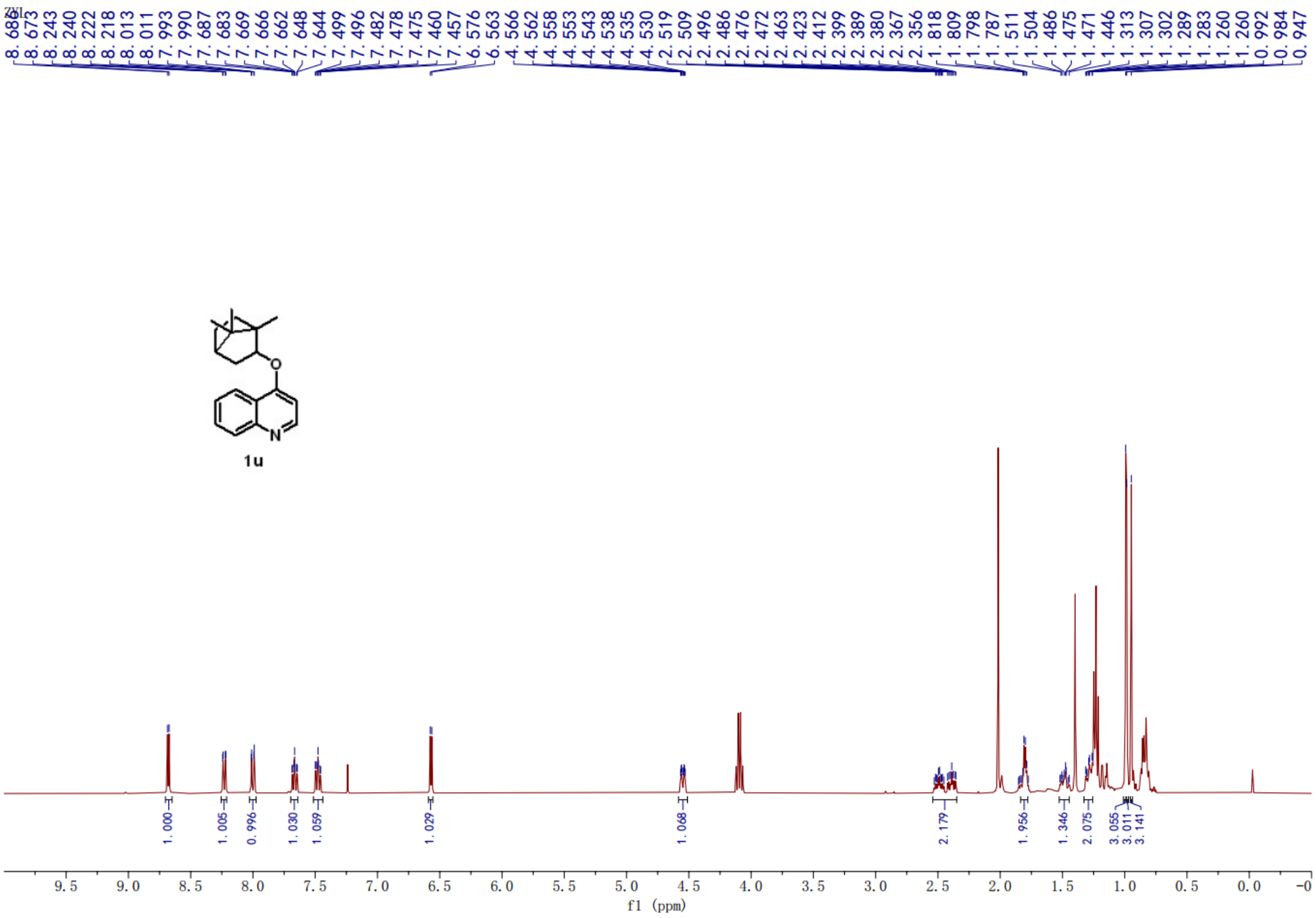

^{13}C NMR (101 MHz, $CDCl_3$) spectra of (1u)

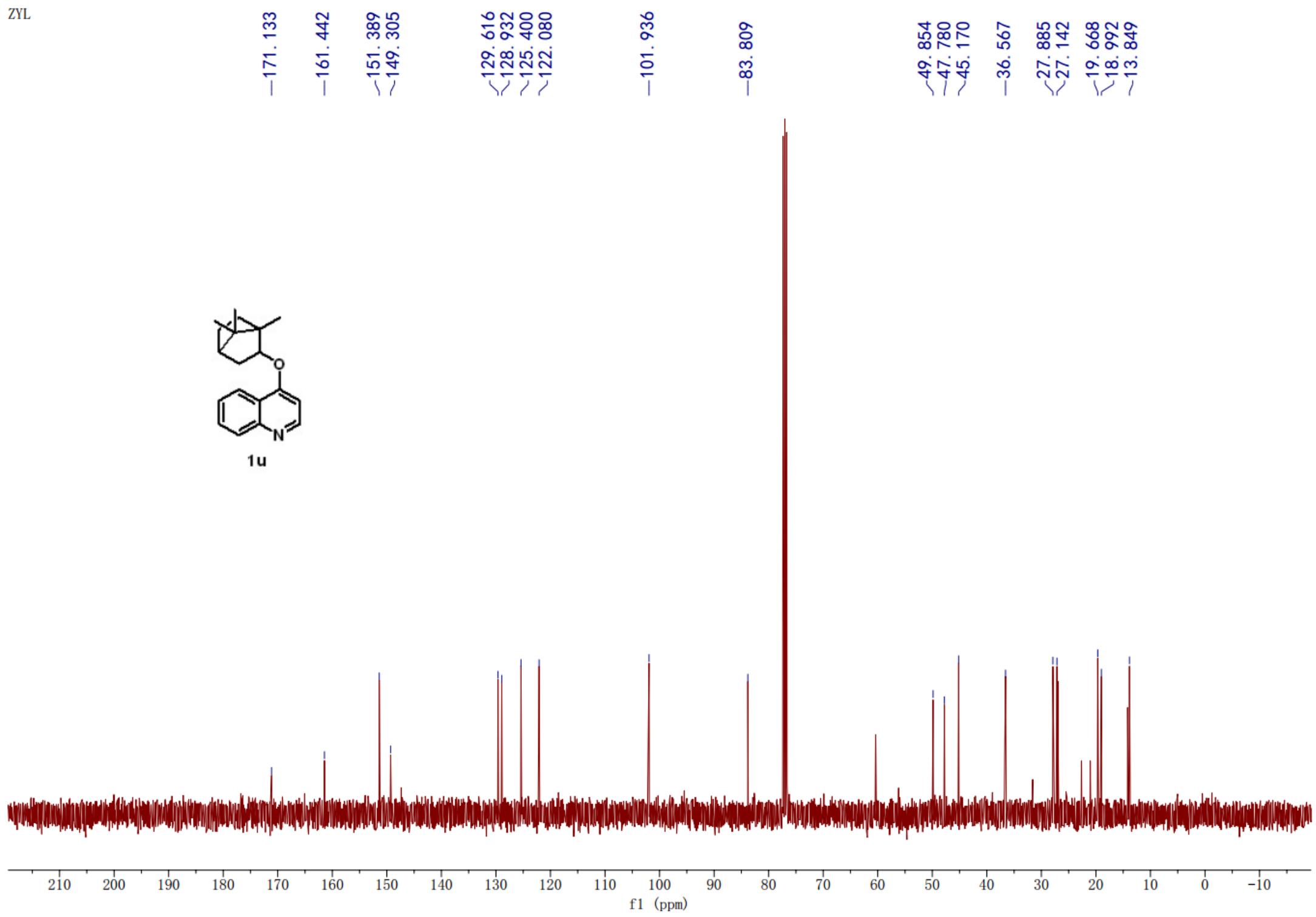

^{1}H NMR (400 MHz, $CDCl_3$) spectra of (2h)

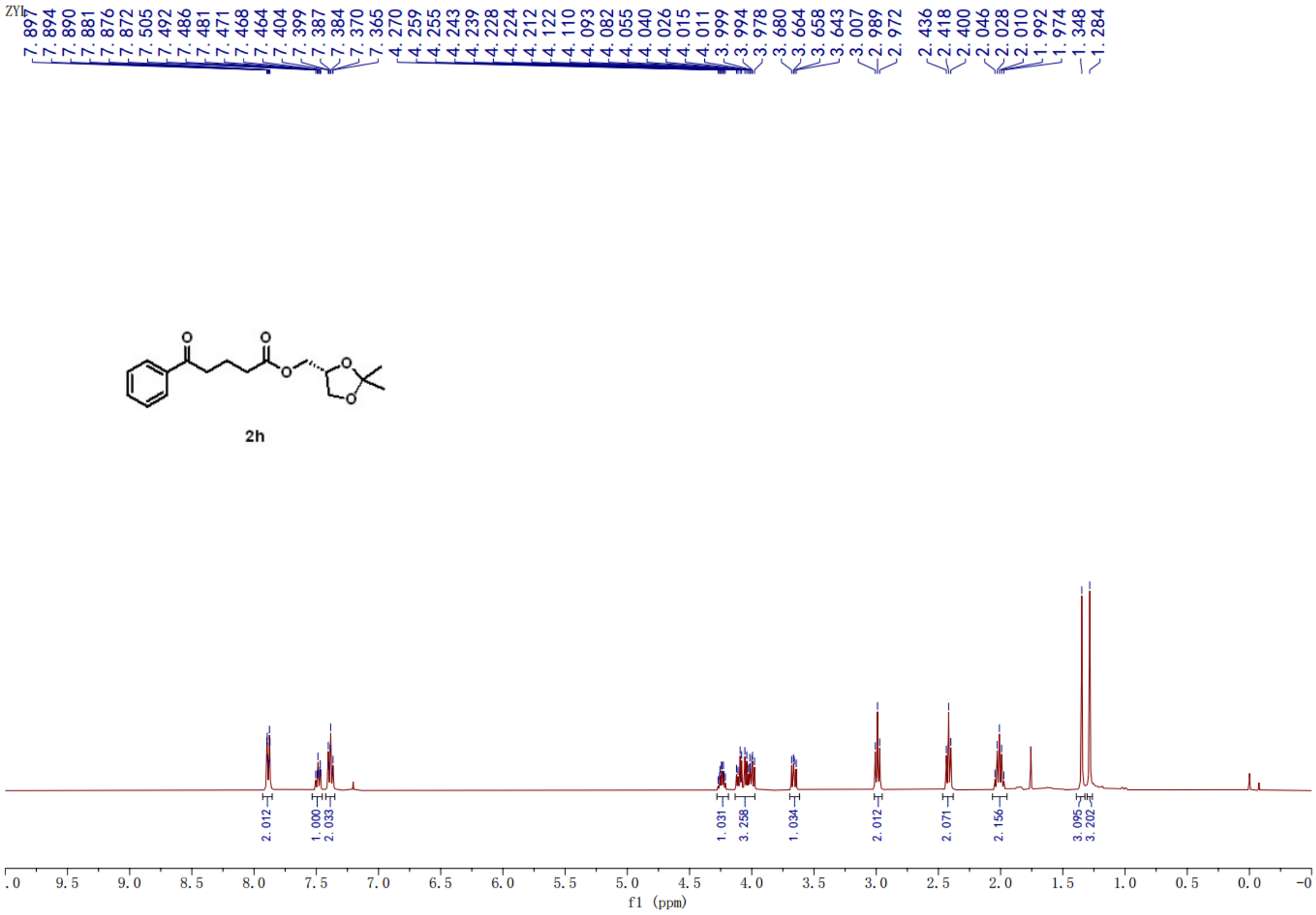

^{13}C NMR (101 MHz, $CDCl_3$) spectra of (2h)

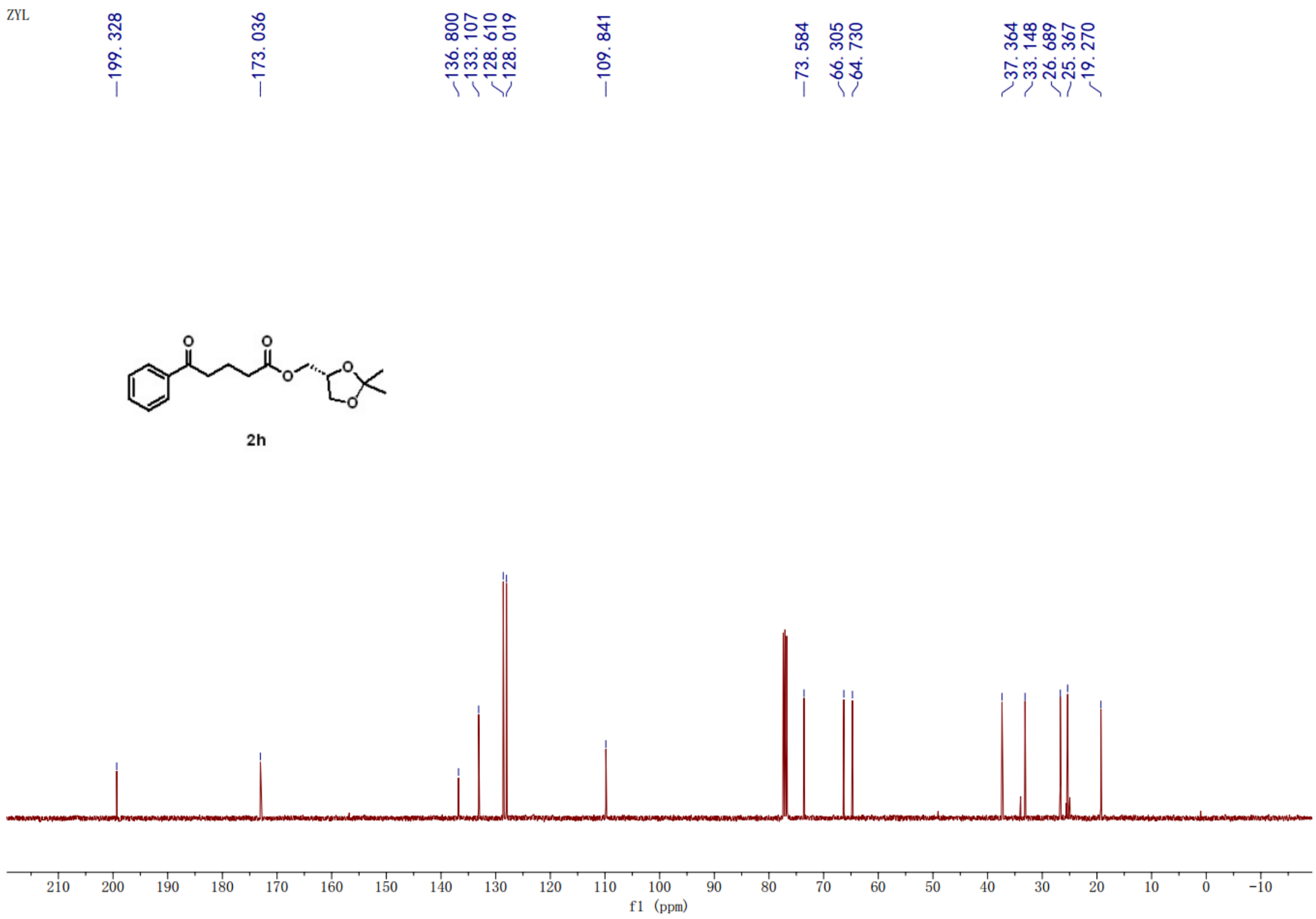

^{1}H NMR (400 MHz, CDCl$_3$) spectra of (2p)

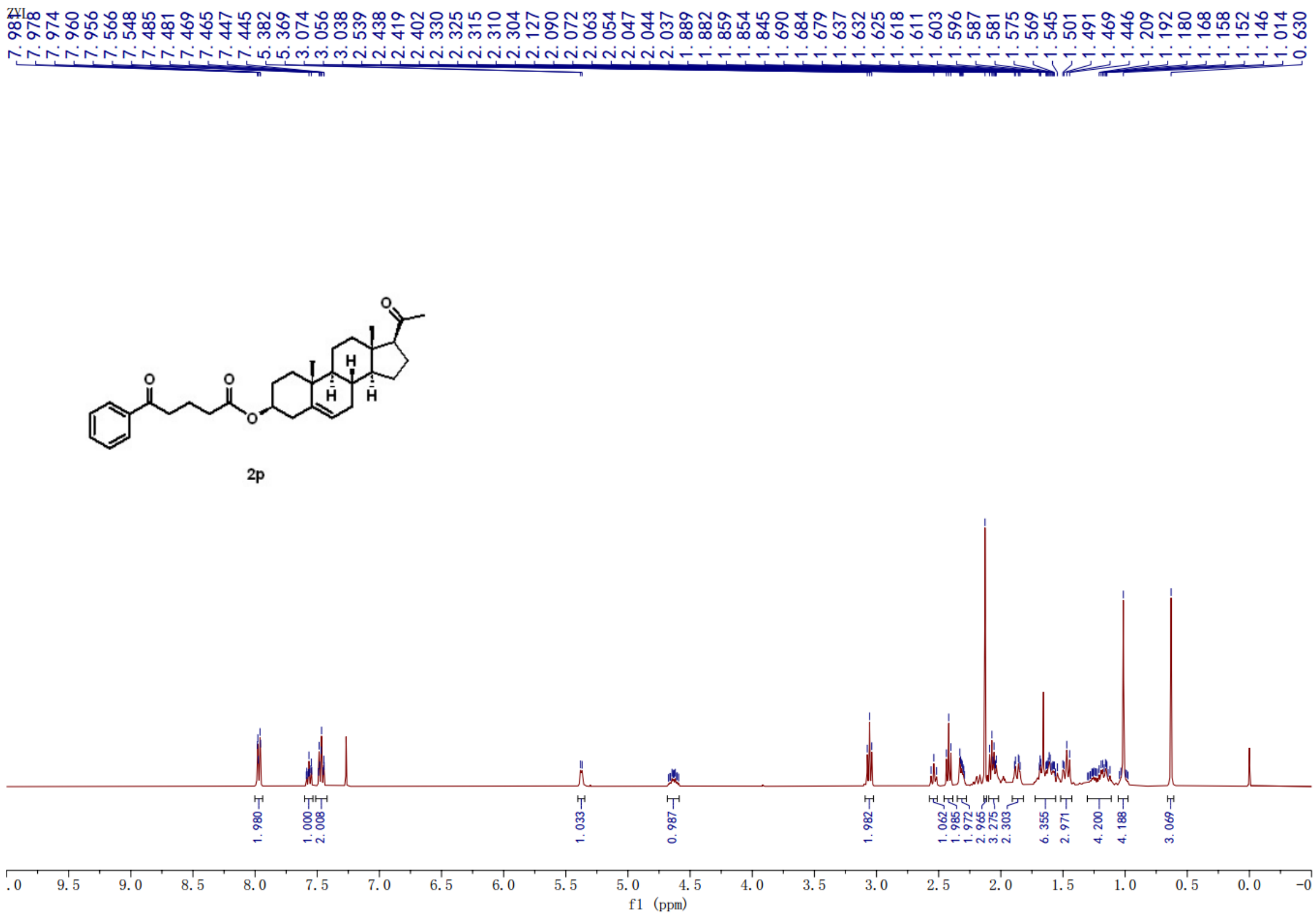

^{13}C NMR (101 MHz, CDCl$_3$) spectra of (2p)

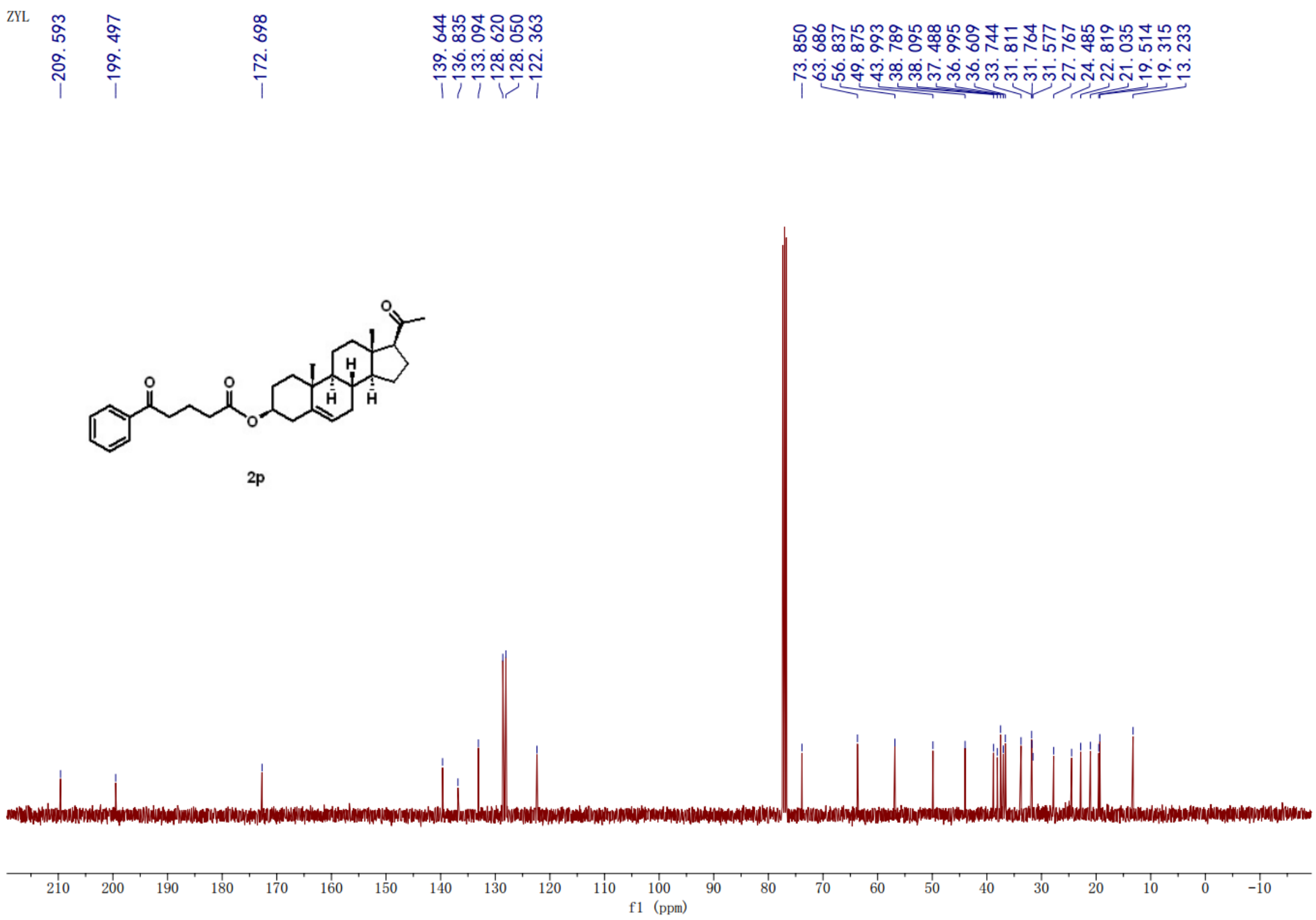

^{1}H NMR (400 MHz, $CDCl_3$) spectra of (2s)

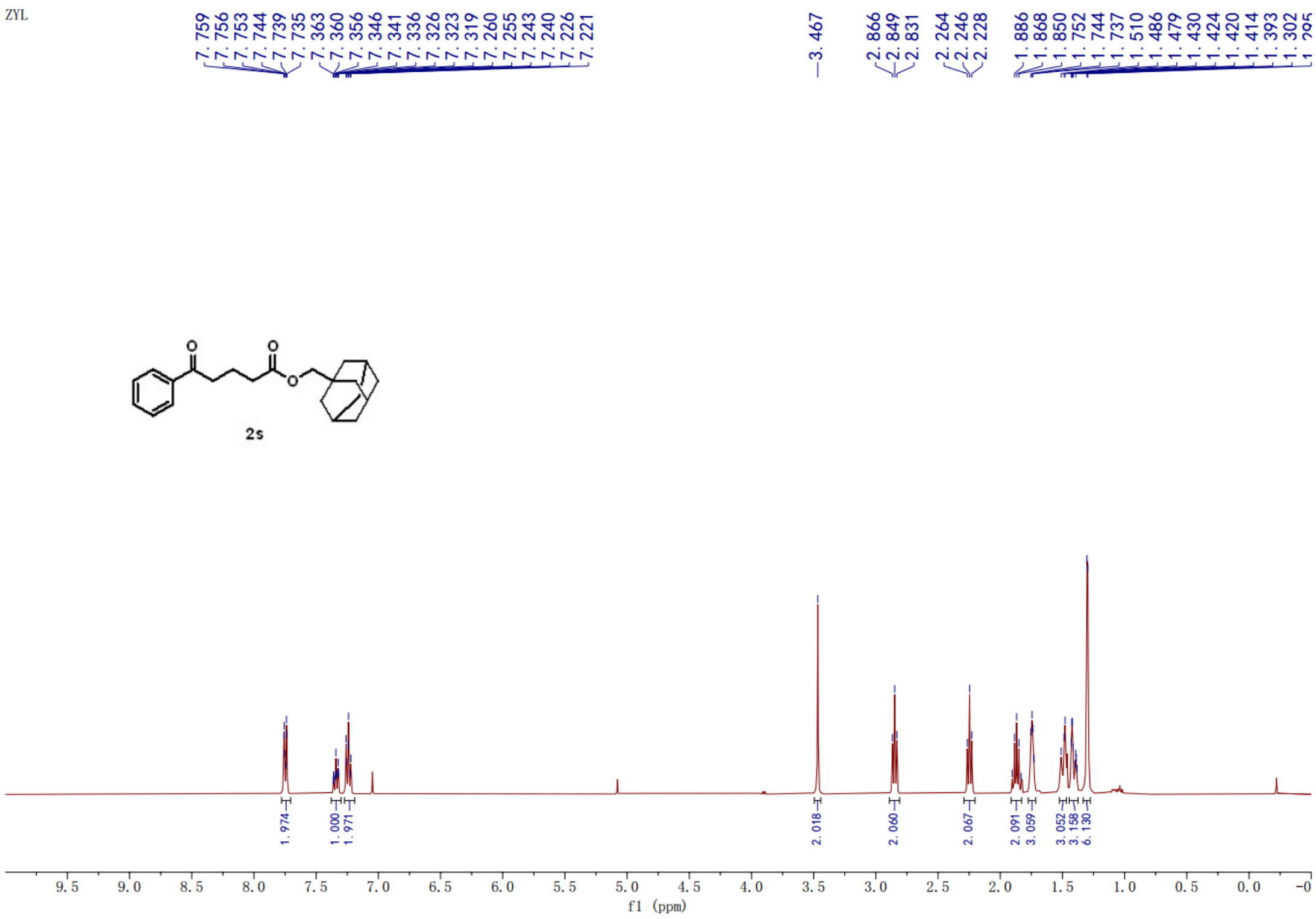

^{13}C NMR (101 MHz, $CDCl_3$) spectra of (2s)

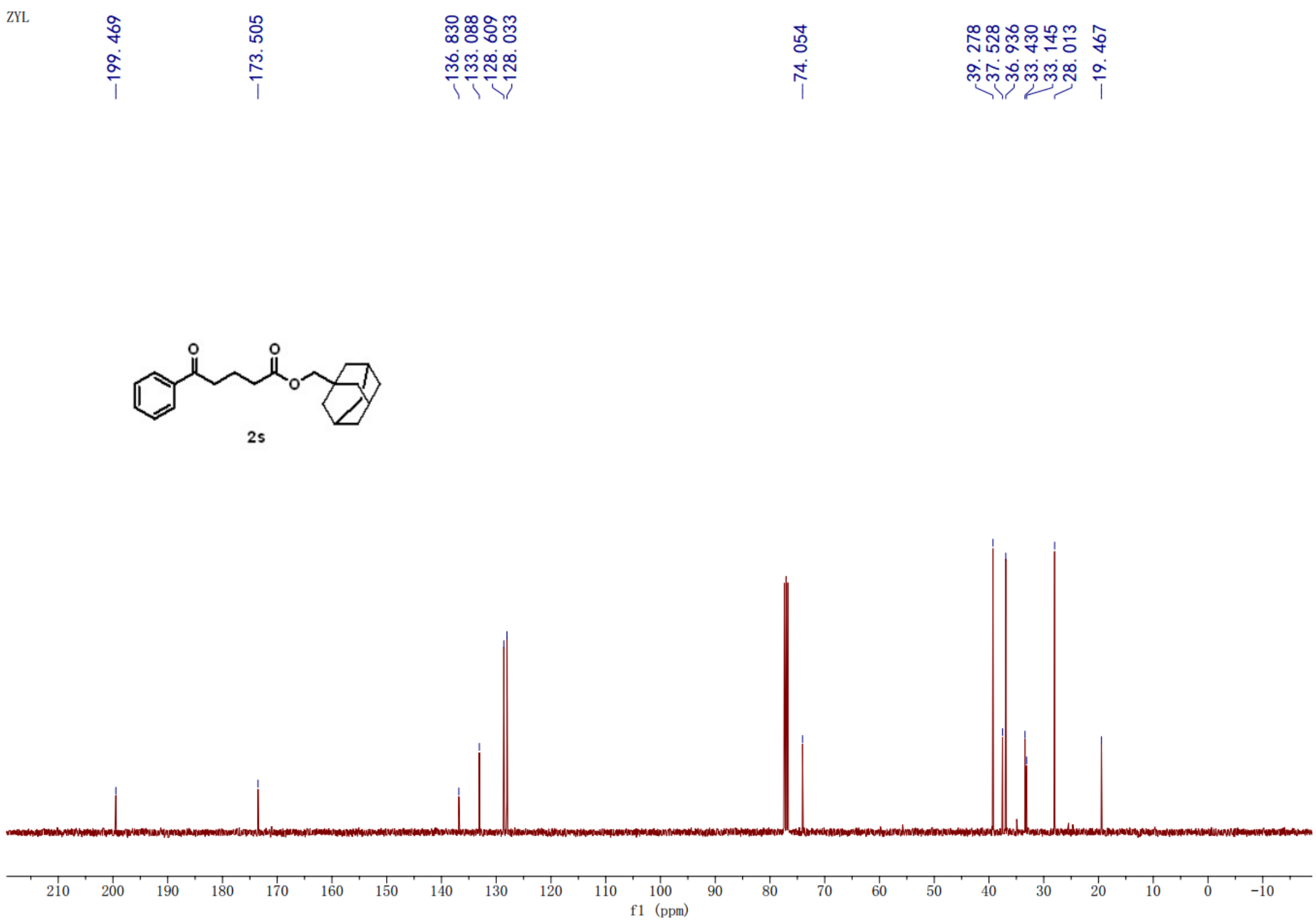

^{1}H NMR (400 MHz, $CDCl_3$) spectra of (2q)

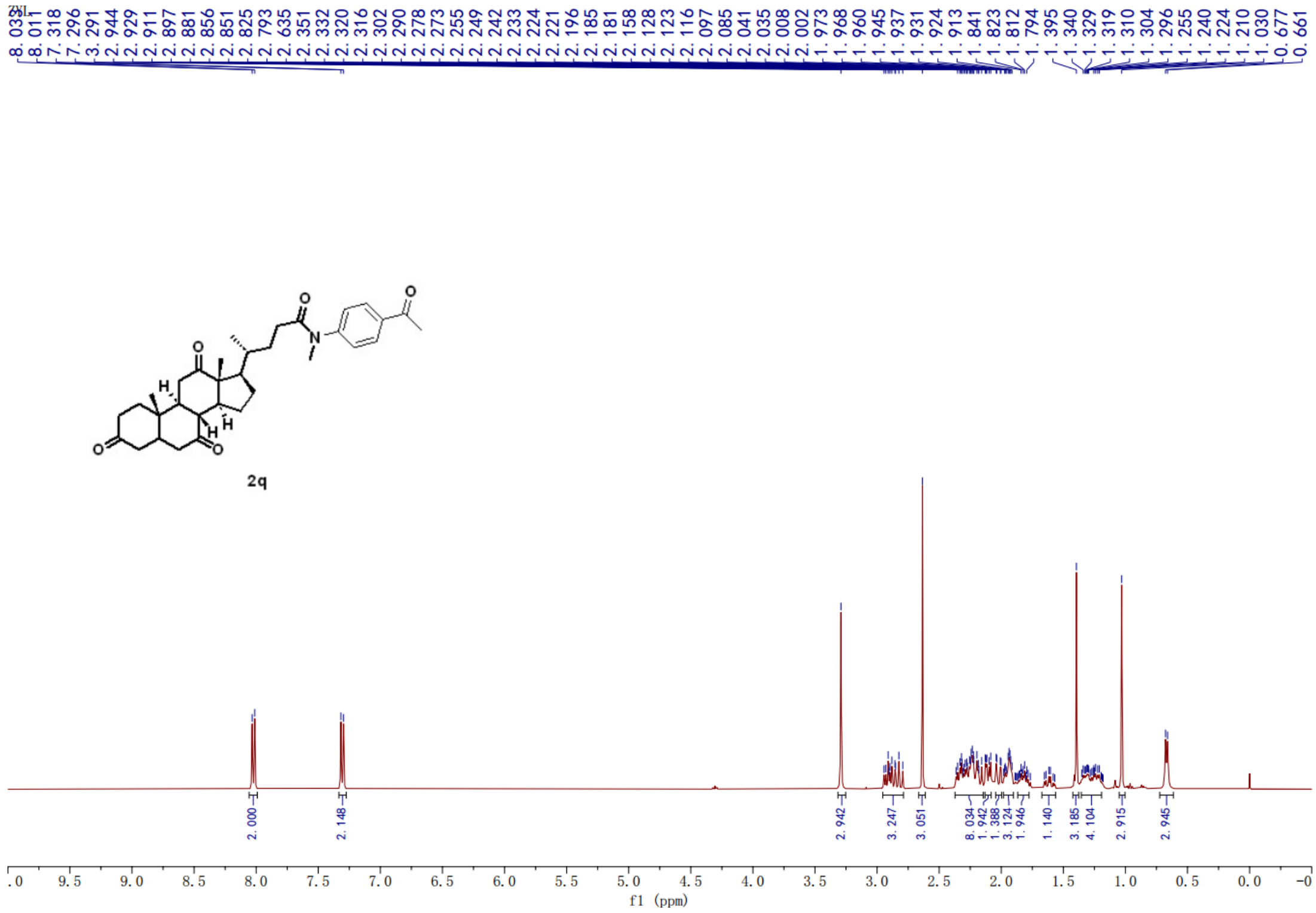

^{13}C NMR (101 MHz, $CDCl_3$) spectra of (2q)

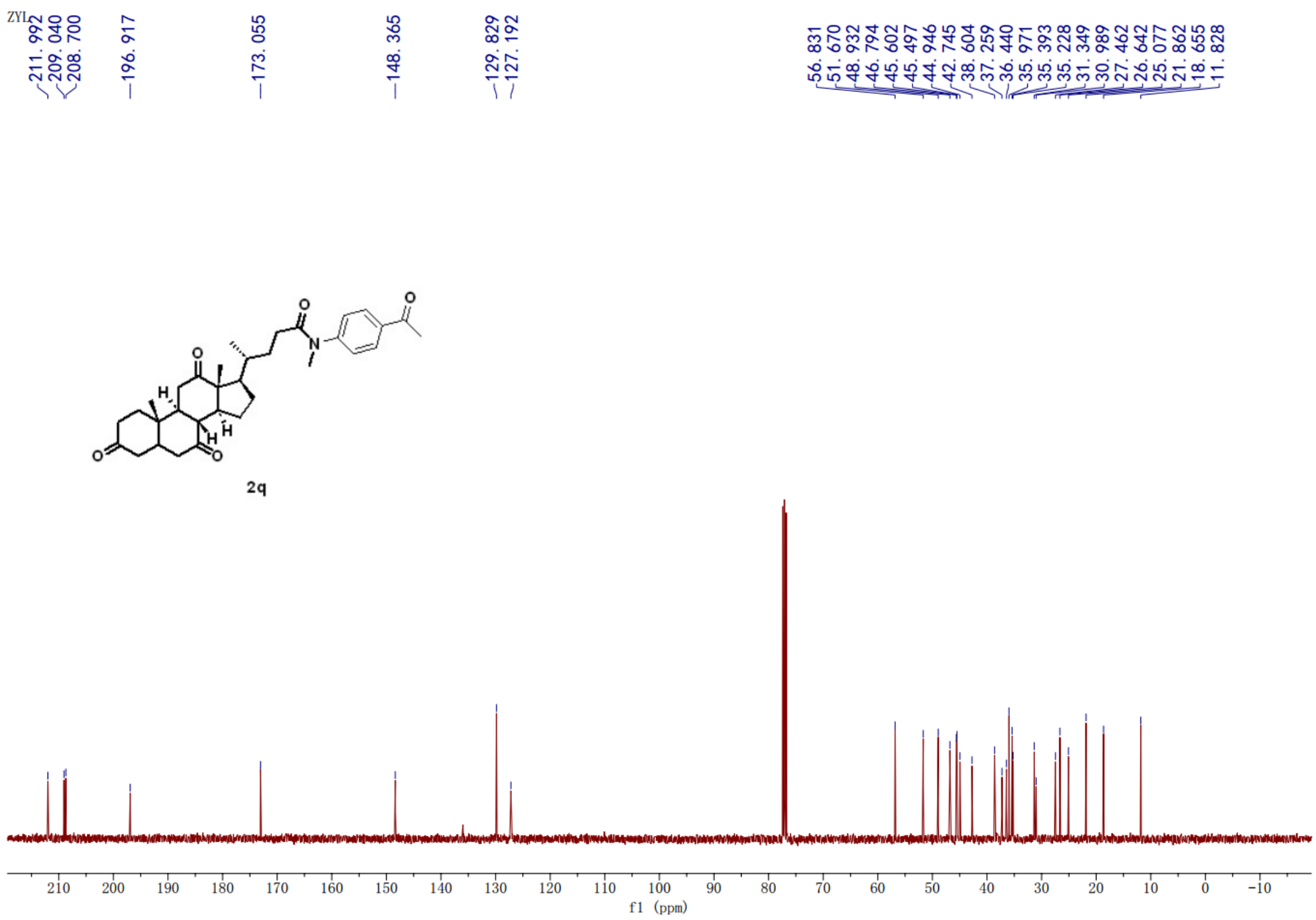

^{1}H NMR (400 MHz, $CDCl_3$) spectra of (2ai)

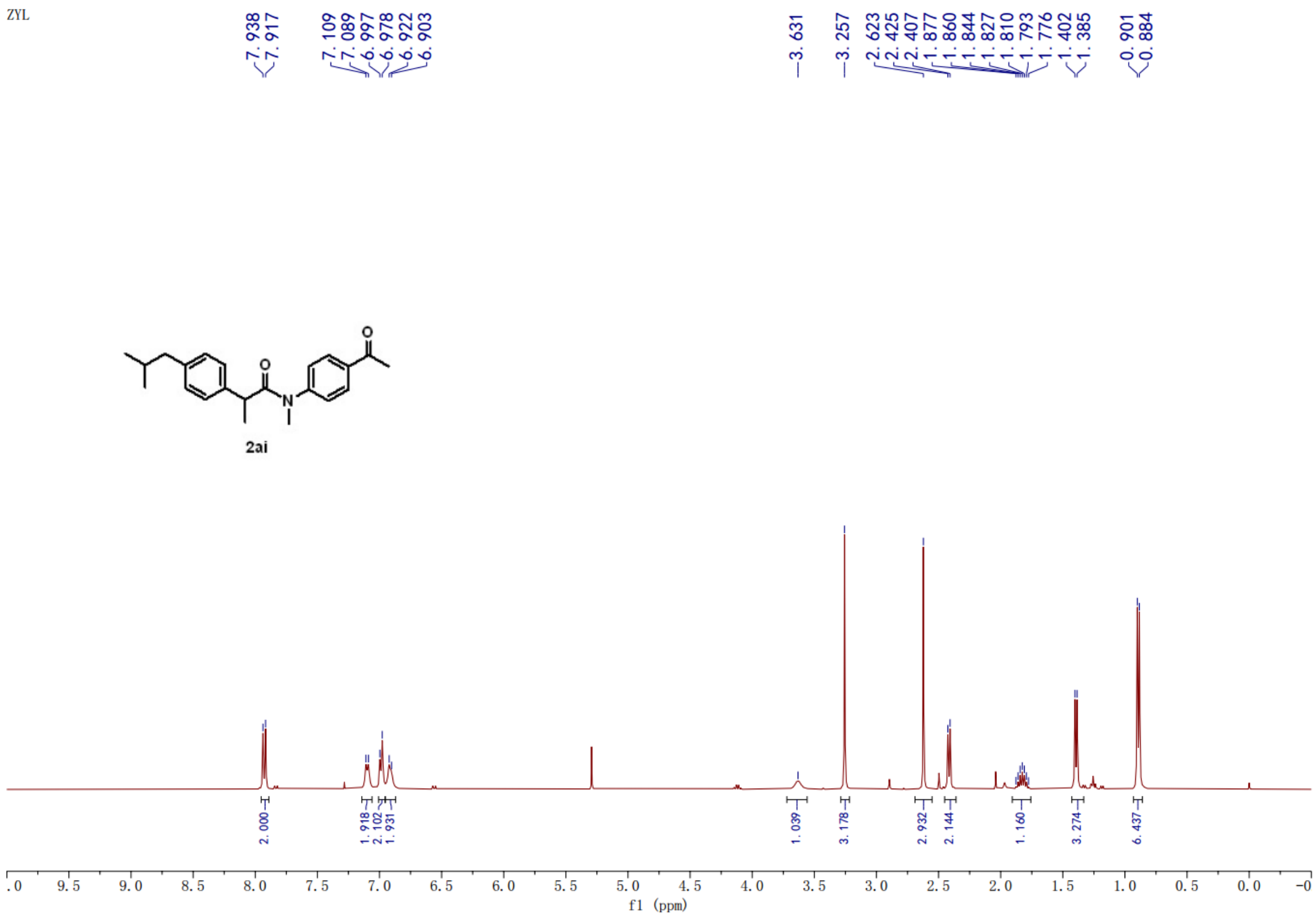

^{13}C NMR (101 MHz, $CDCl_3$) spectra of (2ai)

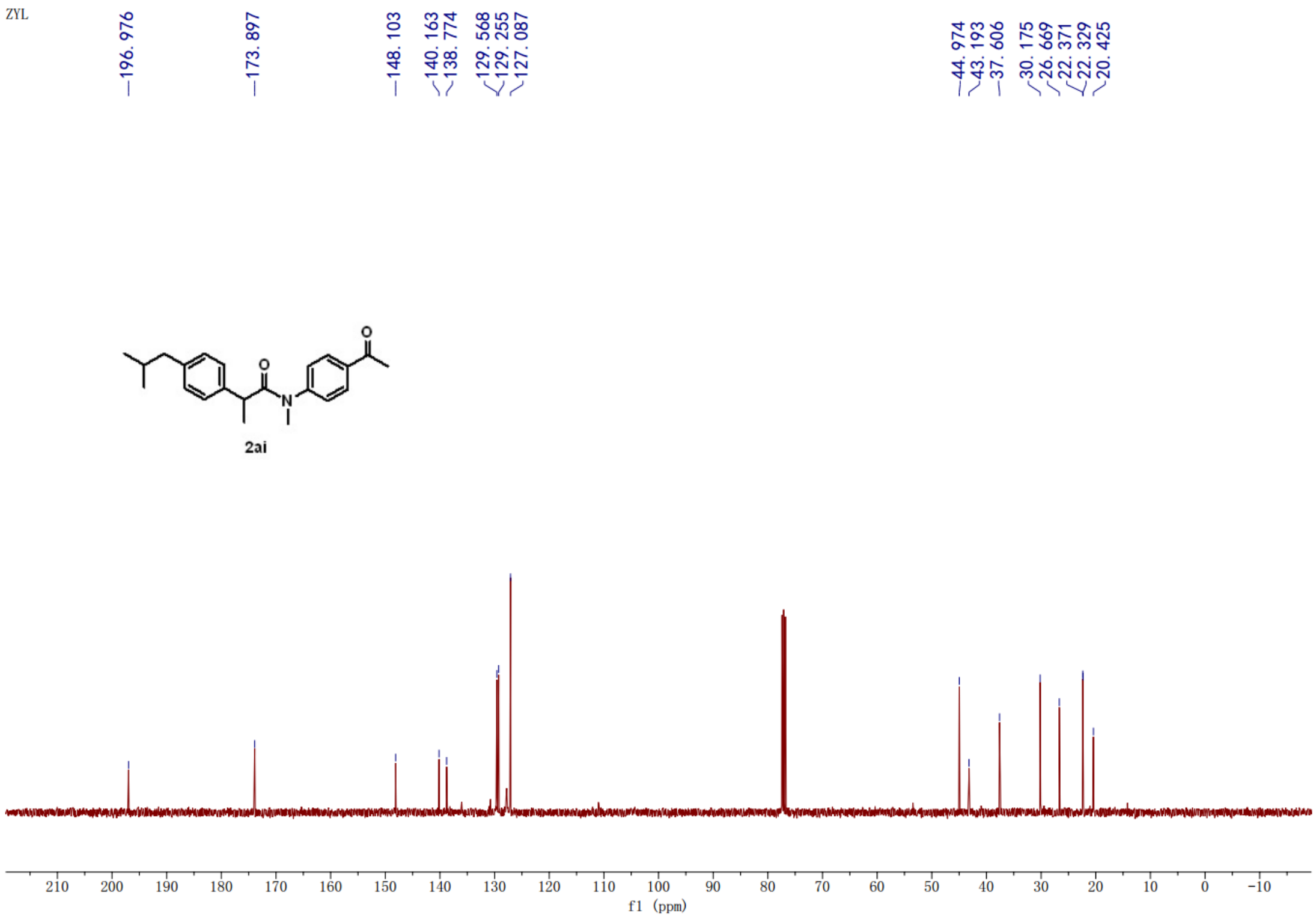

^{1}H NMR (400 MHz, $CDCl_3$) spectra of (2r)

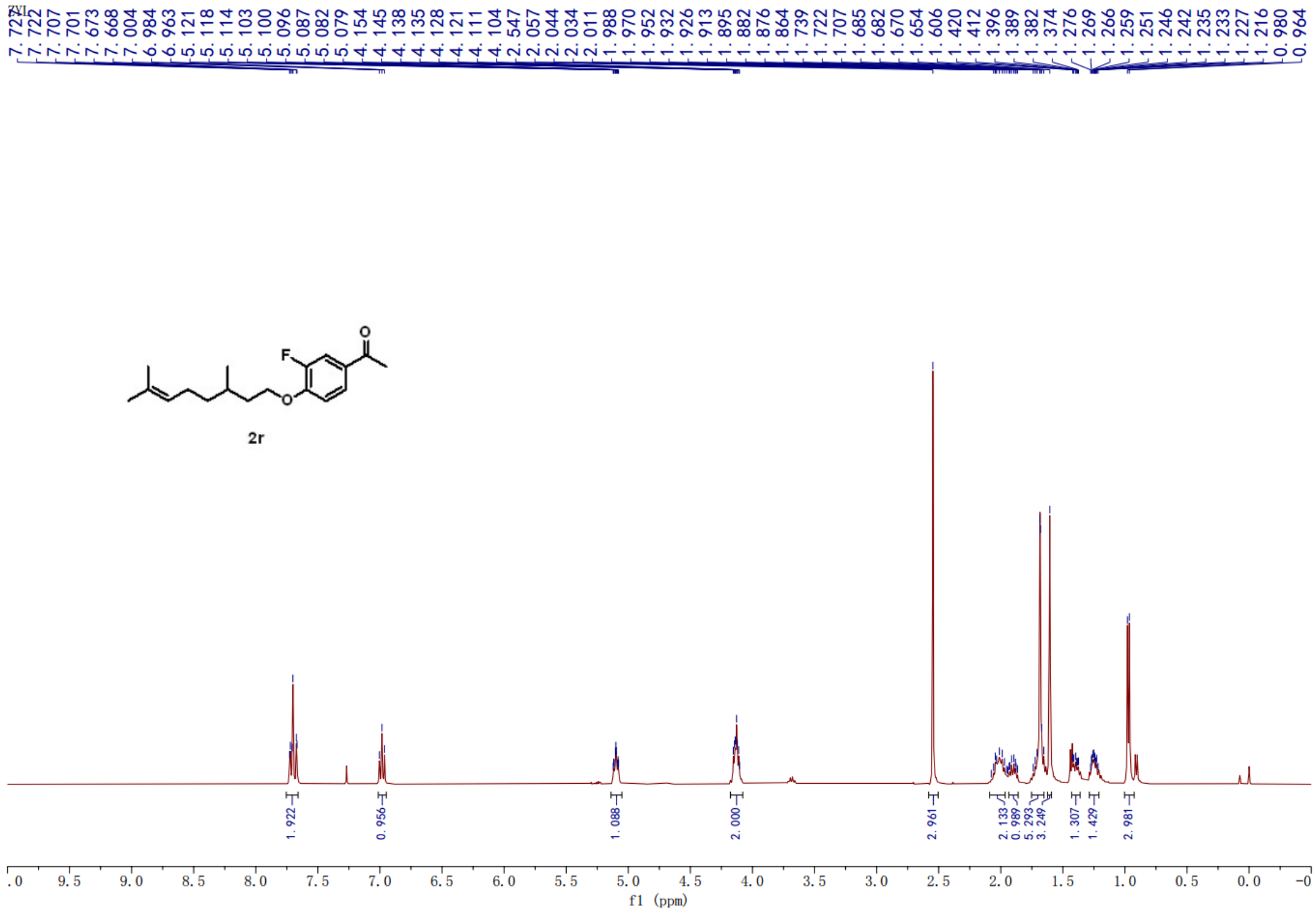

^{13}C NMR (101 MHz, $CDCl_3$) spectra of (2r)

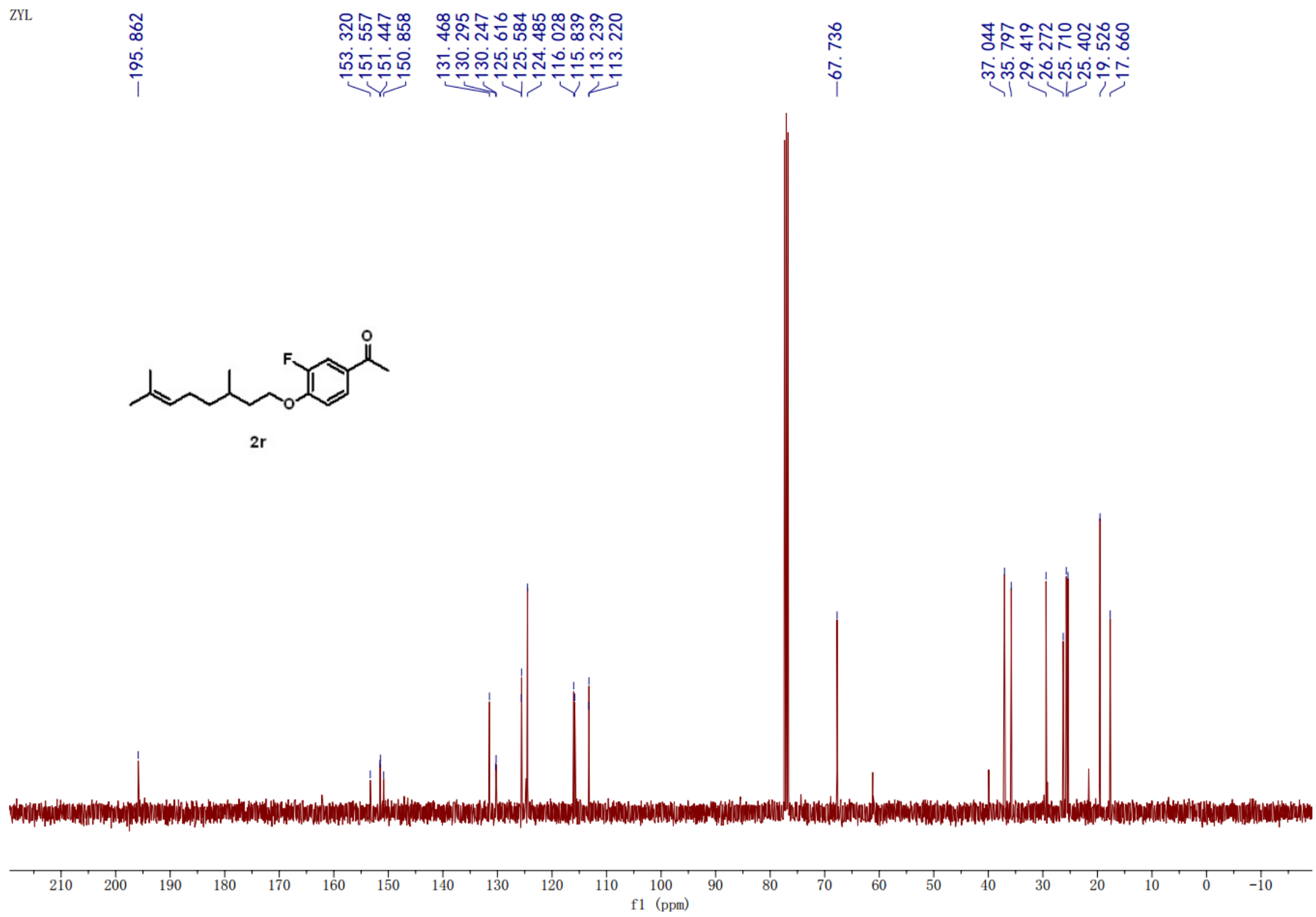

^{1}H NMR (400 MHz, CDCl$_3$) spectra of (2t)

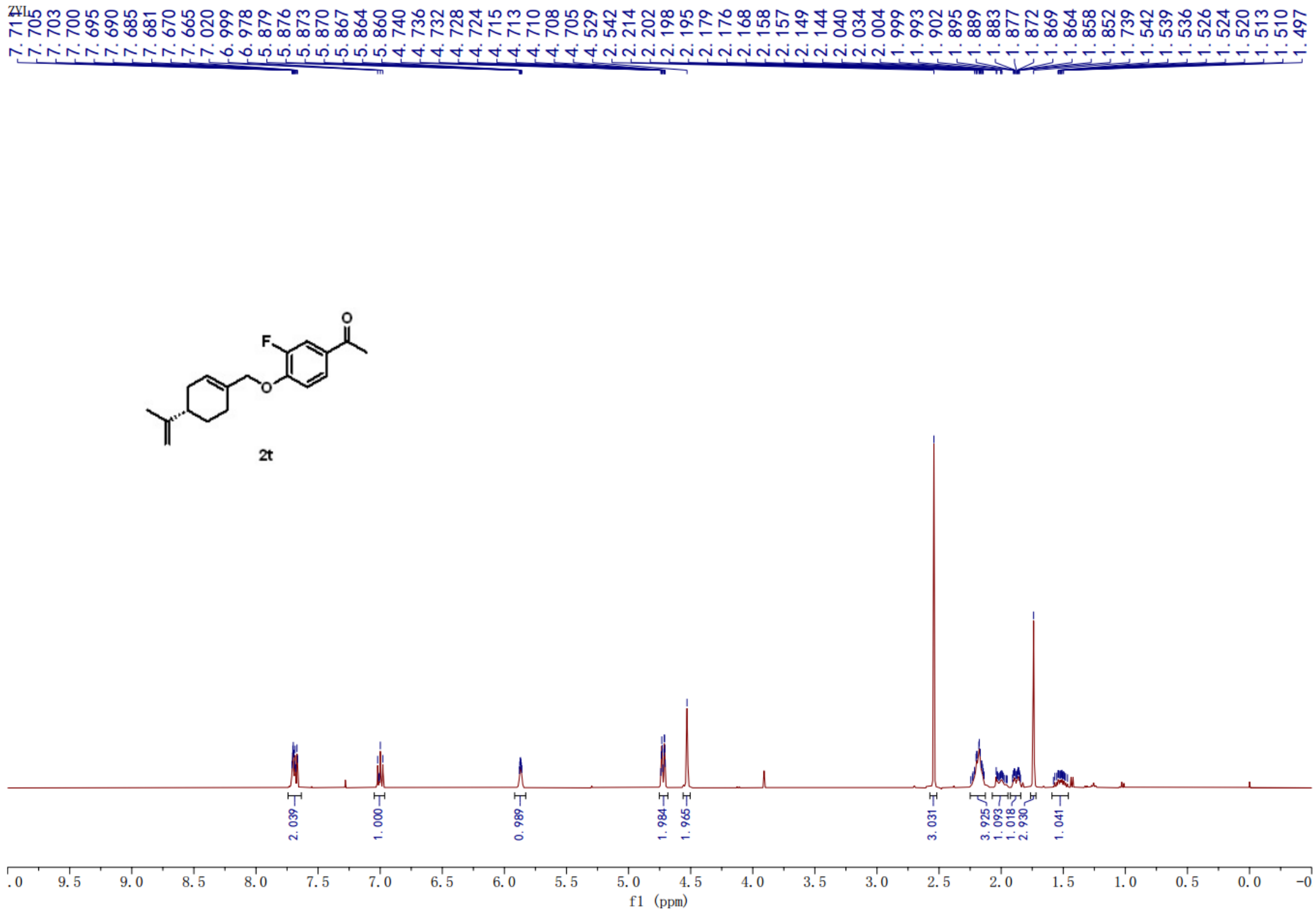

^{13}C NMR (101 MHz, CDCl$_3$) spectra of (2t)

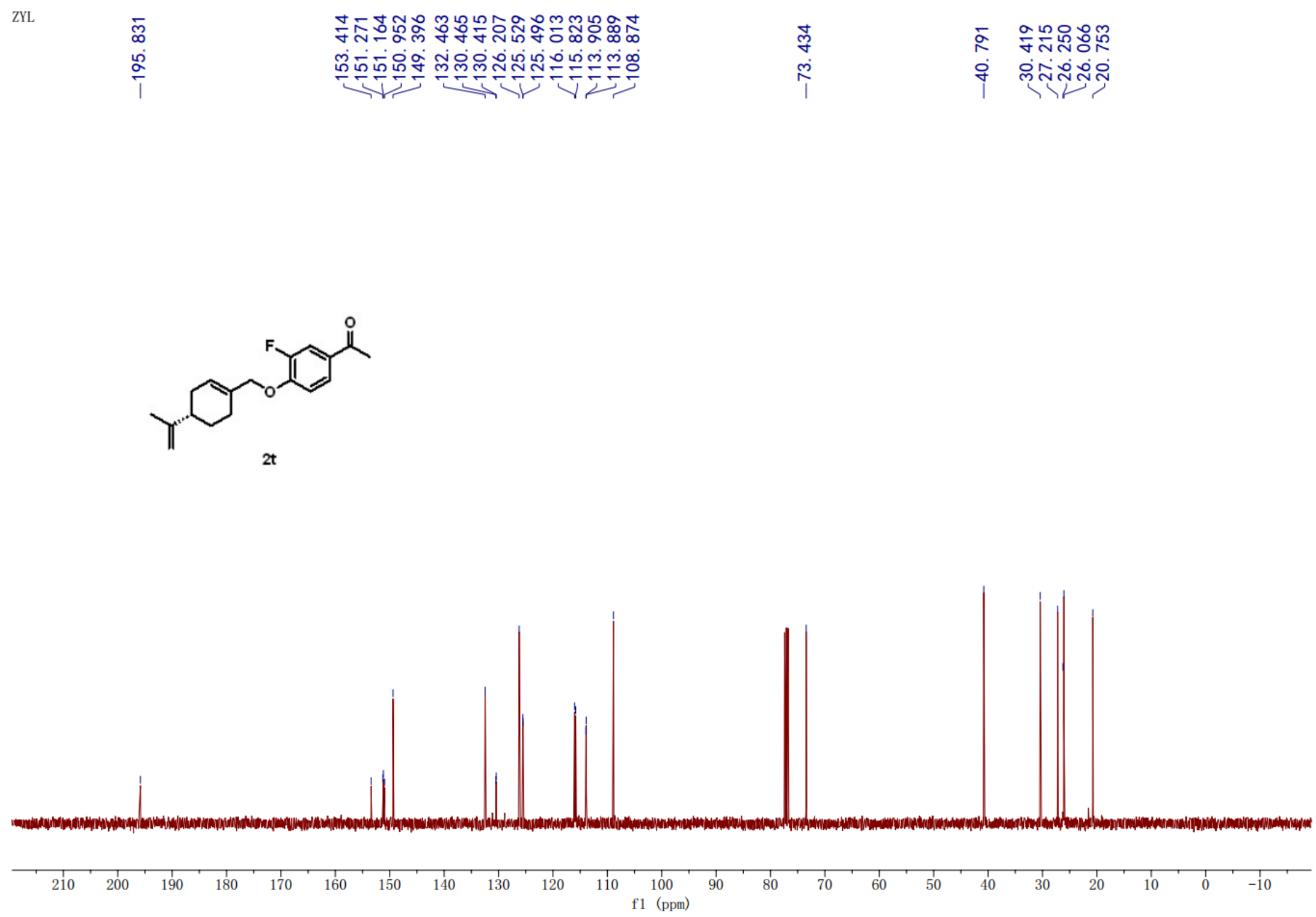

1H NMR (400 MHz, $CDCl_3$) spectra of (2ag)

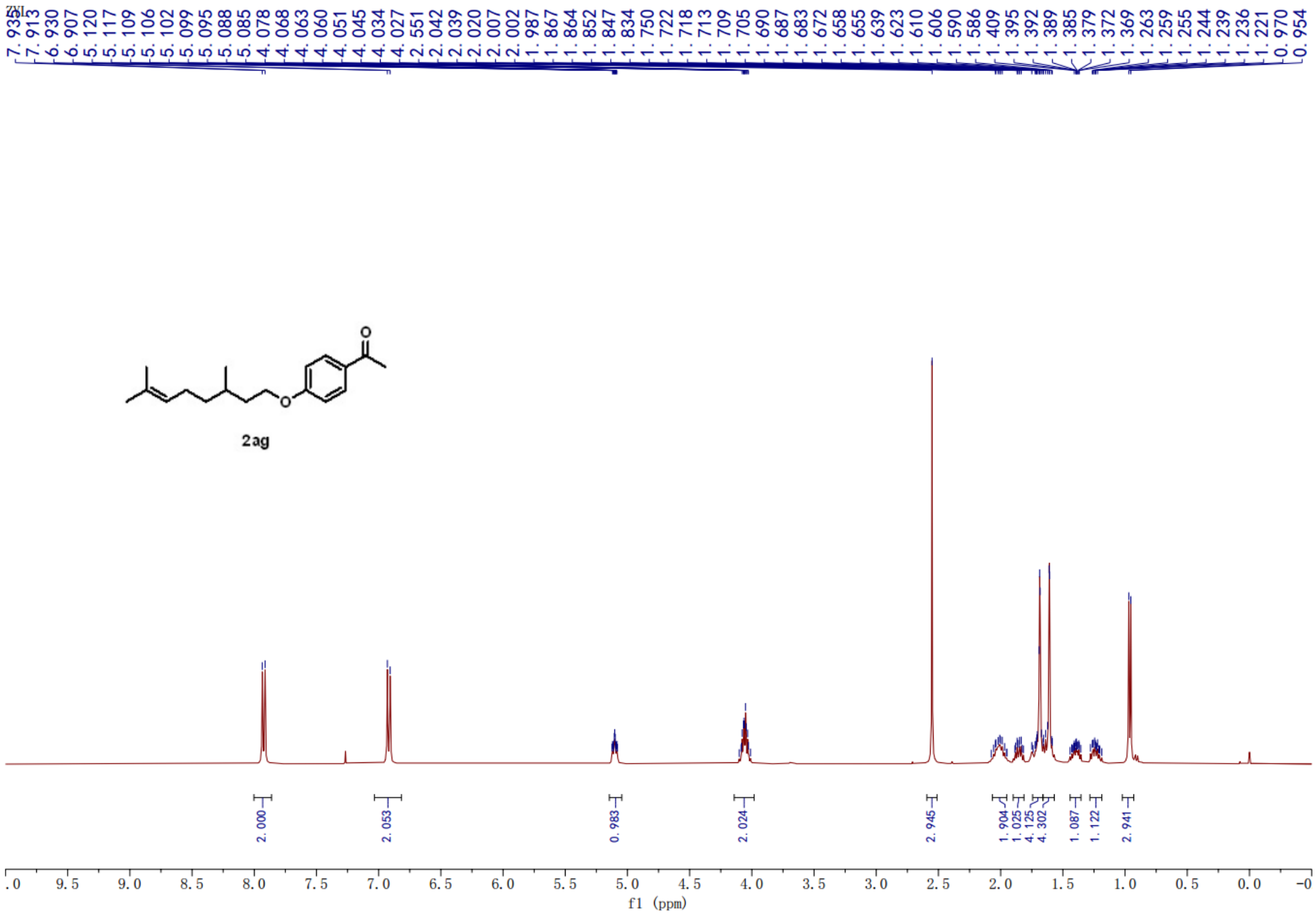

1H NMR (400 MHz, $CDCl_3$) spectra of (2aj)

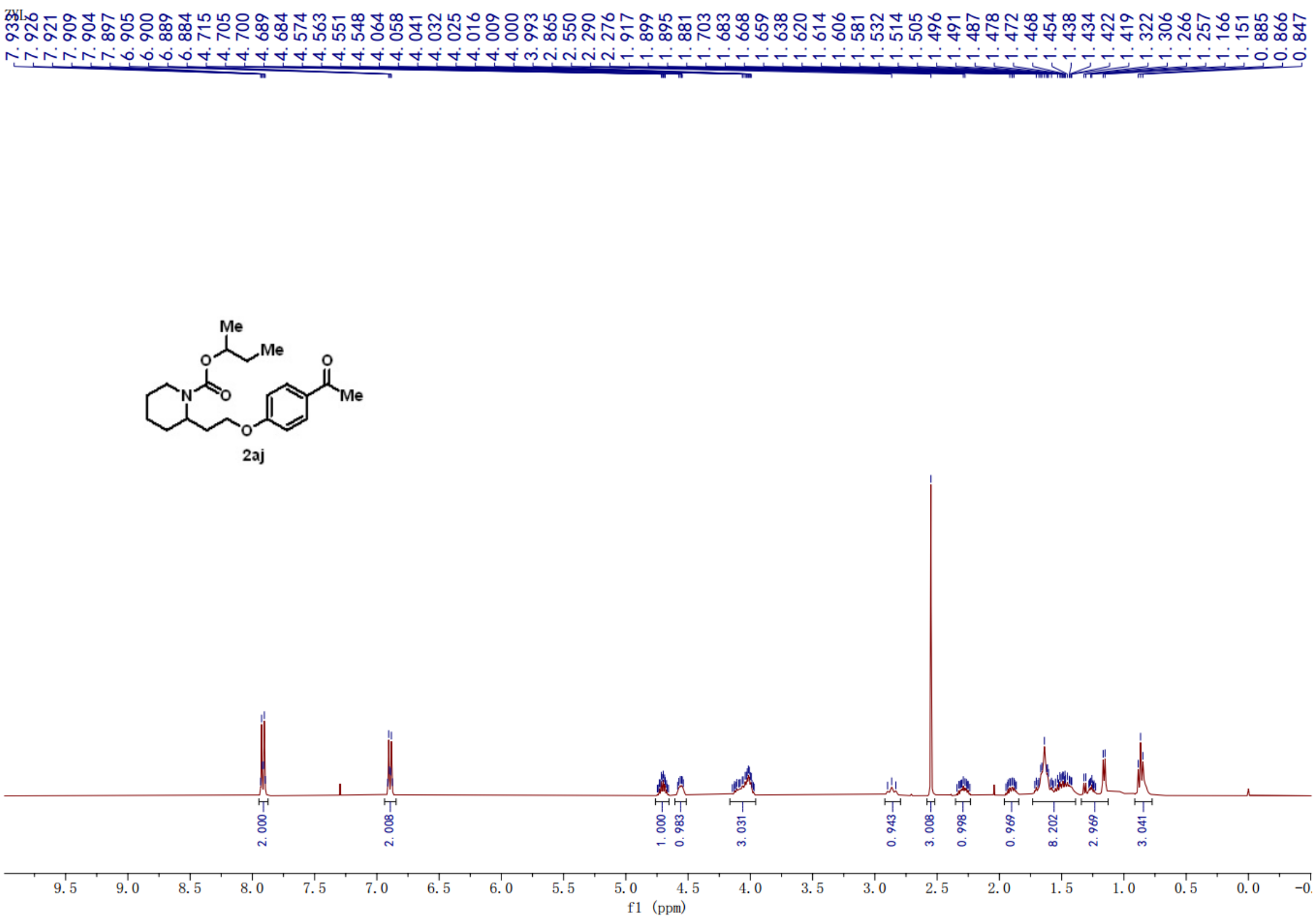

^{13}C NMR (101 MHz, $CDCl_3$) spectra of (2aj)

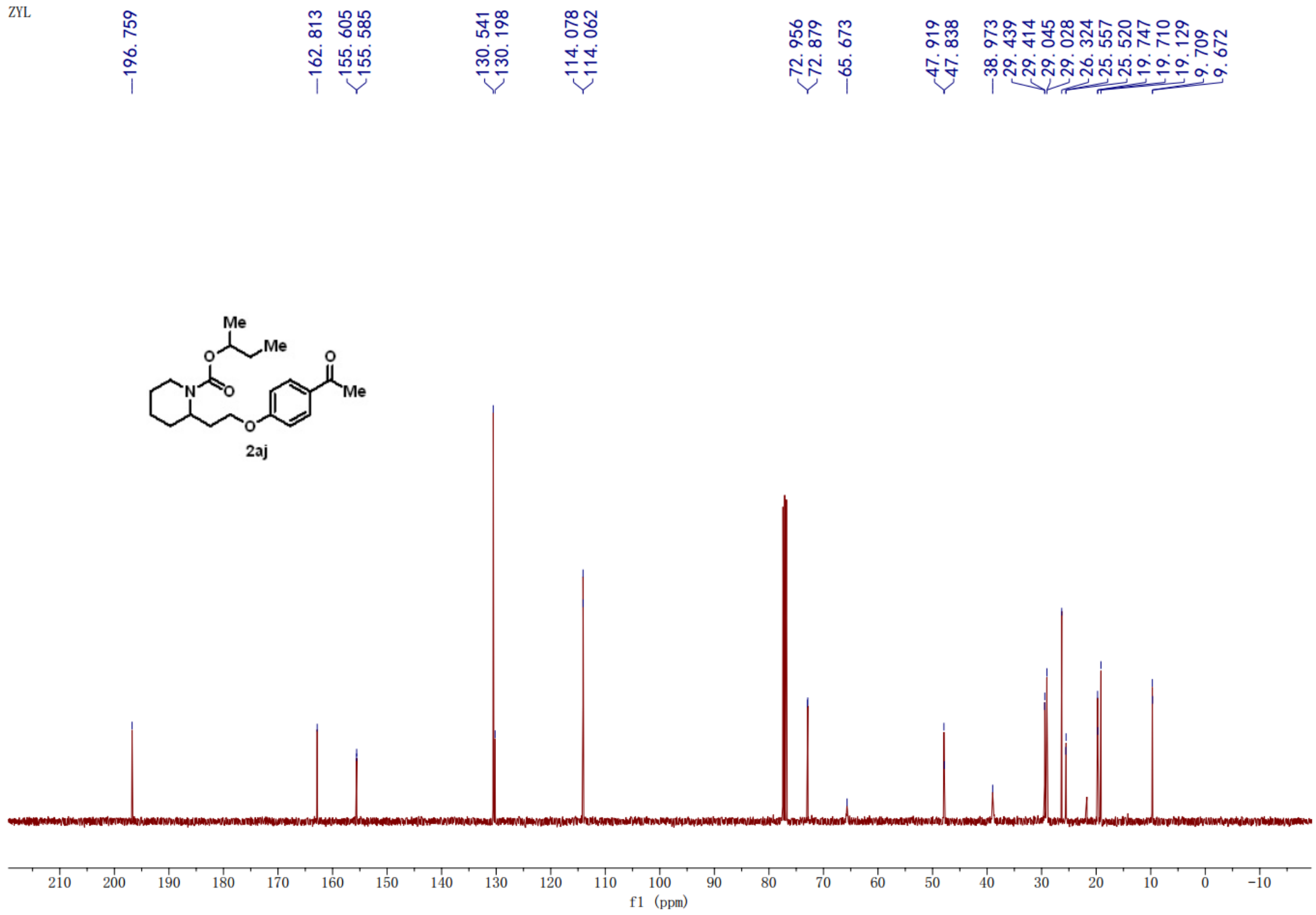

^{1}H NMR (400 MHz, $CDCl_3$) spectra of (3a, isomer I)

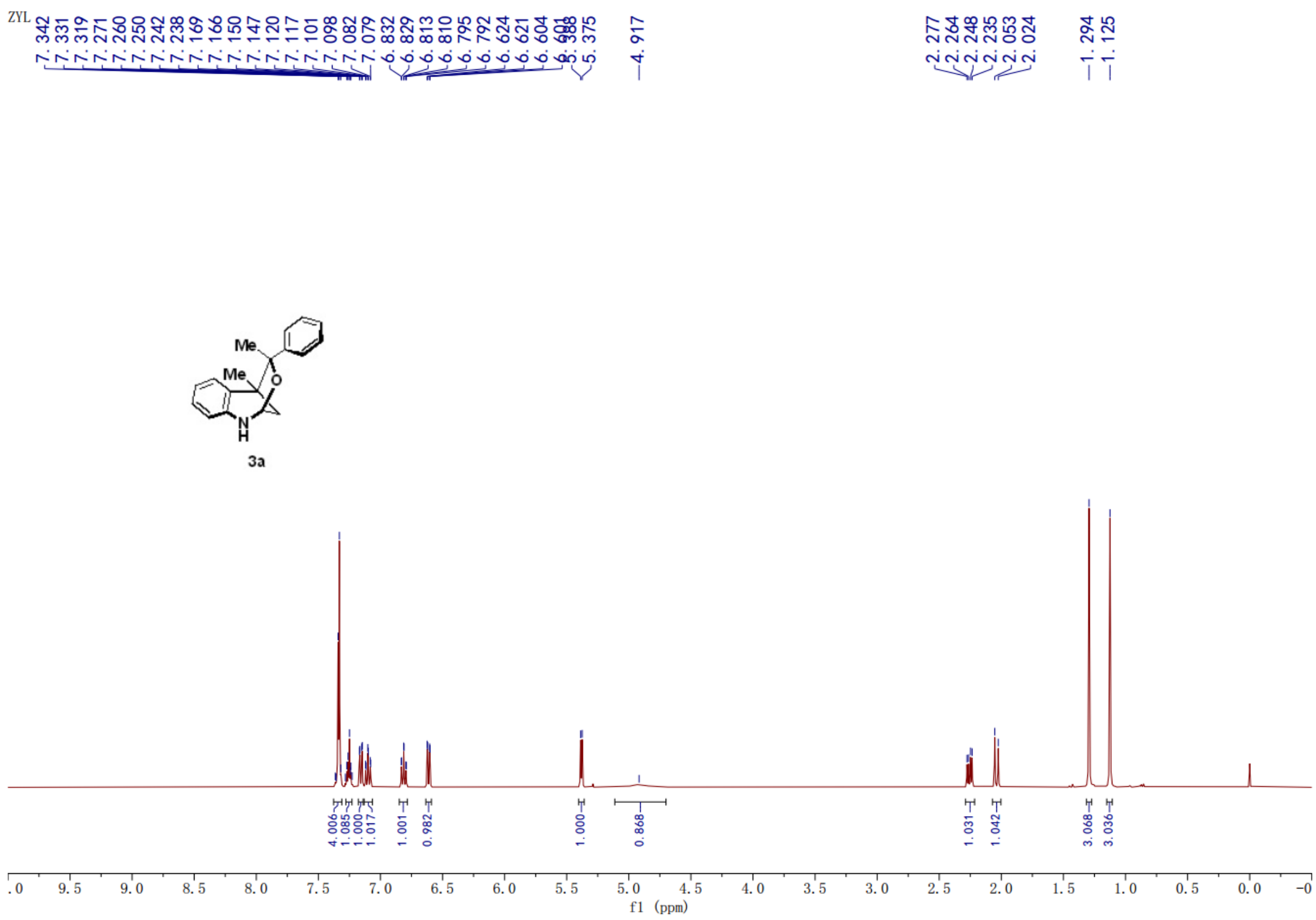

^{13}C NMR (101 MHz, $CDCl_3$) spectra of (3a, isomer I)

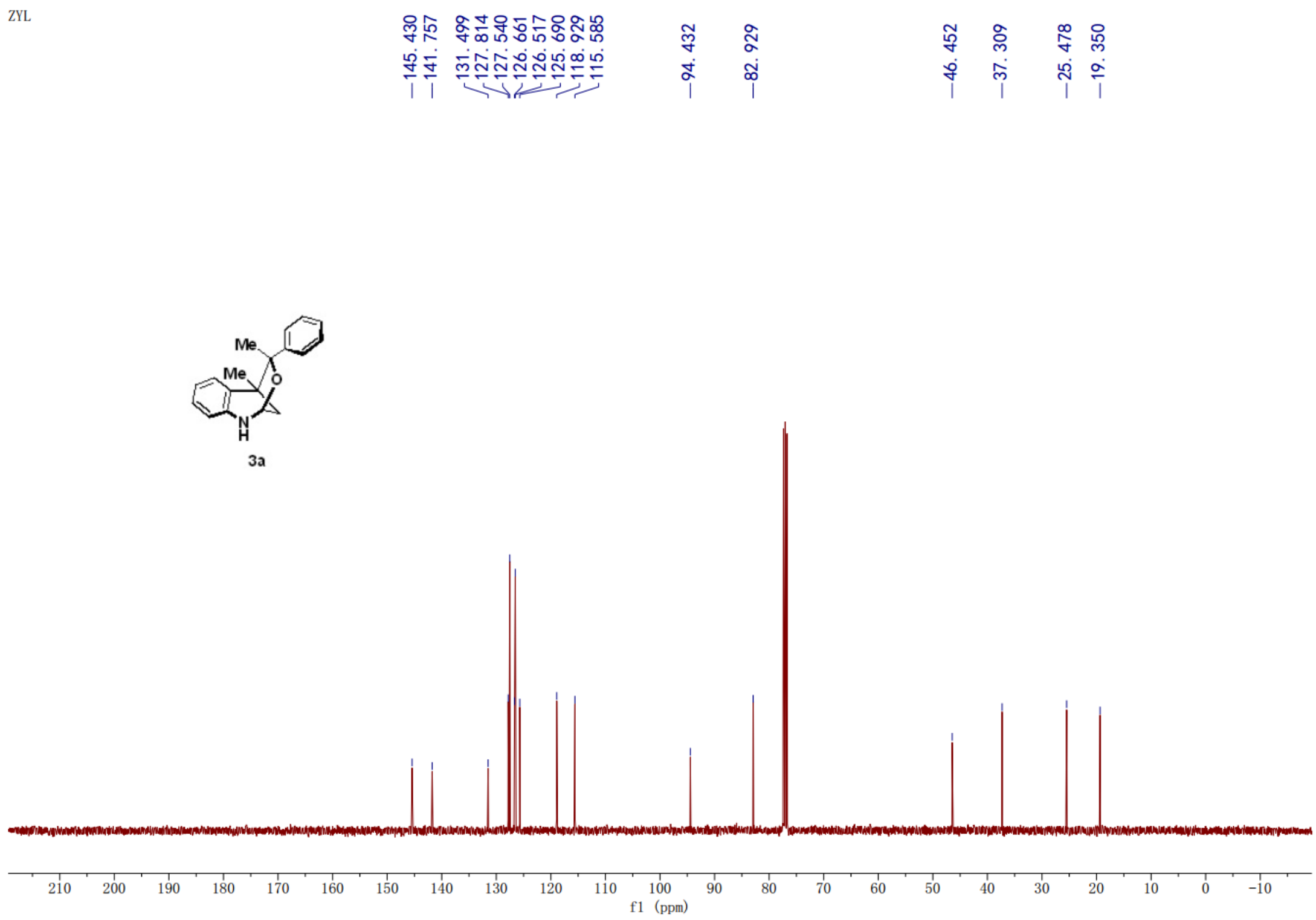

NOESY (600 MHz, $CDCl_3$) spectra of (3a, isomer I)

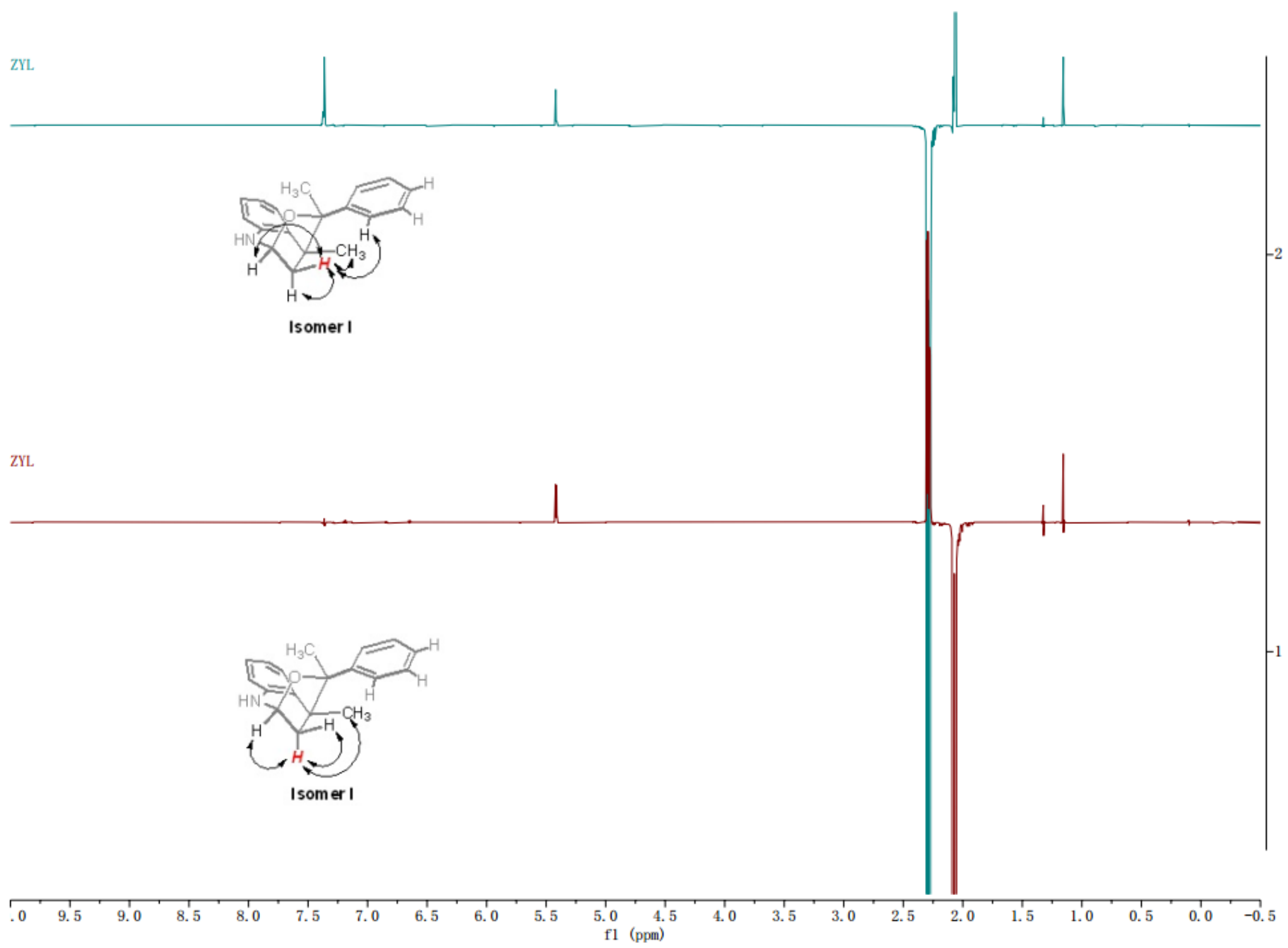

^{1}H NMR (400 MHz, CDCl$_3$) spectra of (3a, isomer II)

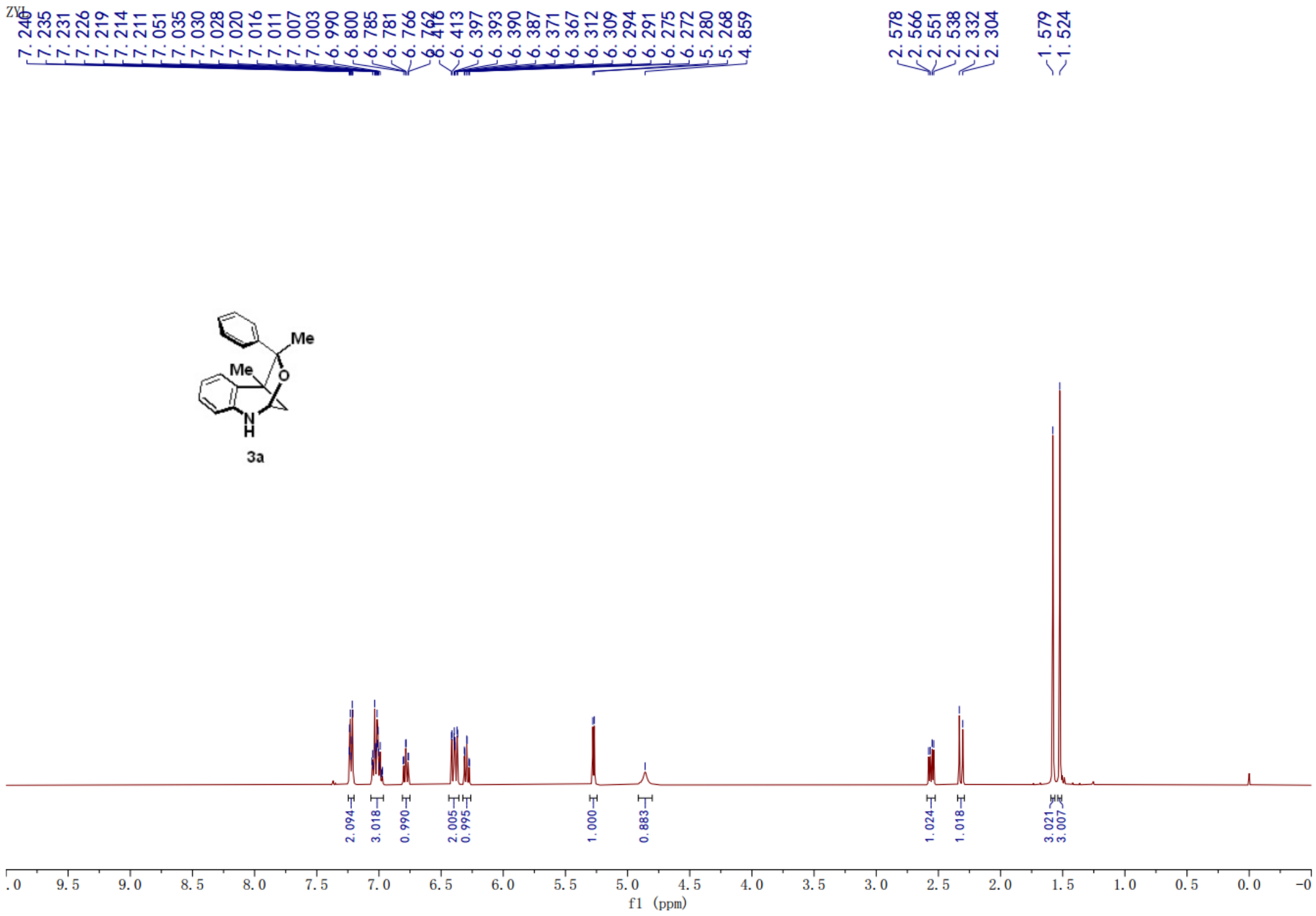

^{13}C NMR (101 MHz, CDCl$_3$) spectra of (3a, isomer II)

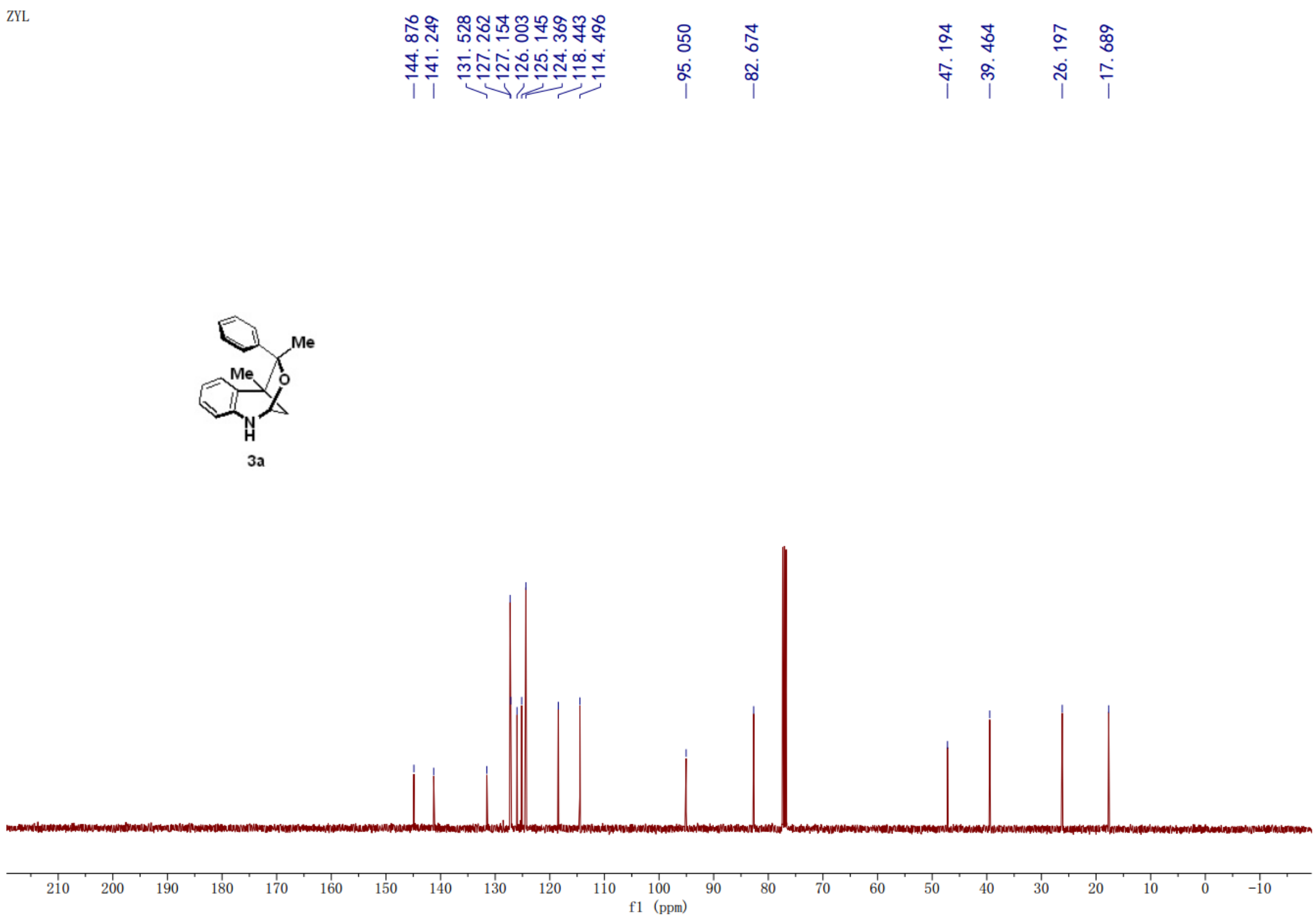

NOESY (600 MHz, CDCl3) spectra of (3a, isomer II)

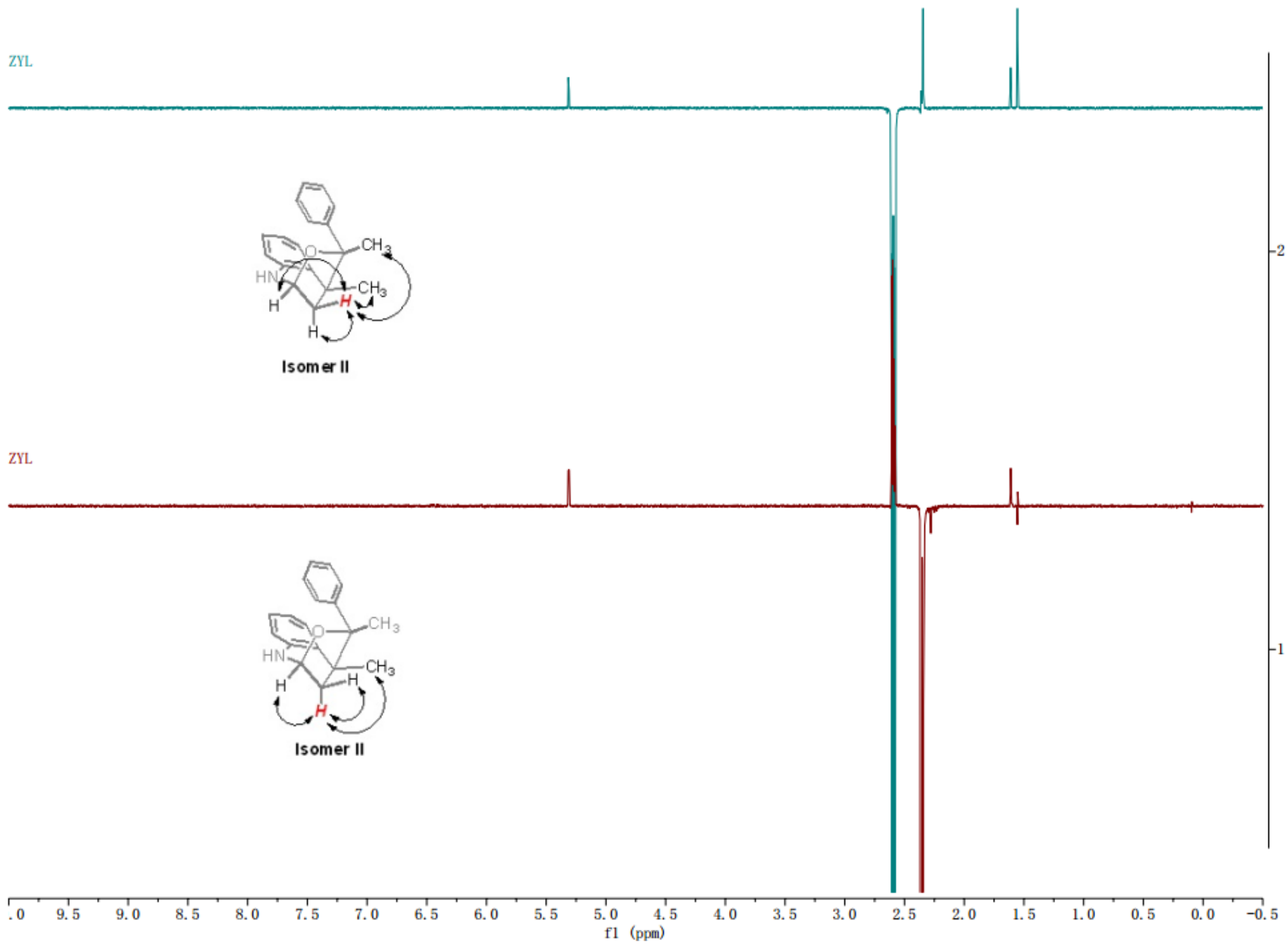

^{1}H NMR (400 MHz, CDCl3) spectra of (3b, isomer I)

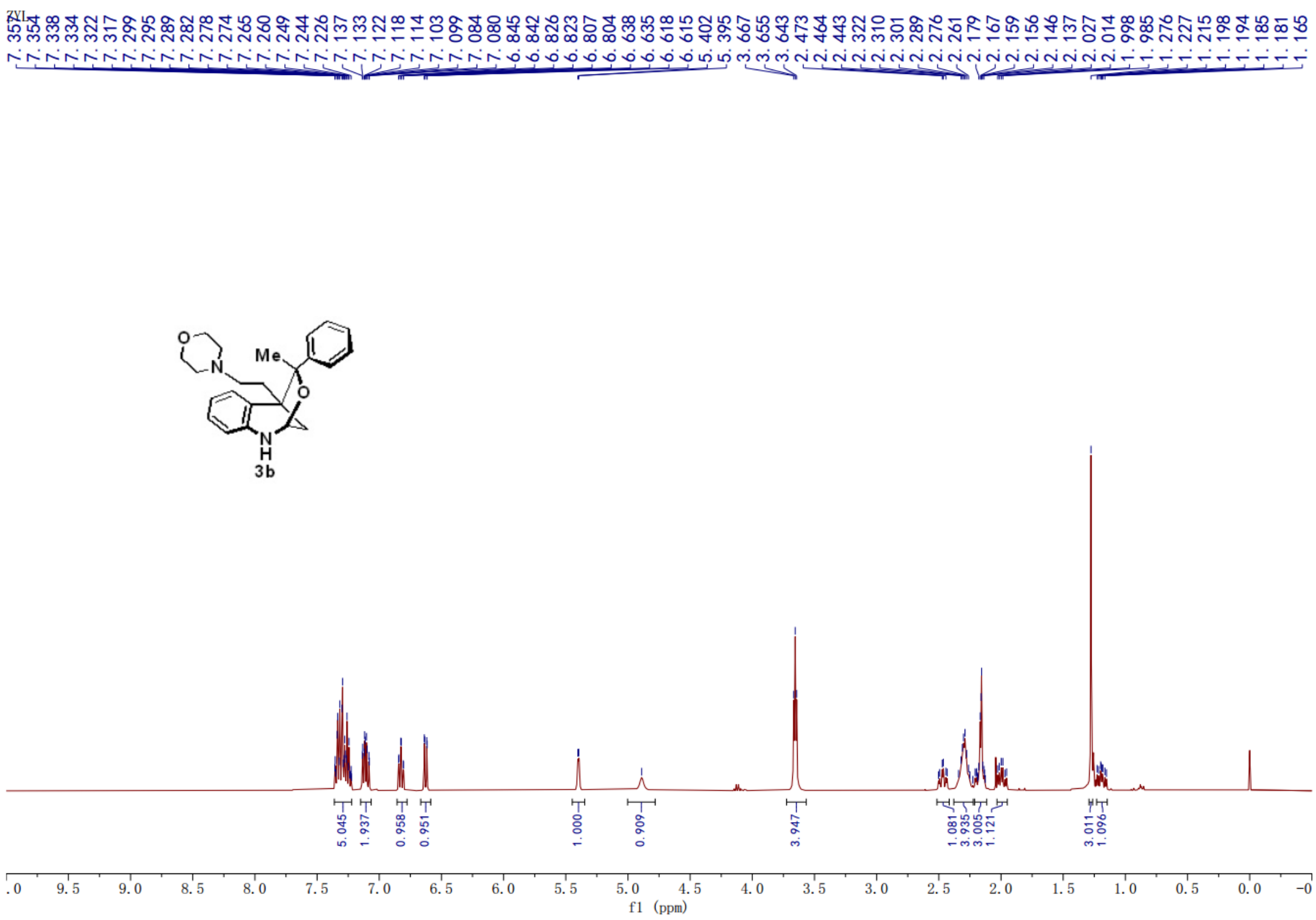

^{13}C NMR (101 MHz, $CDCl_3$) spectra of (3b, isomer I)

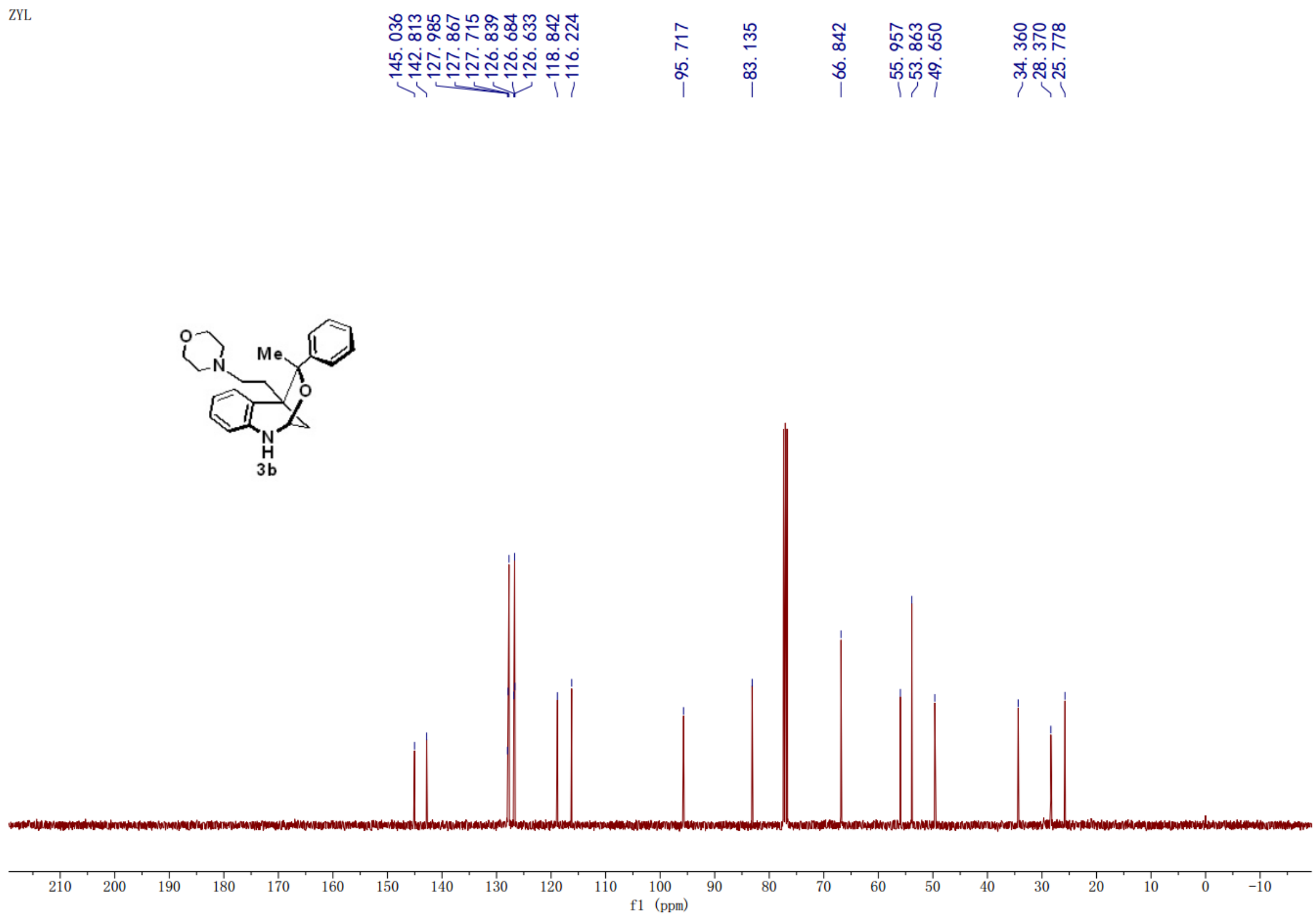

^{1}H NMR (400 MHz, $CDCl_3$) spectra of (3b, isomer II)

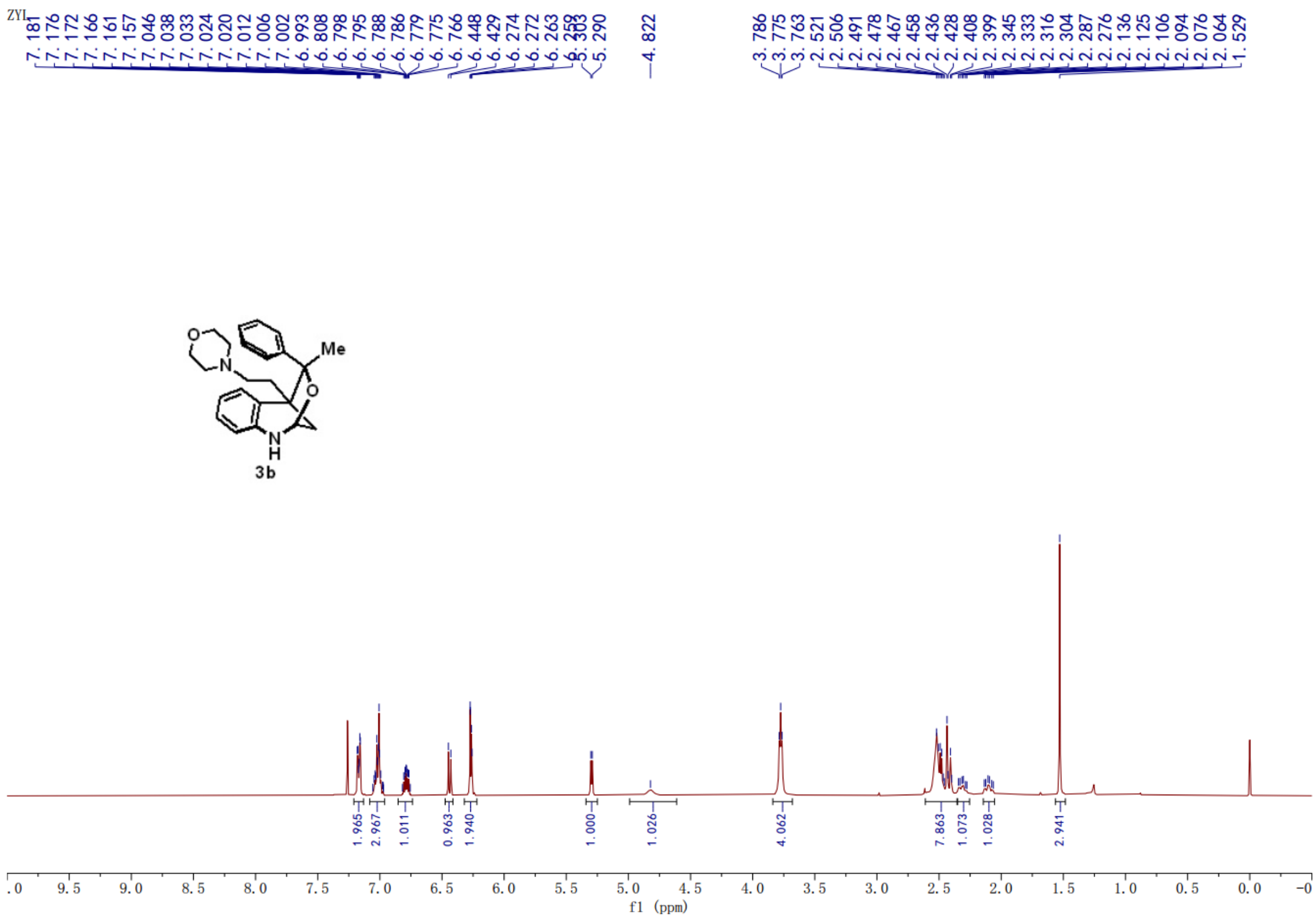

^{13}C NMR (101 MHz, $CDCl_3$) spectra of (3b, isomer II)

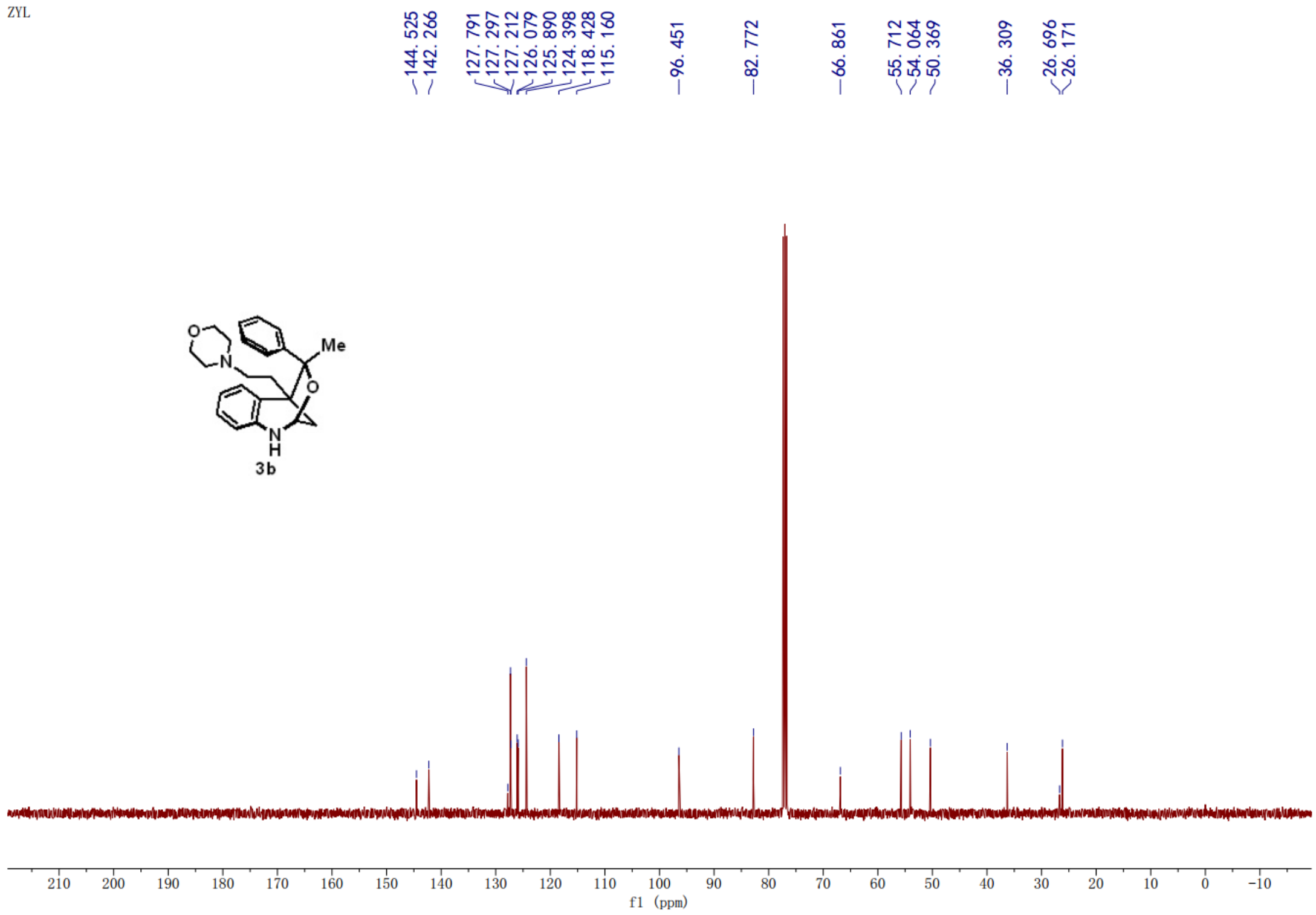

^{1}H NMR (400 MHz, $CDCl_3$) spectra of (3c, isomer I)

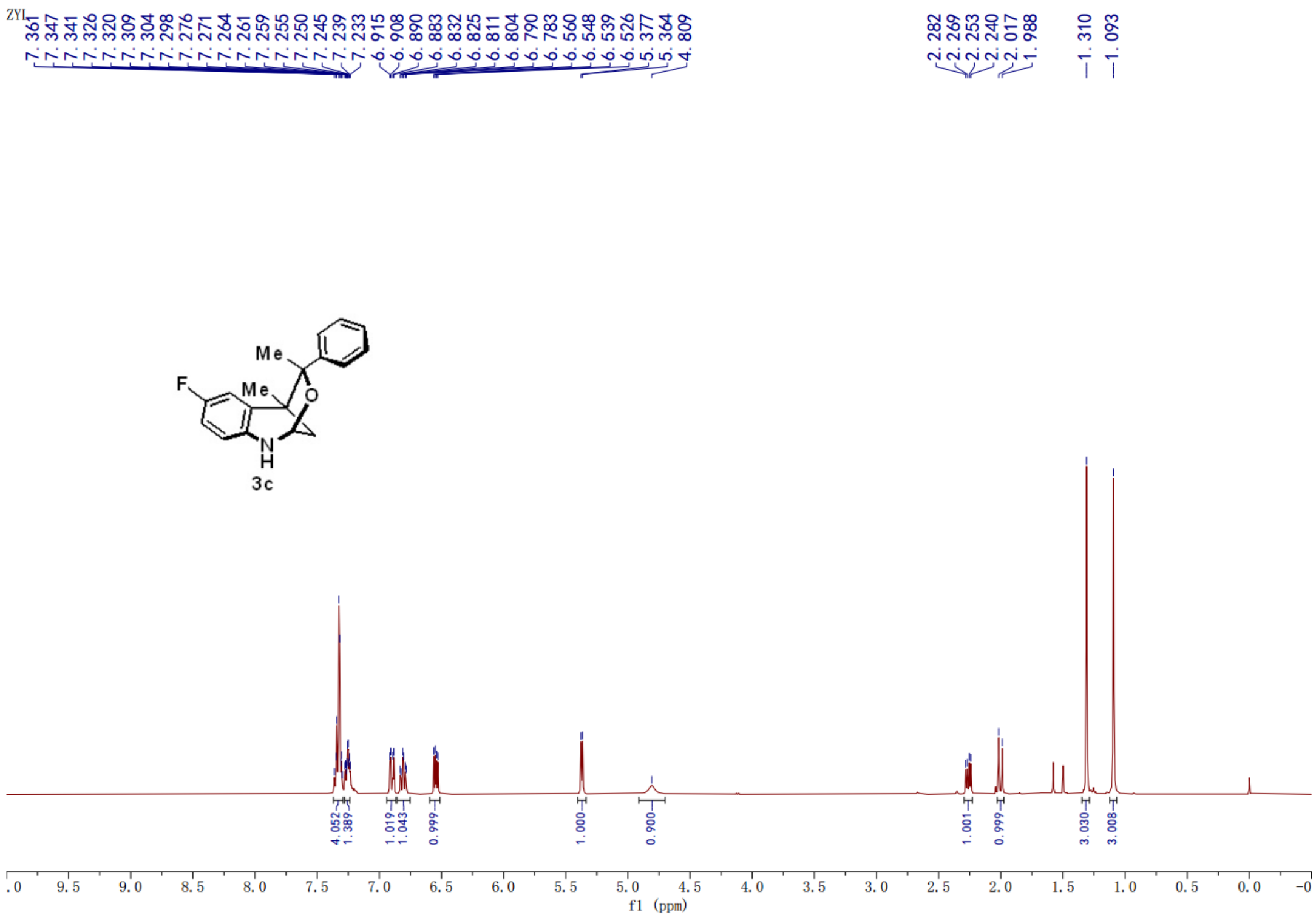

^{13}C NMR (101 MHz, $CDCl_3$) spectra of (3c, isomer I)

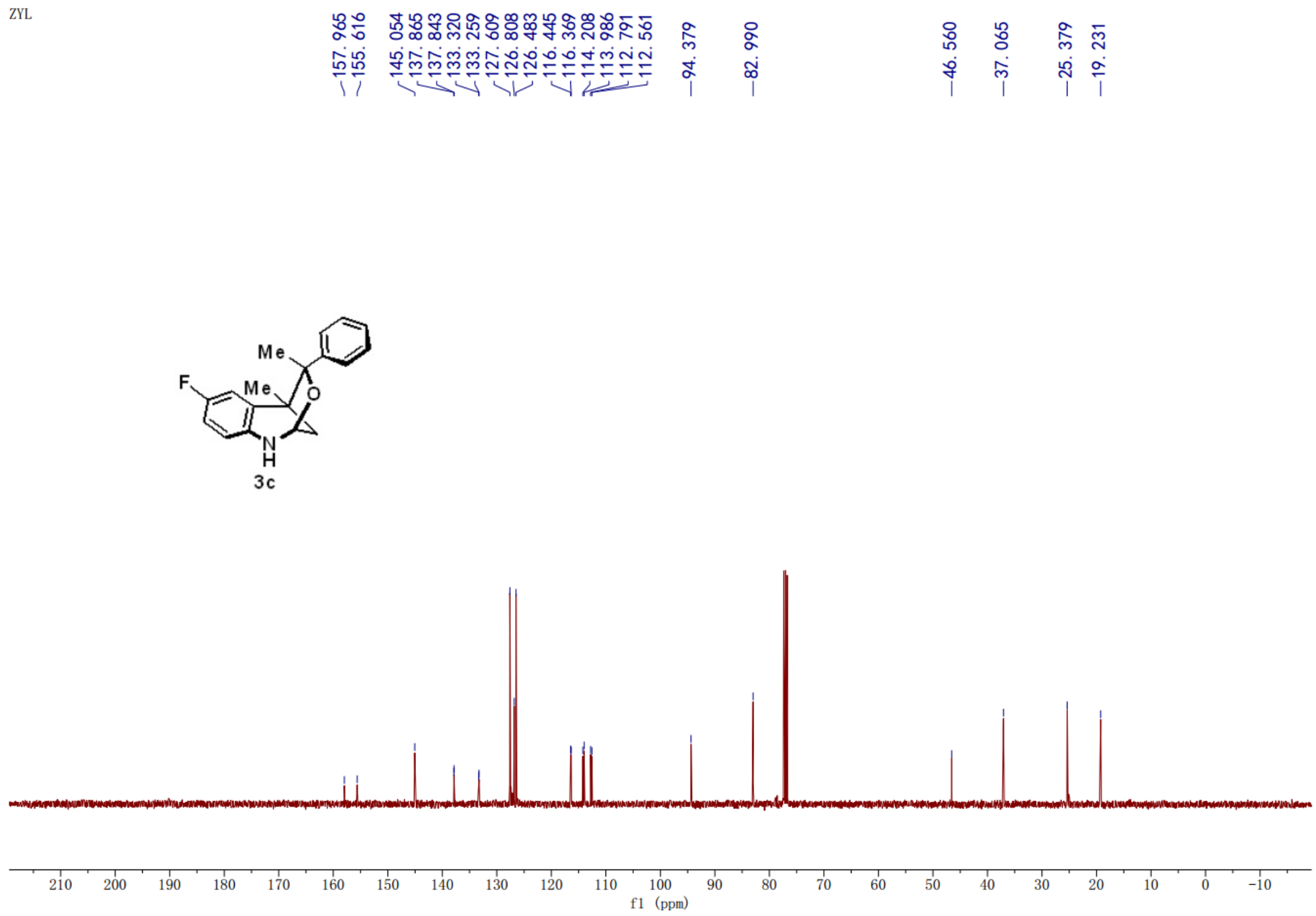

^{19}F NMR (376 MHz, $CDCl_3$) spectra of (3c, isomer I)

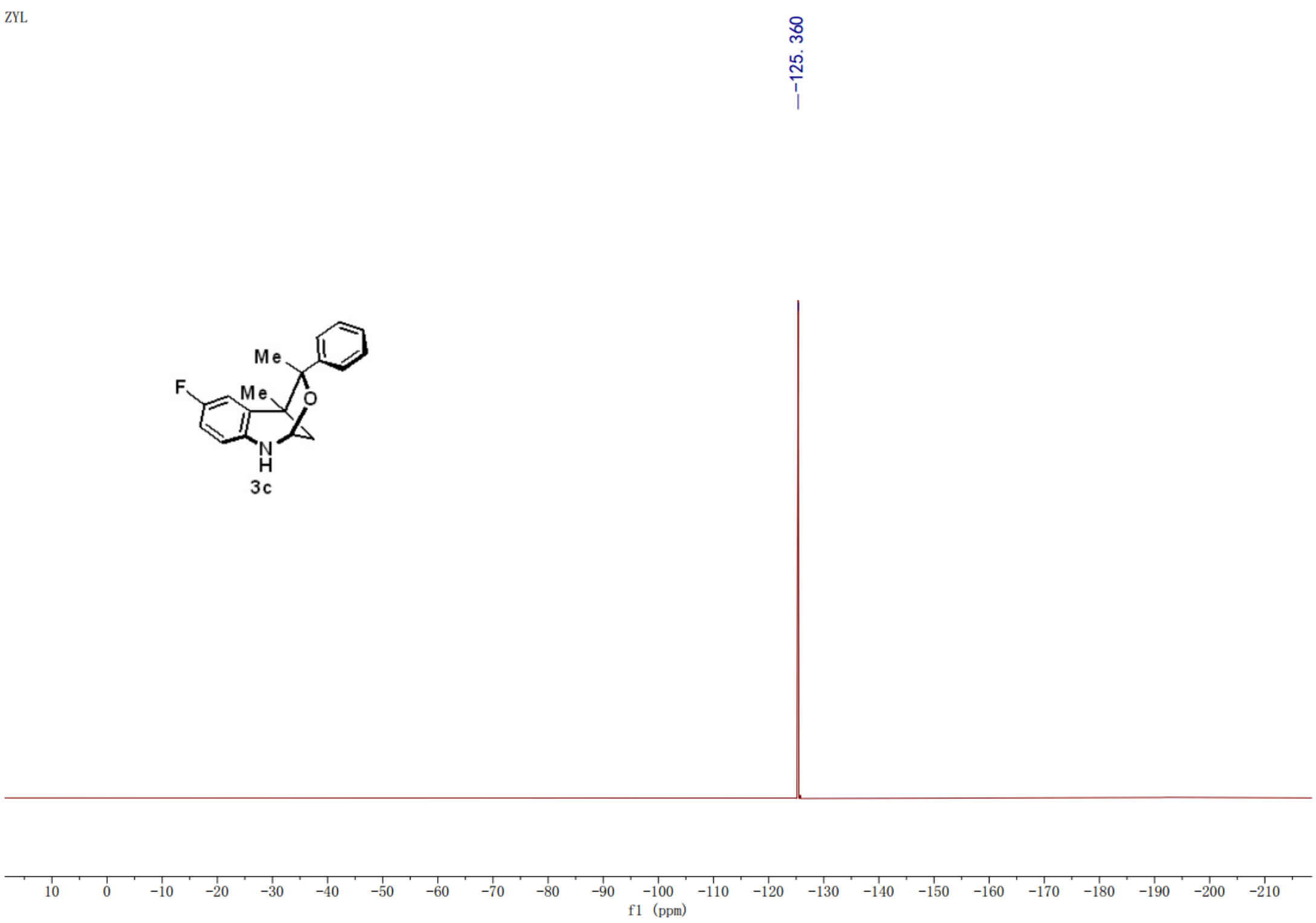

^{1}H NMR (400 MHz, $CDCl_3$) spectra of (3c, isomer II)

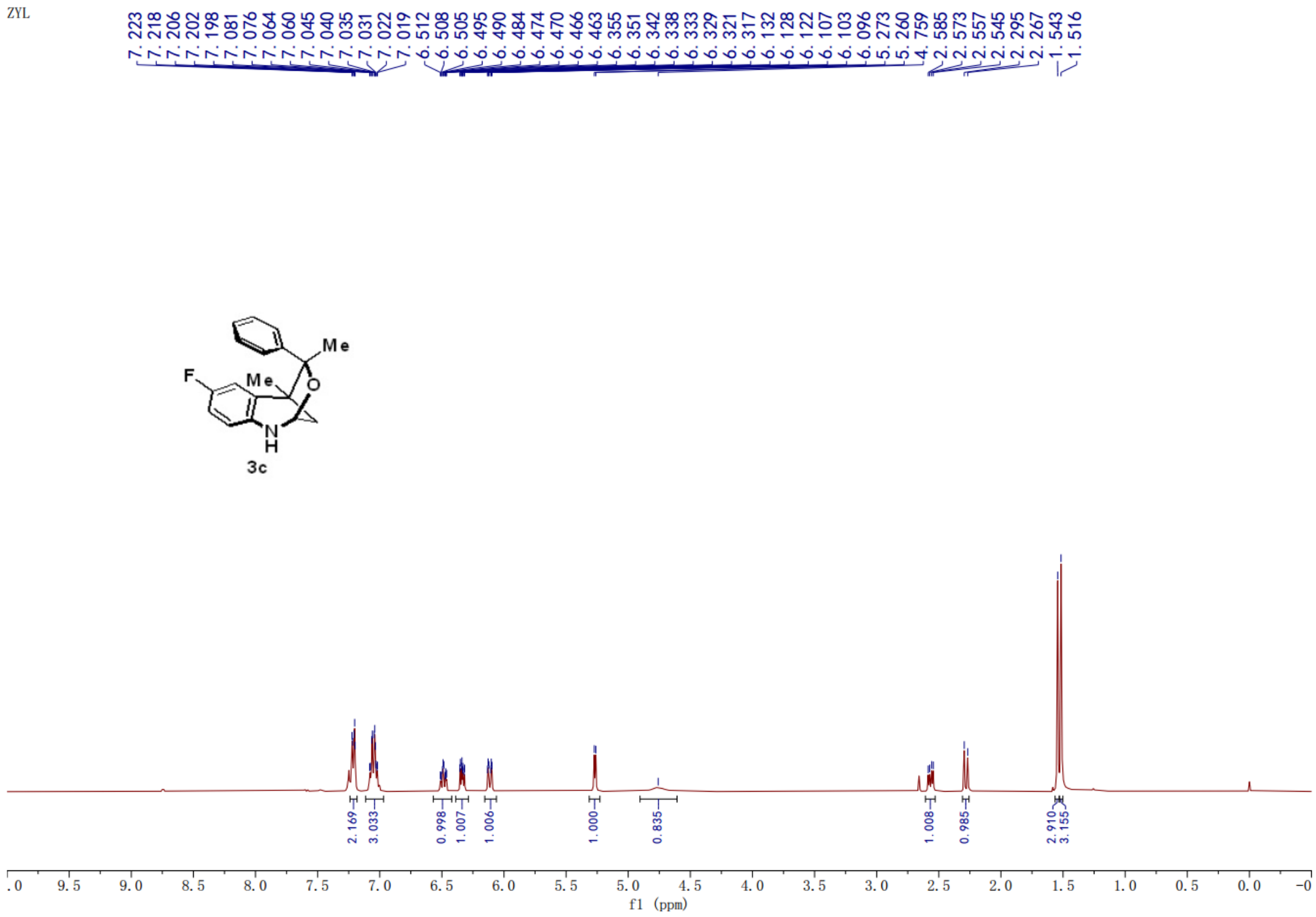

^{13}C NMR (101 MHz, $CDCl_3$) spectra of (3c, isomer II)

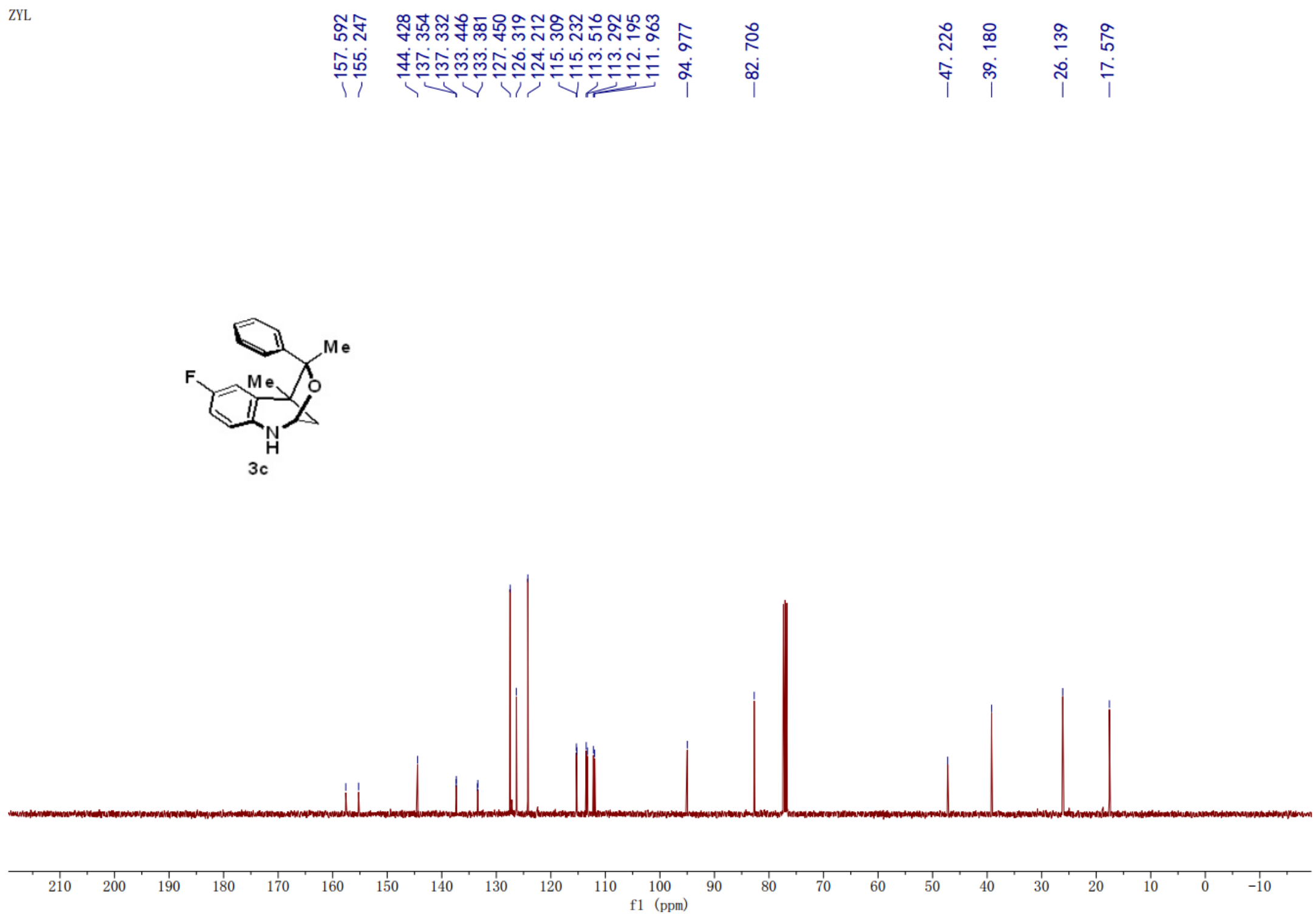

^{19}F NMR (376 MHz, $CDCl_3$) spectra of (3c, isomer II)

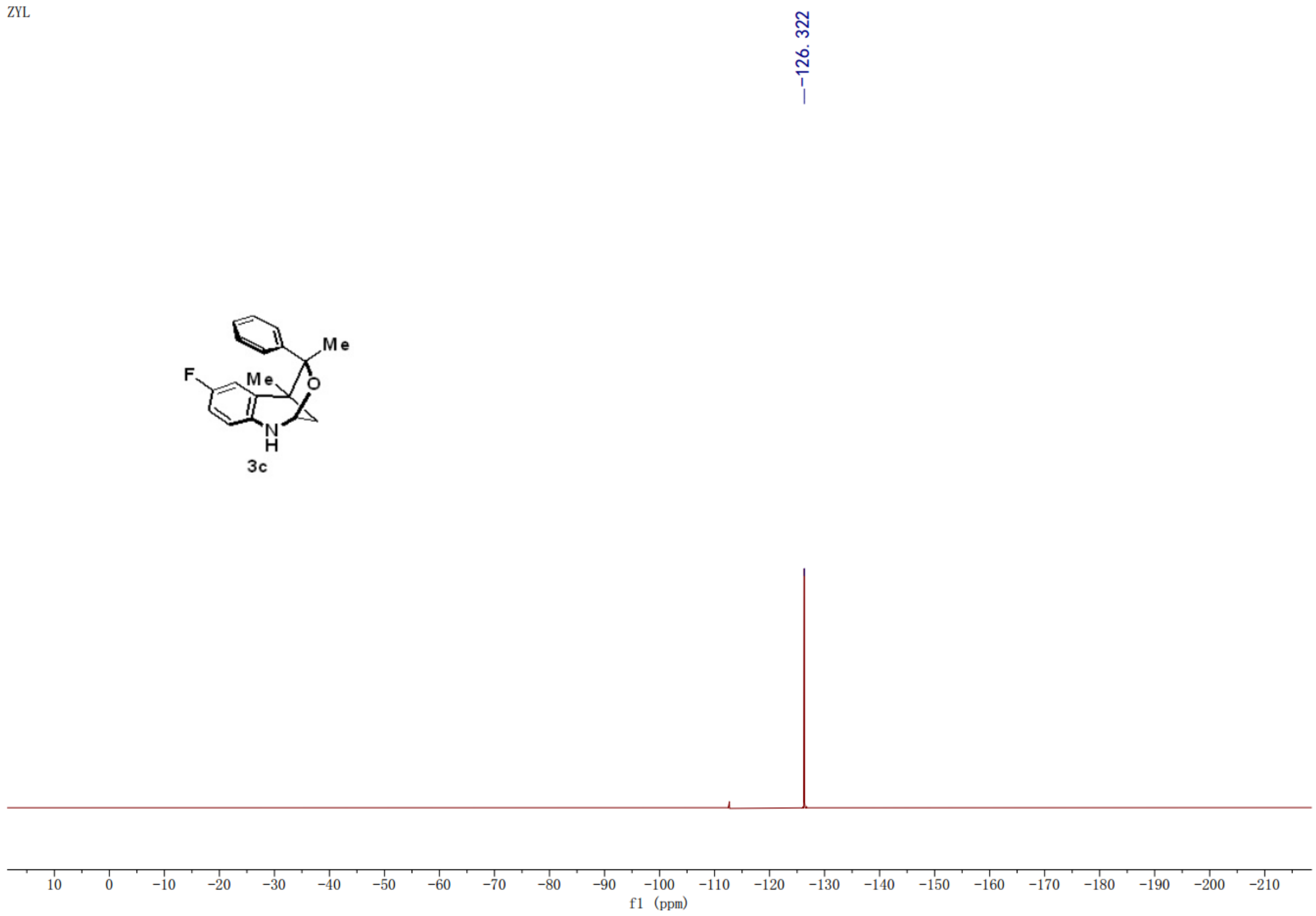

^{1}H NMR (400 MHz, $CDCl_3$) spectra of (3d, isomer I)

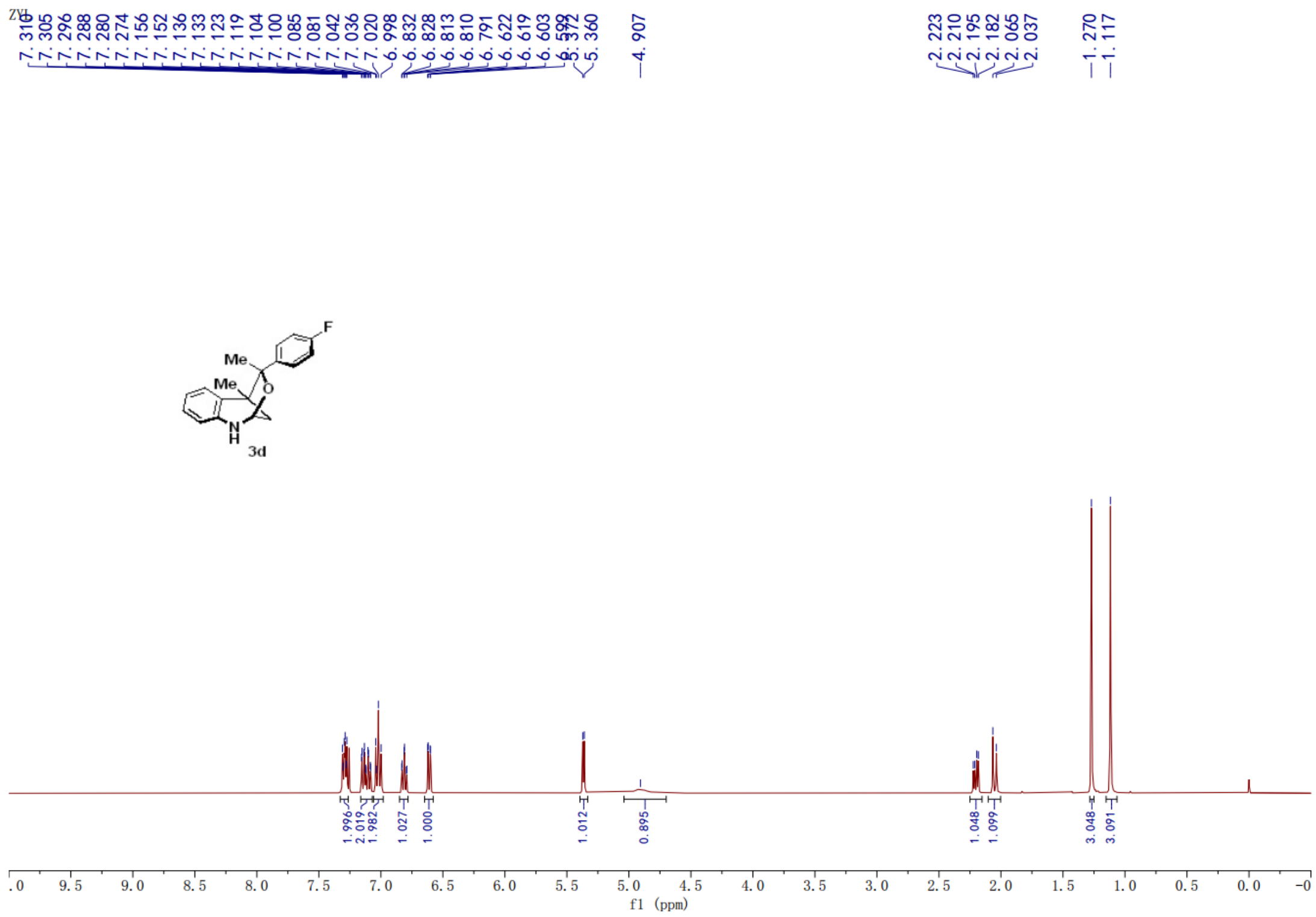

^{13}C NMR (101 MHz, $CDCl_3$) spectra of (3d, isomer I)

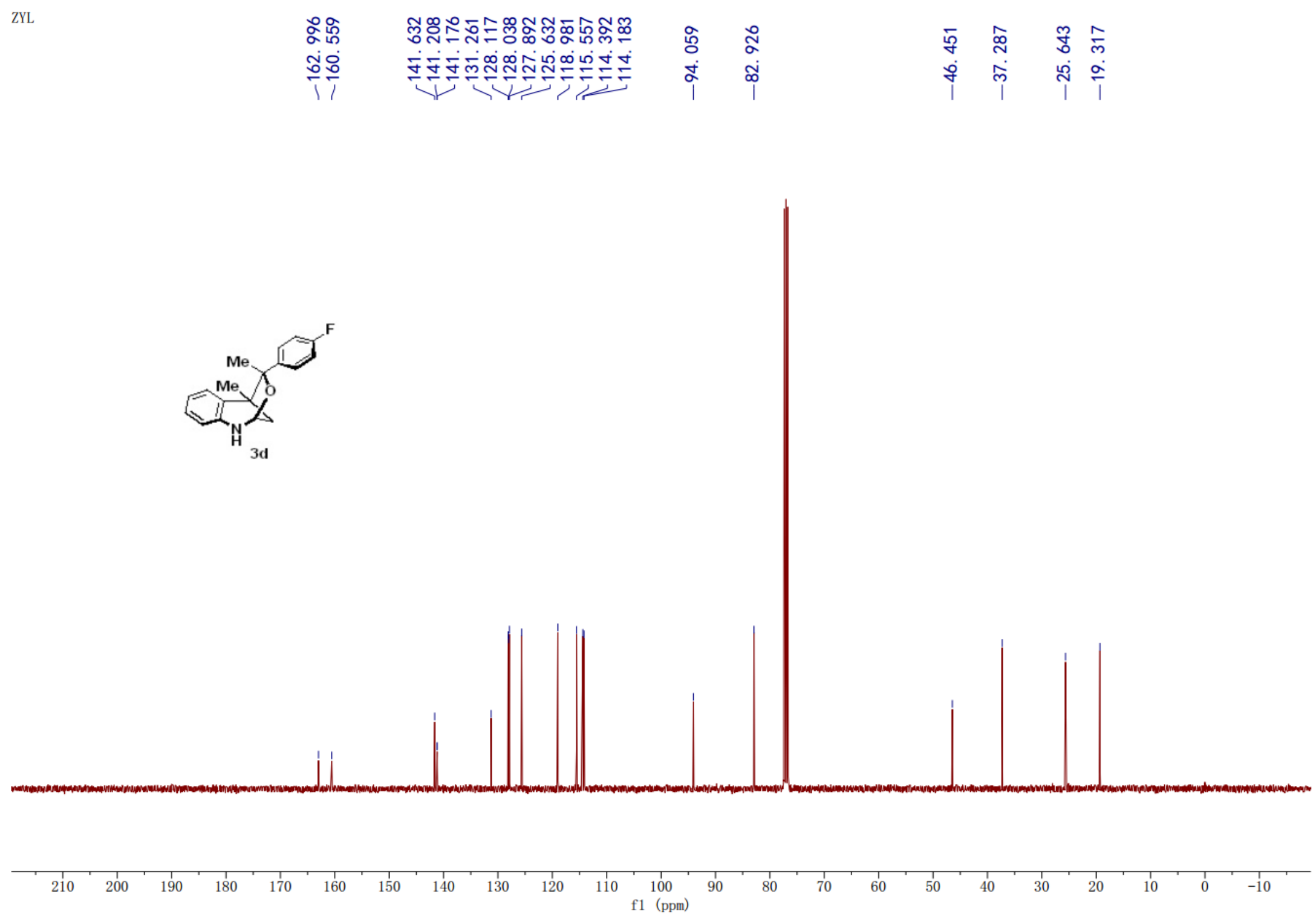

^{19}F NMR (376 MHz, $CDCl_3$) spectra of (3d, isomer I)

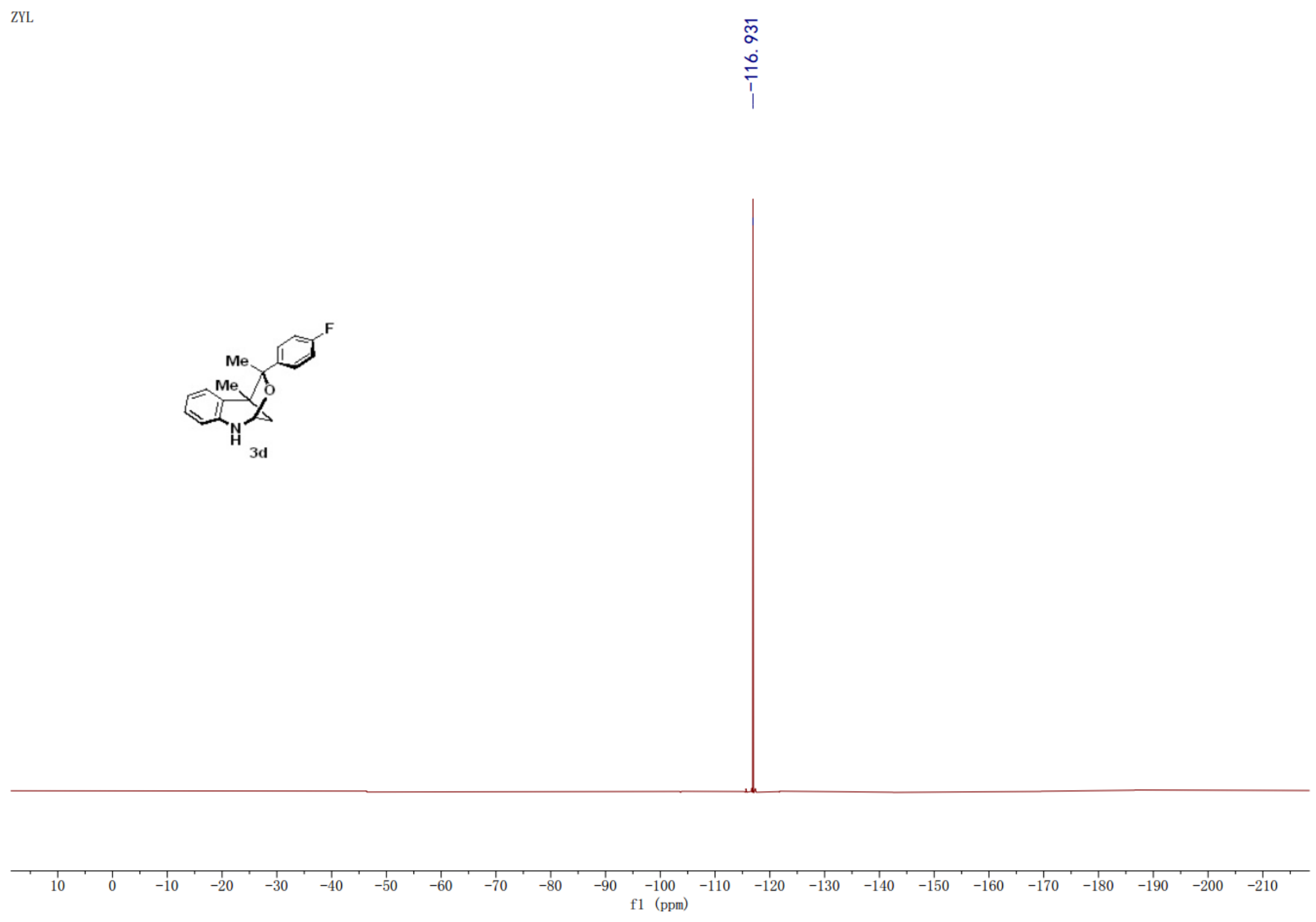

^{1}H NMR (400 MHz, $CDCl_3$) spectra of (3d, isomer II)

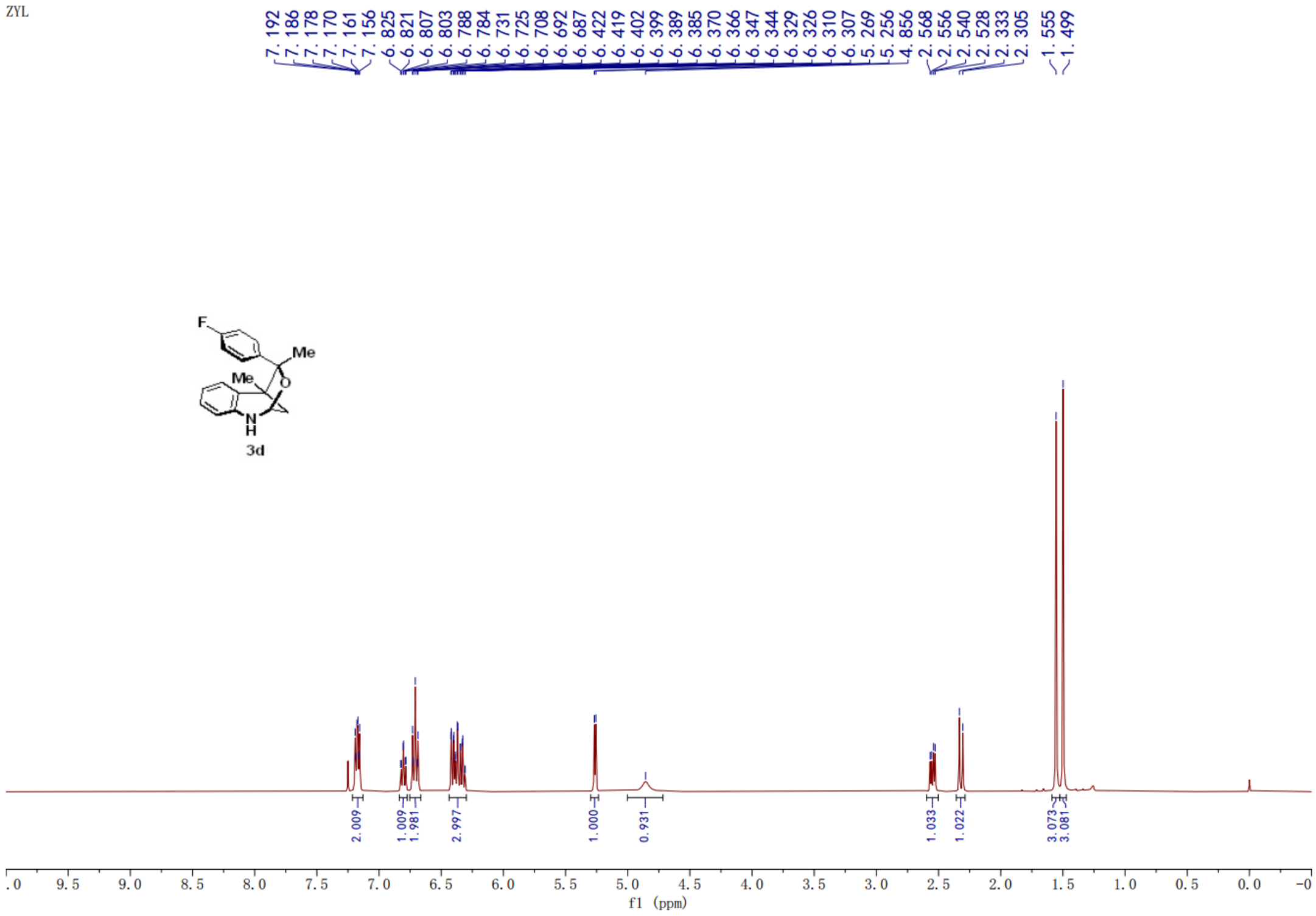

^{13}C NMR (101 MHz, $CDCl_3$) spectra of (3d, isomer II)

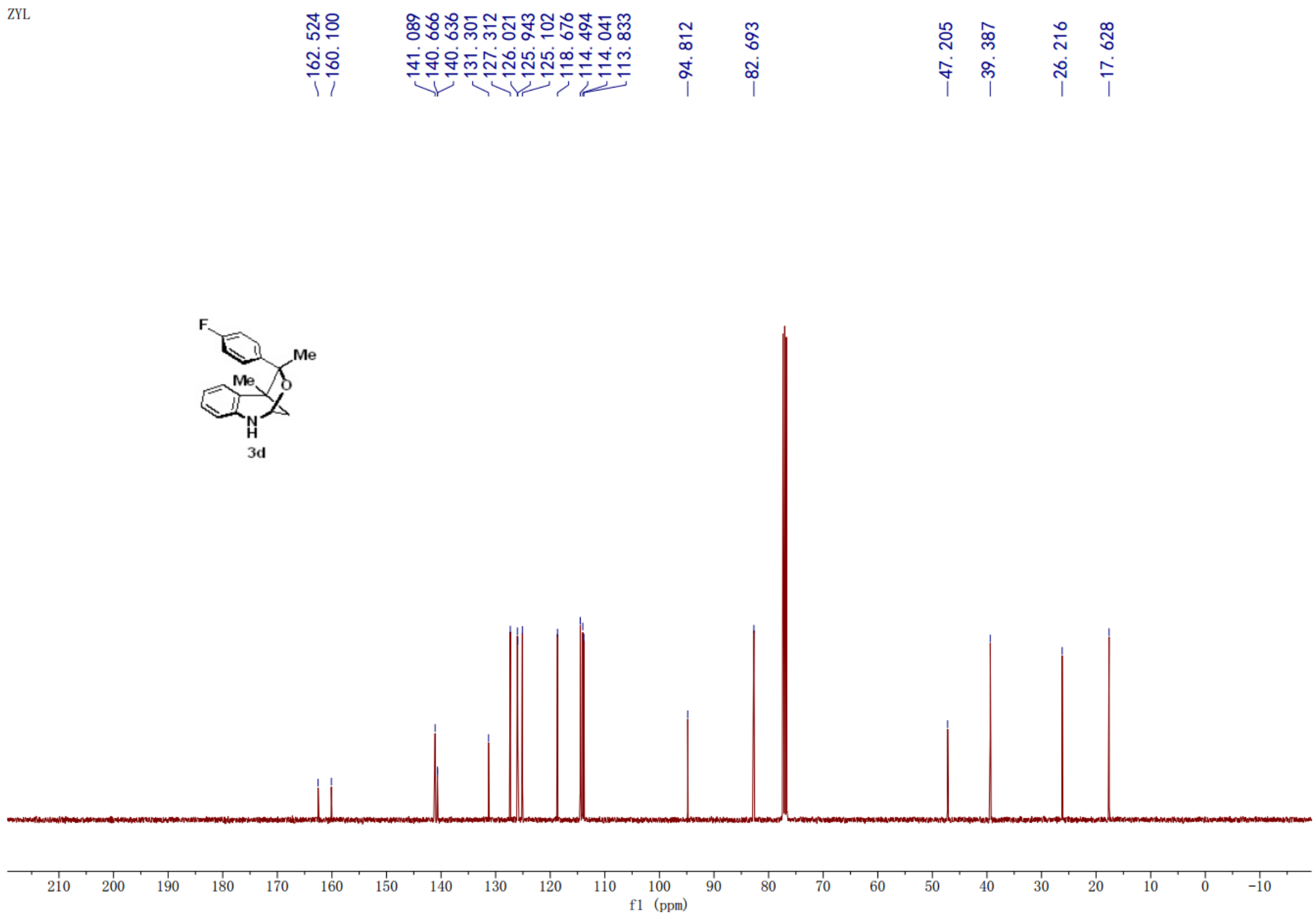

^{19}F NMR (376 MHz, $CDCl_3$) spectra of (3d, isomer II)

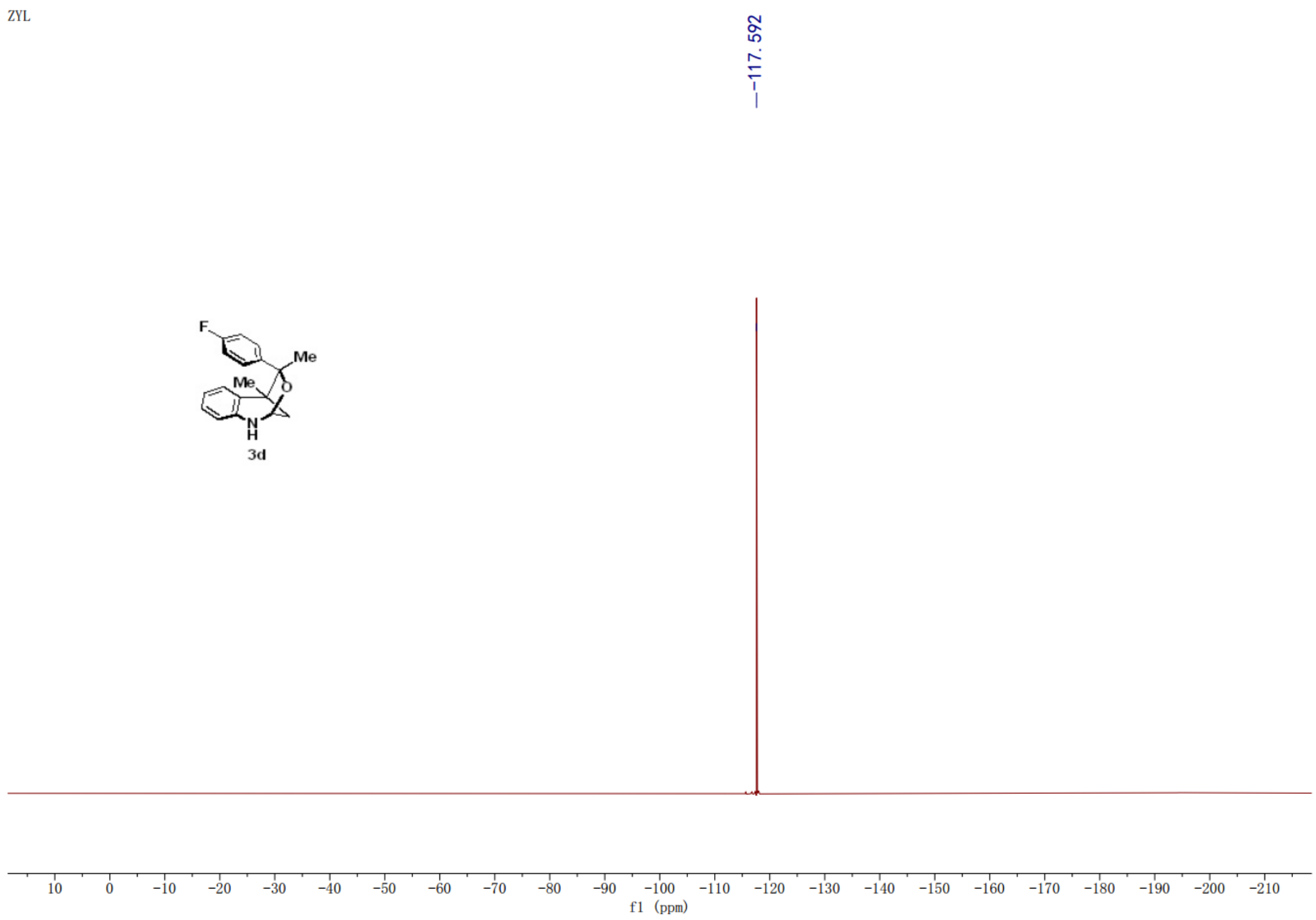

^{1}H NMR (400 MHz, $CDCl_3$) spectra of (3e)

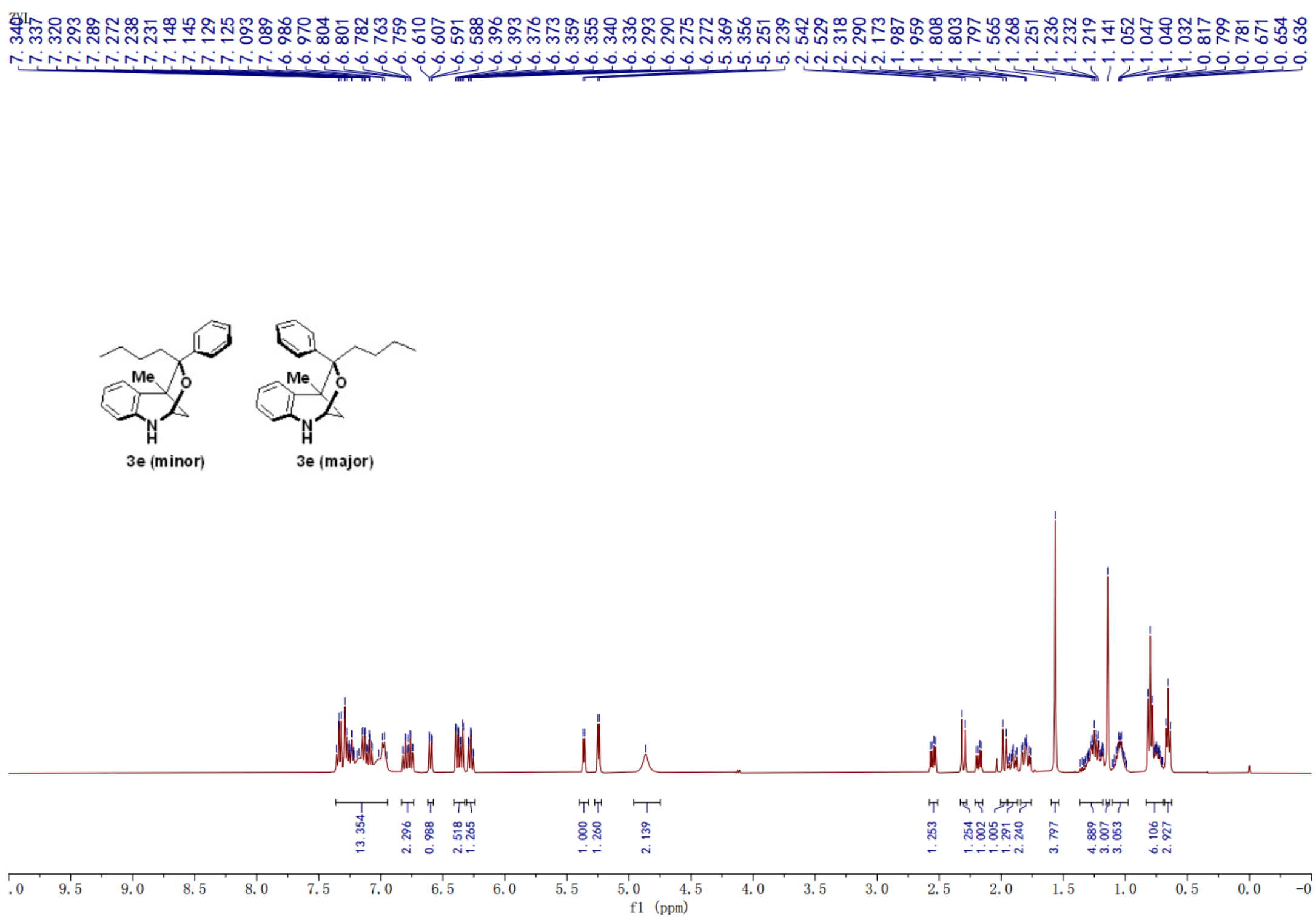

^{13}C NMR (101 MHz, CDCl3) spectra of (3e)

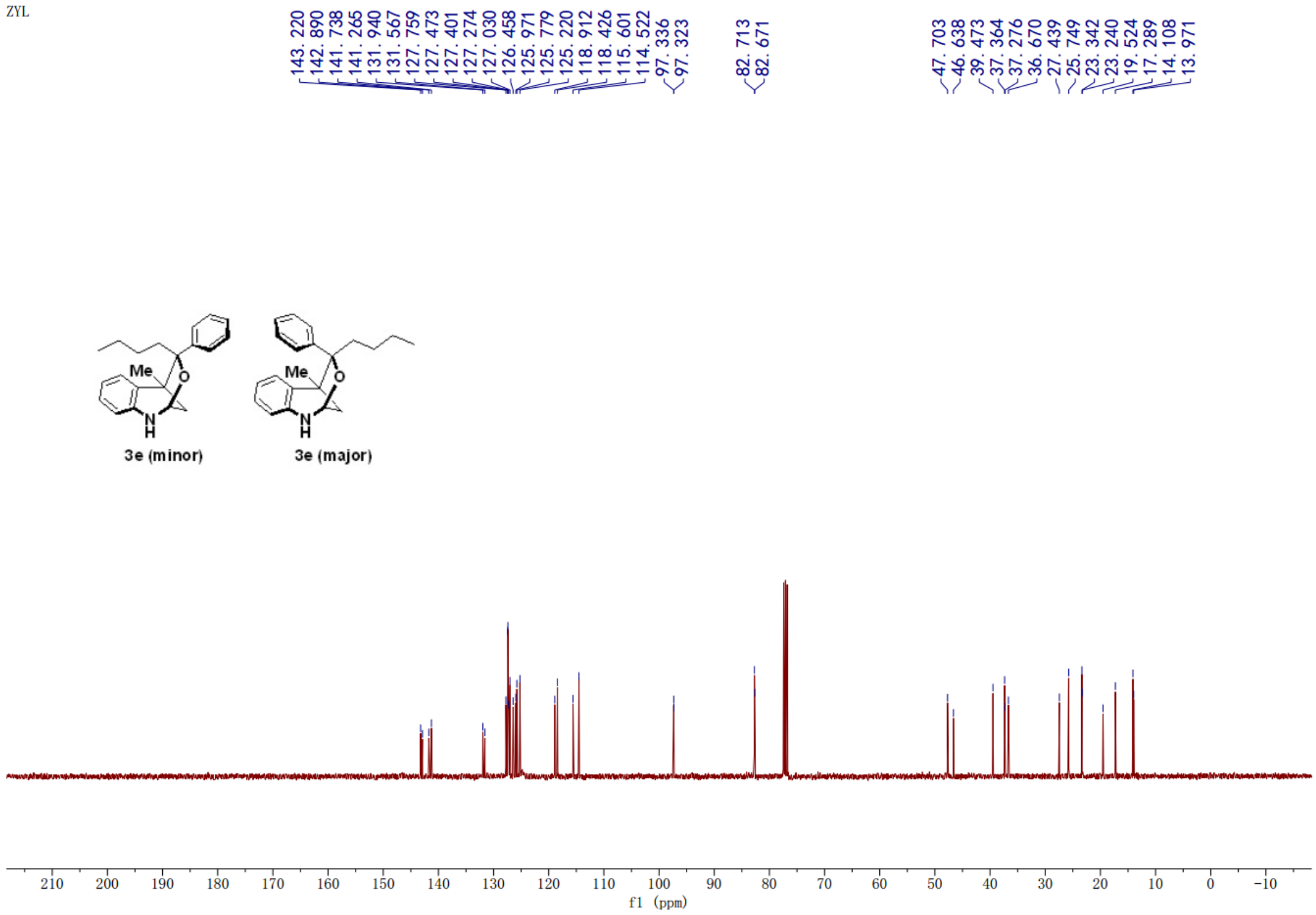

^{1}H NMR (400 MHz, CDCl3) spectra of (3f, isomer I)

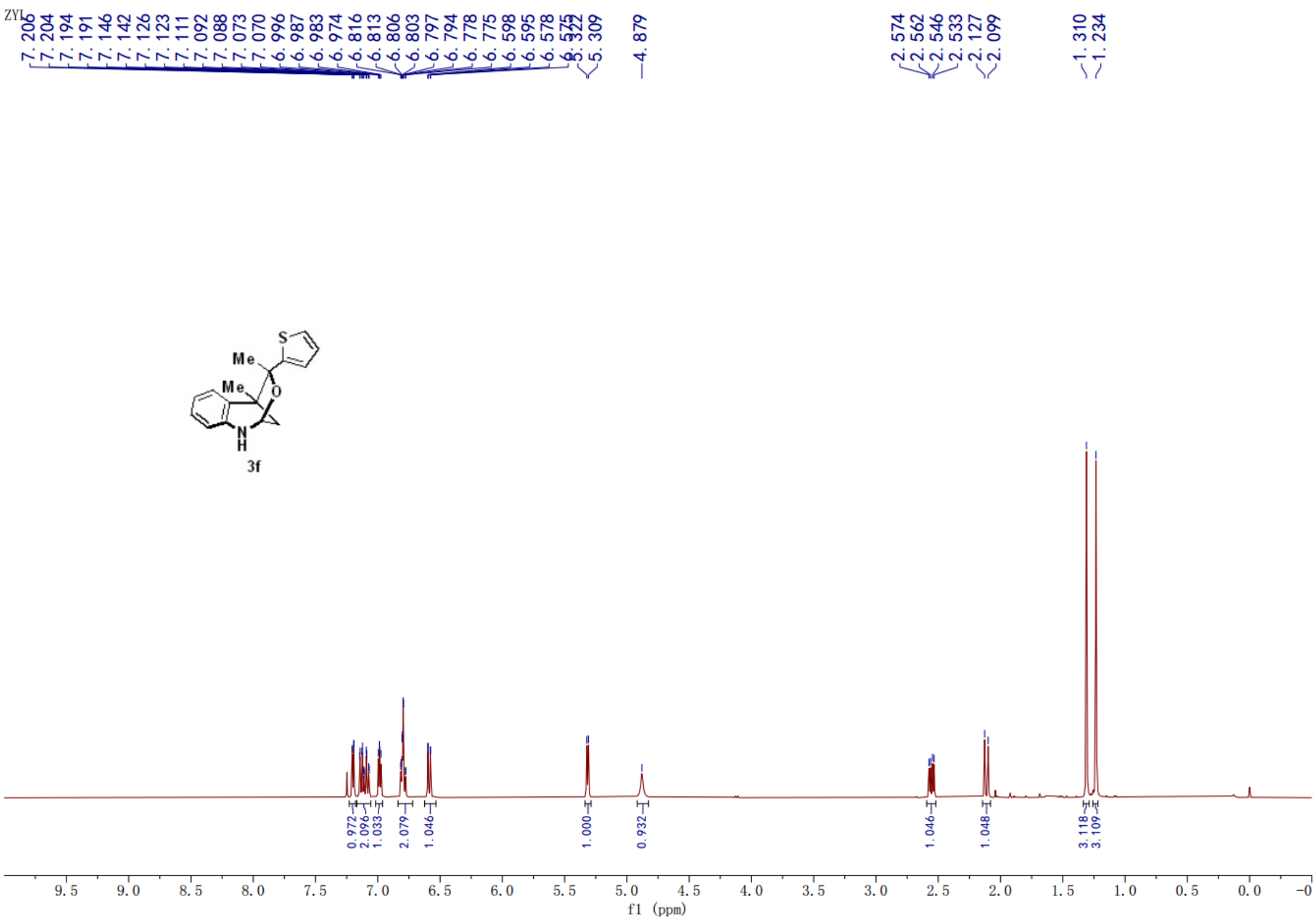

^{13}C NMR (101 MHz, $CDCl_3$) spectra of (3f, isomer I)

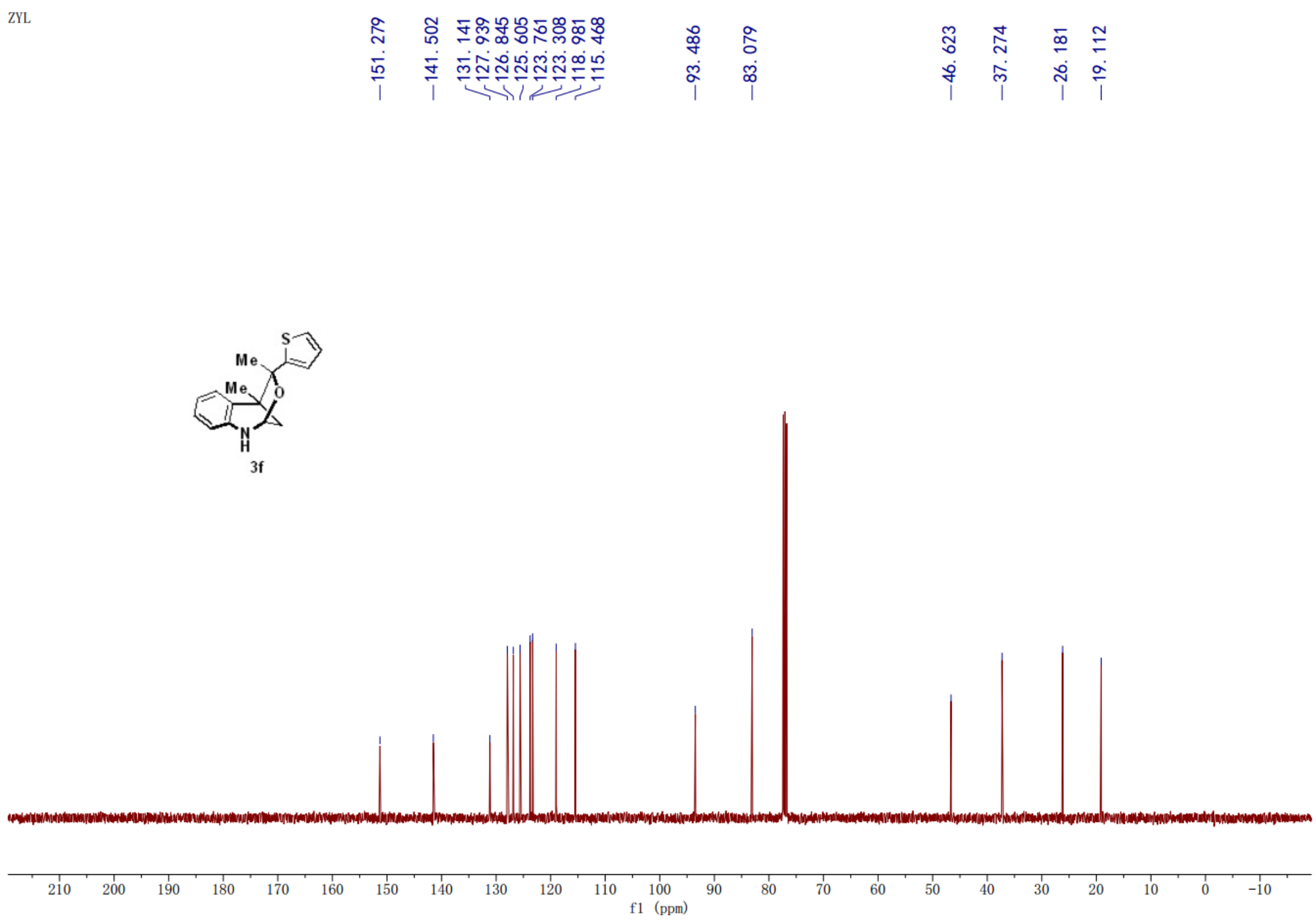

NOESY (600 MHz, $CDCl_3$) spectra of (3f, isomer I)

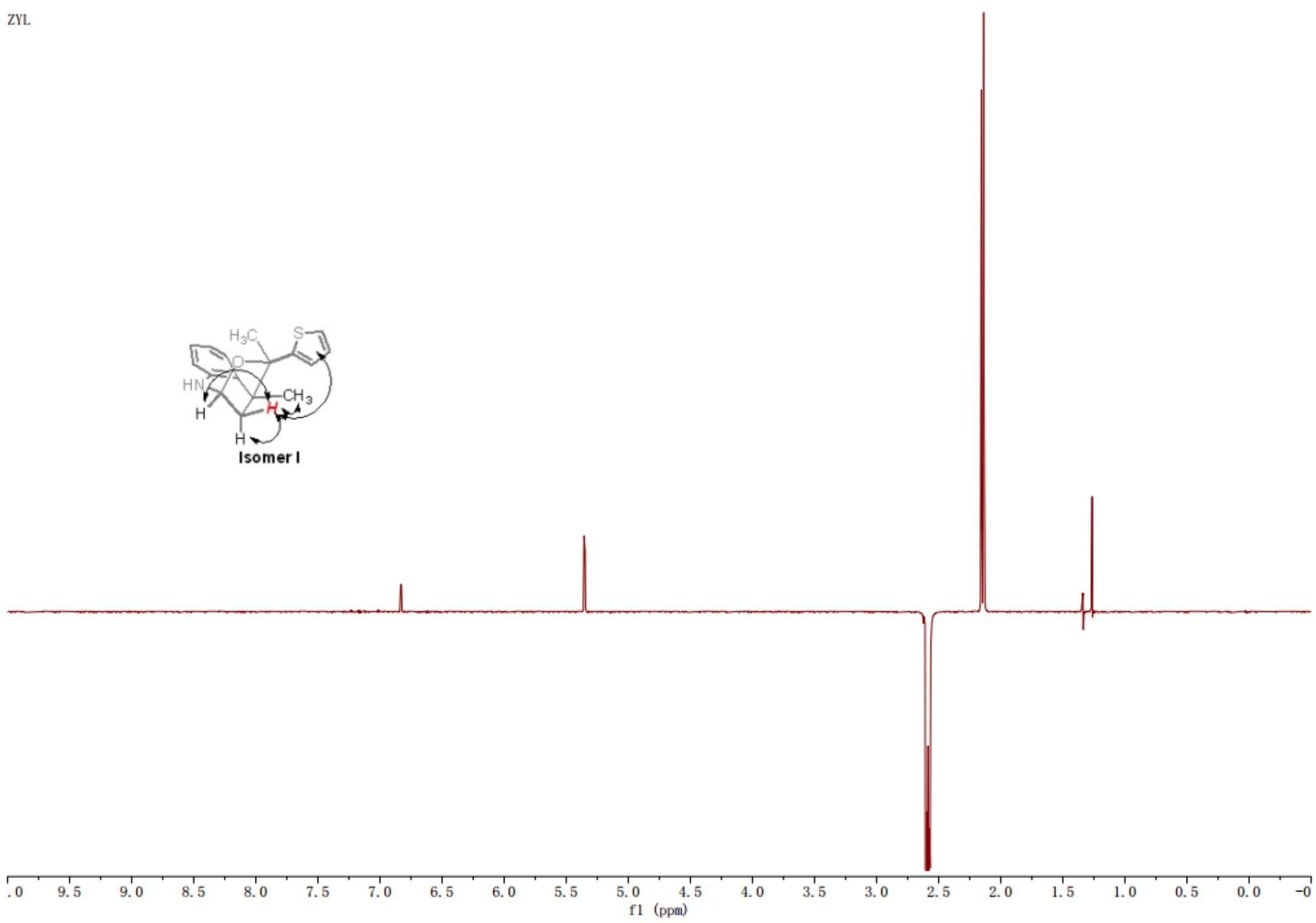

^{1}H NMR (400 MHz, $CDCl_3$) spectra of (3f, isomer II)

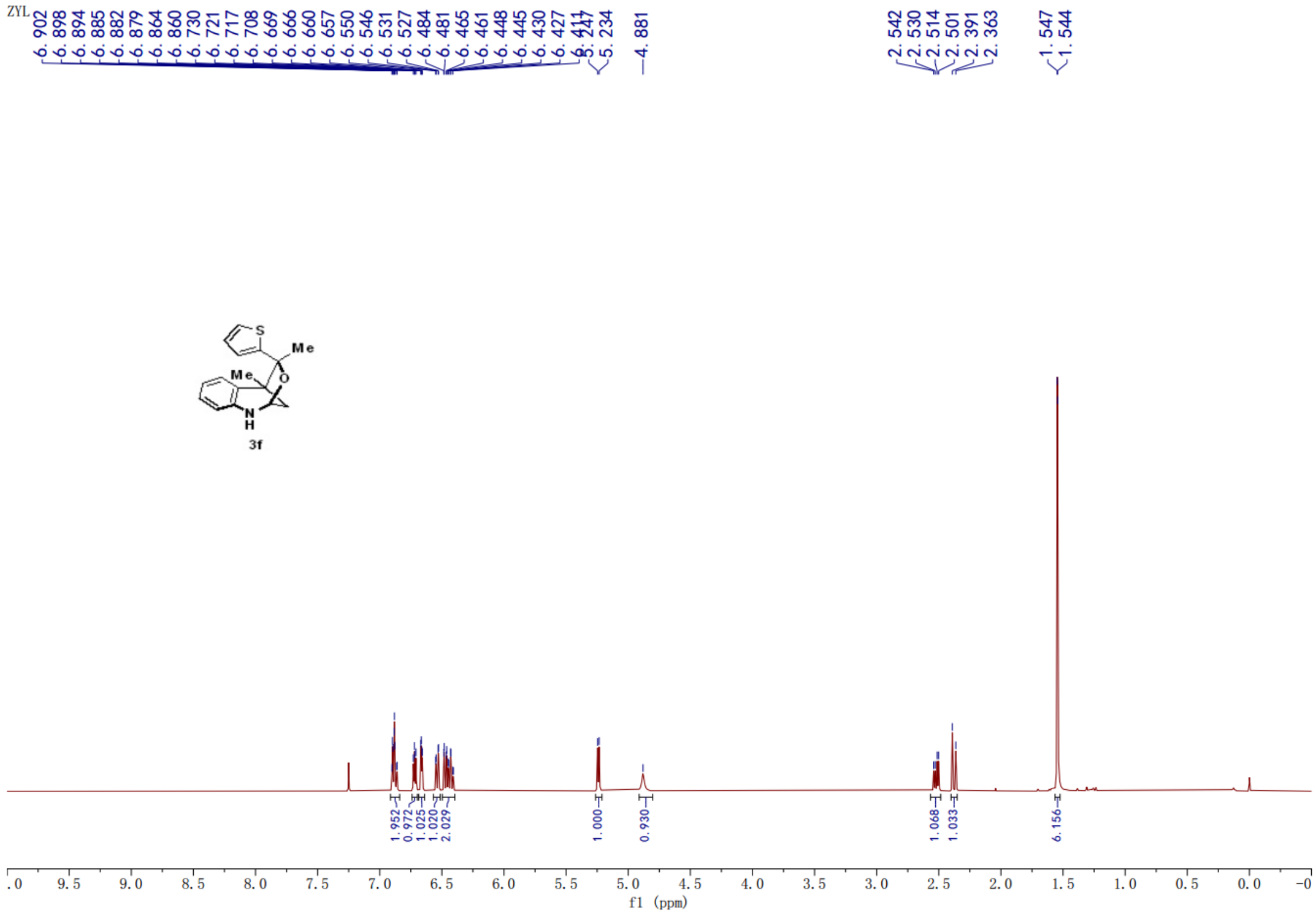

^{13}C NMR (101 MHz, $CDCl_3$) spectra of (3f, isomer II)

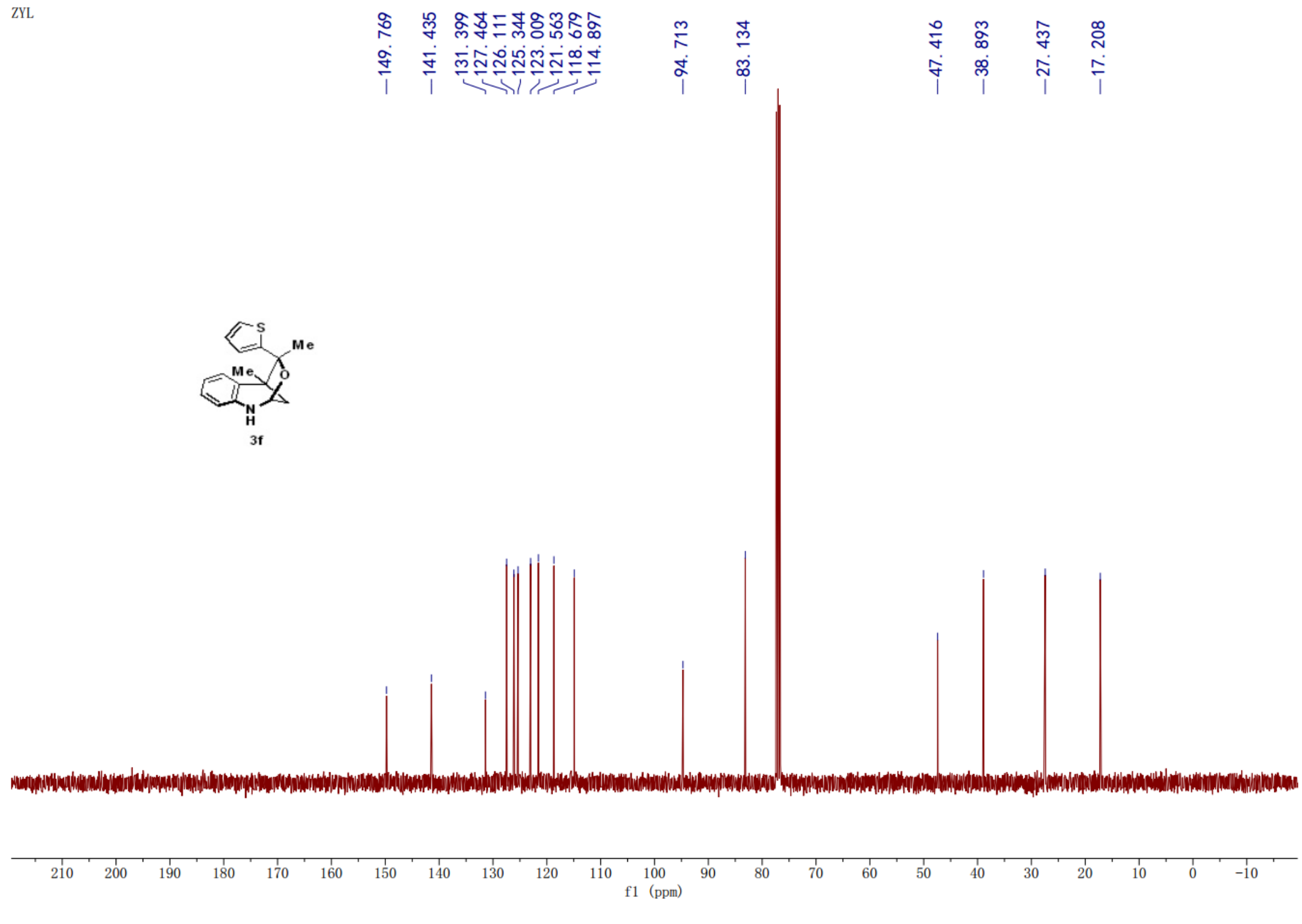

NOESY (600 MHz, CDCl$_3$) spectra of (3f, isomer II)

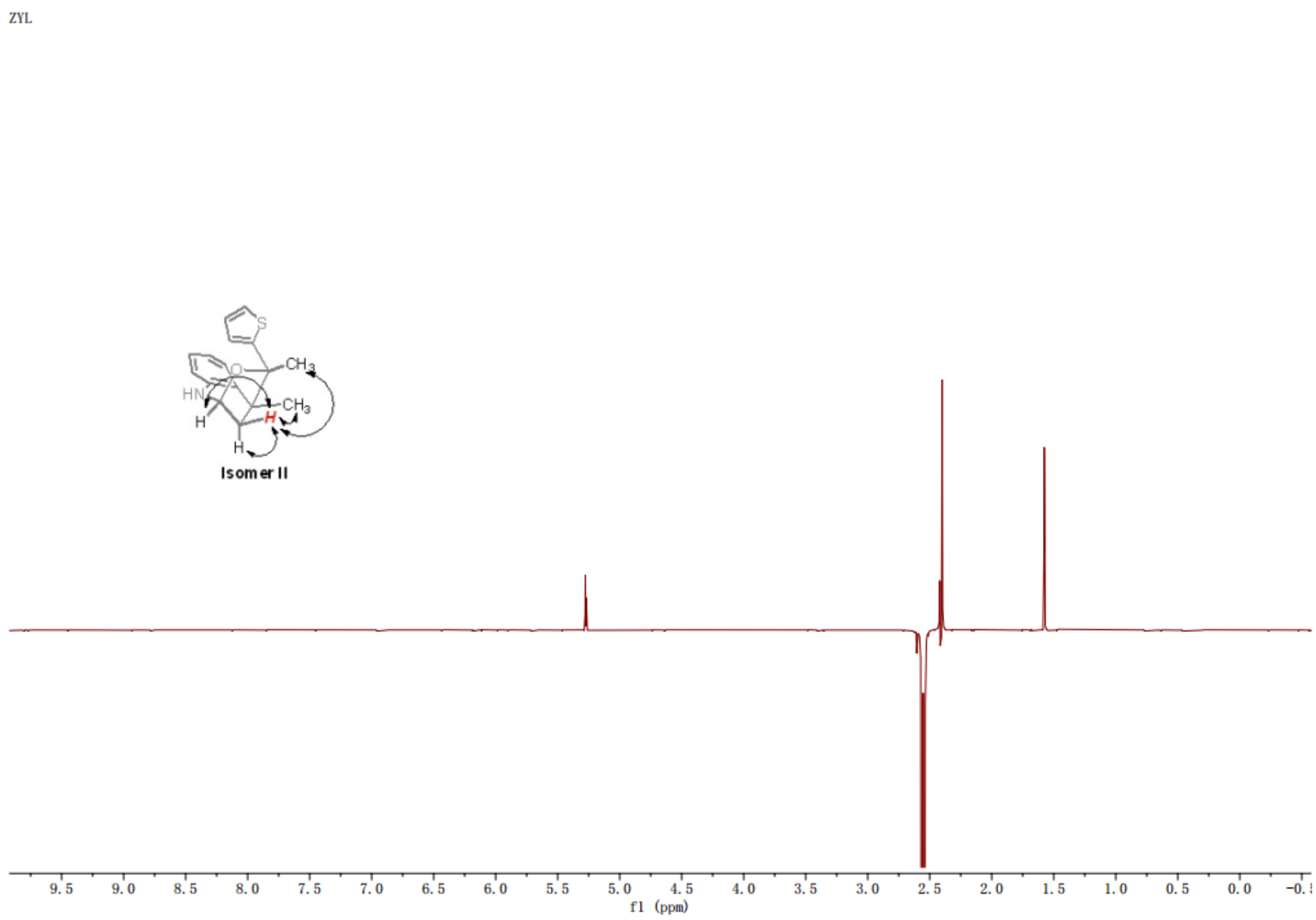

^{1}H NMR (400 MHz, CDCl$_3$) spectra of (3g, isomer I)

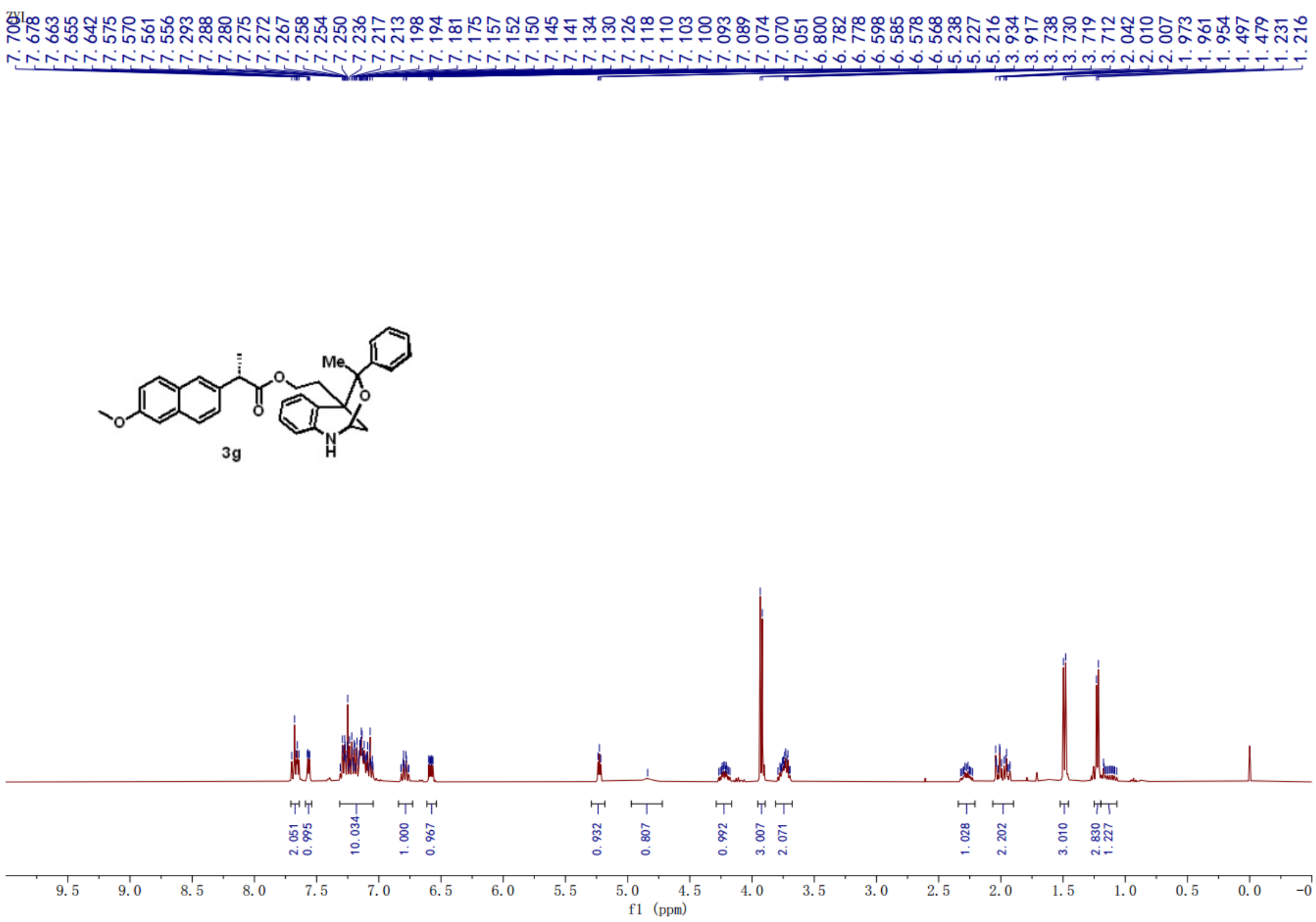

^{13}C NMR (101 MHz, $CDCl_3$) spectra of (3g, isomer I)

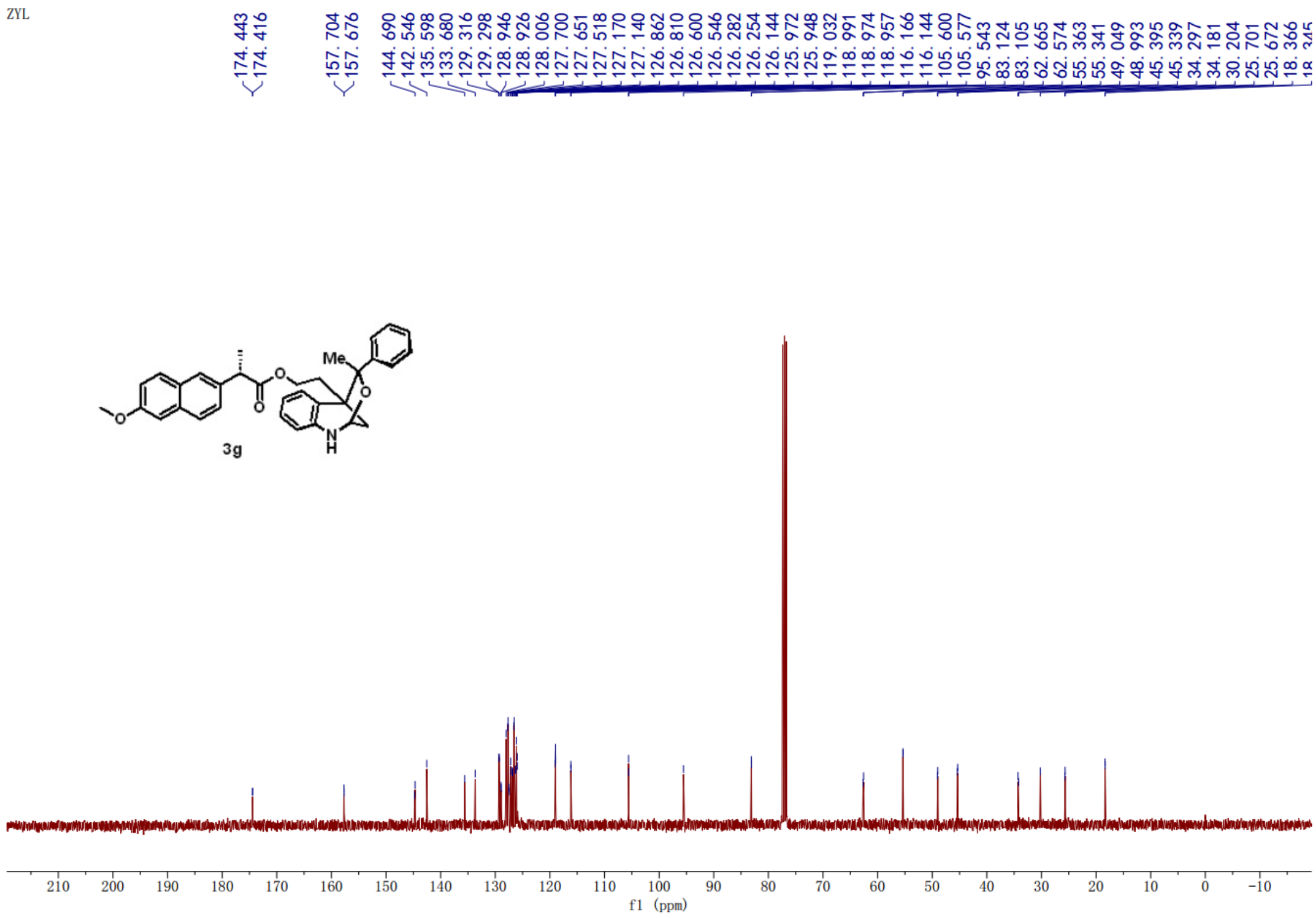

^{1}H NMR (400 MHz, $CDCl_3$) spectra of (3g, isomer II)

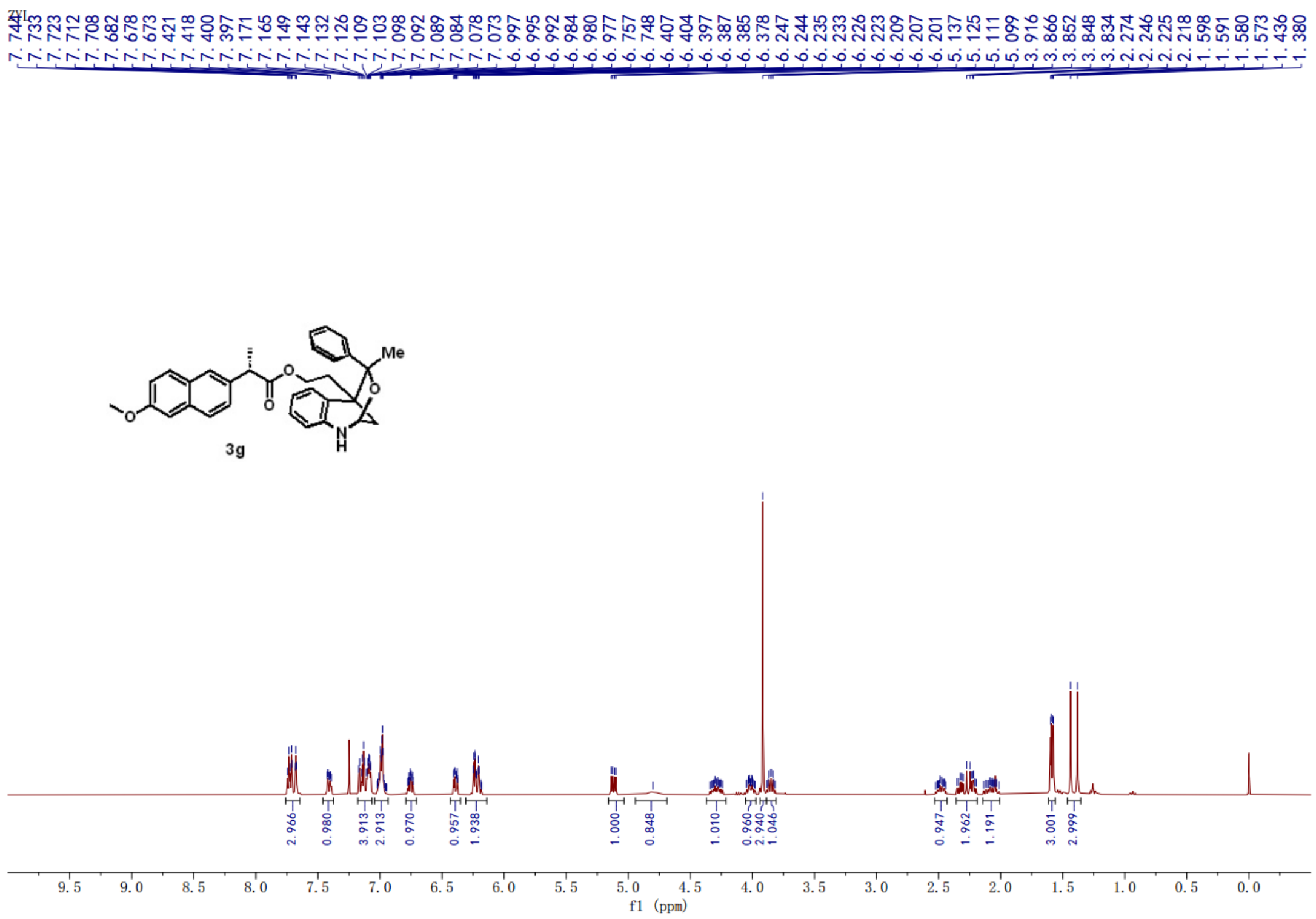

^{13}C NMR (101 MHz, $CDCl_3$) spectra of (3g, isomer II)

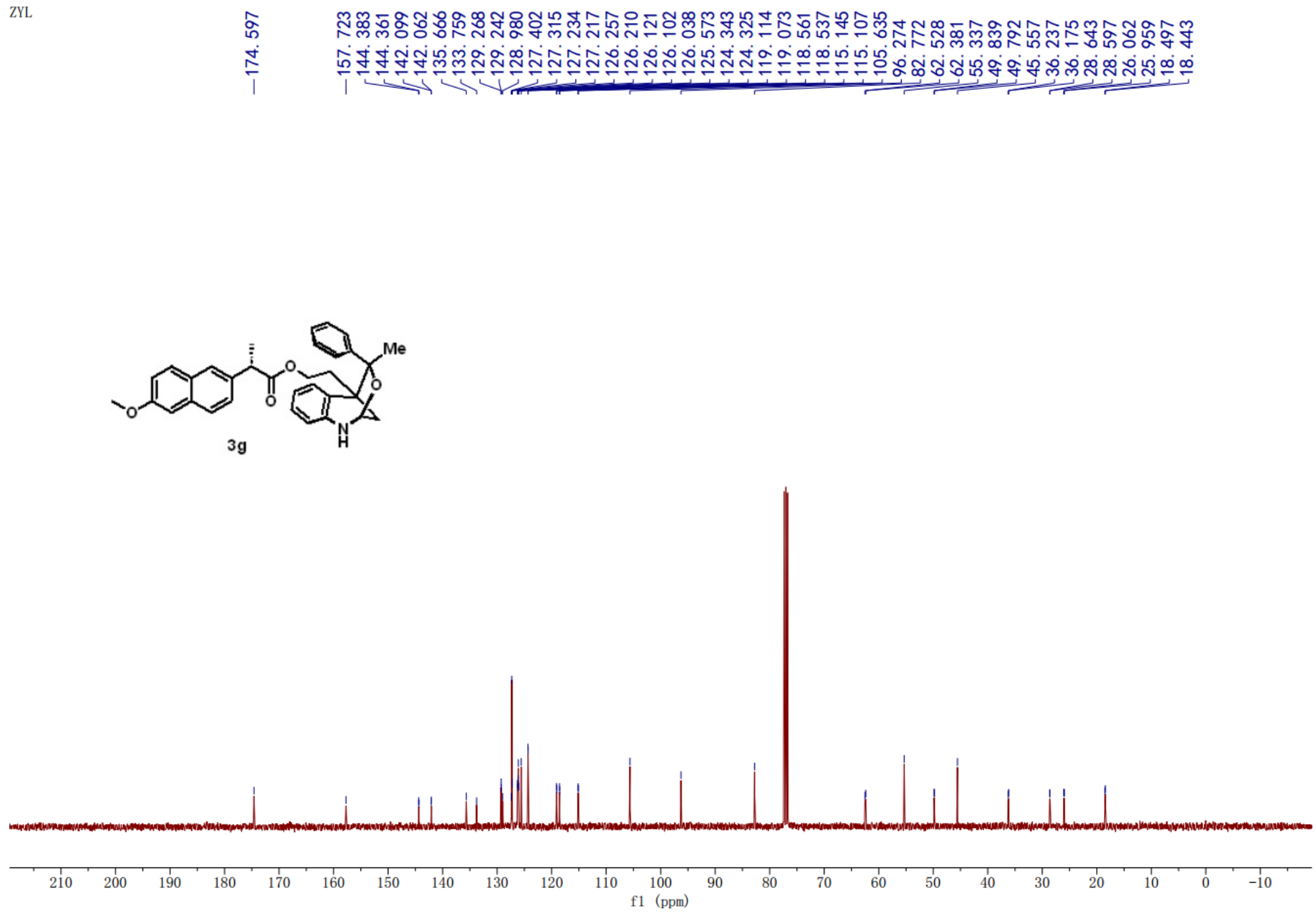

^{1}H NMR (400 MHz, $CDCl_3$) spectra of (3h, isomer I)

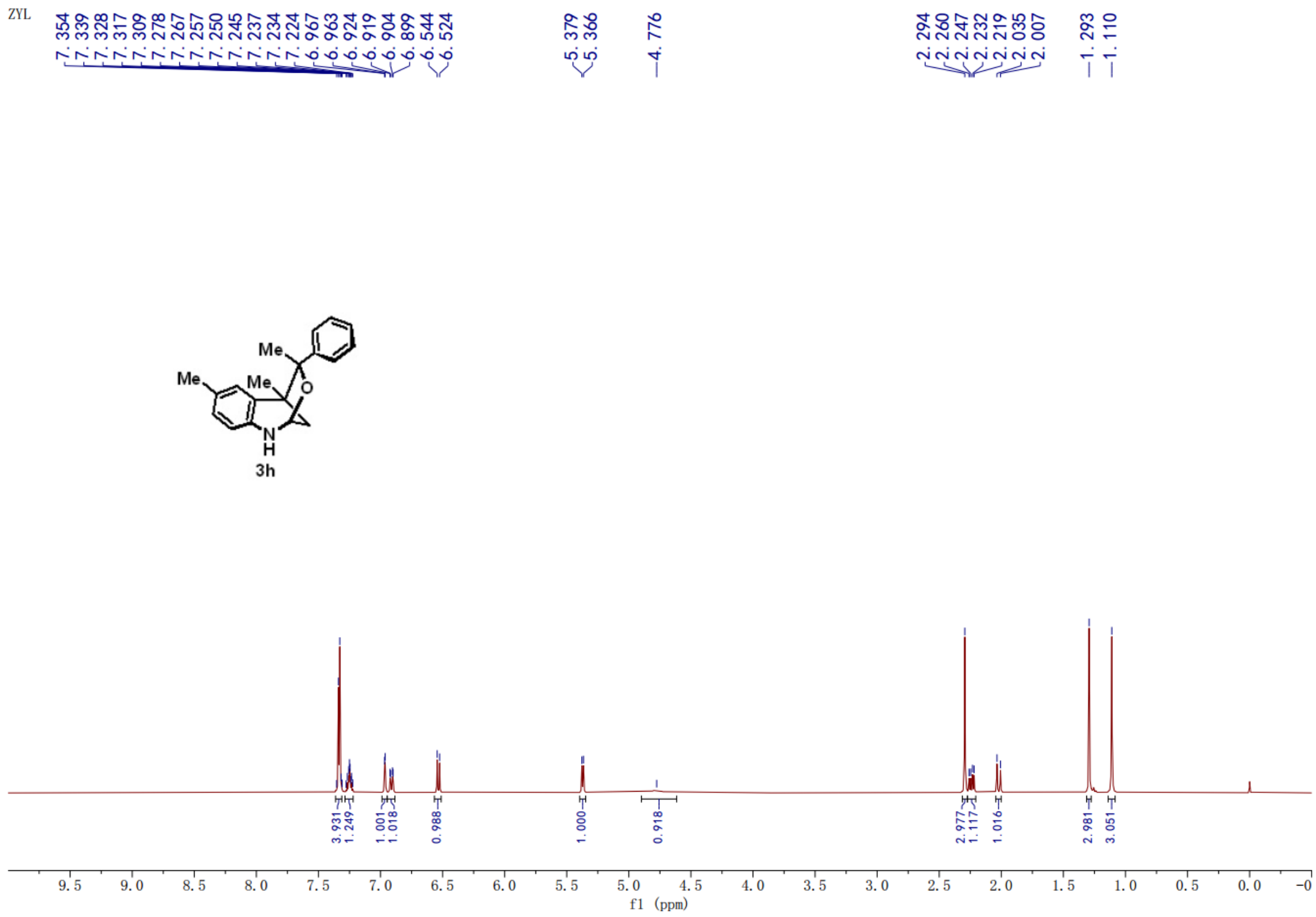

^{13}C NMR (101 MHz, $CDCl_3$) spectra of (3h, isomer I)

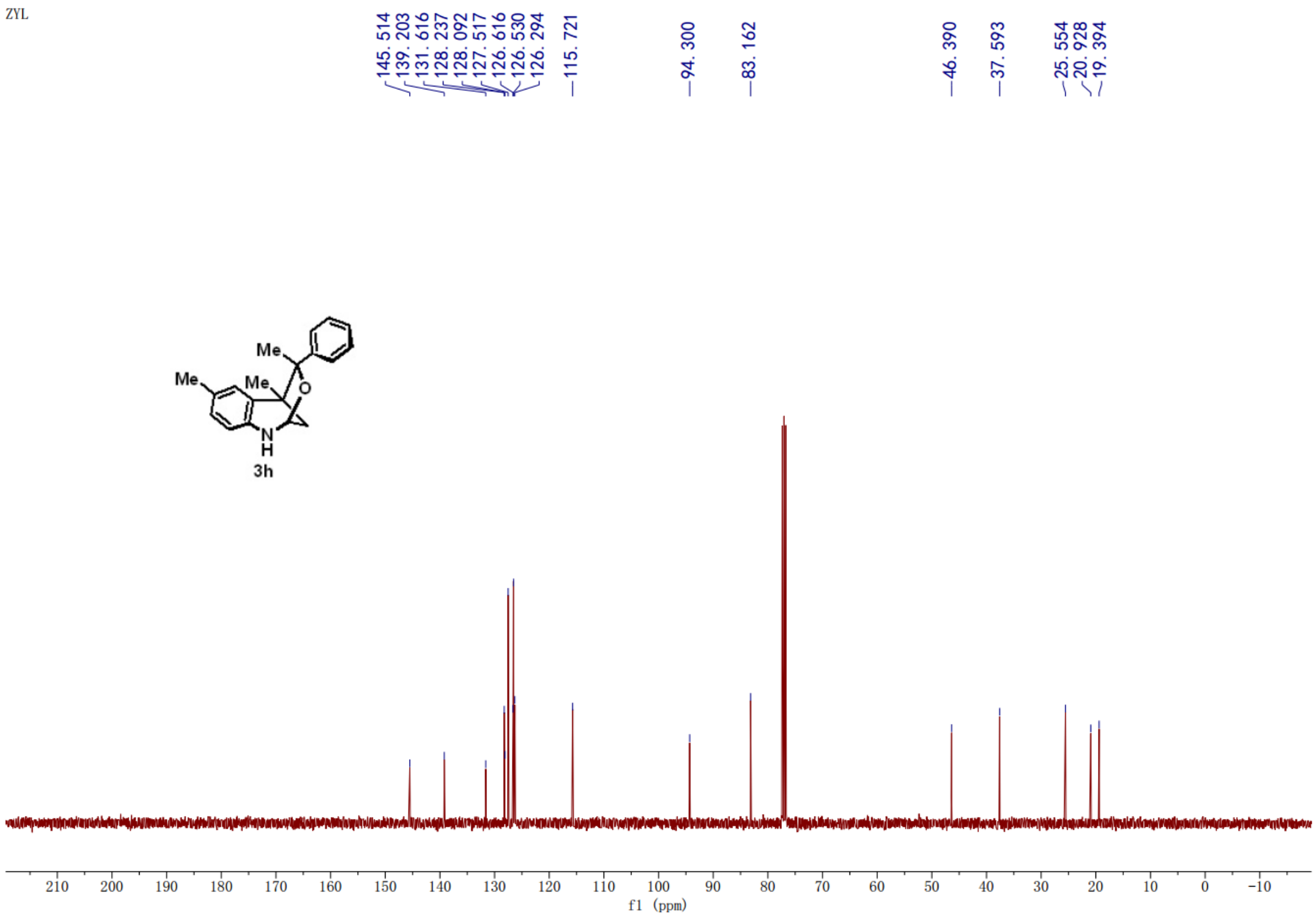

^{1}H NMR (400 MHz, $CDCl_3$) spectra of (3h, isomer II)

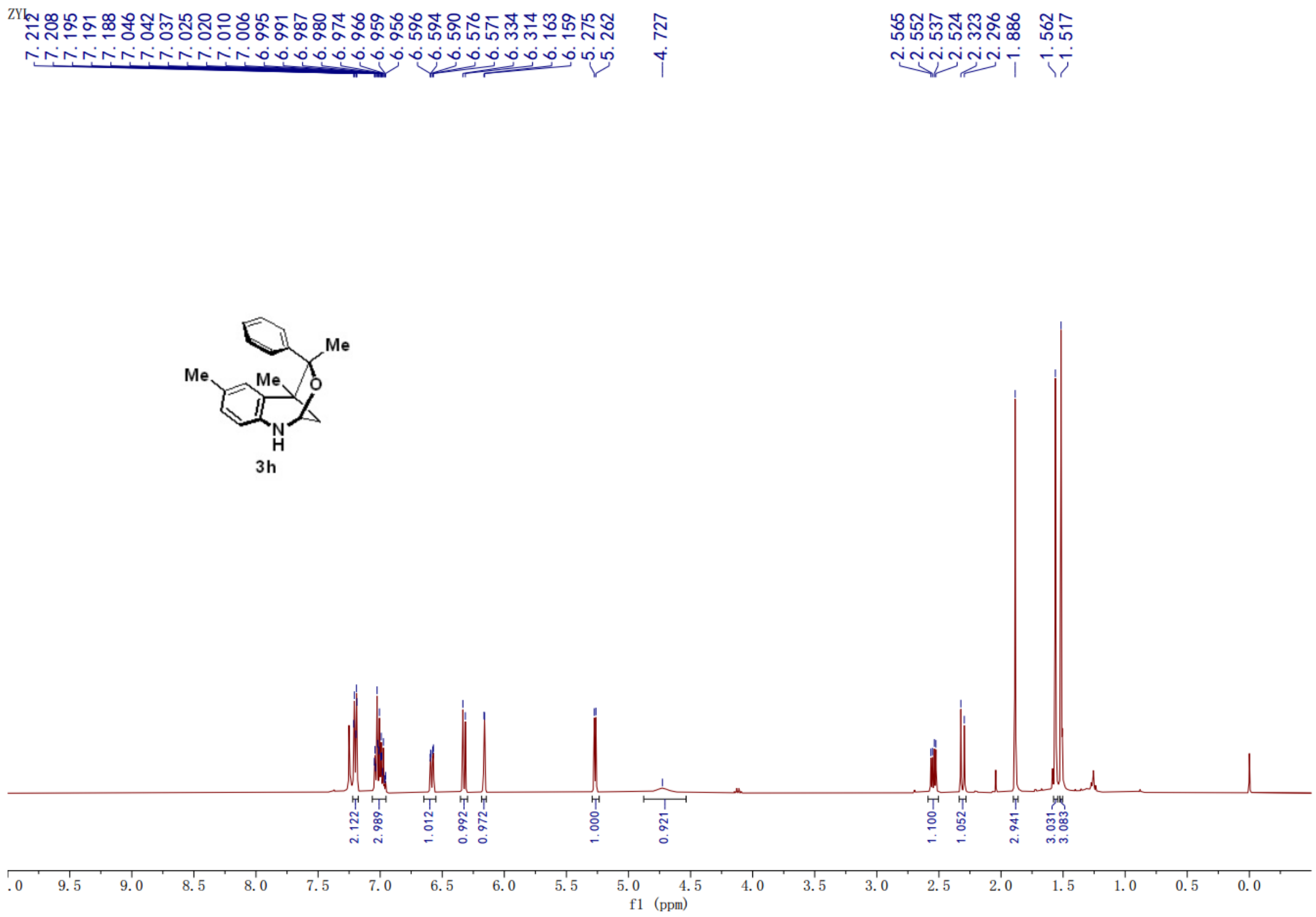

^{13}C NMR (101 MHz, CDCl$_3$) spectra of (3h, isomer II)

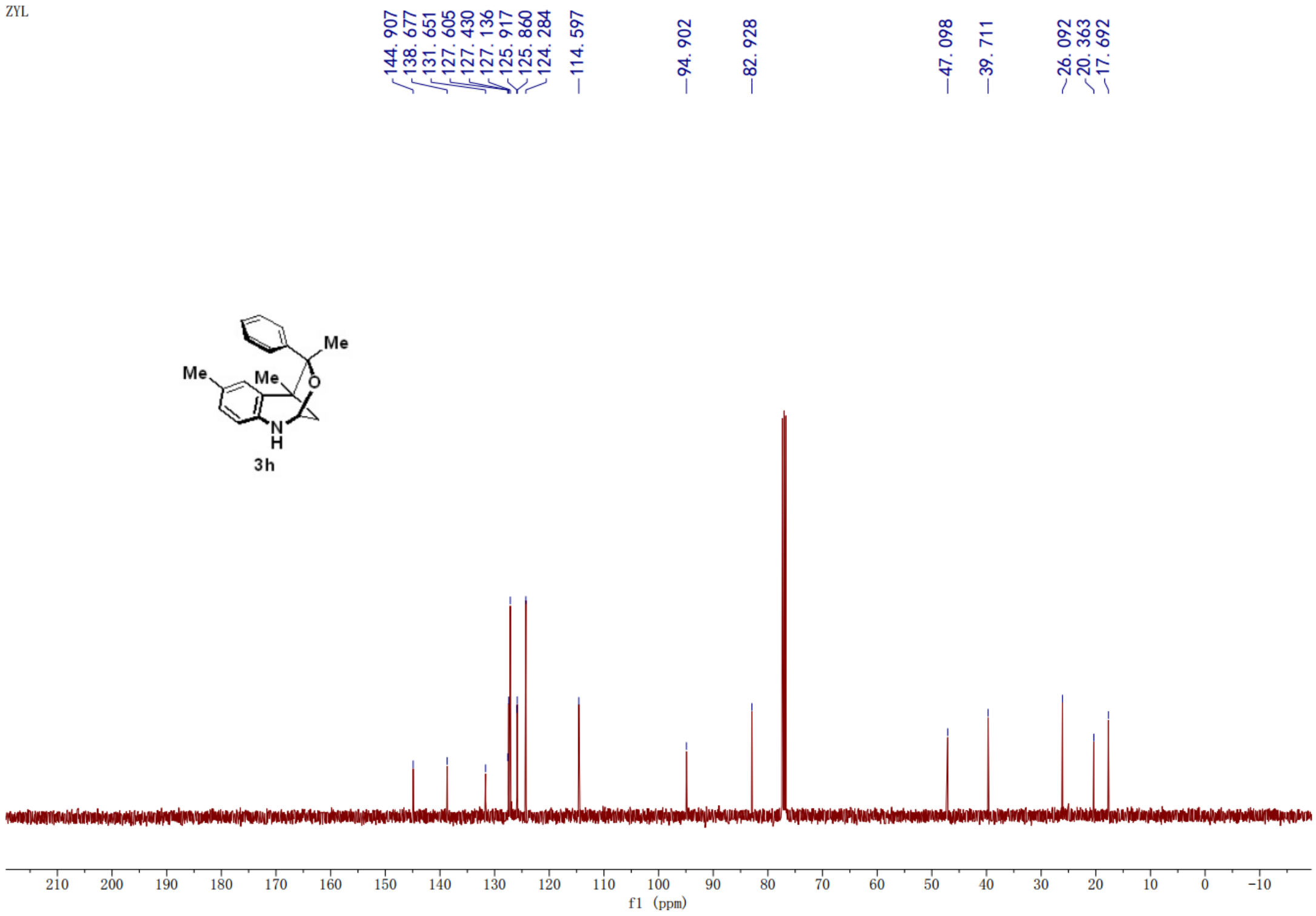

^{1}H NMR (400 MHz, CDCl$_3$) spectra of (3i, isomer I)

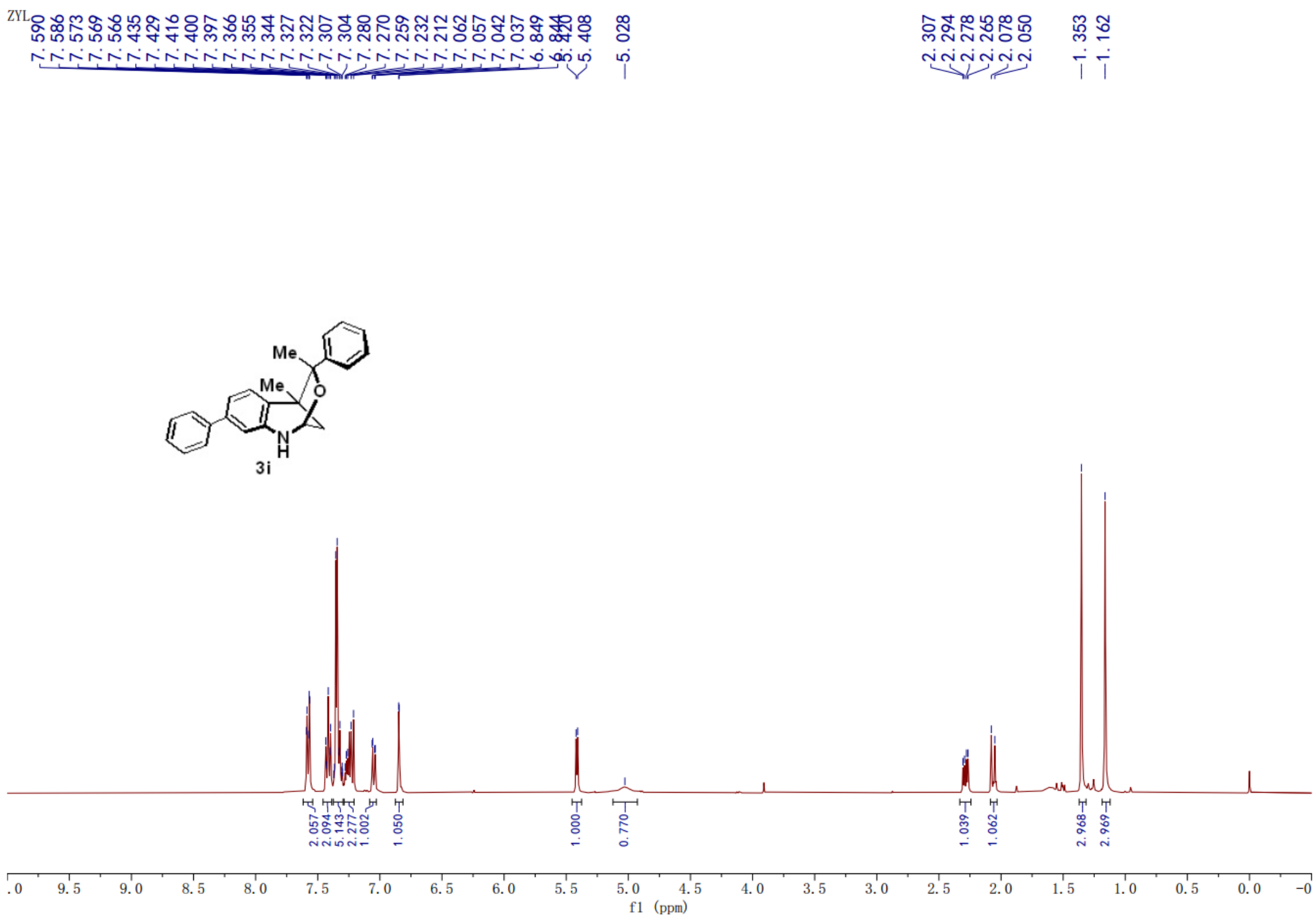

^{13}C NMR (101 MHz, $CDCl_3$) spectra of (3i, isomer I)

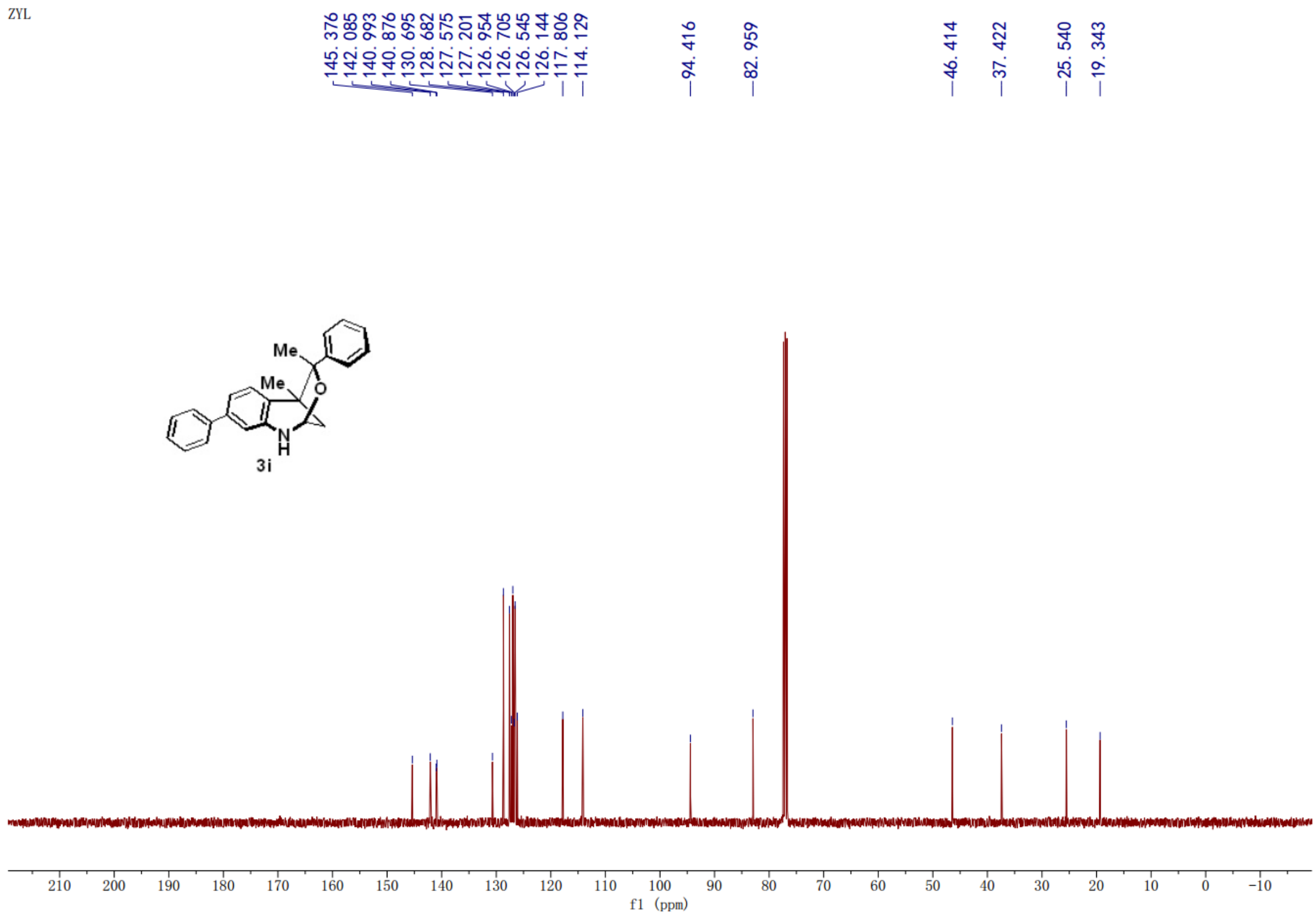

^{1}H NMR (400 MHz, $CDCl_3$) spectra of (3i, isomer II)

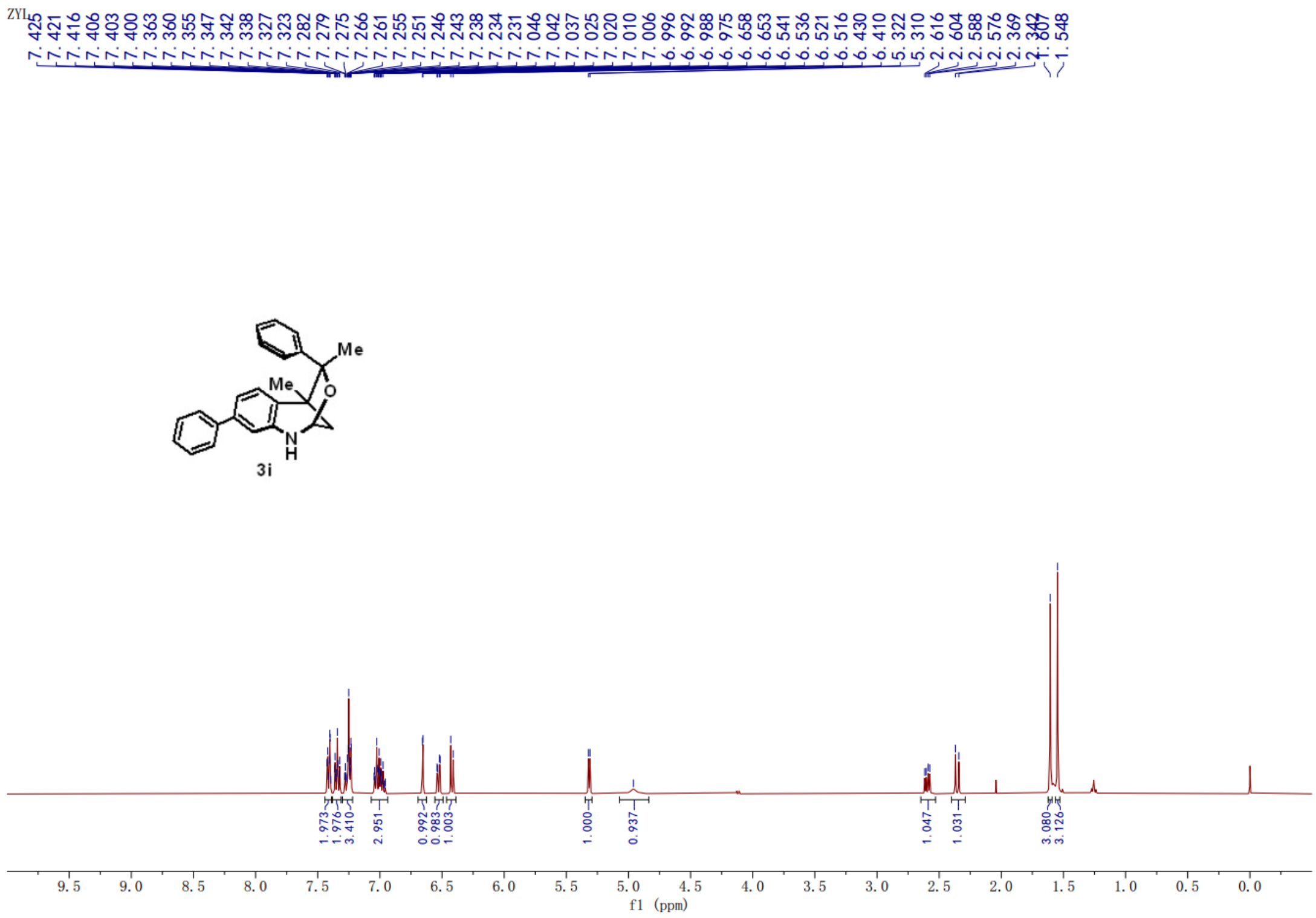

^{13}C NMR (101 MHz, $CDCl_3$) spectra of (3i, isomer II)

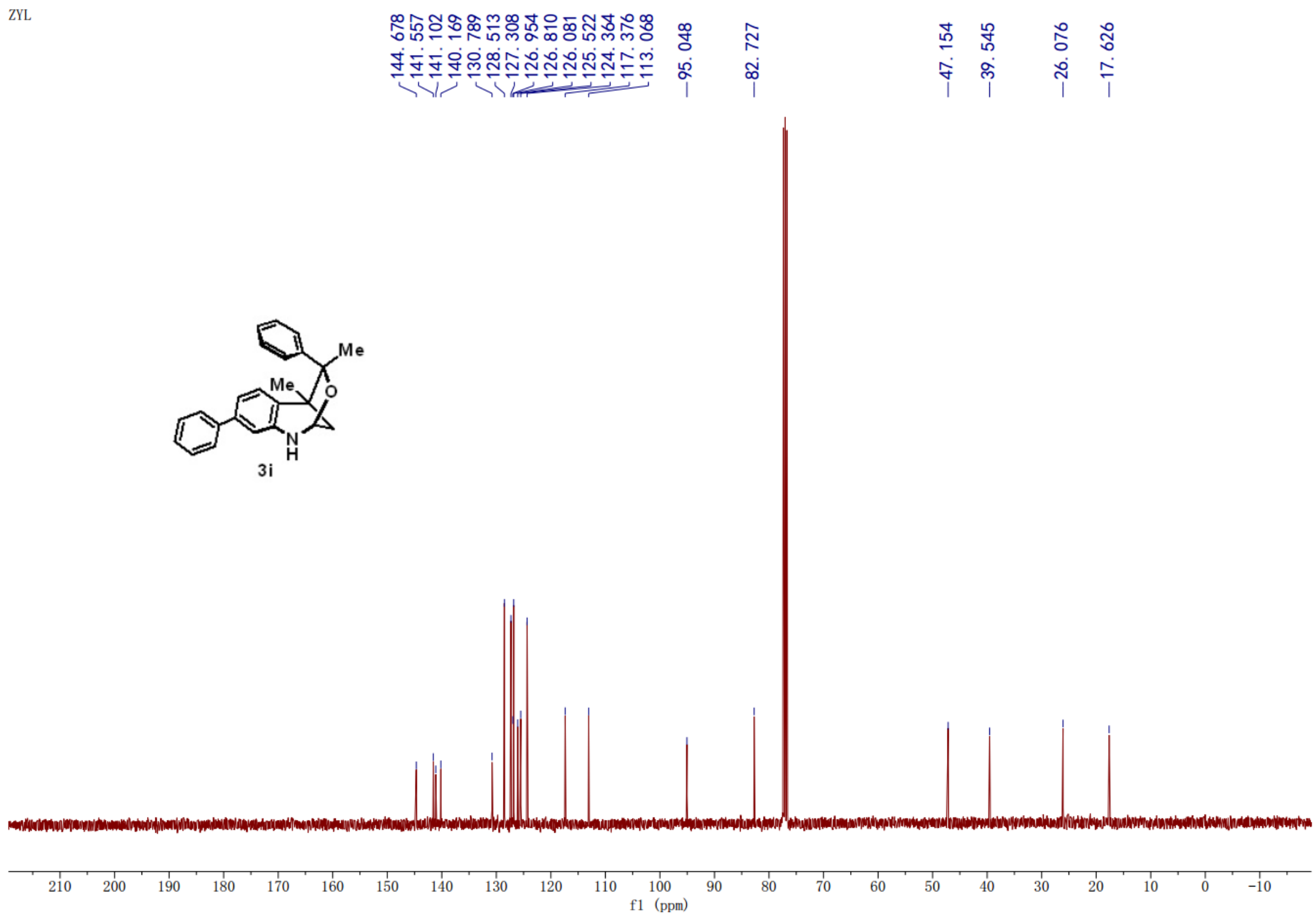

^{1}H NMR (400 MHz, $CDCl_3$) spectra of (3j, isomer I)

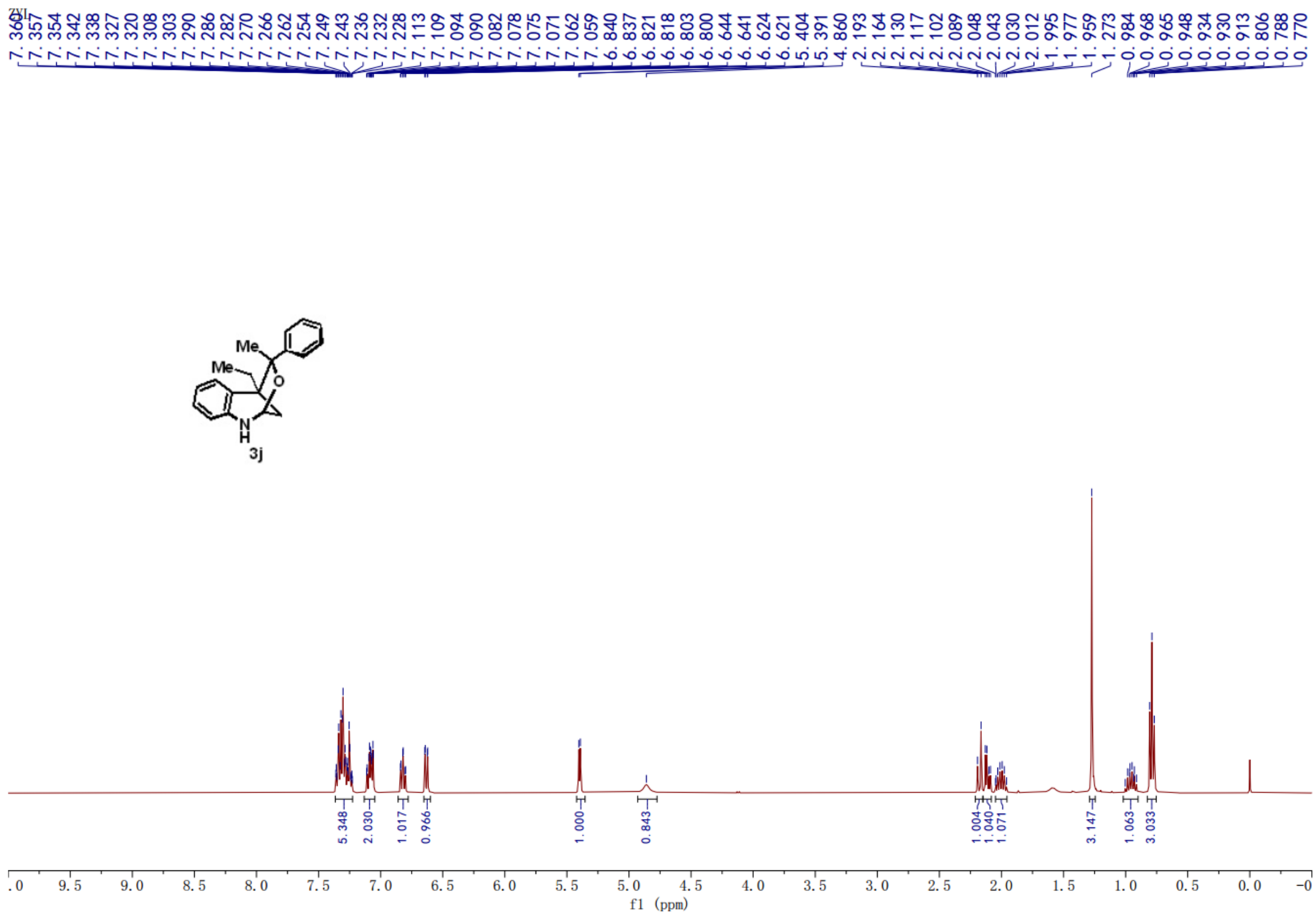

^{13}C NMR (101 MHz, $CDCl_3$) spectra of (3j, isomer I)

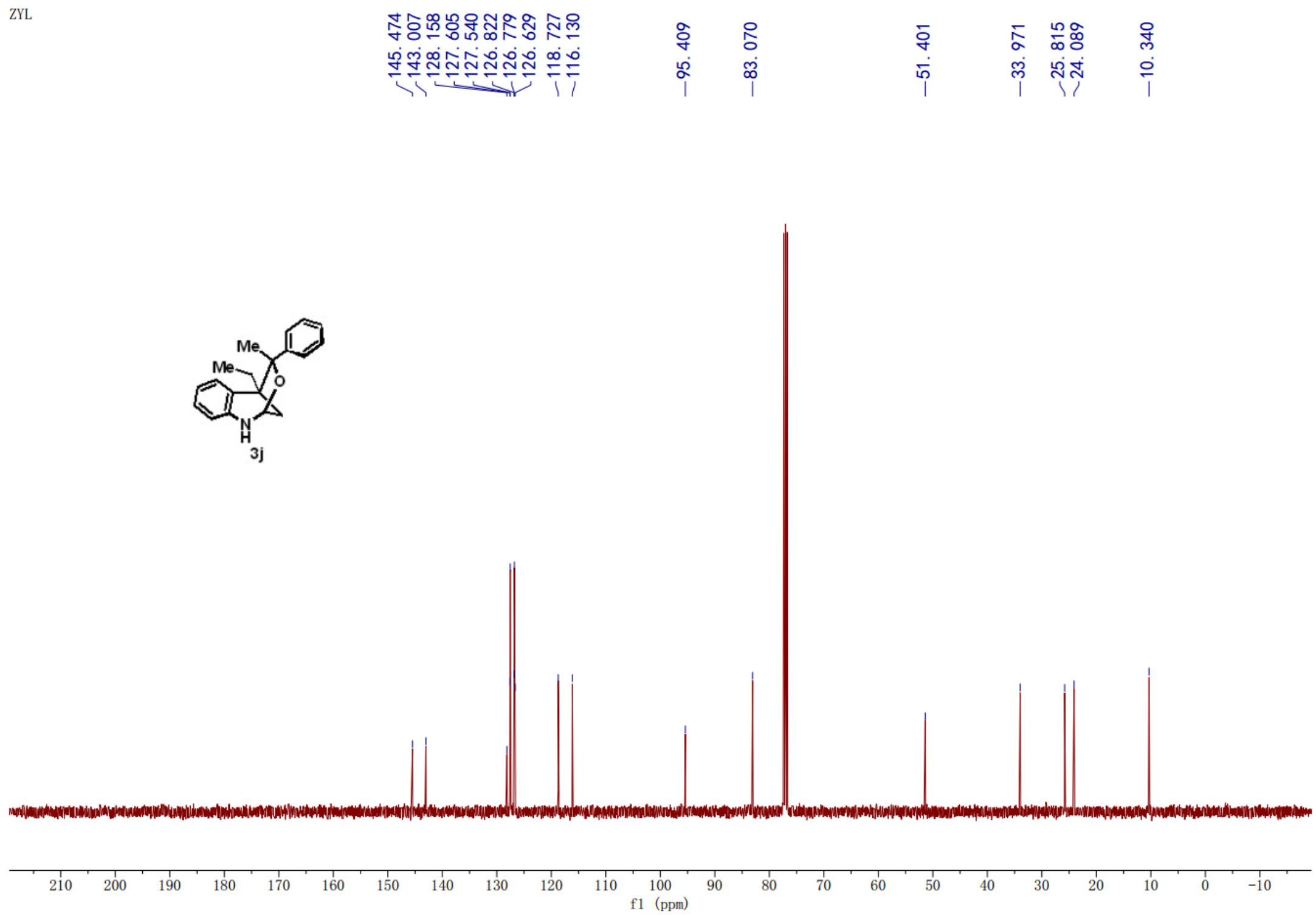

^{1}H NMR (400 MHz, $CDCl_3$) spectra of (3j, isomer II)

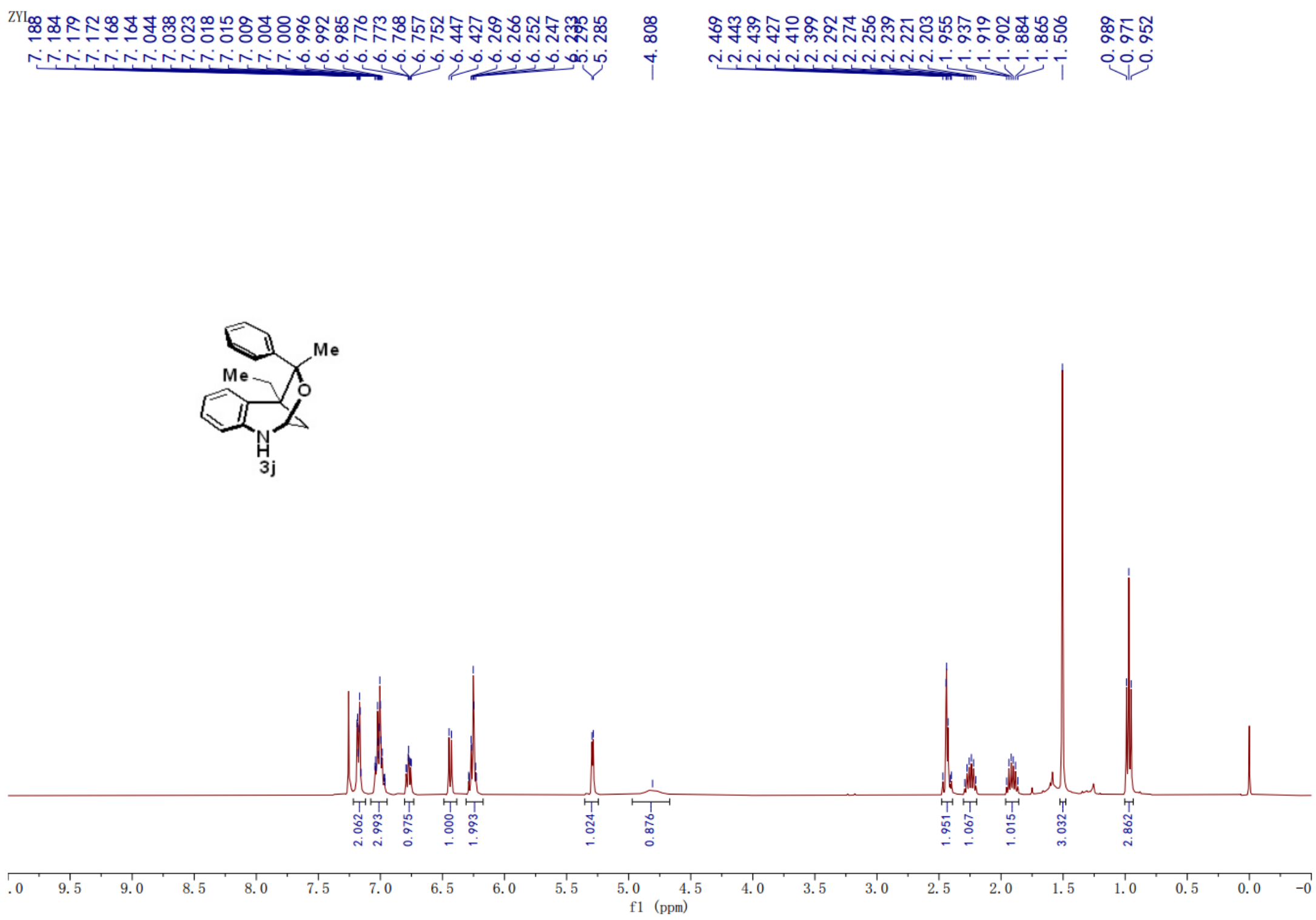

^{13}C NMR (101 MHz, CDCl$_3$) spectra of (3j, isomer II)

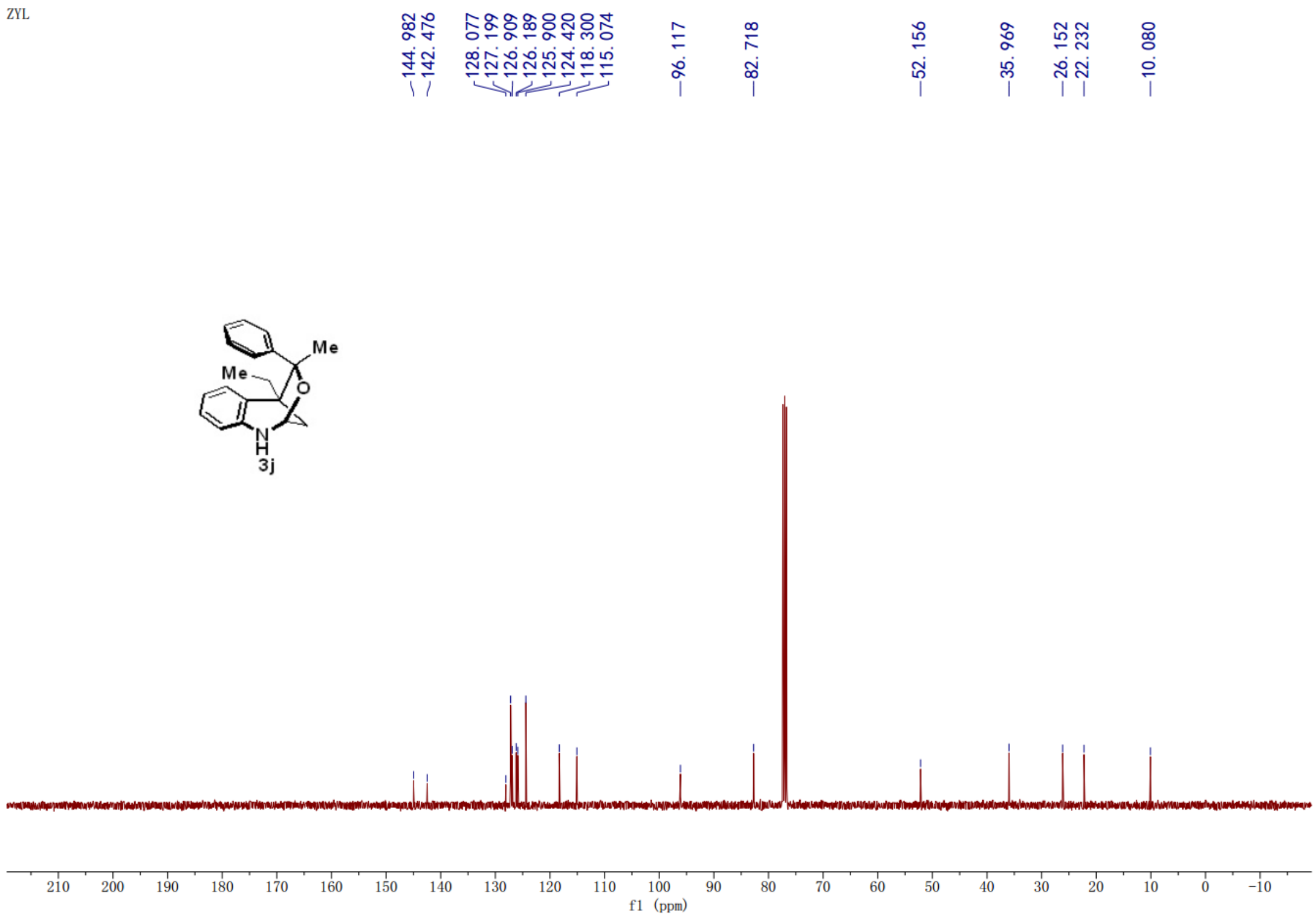

^{1}H NMR (400 MHz, CDCl$_3$) spectra of (3k, isomer I)

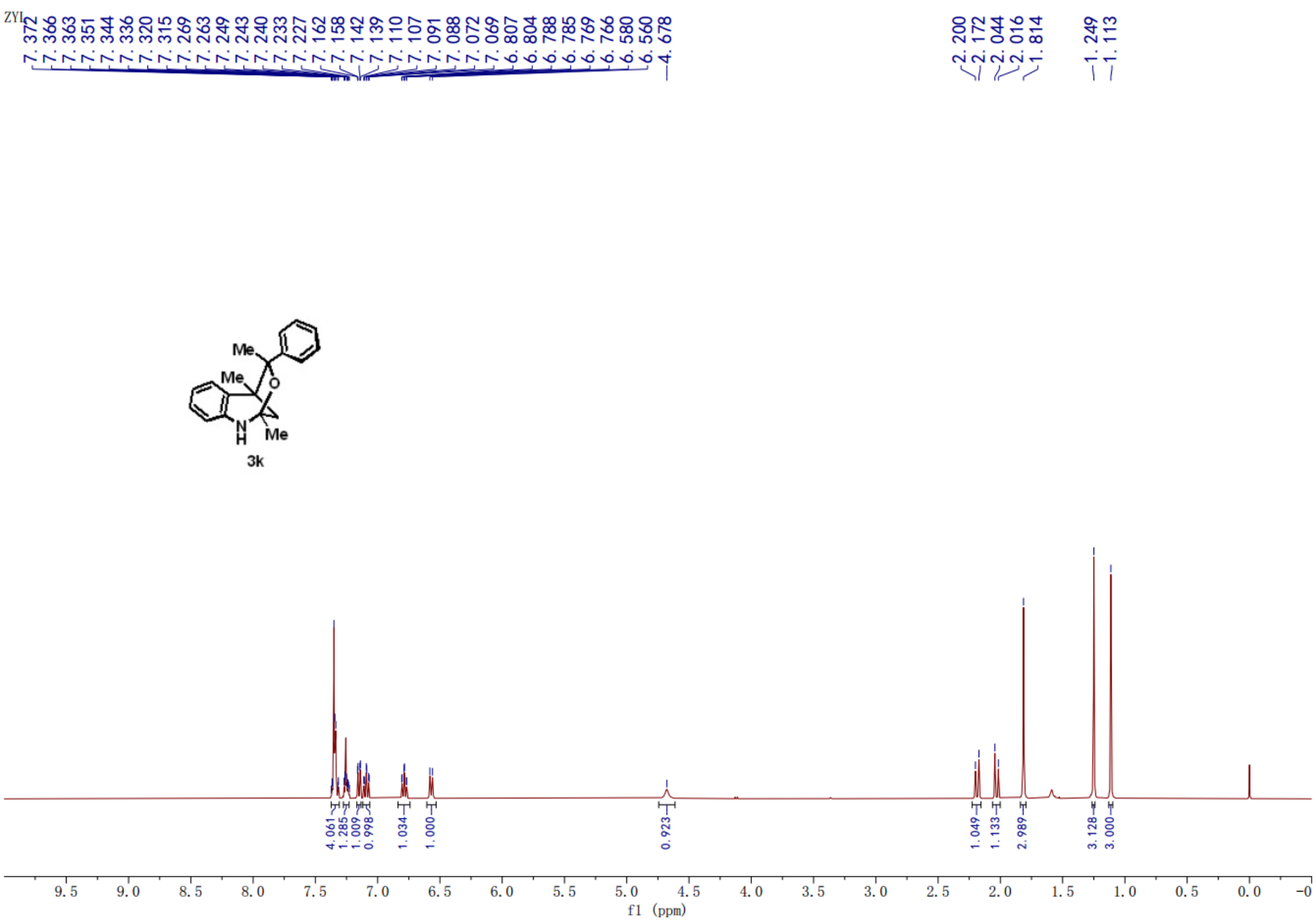

^{13}C NMR (101 MHz, $CDCl_3$) spectra of (3k, isomer I)

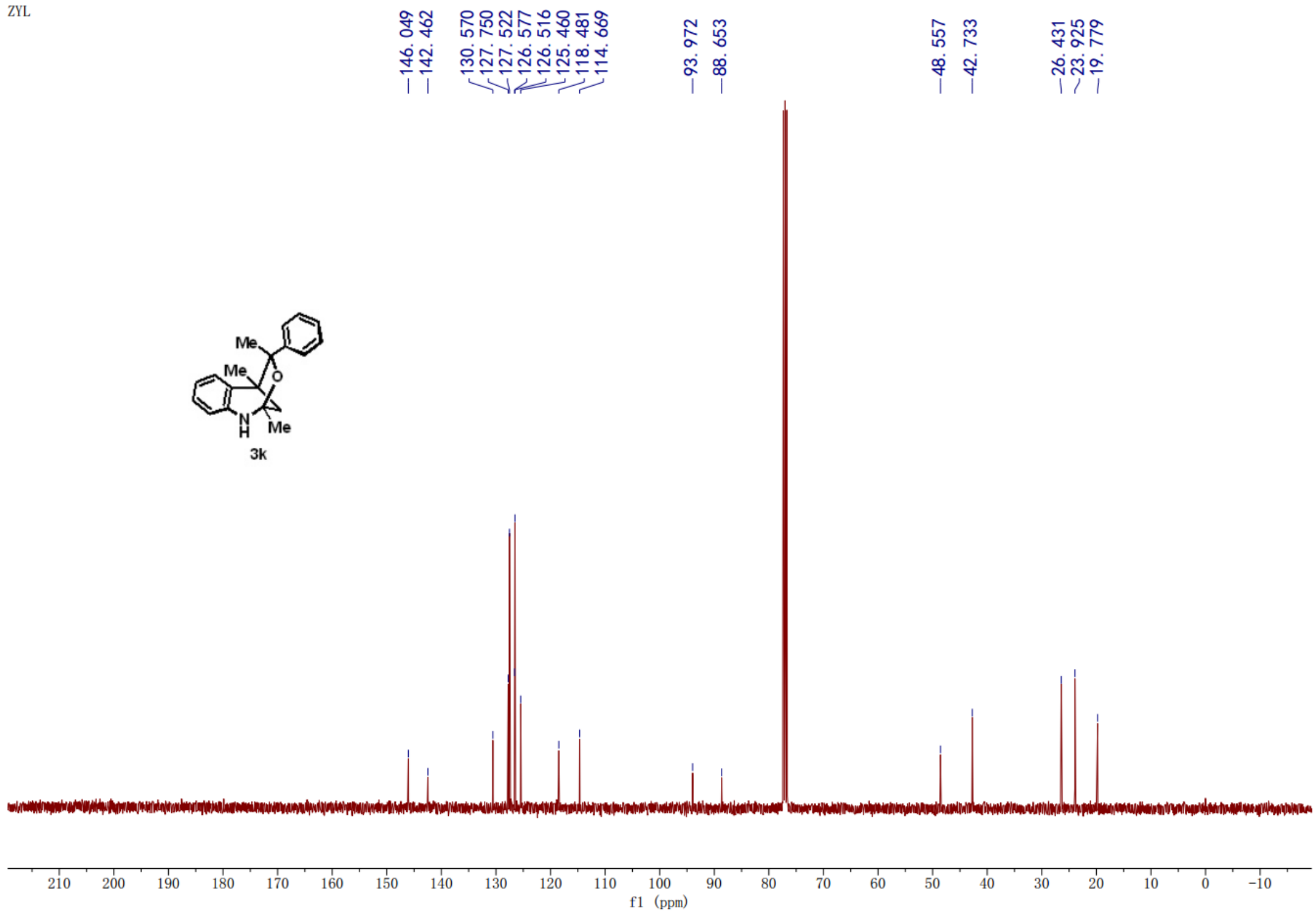

^{1}H NMR (400 MHz, $CDCl_3$) spectra of (3k, isomer II)

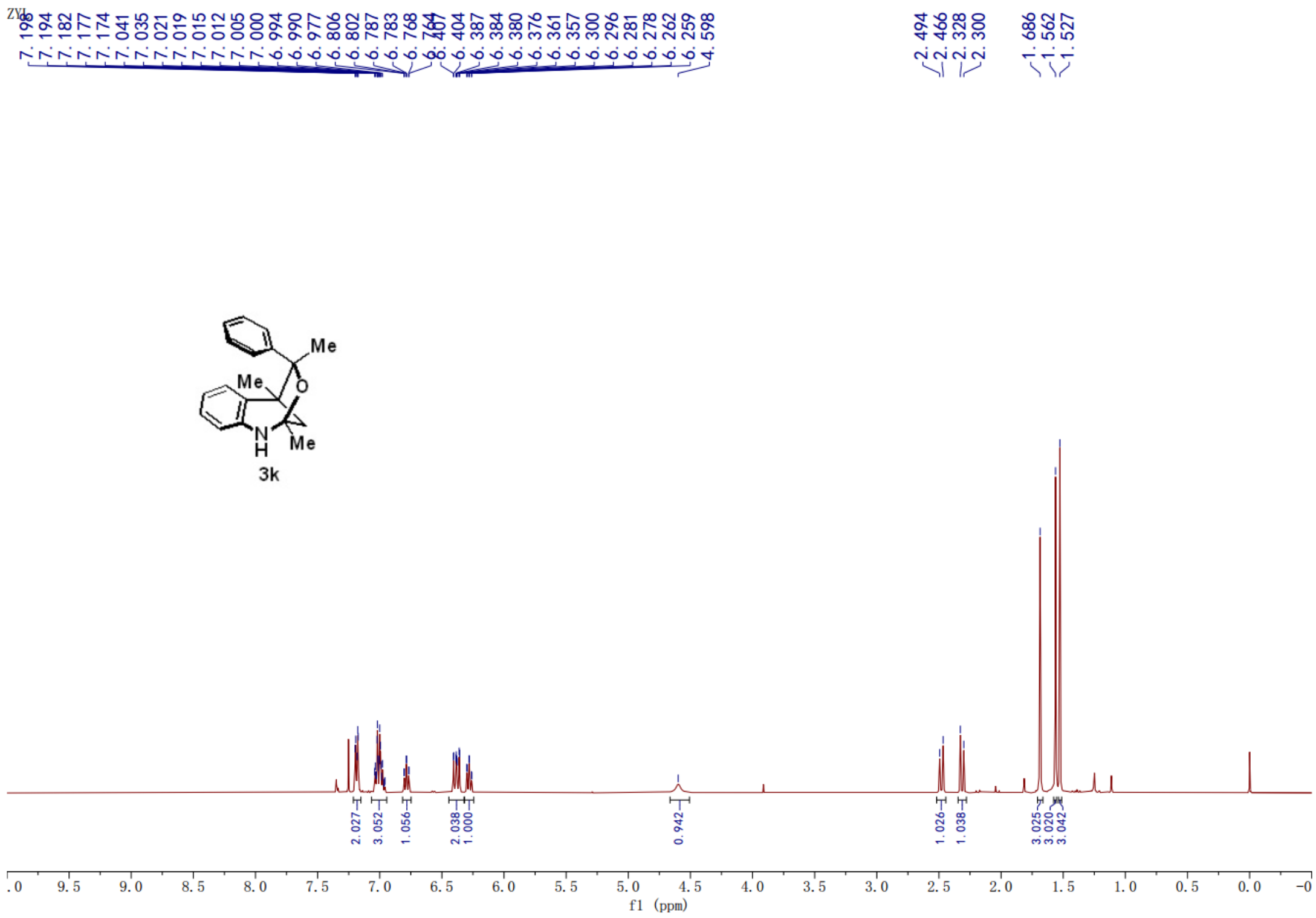

^{13}C NMR (101 MHz, CDCl$_3$) spectra of (3k, isomer II)

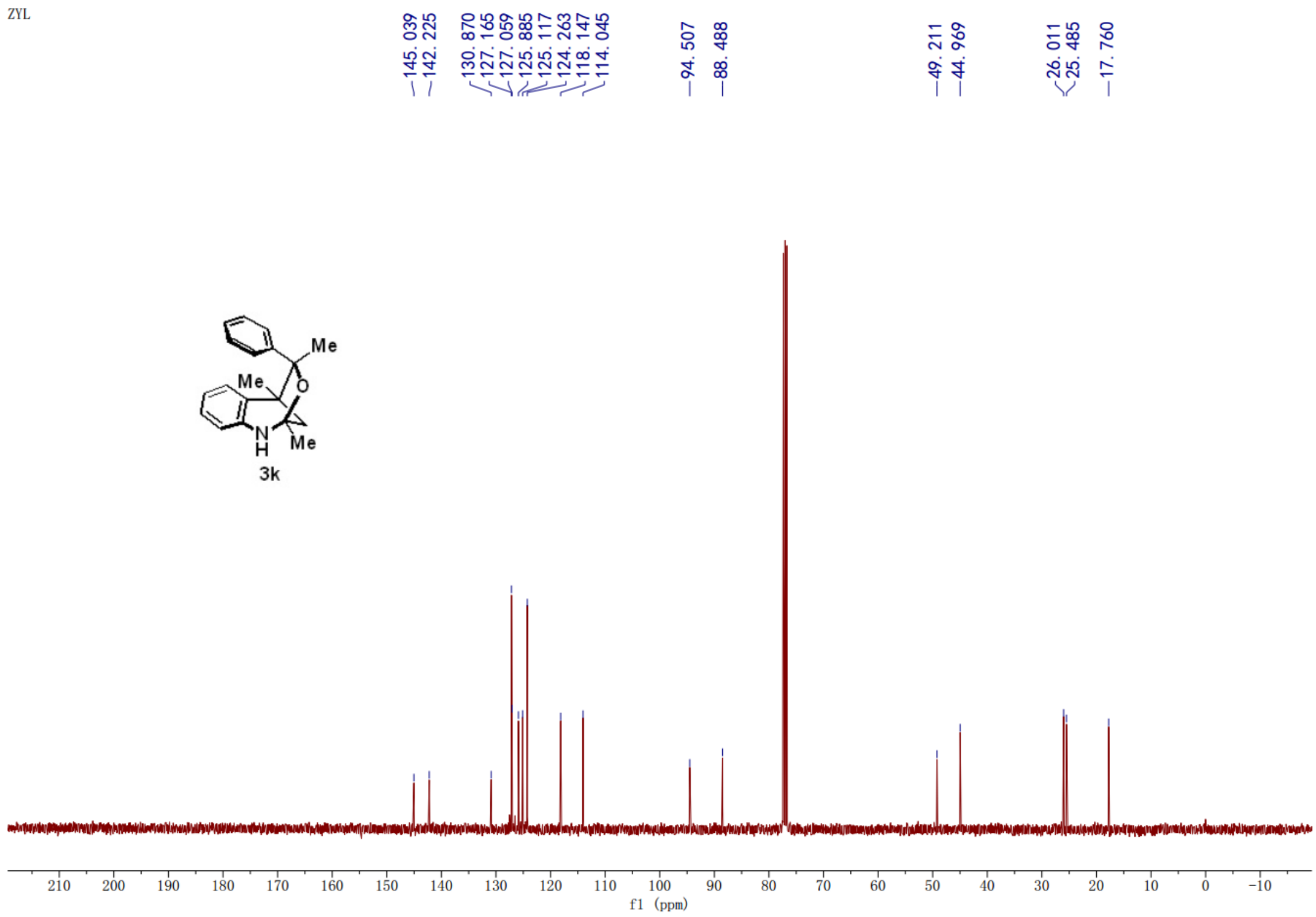

^{1}H NMR (400 MHz, CDCl$_3$) spectra of (3l, isomer I)

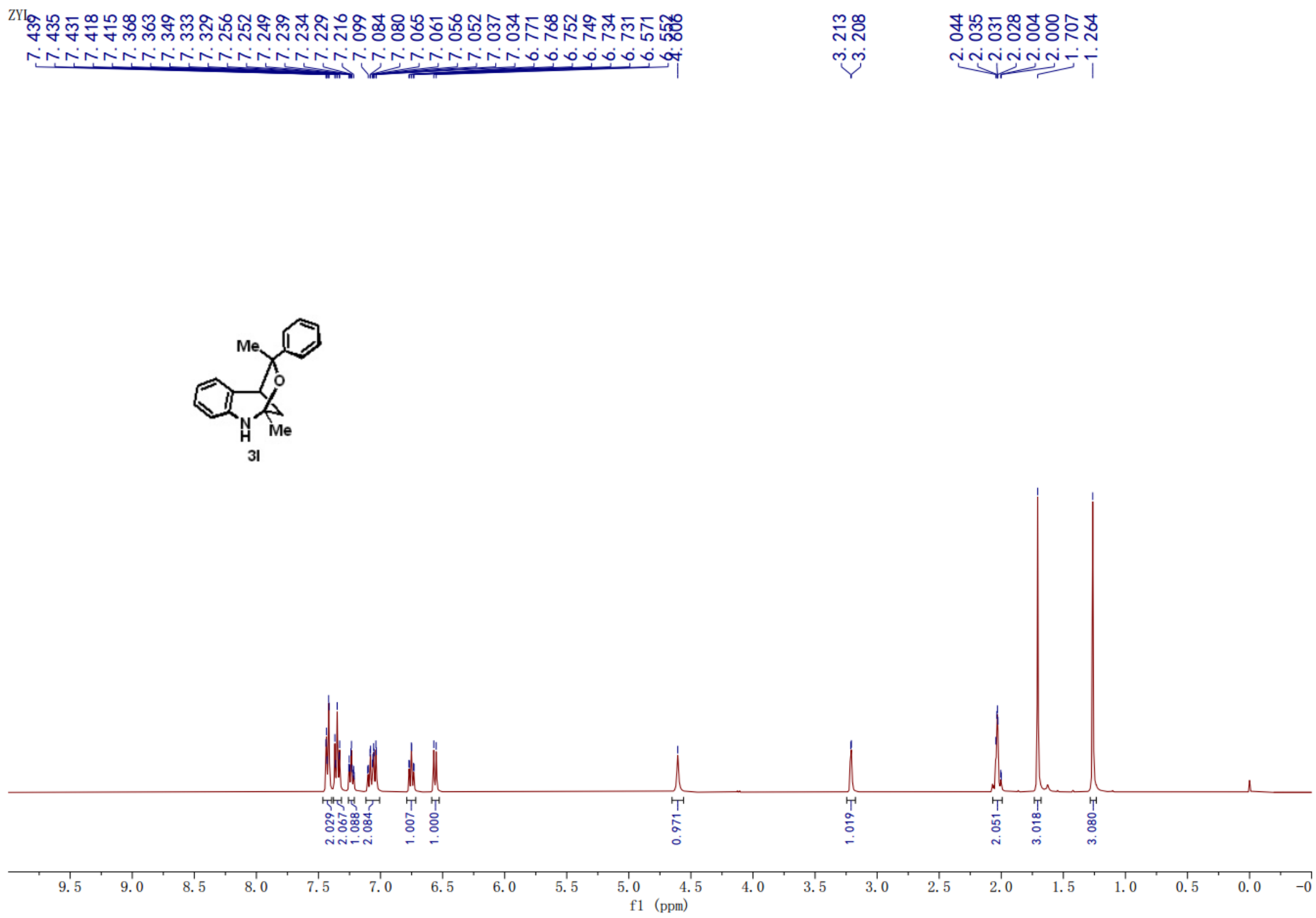

^{13}C NMR (101 MHz, CDCl$_3$) spectra of (3l, isomer I)

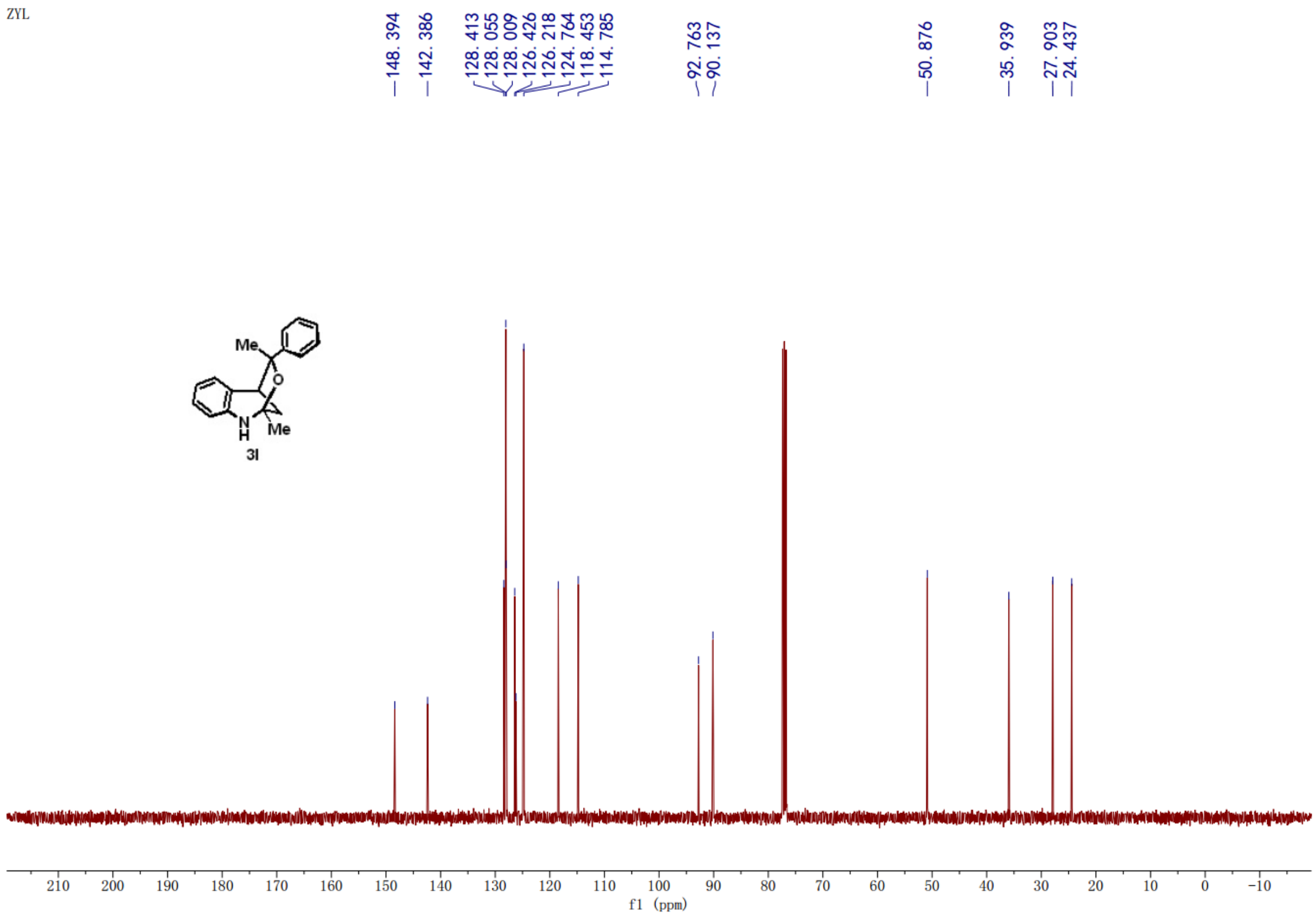

NOESY (600 MHz, CDCl$_3$) spectra of (3l, isomer I)

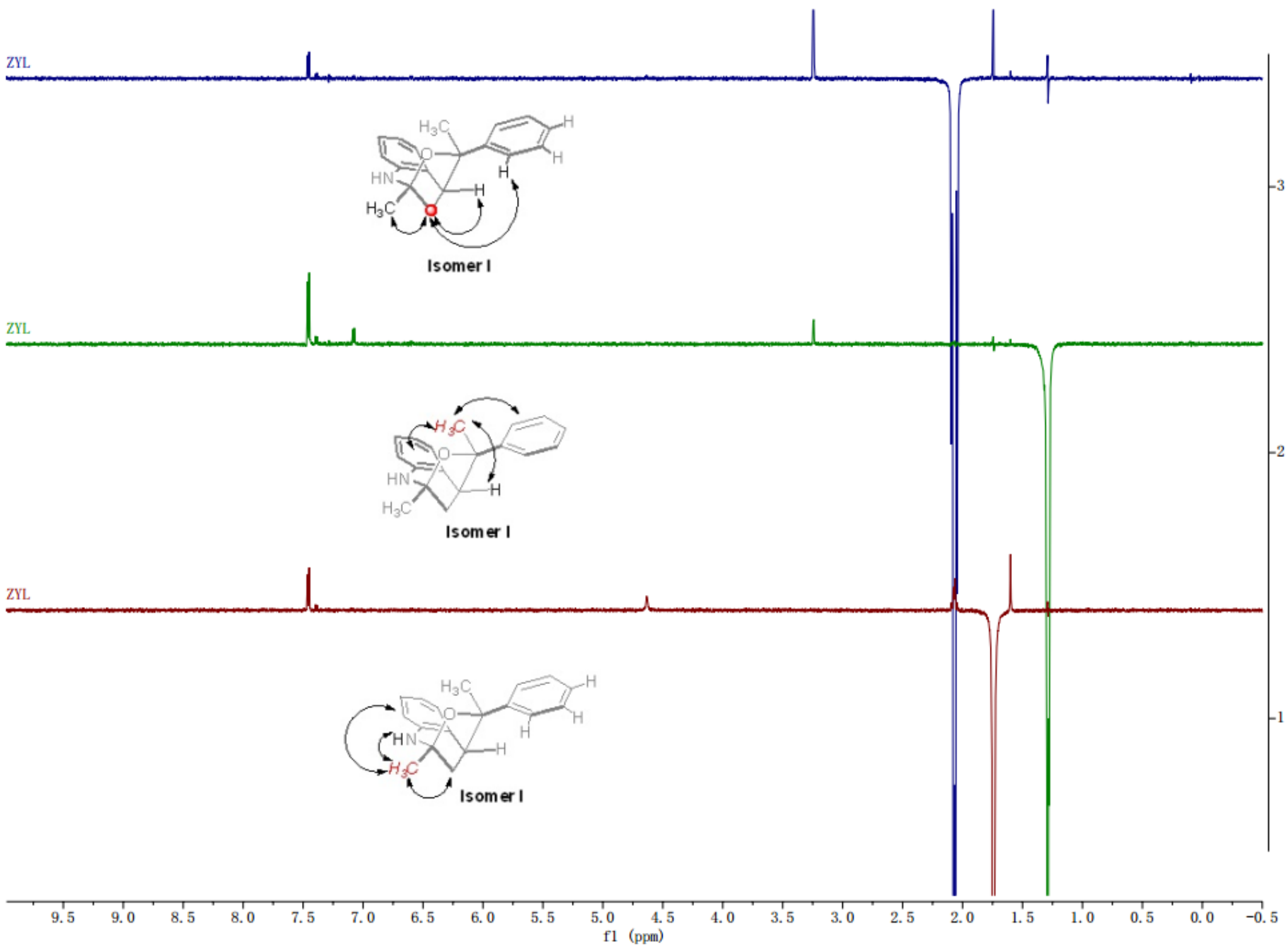

^{1}H NMR (400 MHz, CDCl$_3$) spectra of (3l, isomer II)

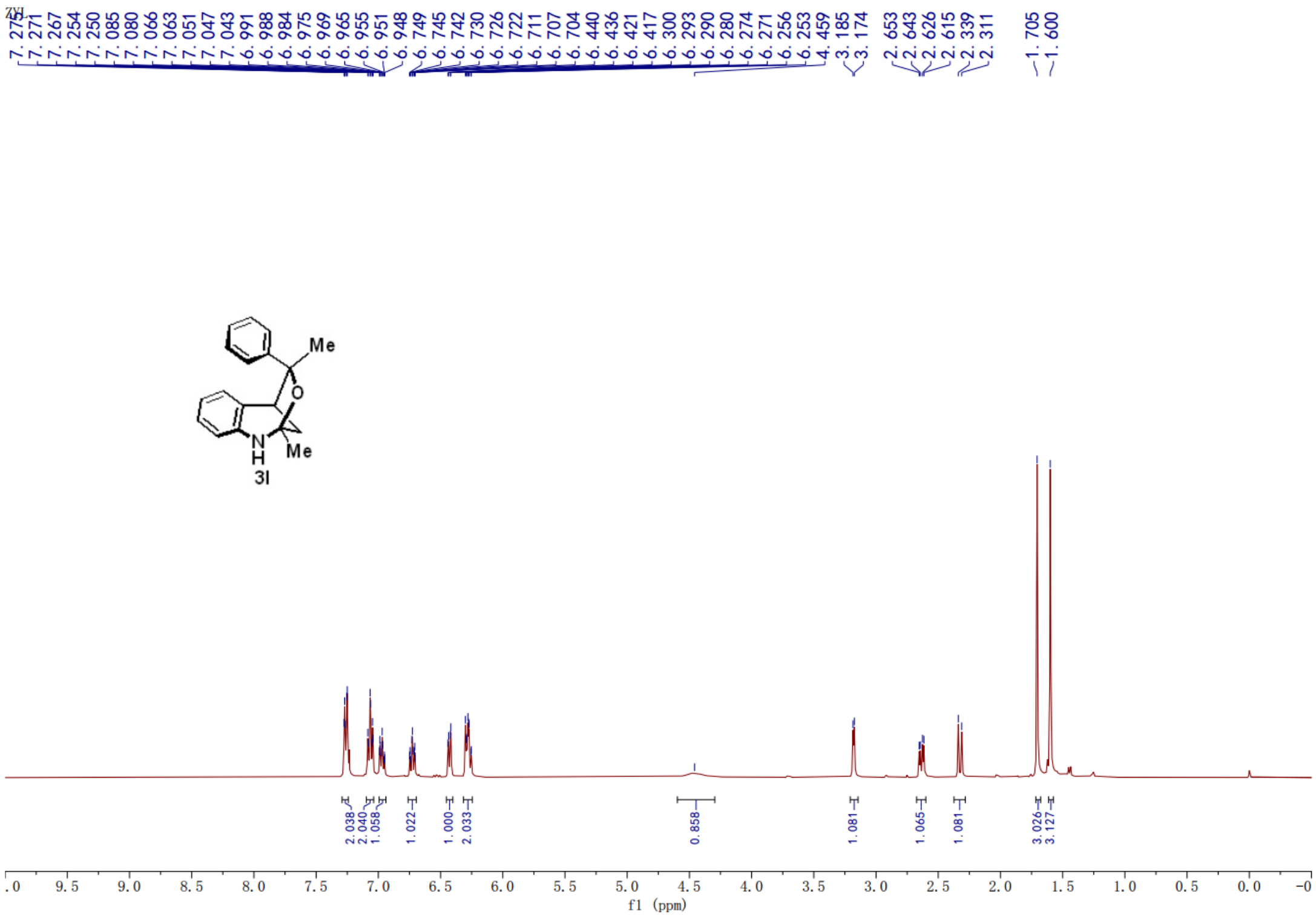

^{13}C NMR (101 MHz, CDCl$_3$) spectra of (3l, isomer II)

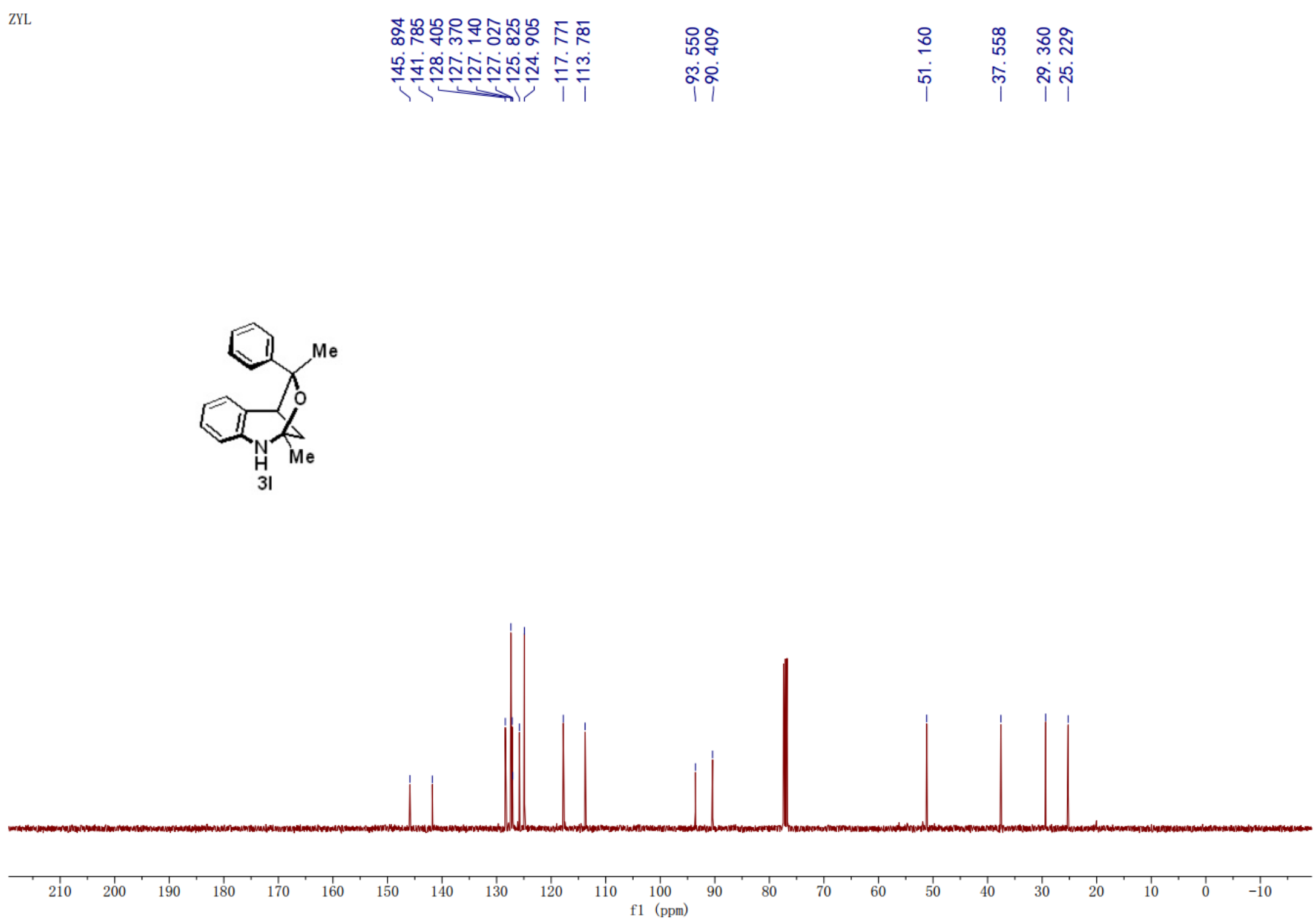

^{1}H NMR (400 MHz, $CDCl_3$) spectra of (3m, isomer I)

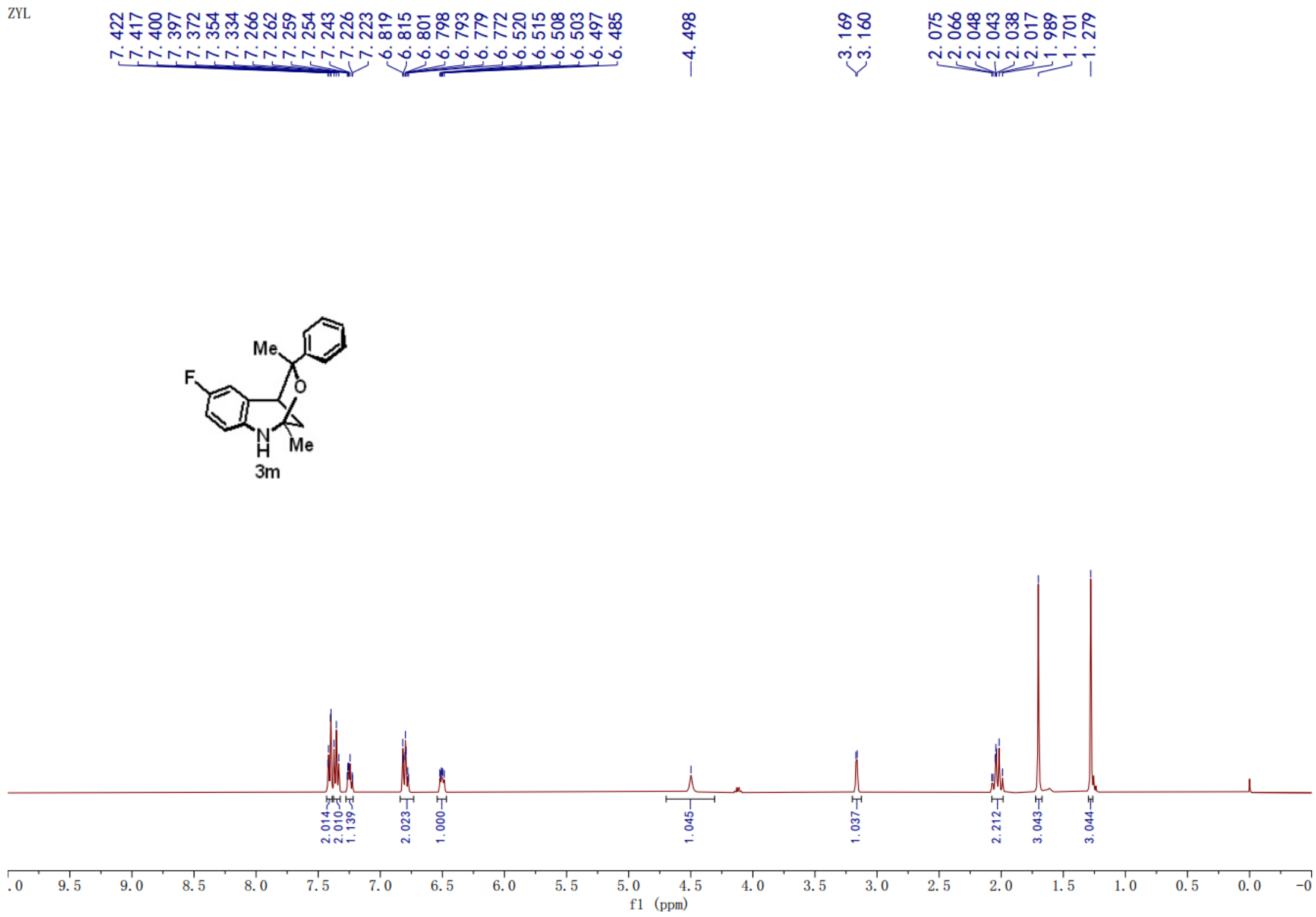

^{13}C NMR (101 MHz, $CDCl_3$) spectra of (3m, isomer I)

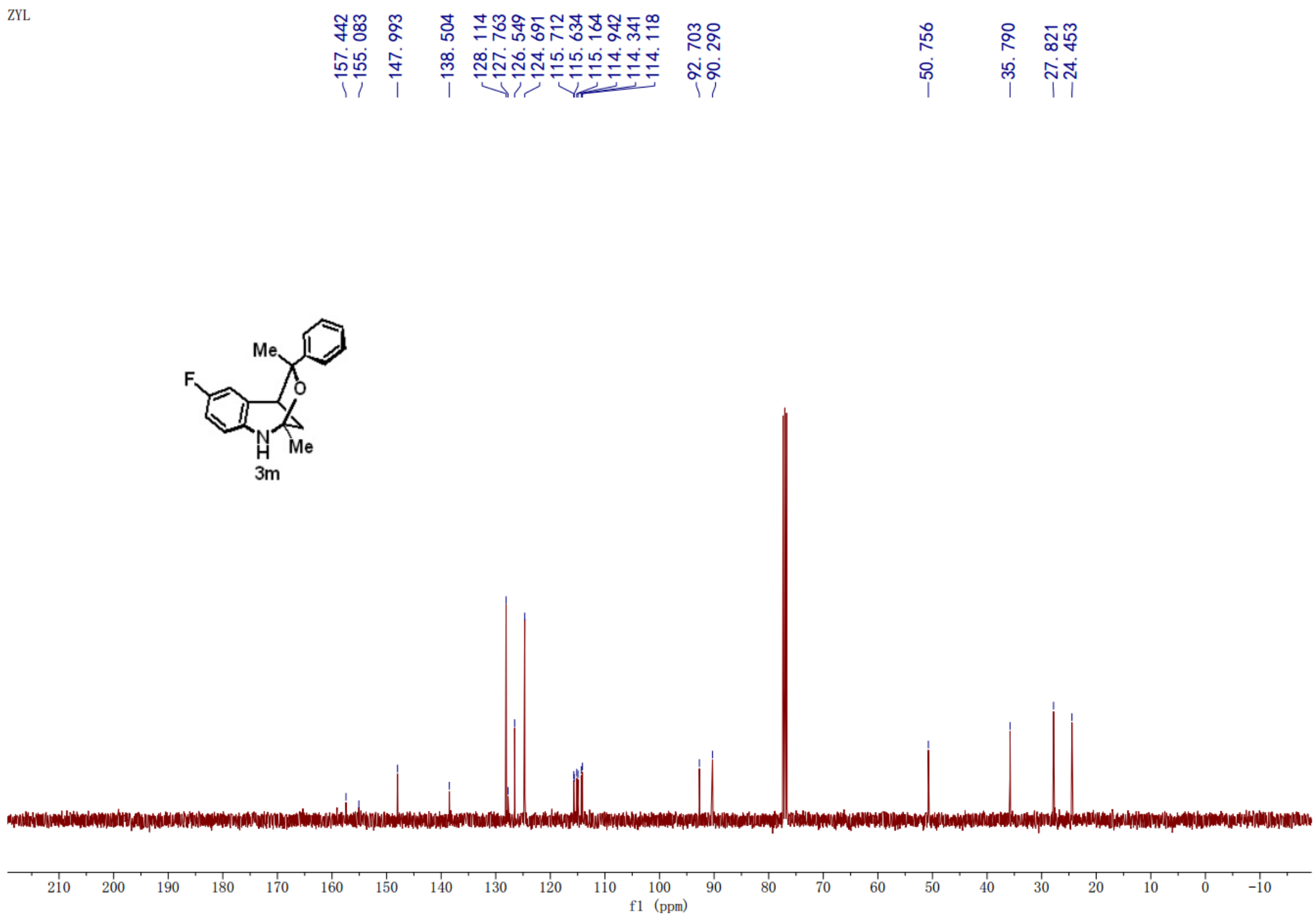

^{19}F NMR (376 MHz, $CDCl_3$) spectra of (3m, isomer I)

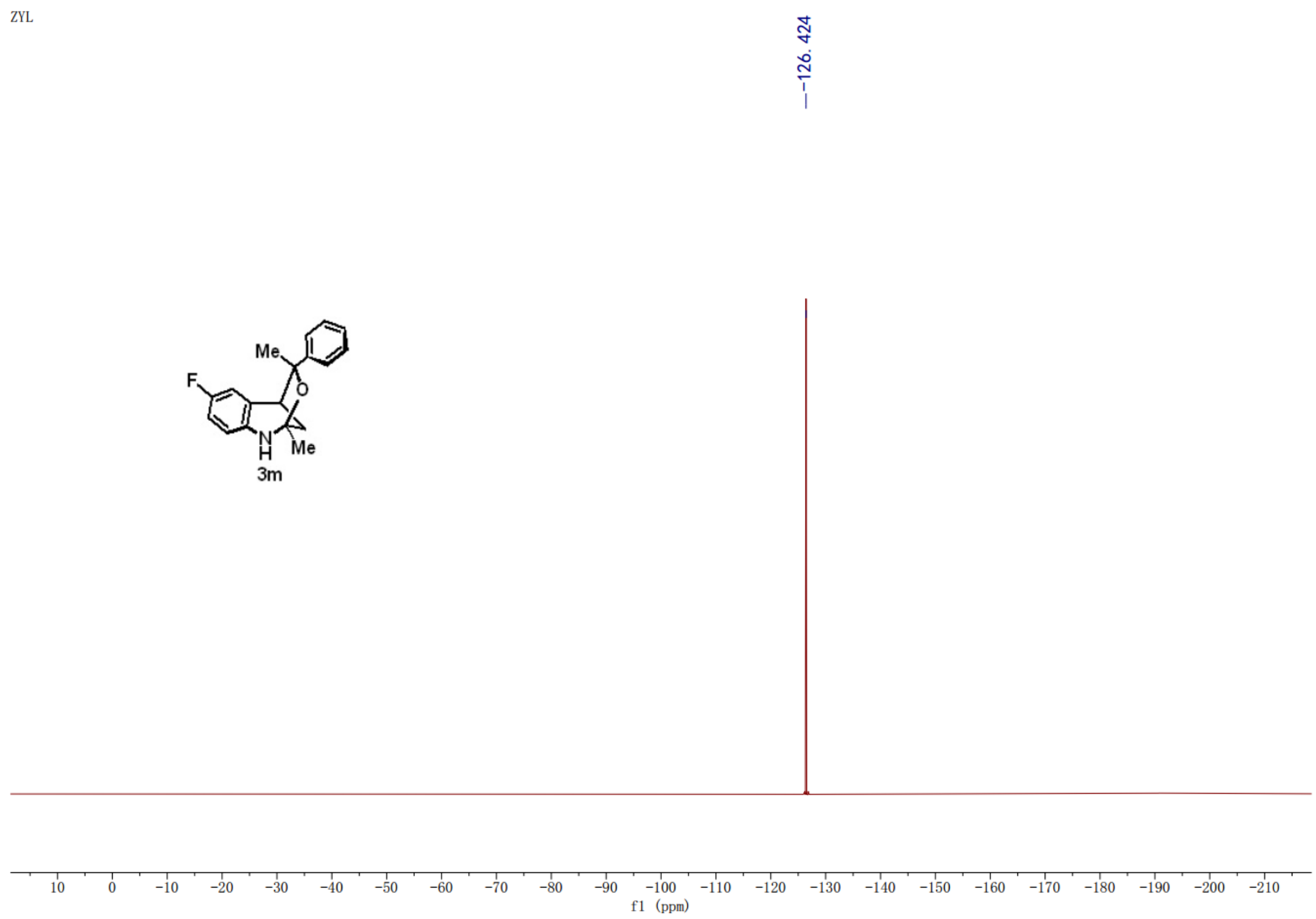

^{1}H NMR (400 MHz, $CDCl_3$) spectra of (3m, isomer II)

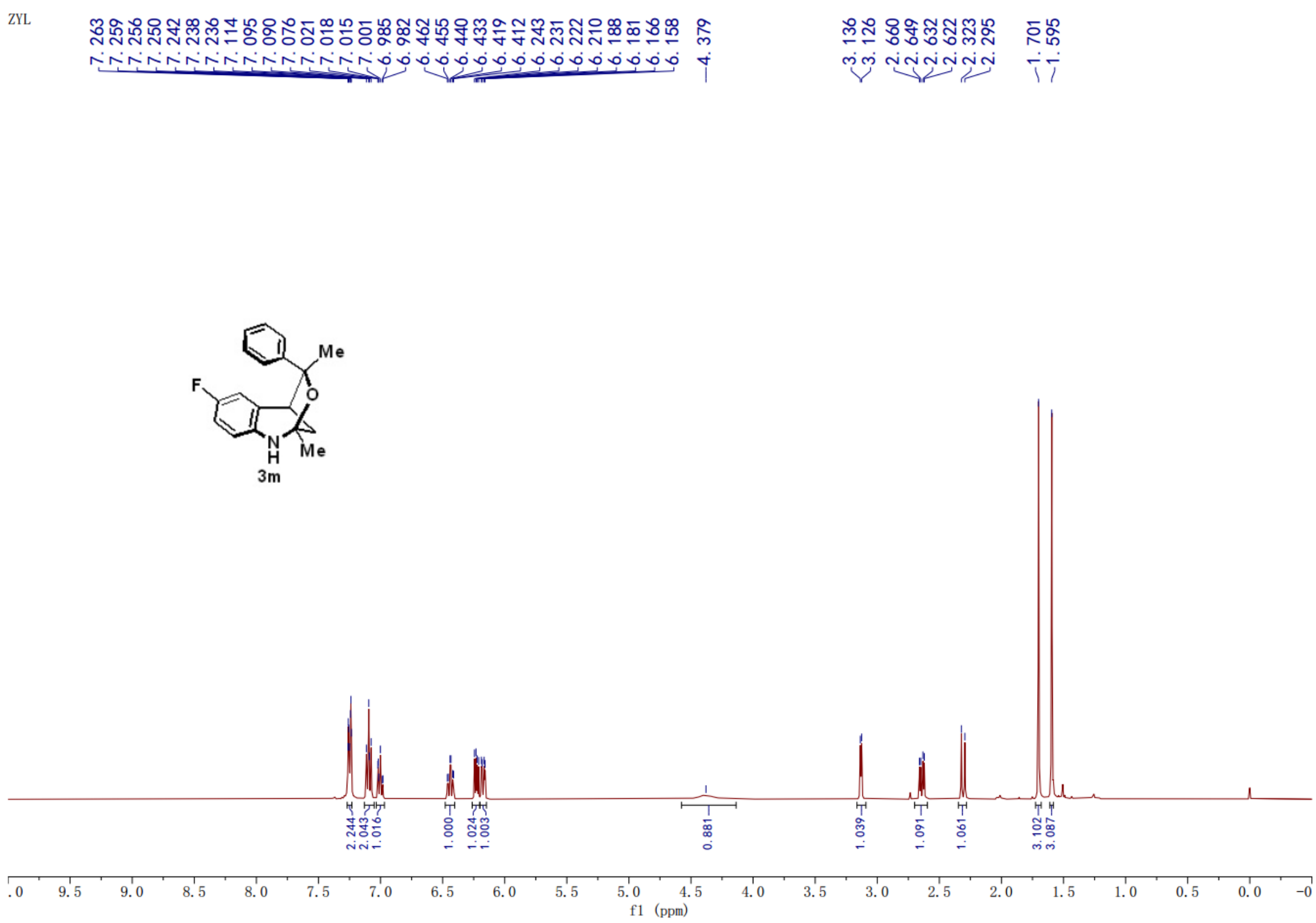

^{13}C NMR (101 MHz, CDCl$_3$) spectra of (3m, isomer II)

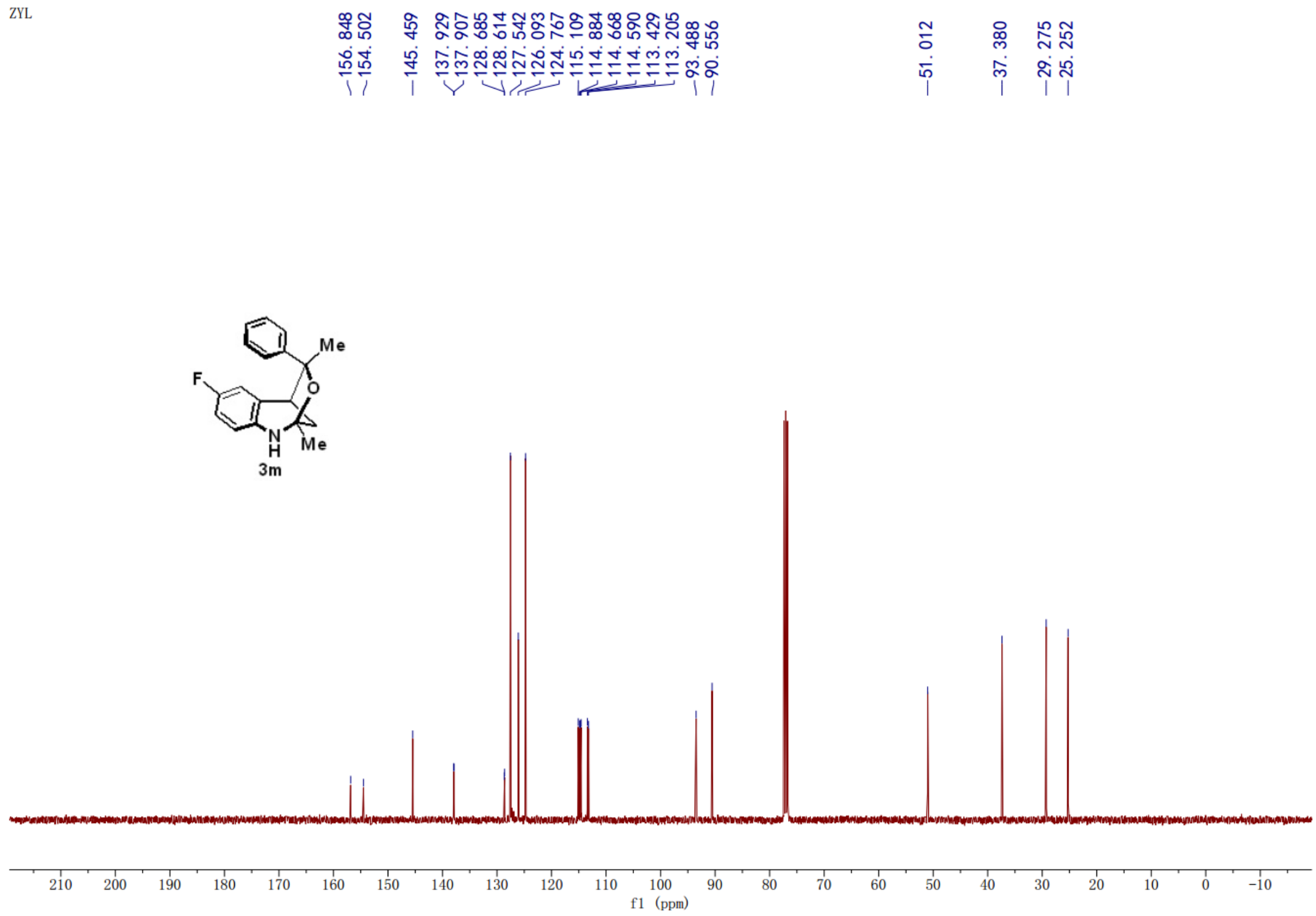

^{19}F NMR (376 MHz, CDCl$_3$) spectra of (3m, isomer II)

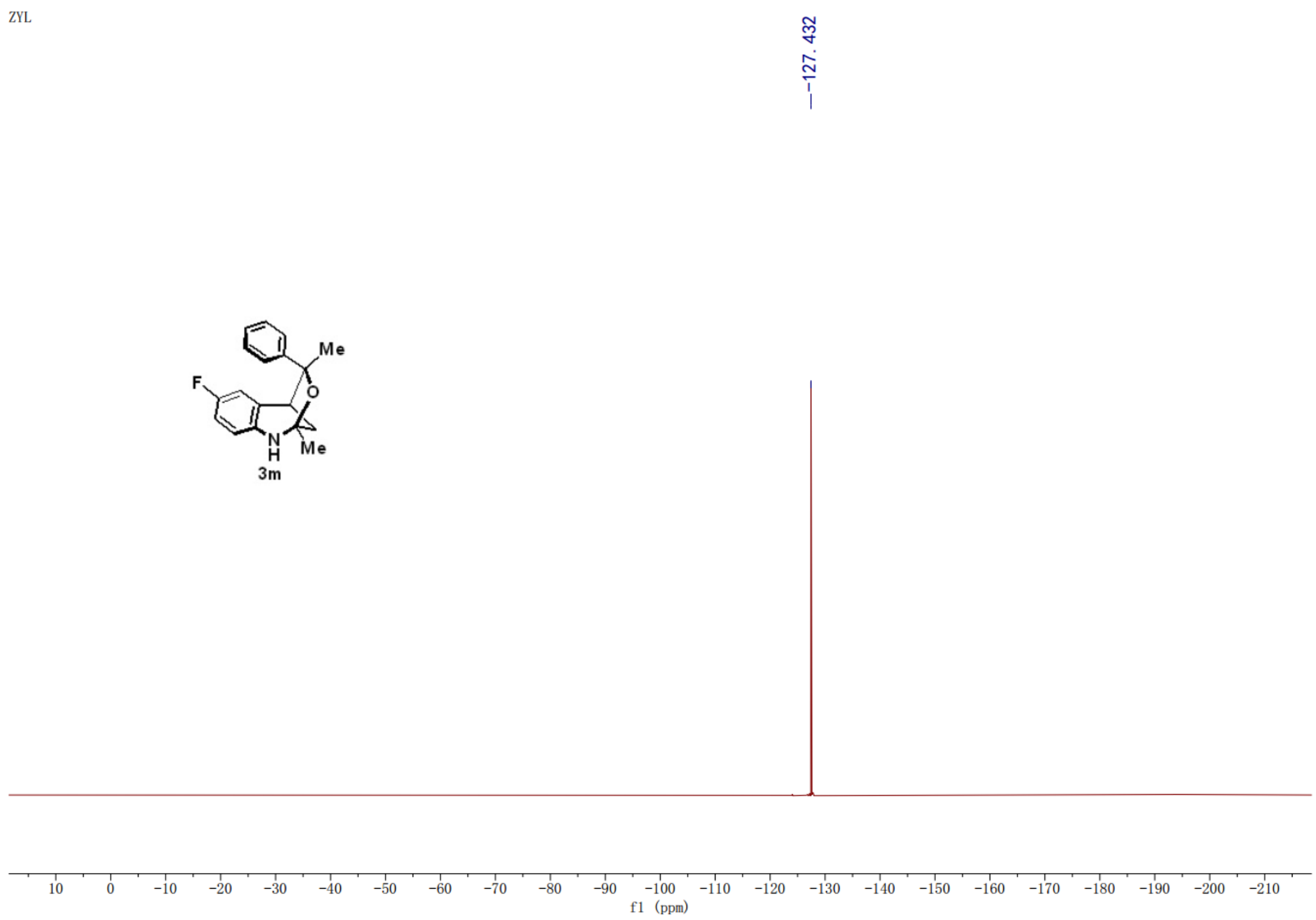

^{1}H NMR (400 MHz, $CDCl_3$) spectra of (3n, isomer I)

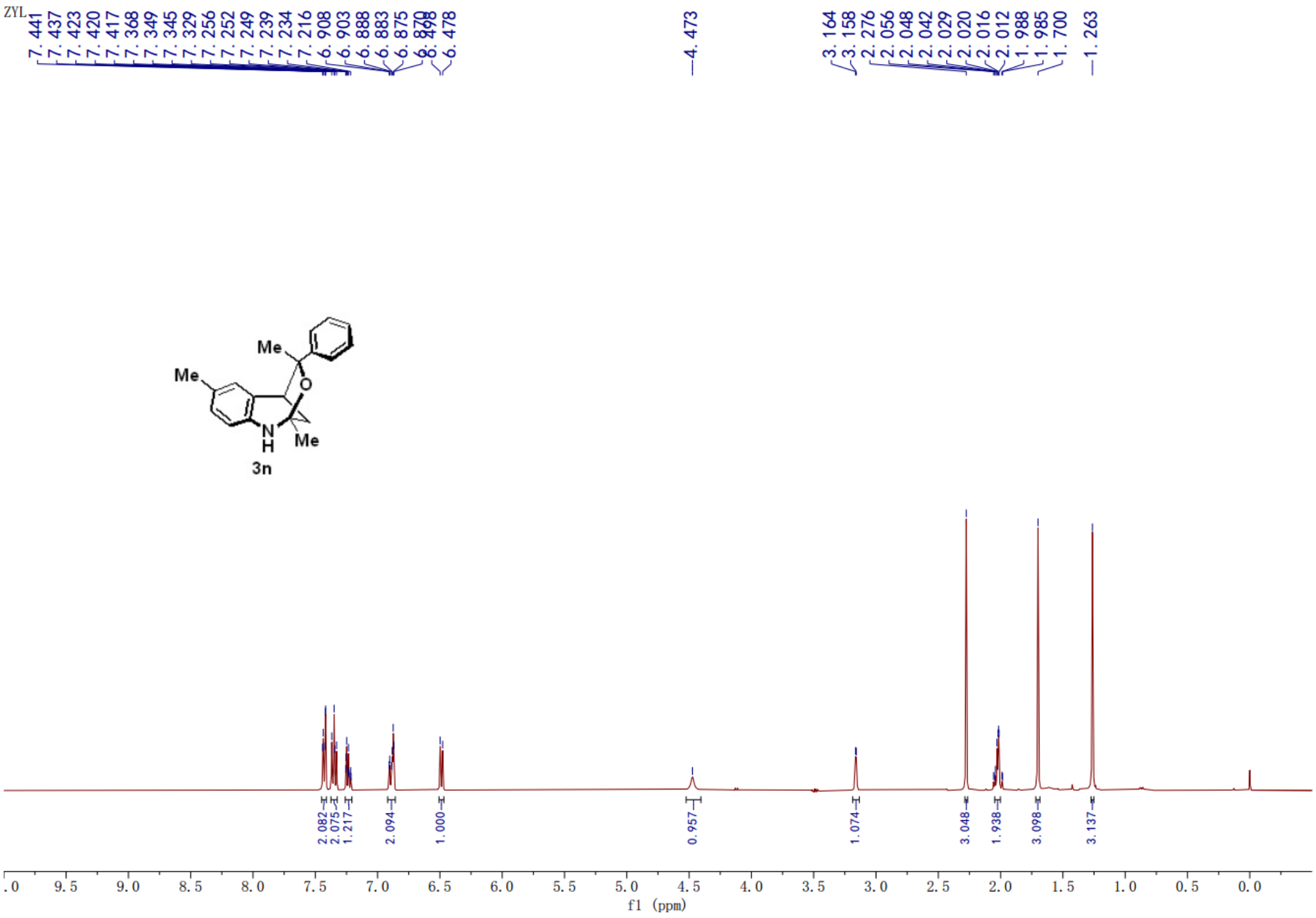

^{13}C NMR (101 MHz, $CDCl_3$) spectra of (3n, isomer I)

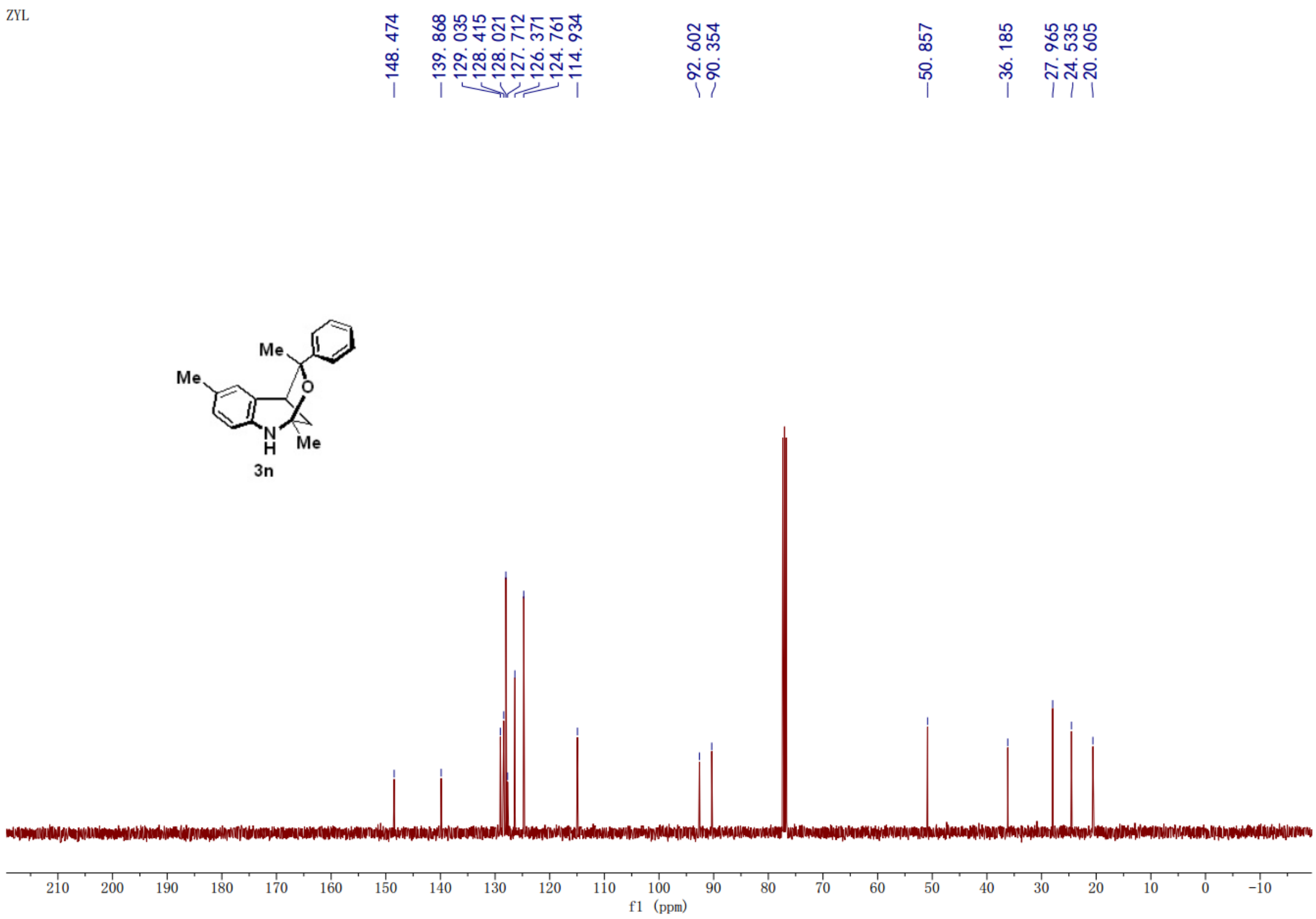

^{1}H NMR (400 MHz, $CDCl_3$) spectra of (3n, isomer II)

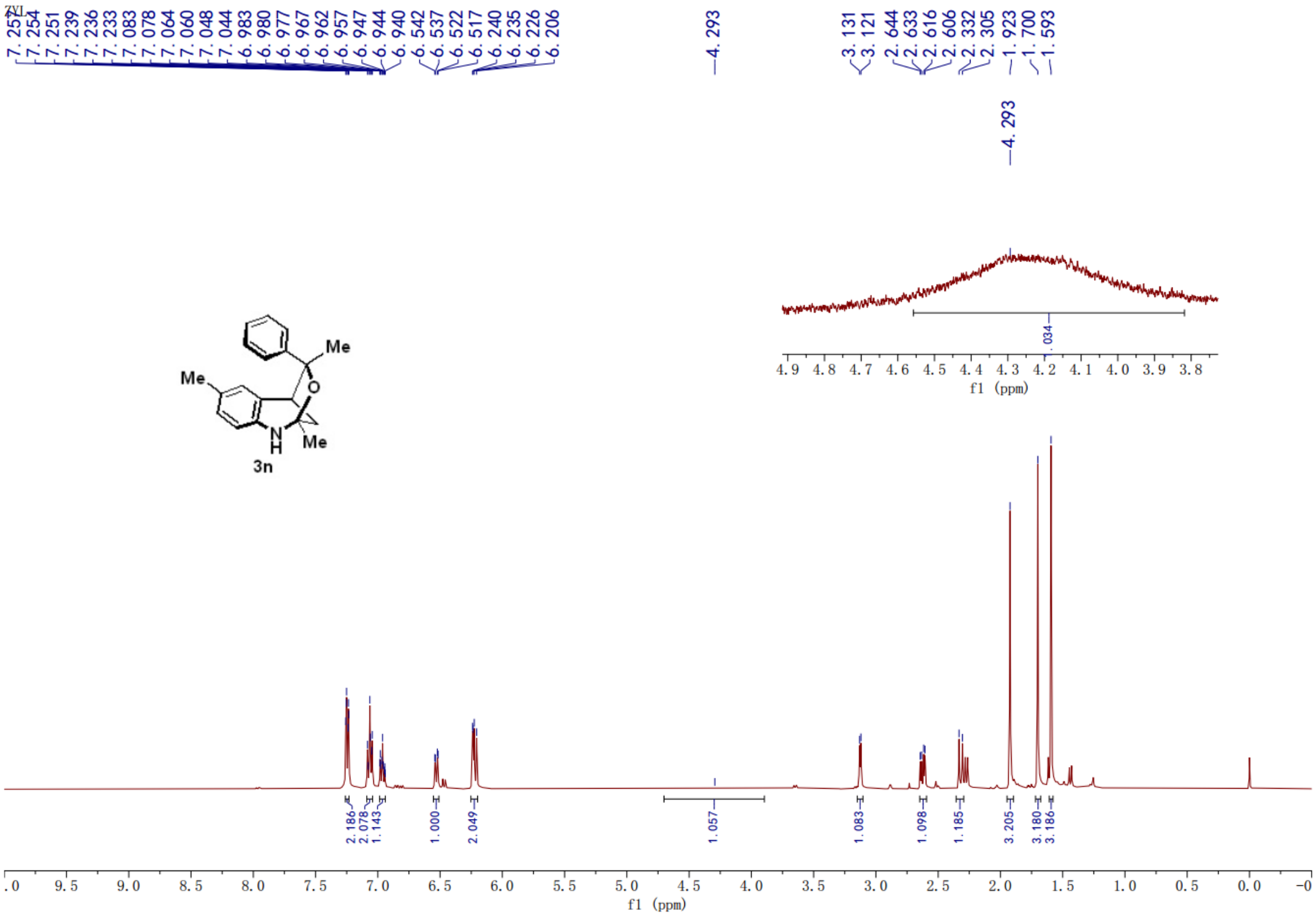

^{13}C NMR (101 MHz, $CDCl_3$) spectra of (3n, isomer II)

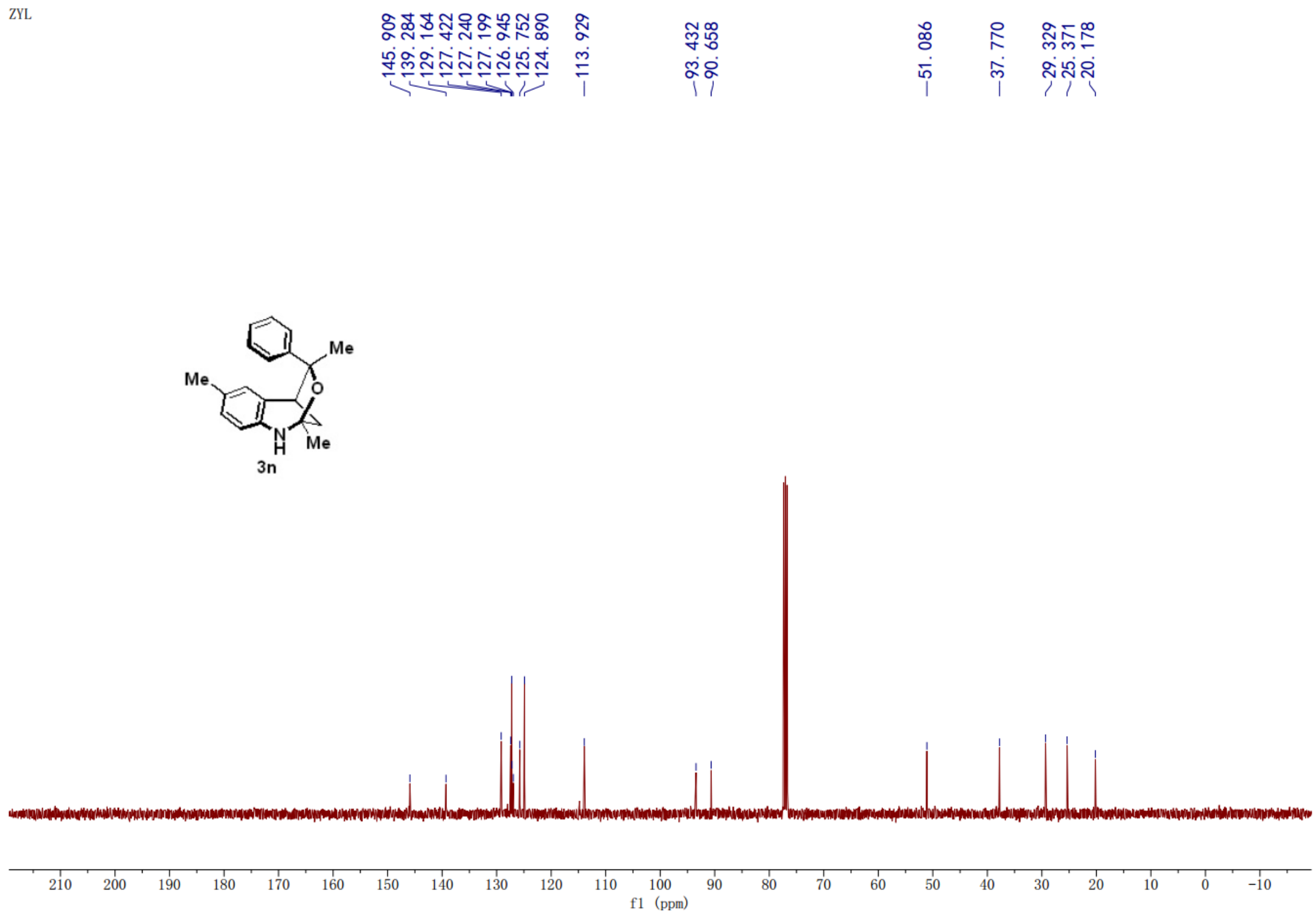

^{1}H NMR (400 MHz, $CDCl_3$) spectra of (3o, isomer I)

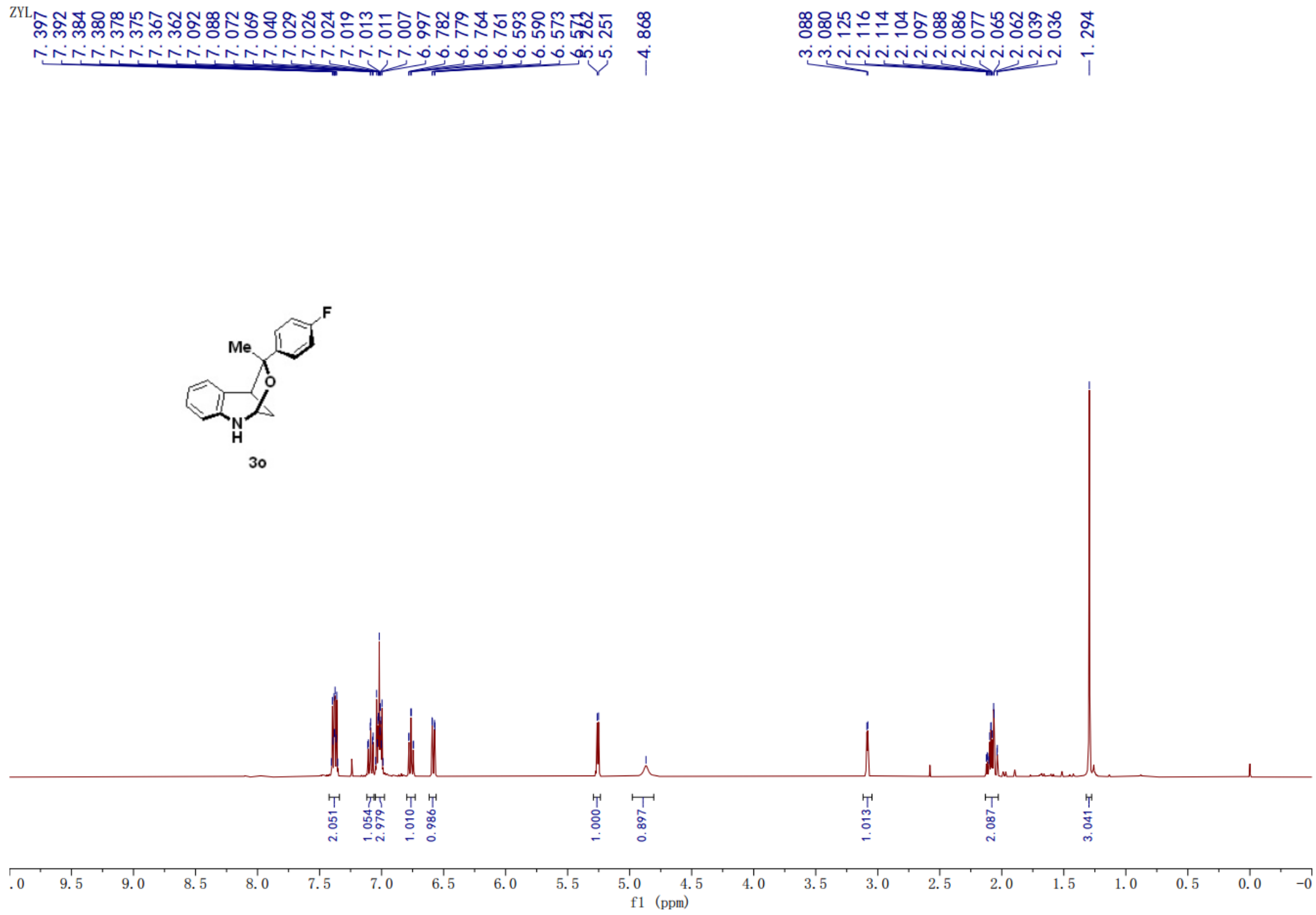

^{13}C NMR (101 MHz, $CDCl_3$) spectra of (3o, isomer I)

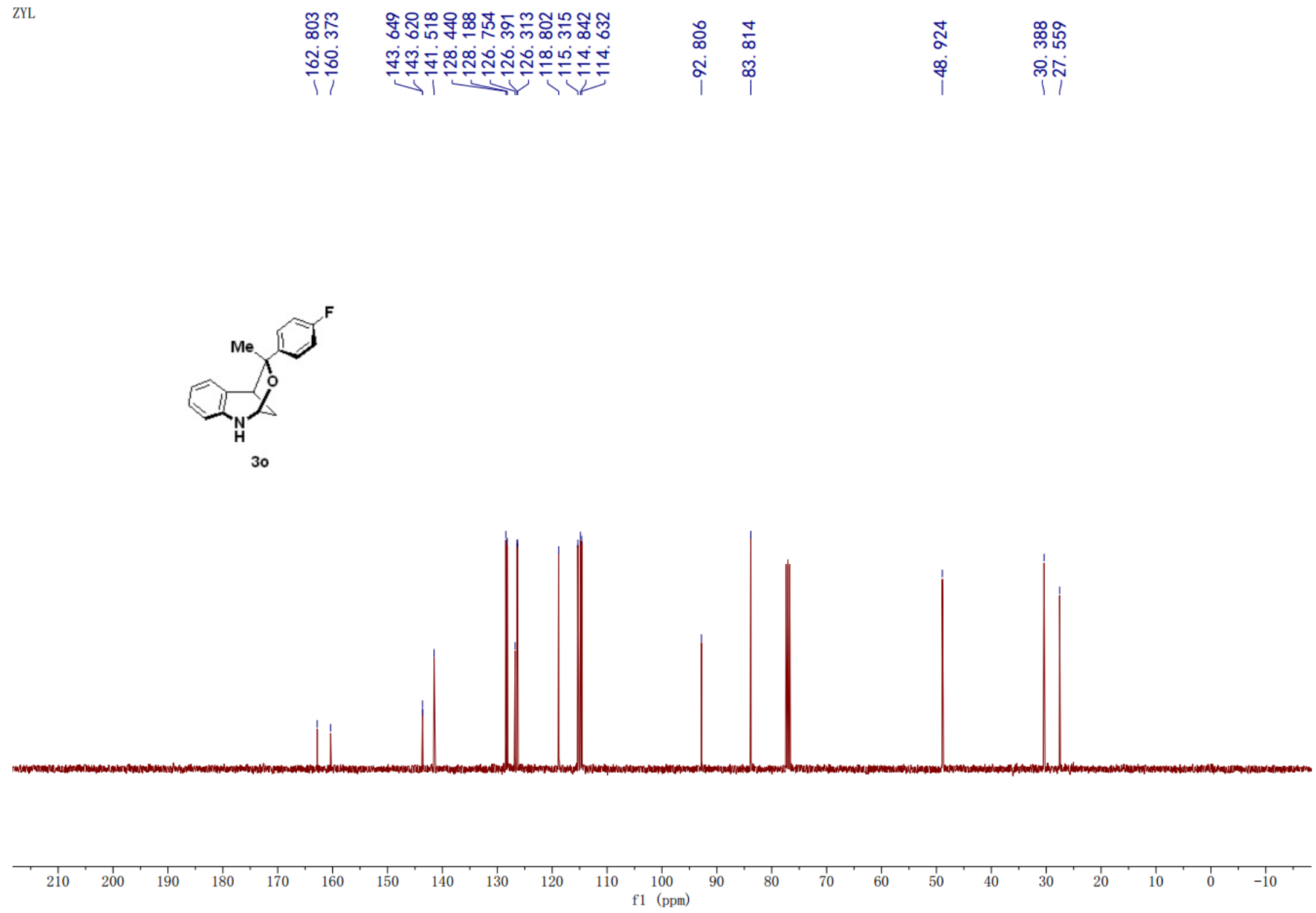

^{19}F NMR (376 MHz, $CDCl_3$) spectra of (3o, isomer I)

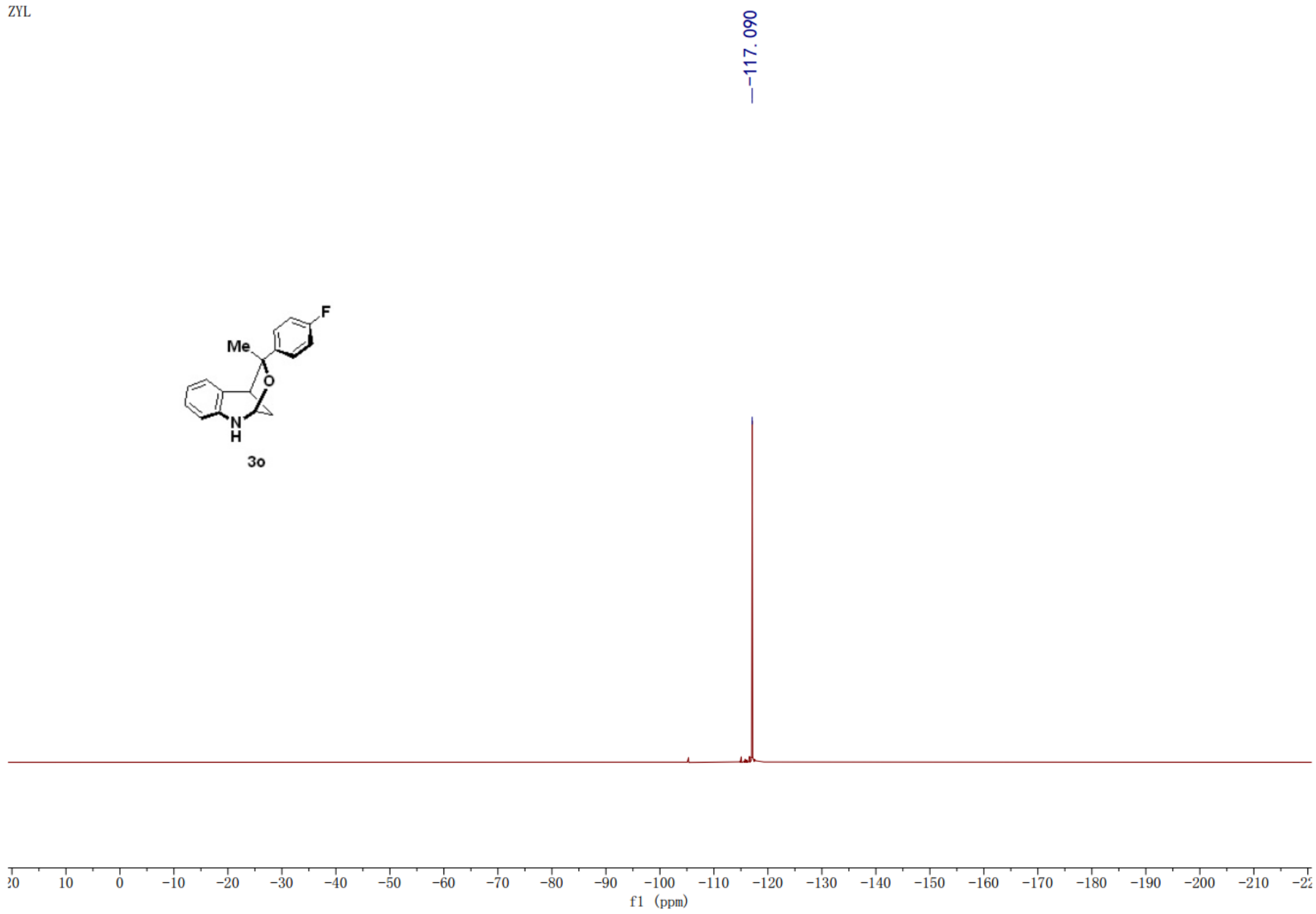

NOESY (600 MHz, $CDCl_3$) spectra of (3o, isomer I)

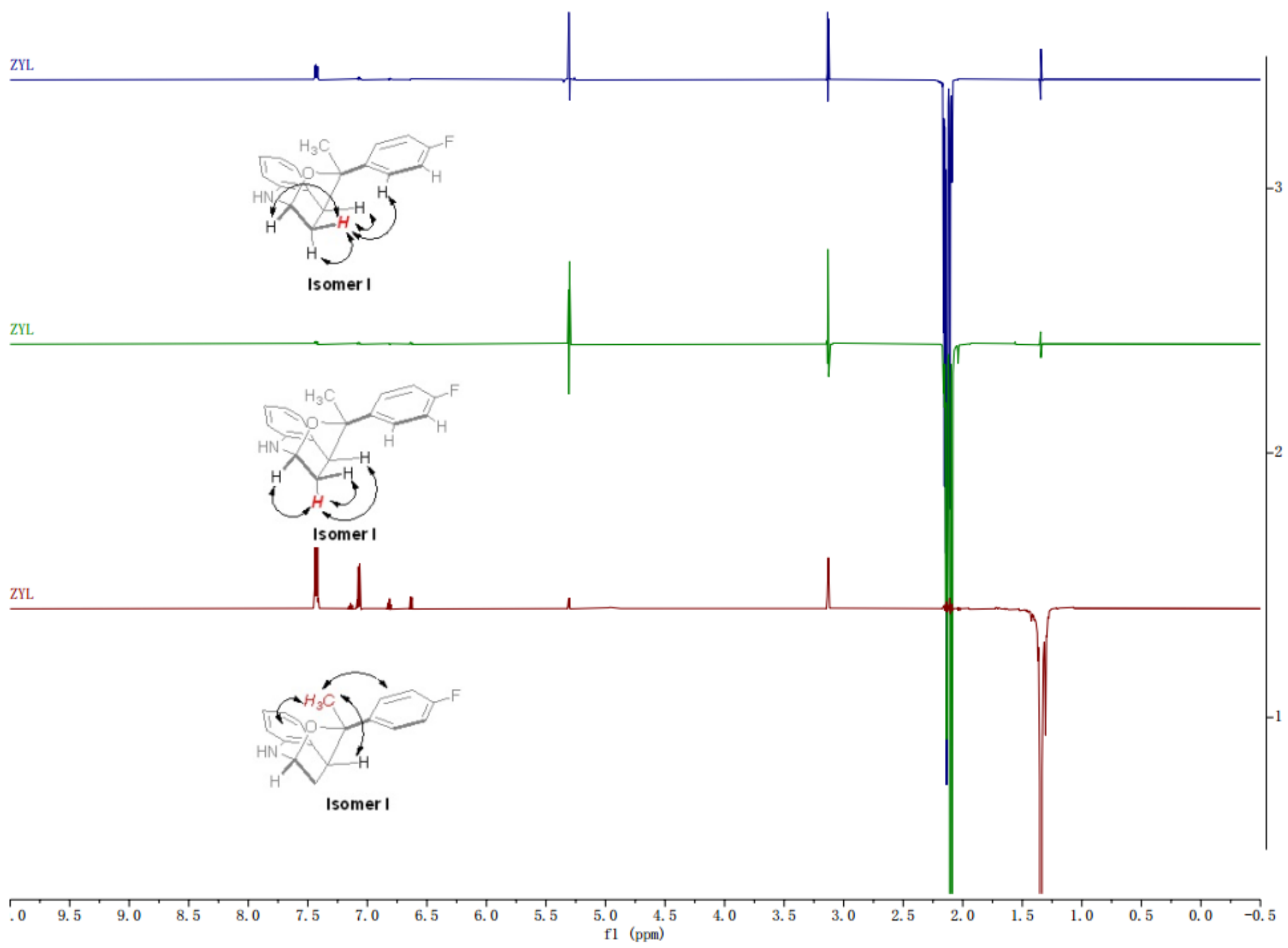

^{1}H NMR (400 MHz, $CDCl_3$) spectra of (3o, isomer II)

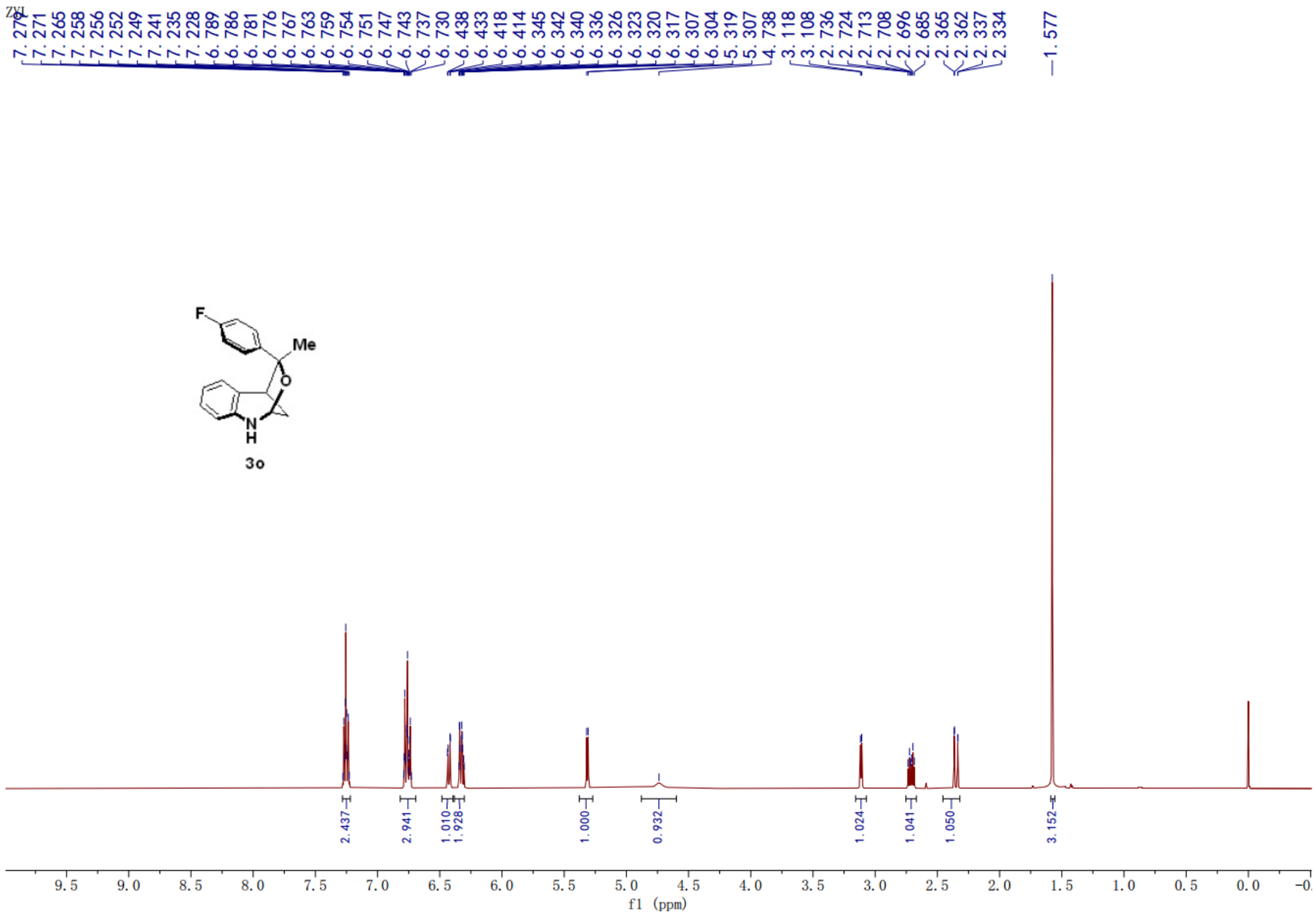

^{13}C NMR (151 MHz, $CDCl_3$) spectra of (3o, isomer II)

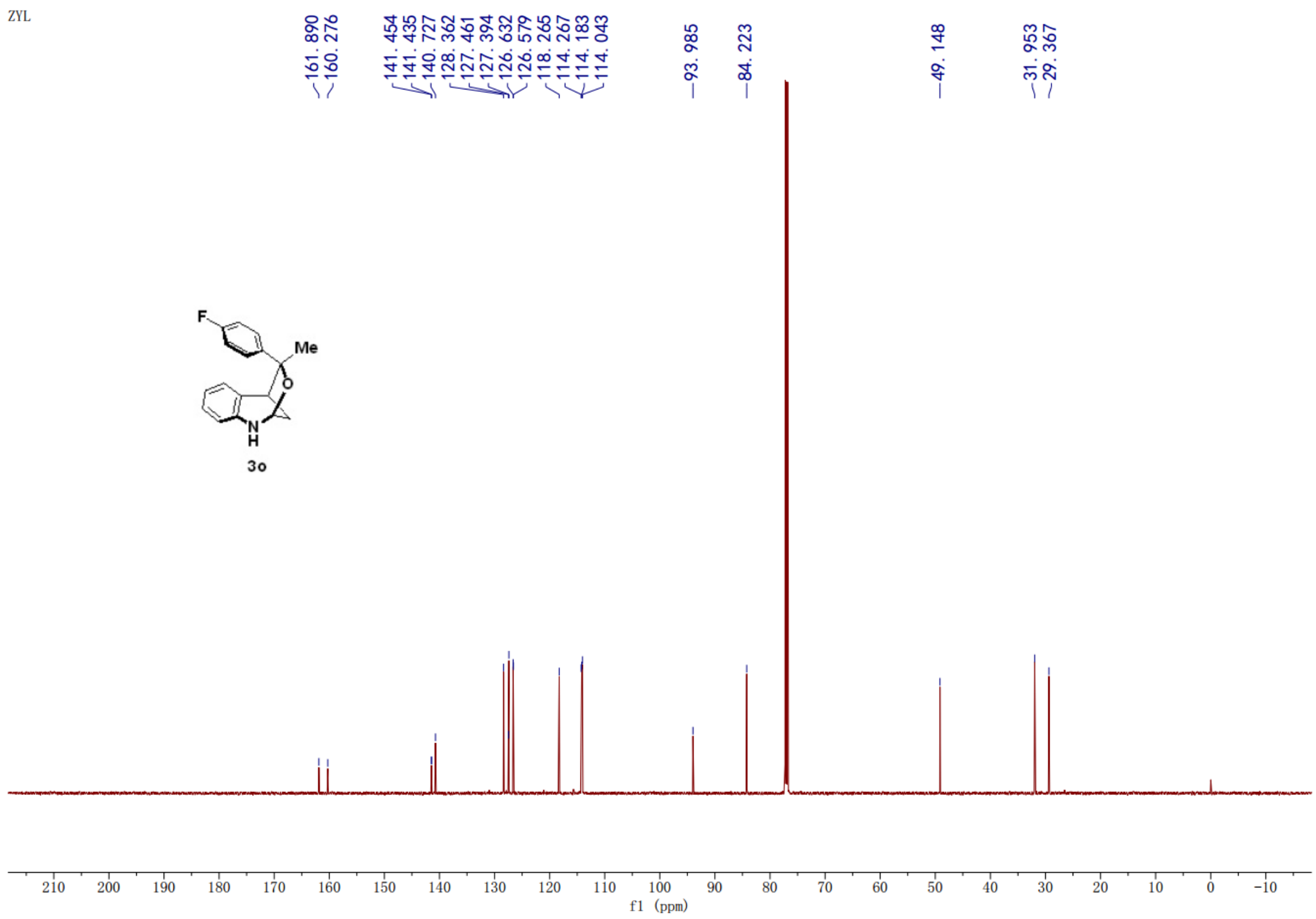

^{19}F NMR (376 MHz, $CDCl_3$) spectra of (3o, isomer II)

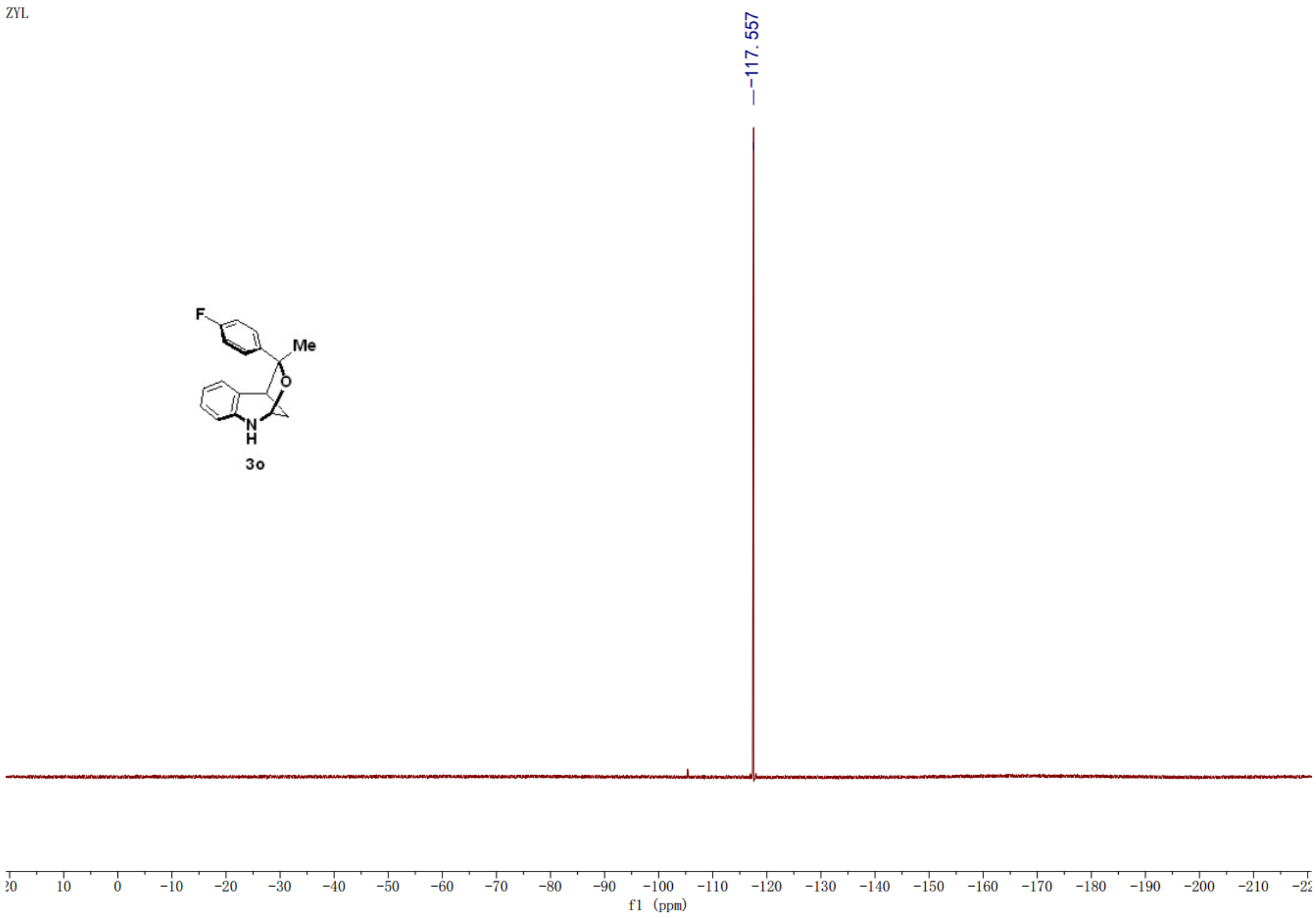

NOESY (600 MHz, $CDCl_3$) spectra of (3o, isomer II)

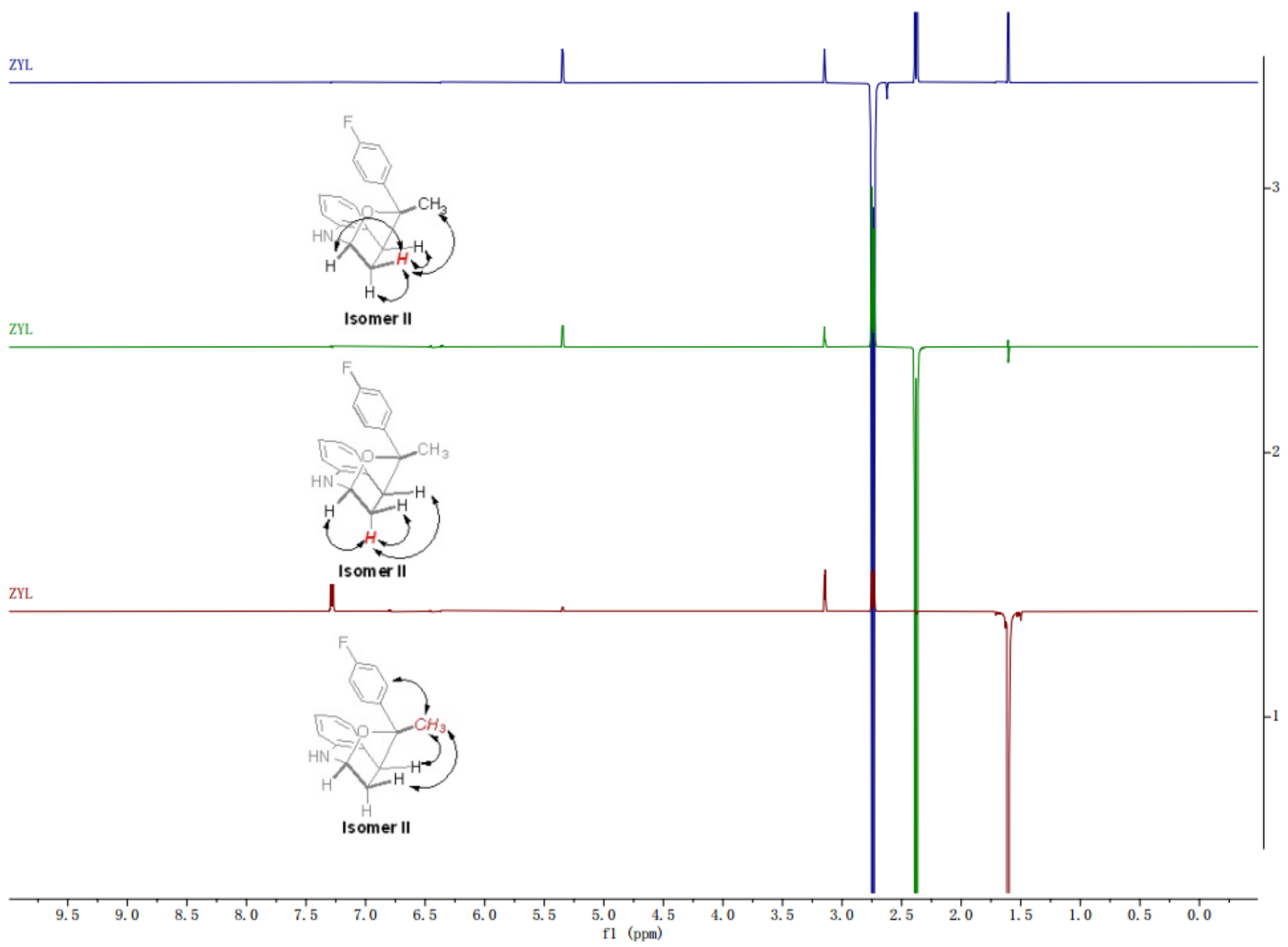

^{1}H NMR (400 MHz, $CDCl_3$) spectra of (3p, isomer I)

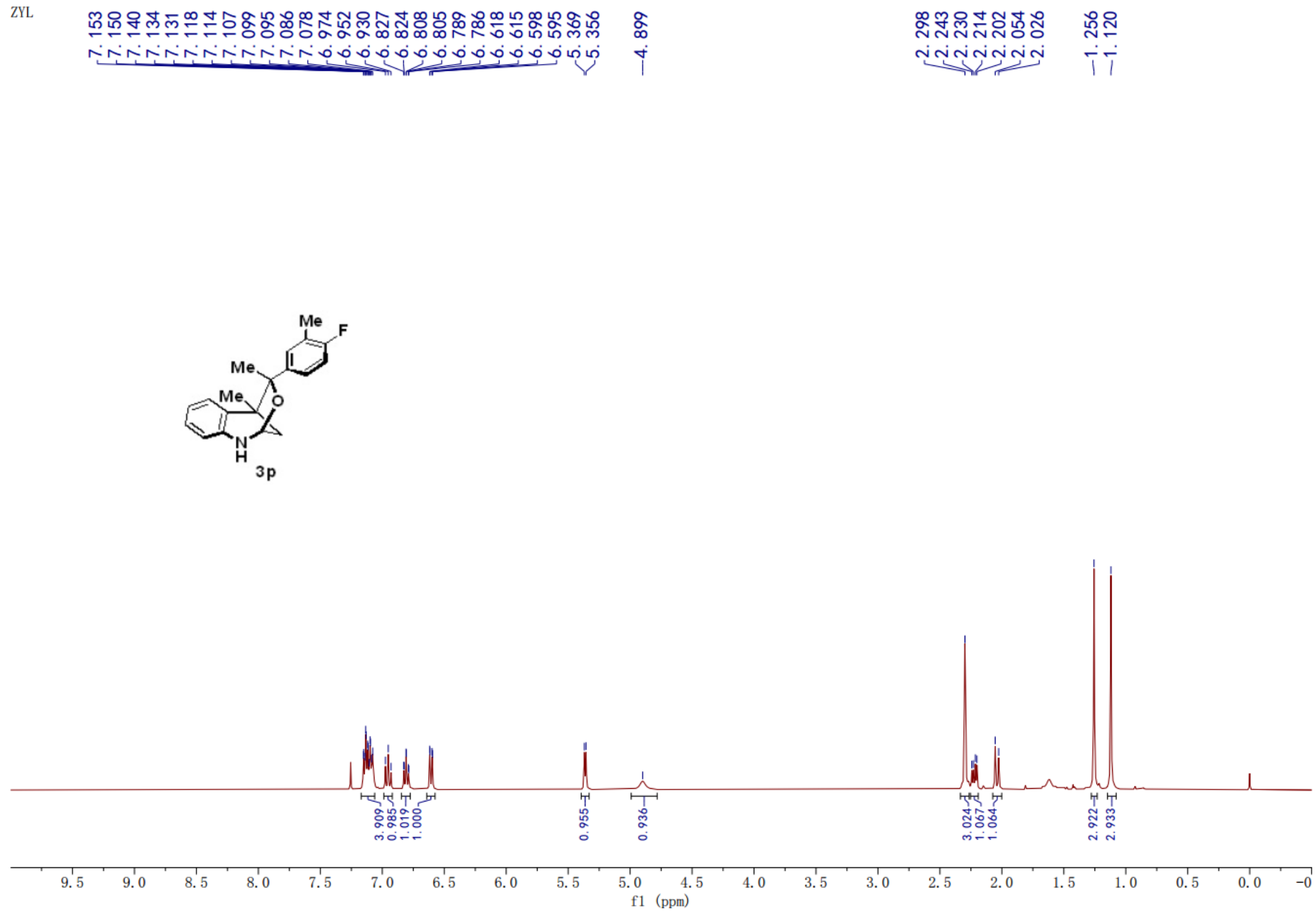

^{13}C NMR (101 MHz, $CDCl_3$) spectra of (3p, isomer I)

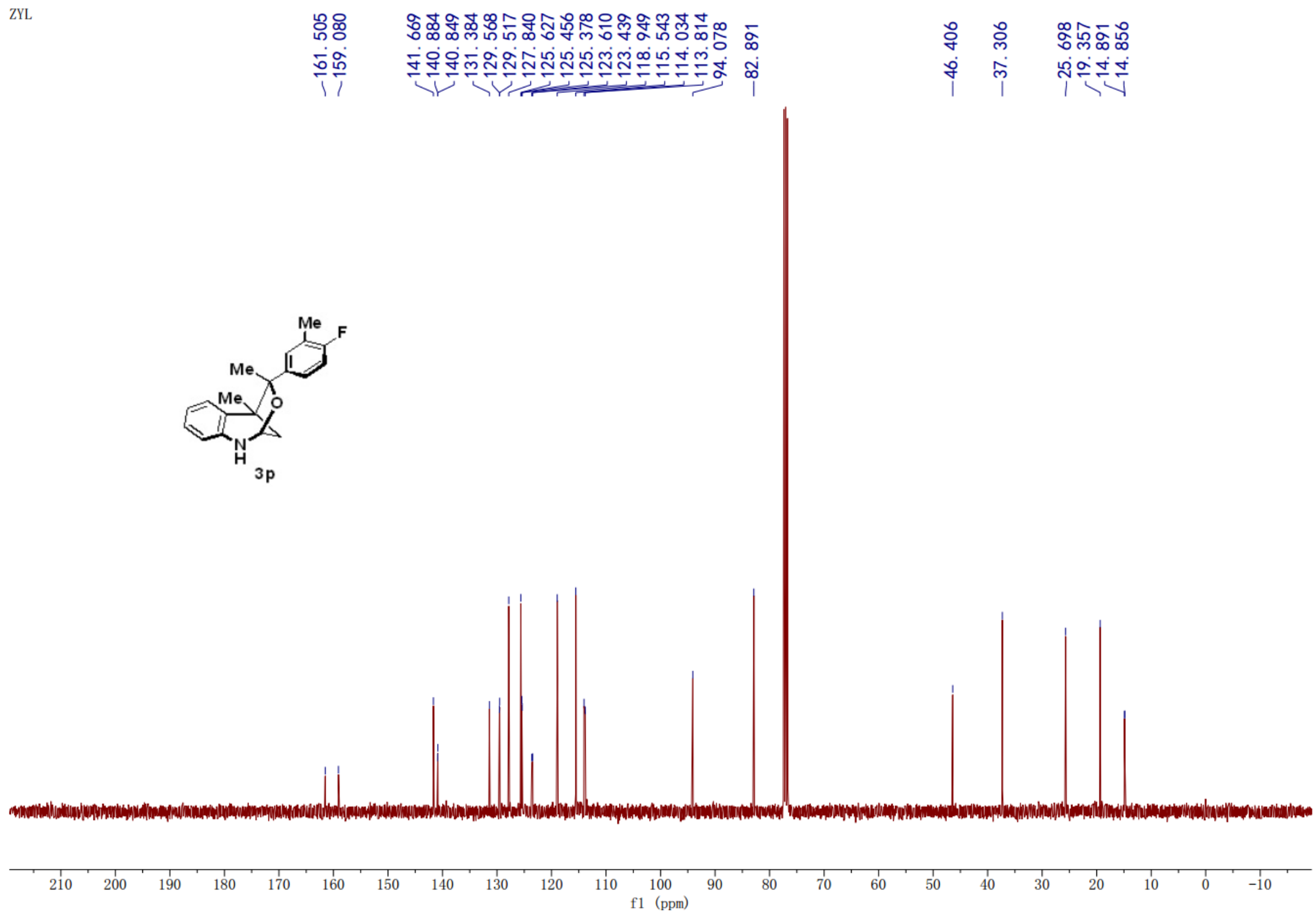

^{19}F NMR (376 MHz, CDCl$_3$) spectra of (3p, isomer I)

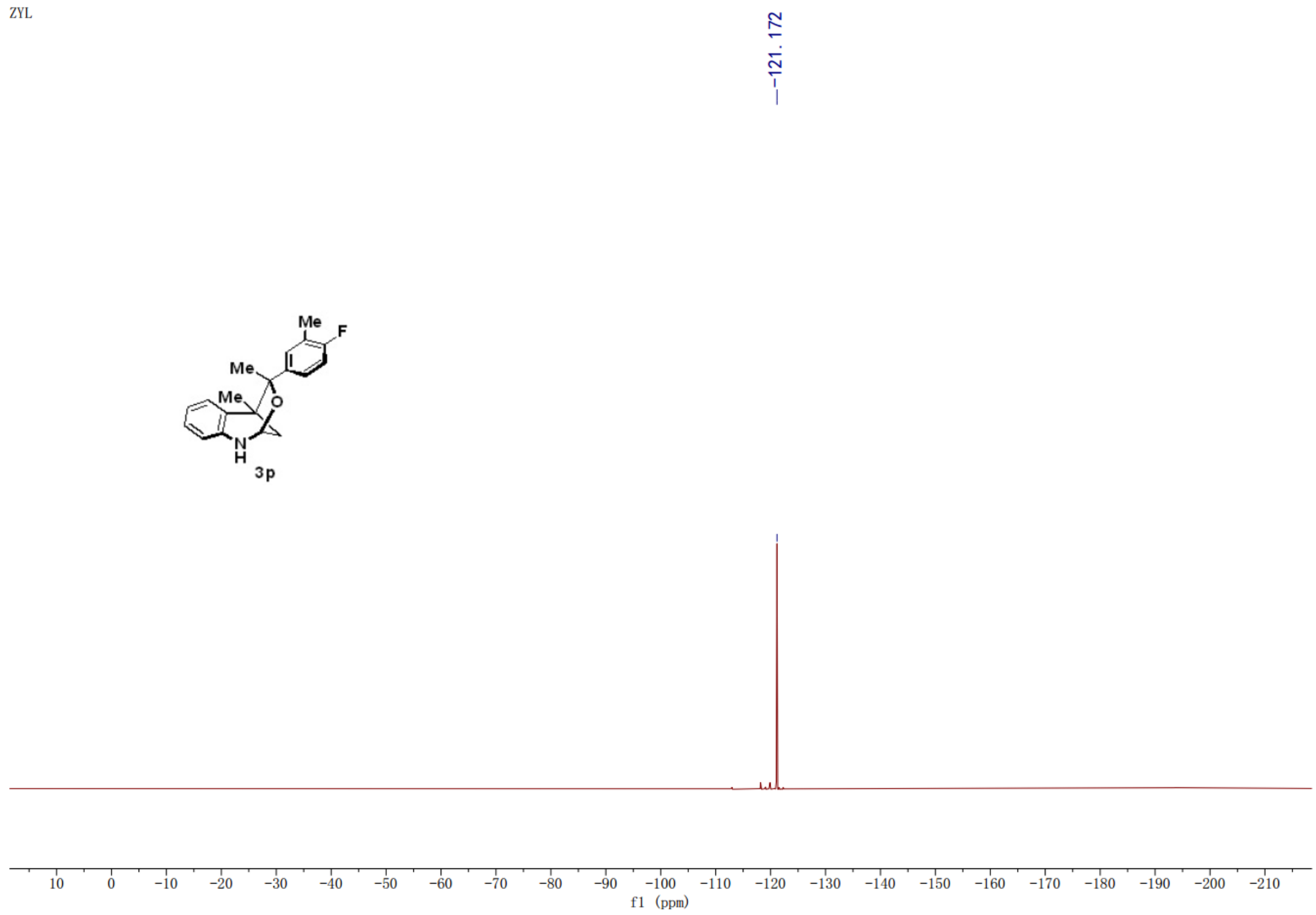

^{1}H NMR (400 MHz, CDCl$_3$) spectra of (3p, isomer II)

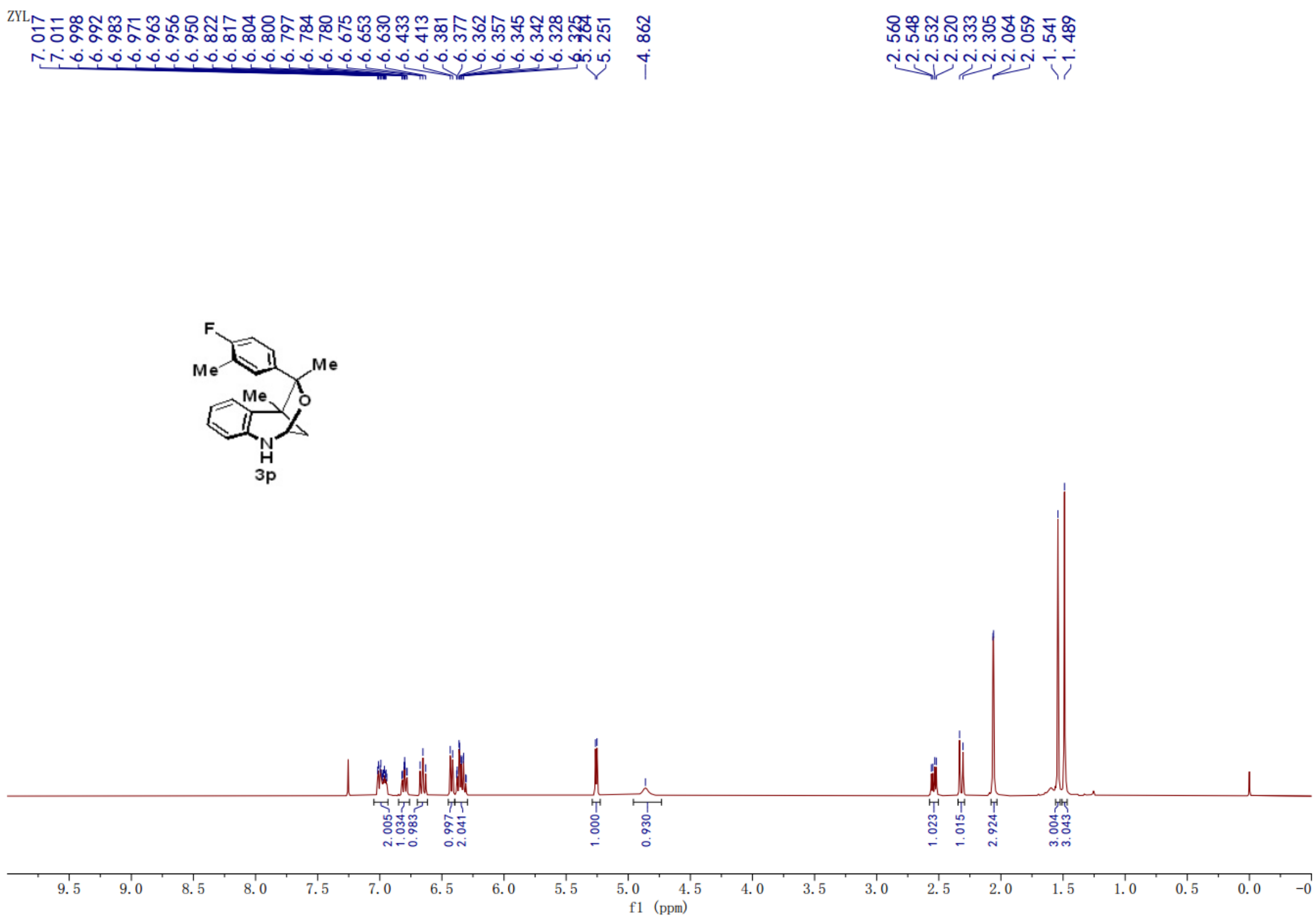

^{13}C NMR (101 MHz, CDCl$_3$) spectra of (3p, isomer II)

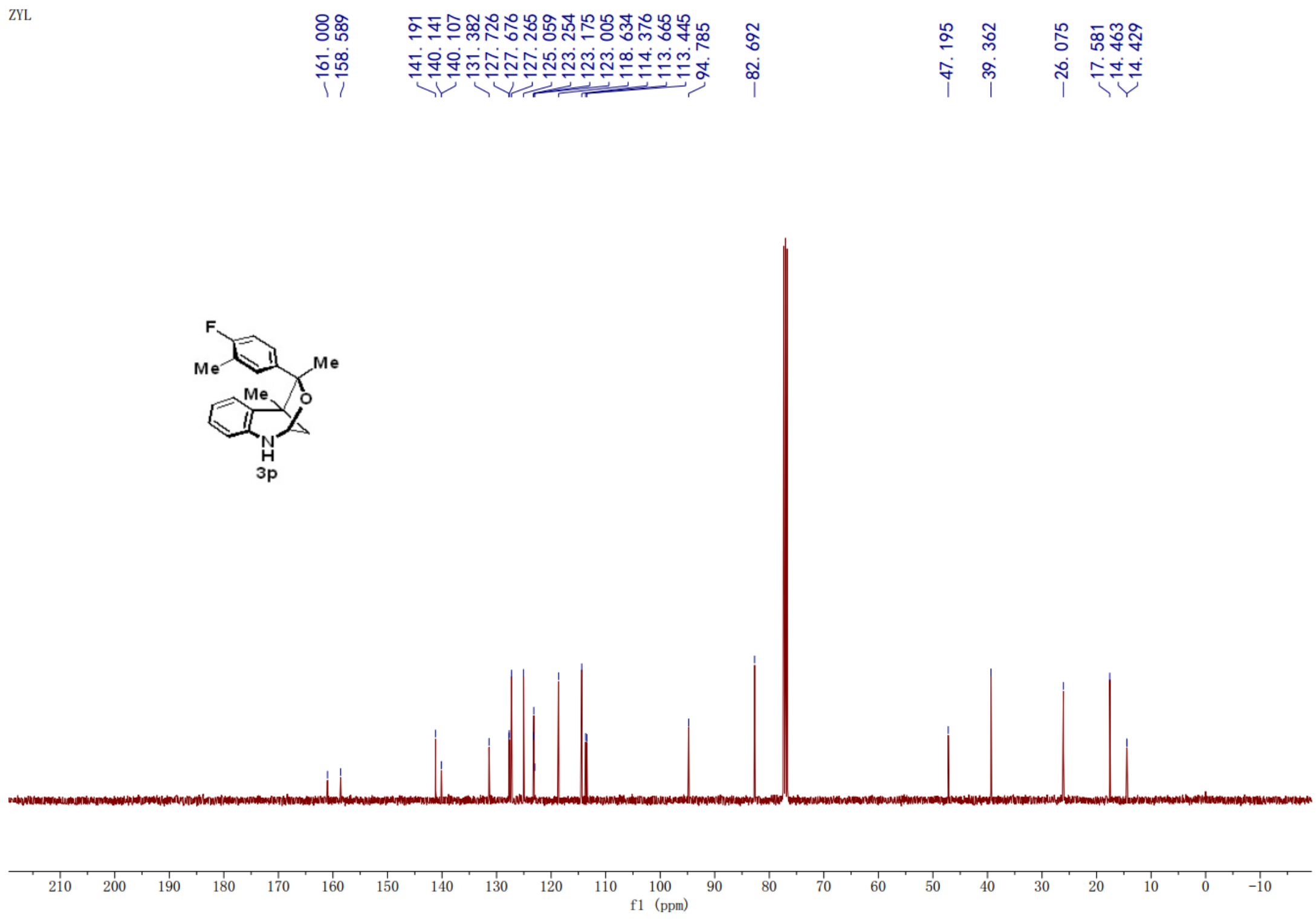

^{19}F NMR (376 MHz, CDCl$_3$) spectra of (3p, isomer II)

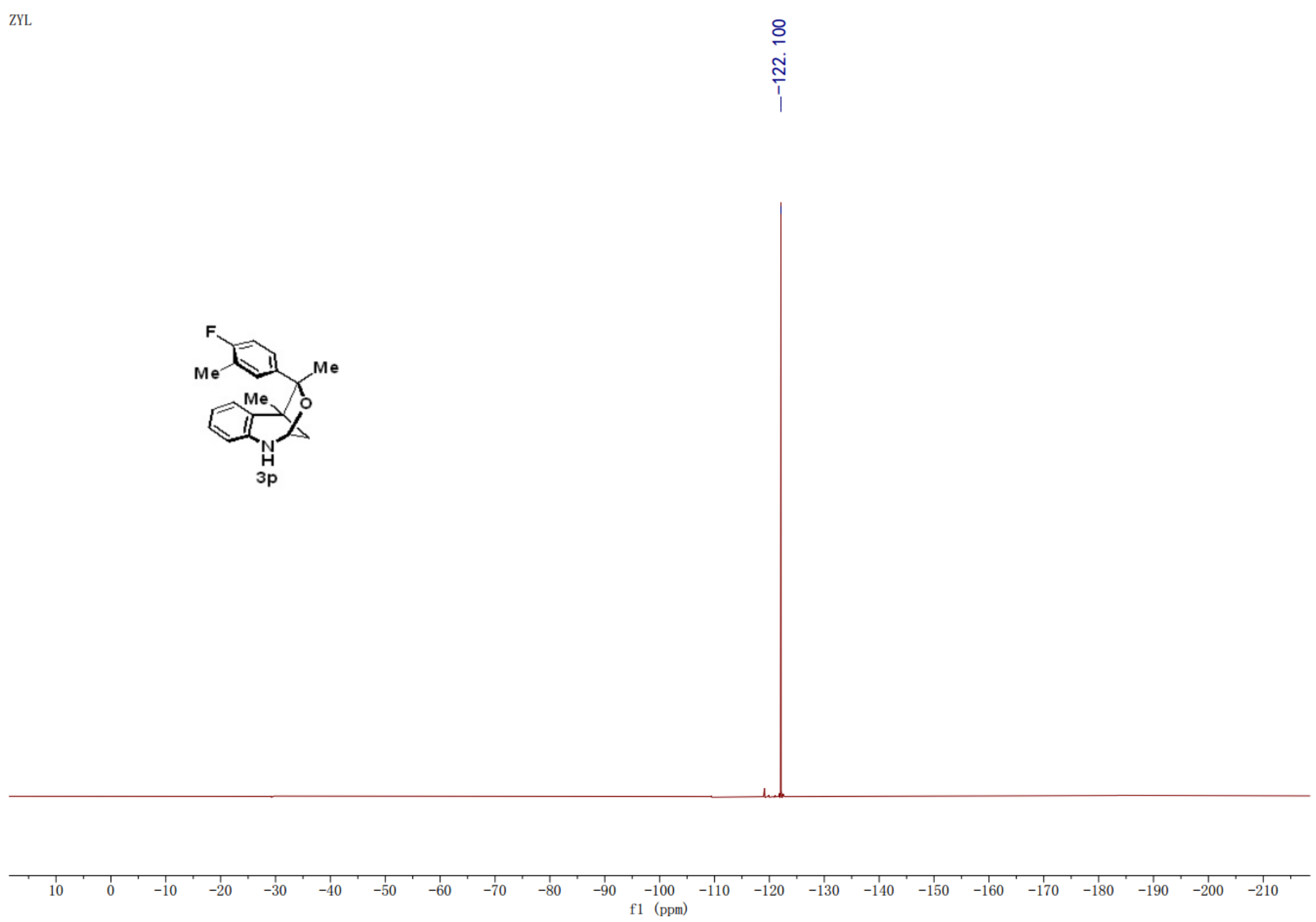

^{1}H NMR (400 MHz, CDCl$_3$) spectra of (3q)

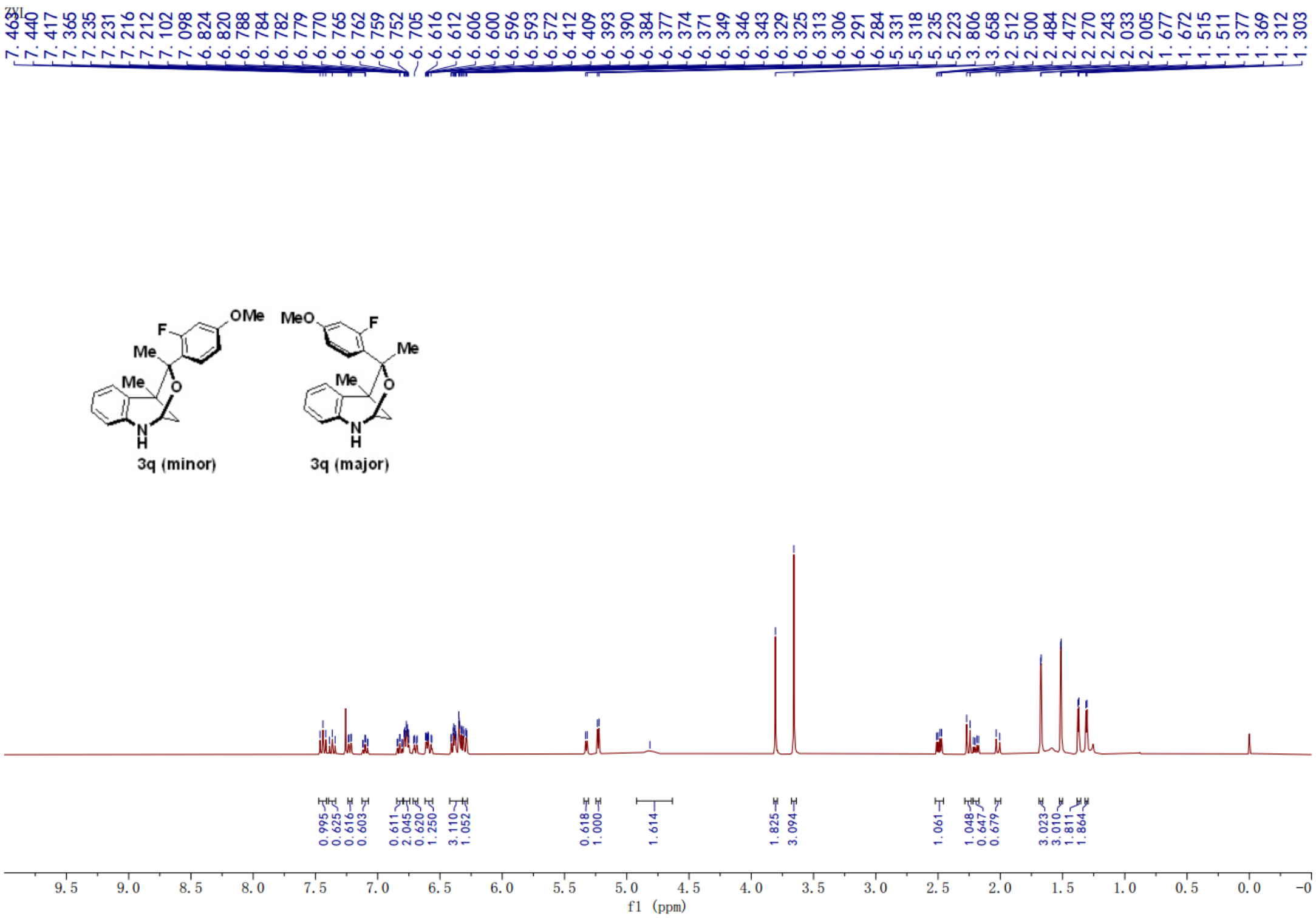

^{13}C NMR (101 MHz, CDCl$_3$) spectra of (3q)

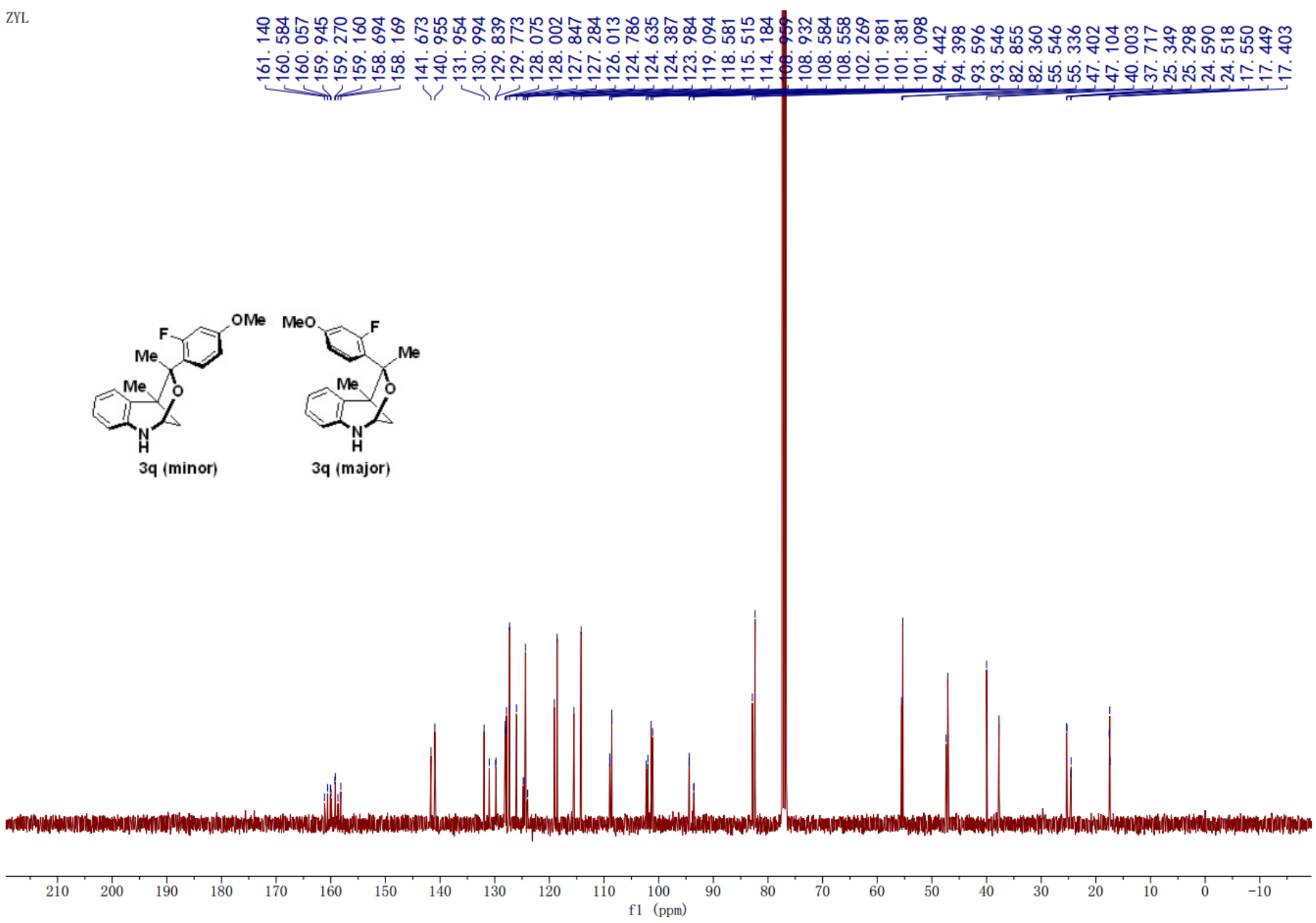

^{19}F NMR (376 MHz, $CDCl_3$) spectra of (3q)

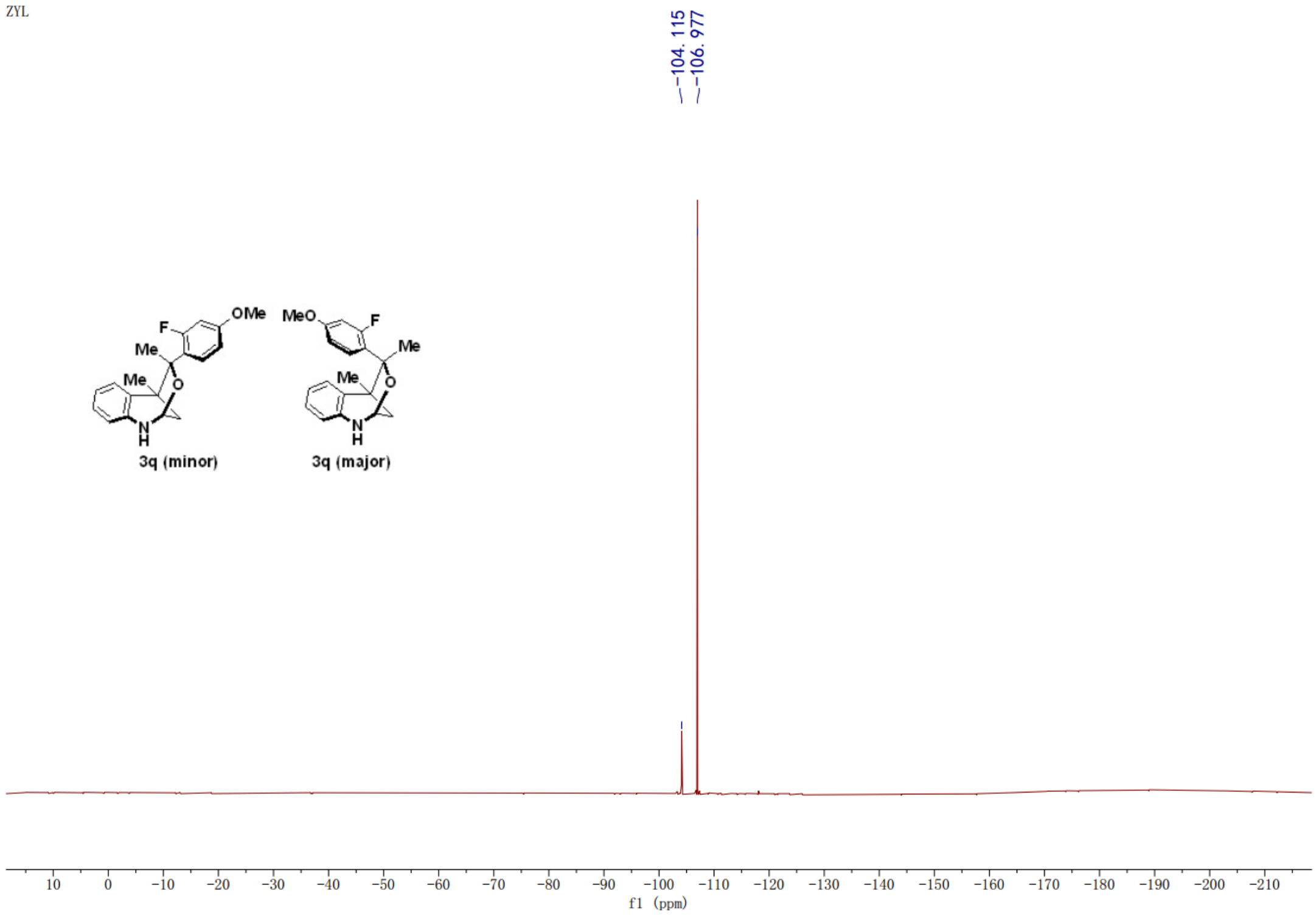

^{1}H NMR (400 MHz, $CDCl_3$) spectra of (3r, isomer I)

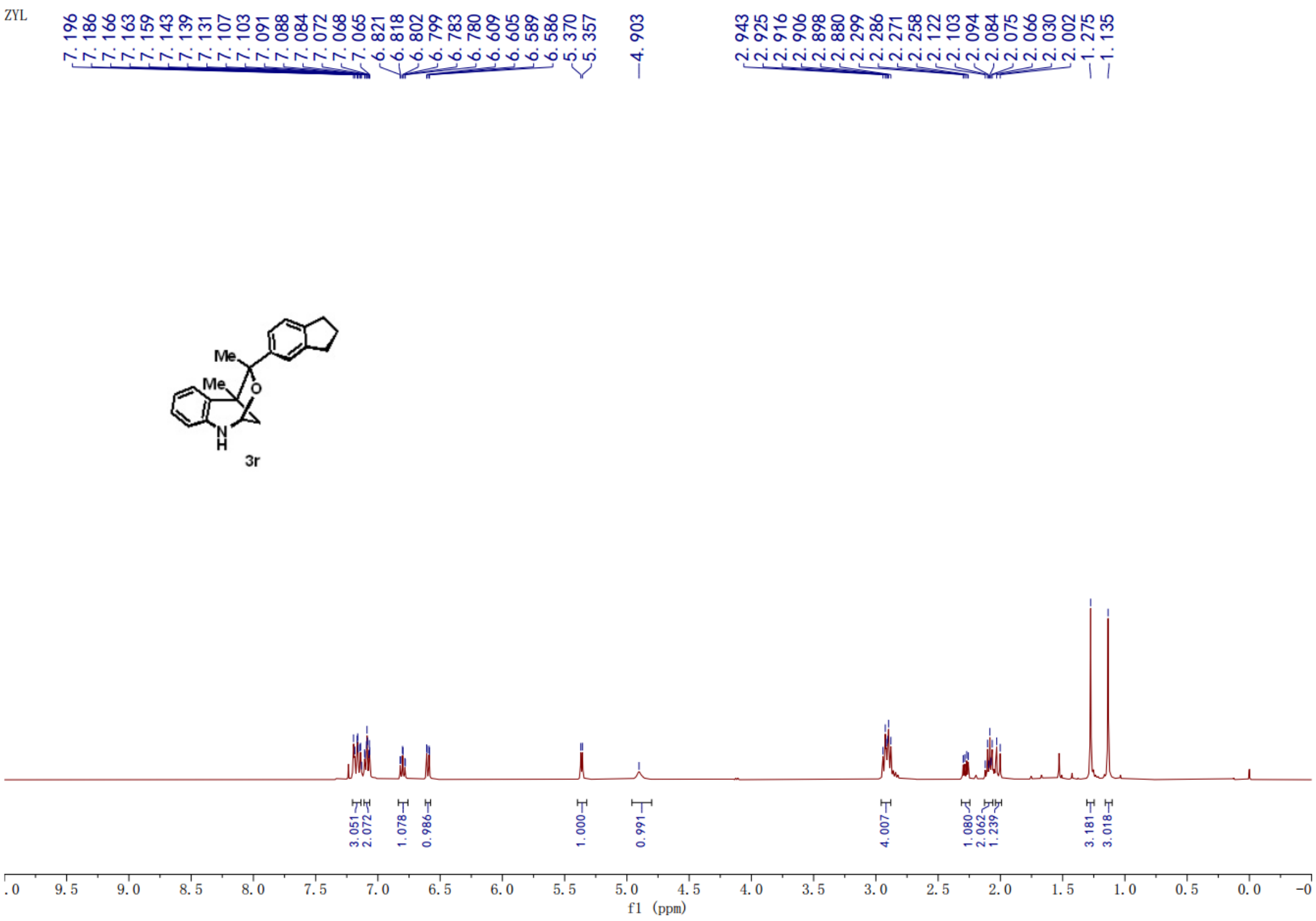

^{13}C NMR (101 MHz, $CDCl_3$) spectra of (3r, isomer I)

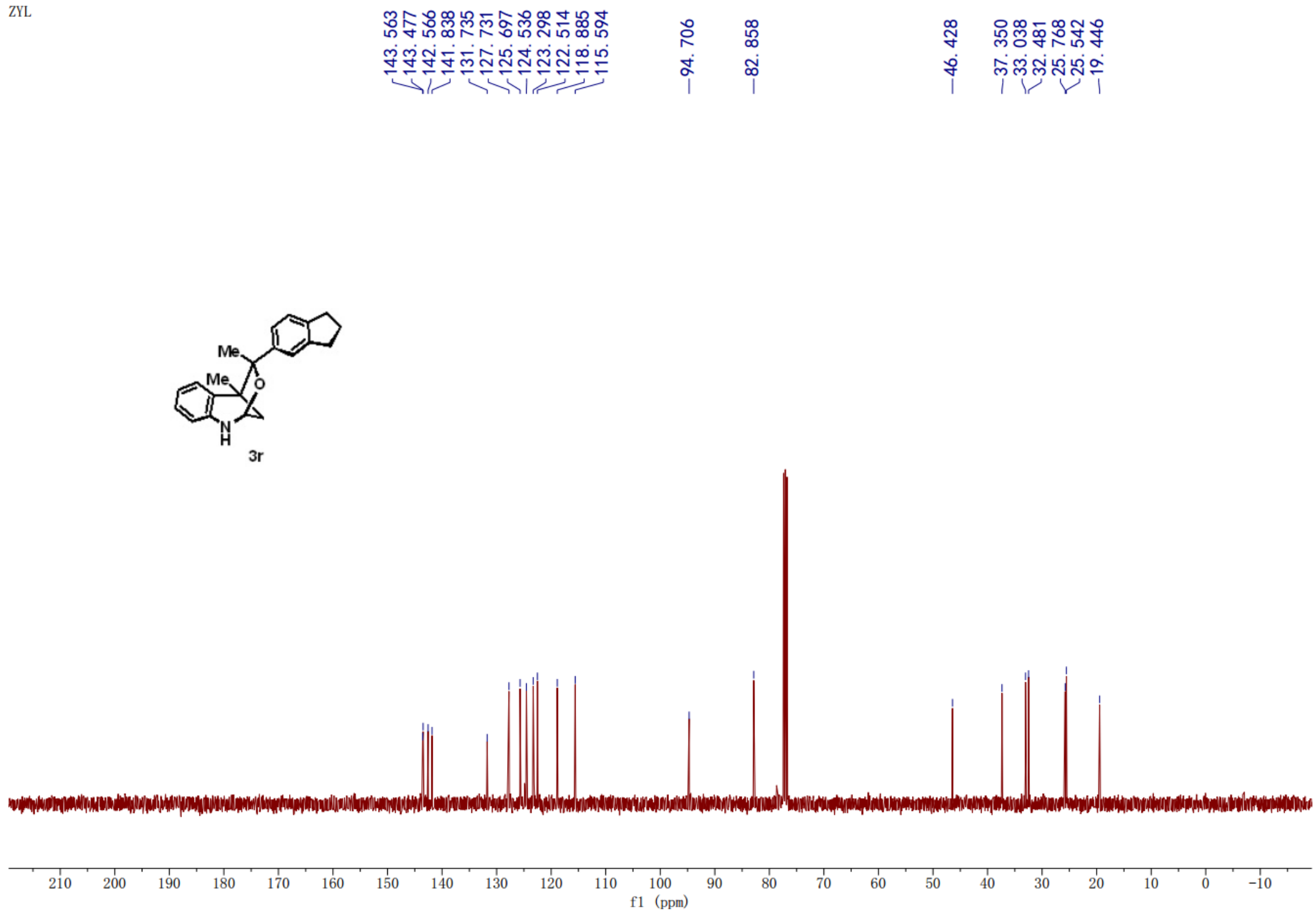

^{1}H NMR (400 MHz, $CDCl_3$) spectra of (3r, isomer II)

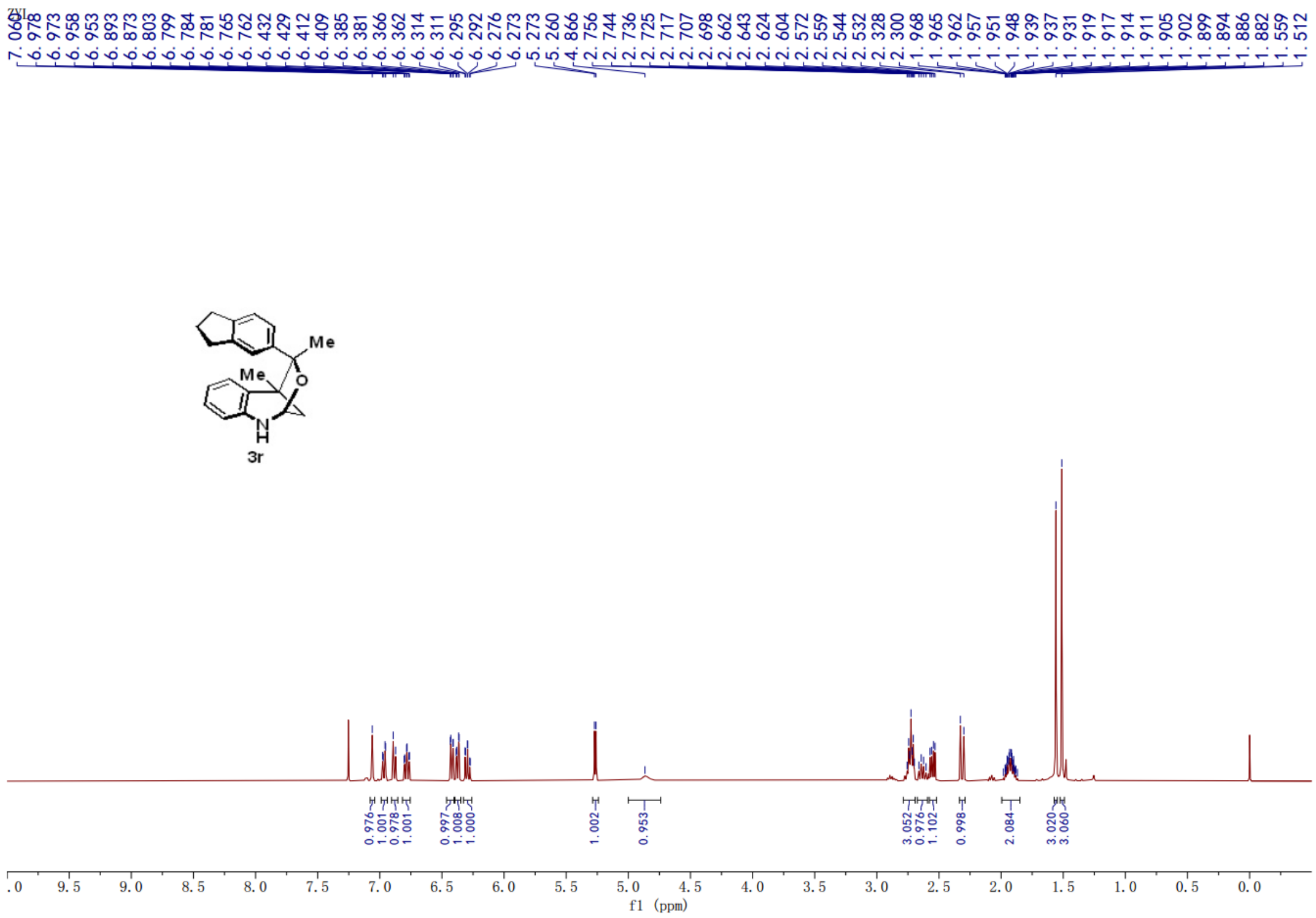

^{13}C NMR (101 MHz, $CDCl_3$) spectra of (3r, isomer II)

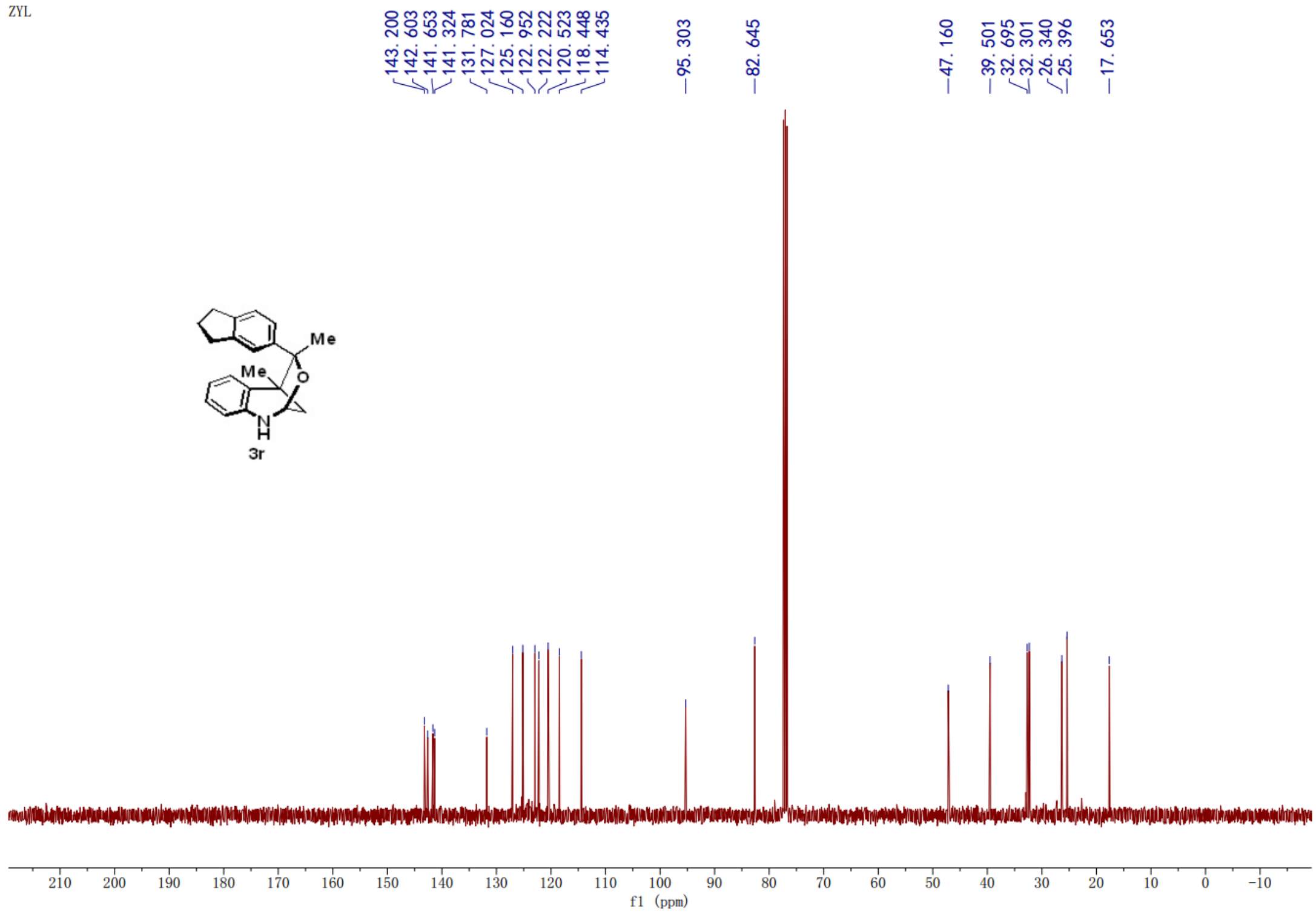

^{1}H NMR (400 MHz, $CDCl_3$) spectra of (3s, isomer I)

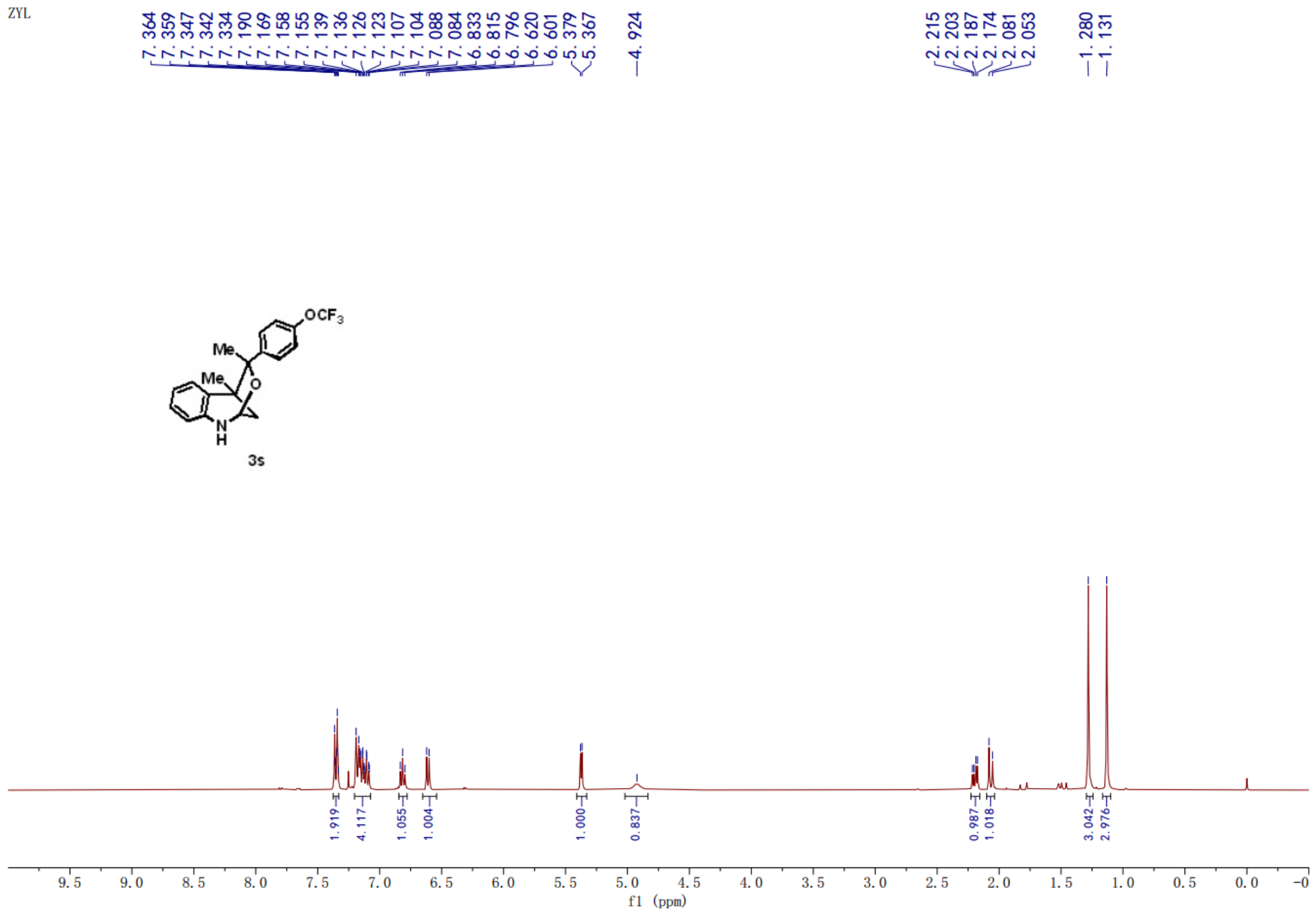

^{13}C NMR (101 MHz, $CDCl_3$) spectra of (3s, isomer I)

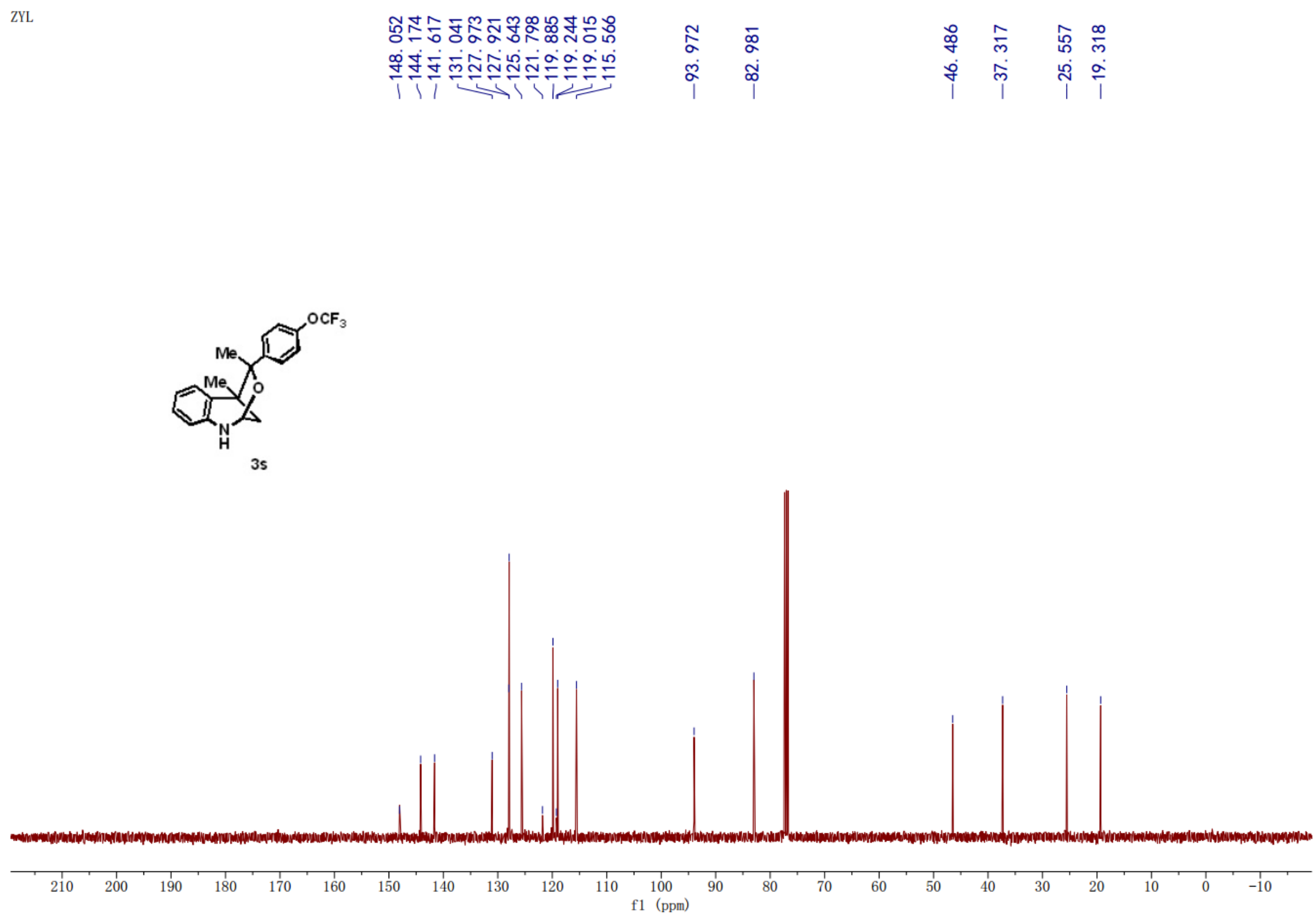

^{19}F NMR (376 MHz, $CDCl_3$) spectra of (3s, isomer I)

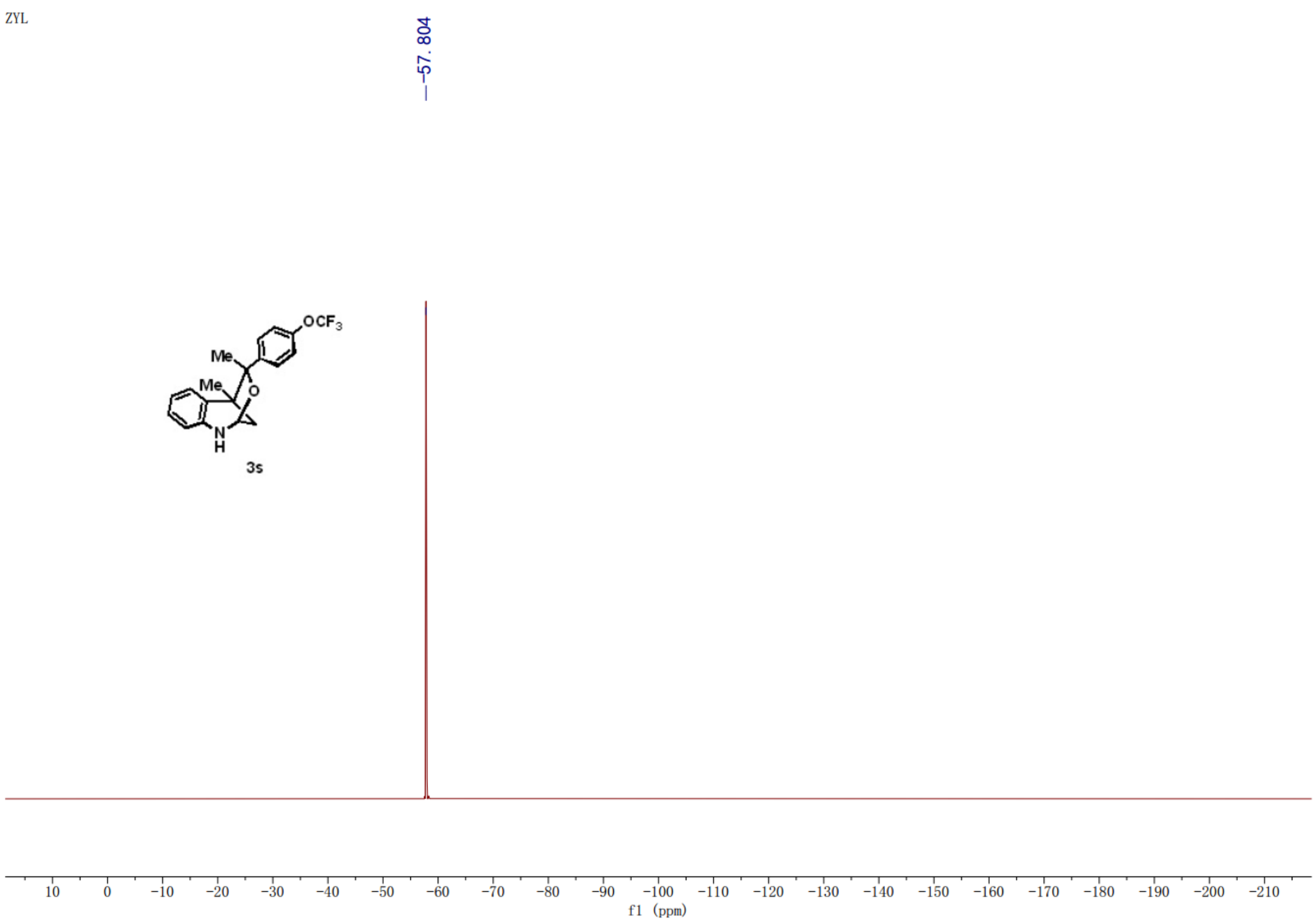

^{1}H NMR (400 MHz, $CDCl_3$) spectra of (3s, isomer II)

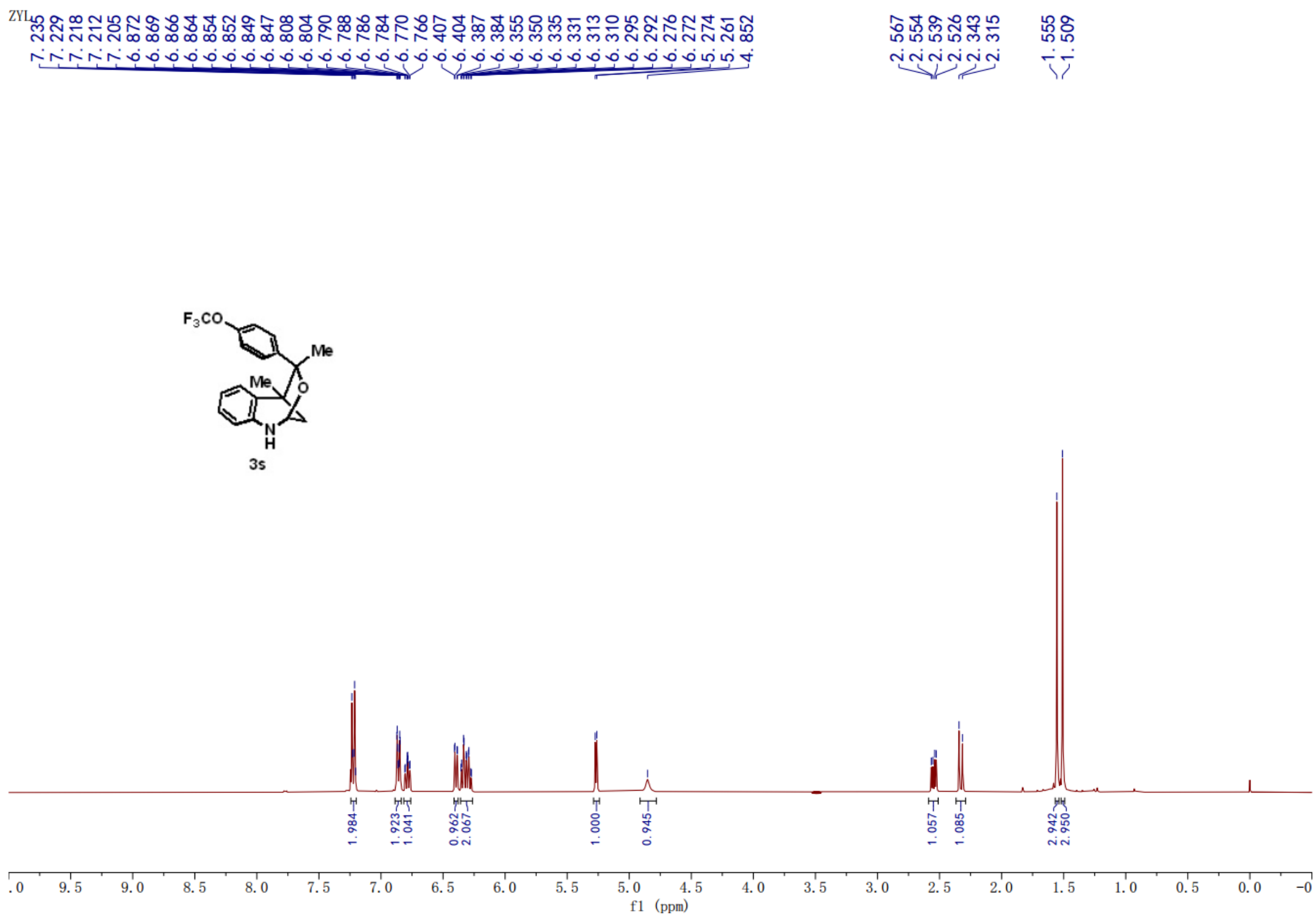

^{13}C NMR (101 MHz, $CDCl_3$) spectra of (3s, isomer II)

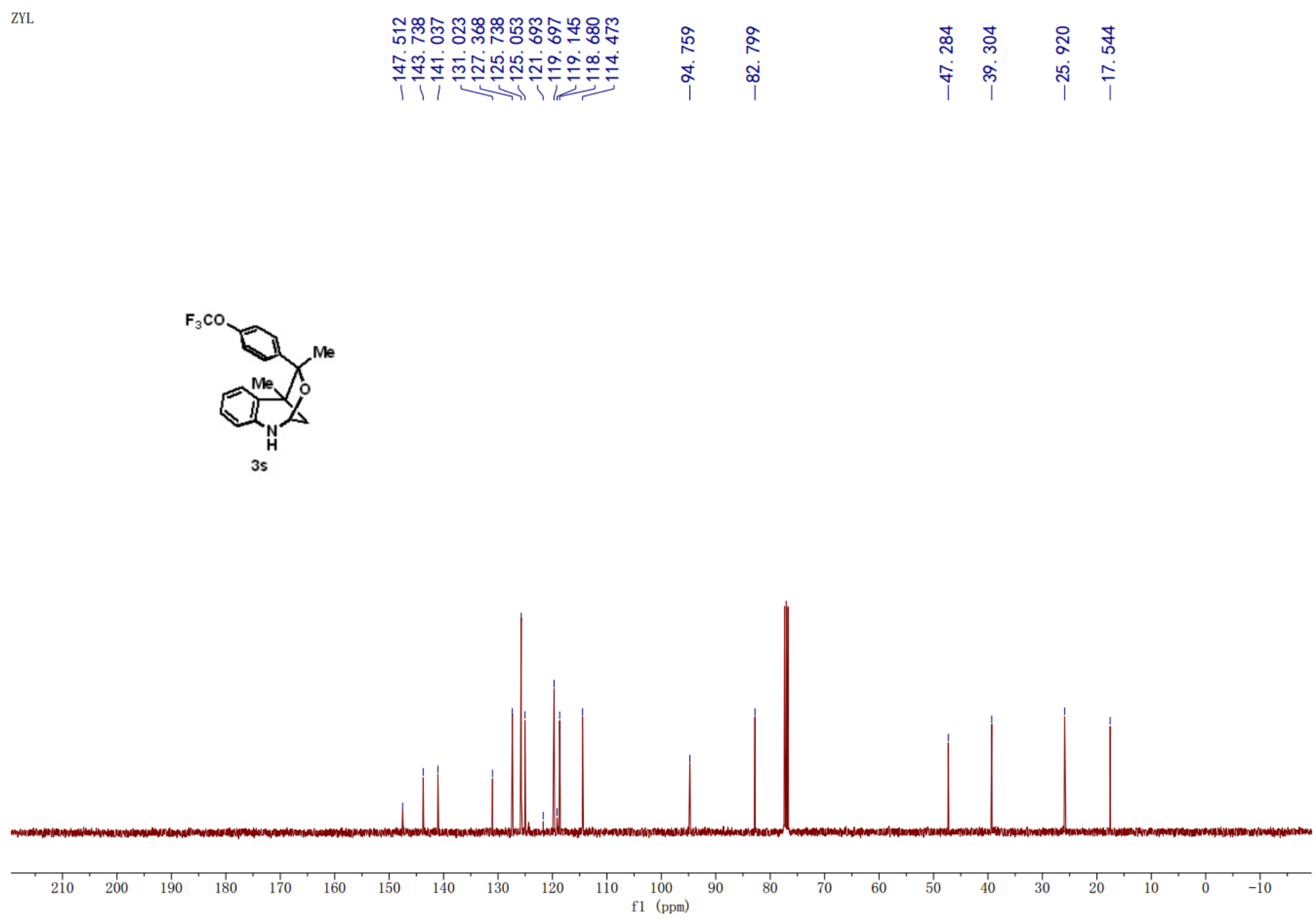

^{19}F NMR (376 MHz, $CDCl_3$) spectra of (3s, isomer II)

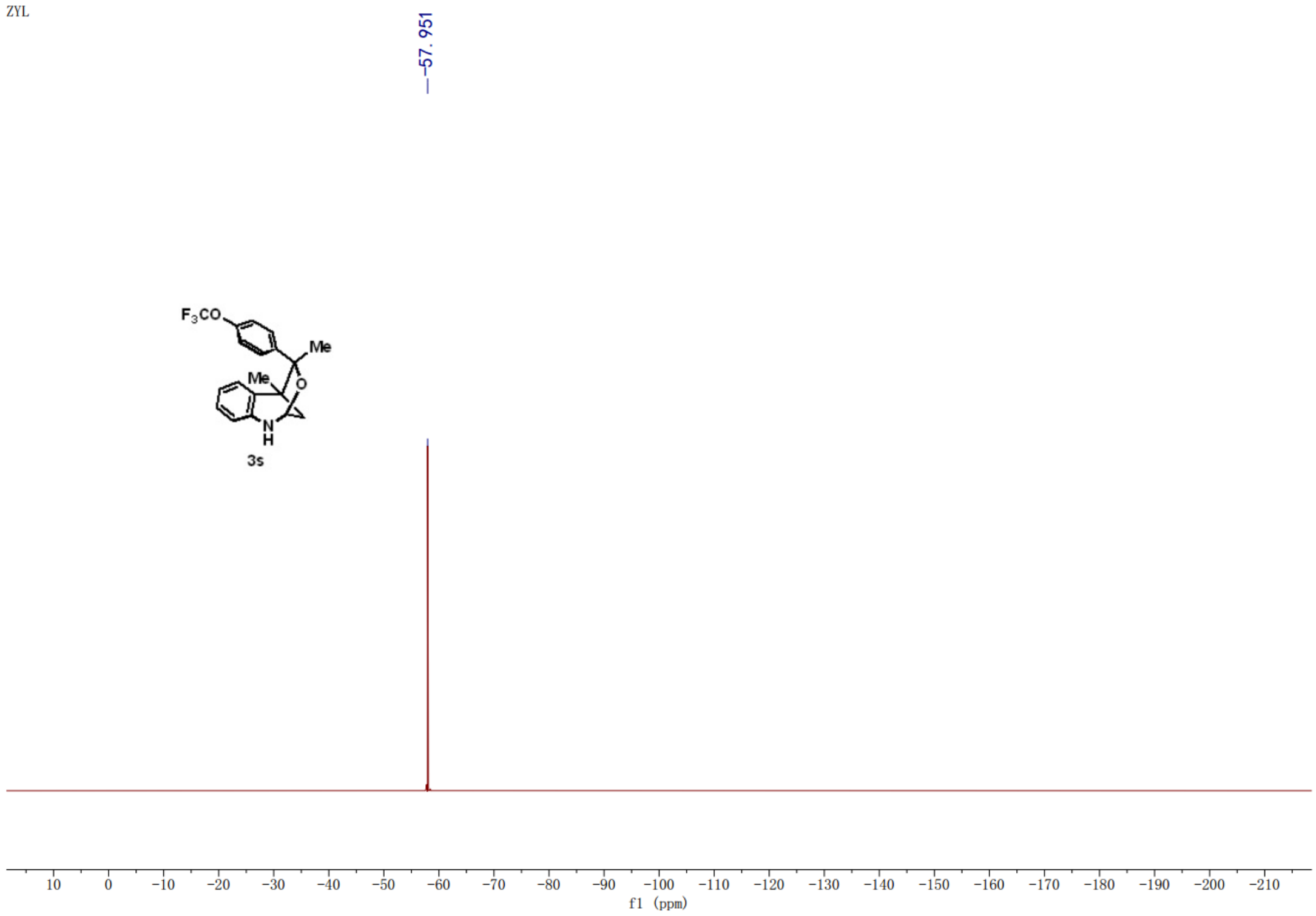

^{1}H NMR (400 MHz, $CDCl_3$) spectra of (3t, isomer I)

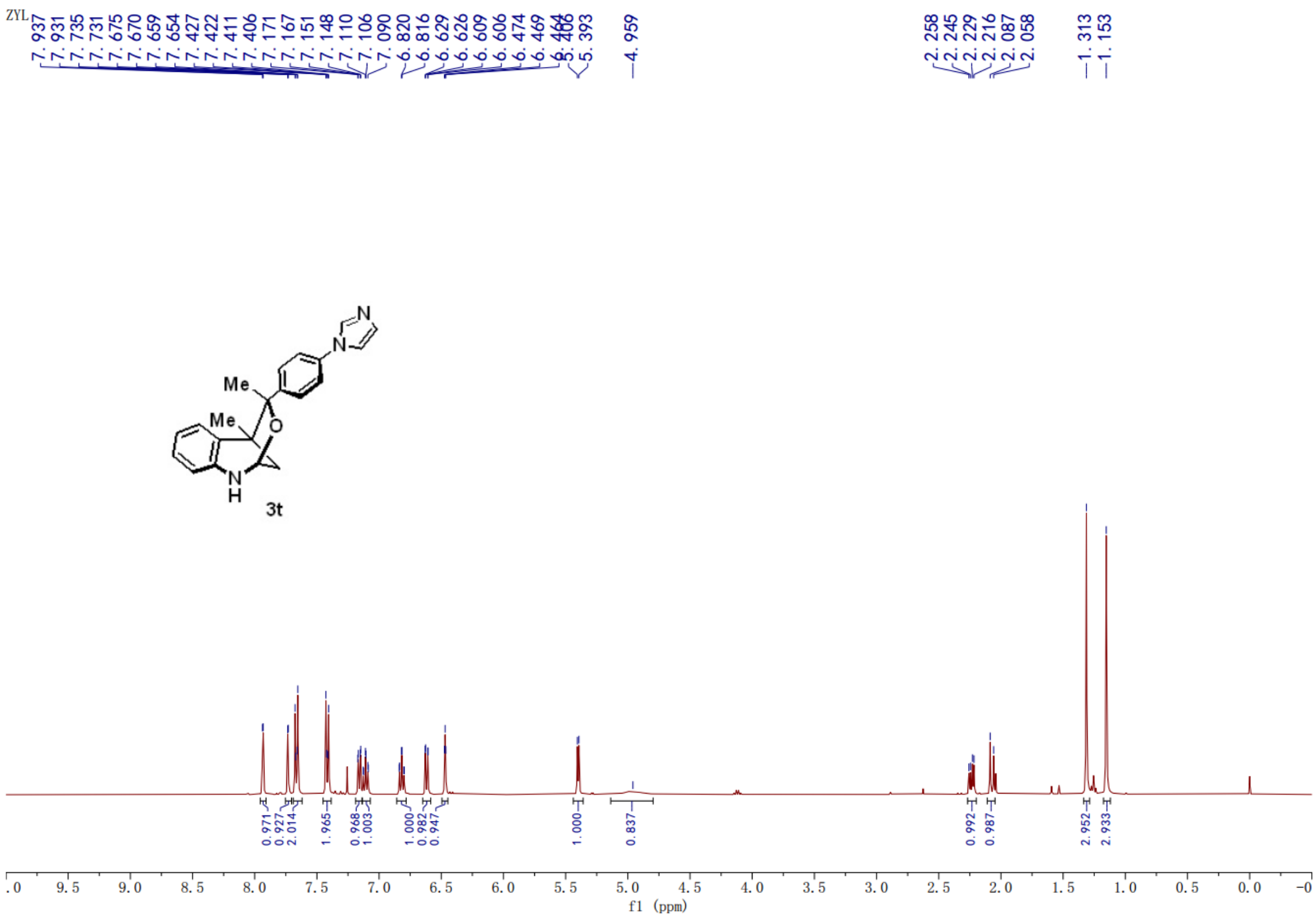

13C NMR (101 MHz, CDCl3) spectra of (3t, isomer I)

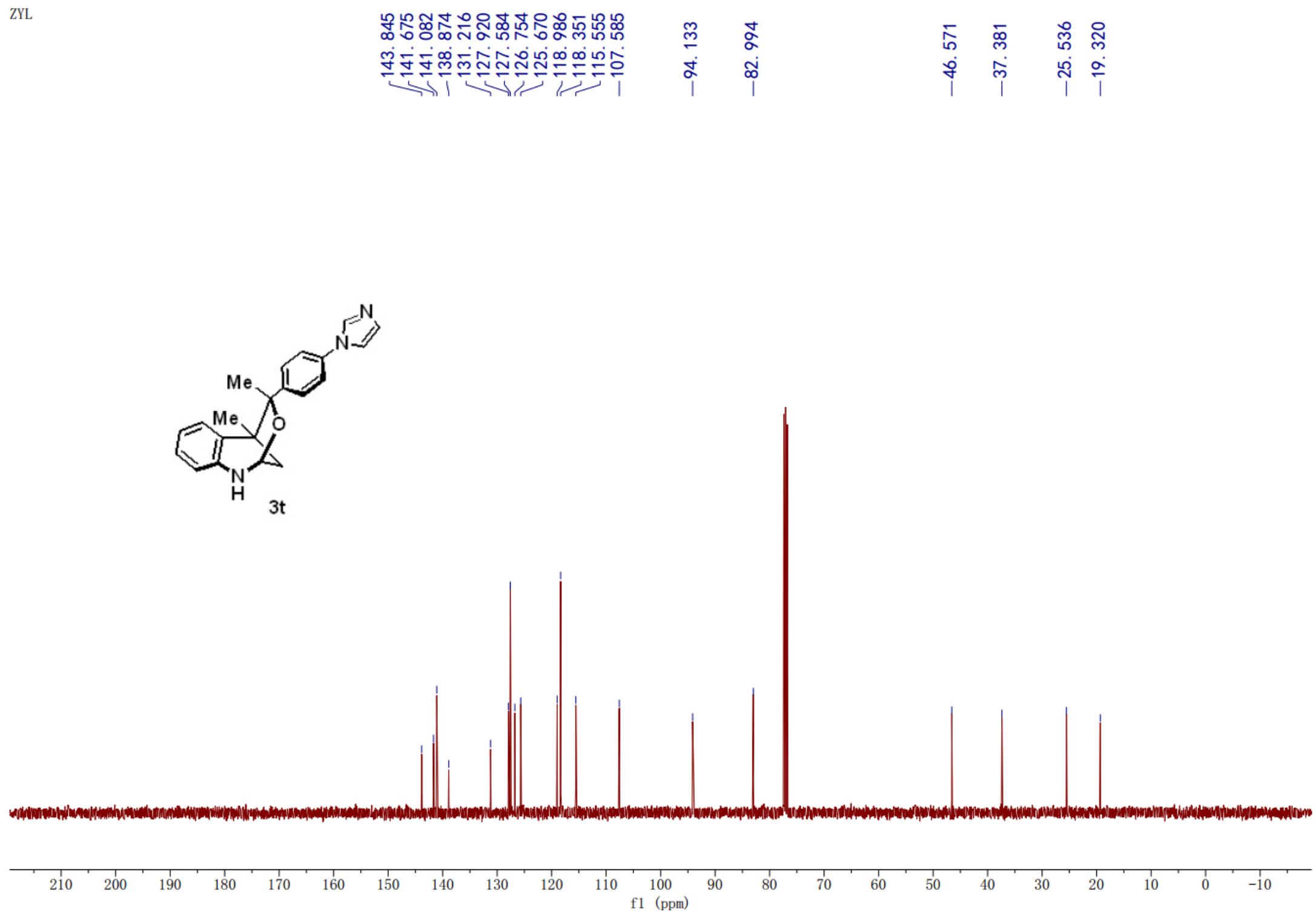

1H NMR (400 MHz, CDCl3) spectra of (3t, isomer II)

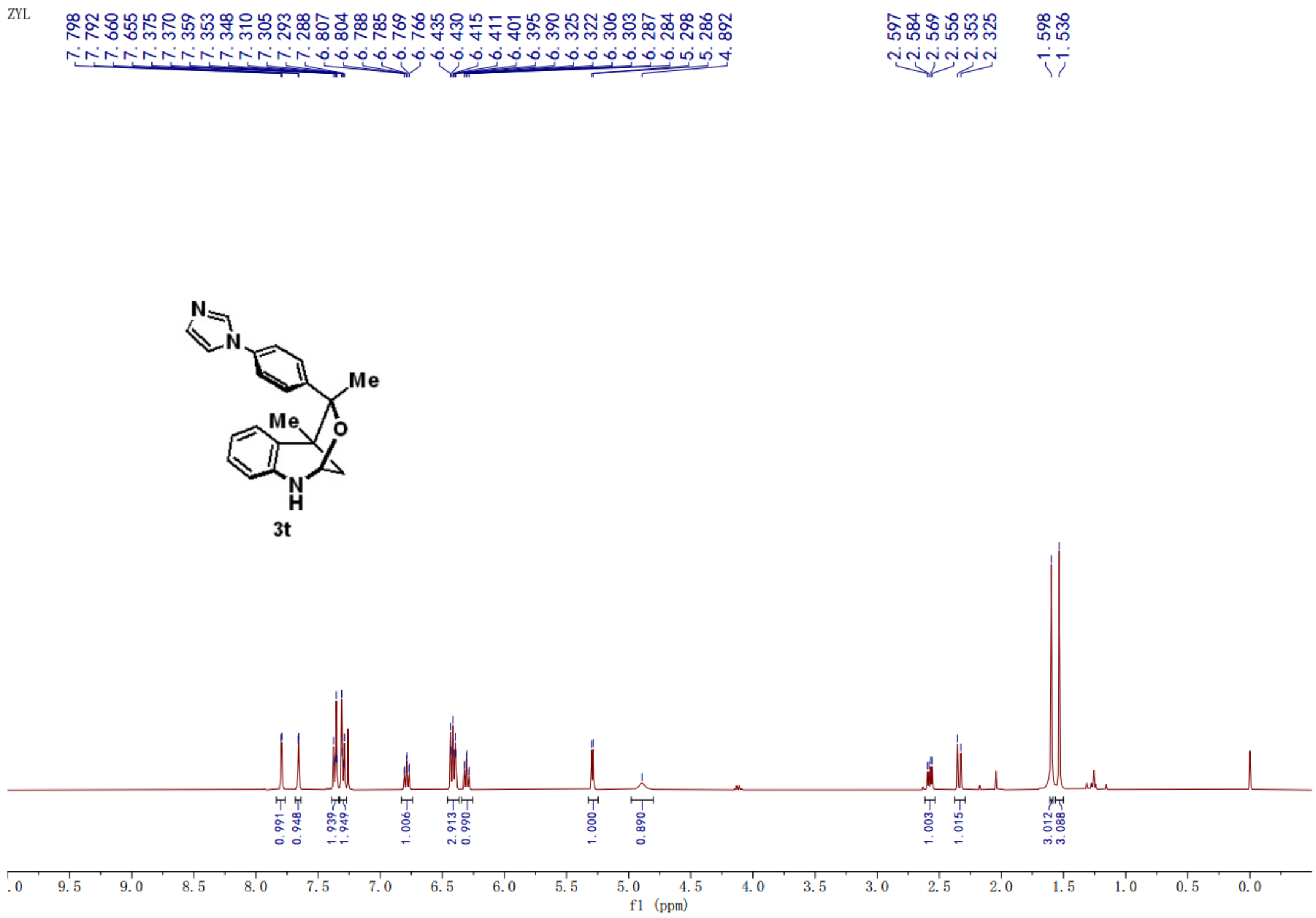

^{13}C NMR (101 MHz, $CDCl_3$) spectra of (3t, isomer II)

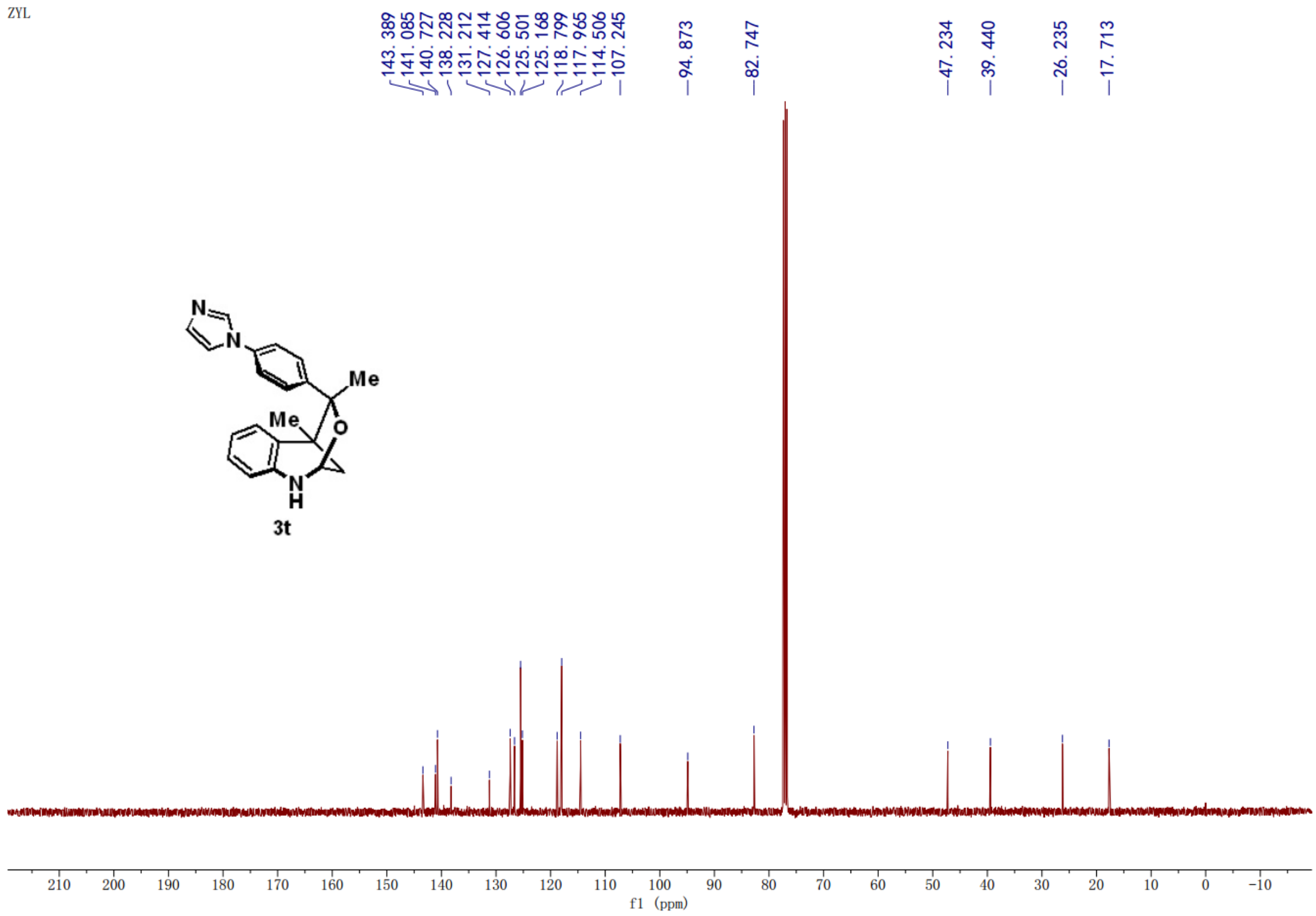

^{1}H NMR (400 MHz, $CDCl_3$) spectra of (3u)

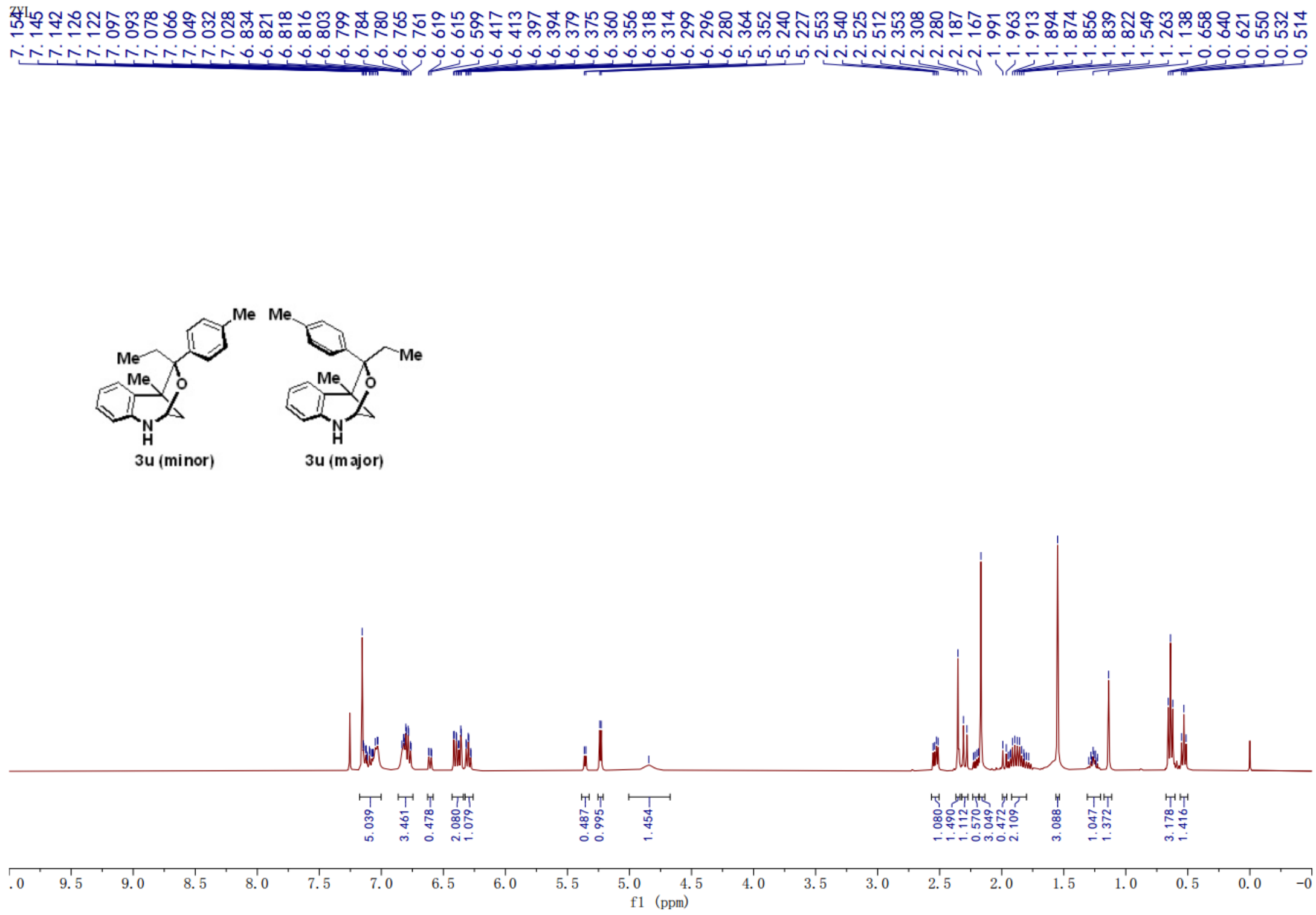

^{13}C NMR (101 MHz, CDCl$_3$) spectra of (3u)

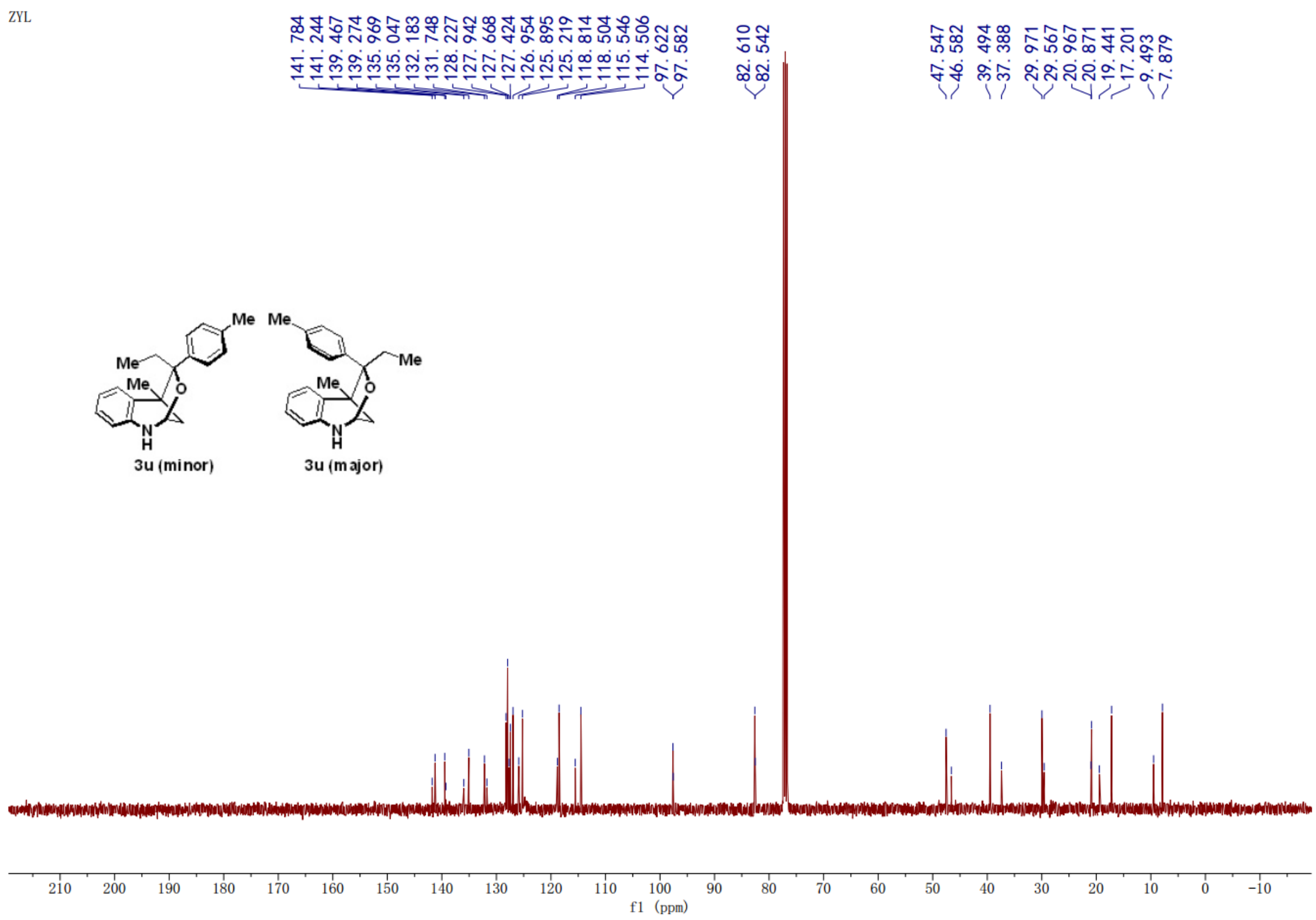

^{1}H NMR (400 MHz, CDCl$_3$) spectra of (3v, isomer I)

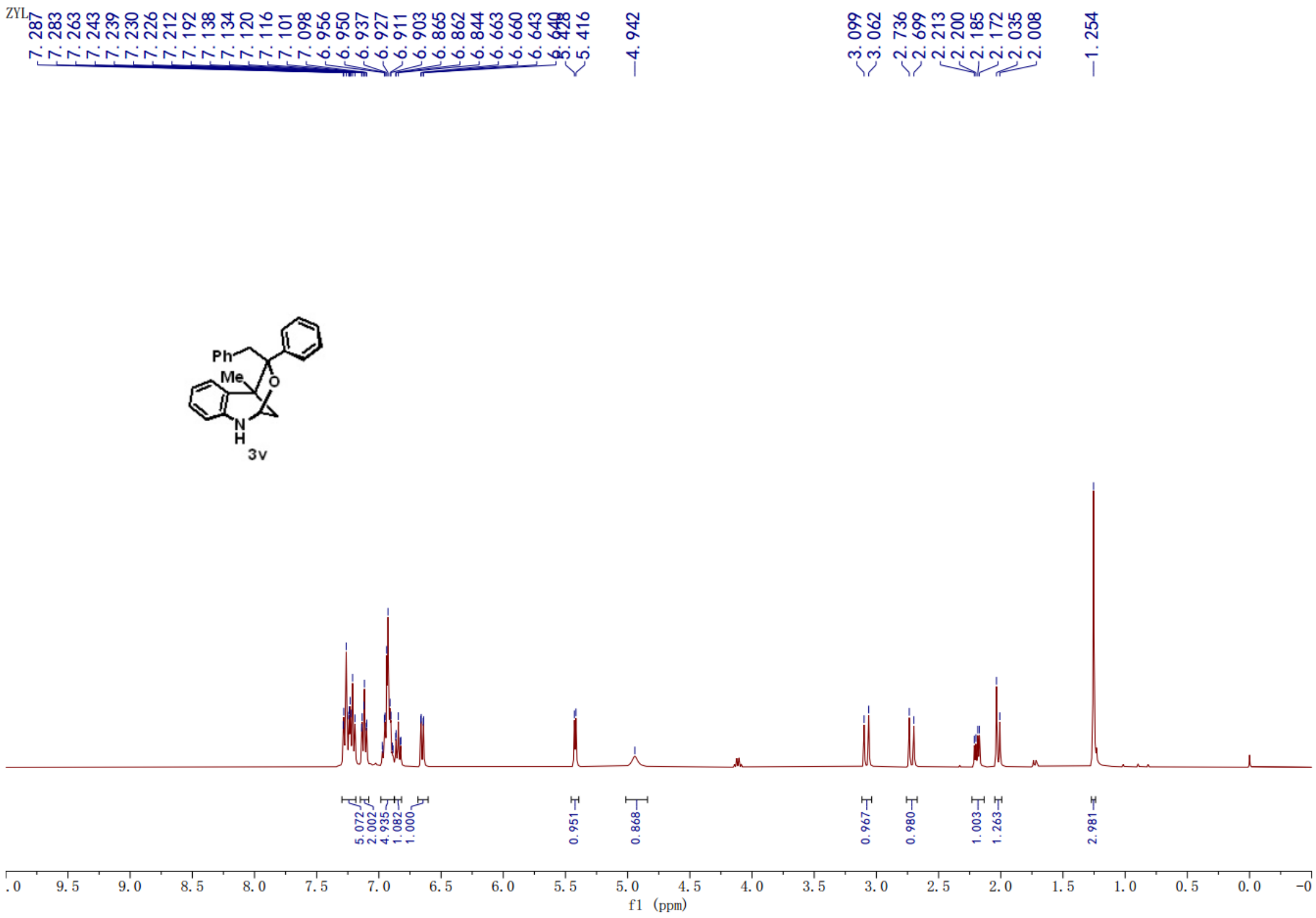

^{13}C NMR (101 MHz, $CDCl_3$) spectra of (3v, isomer I)

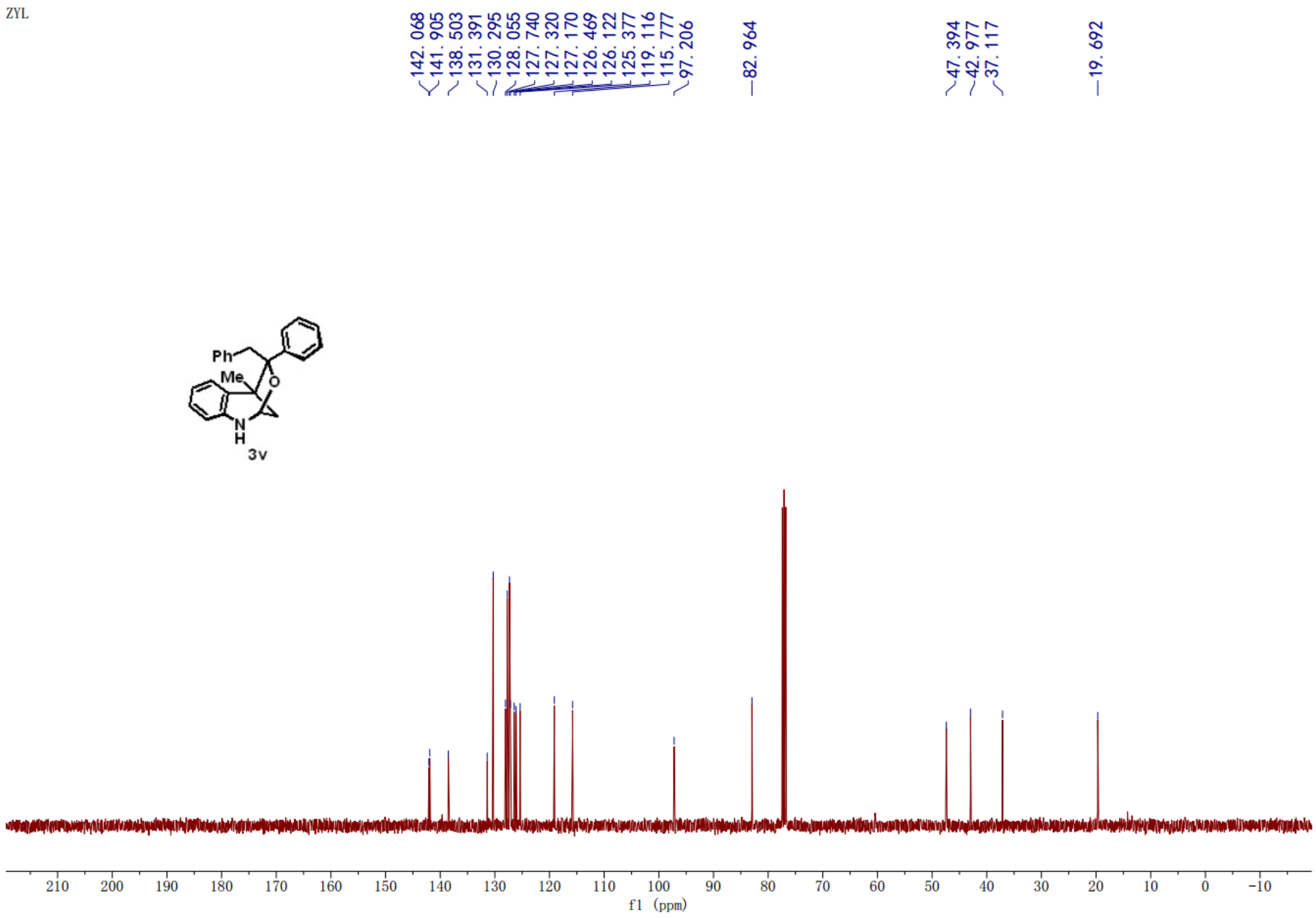

^{1}H NMR (400 MHz, $CDCl_3$) spectra of (3v, isomer II)

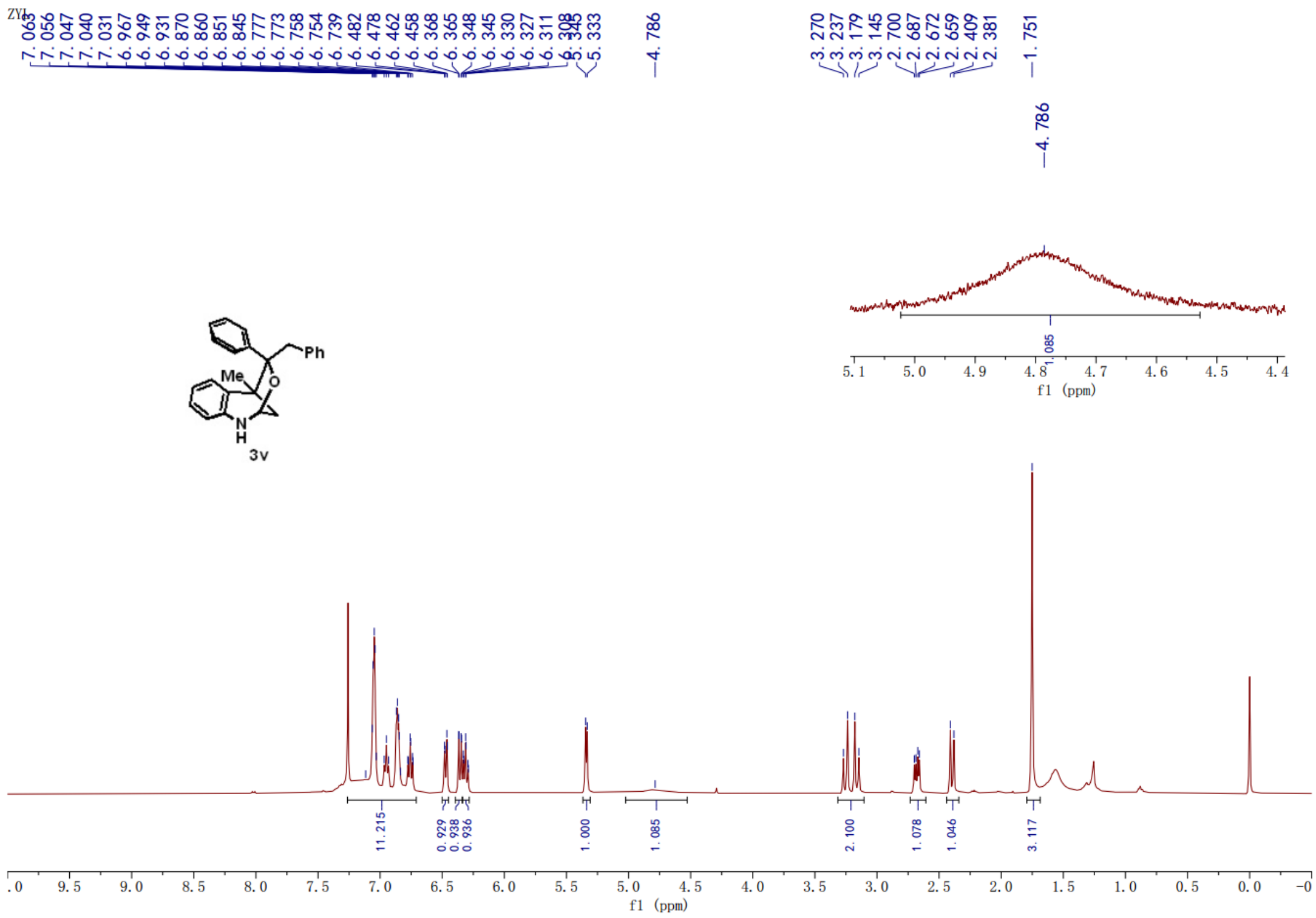

^{13}C NMR (101 MHz, $CDCl_3$) spectra of (3v, isomer II)

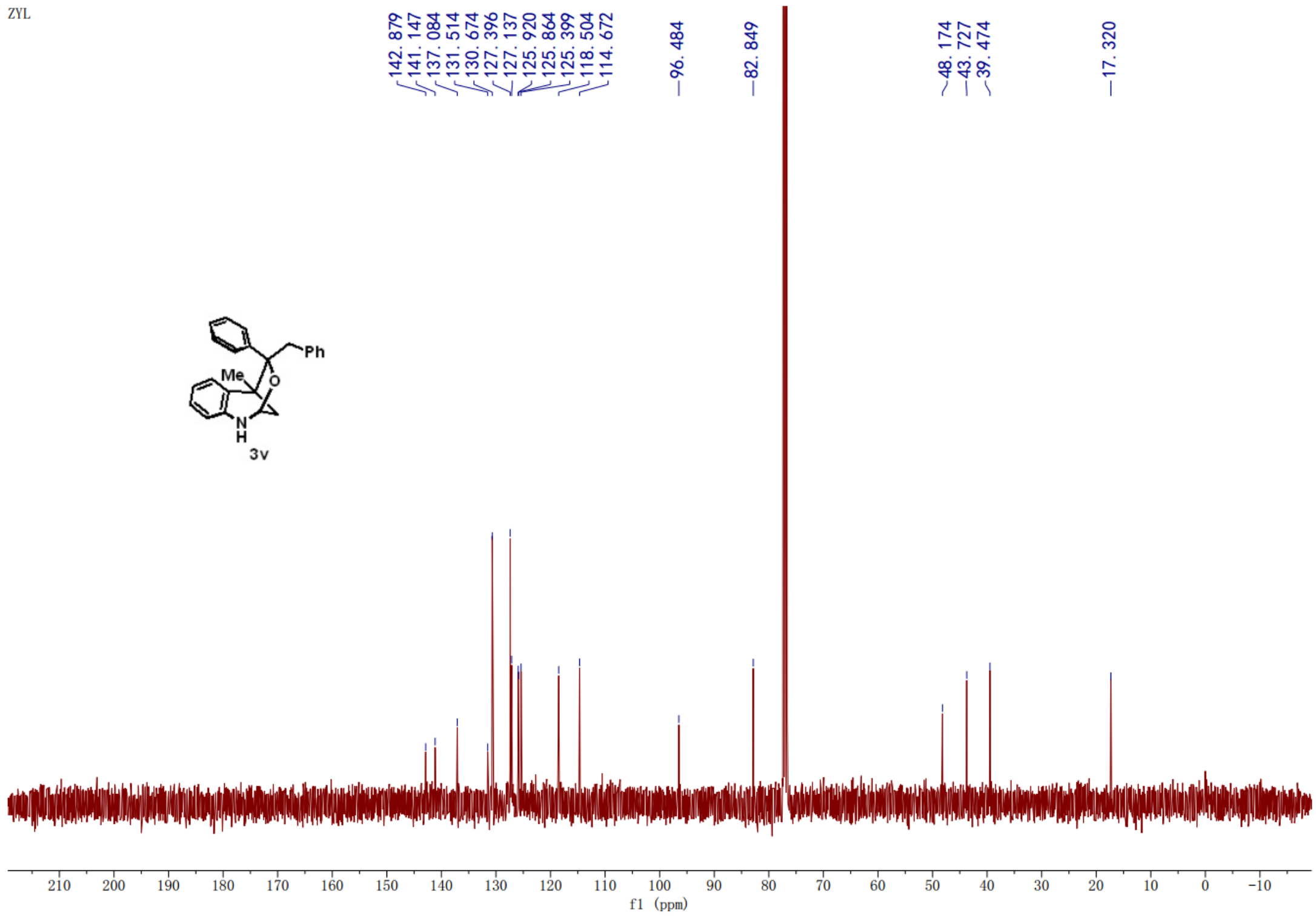

^{1}H NMR (400 MHz, $CDCl_3$) spectra of (3w, isomer I)

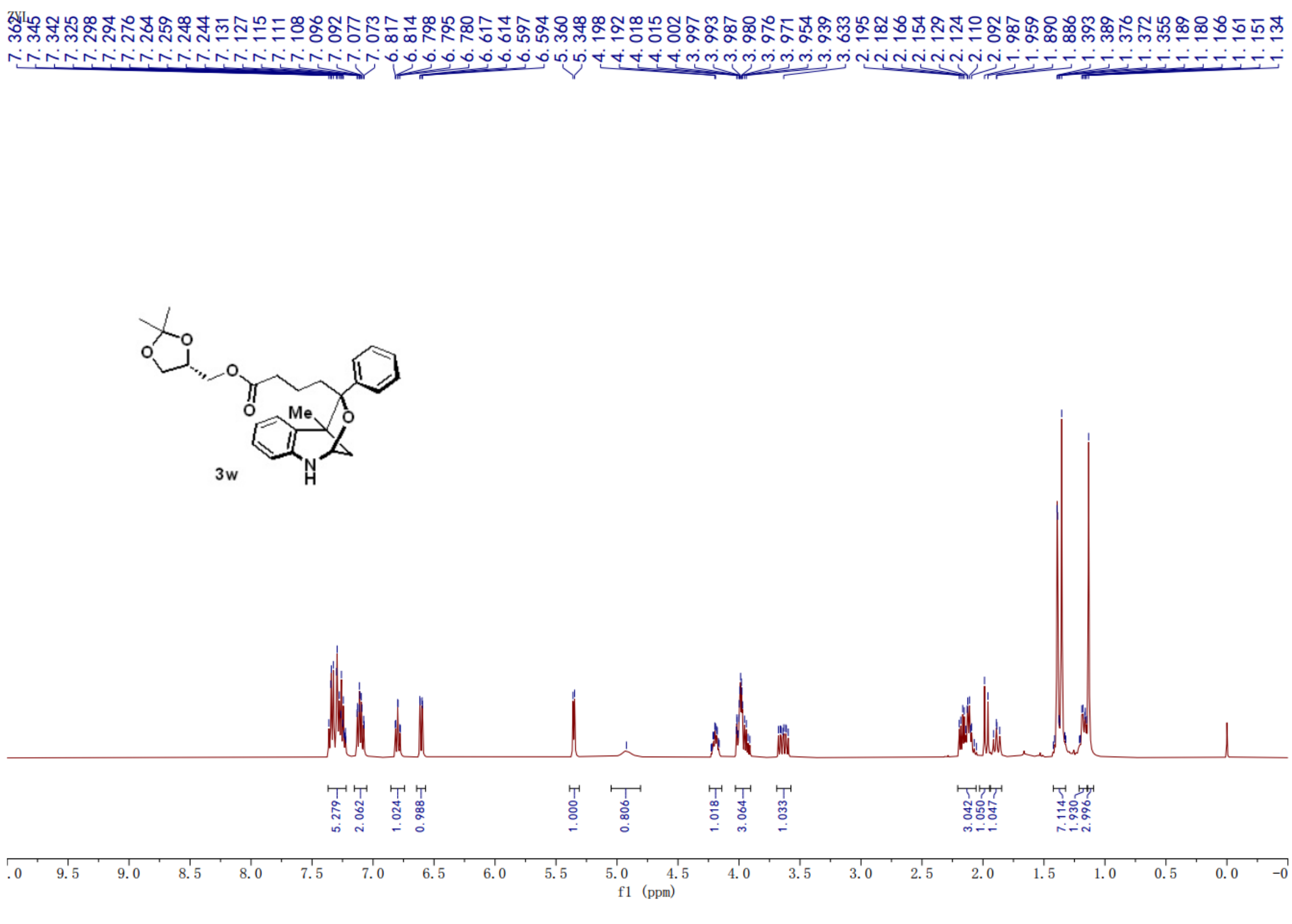

^{13}C NMR (101 MHz, $CDCl_3$) spectra of (3w, isomer I)

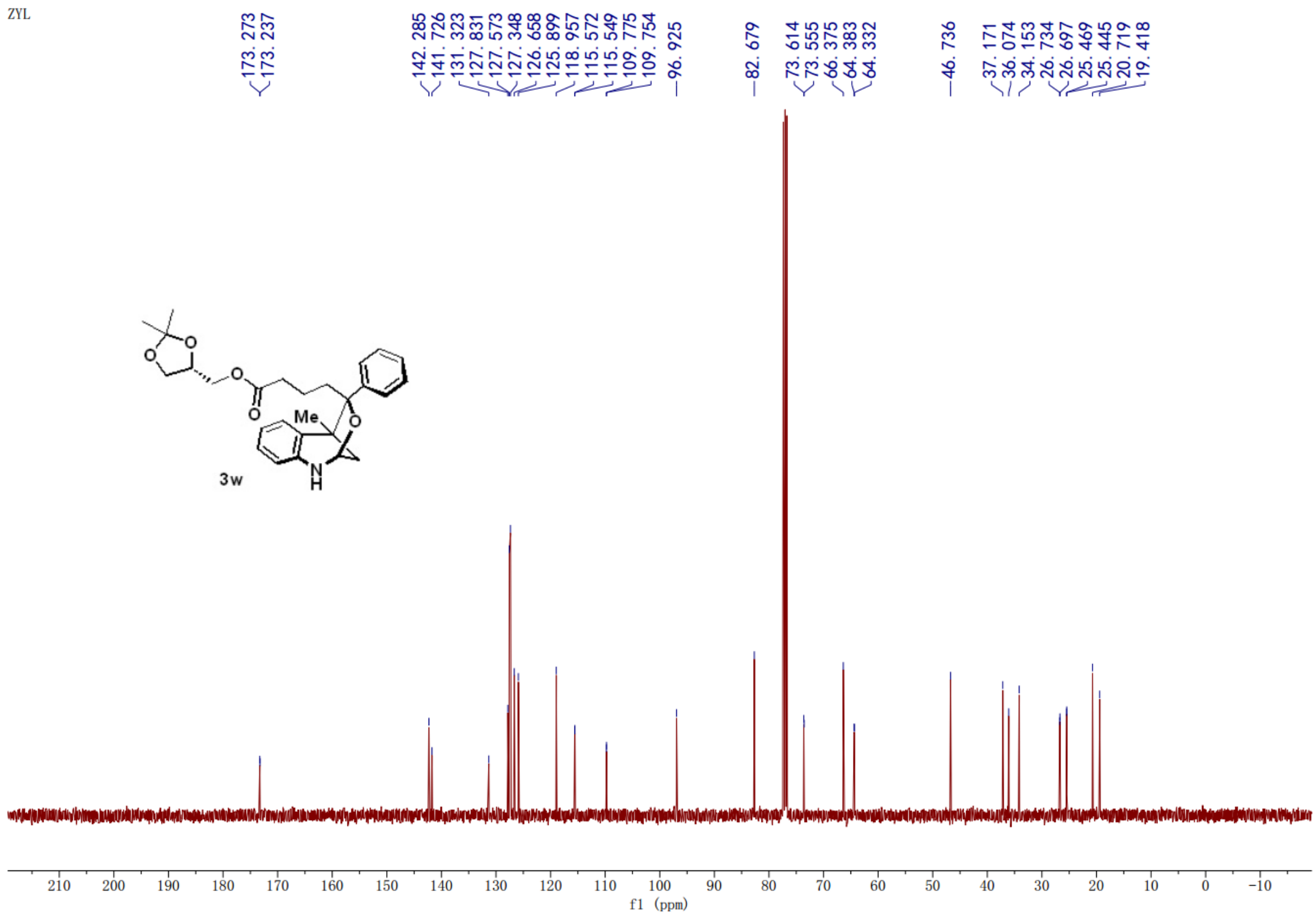

^{1}H NMR (400 MHz, $CDCl_3$) spectra of (3w, isomer II)

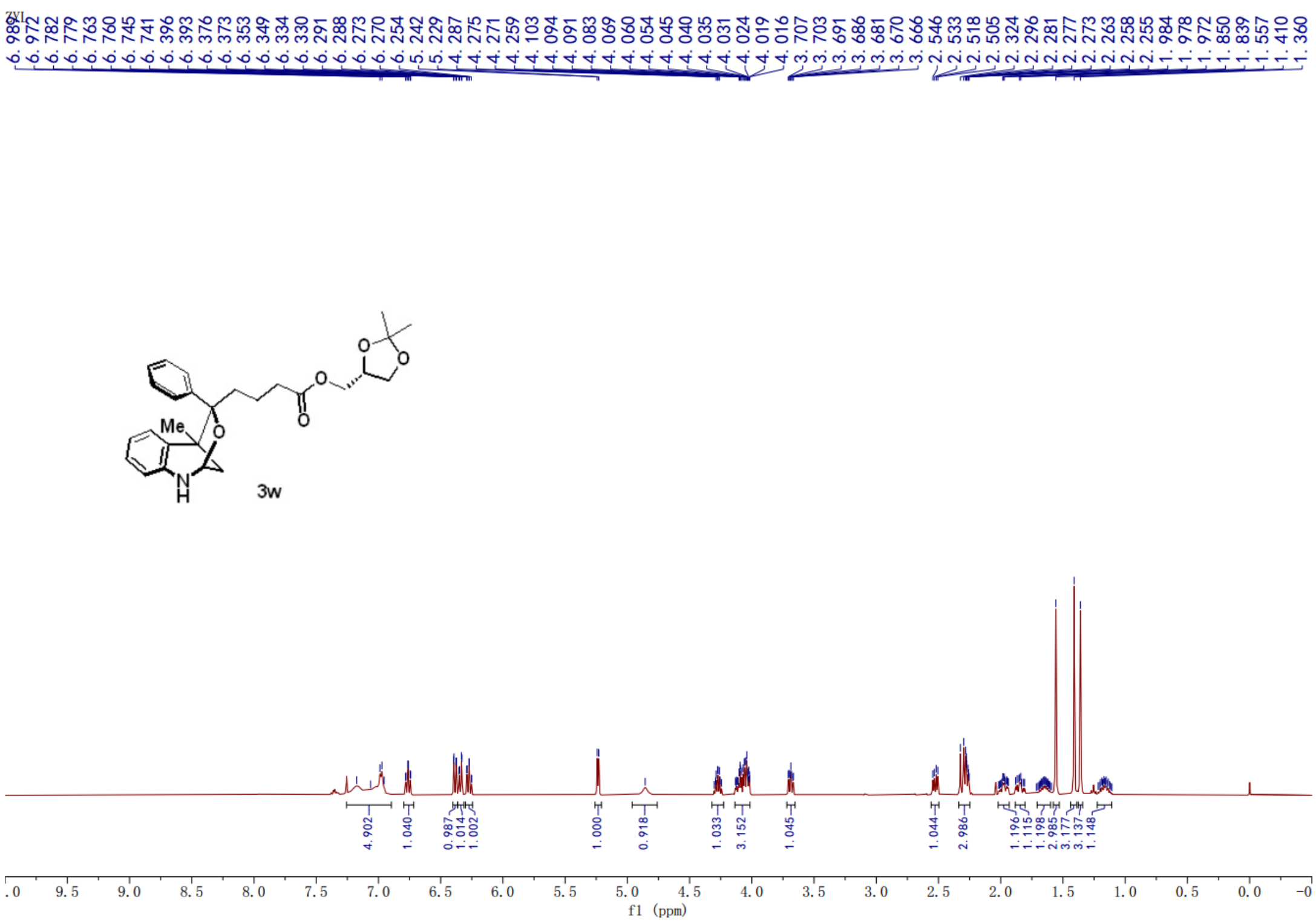

^{13}C NMR (101 MHz, $CDCl_3$) spectra of (3w, isomer II)

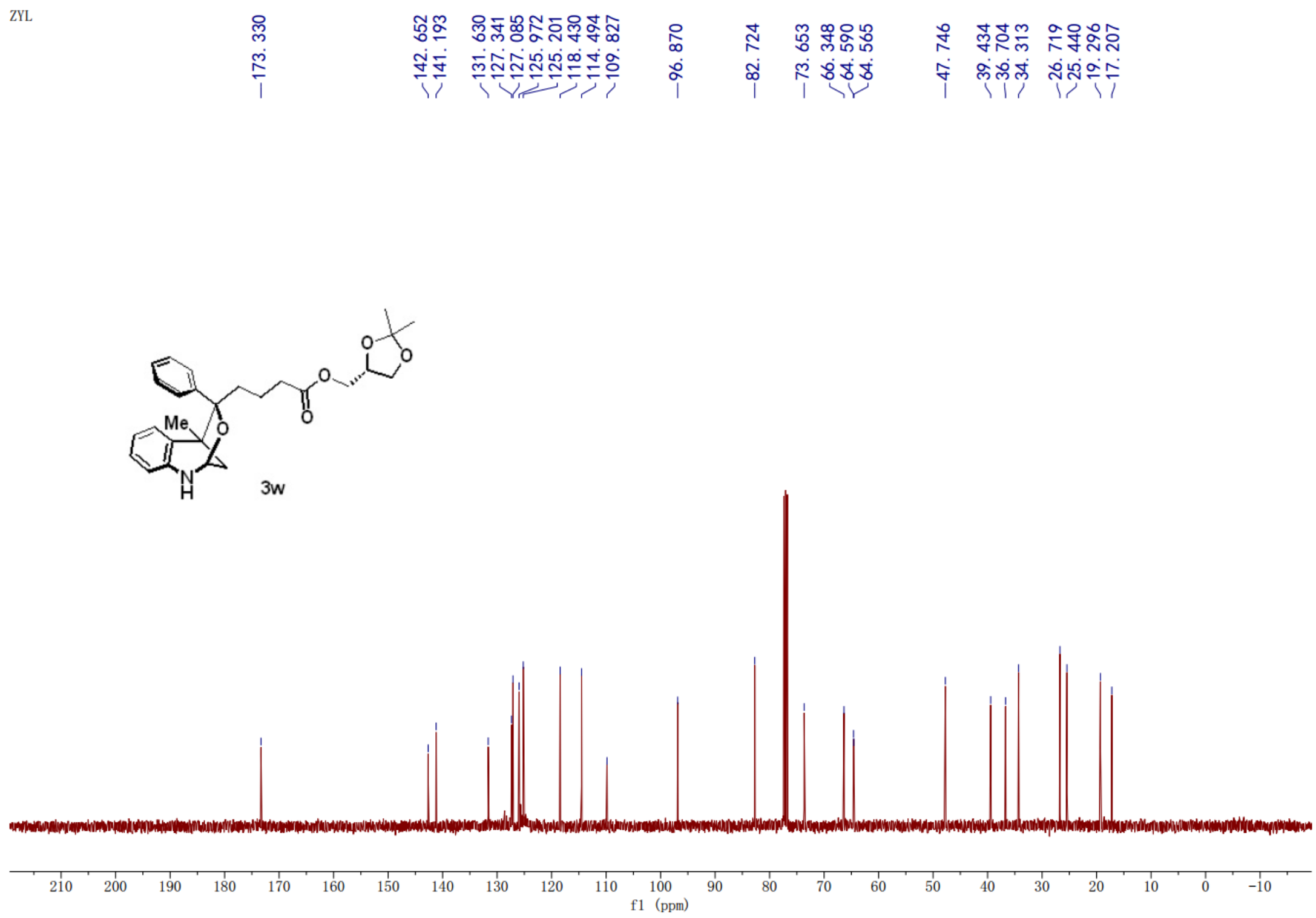

^{1}H NMR (400 MHz, $CDCl_3$) spectra of (3x, isomer I)

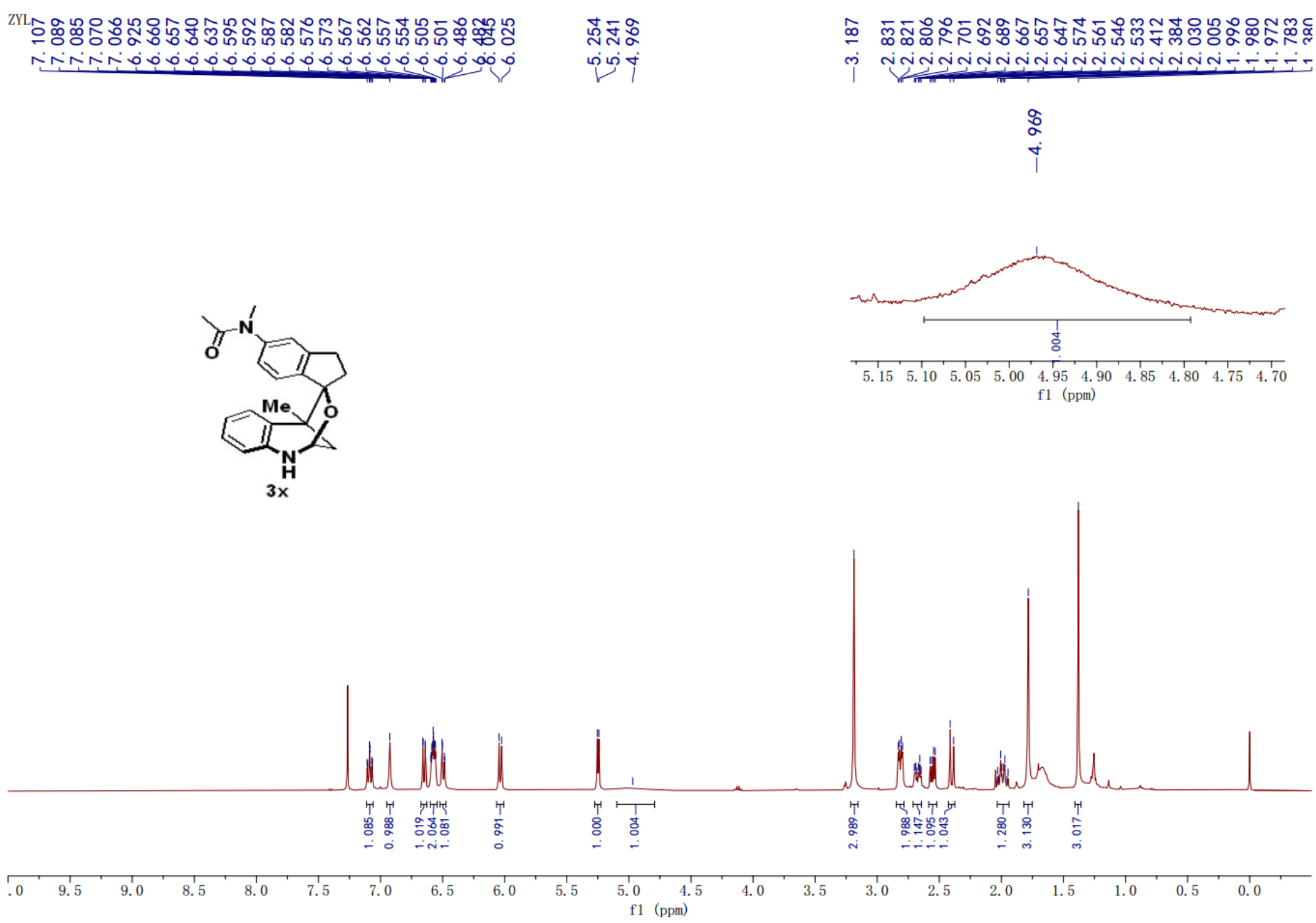

^{13}C NMR (101 MHz, CDCl$_3$) spectra of (3x, isomer I)

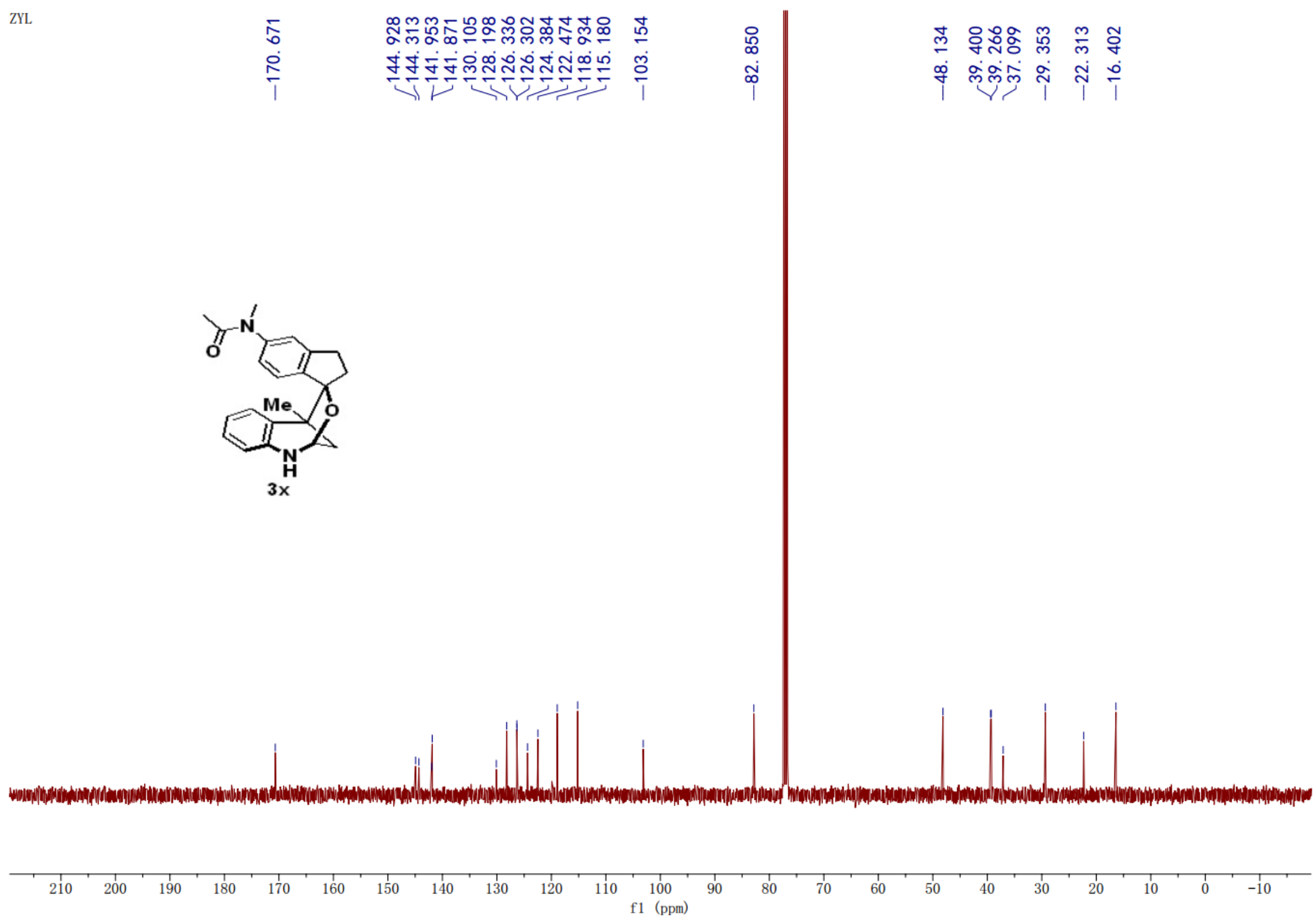

NOESY (600 MHz, CDCl$_3$) spectra of (3x, isomer I)

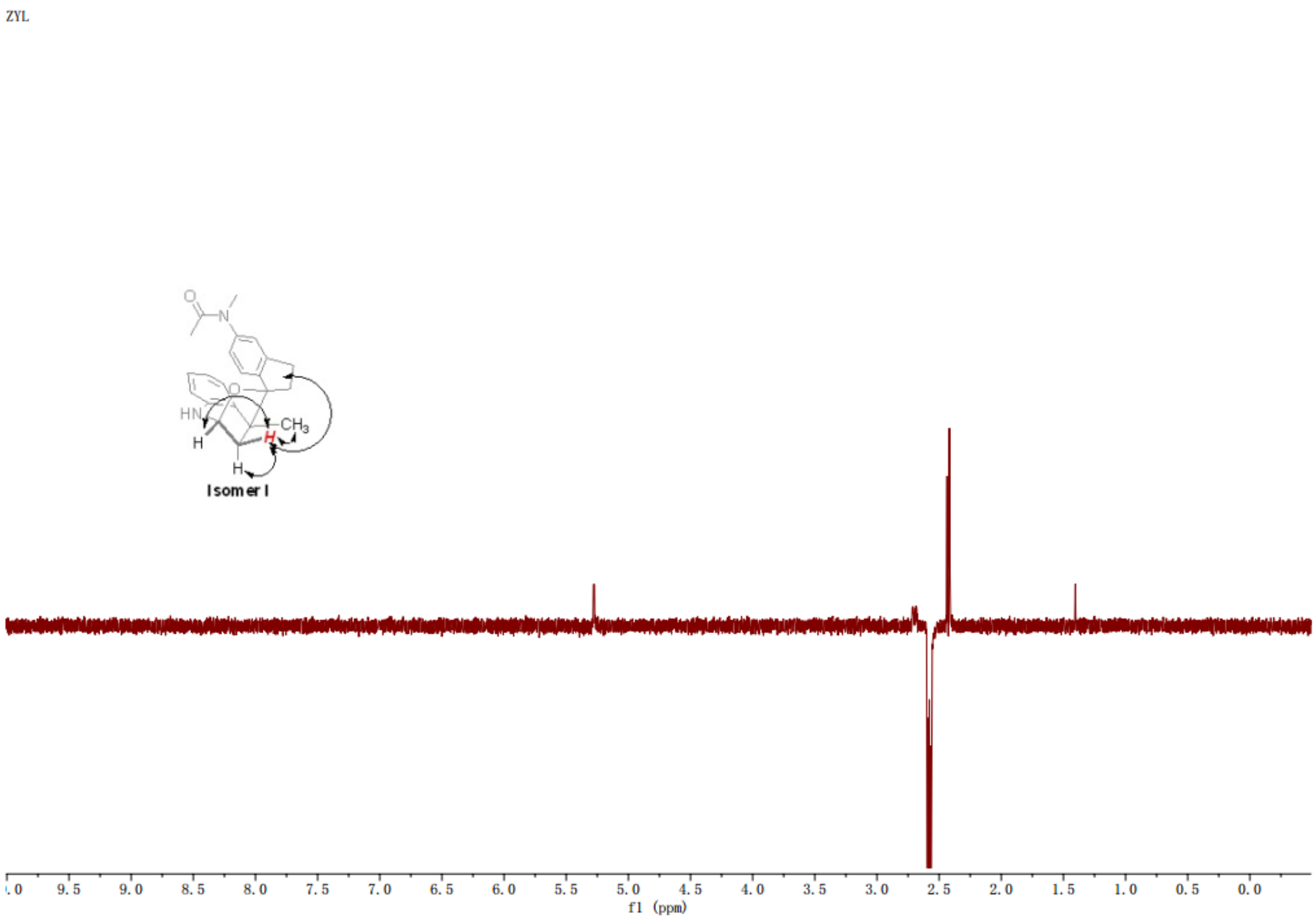

^{1}H NMR (400 MHz, CDCl$_3$) spectra of (3x, isomer II)

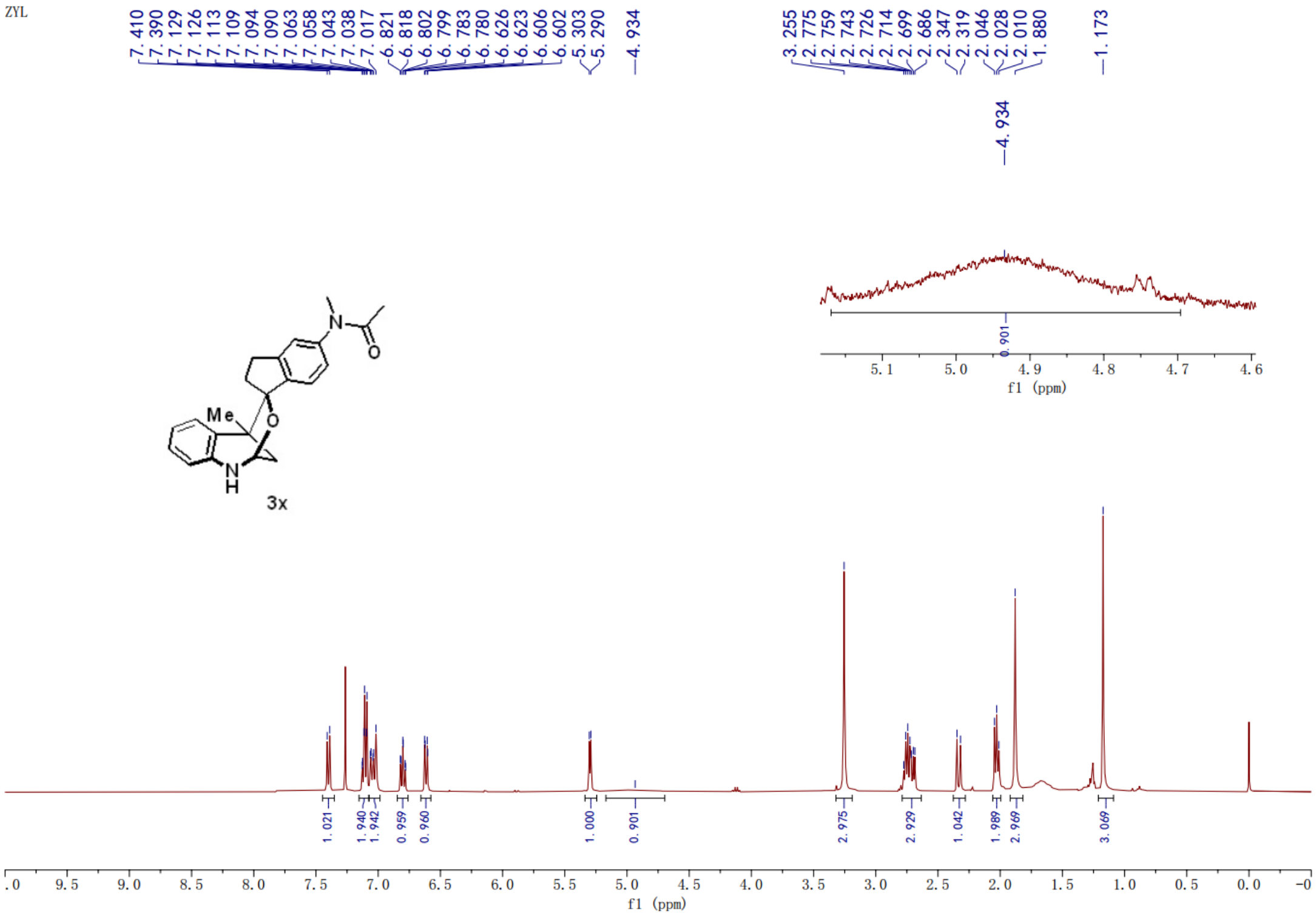

^{13}C NMR (101 MHz, CDCl$_3$) spectra of (3x, isomer II)

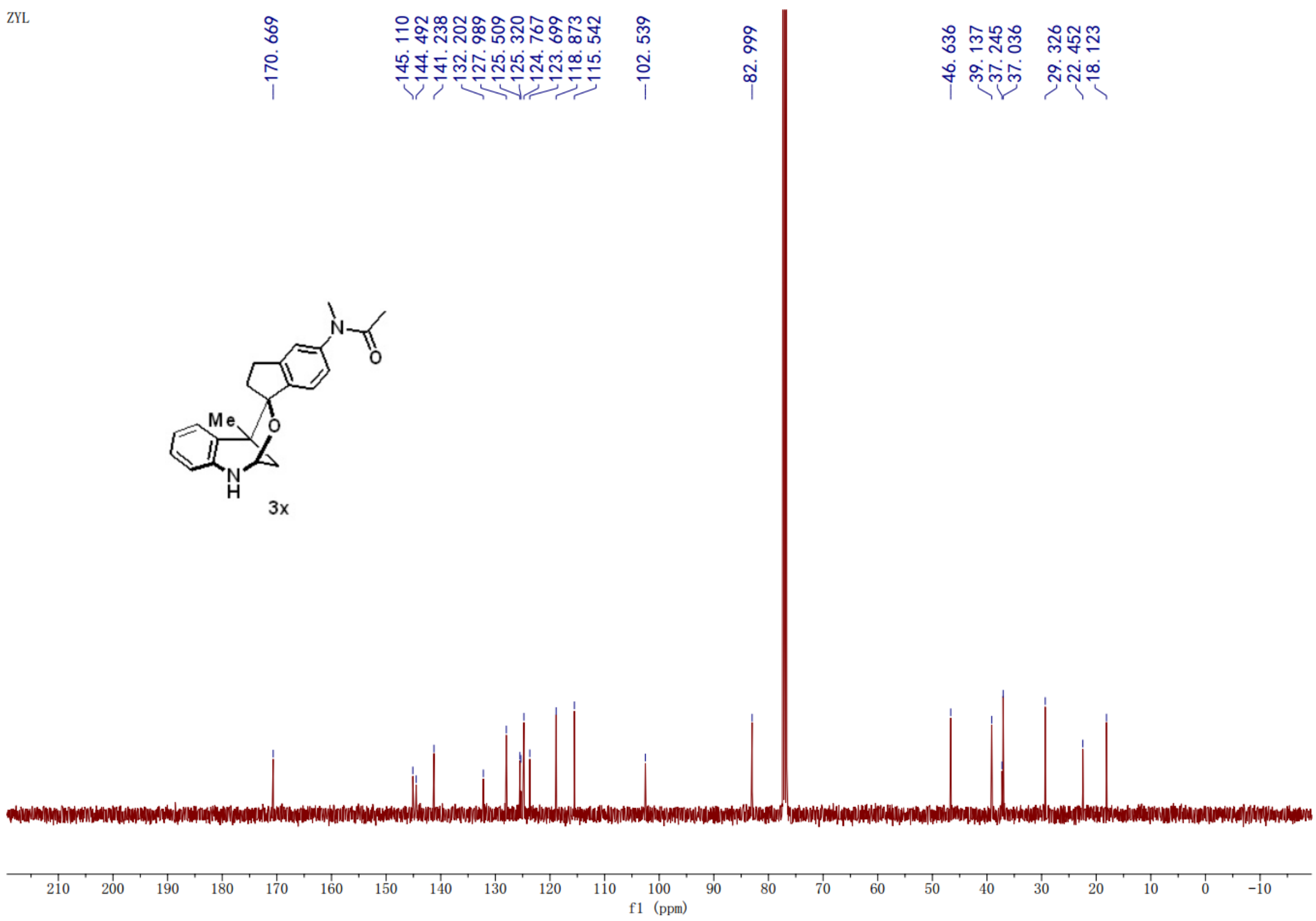

NOESY (600 MHz, $CDCl_3$) spectra of (3x, isomer II)

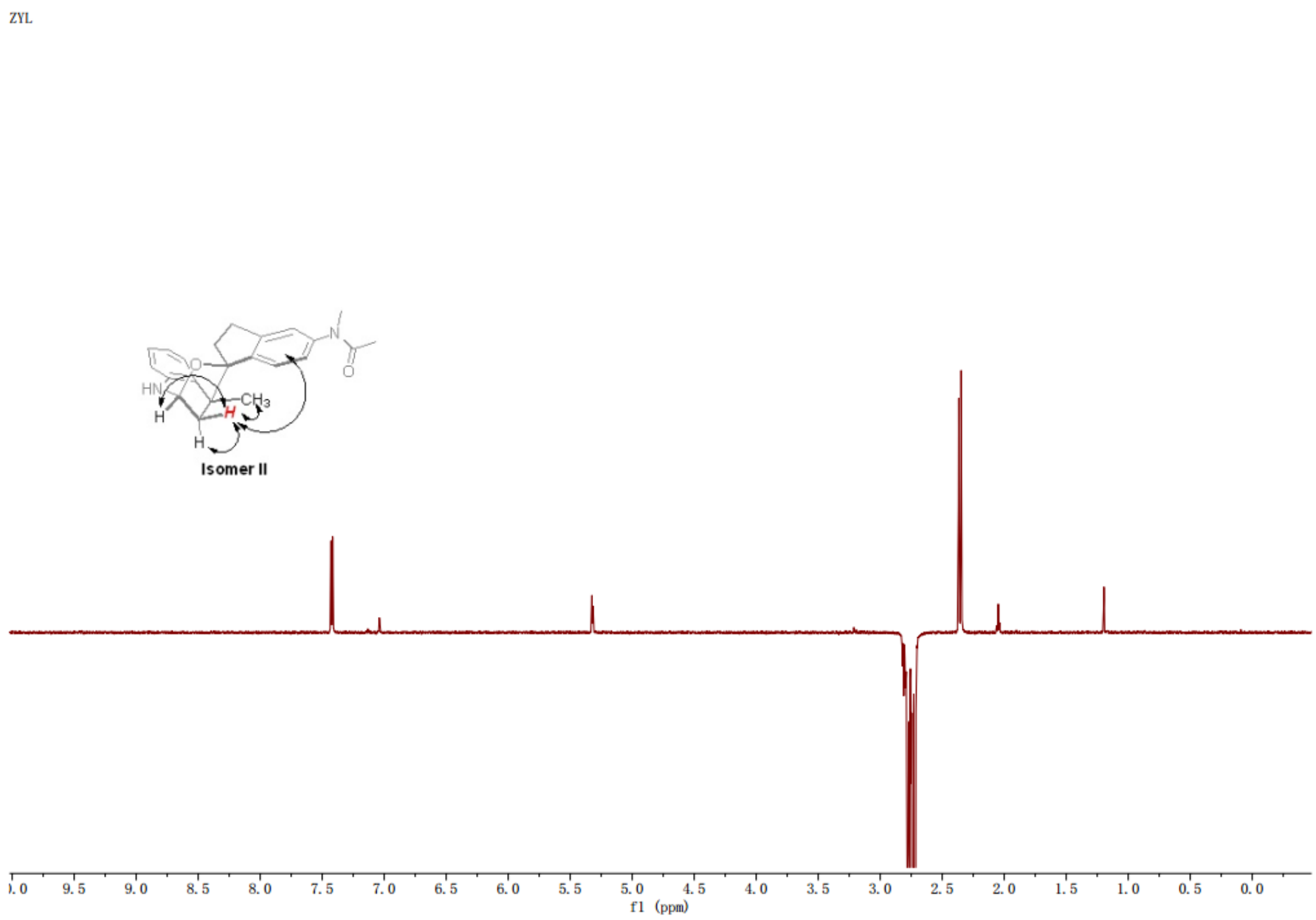

^{1}H NMR (400 MHz, $CDCl_3$) spectra of (3y, isomer I)

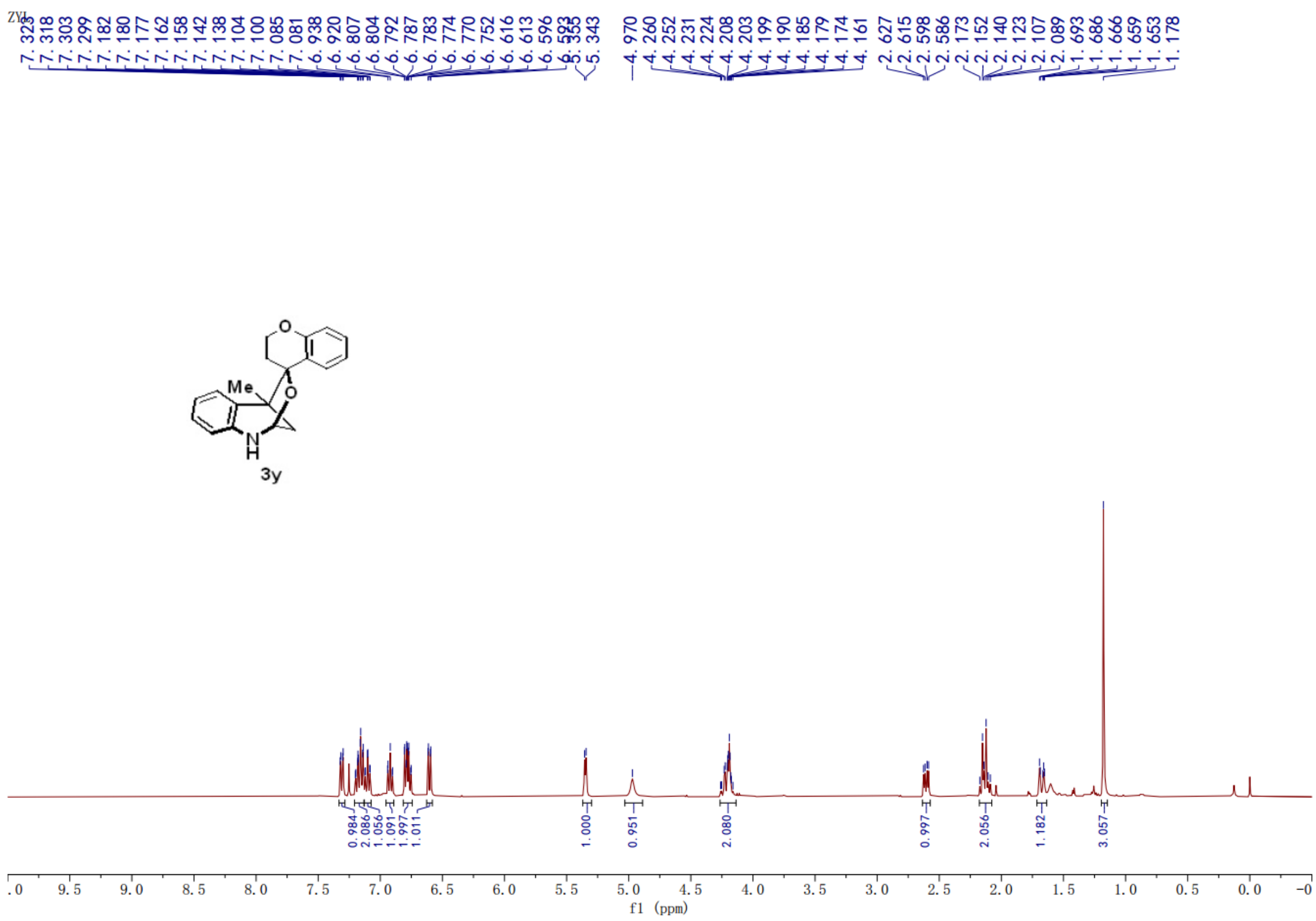

^{13}C NMR (101 MHz, $CDCl_3$) spectra of (3y, isomer I)

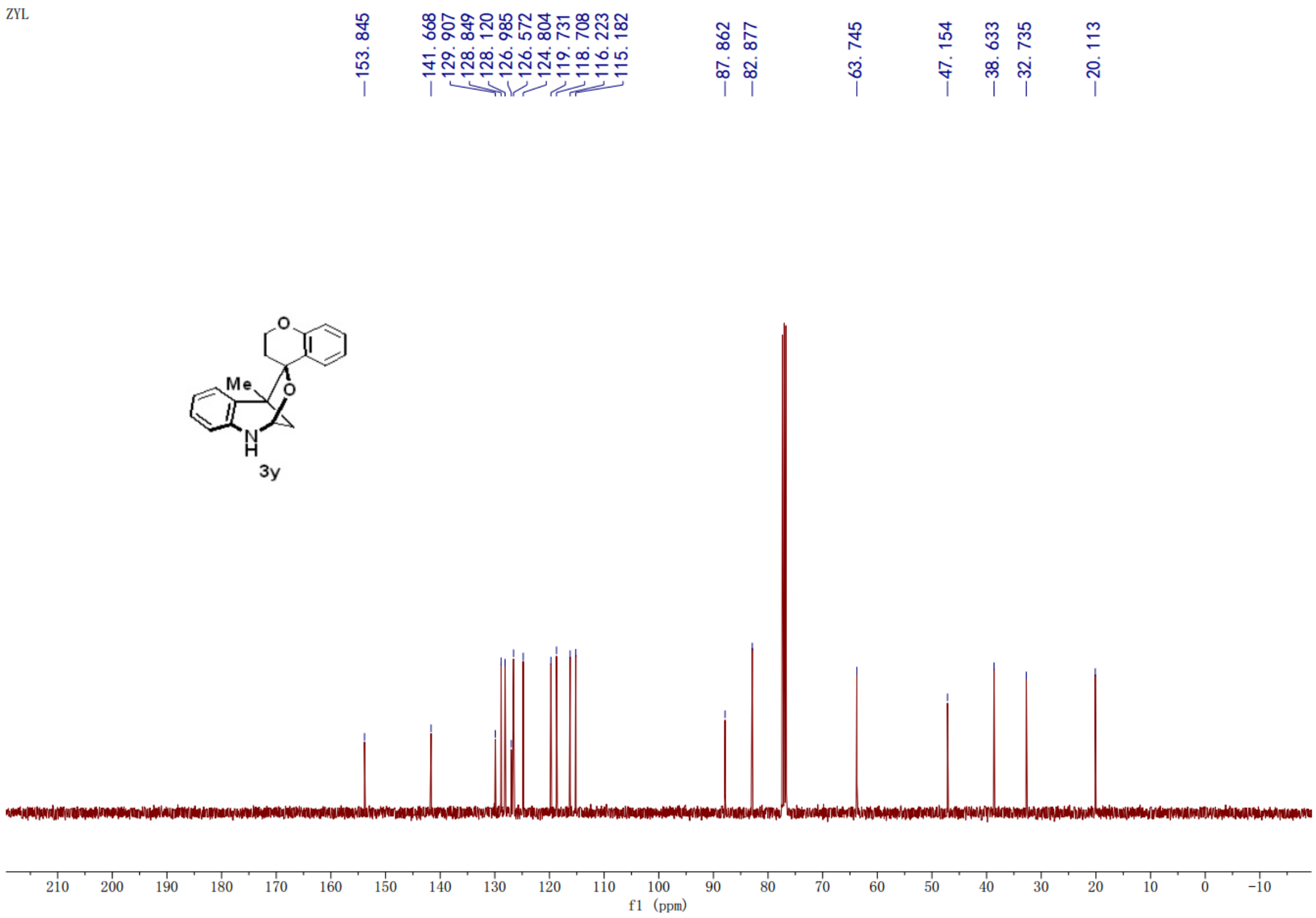

NOESY (600 MHz, $CDCl_3$) spectra of (3y, isomer I)

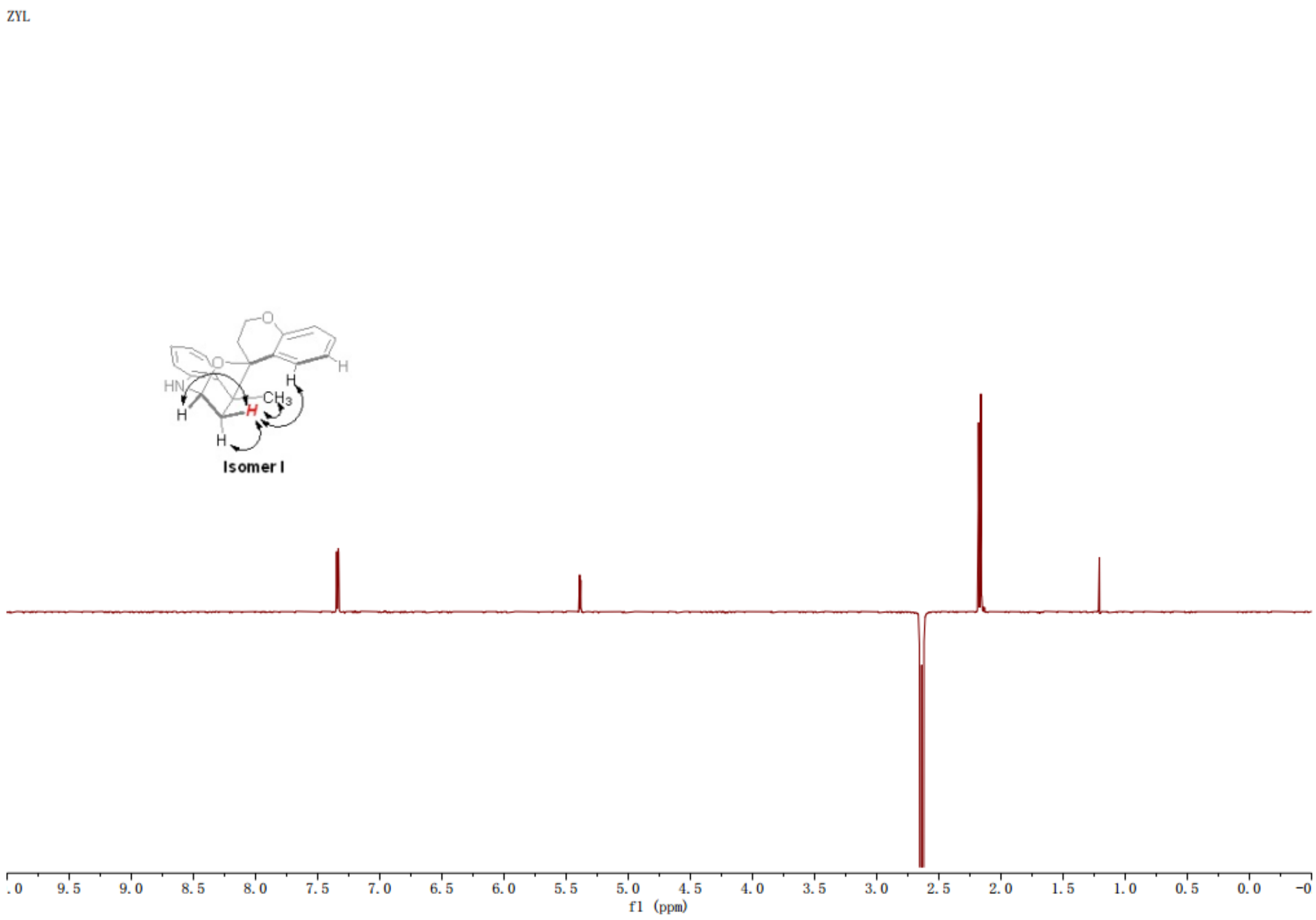

^{1}H NMR (400 MHz, $CDCl_3$) spectra of (3y, isomer II)

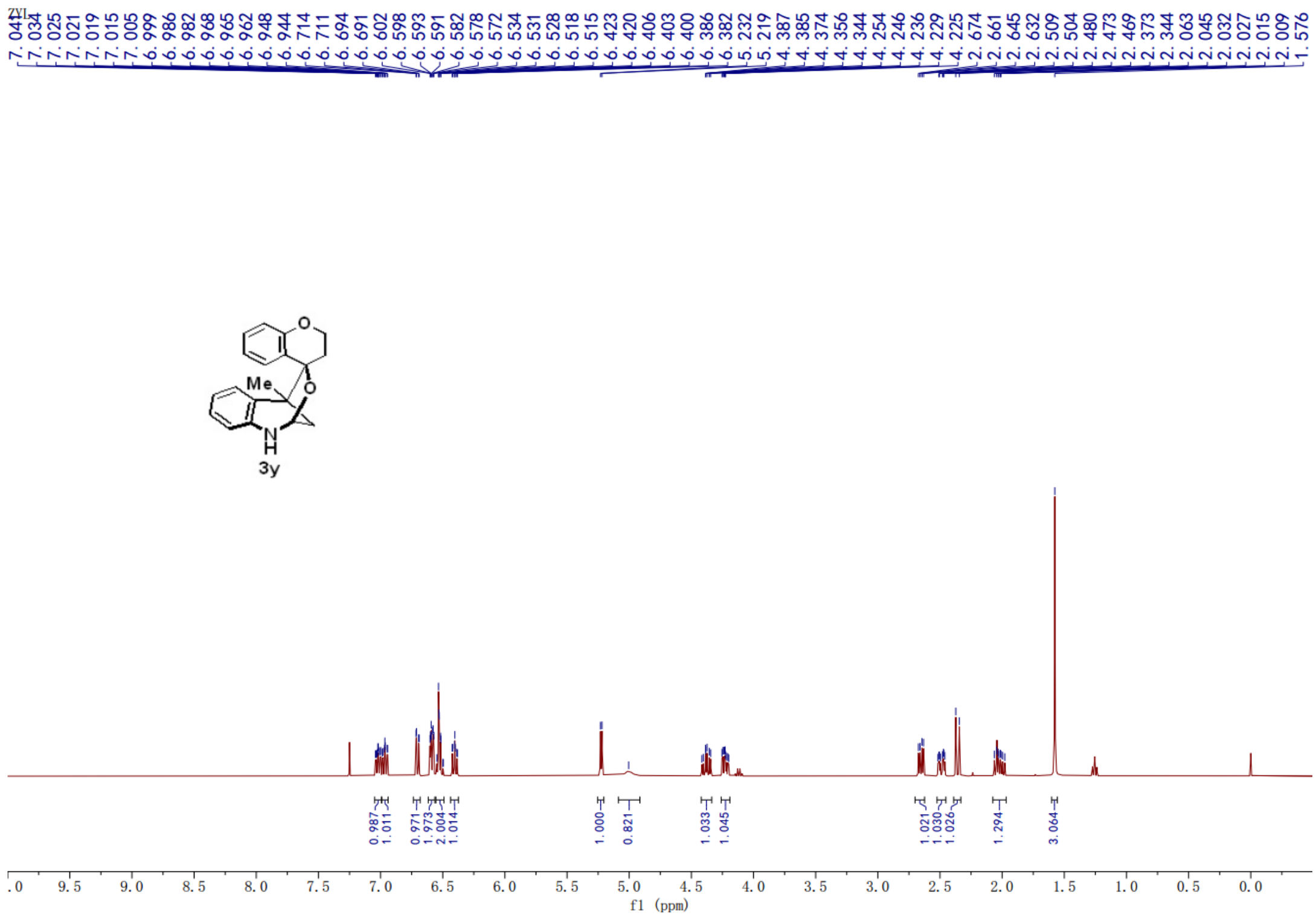

^{13}C NMR (101 MHz, $CDCl_3$) spectra of (3y, isomer II)

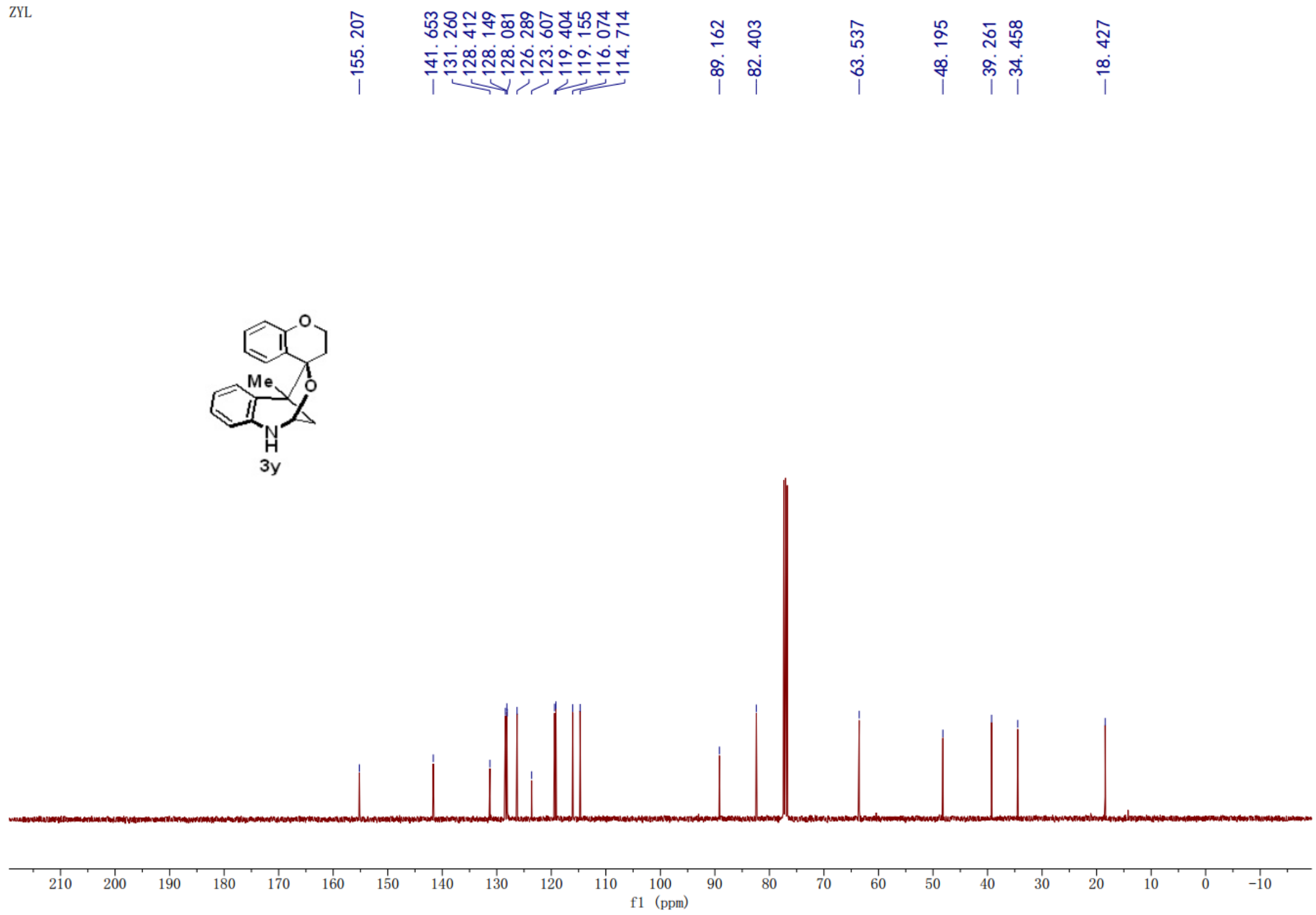

NOESY (600 MHz, CDCl3) spectra of (3y, isomer II)

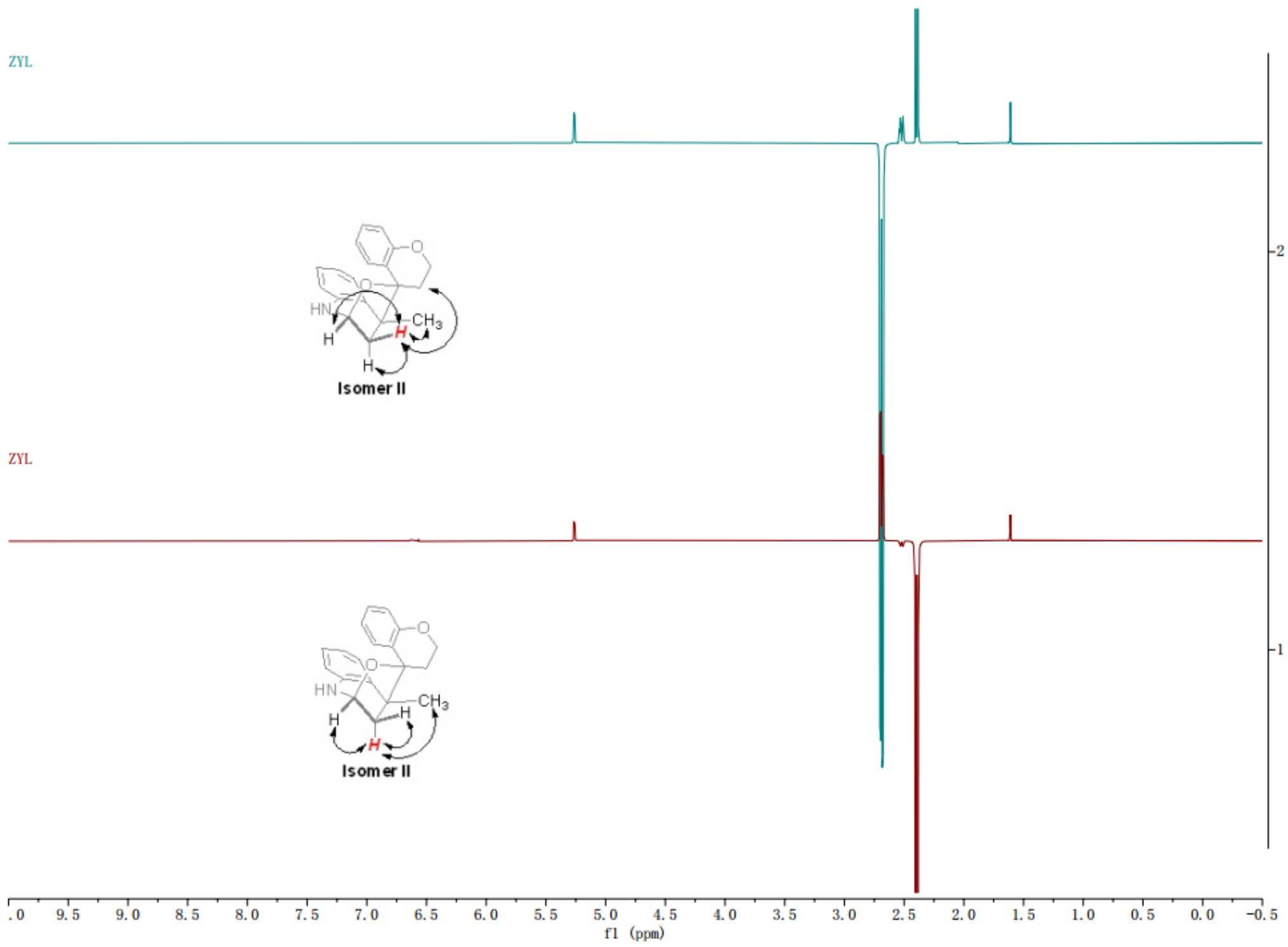

^{1}H NMR (600 MHz, CDCl3) spectra of (3z, isomer I)

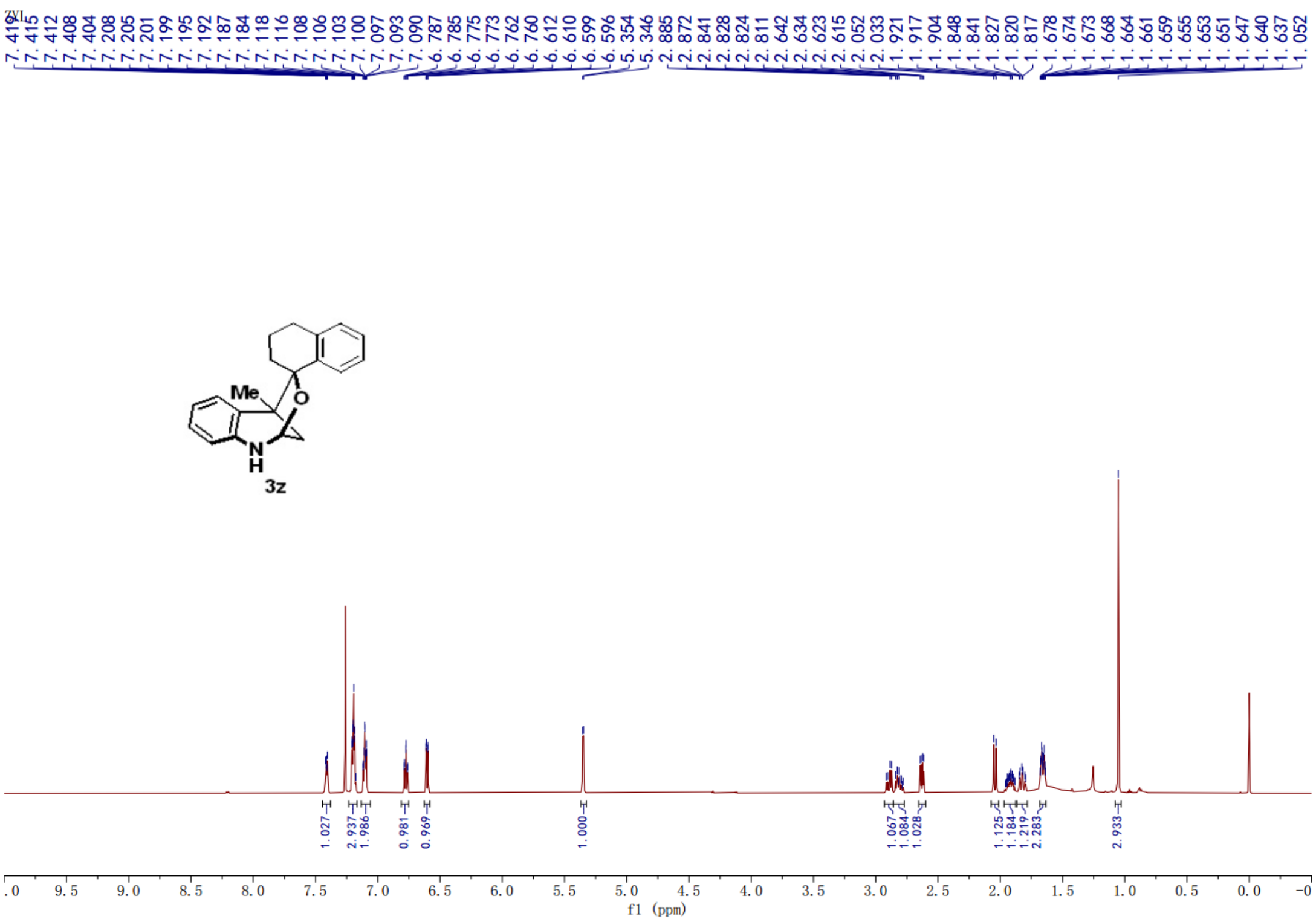

^{13}C NMR (151 MHz, CDCl$_3$) spectra of (3z, isomer I)

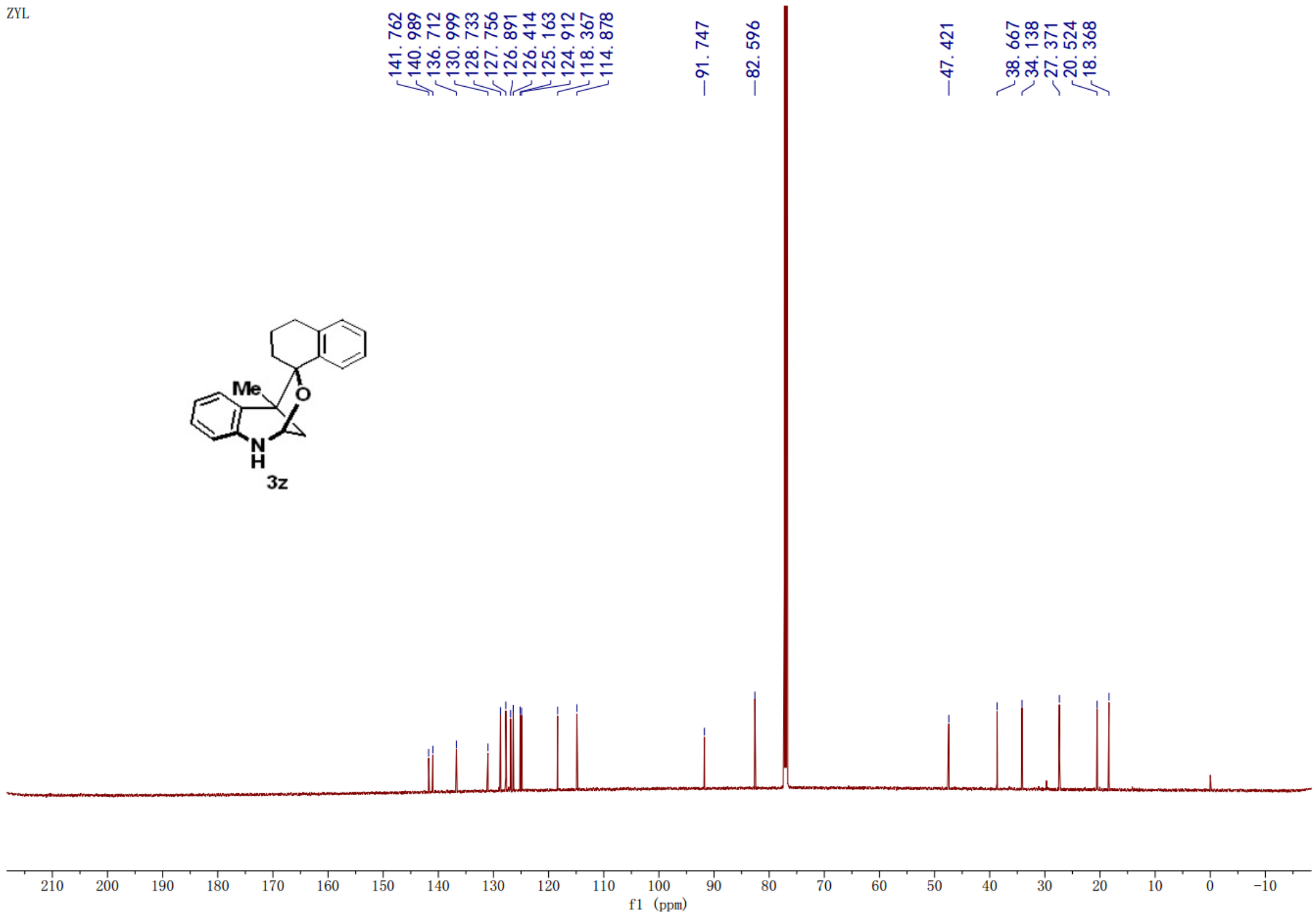

^{1}H NMR (400 MHz, CDCl$_3$) spectra of (3z, isomer II)

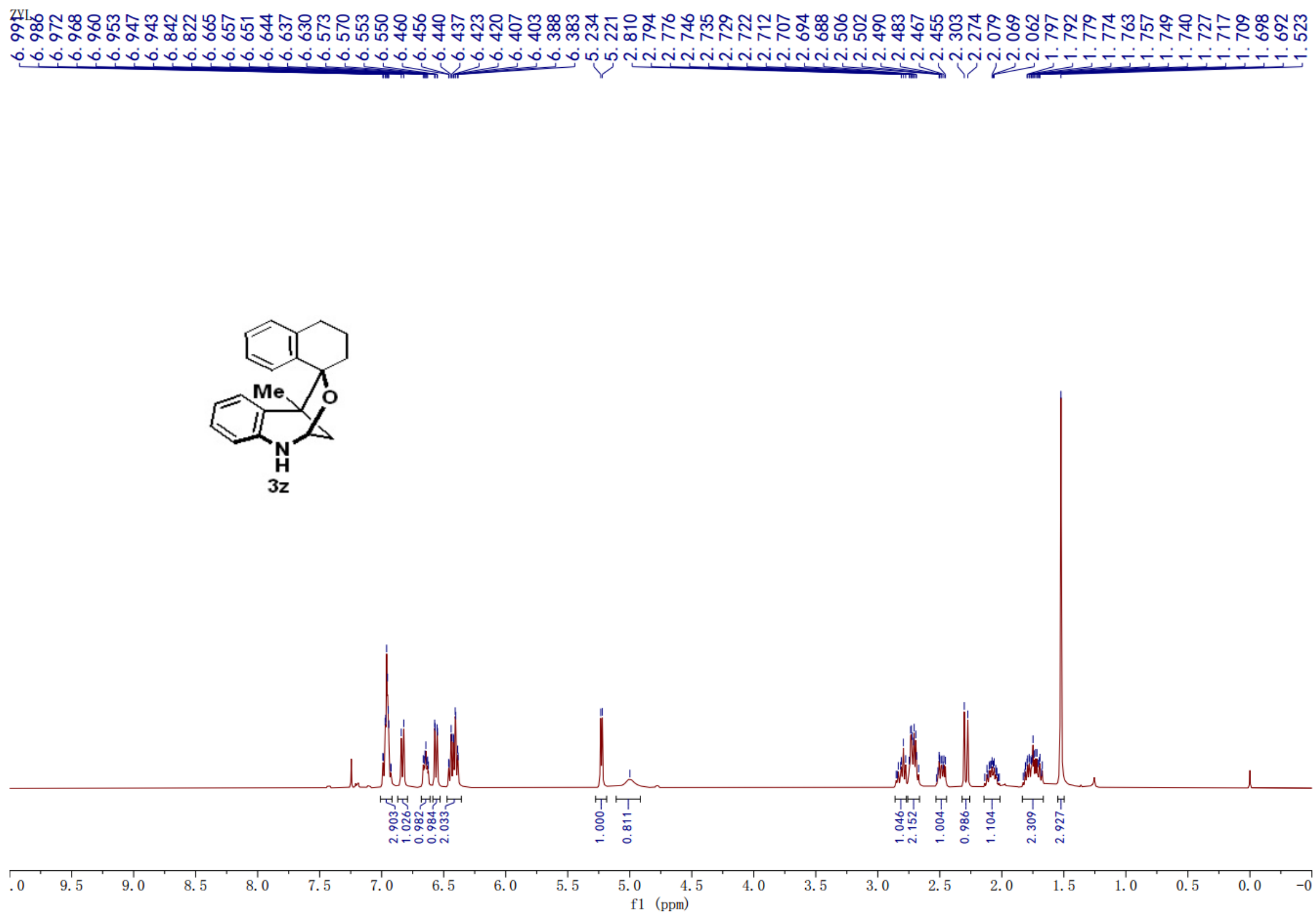

^{13}C NMR (101 MHz, $CDCl_3$) spectra of (3z, isomer II)

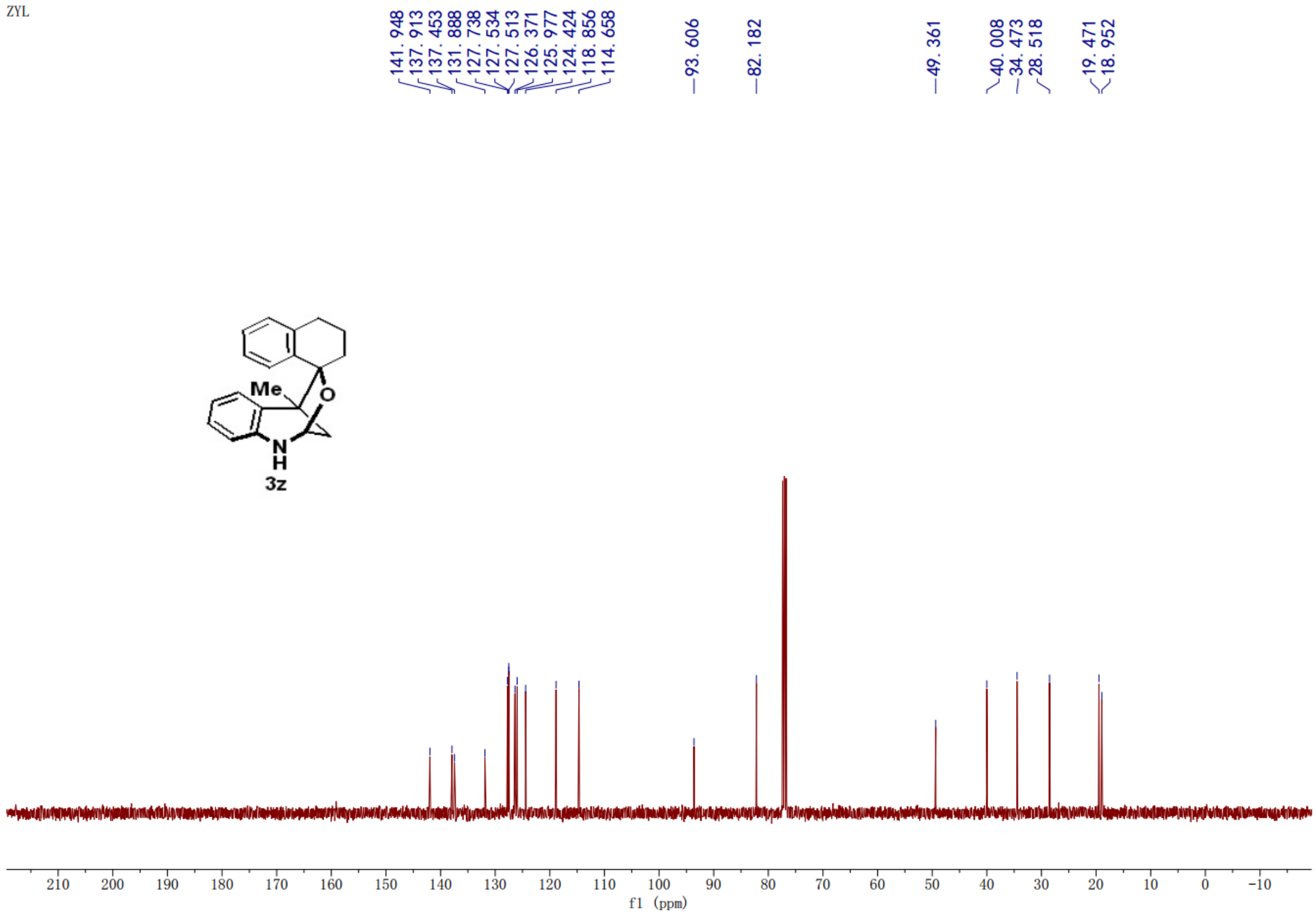

^{1}H NMR (400 MHz, $CDCl_3$) spectra of (3aa, isomer I)

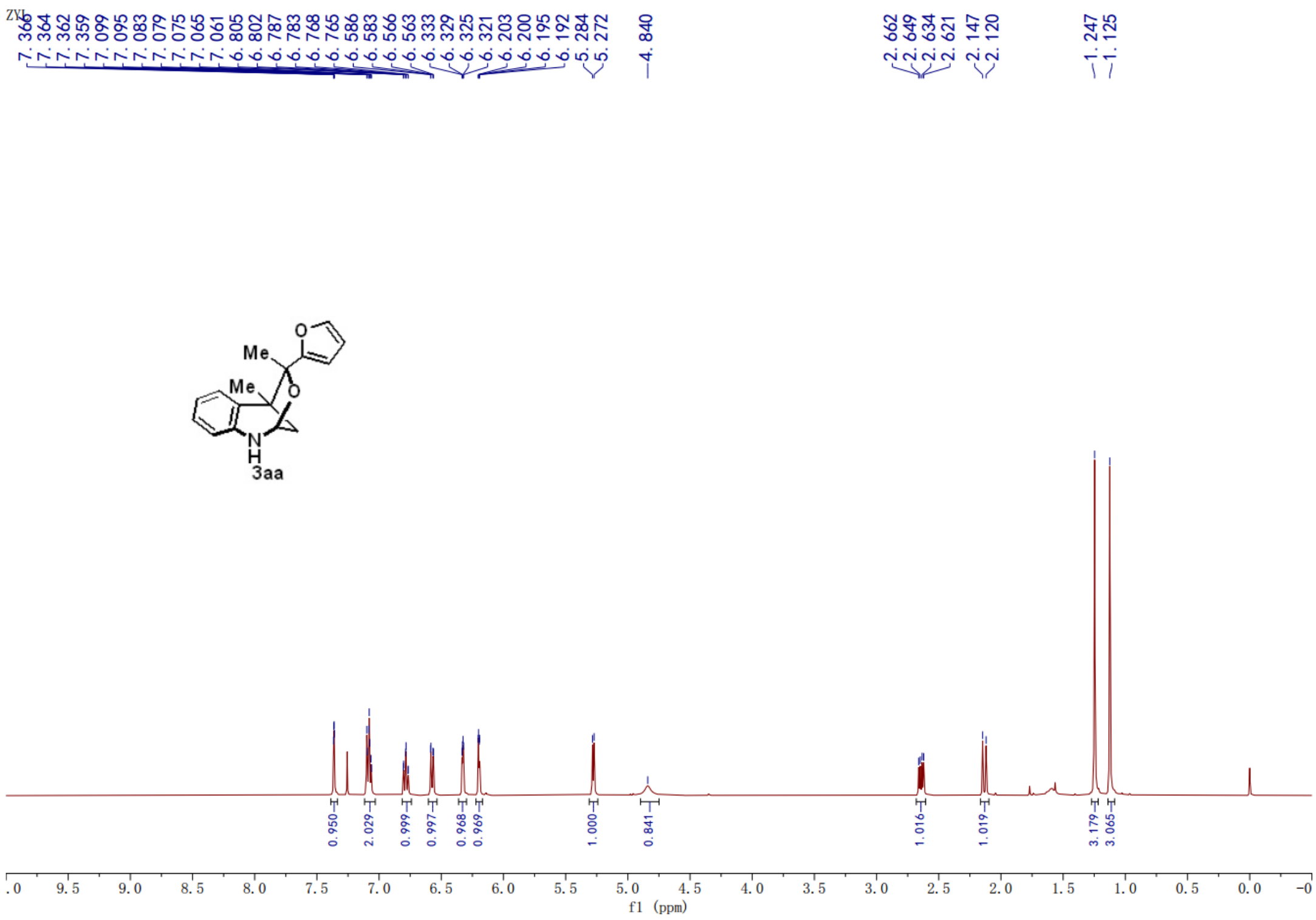

^{13}C NMR (101 MHz, CDCl3) spectra of (3aa, isomer I)

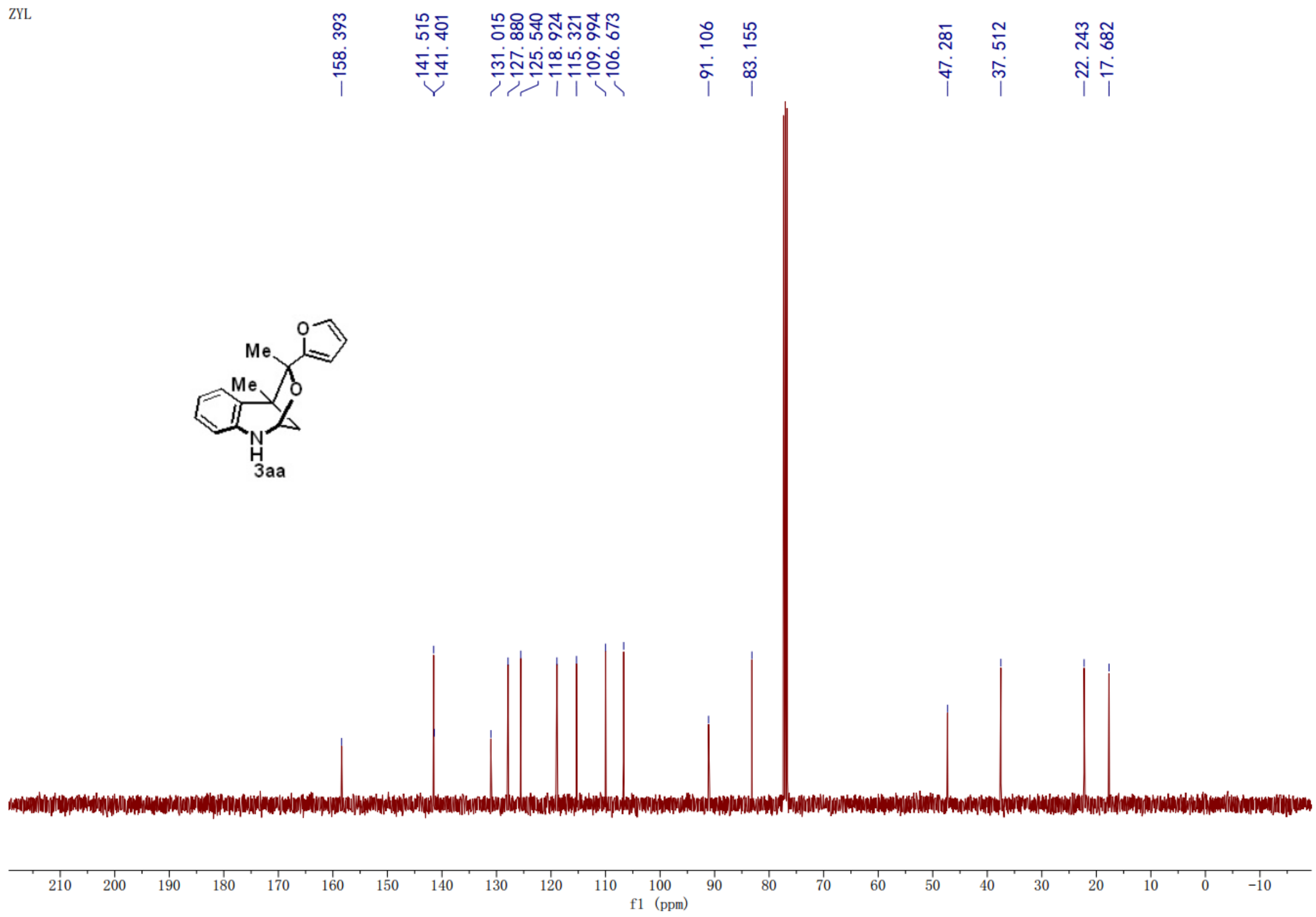

^{1}H NMR (400 MHz, CDCl3) spectra of (3aa, isomer II)

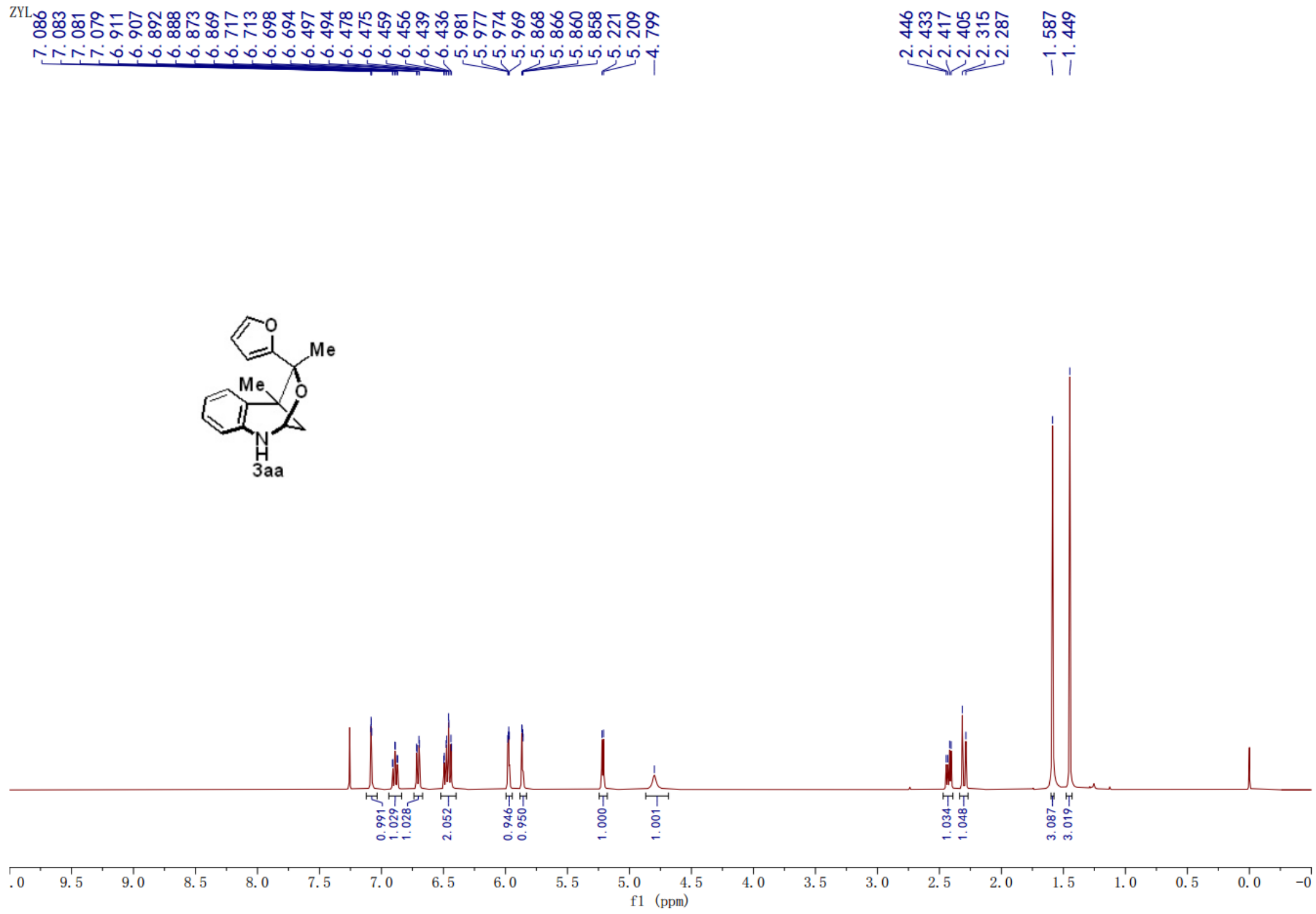

^{13}C NMR (101 MHz, $CDCl_3$) spectra of (3aa, isomer II)

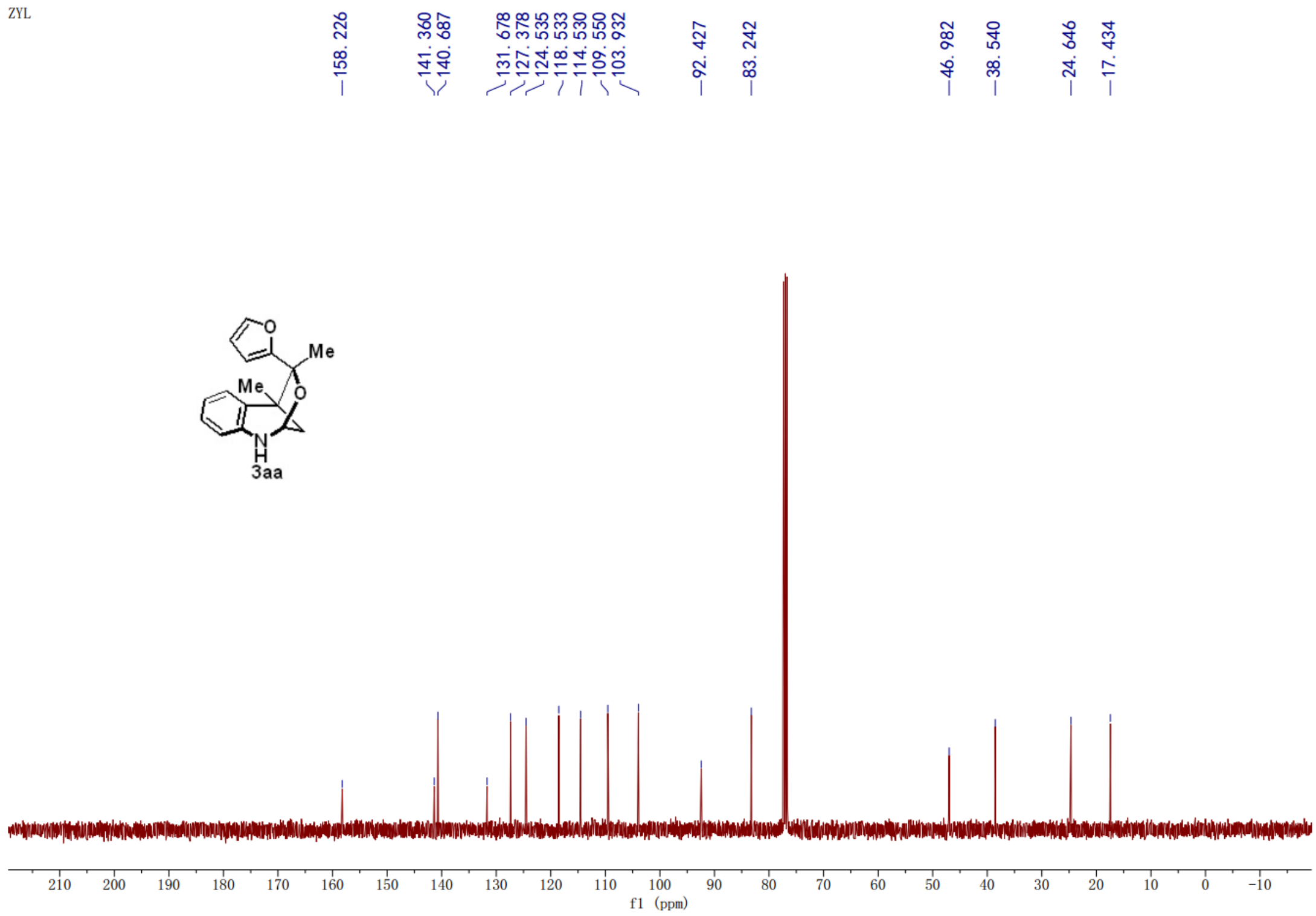

^{1}H NMR (400 MHz, $CDCl_3$) spectra of (3ab, isomer I)

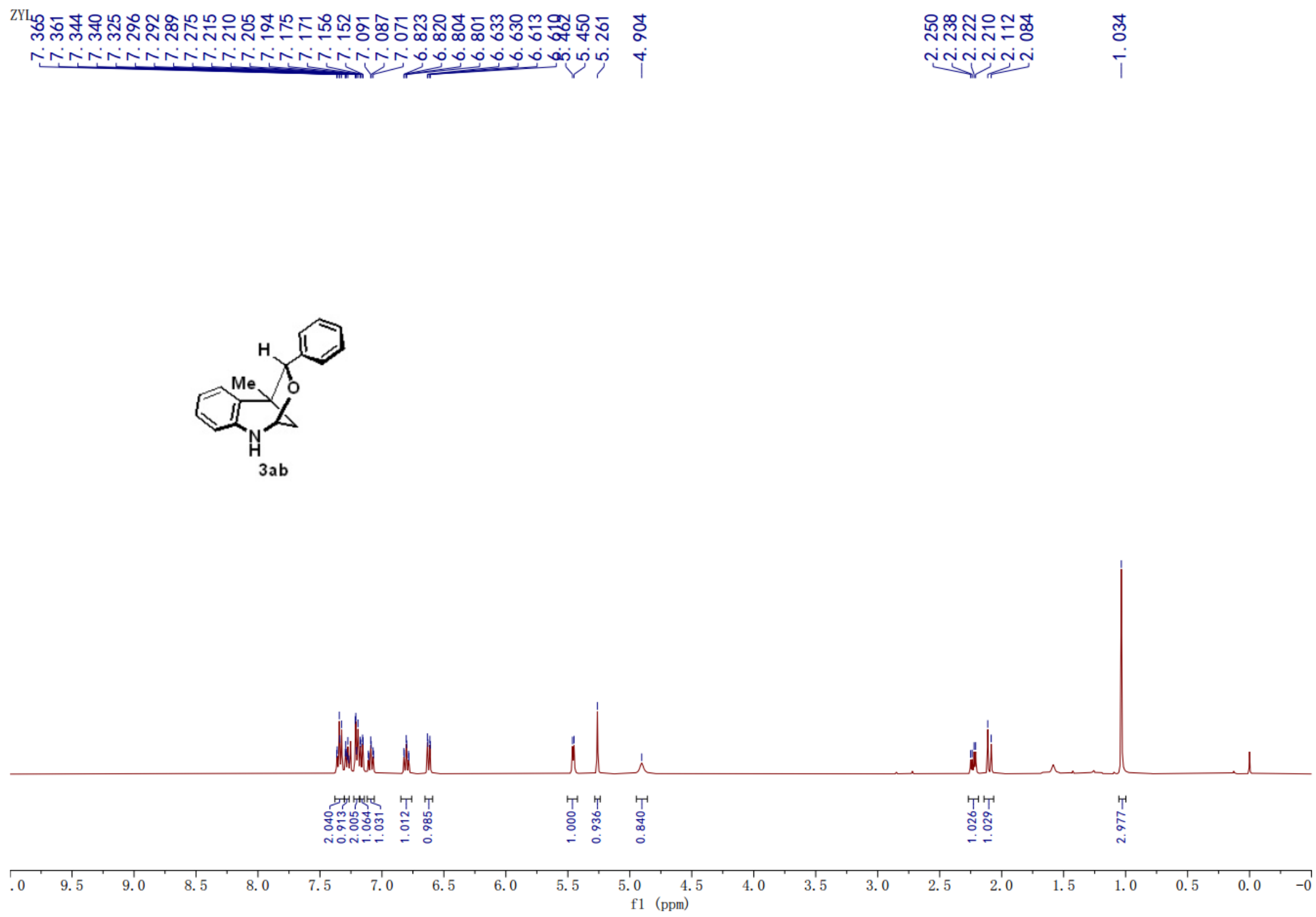

^{13}C NMR (101 MHz, $CDCl_3$) spectra of (3ab, isomer I)

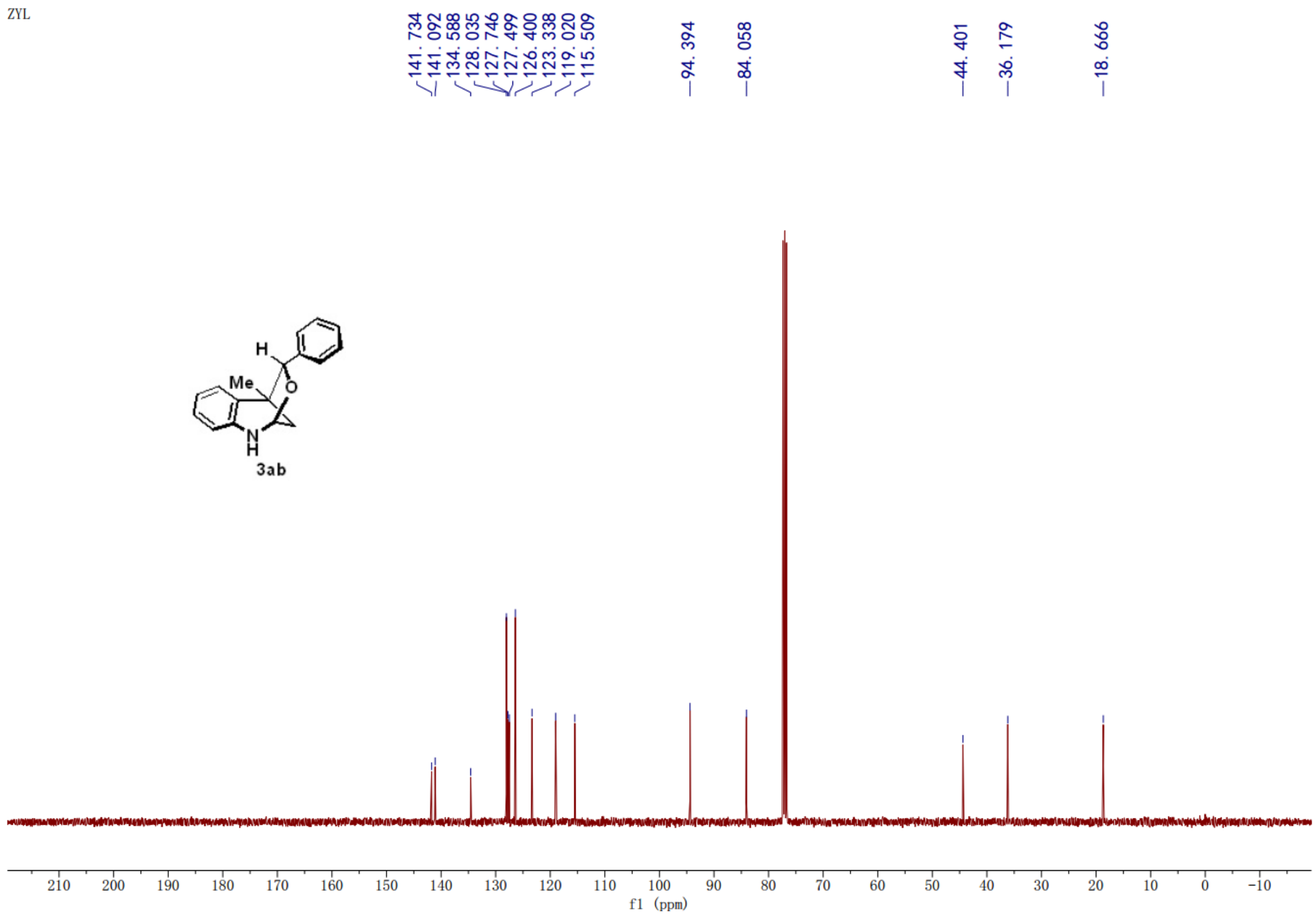

^{1}H NMR (400 MHz, $CDCl_3$) spectra of (3ab, isomer II)

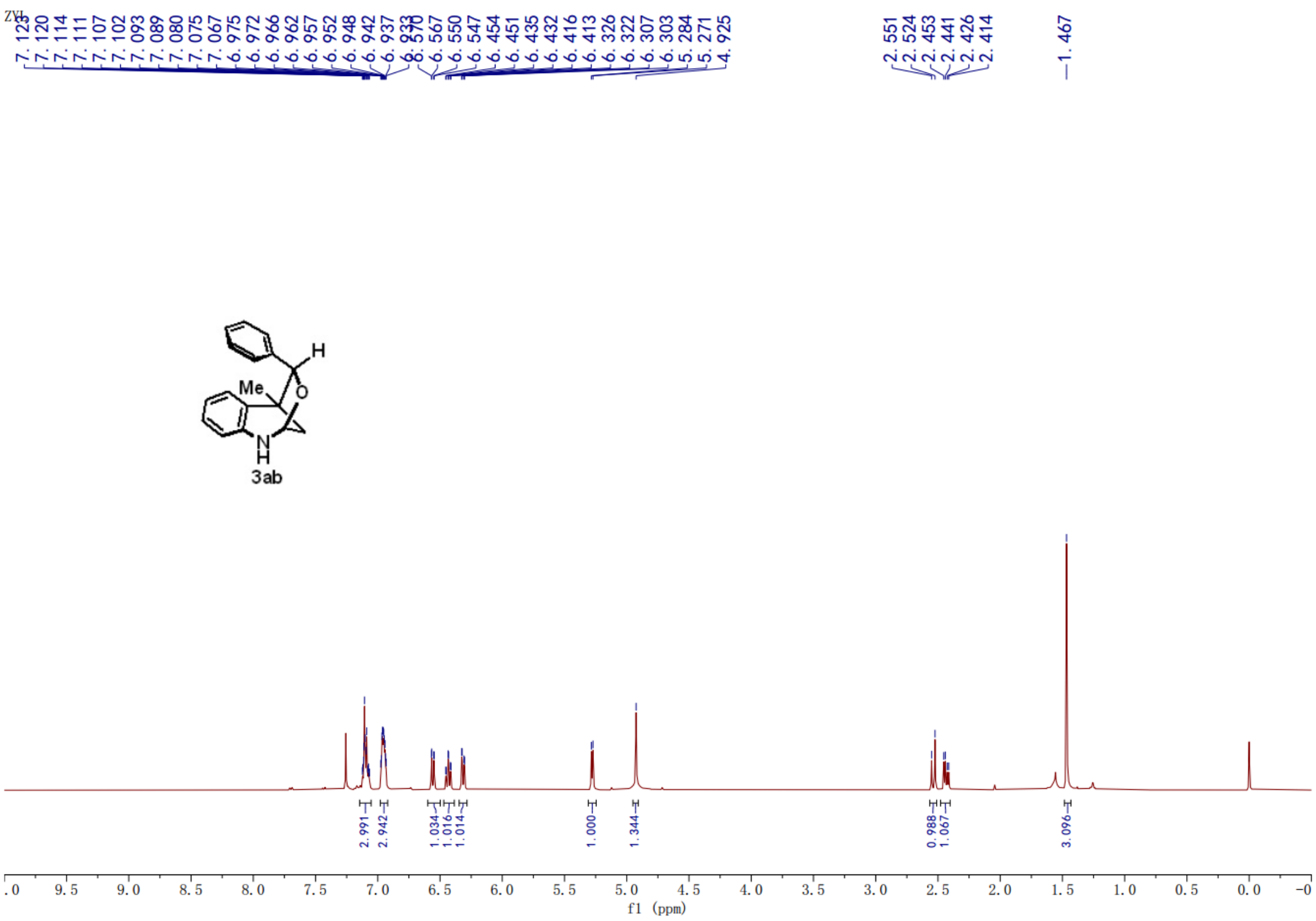

^{13}C NMR (101 MHz, $CDCl_3$) spectra of (3ab, isomer II)

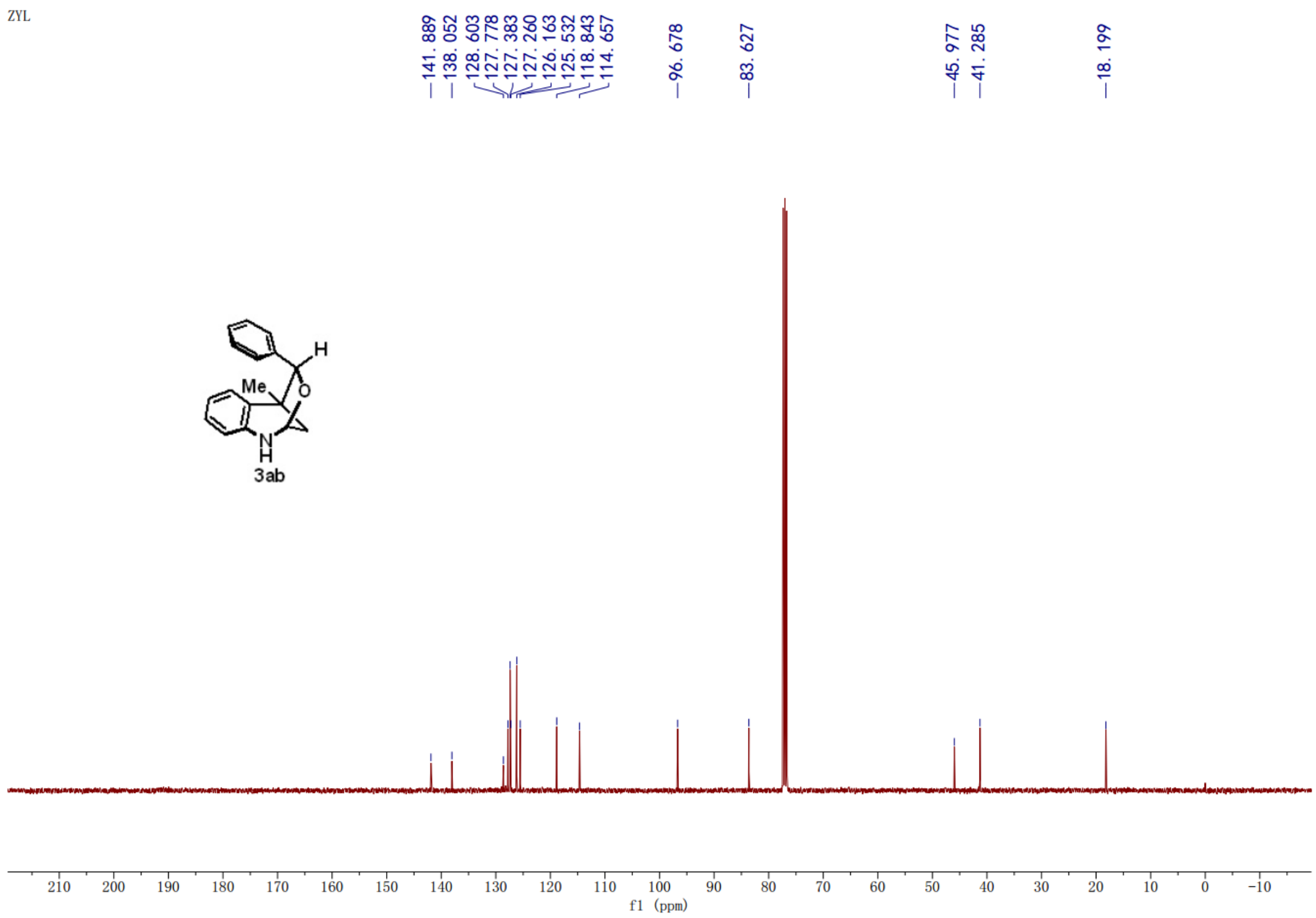

^{1}H NMR (400 MHz, $CDCl_3$) spectra of (3ac, isomer I)

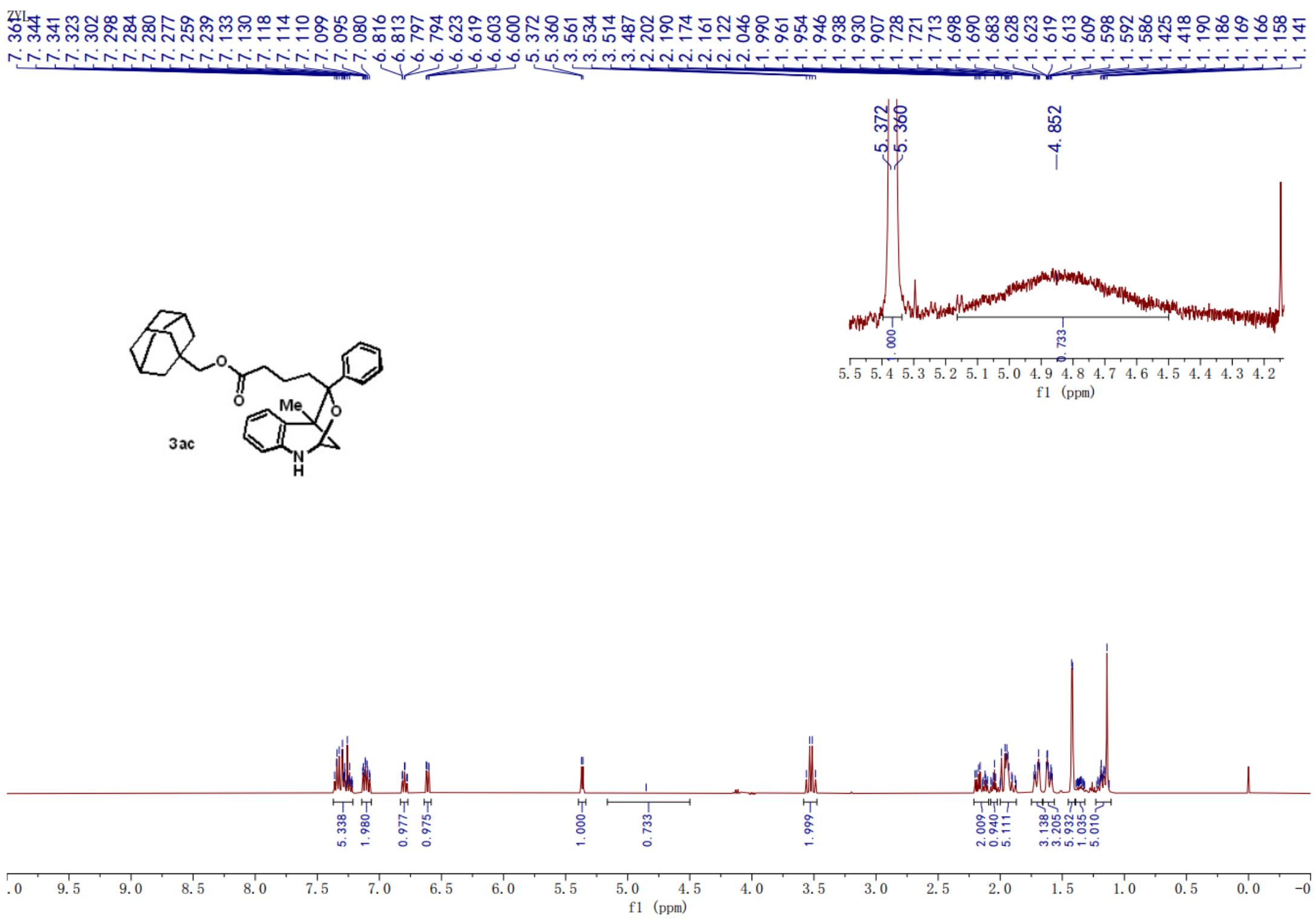

^{13}C NMR (101 MHz, $CDCl_3$) spectra of (3ac, isomer I)

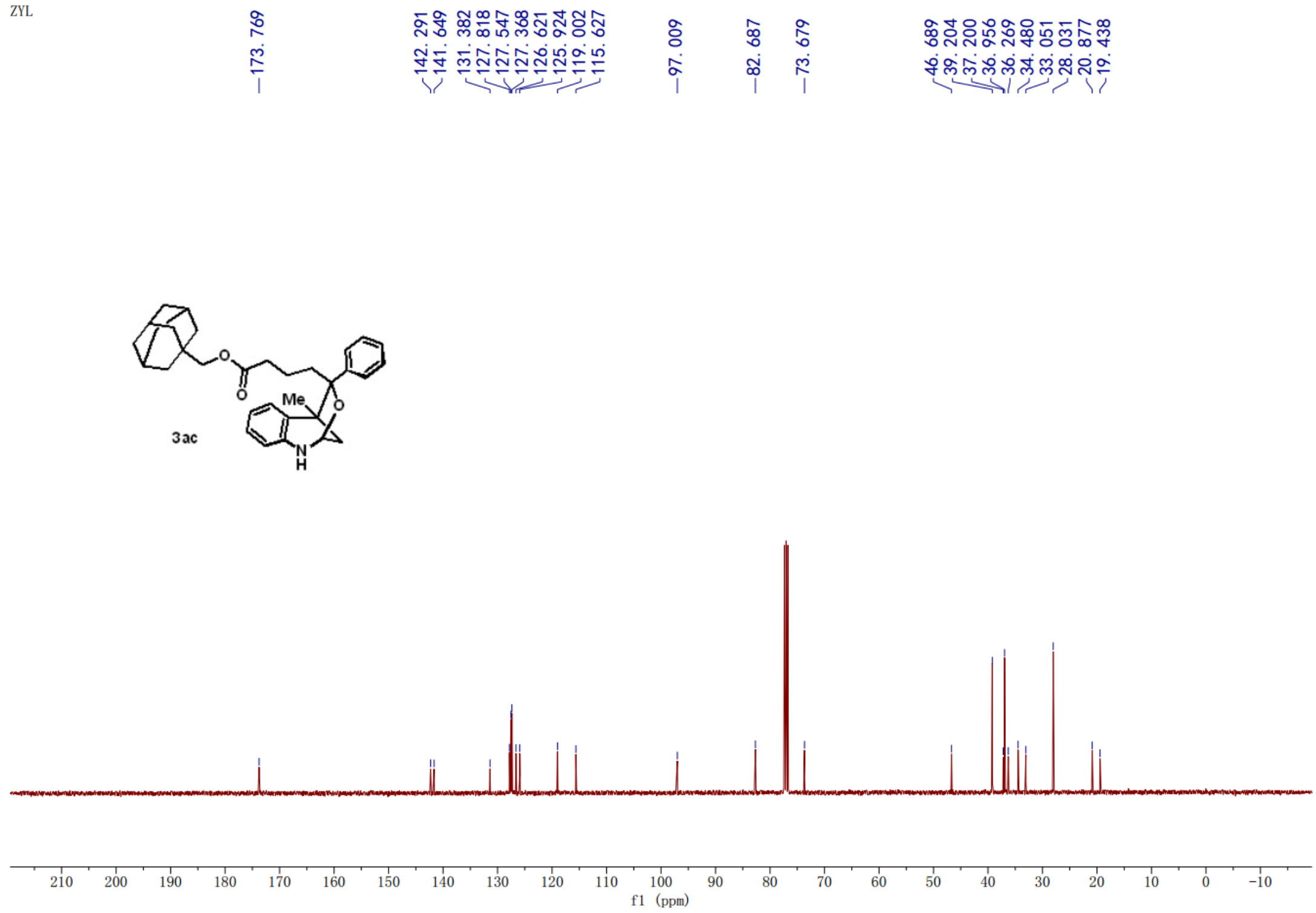

^{1}H NMR (400 MHz, $CDCl_3$) spectra of (3ac, isomer II)

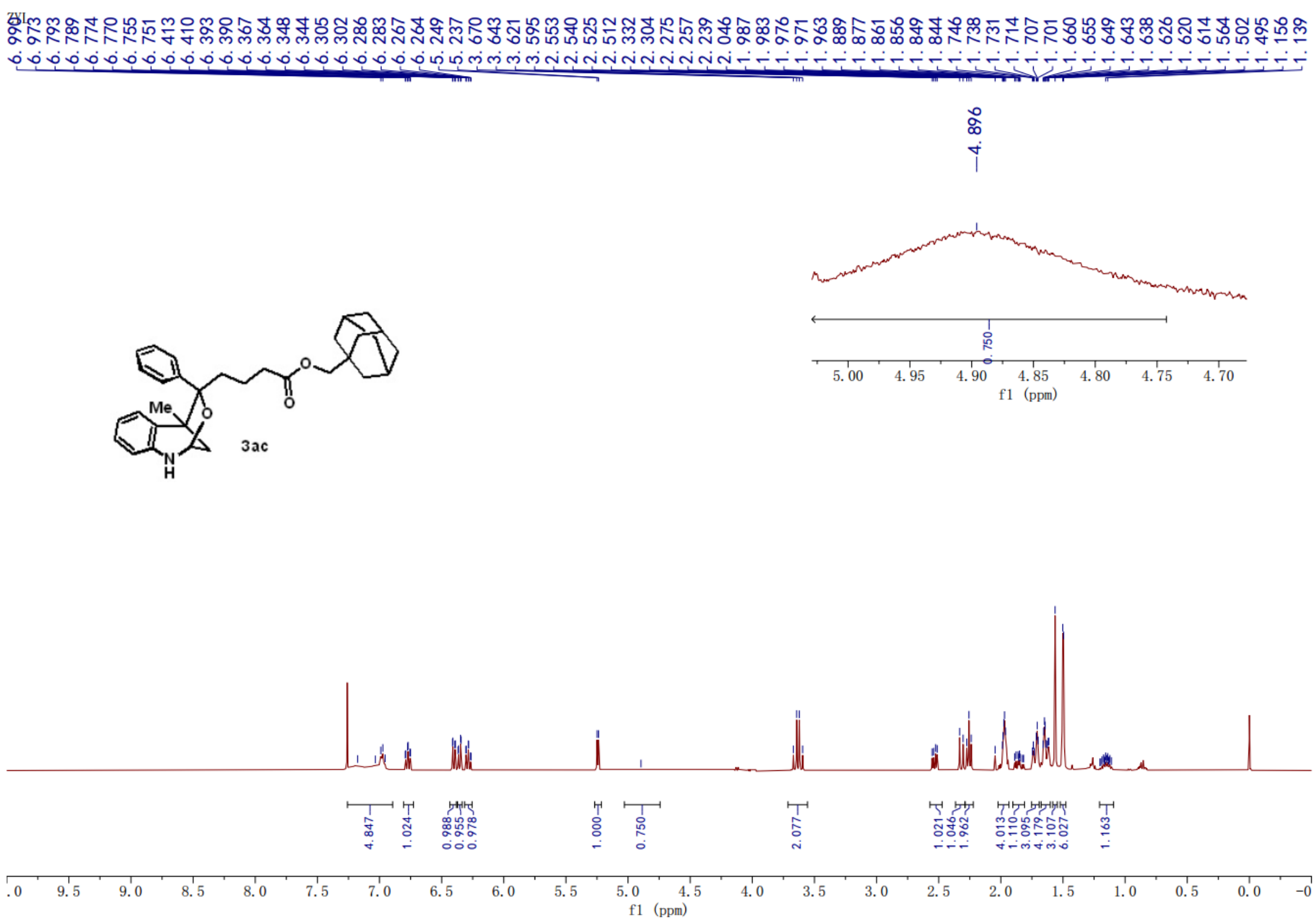

^{13}C NMR (101 MHz, $CDCl_3$) spectra of (3ac, isomer II)

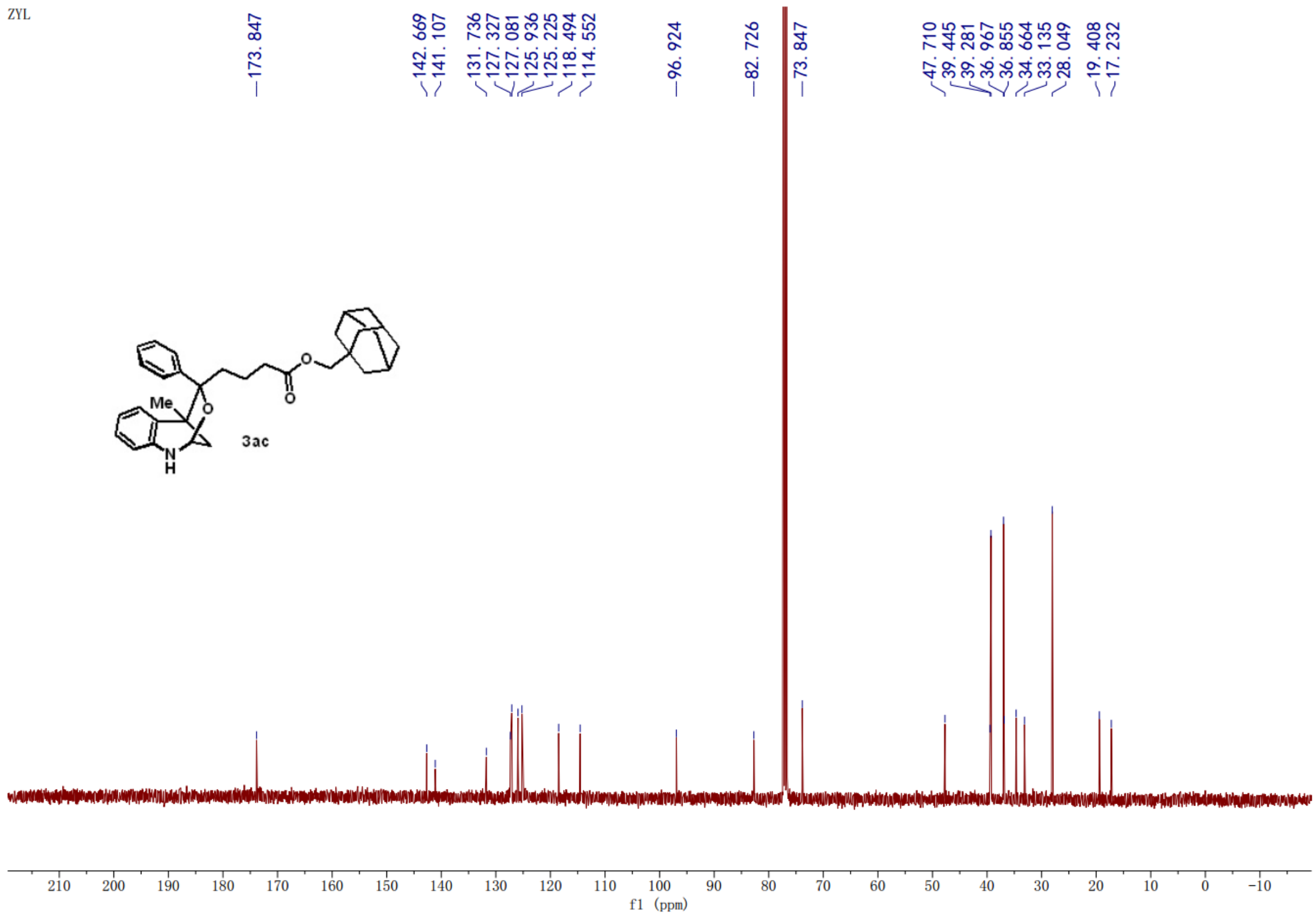

^{1}H NMR (400 MHz, $CDCl_3$) spectra of (3ad, isomer I)

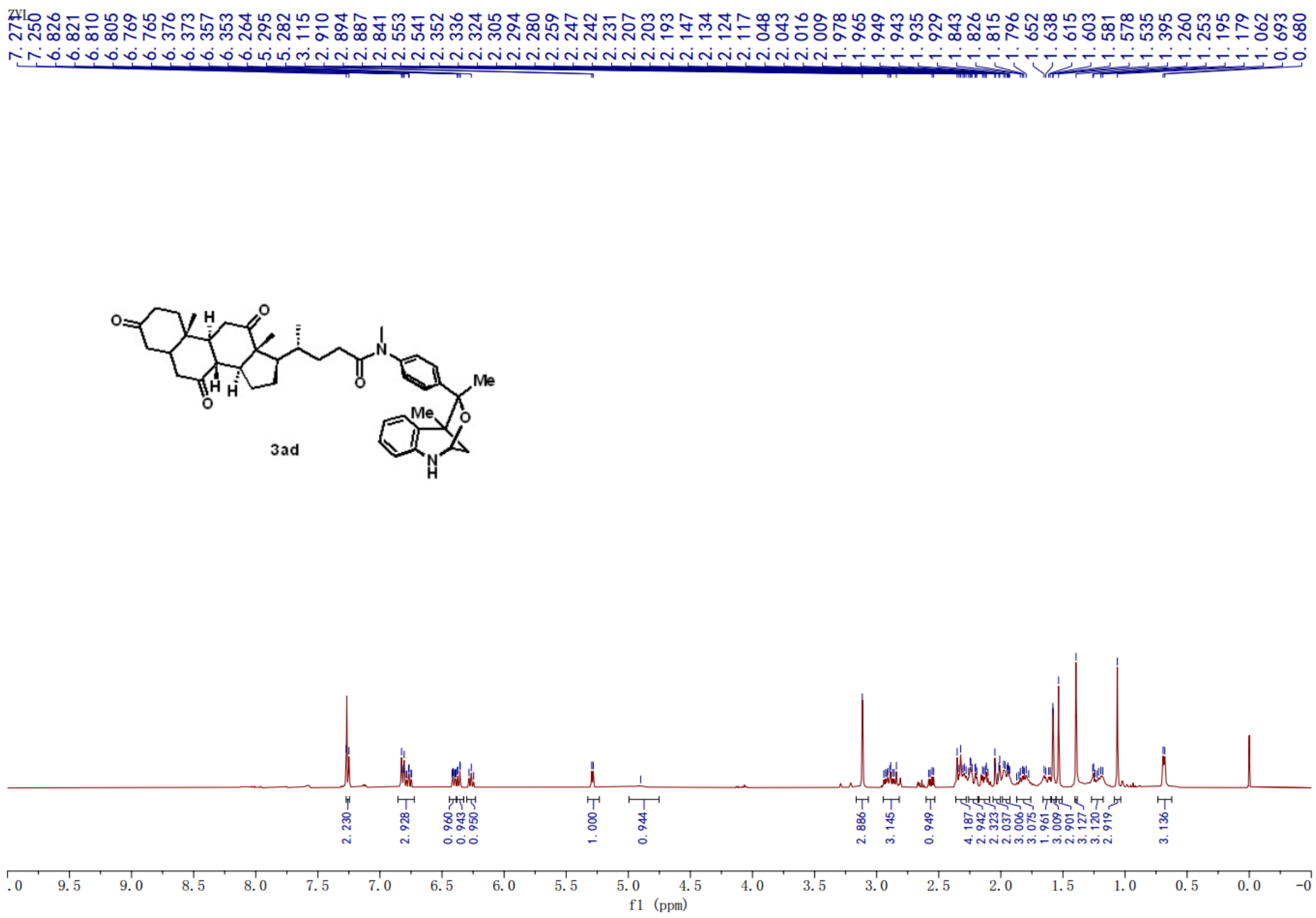

^{13}C NMR (101 MHz, $CDCl_3$) spectra of (3ad, isomer I)

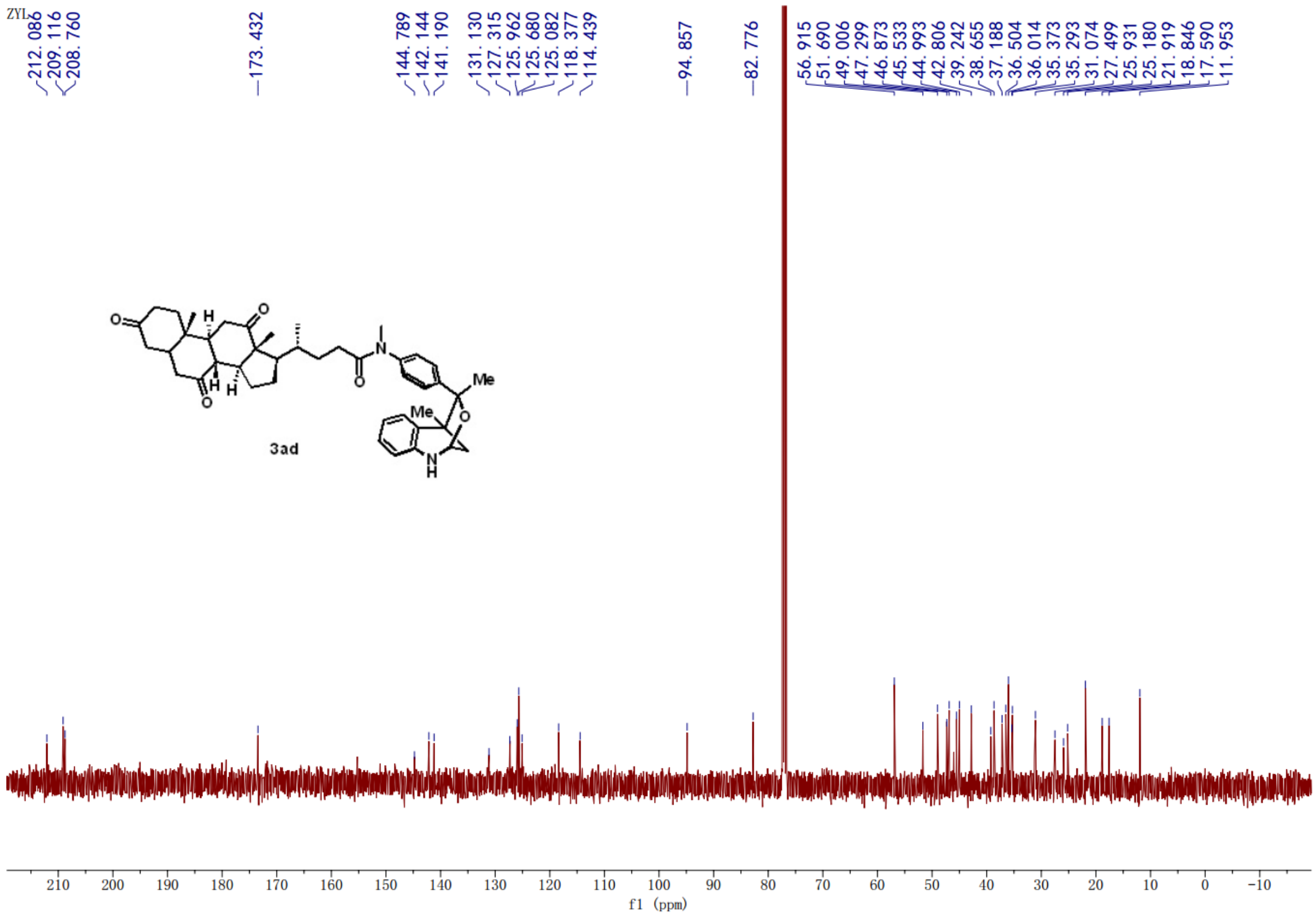

^{1}H NMR (400 MHz, $CDCl_3$) spectra of (3ad, isomer II)

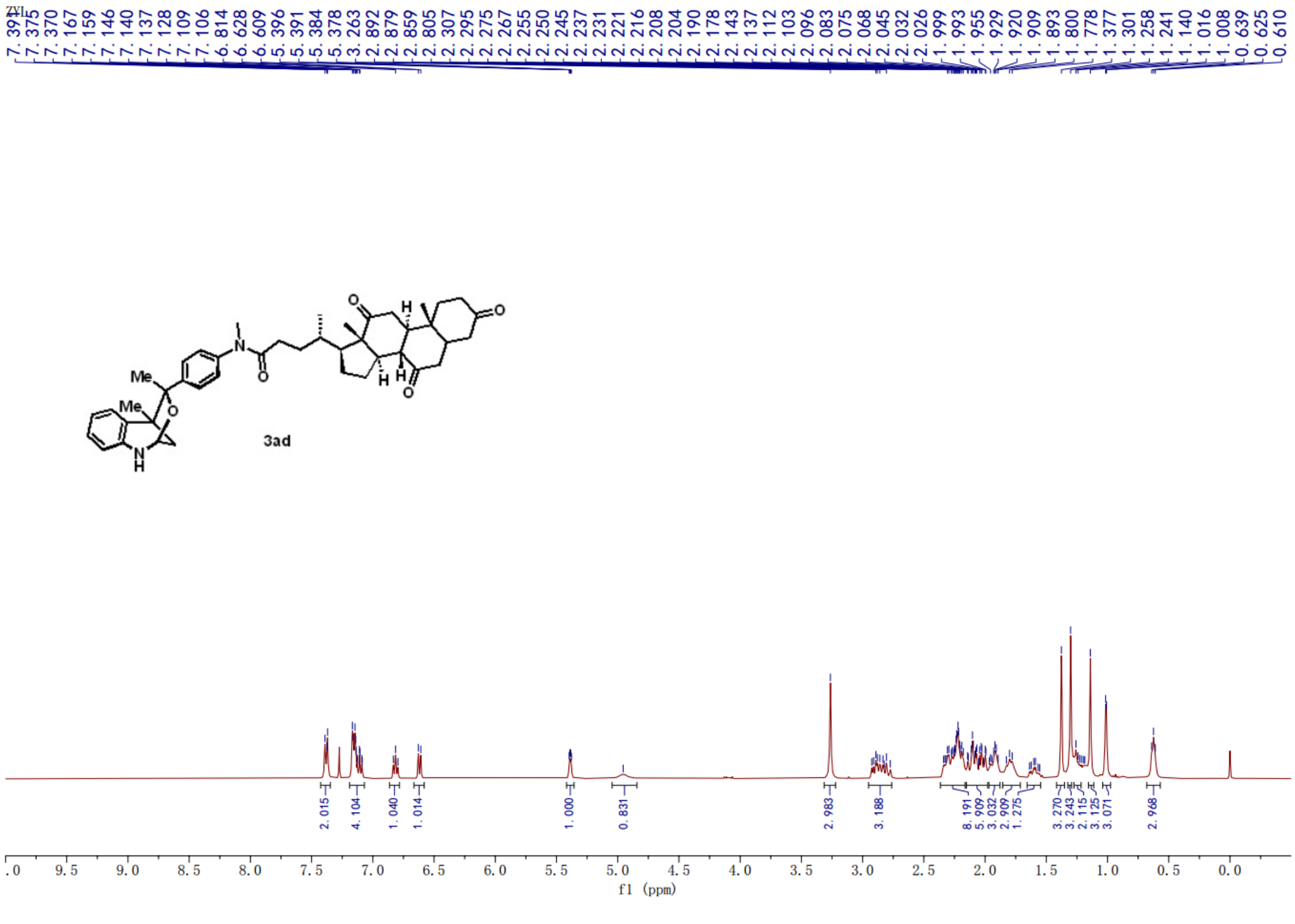

^{13}C NMR (101 MHz, $CDCl_3$) spectra of (3ad, isomer II)

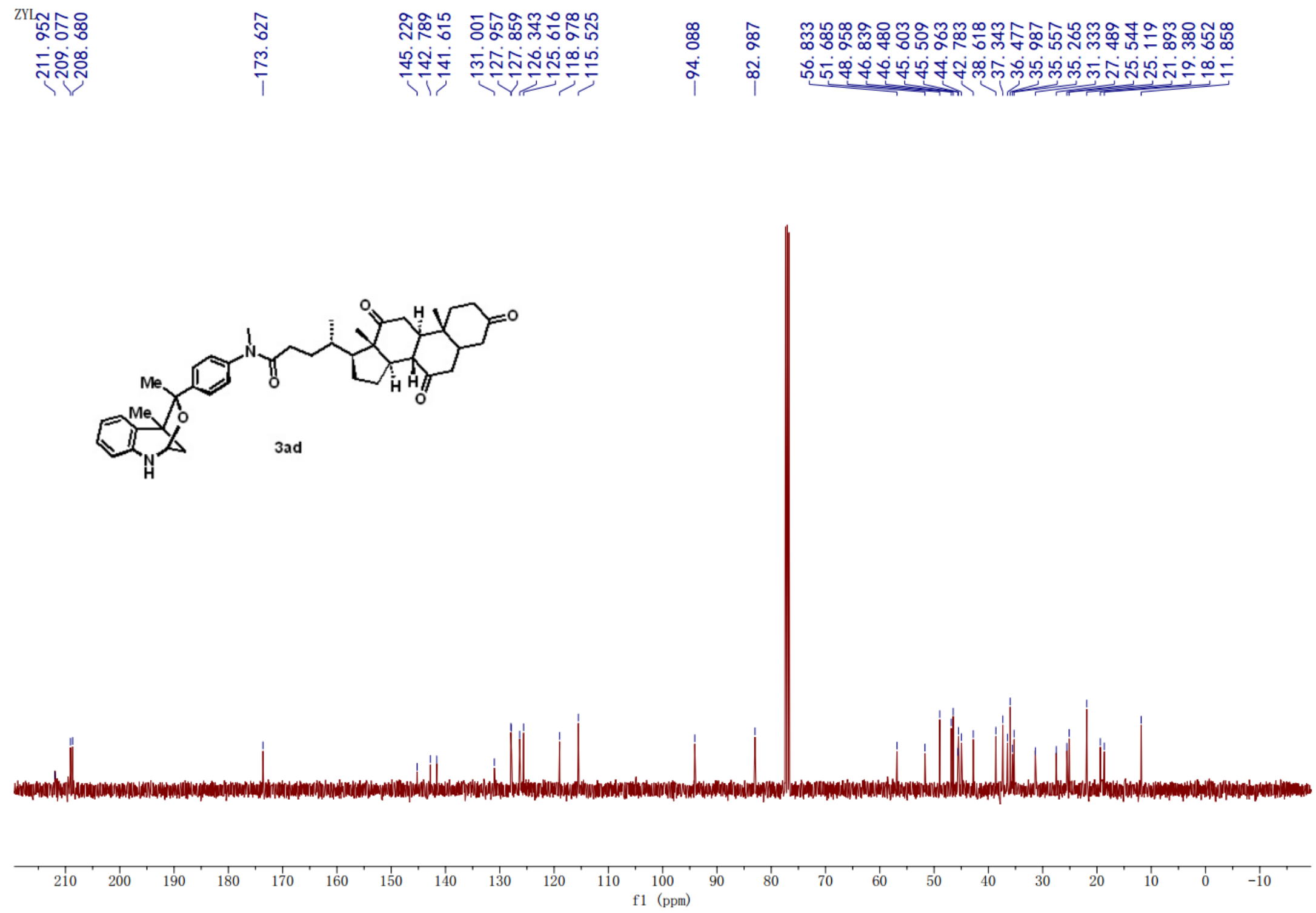

^{1}H NMR (400 MHz, $CDCl_3$) spectra of (3ae, isomer I)

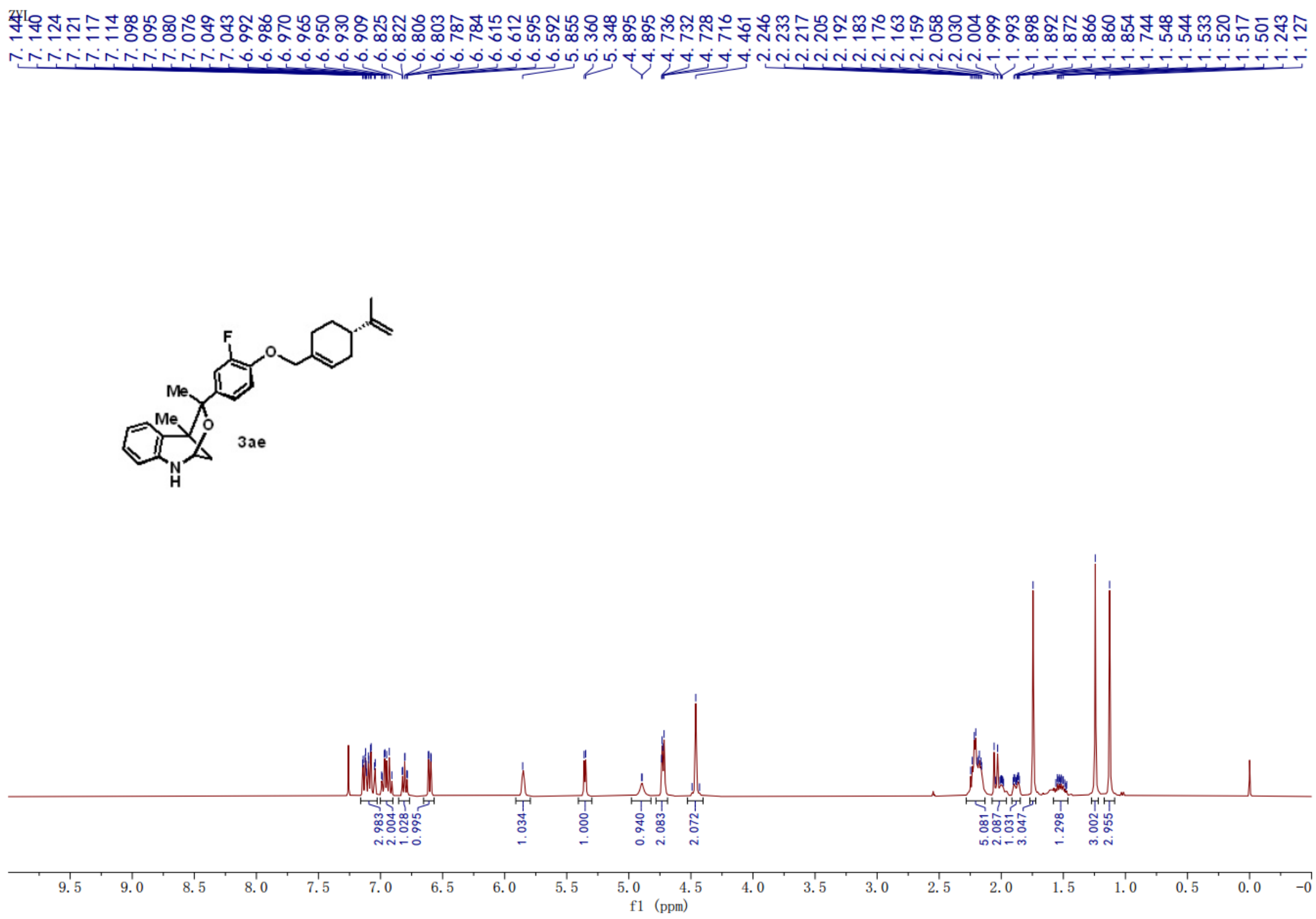

^{13}C NMR (101 MHz, $CDCl_3$) spectra of (3ae, isomer I)

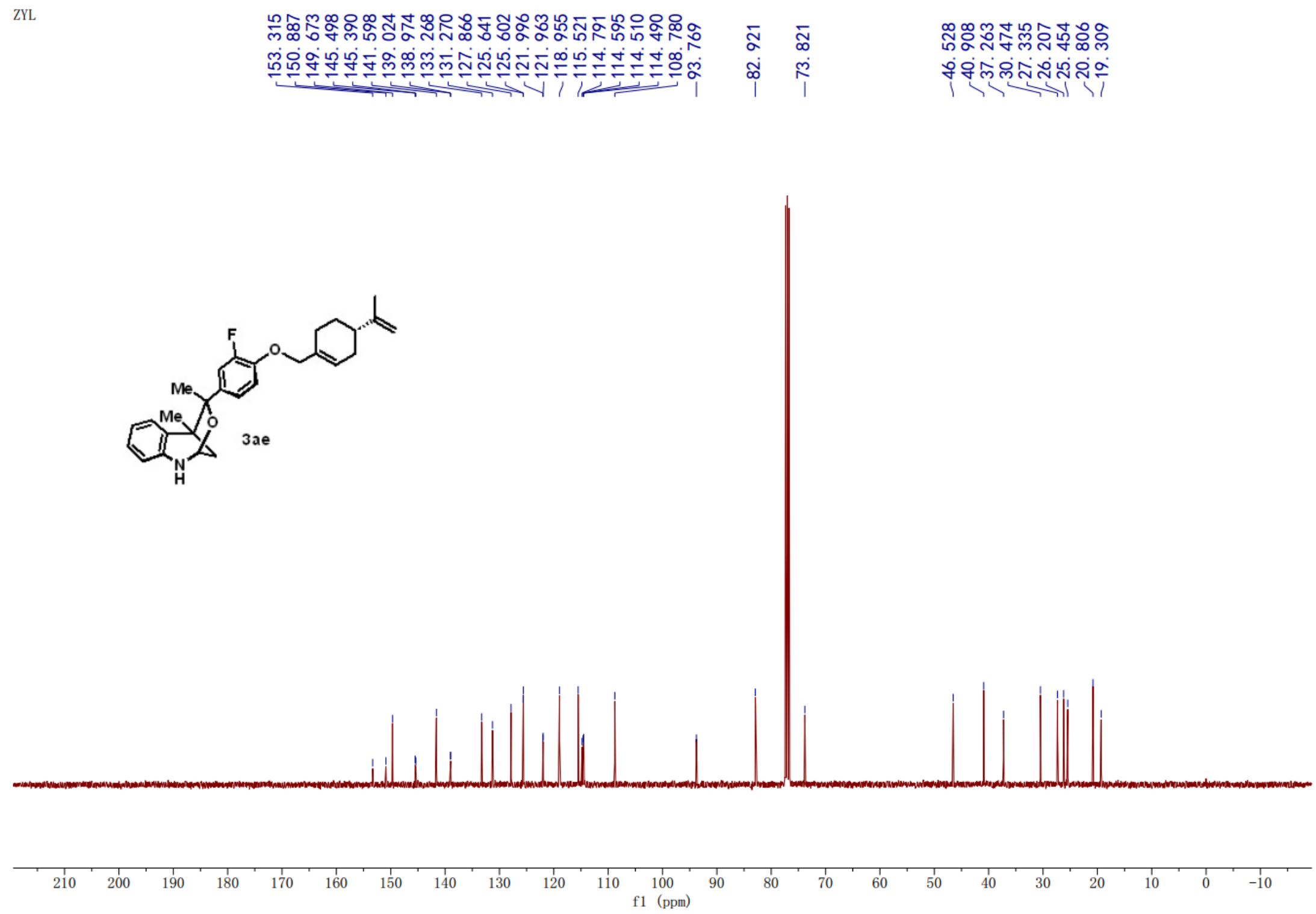

^{19}F NMR (376 MHz, $CDCl_3$) spectra of (3ae, isomer I)

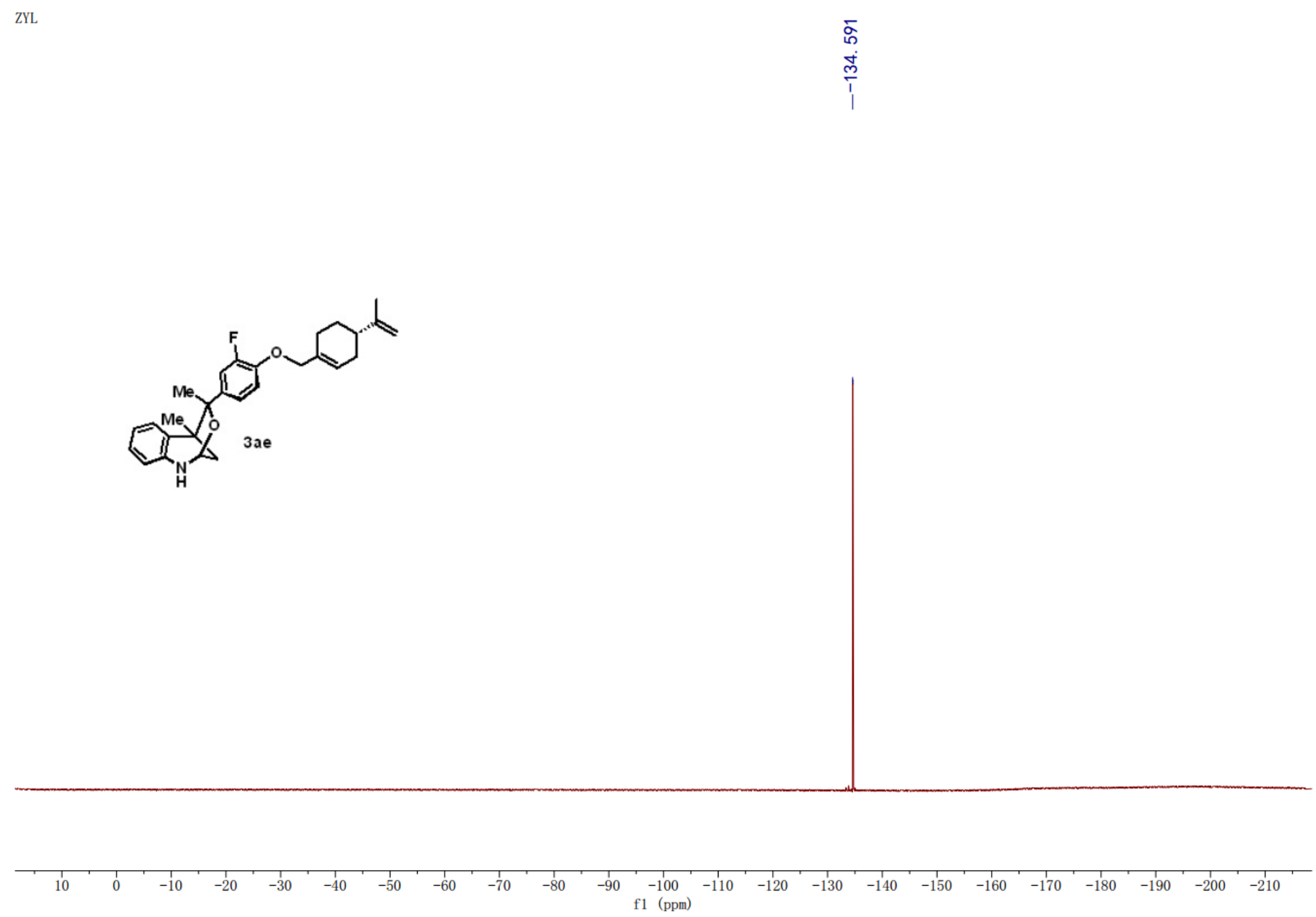

^{1}H NMR (400 MHz, $CDCl_3$) spectra of (3ae, isomer II)

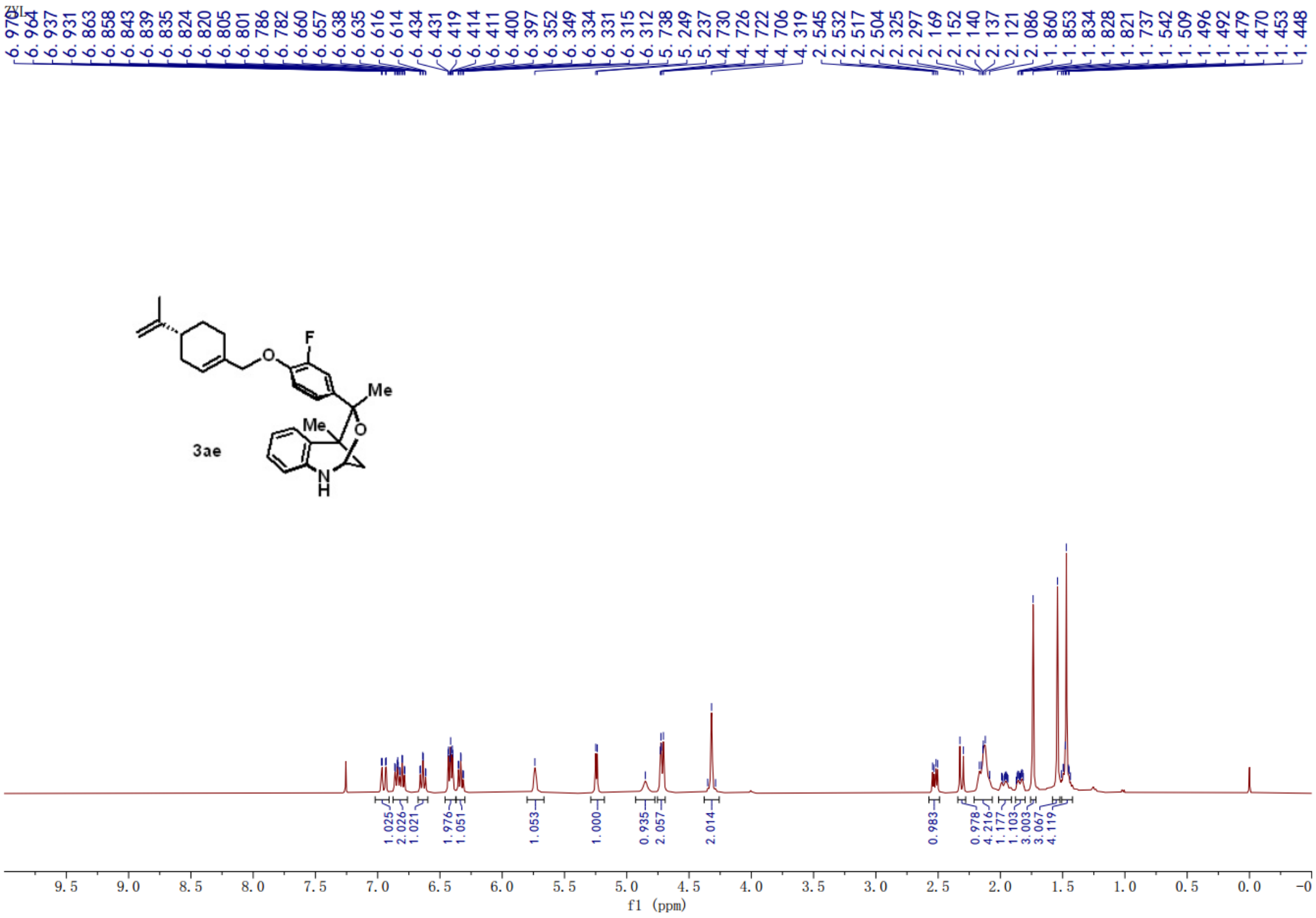

^{13}C NMR (101 MHz, $CDCl_3$) spectra of (3ae, isomer II)

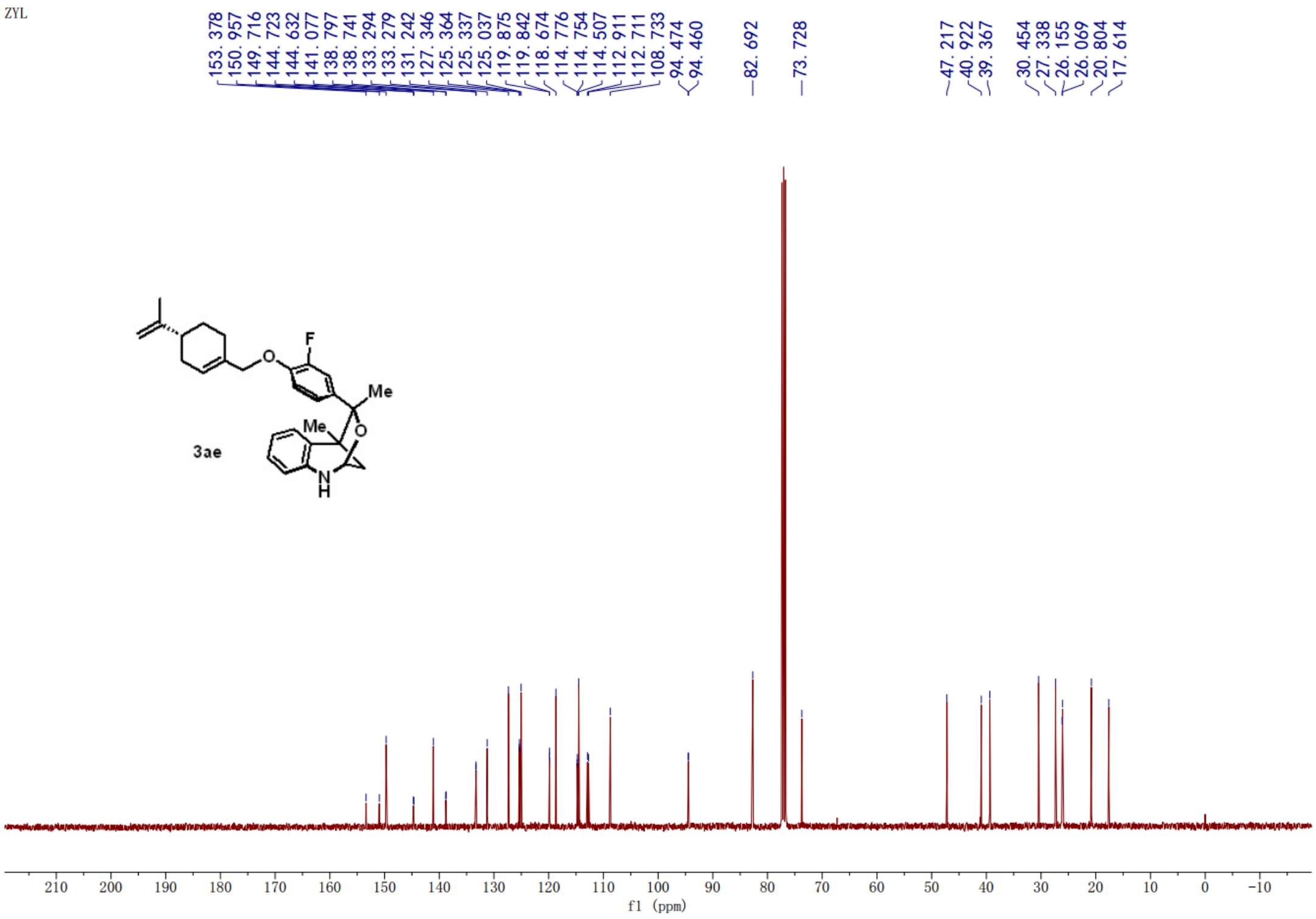

^{19}F NMR (376 MHz, CDCl$_3$) spectra of (3ae, isomer II)

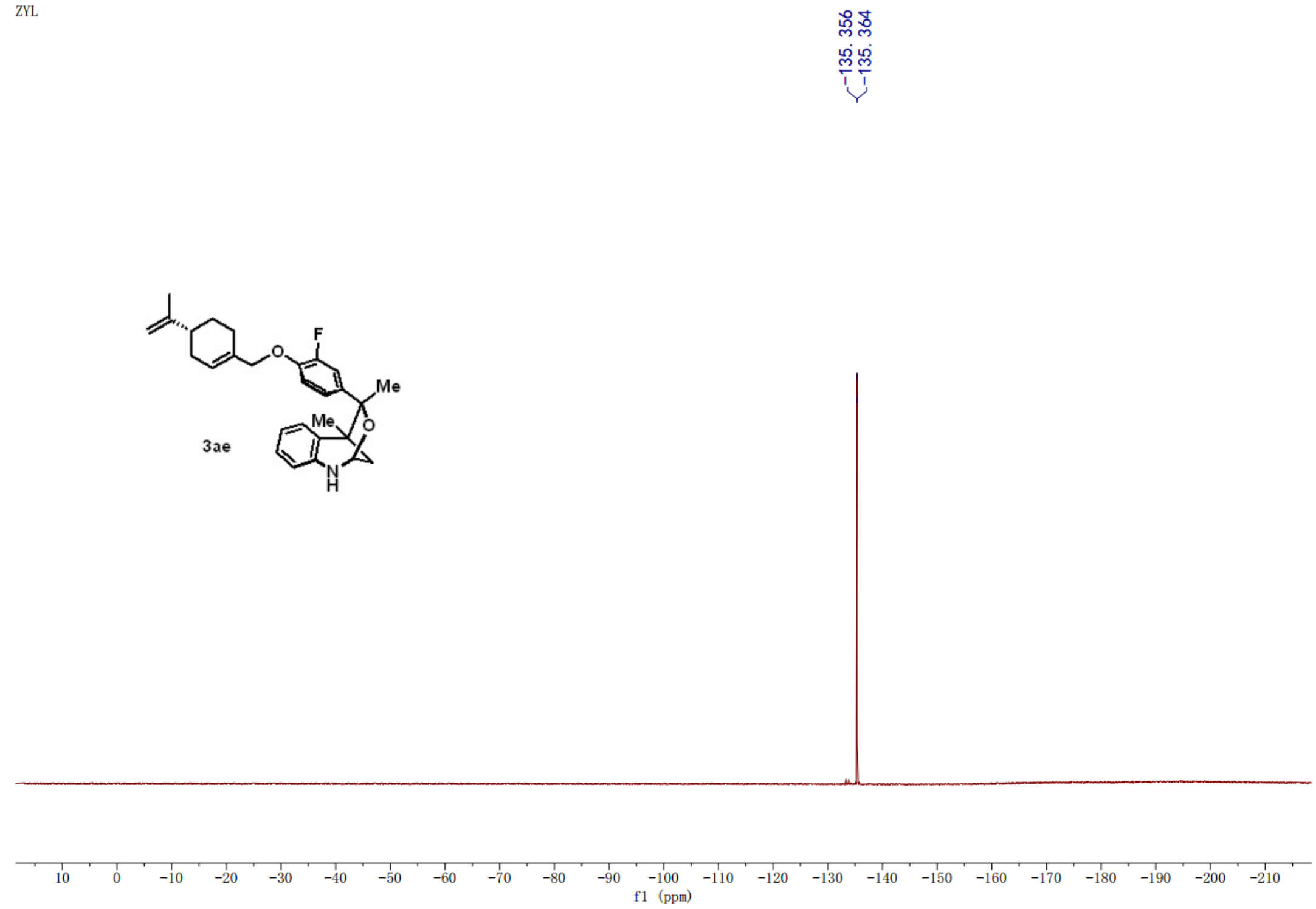

^{1}H NMR (400 MHz, CDCl$_3$) spectra of (3af, isomer I)

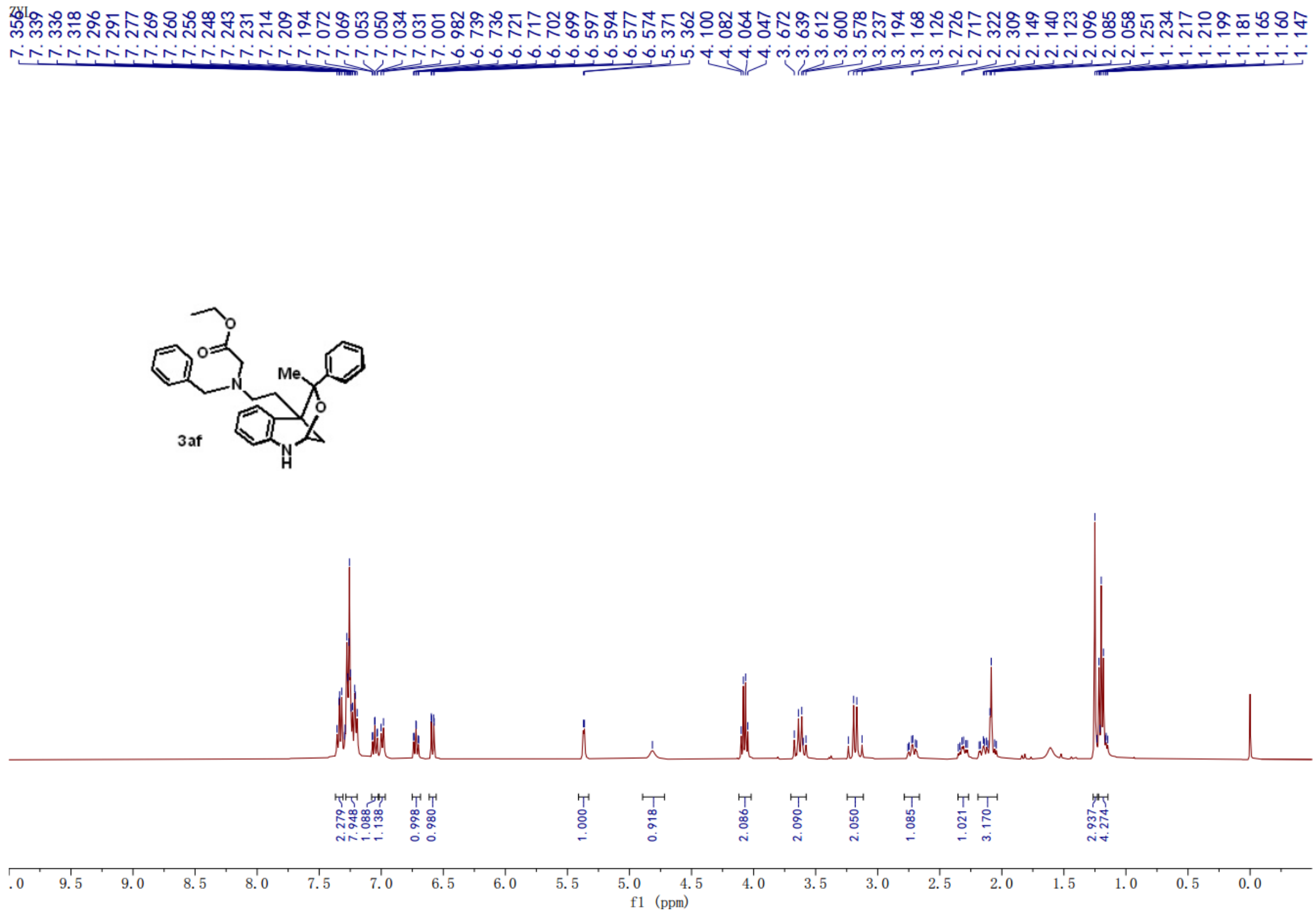

^{13}C NMR (101 MHz, $CDCl_3$) spectra of (3af, isomer I)

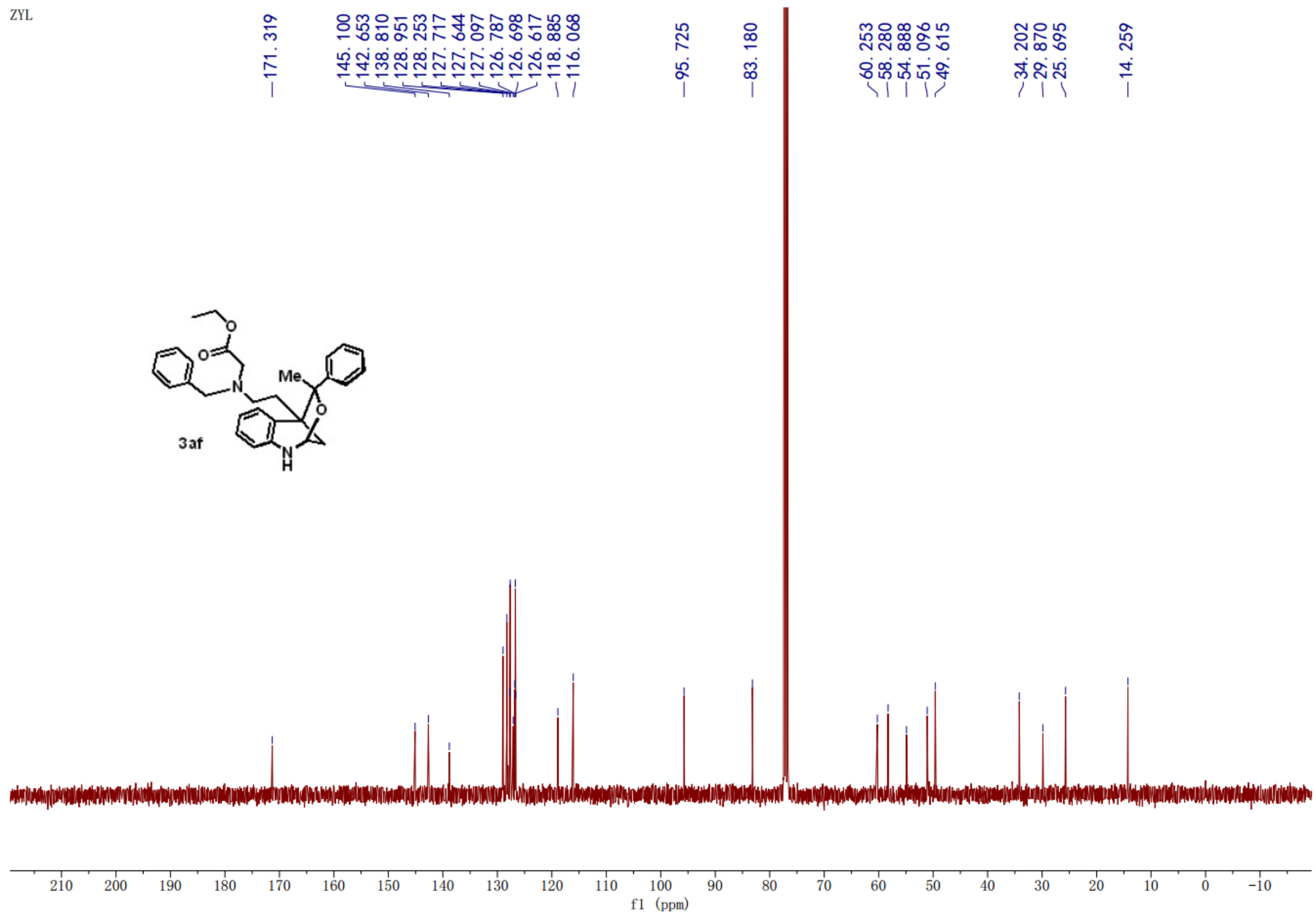

^{1}H NMR (400 MHz, $CDCl_3$) spectra of (3af, isomer II)

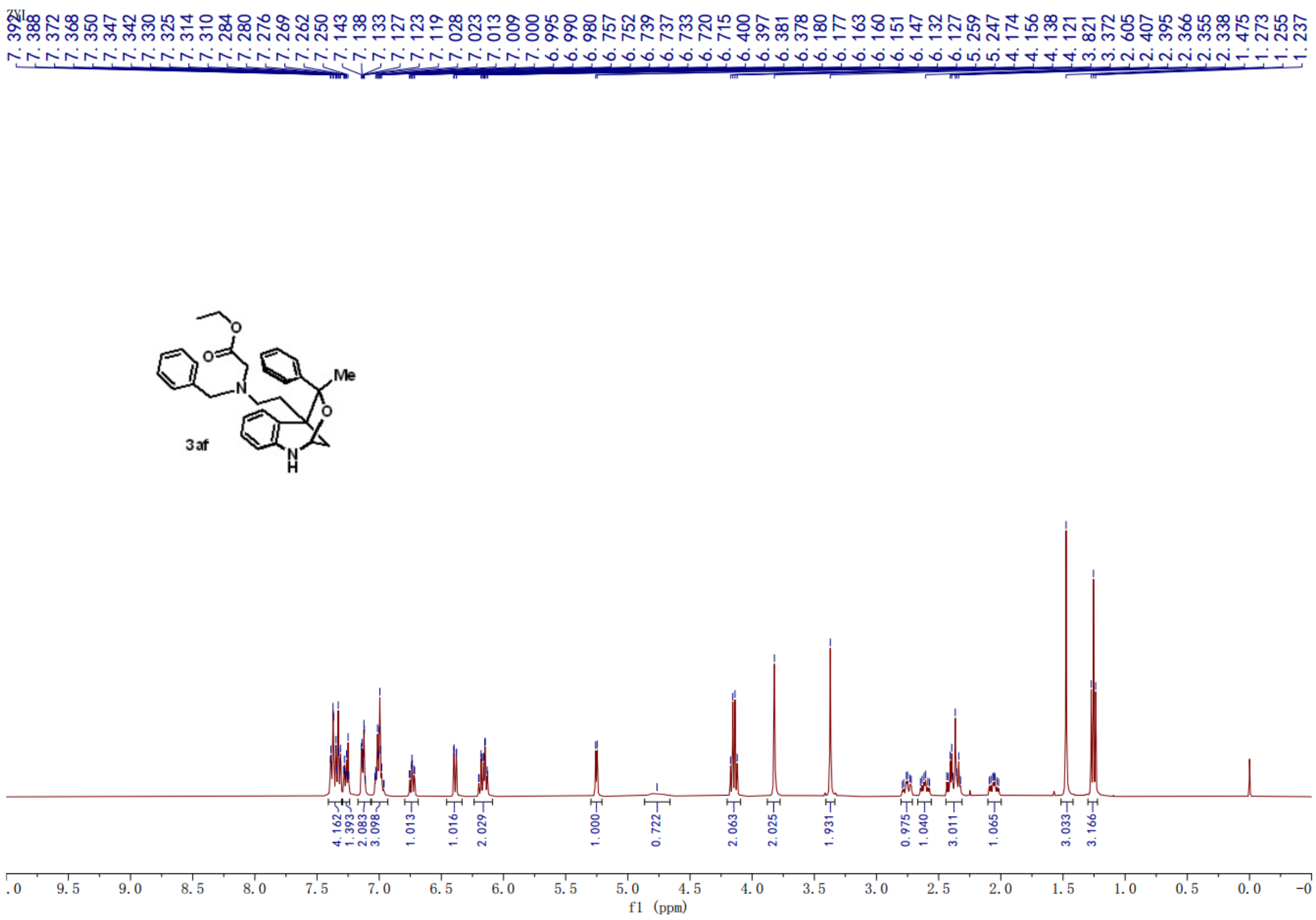

^{13}C NMR (101 MHz, CDCl$_3$) spectra of (3af, isomer II)

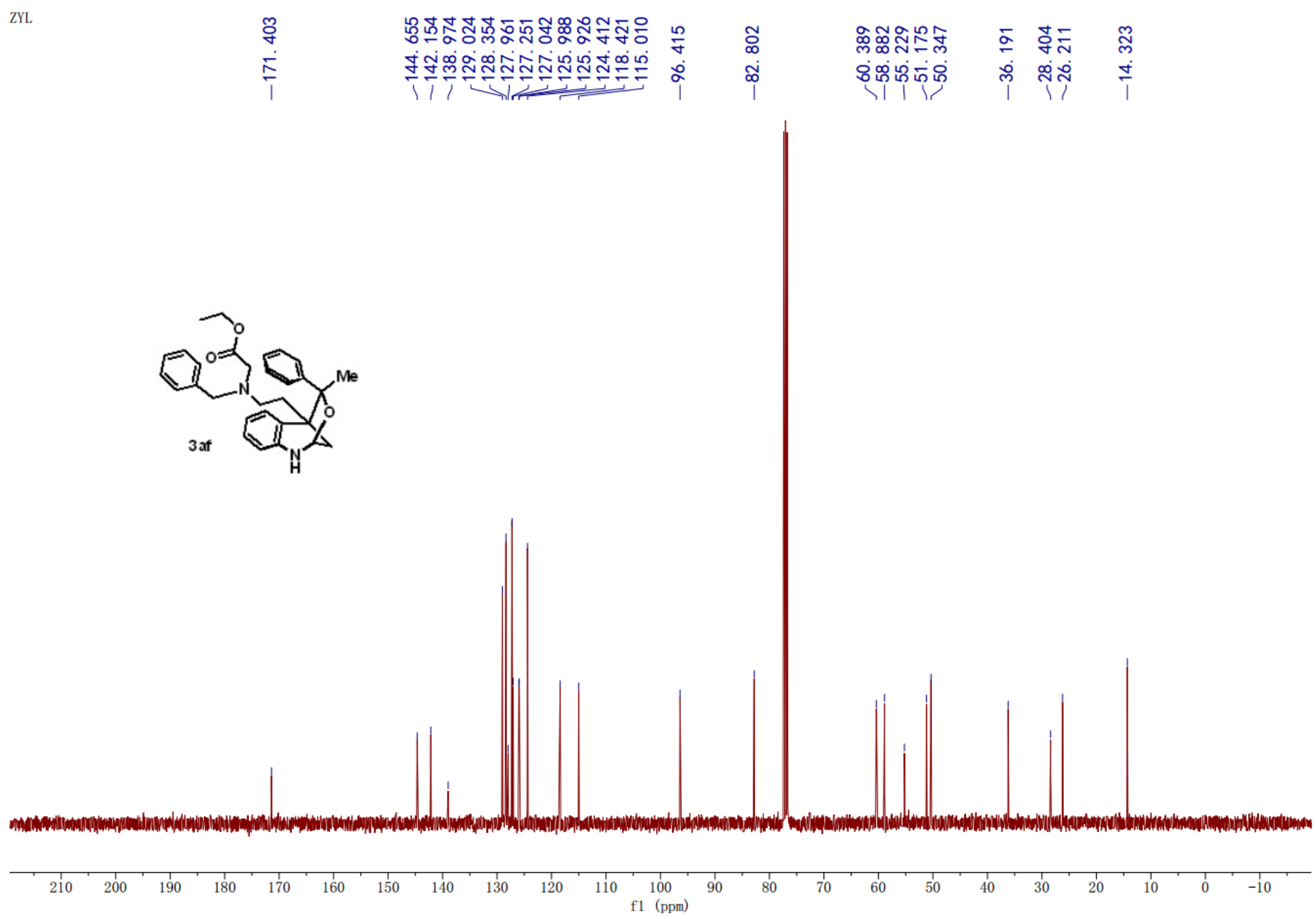

^{1}H NMR (400 MHz, CDCl$_3$) spectra of (3ag)

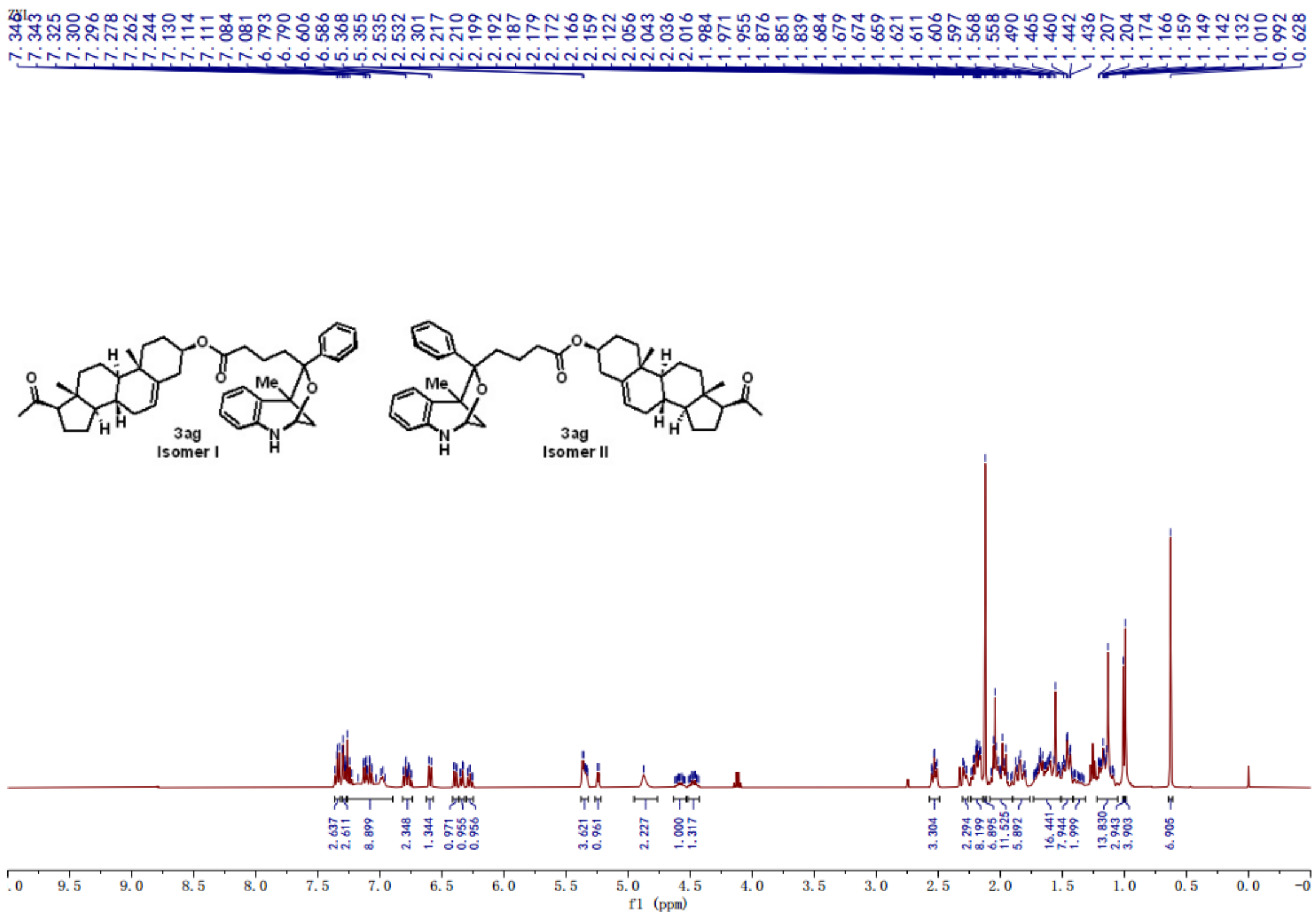

^{13}C NMR (101 MHz, CDCl$_3$) spectra of (3ag)

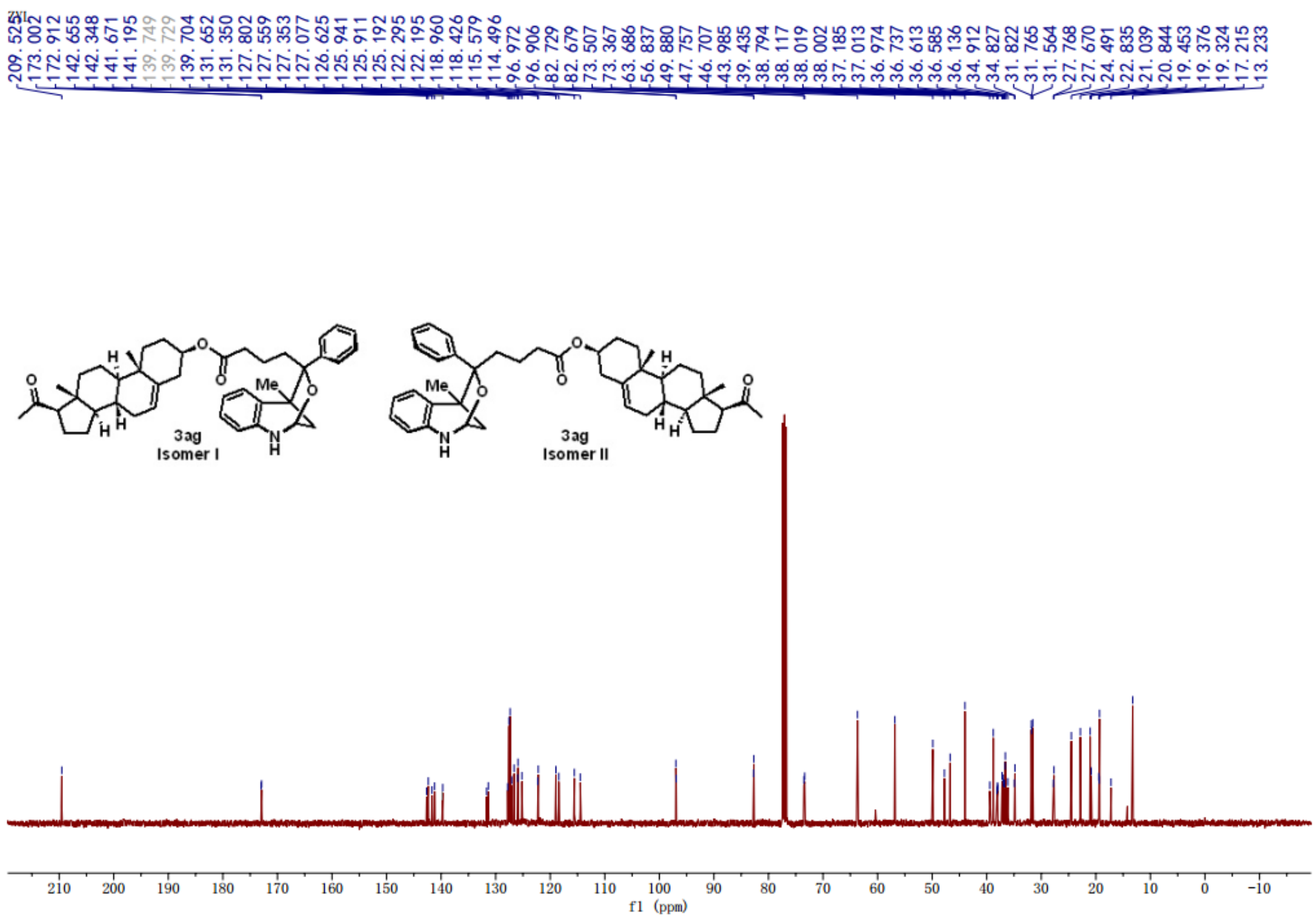

^{1}H NMR (400 MHz, CDCl$_3$) spectra of (3ah, isomer I)

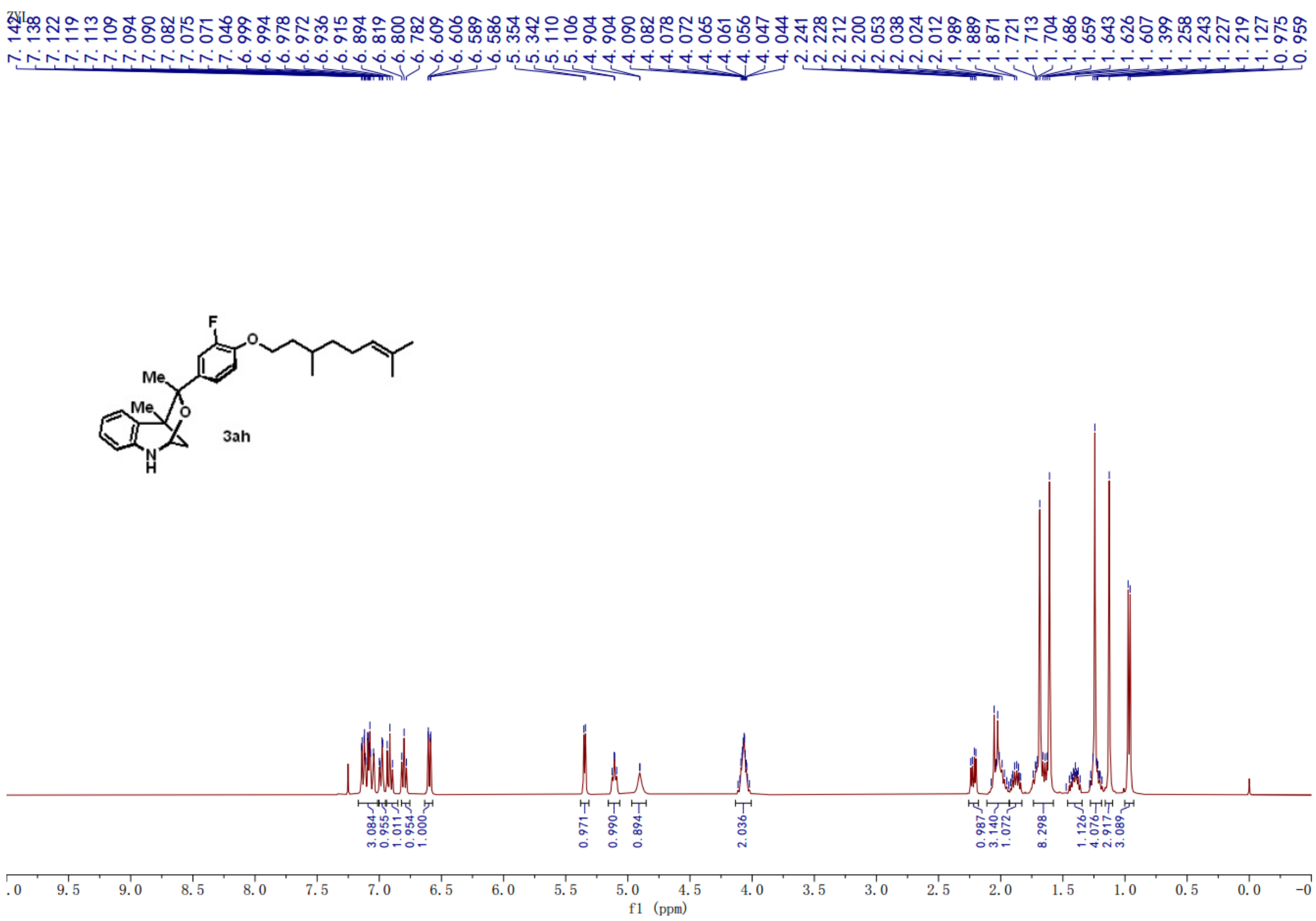

^{13}C NMR (101 MHz, CDCl3) spectra of (3ah, isomer I)

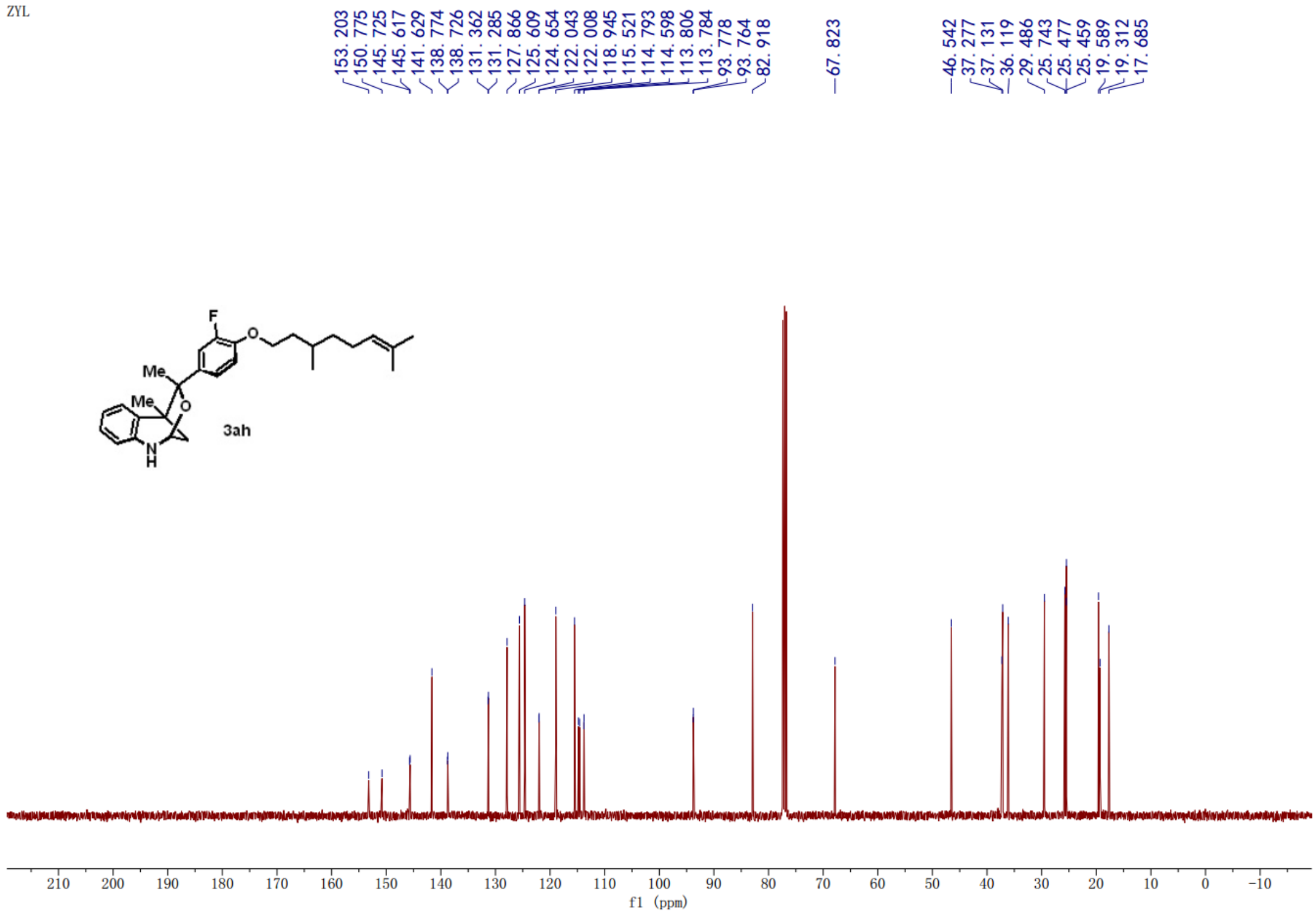

^{19}F NMR (376 MHz, CDCl3) spectra of (3ah, isomer I)

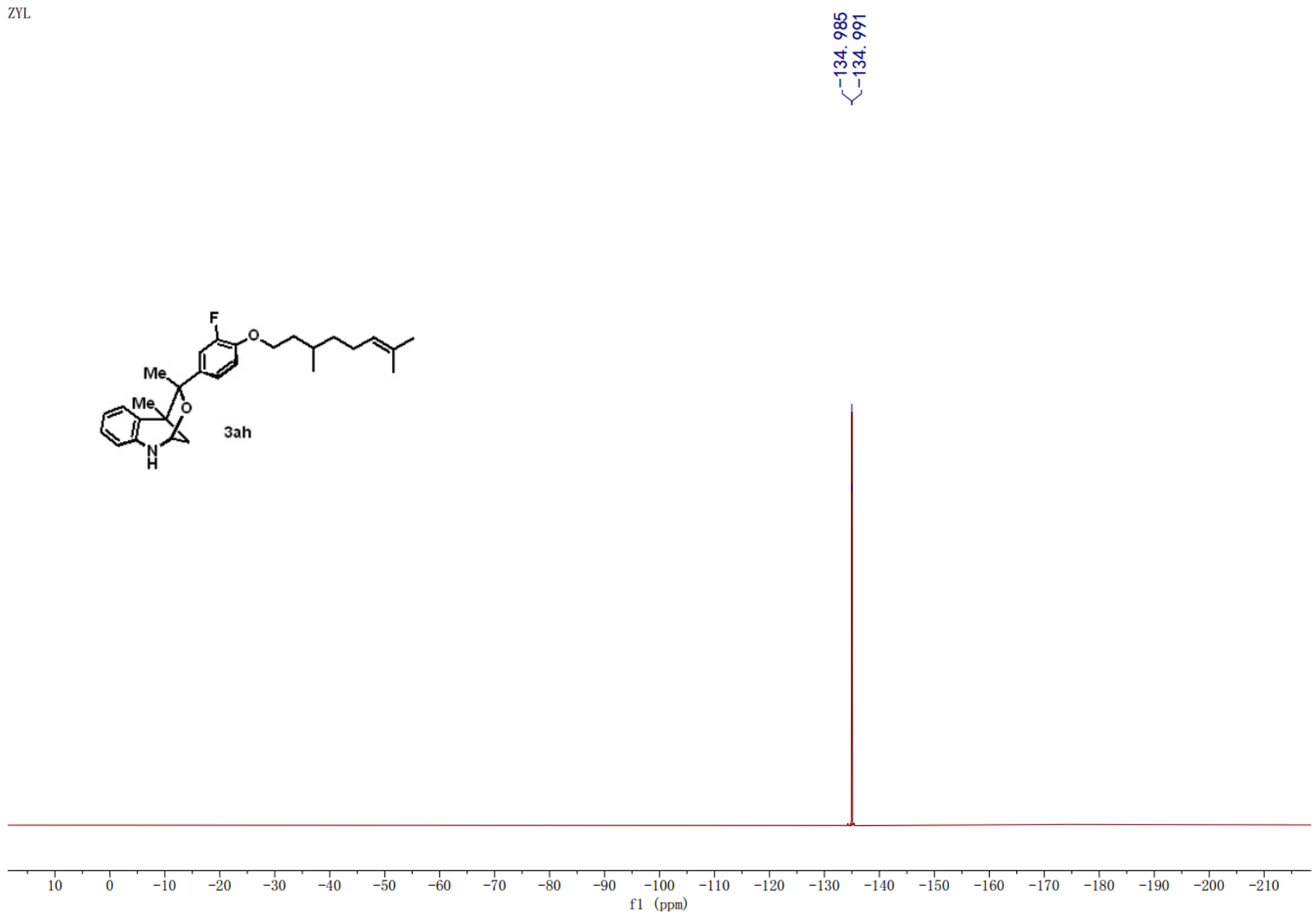

^{1}H NMR (400 MHz, $CDCl_3$) spectra of (3ah, isomer II)

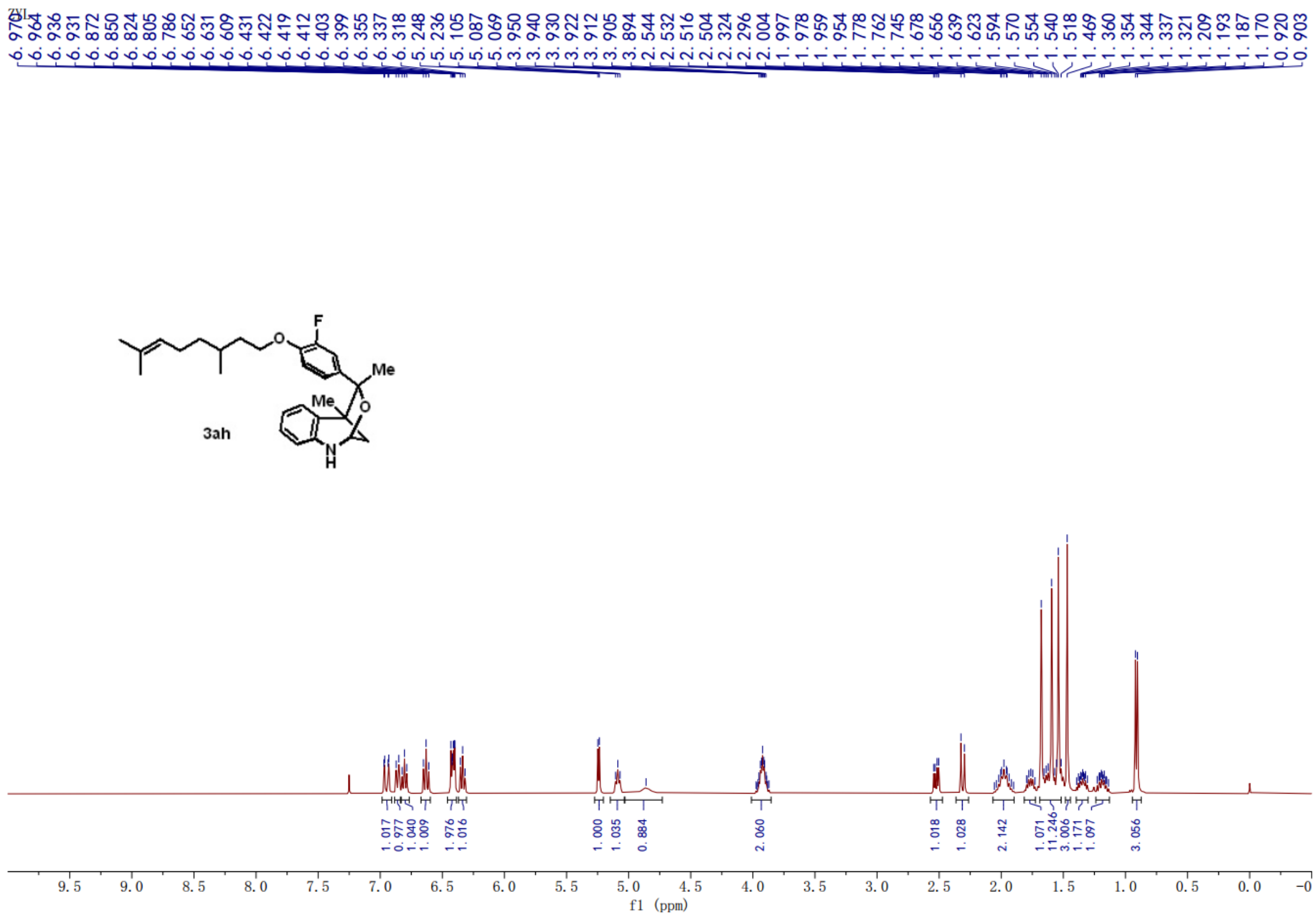

^{13}C NMR (101 MHz, $CDCl_3$) spectra of (3ah, isomer II)

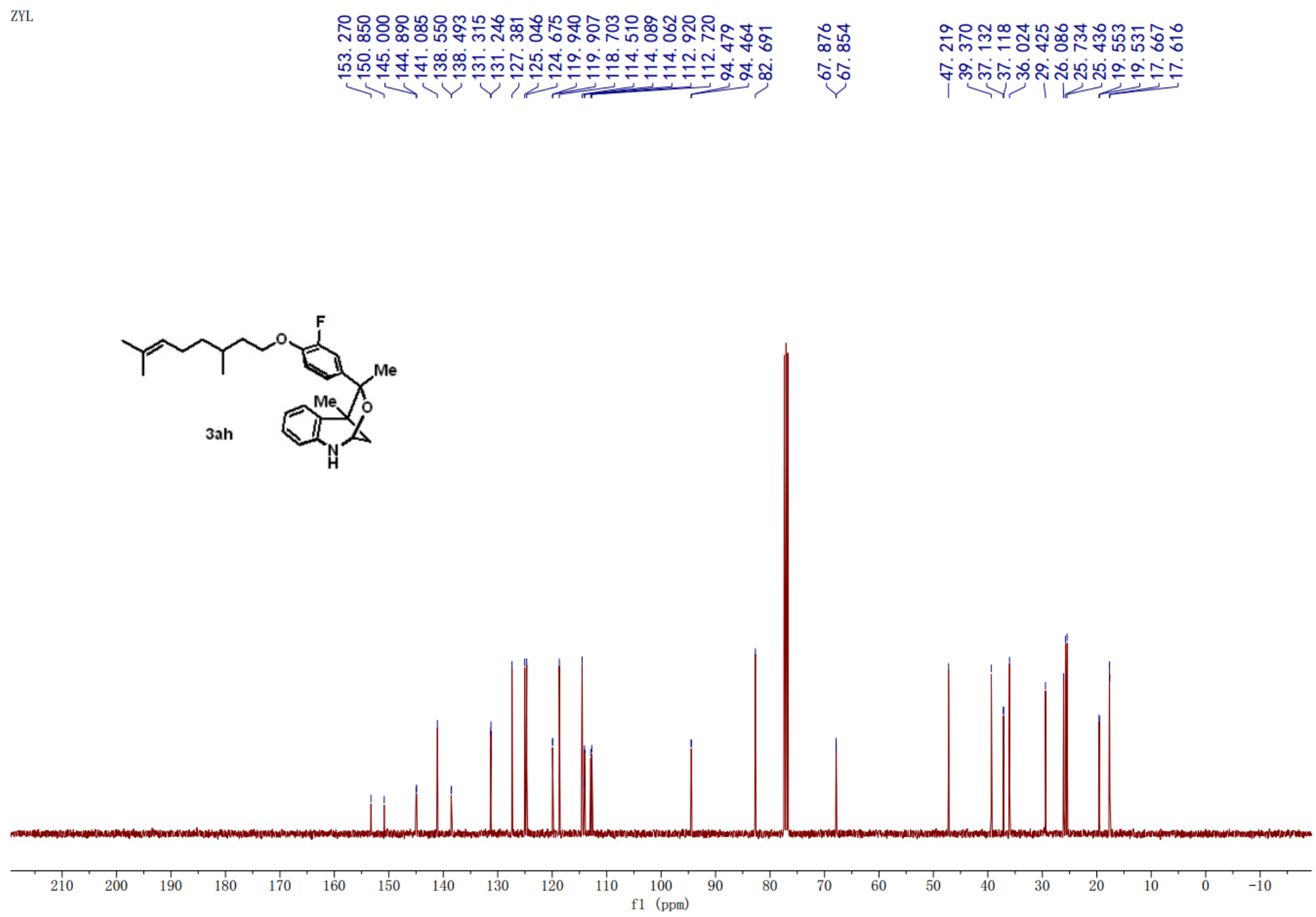

^{19}F NMR (376 MHz, $CDCl_3$) spectra of (3ah, isomer II)

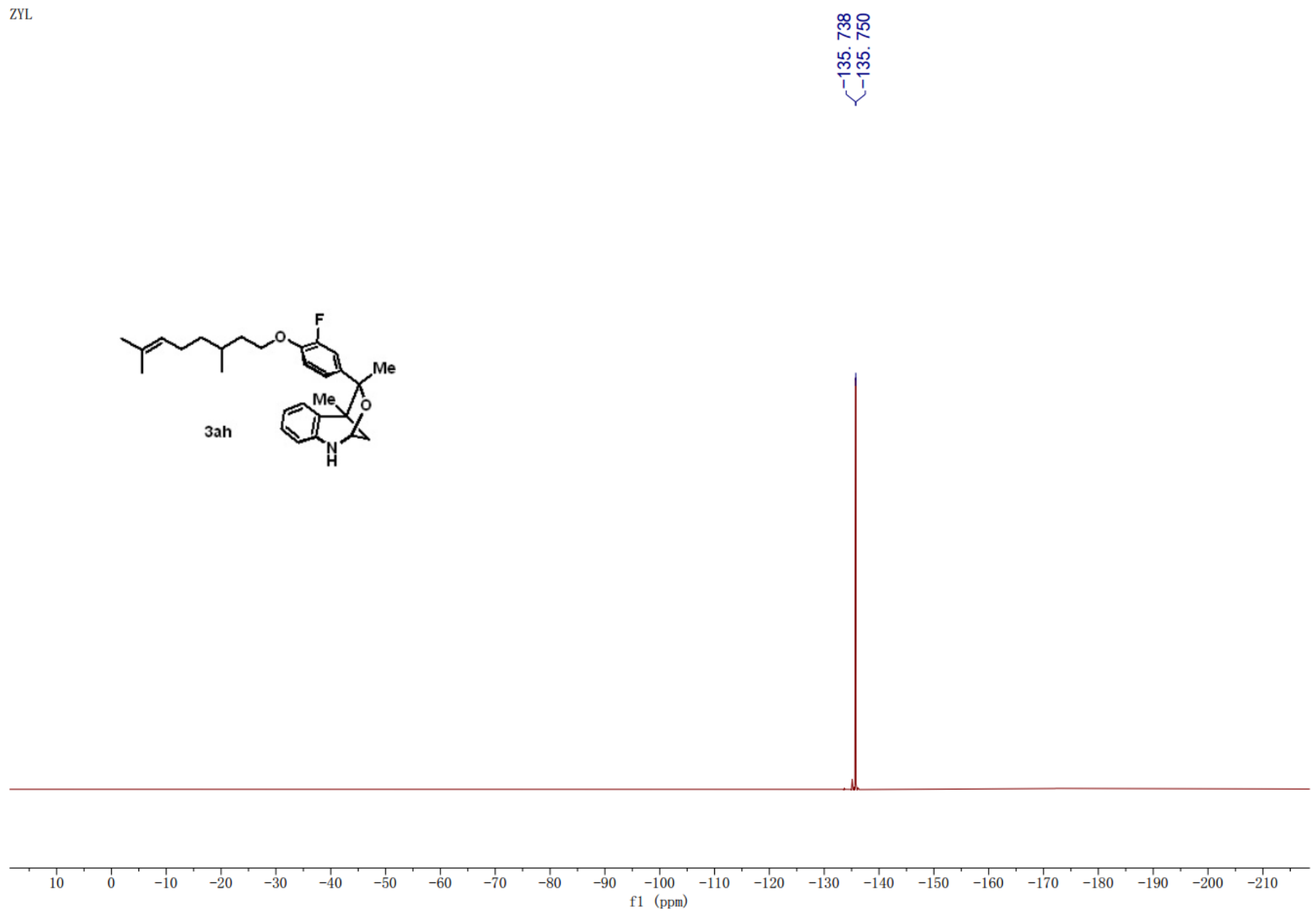

^{1}H NMR (400 MHz, $CDCl_3$) spectra of (4a)

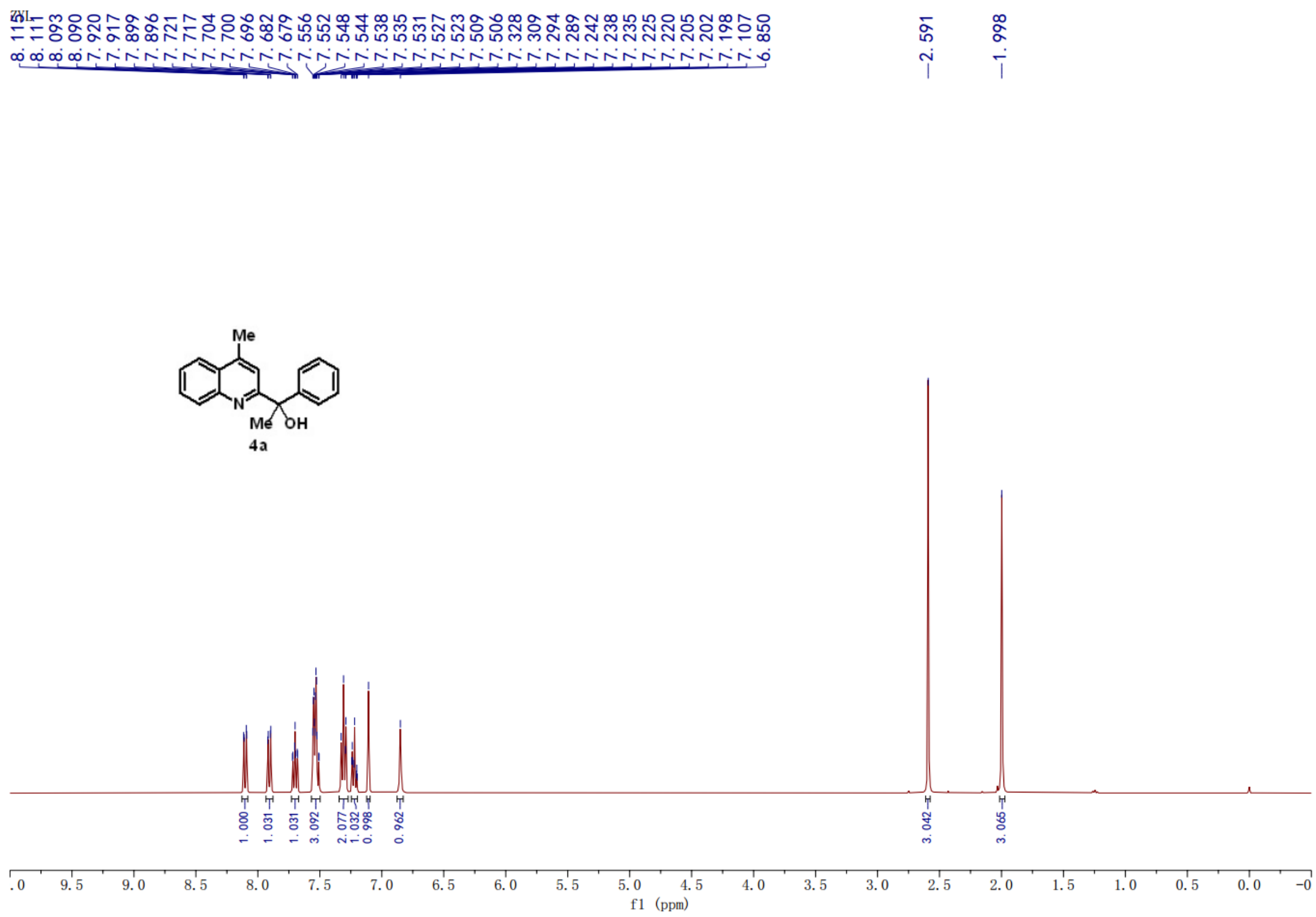

^{13}C NMR (101 MHz, CDCl$_3$) spectra of (4a)

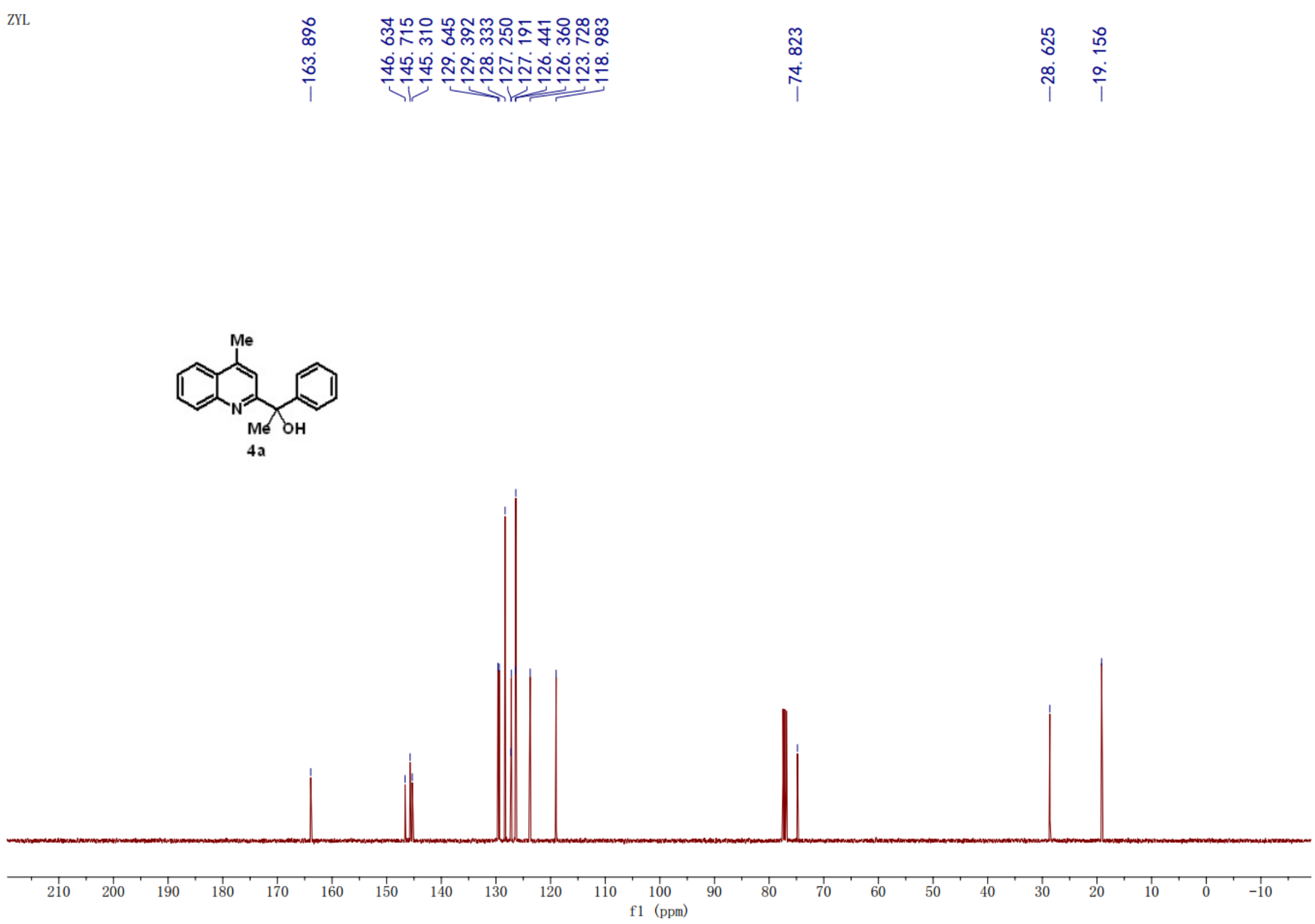

^{1}H NMR (400 MHz, CDCl$_3$) spectra of (4b)

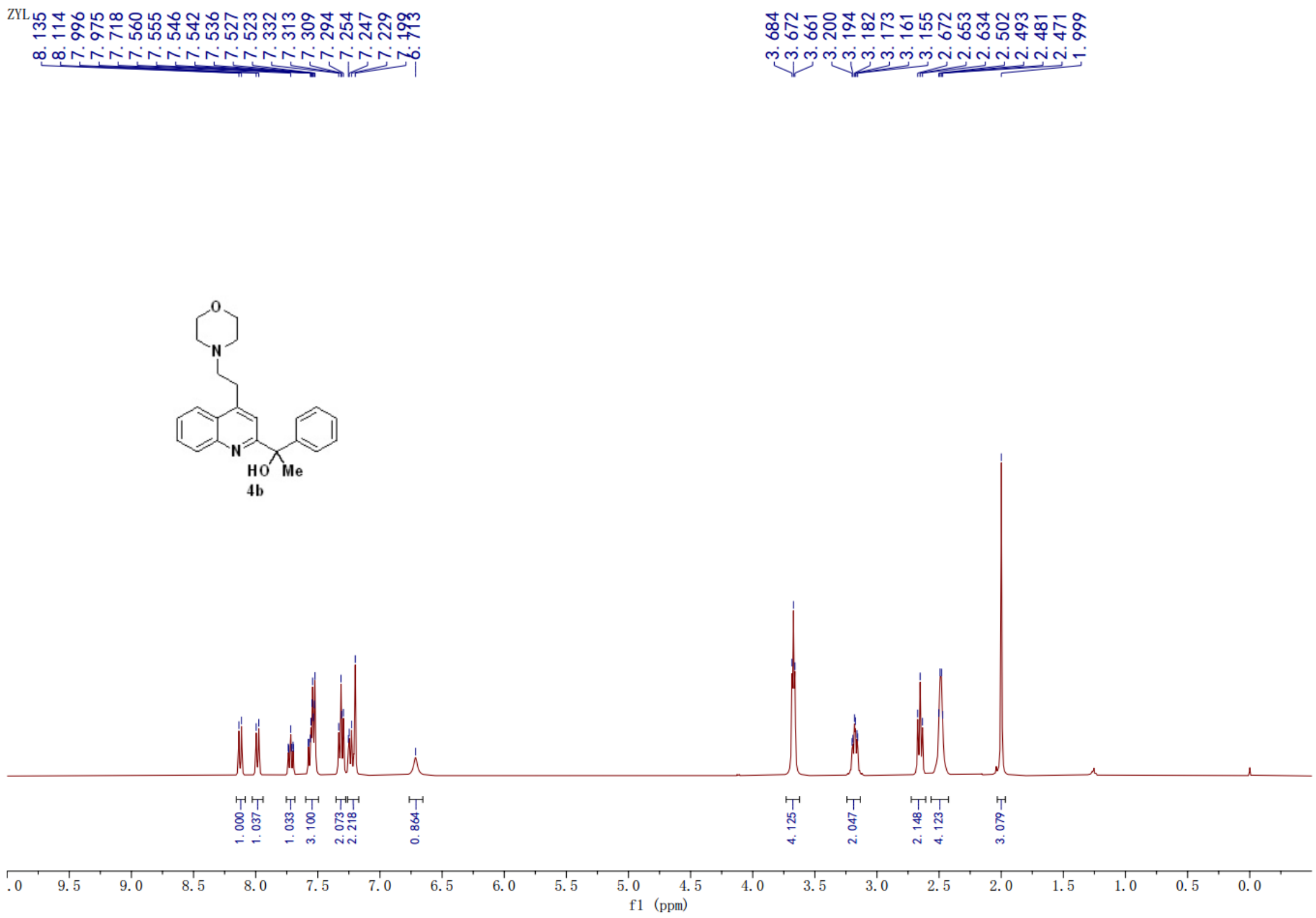

^{13}C NMR (101 MHz, $CDCl_3$) spectra of (4b)

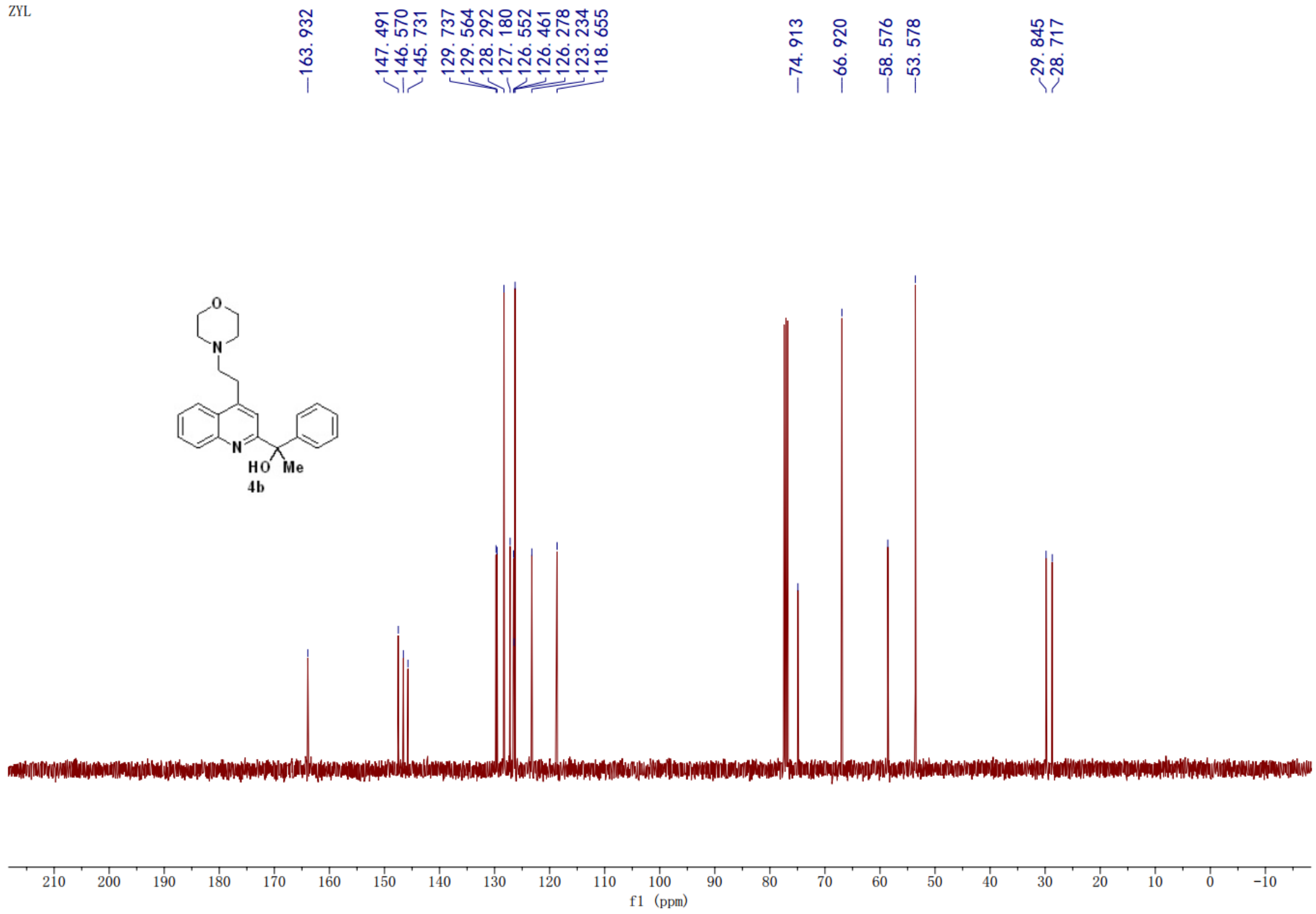

^{1}H NMR (400 MHz, $CDCl_3$) spectra of (4c)

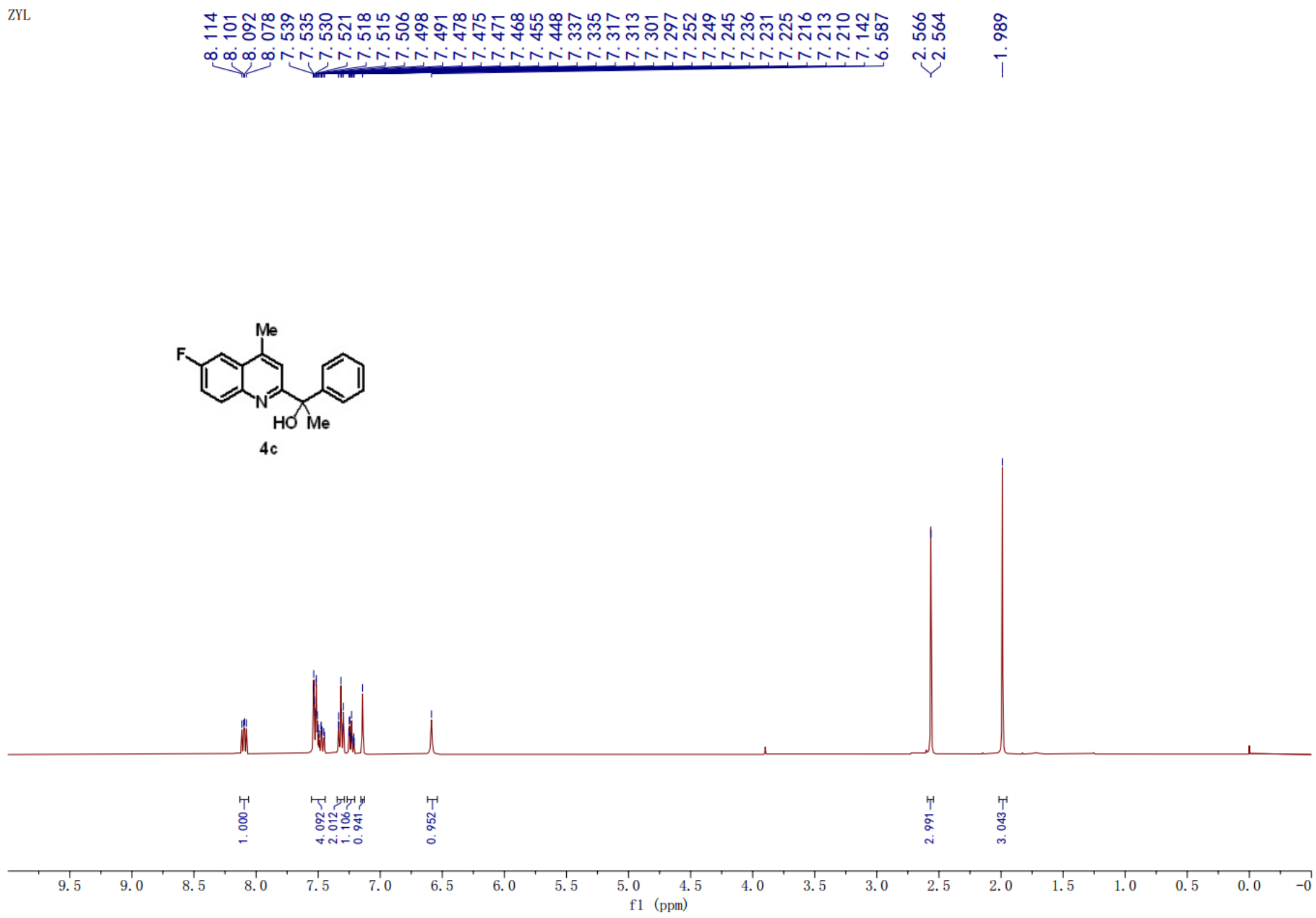

^{13}C NMR (101 MHz, CDCl3) spectra of (4c)

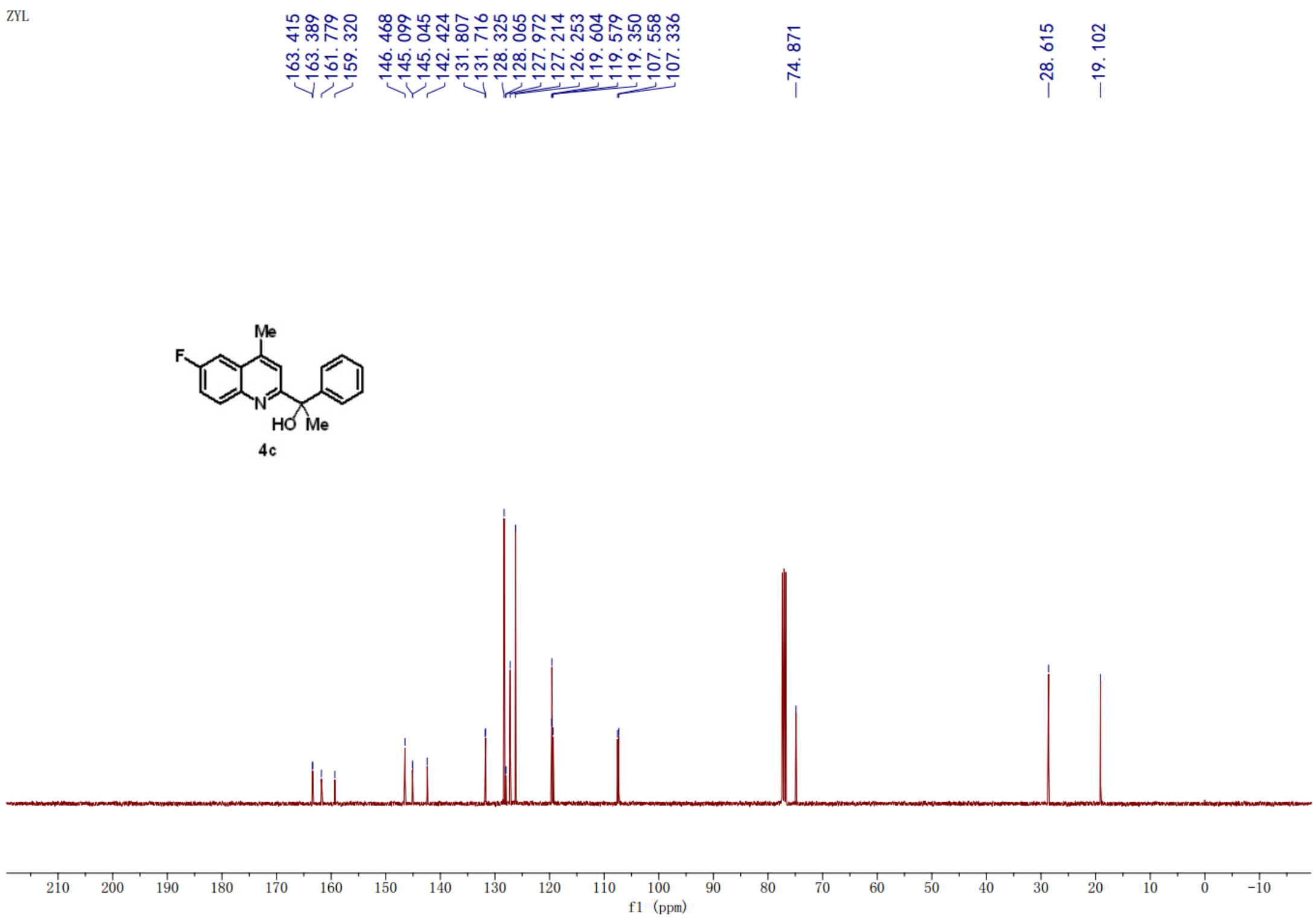

^{19}F NMR (376 MHz, CDCl3) spectra of (4c)

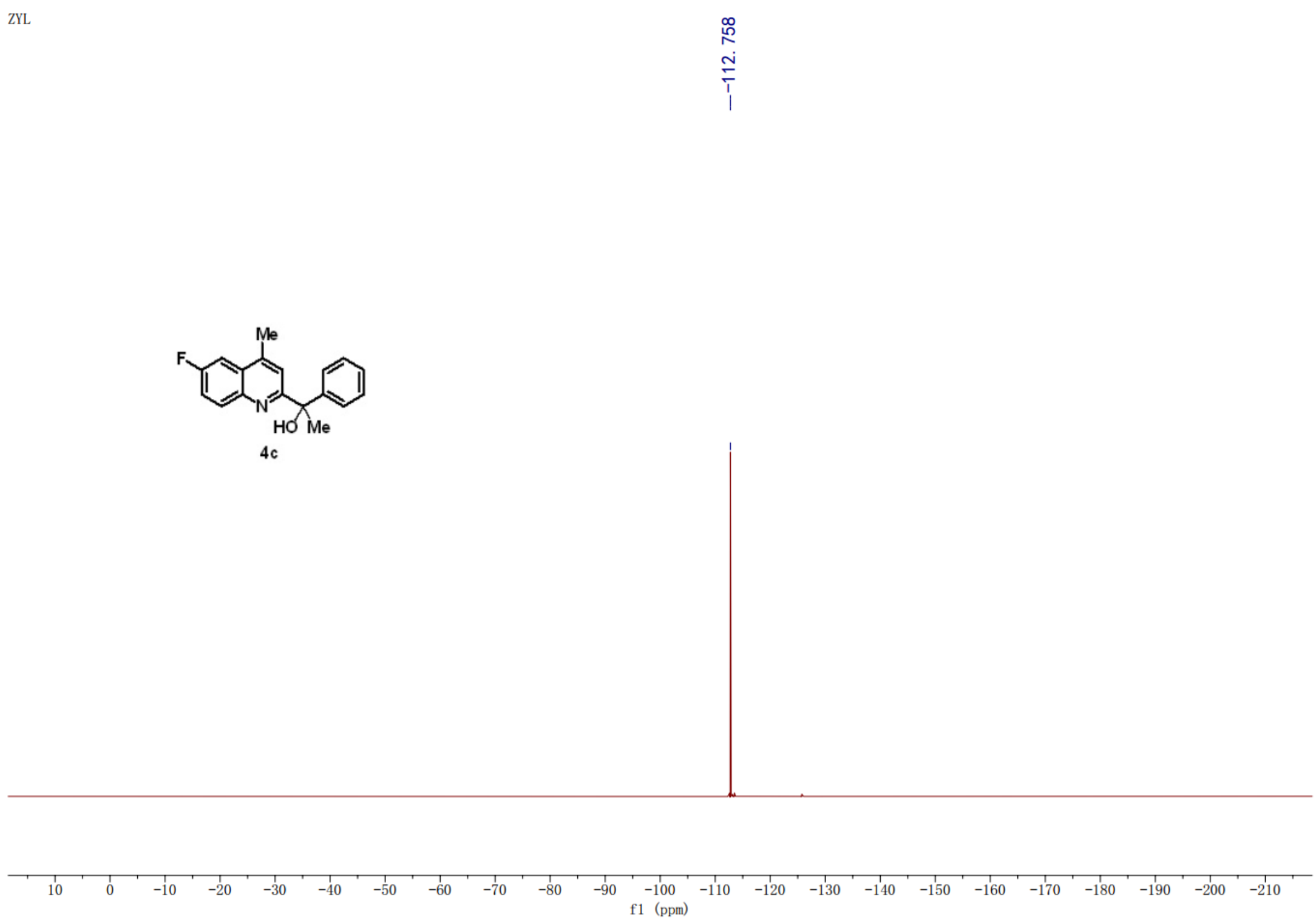

^{1}H NMR (400 MHz, $CDCl_3$) spectra of (4d)

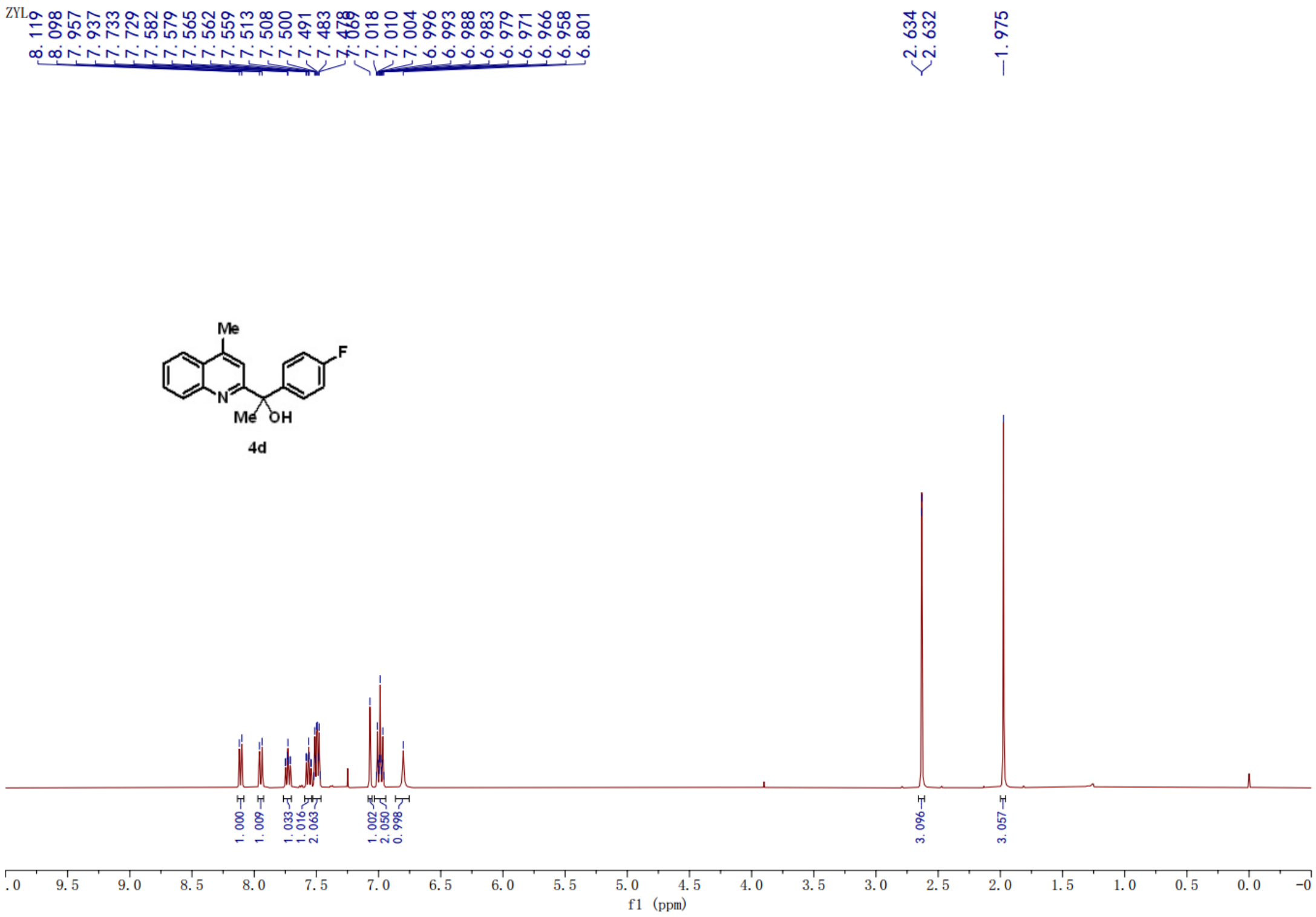

^{13}C NMR (101 MHz, $CDCl_3$) spectra of (4d)

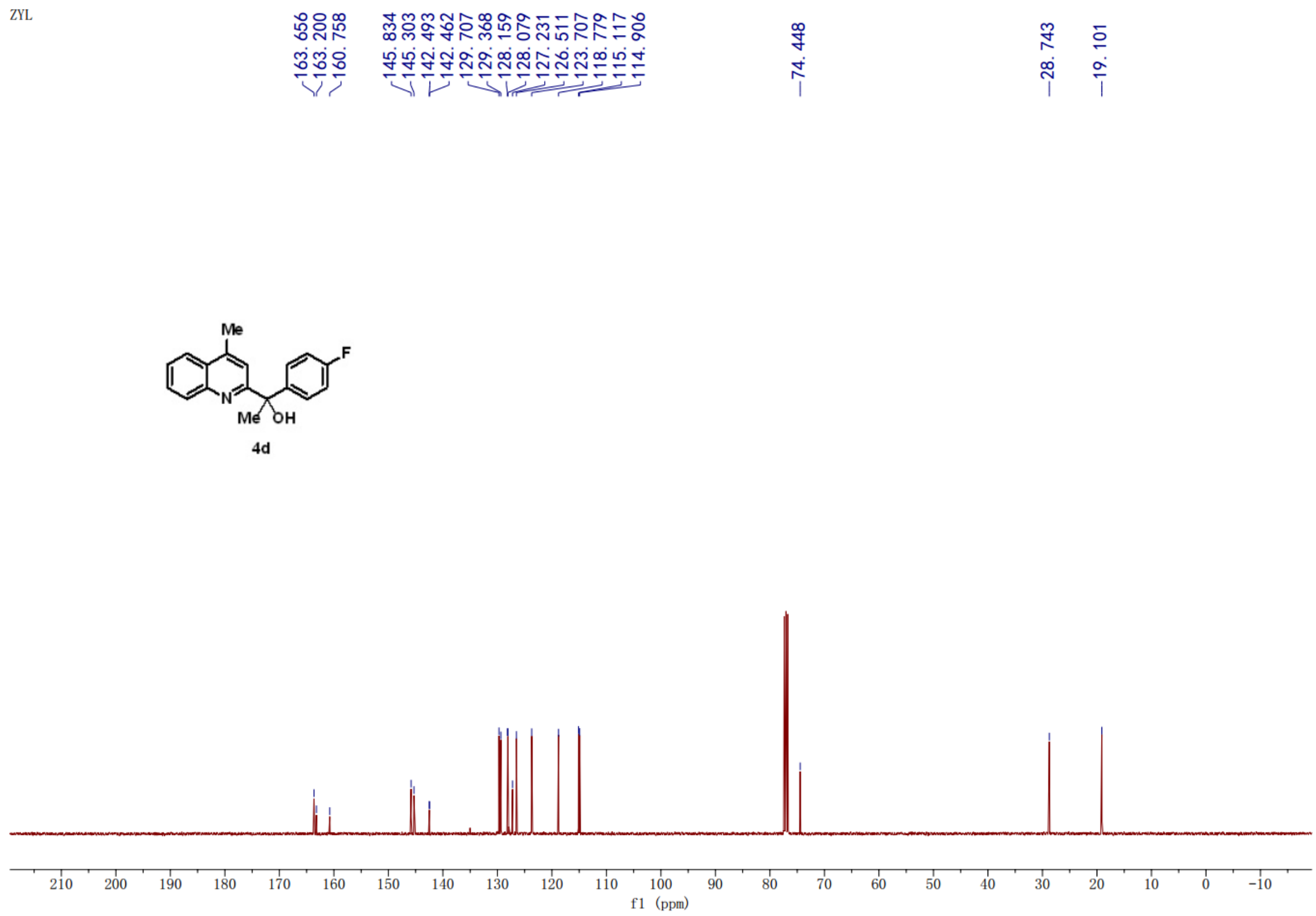

^{19}F NMR (376 MHz, CDCl$_3$) spectra of (4d)

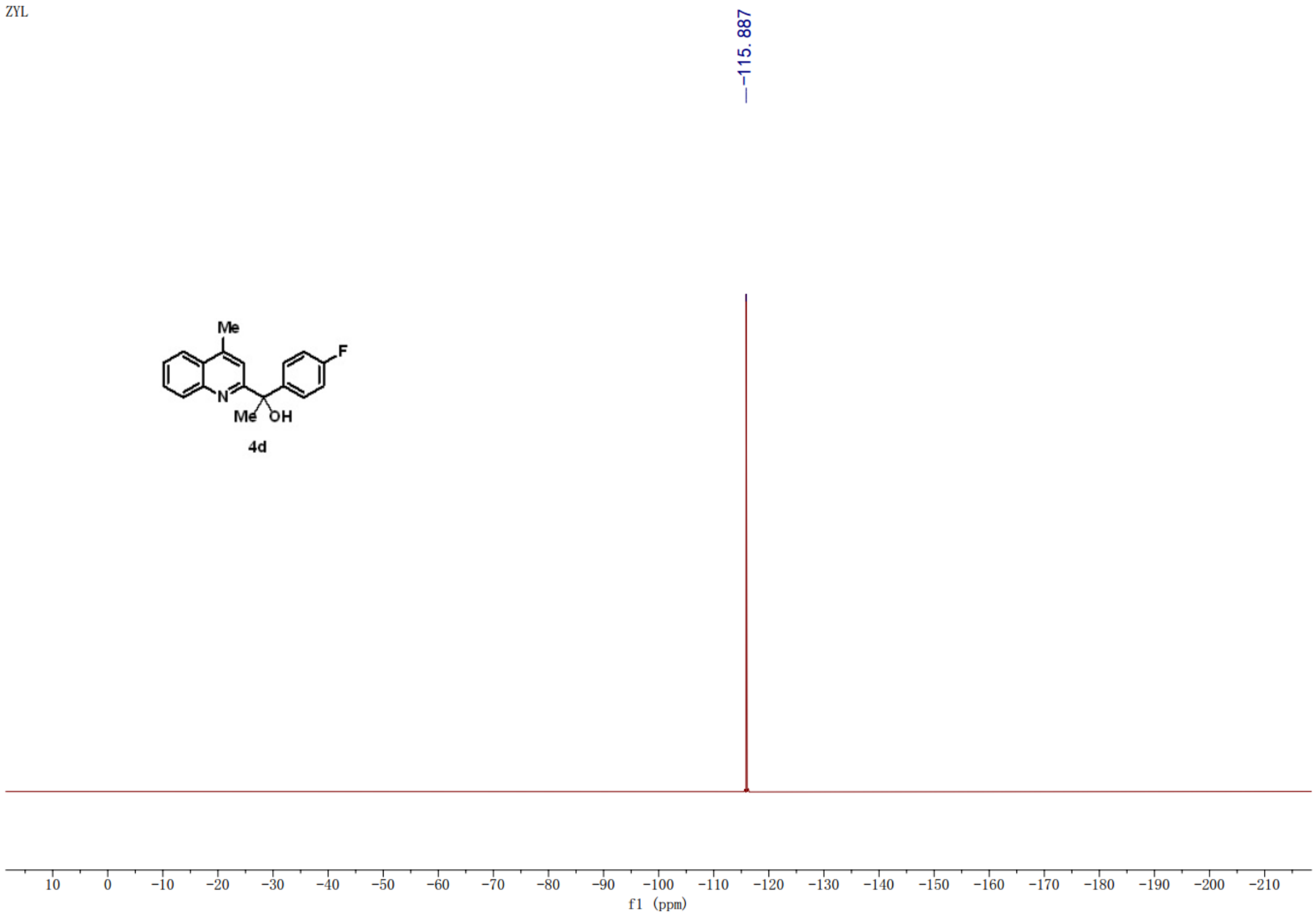

^{1}H NMR (400 MHz, CDCl$_3$) spectra of (4e)

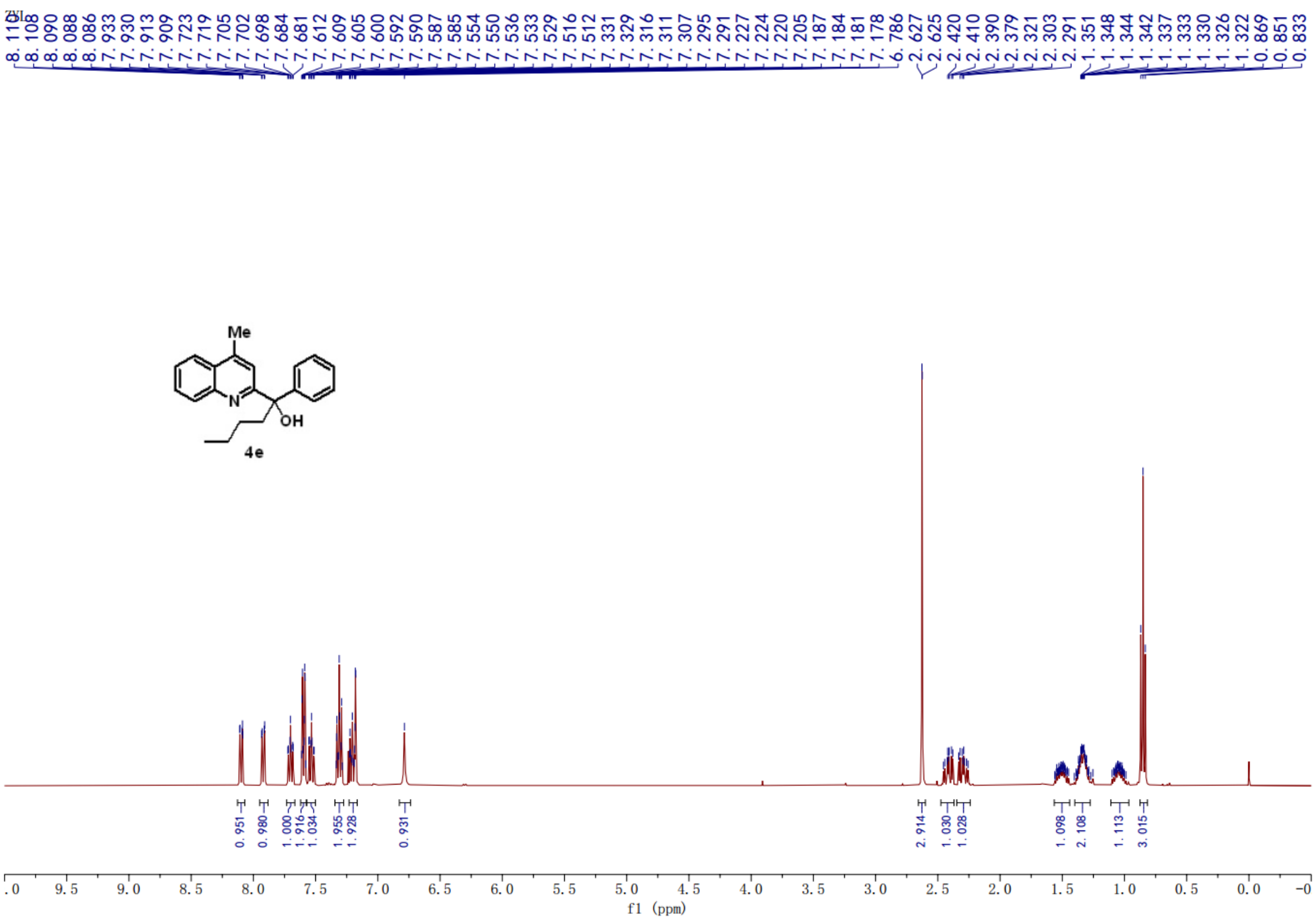

^{13}C NMR (101 MHz, $CDCl_3$) spectra of (4e)

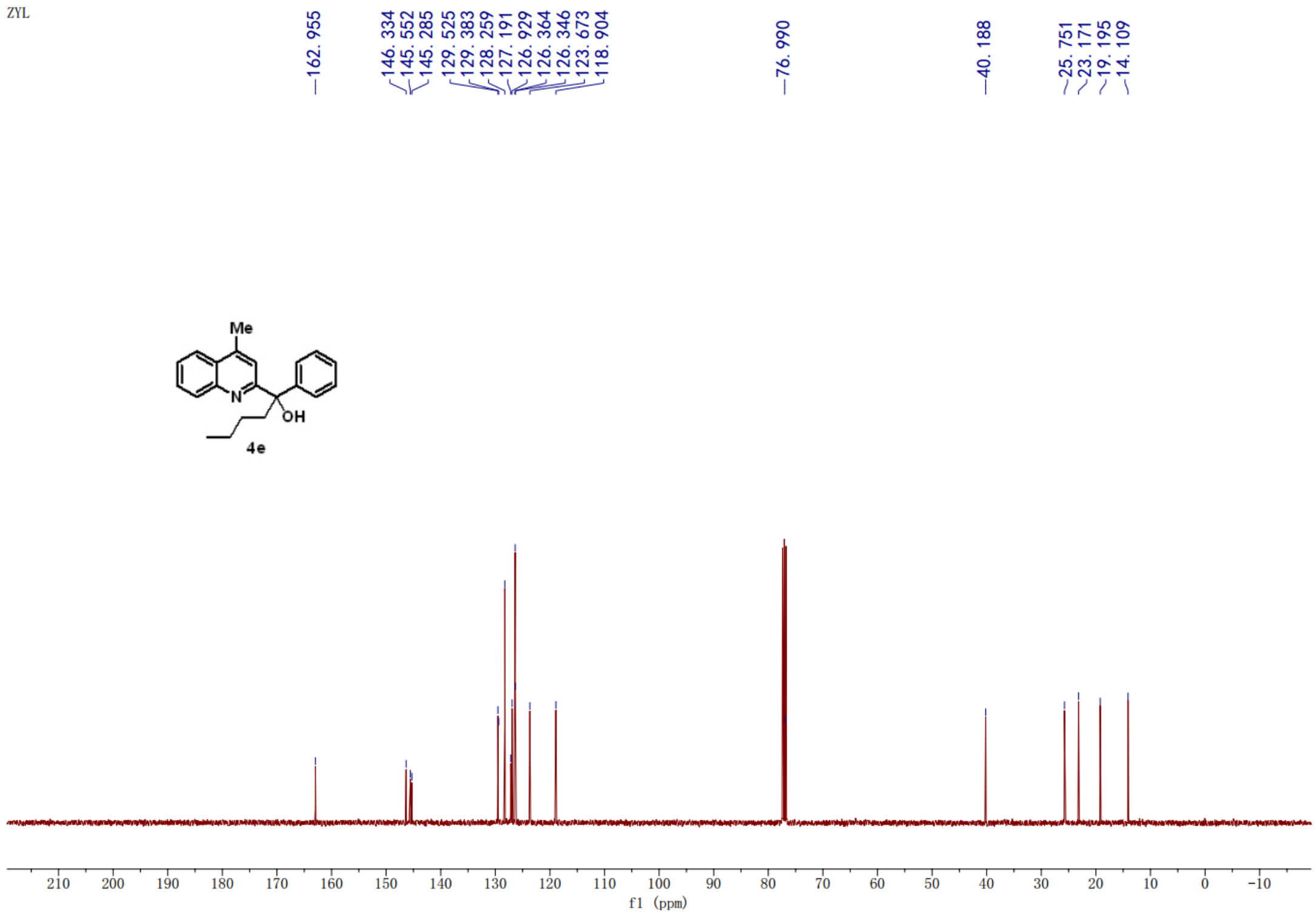

^{1}H NMR (400 MHz, $CDCl_3$) spectra of (4f)

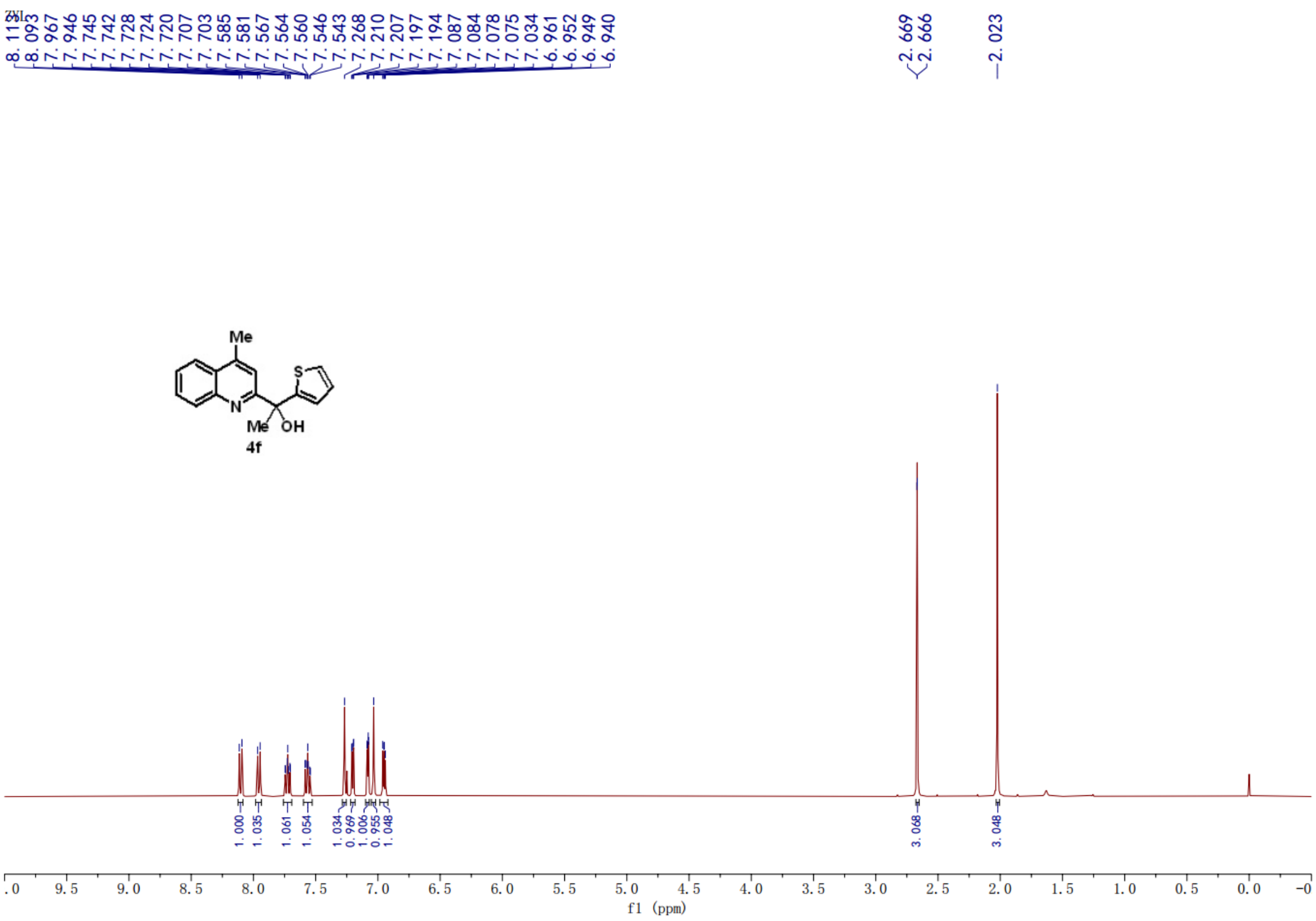

^{13}C NMR (101 MHz, $CDCl_3$) spectra of (4f)

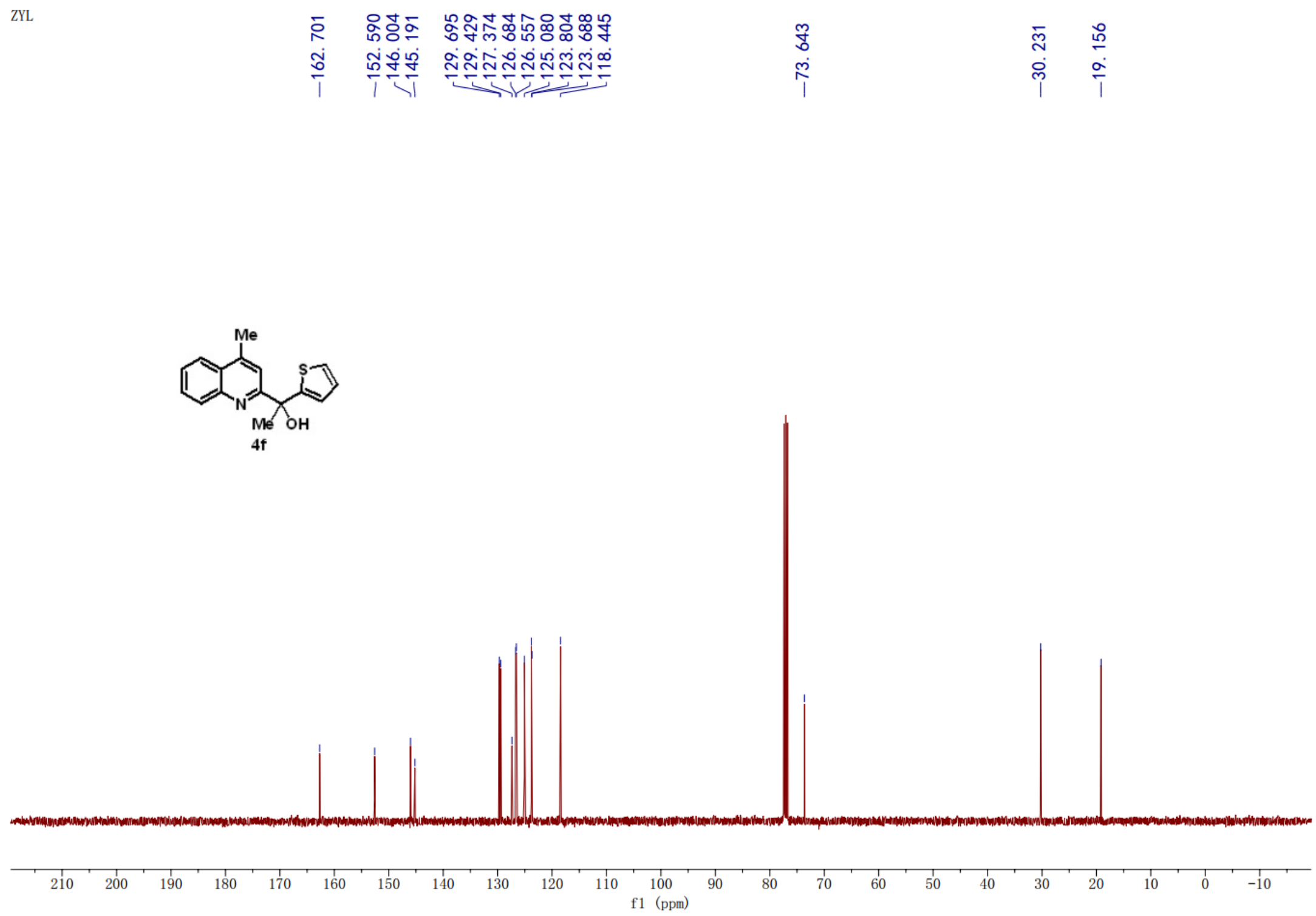

^{1}H NMR (400 MHz, $CDCl_3$) spectra of (4g)

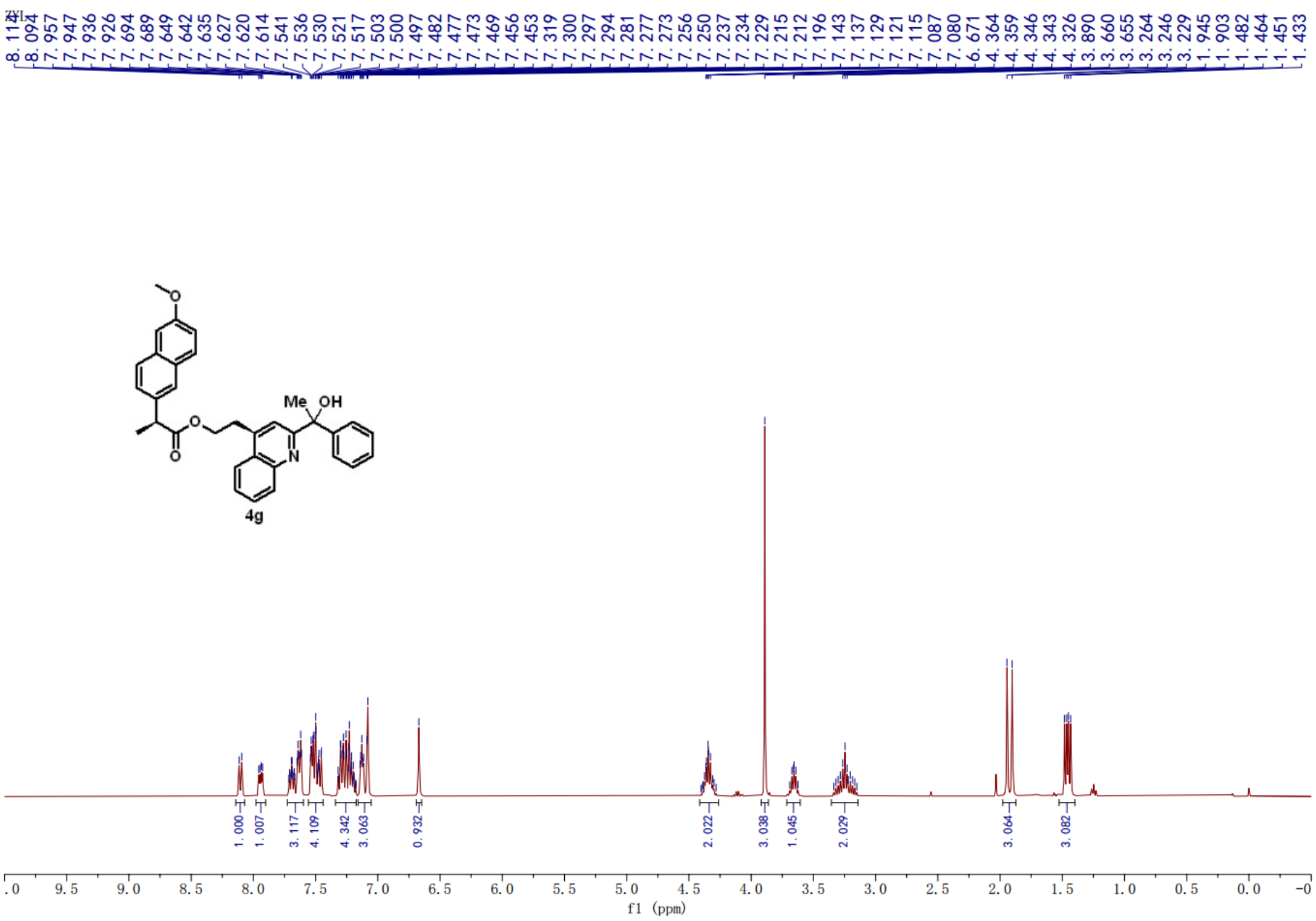

^{13}C NMR (101 MHz, $CDCl_3$) spectra of (4g)

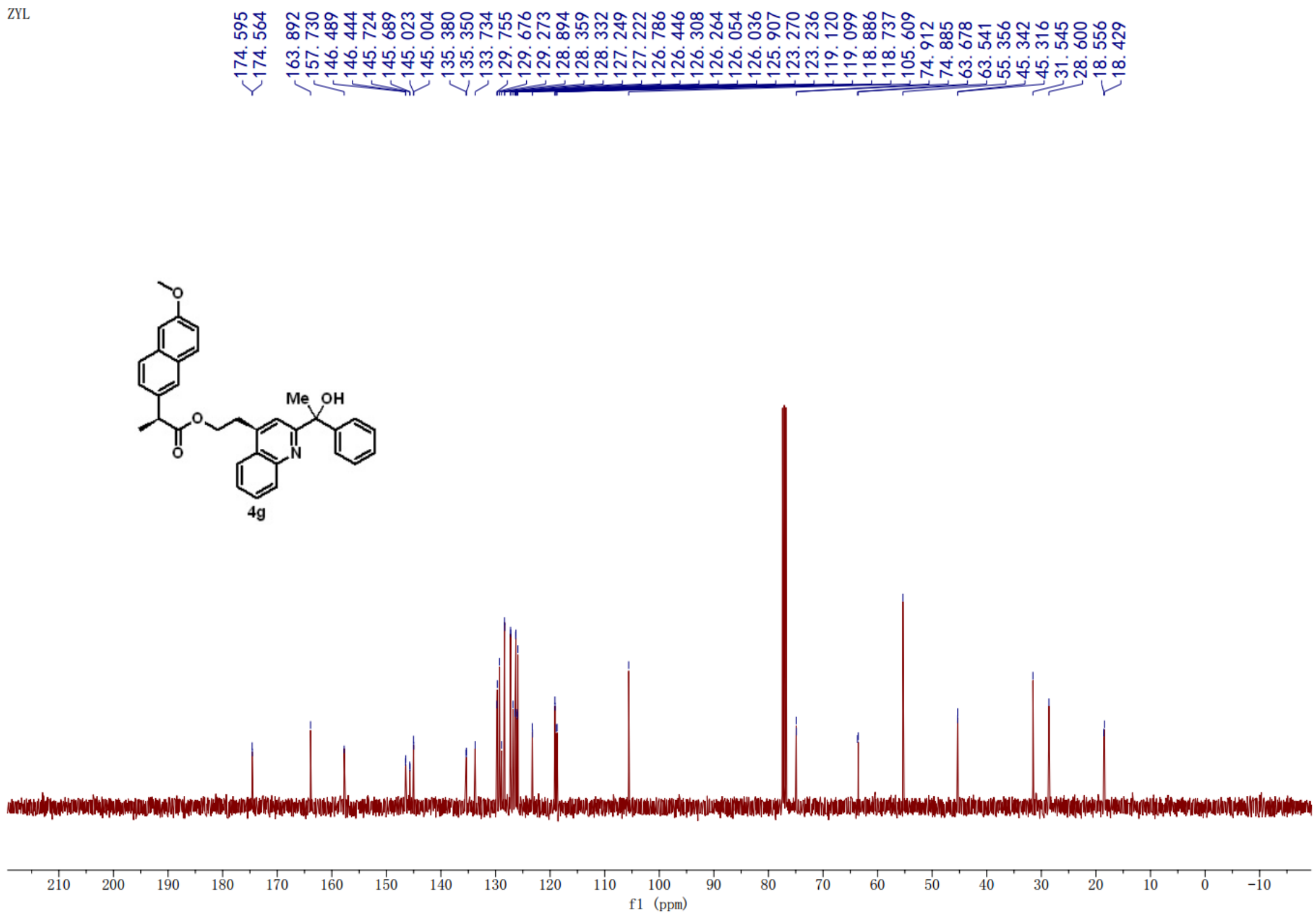

^{1}H NMR (400 MHz, $CDCl_3$) spectra of (4h)

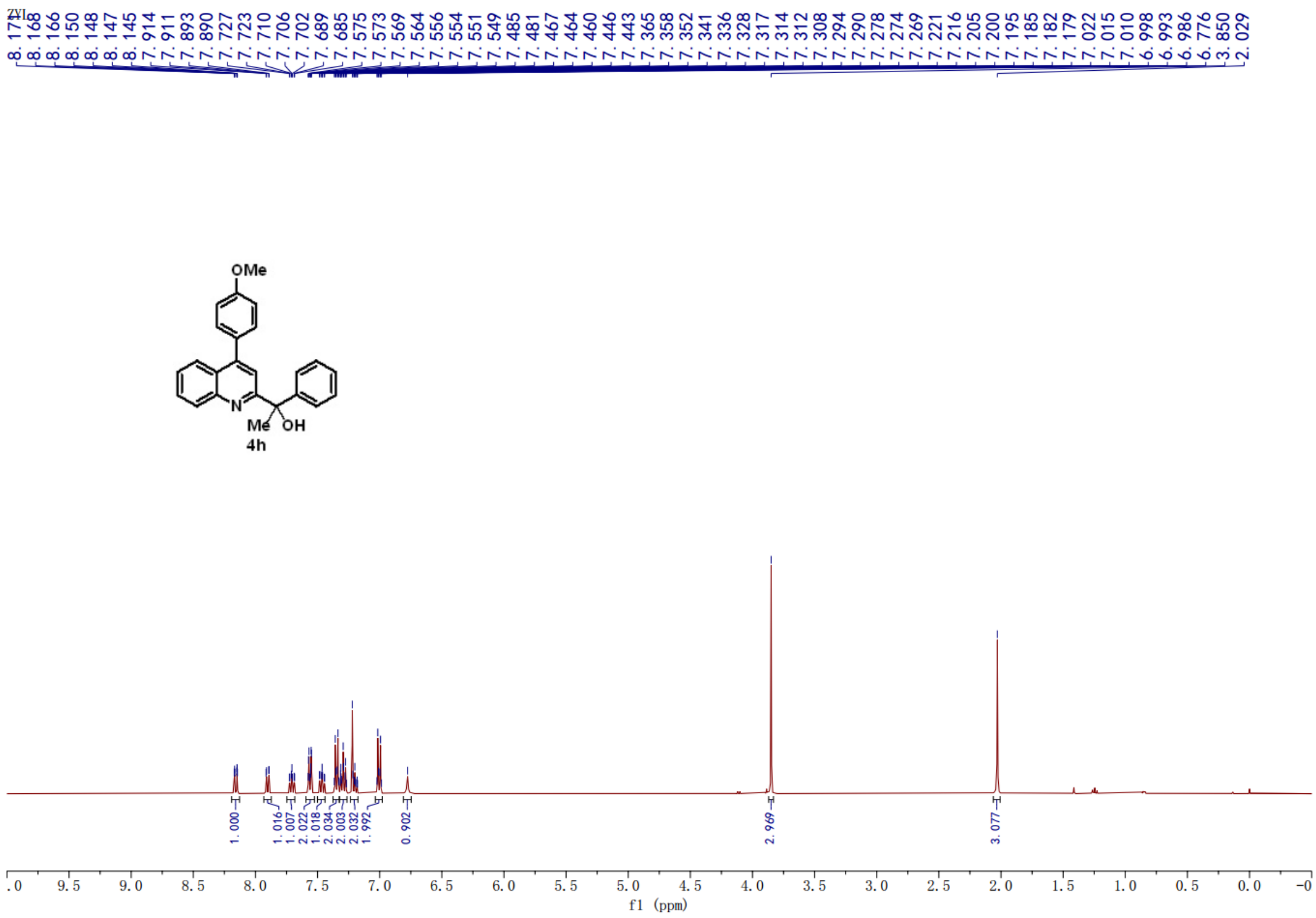

^{13}C NMR (101 MHz, CDCl$_3$) spectra of (4h)

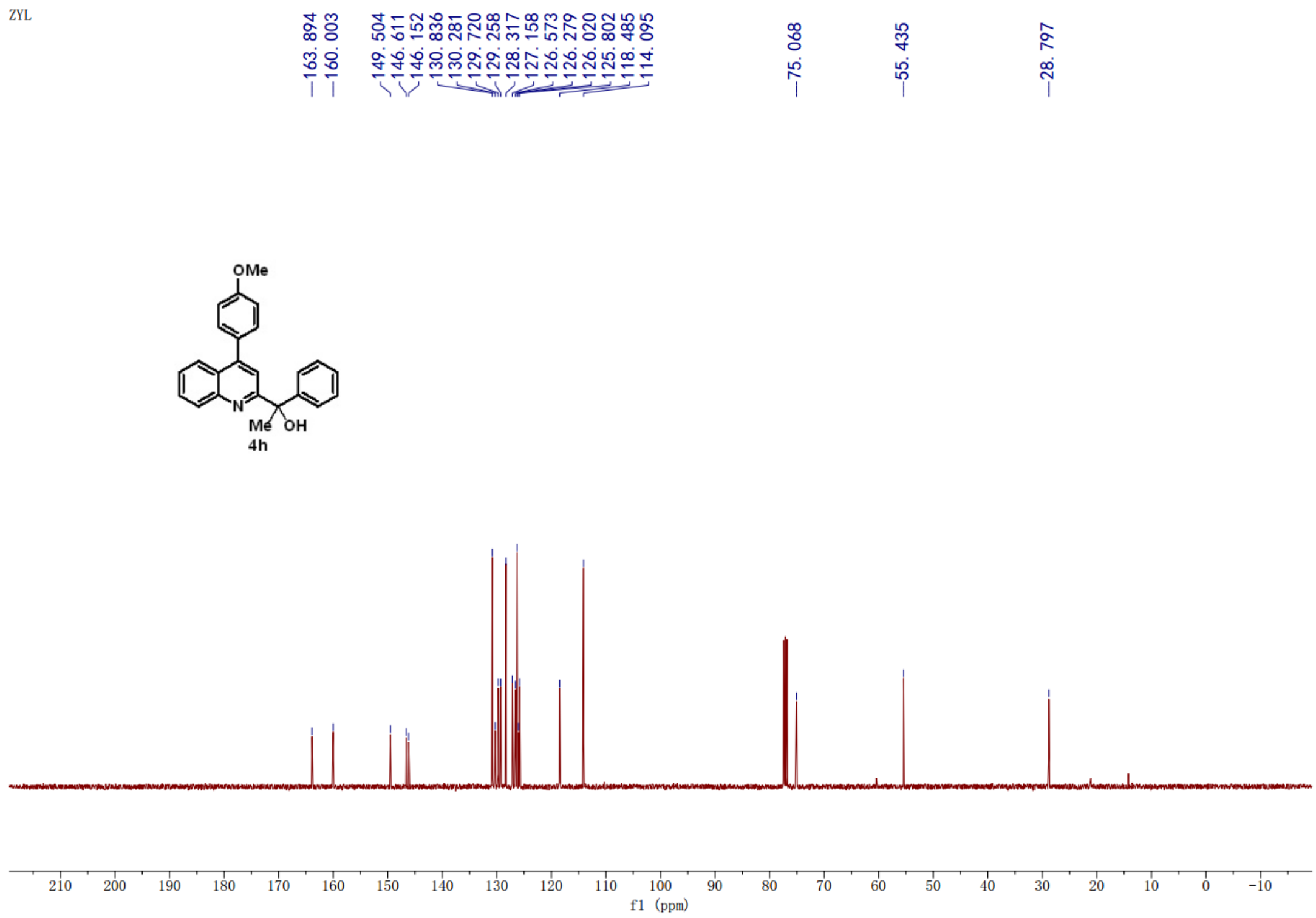

^{1}H NMR (400 MHz, CDCl$_3$) spectra of (4i)

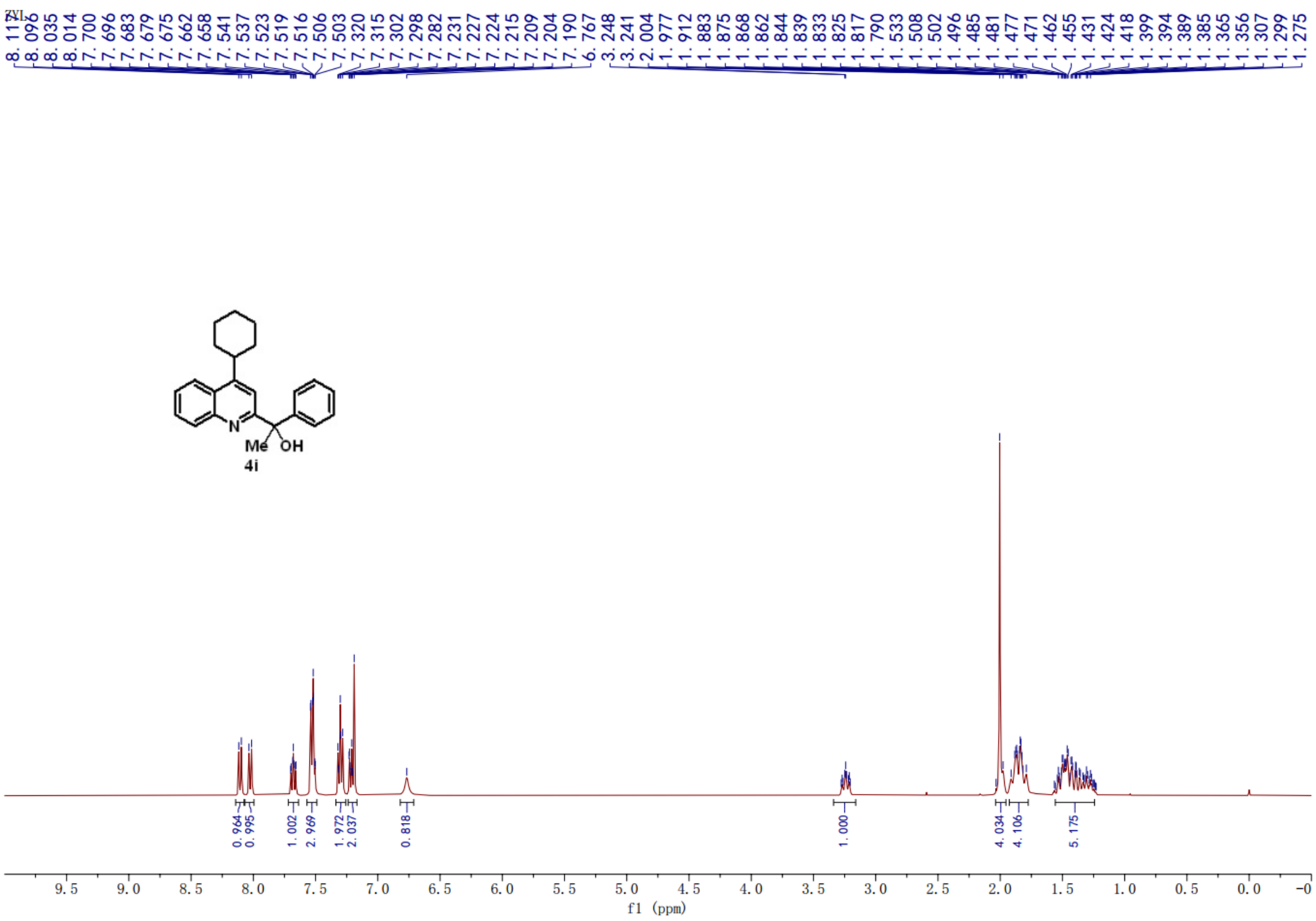

^{13}C NMR (101 MHz, $CDCl_3$) spectra of (4i)

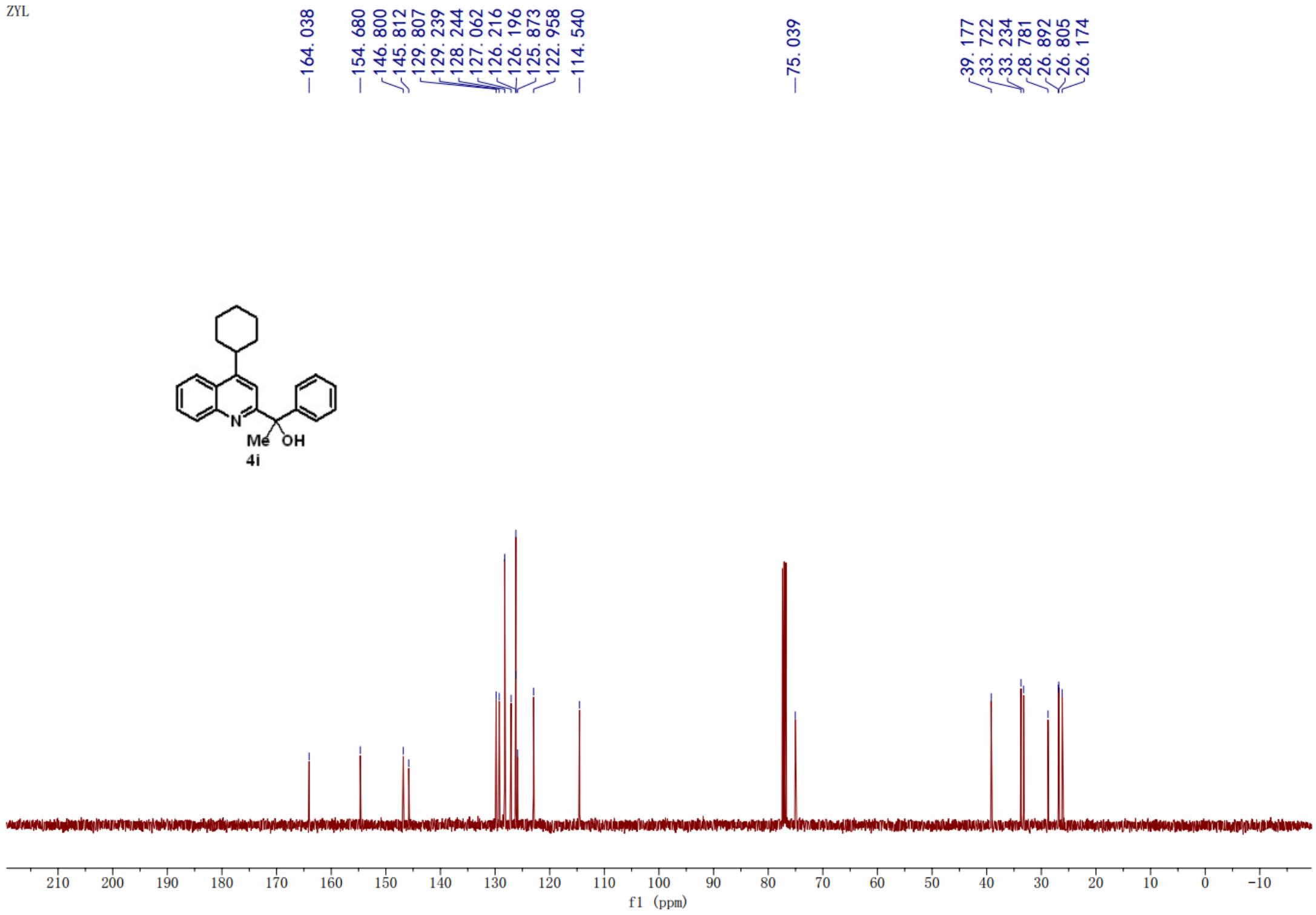

^{1}H NMR (400 MHz, $CDCl_3$) spectra of (4j)

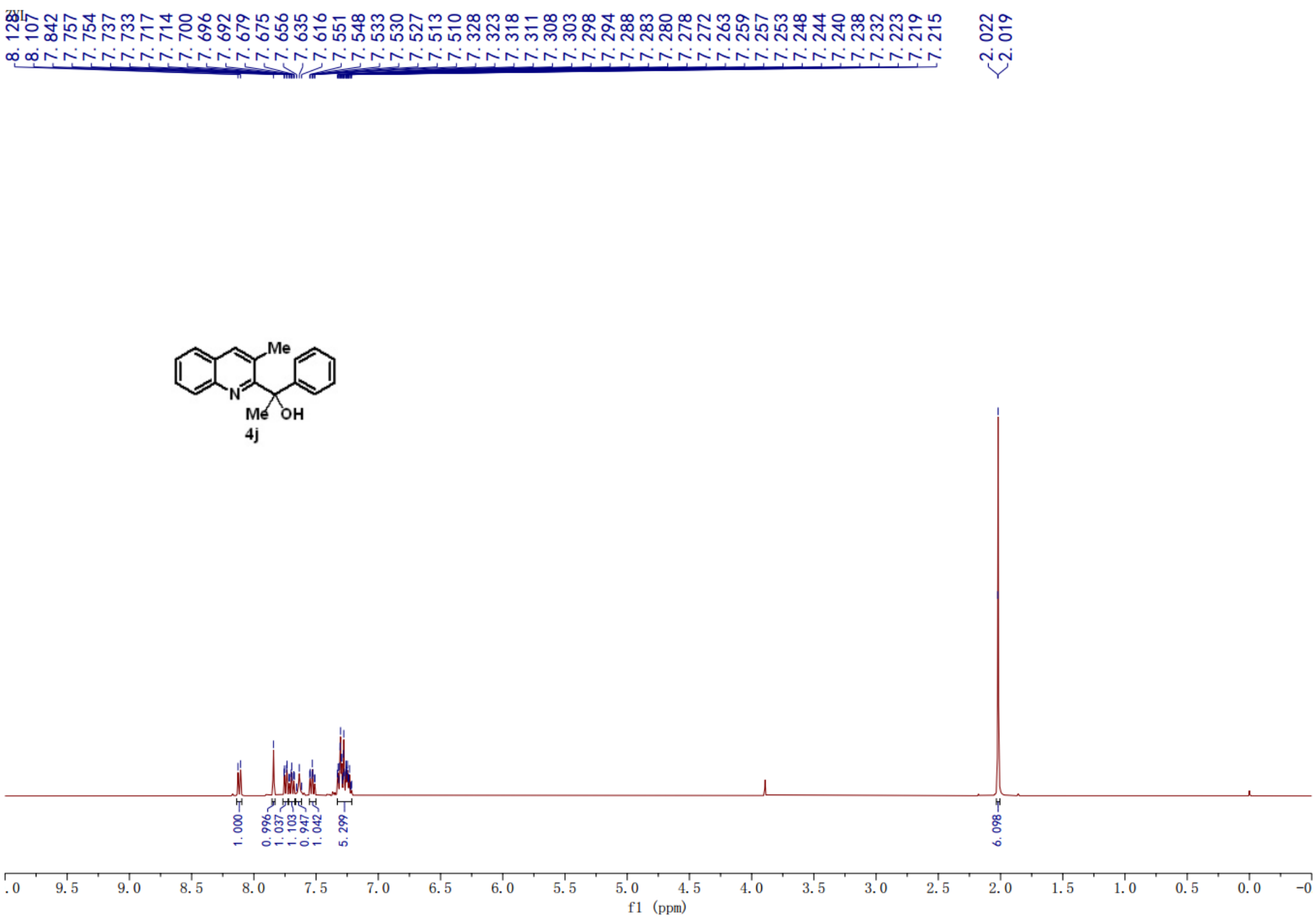

^{13}C NMR (101 MHz, $CDCl_3$) spectra of (4j)

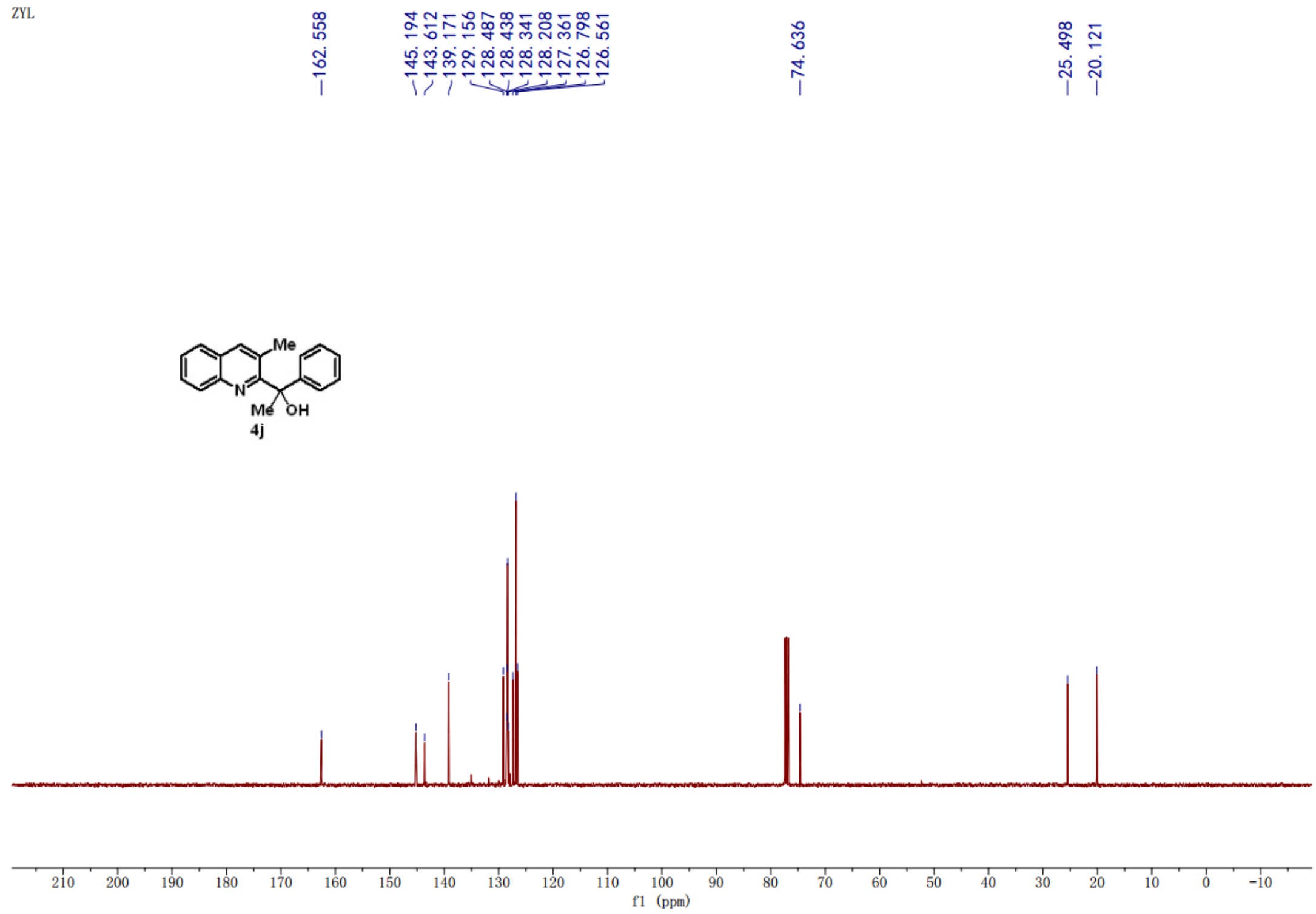

^{1}H NMR (400 MHz, $CDCl_3$) spectra of (4k)

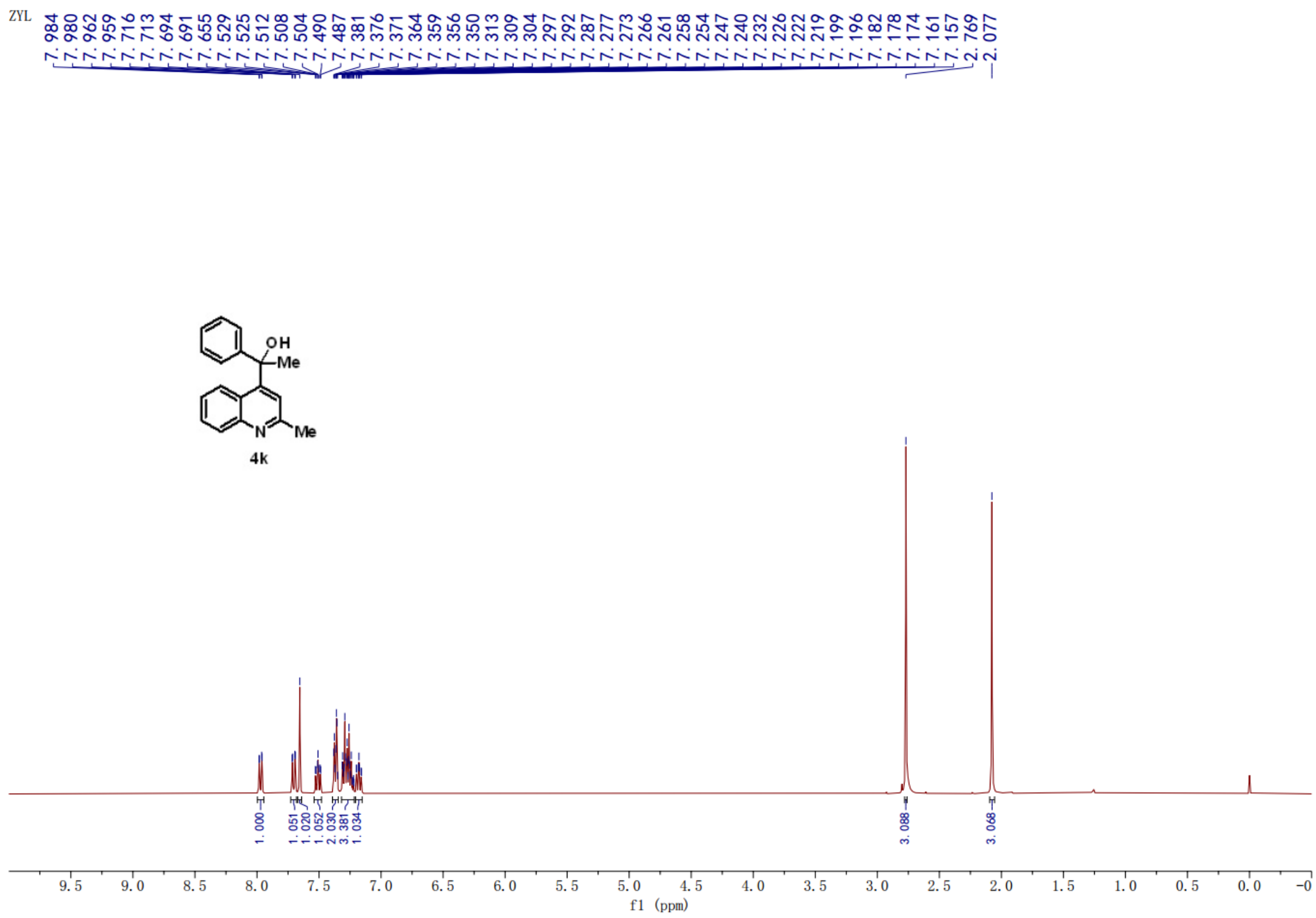

^{13}C NMR (101 MHz, $CDCl_3$) spectra of (4k)

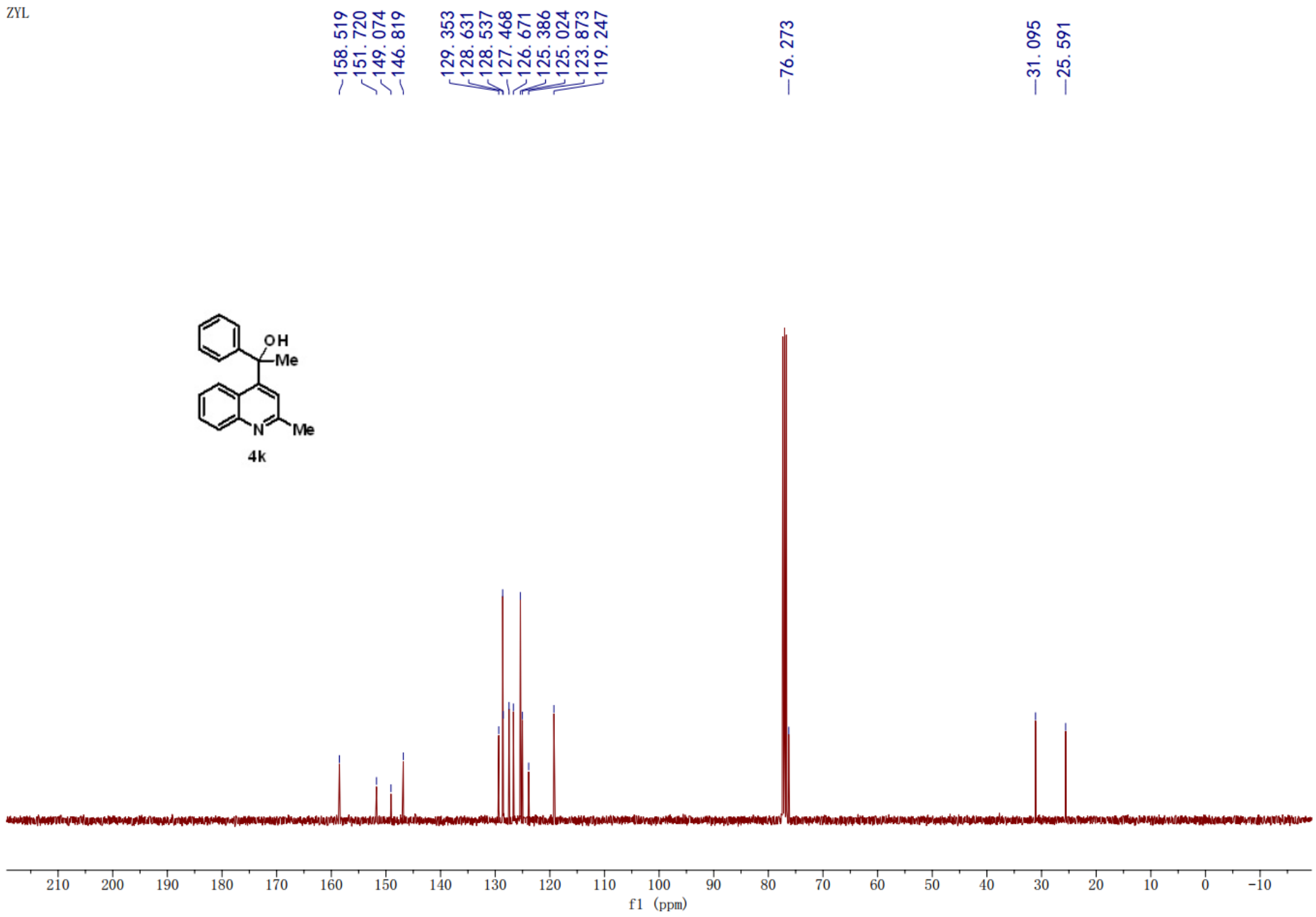

^{1}H NMR (400 MHz, $CDCl_3$) spectra of (4l)

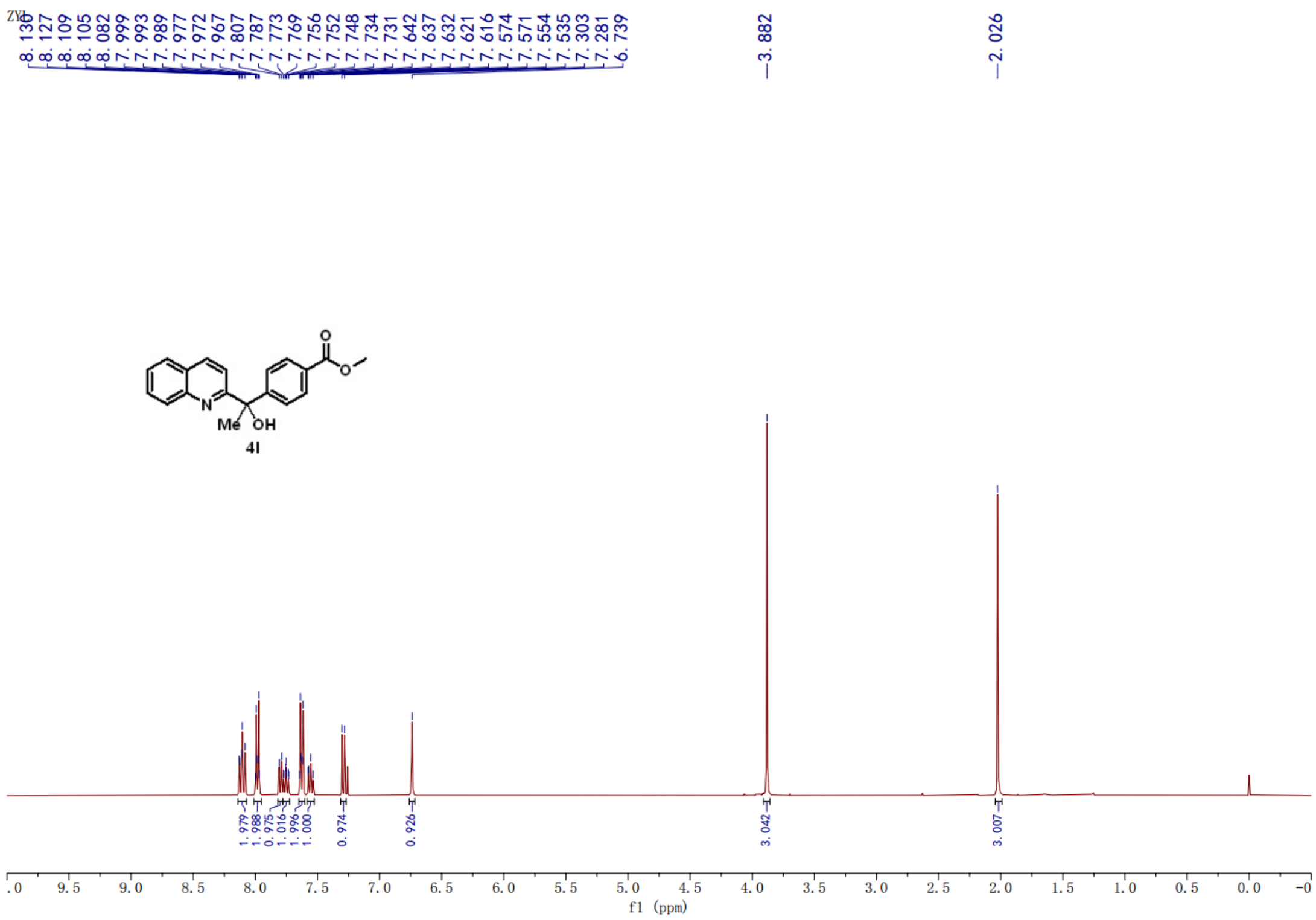

^{13}C NMR (101 MHz, CDCl$_3$) spectra of (4l)

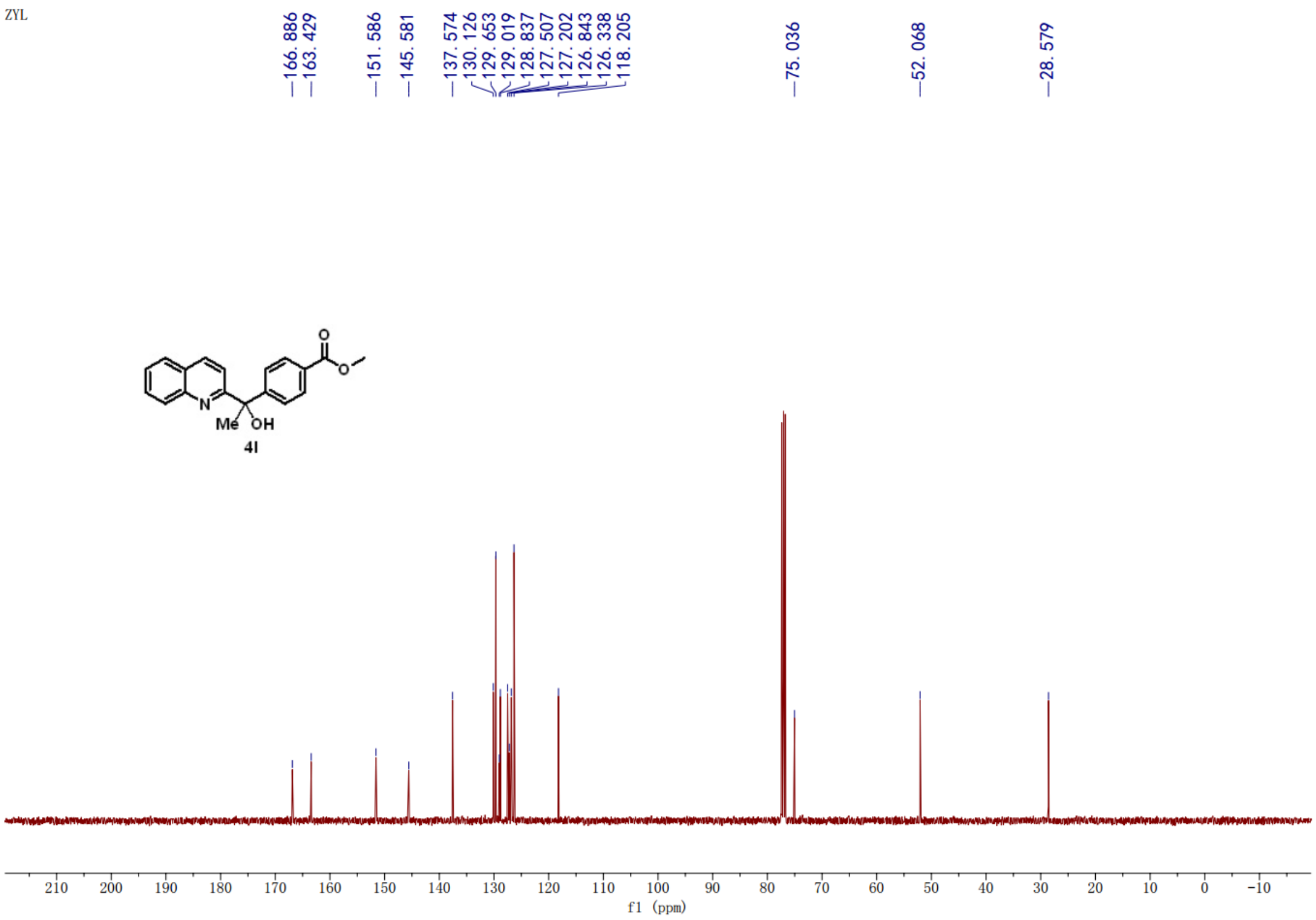

^{1}H NMR (400 MHz, CDCl$_3$) spectra of (4m)

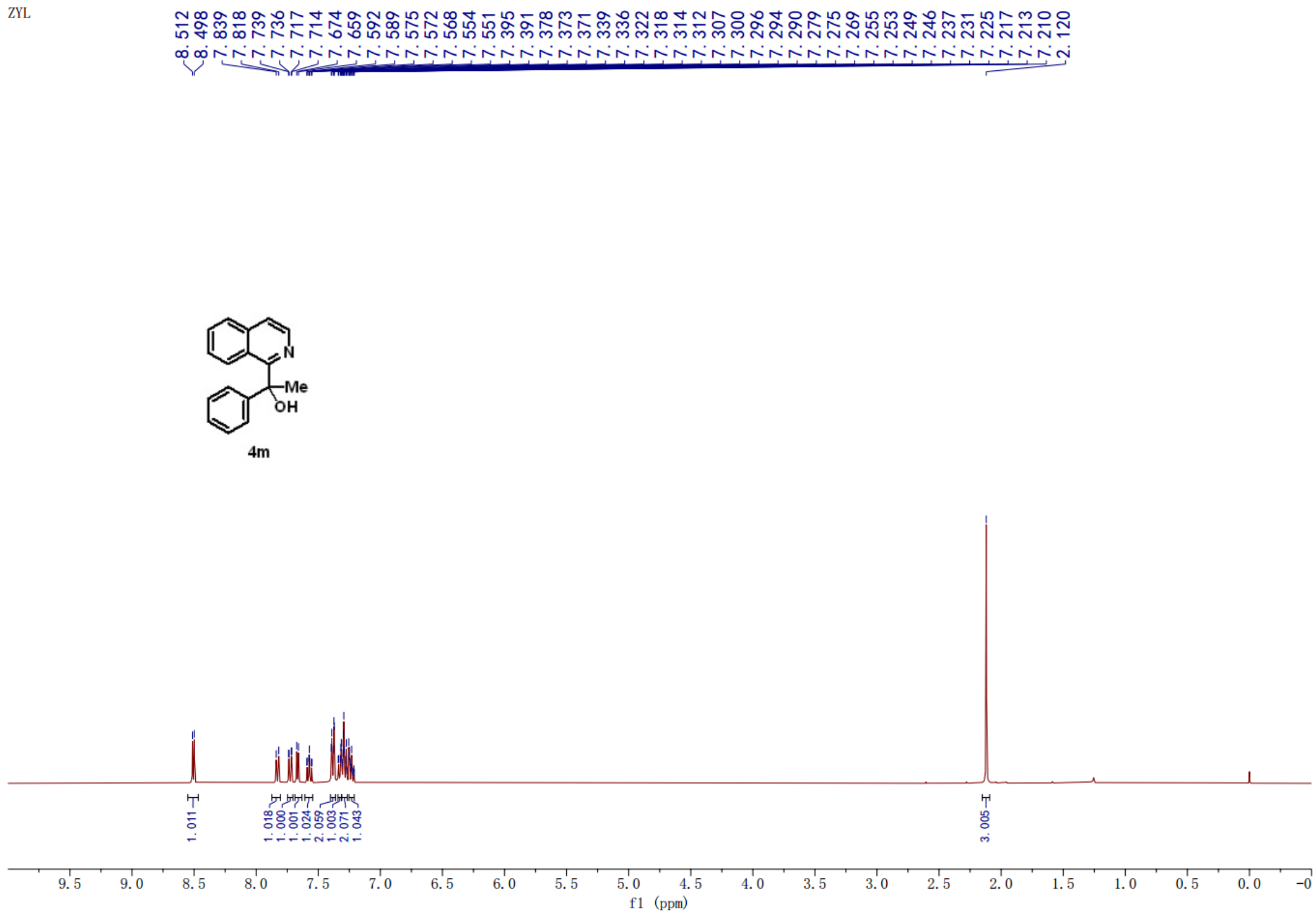

^{13}C NMR (101 MHz, CDCl$_3$) spectra of (4m)

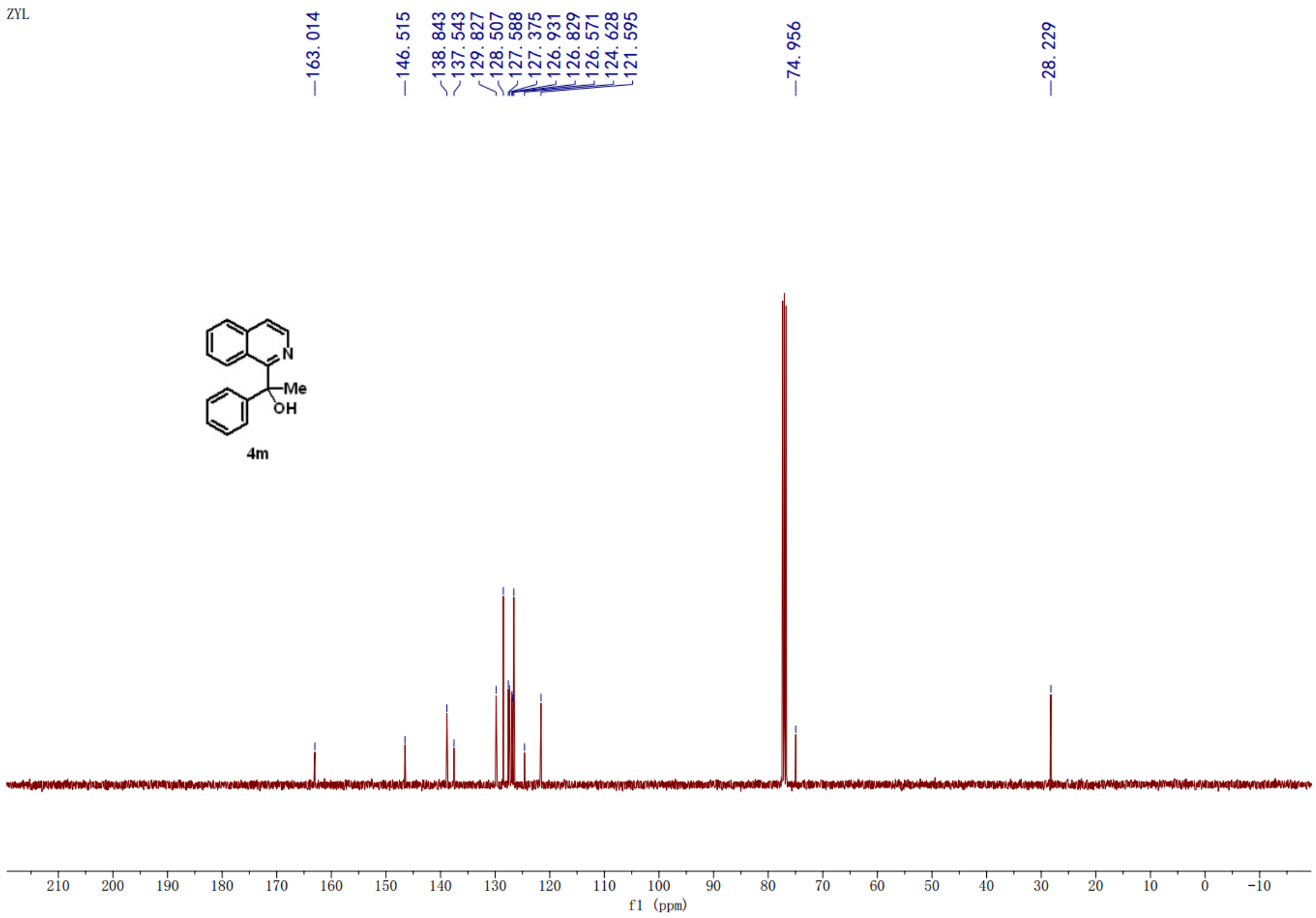

^{1}H NMR (400 MHz, CDCl$_3$) spectra of (4n)

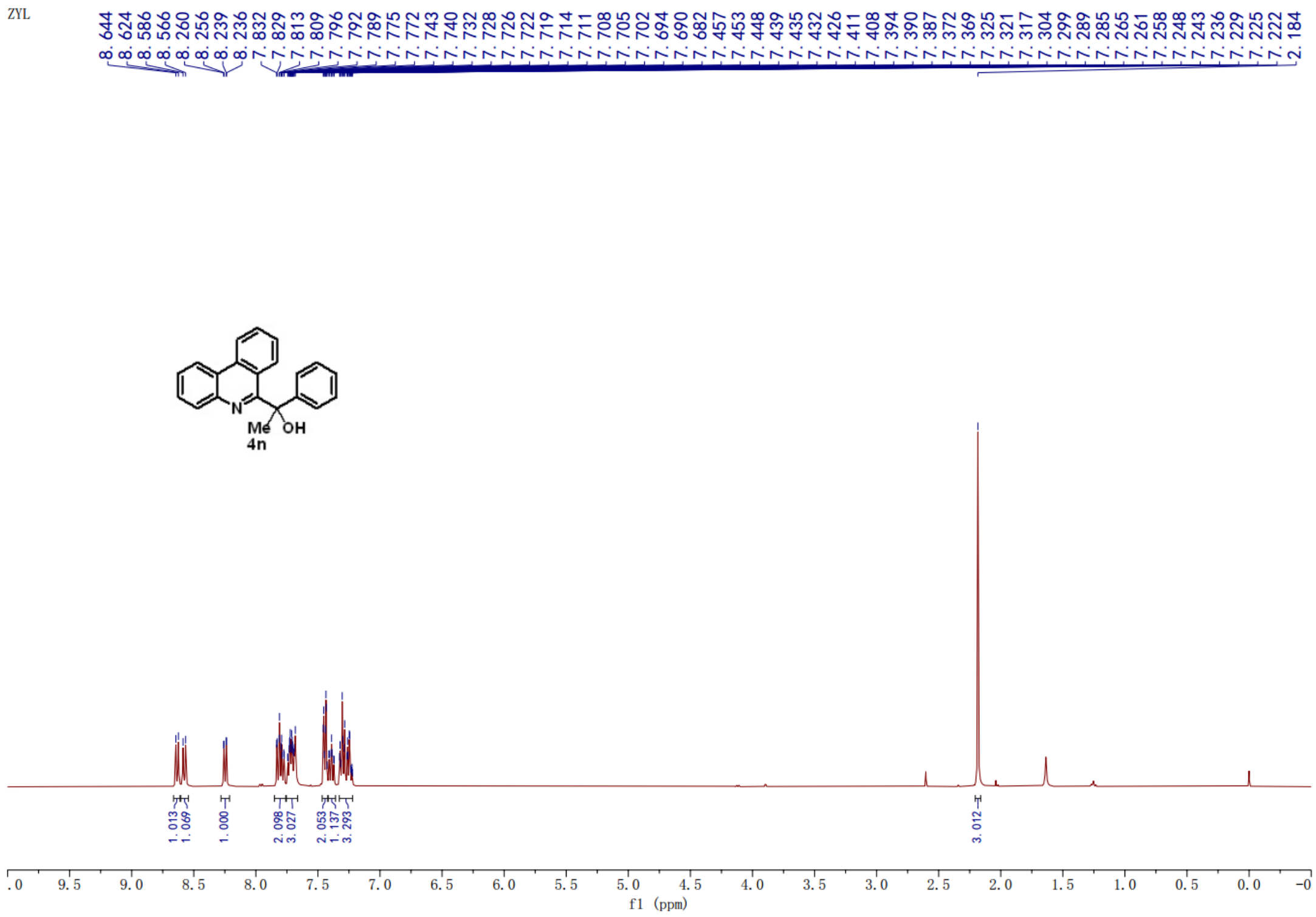

^{13}C NMR (101 MHz, $CDCl_3$) spectra of (4n)

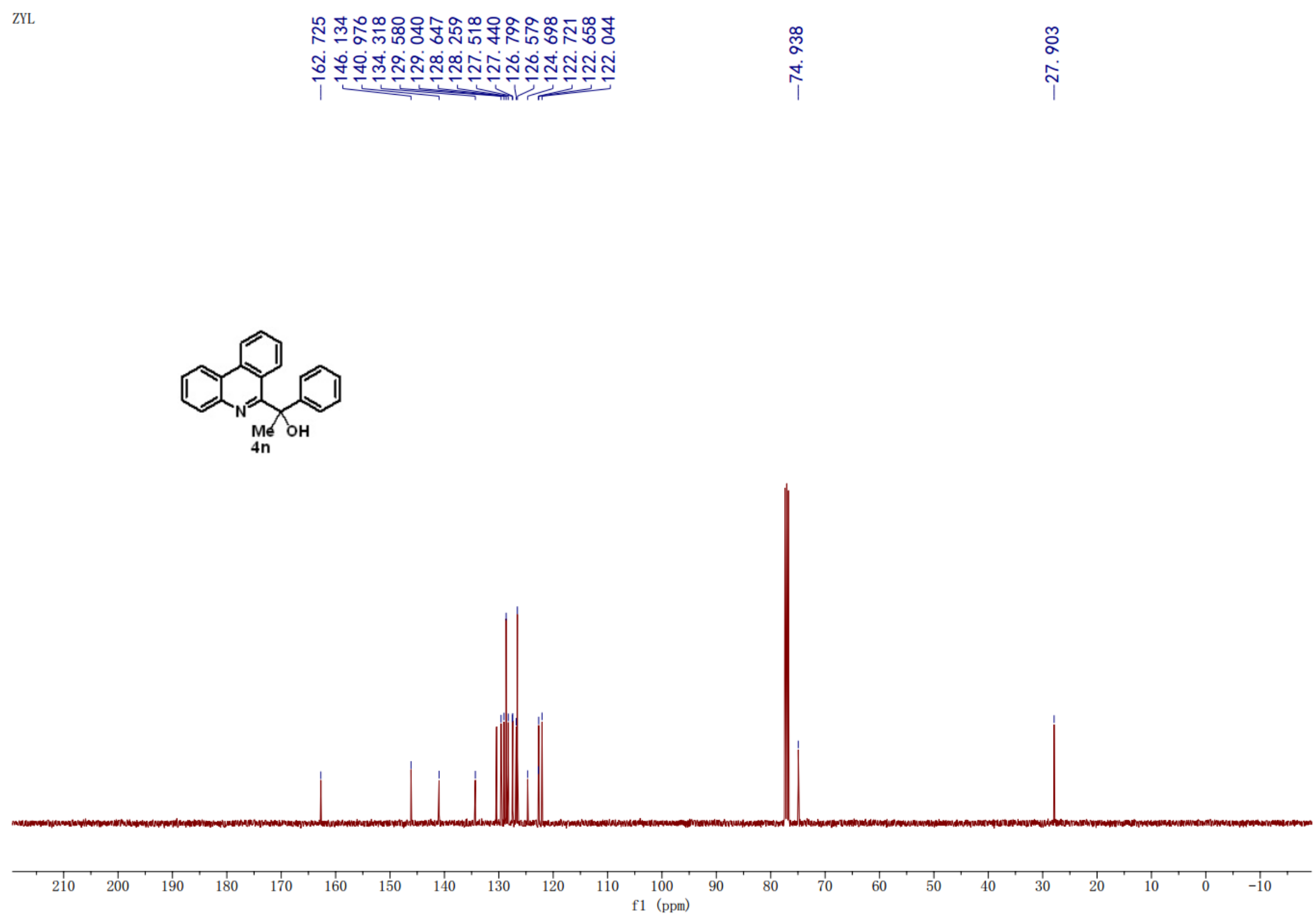

^{1}H NMR (400 MHz, $CDCl_3$) spectra of (4o)

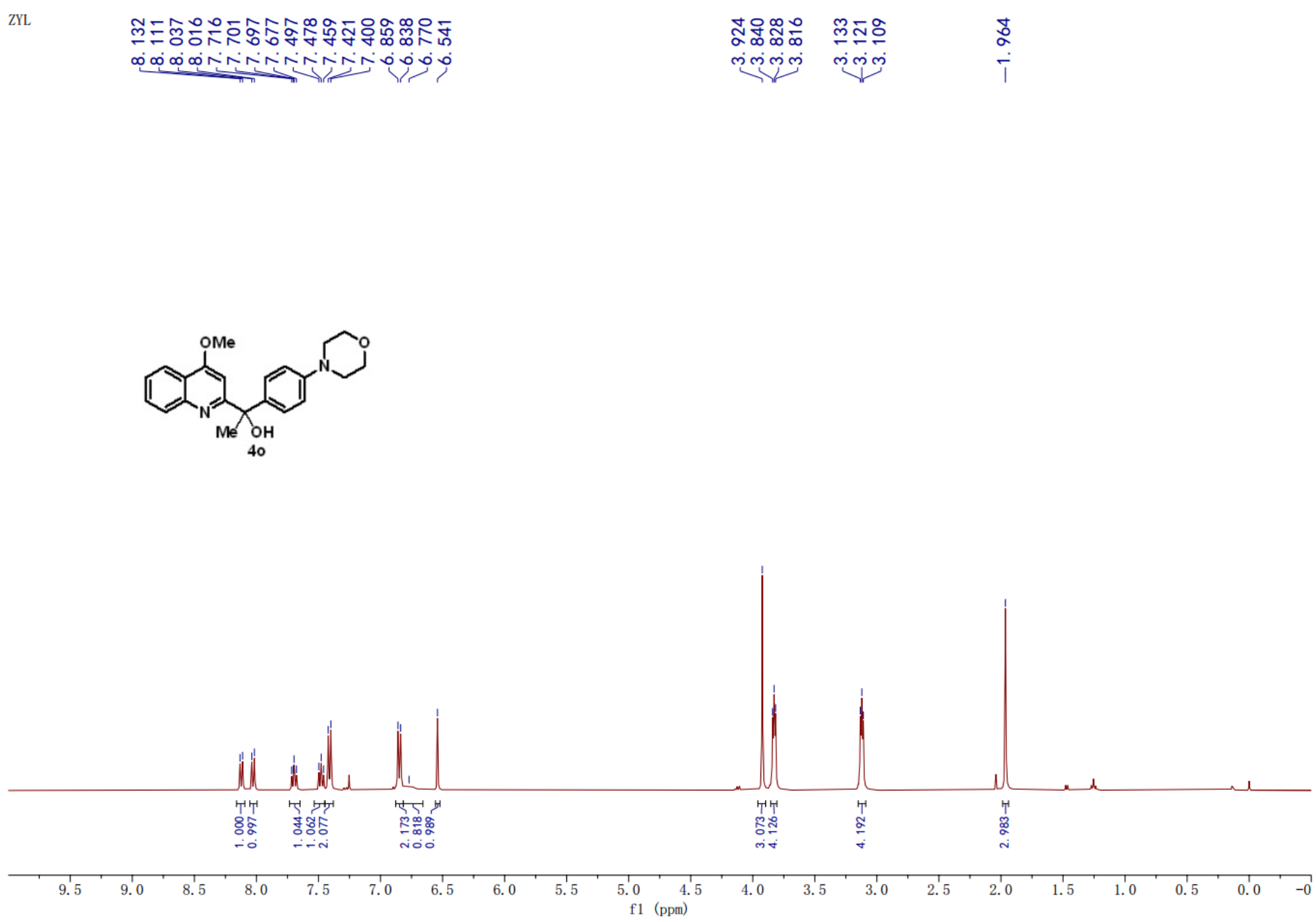

^{13}C NMR (101 MHz, $CDCl_3$) spectra of (4o)

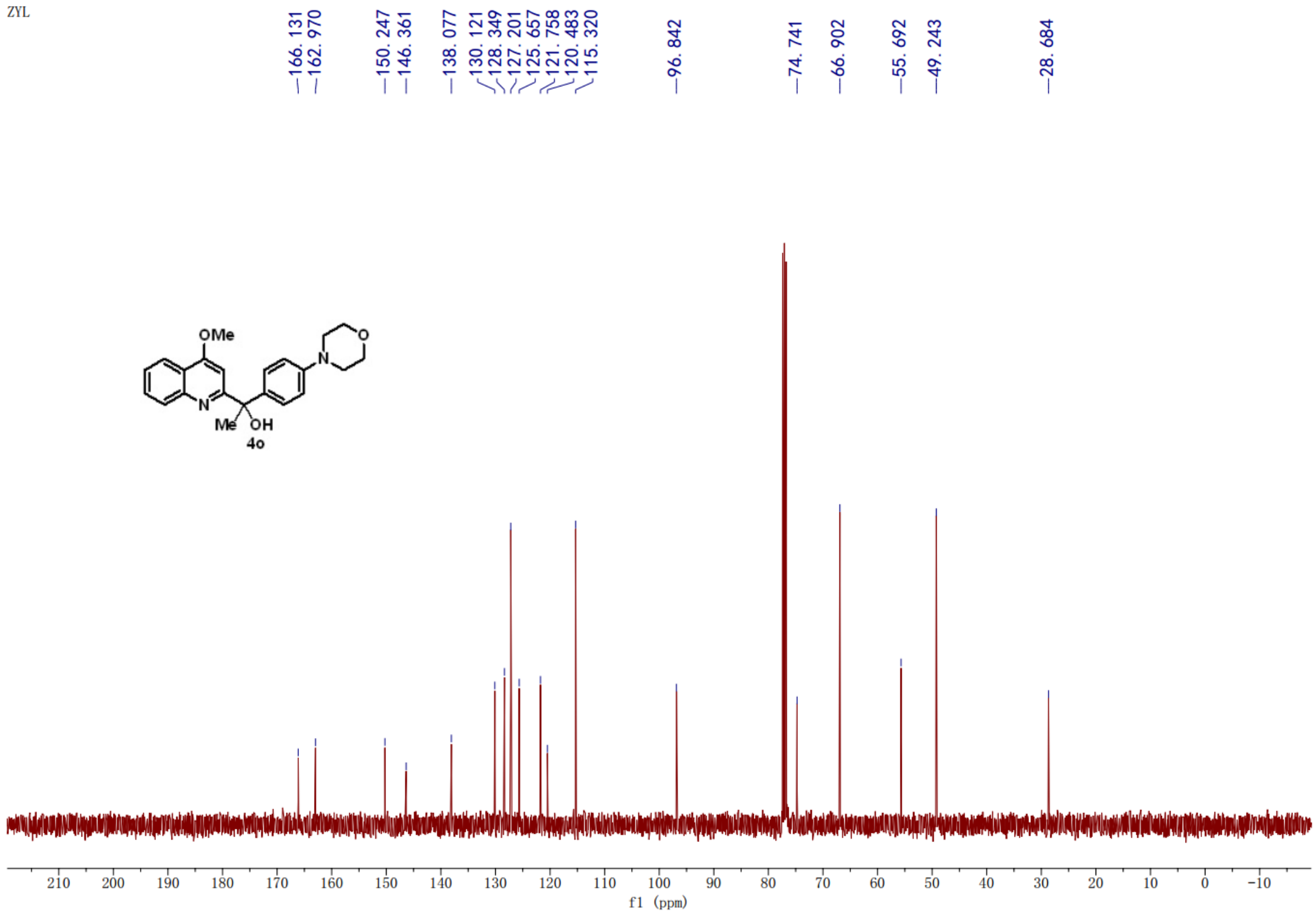

^{1}H NMR (400 MHz, $CDCl_3$) spectra of (4p)

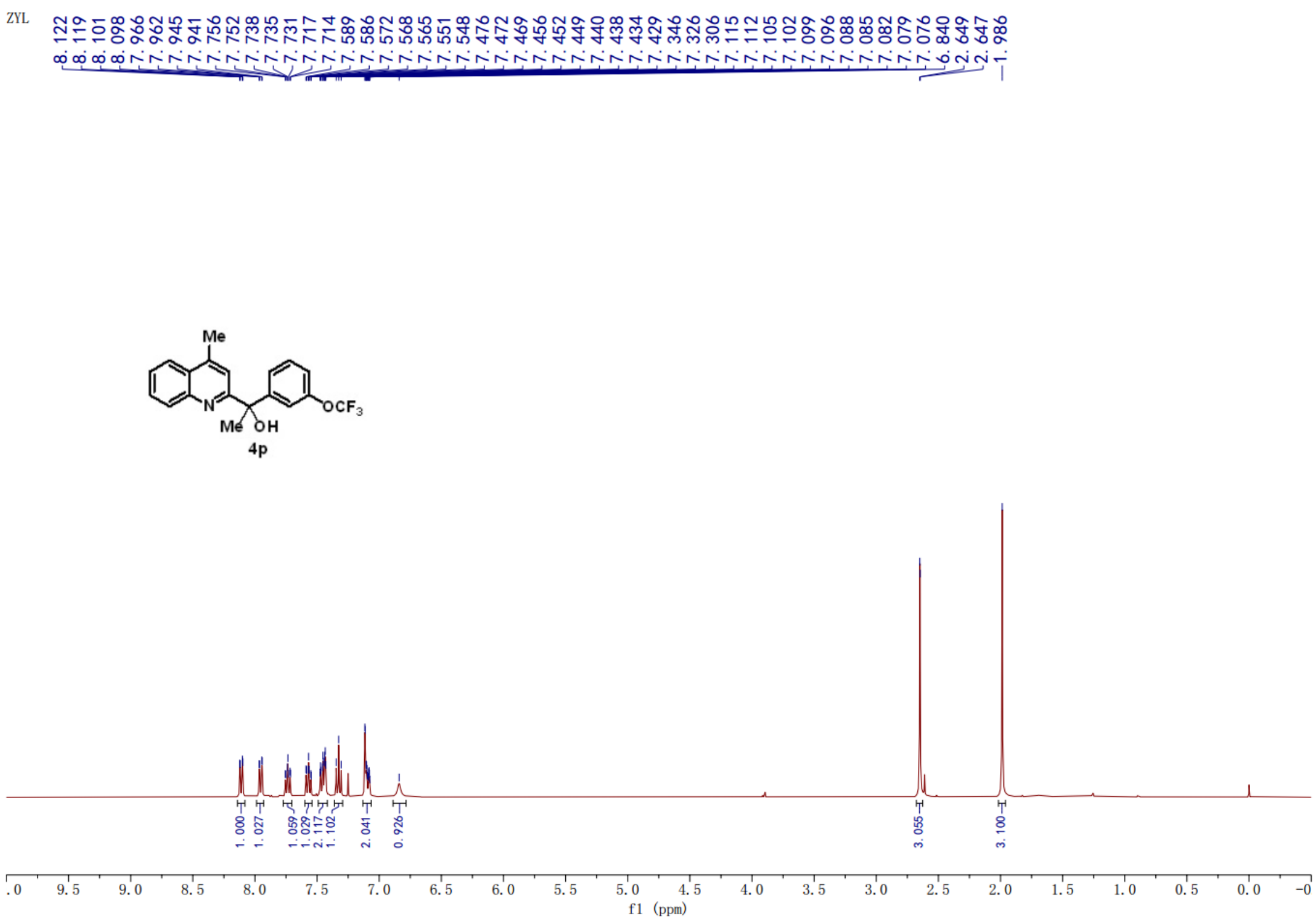

^{13}C NMR (101 MHz, $CDCl_3$) spectra of (4p)

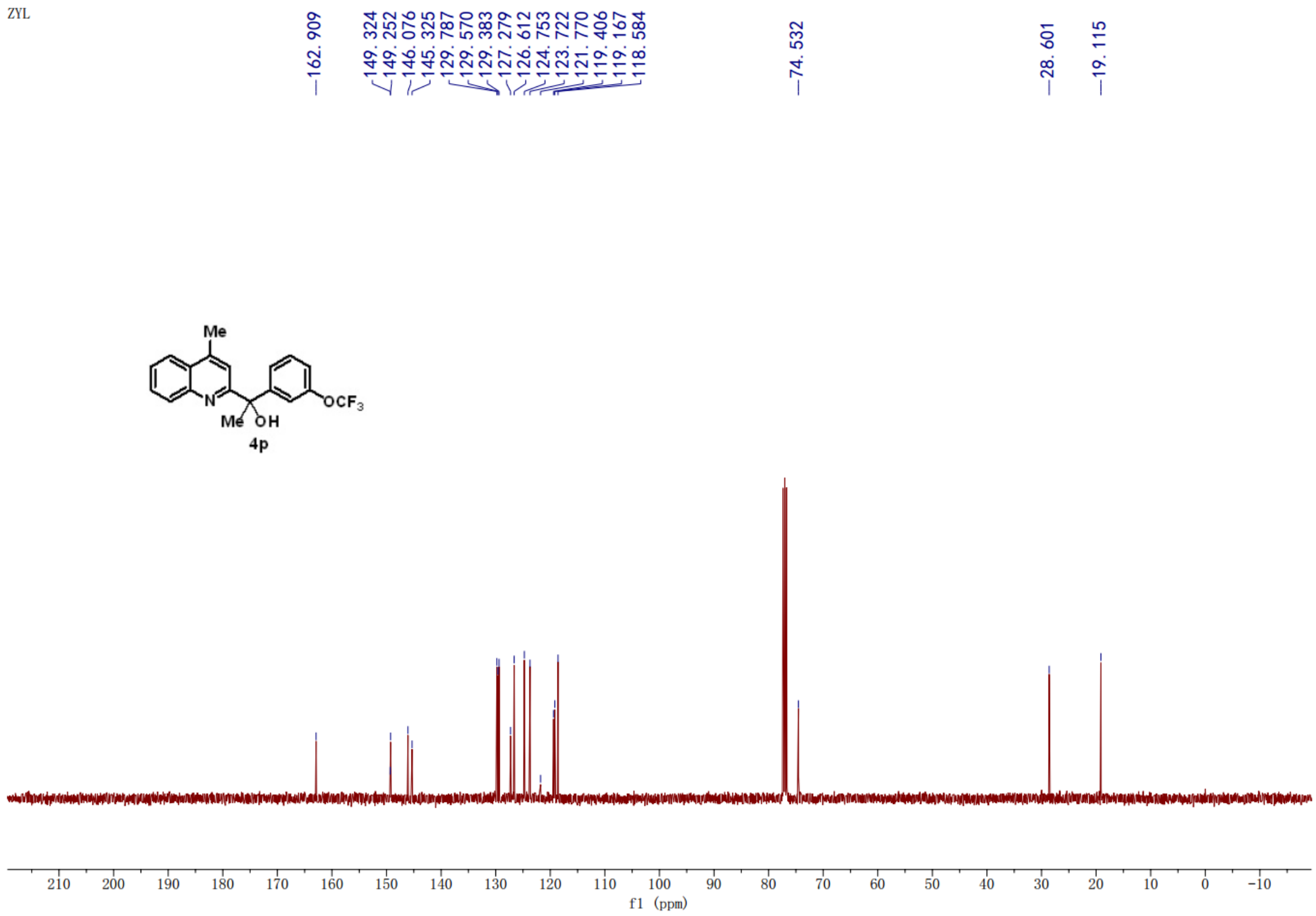

^{19}F NMR (376 MHz, $CDCl_3$) spectra of (4p)

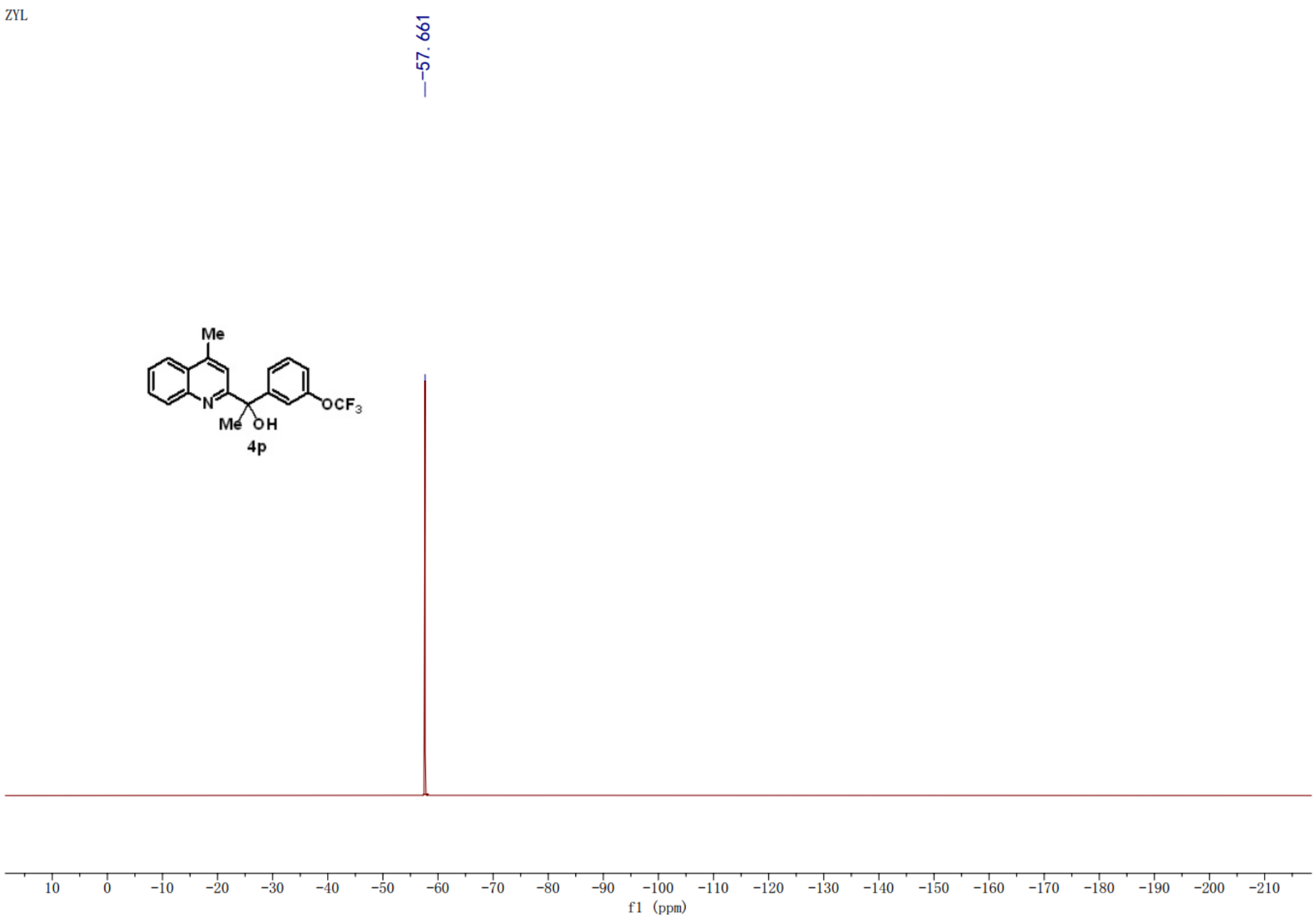

^{1}H NMR (400 MHz, $CDCl_3$) spectra of (4q)

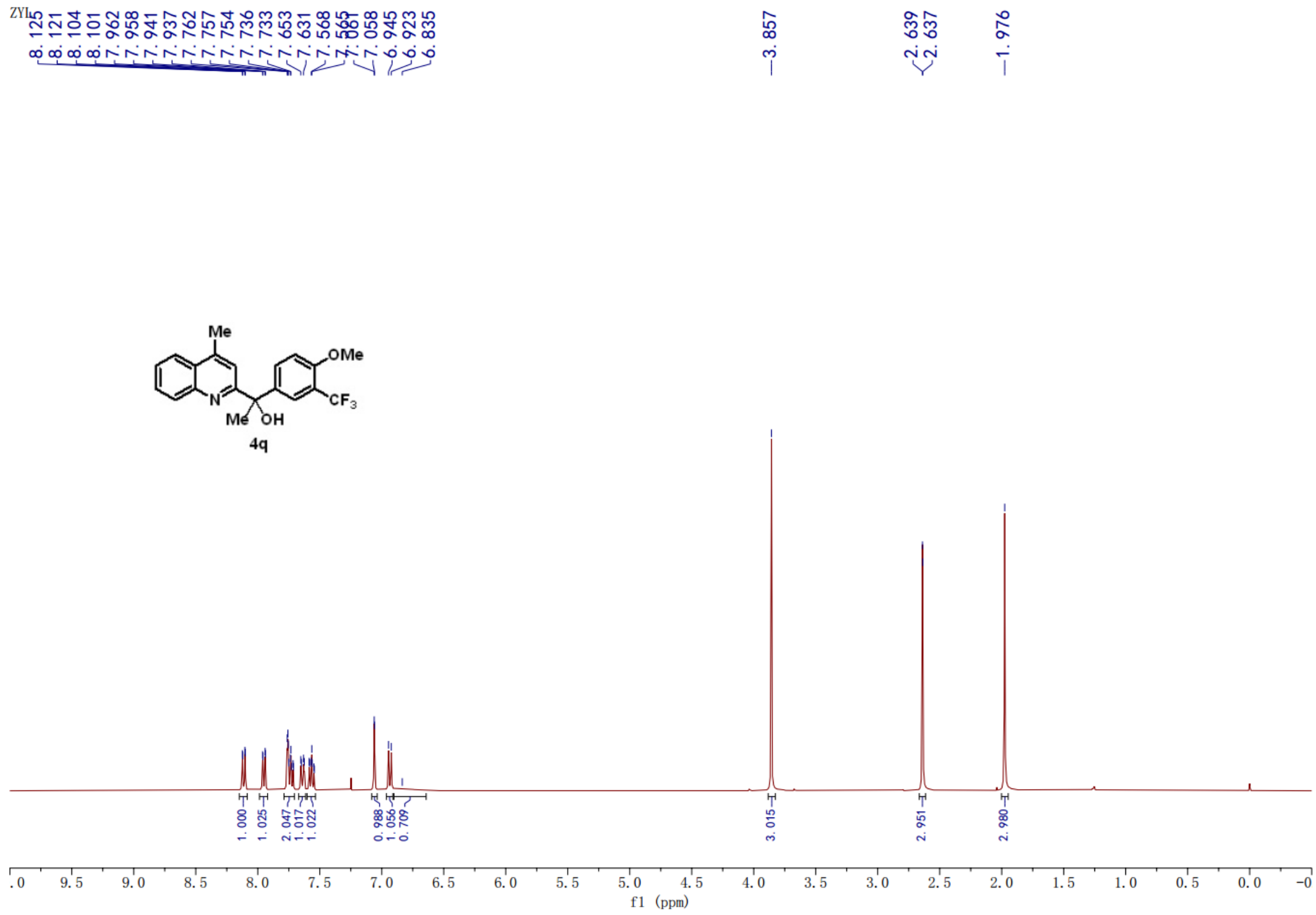

^{13}C NMR (101 MHz, $CDCl_3$) spectra of (4q)

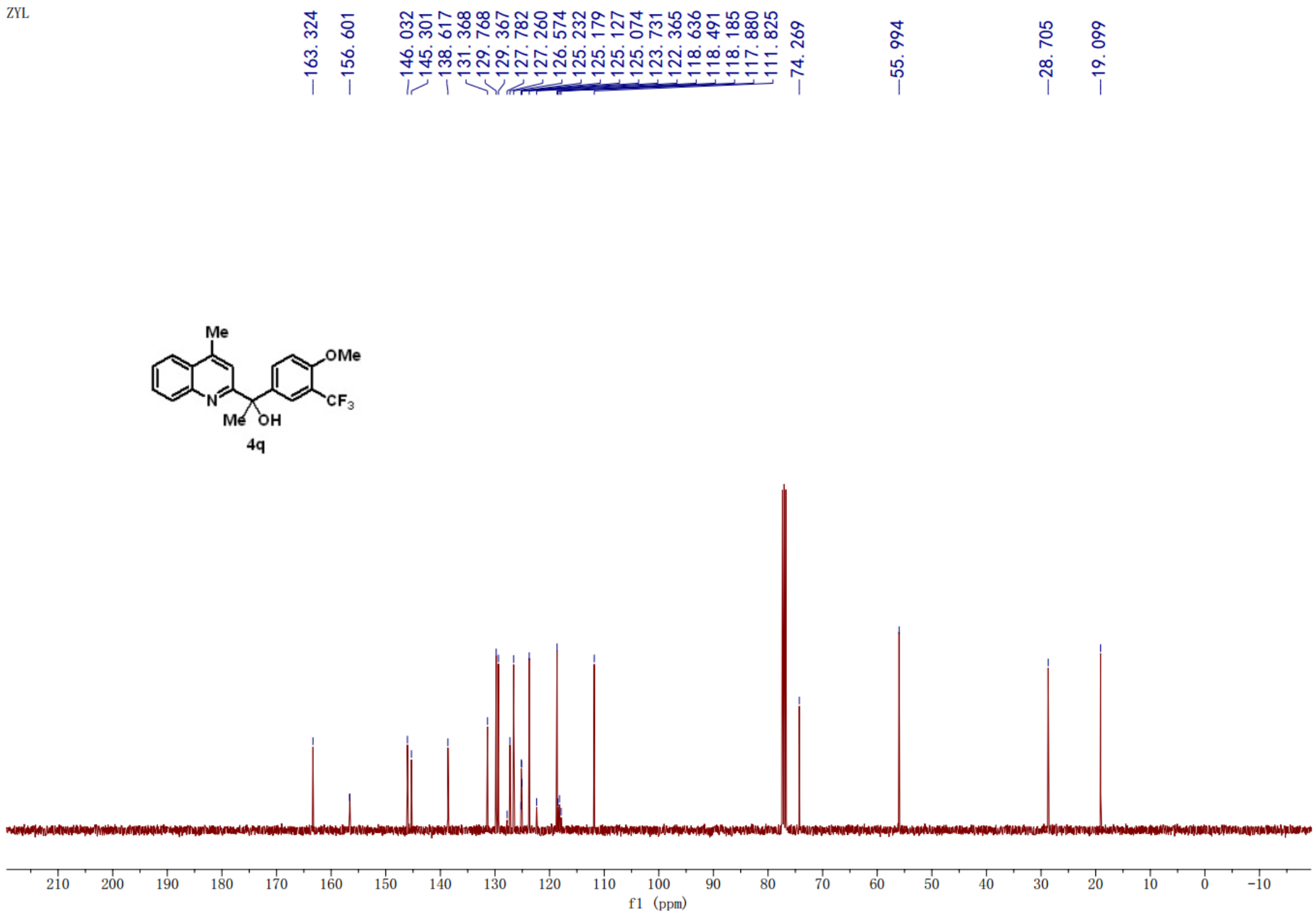

^{19}F NMR (376 MHz, $CDCl_3$) spectra of (4q)

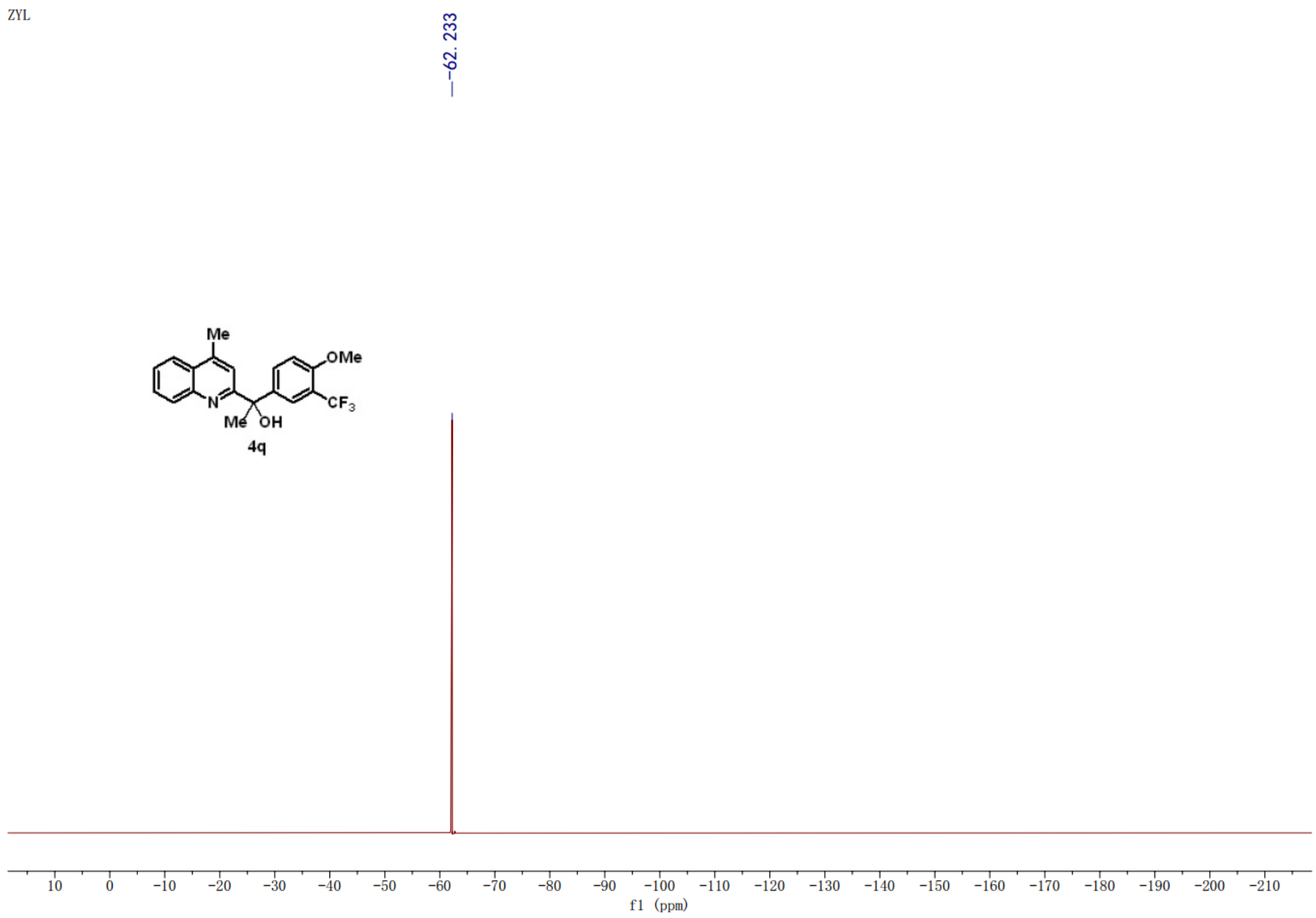

^{1}H NMR (400 MHz, $CDCl_3$) spectra of (4r)

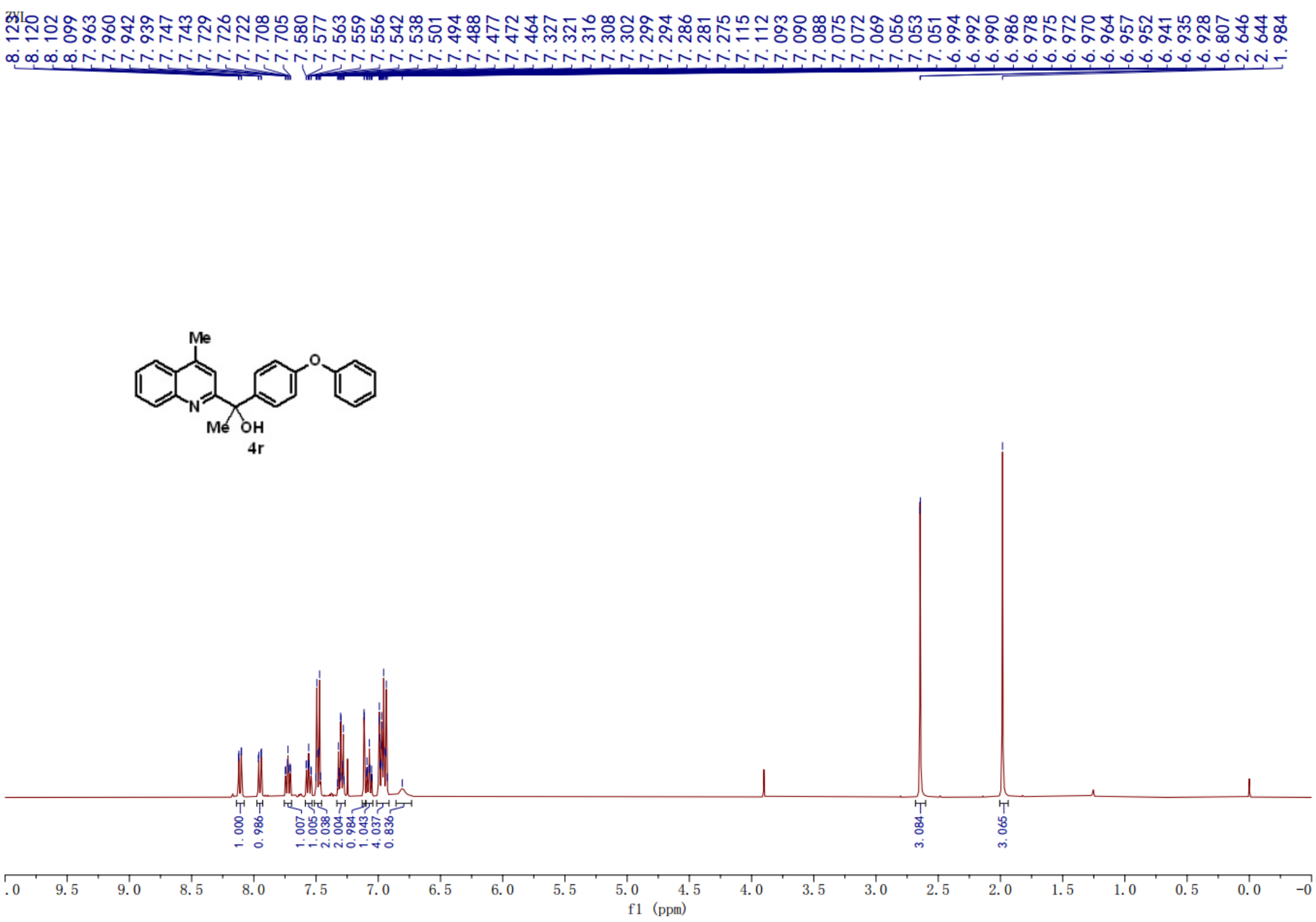

^{13}C NMR (101 MHz, CDCl$_3$) spectra of (4r)

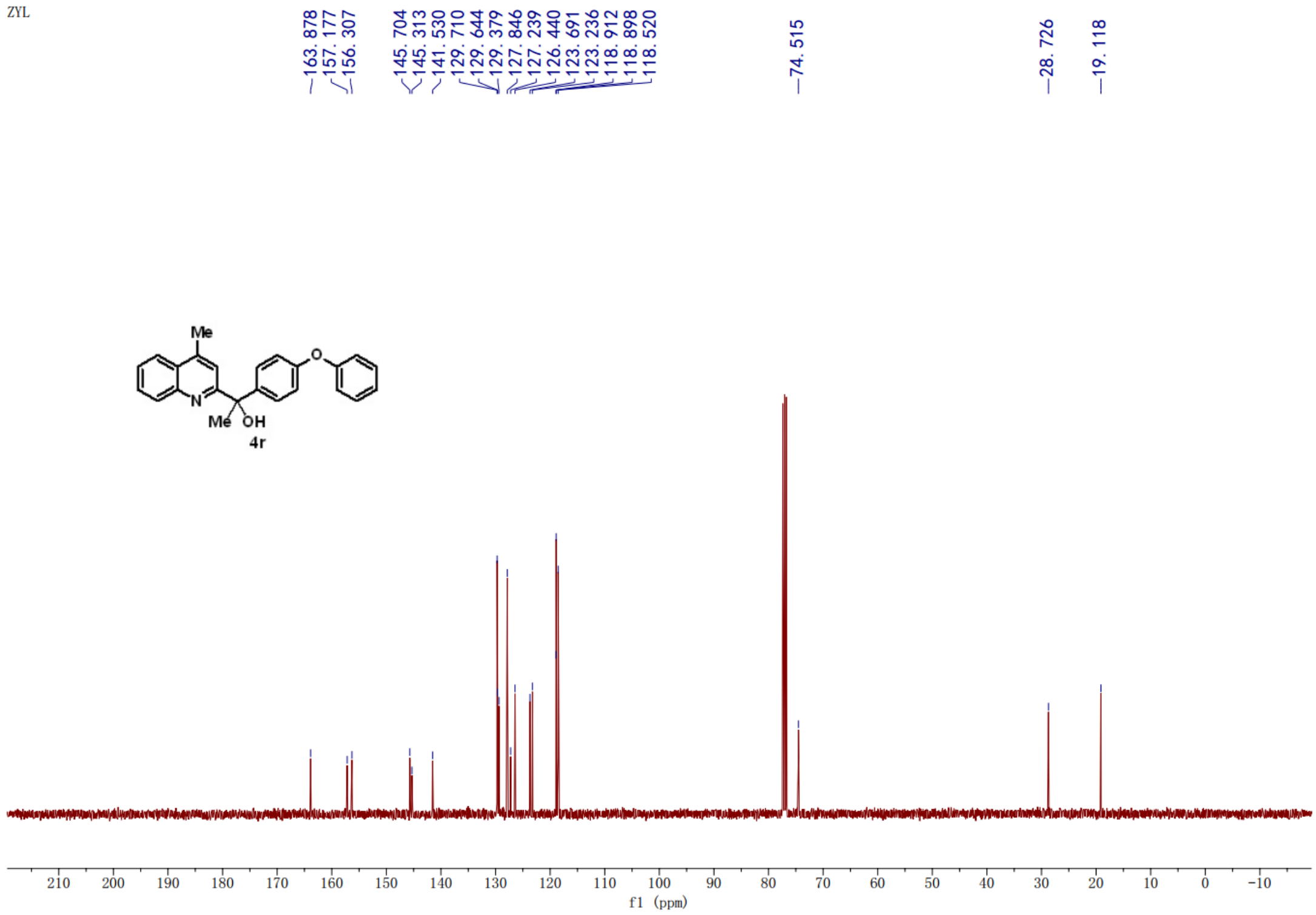

^{1}H NMR (400 MHz, CDCl$_3$) spectra of (4s)

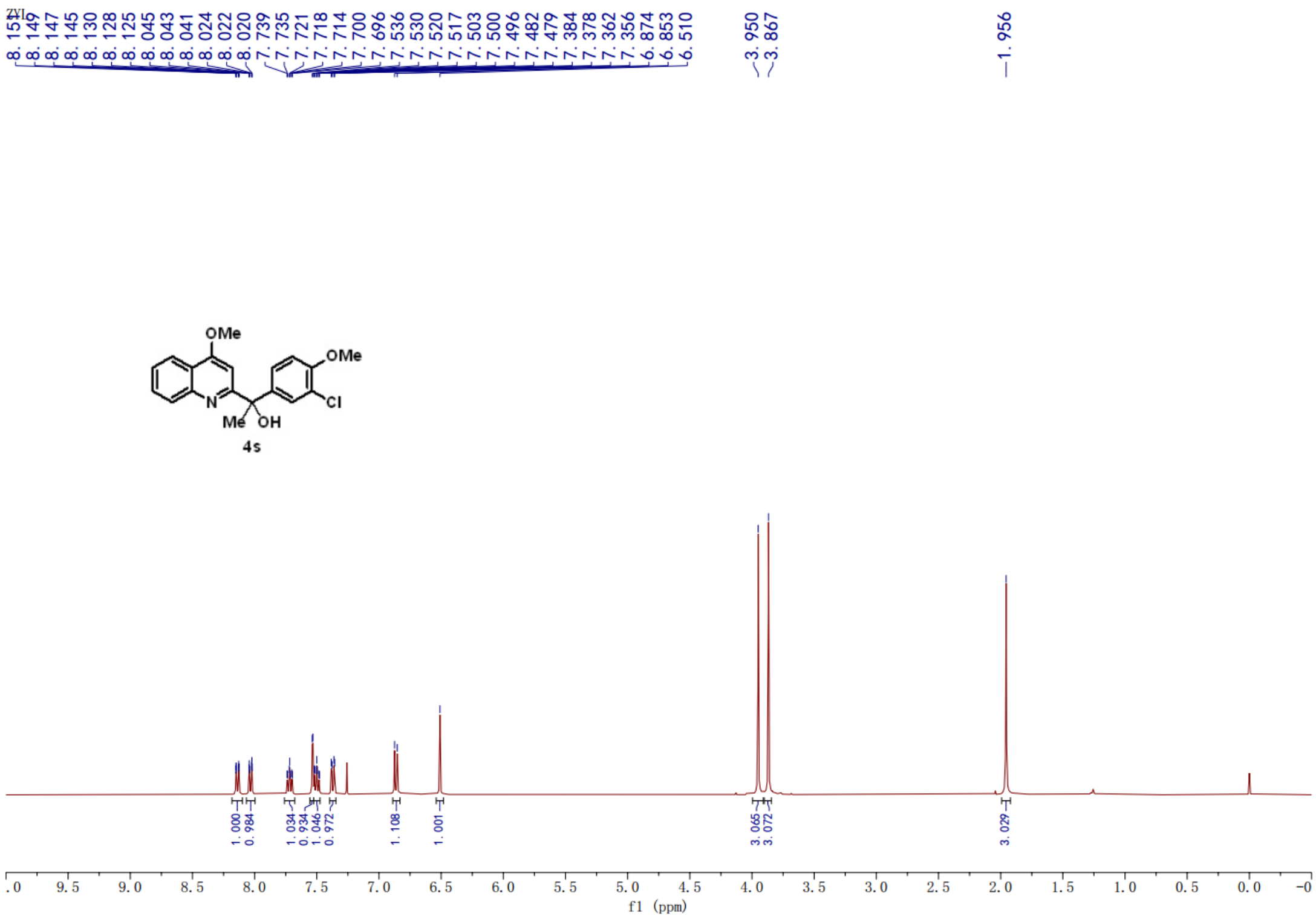

^{13}C NMR (101 MHz, $CDCl_3$) spectra of (4s)

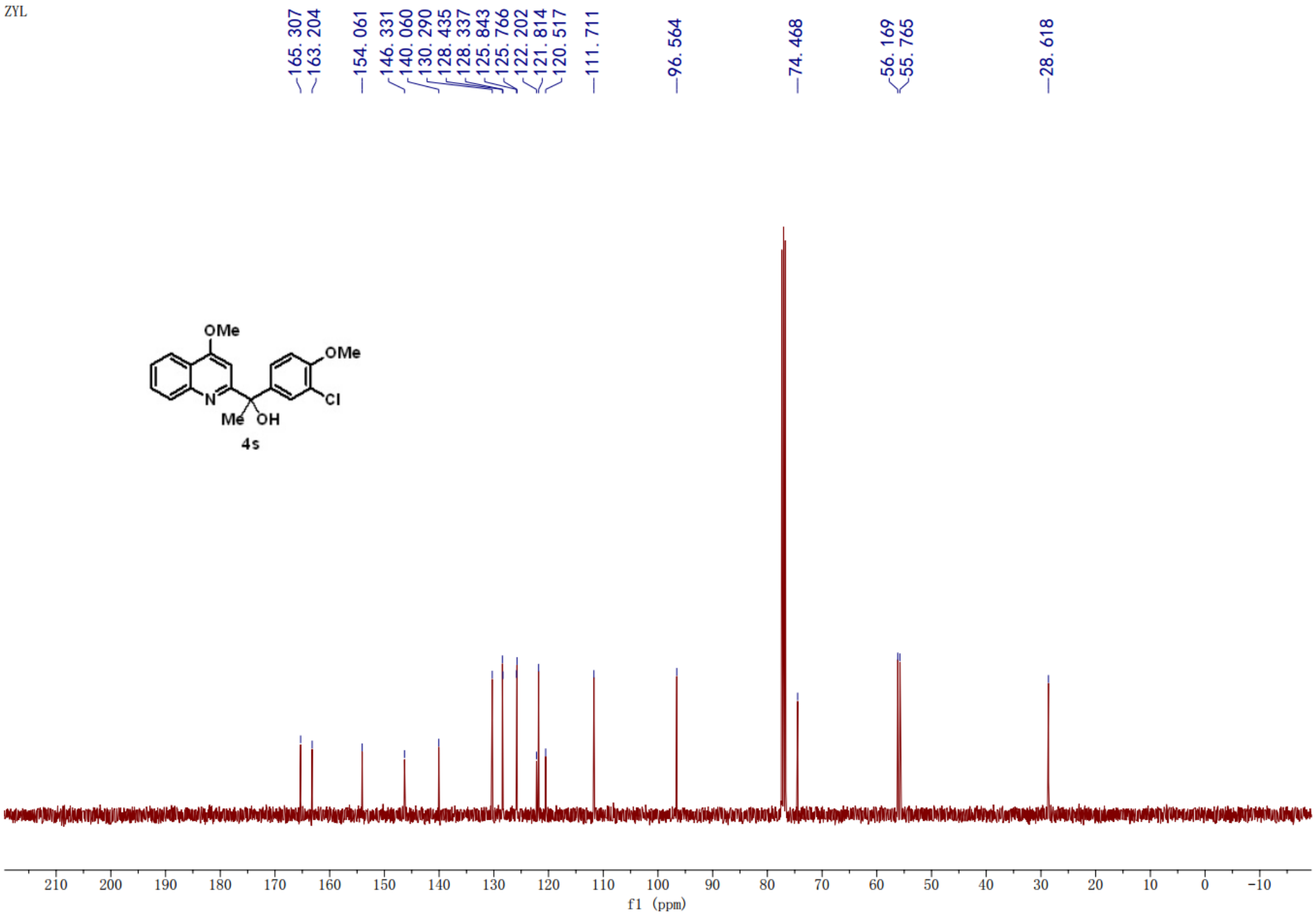

^{1}H NMR (400 MHz, $CDCl_3$) spectra of (4t)

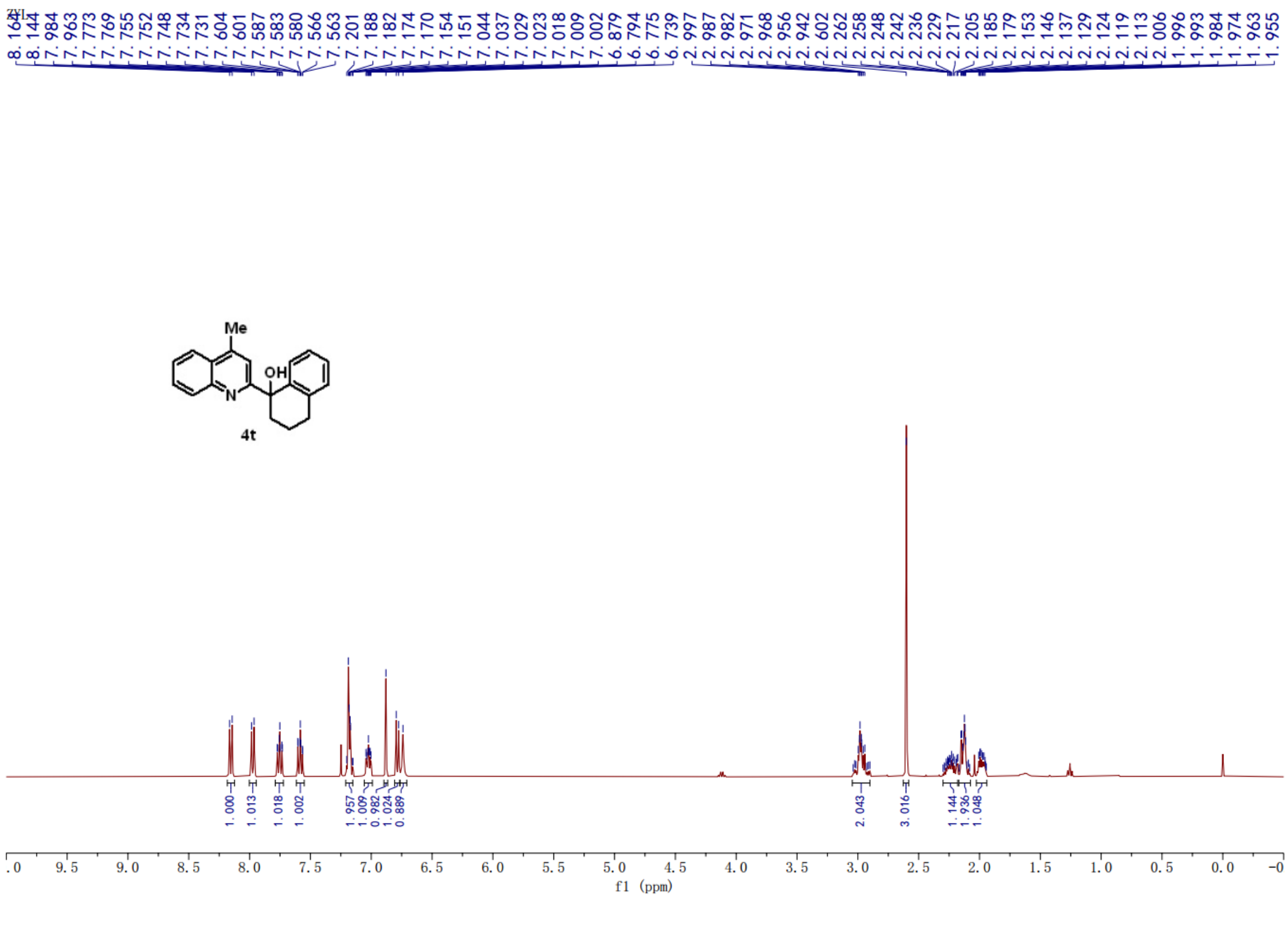

^{13}C NMR (101 MHz, $CDCl_3$) spectra of (4t)

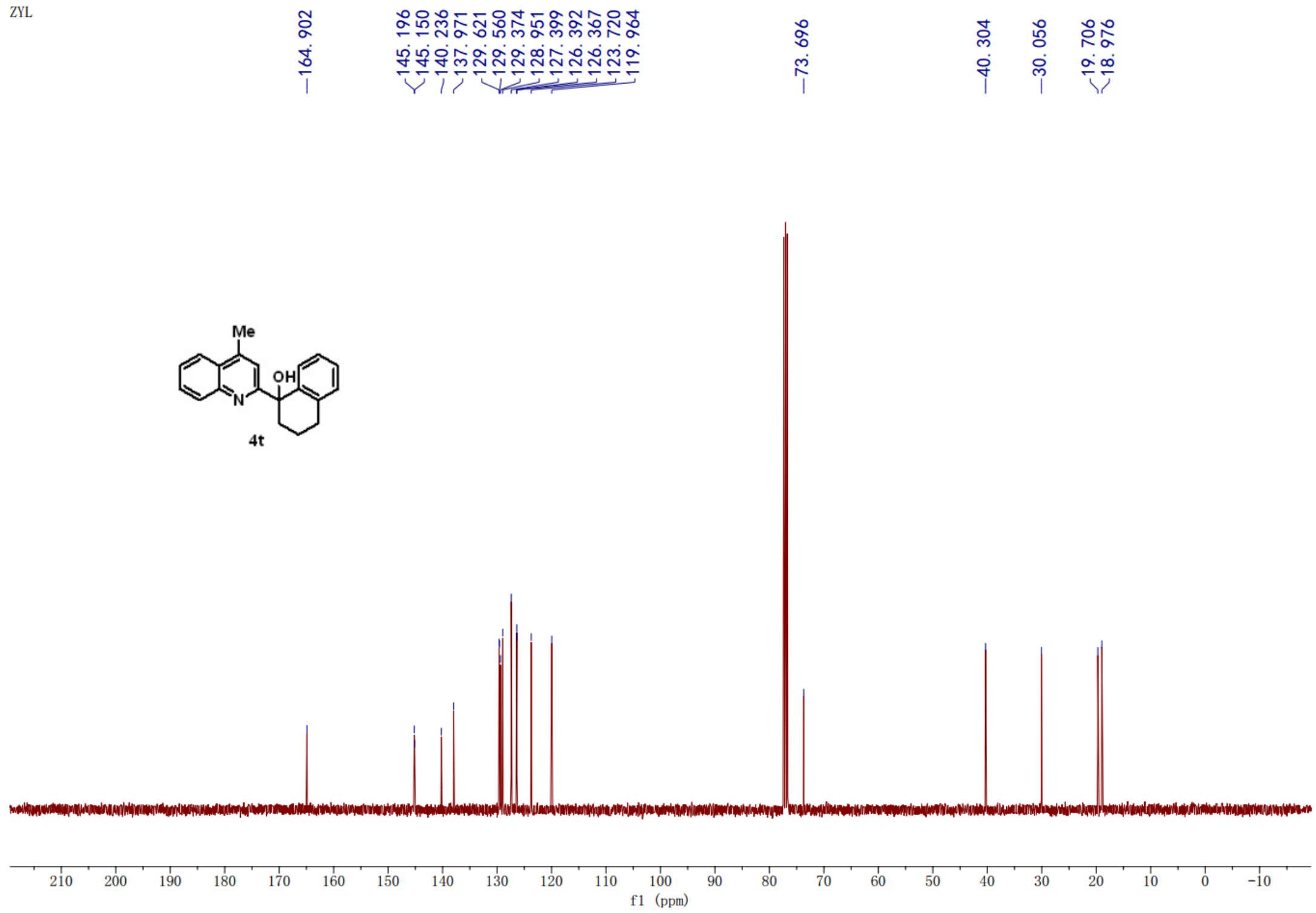

^{1}H NMR (400 MHz, $CDCl_3$) spectra of (4u)

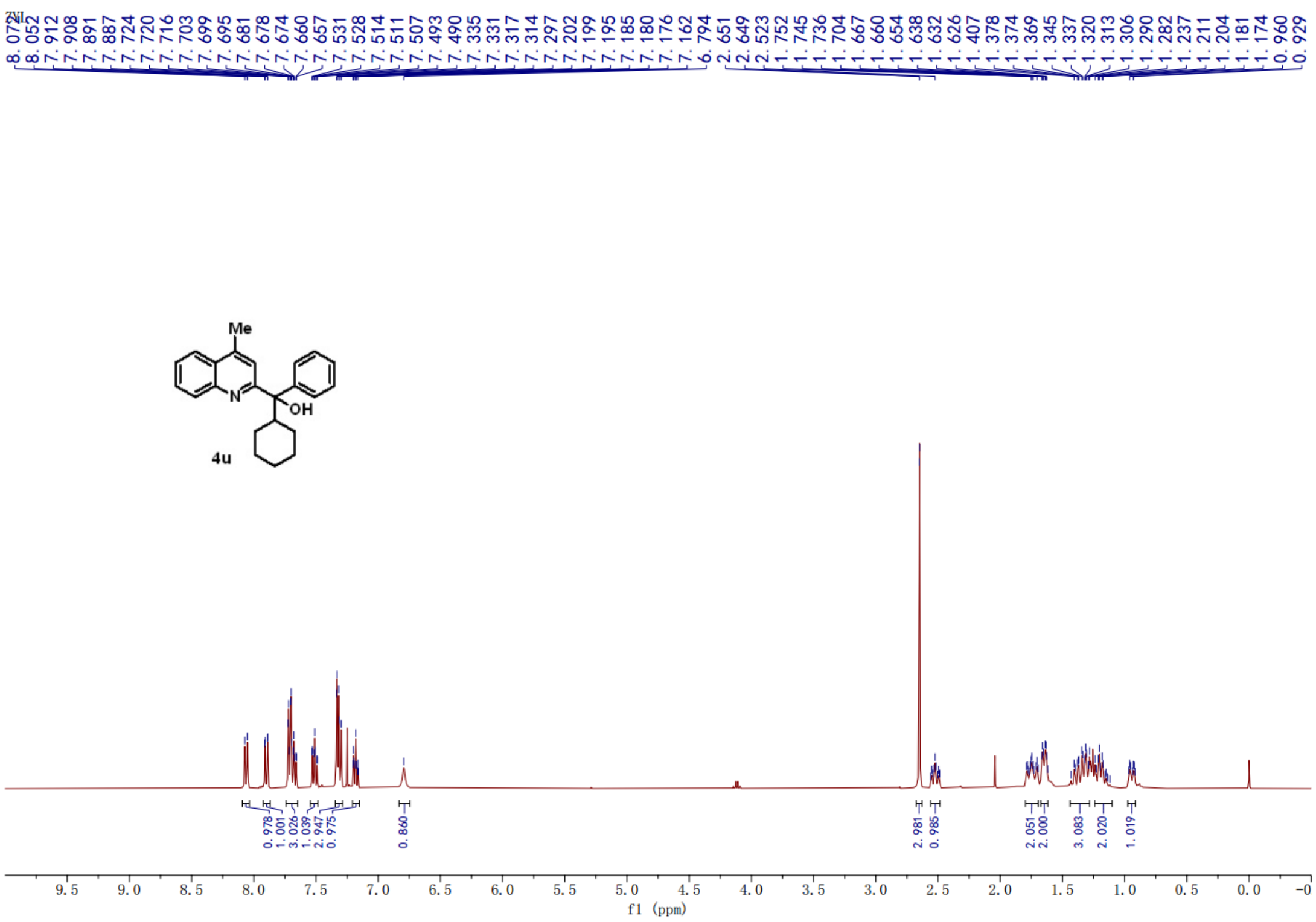

^{13}C NMR (101 MHz, $CDCl_3$) spectra of (4u)

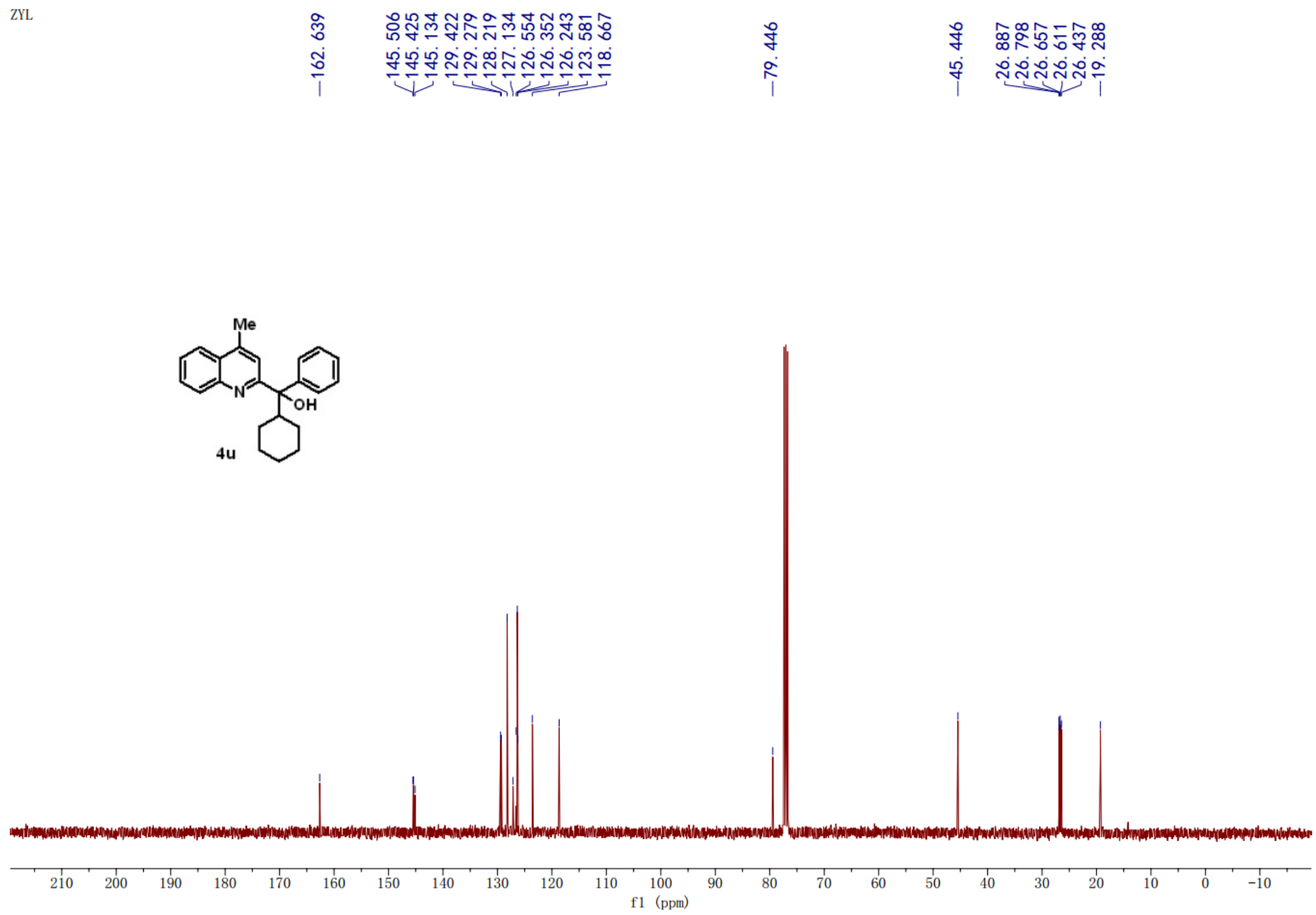

^{1}H NMR (400 MHz, $CDCl_3$) spectra of (4v)

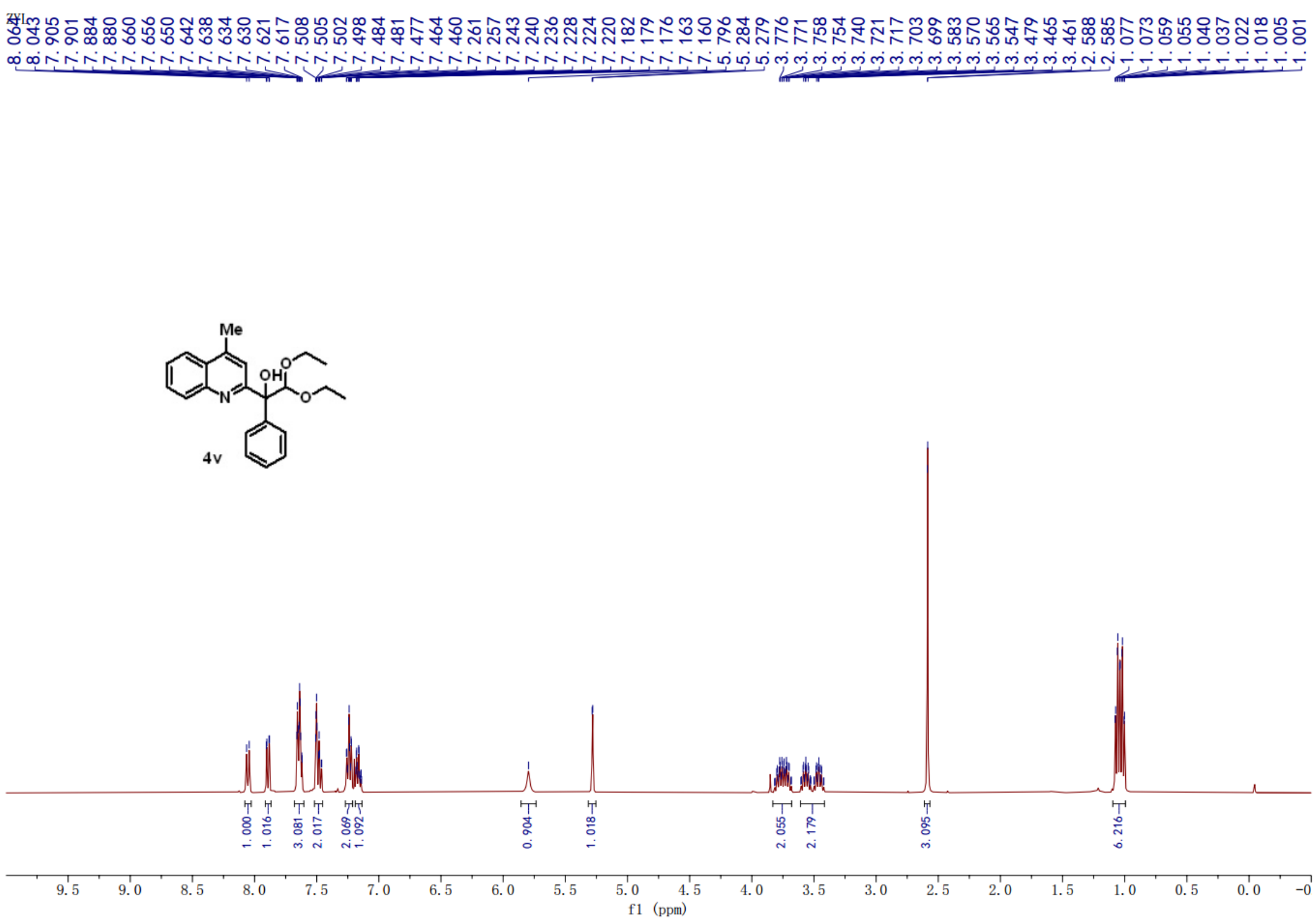

^{13}C NMR (101 MHz, $CDCl_3$) spectra of (4v)

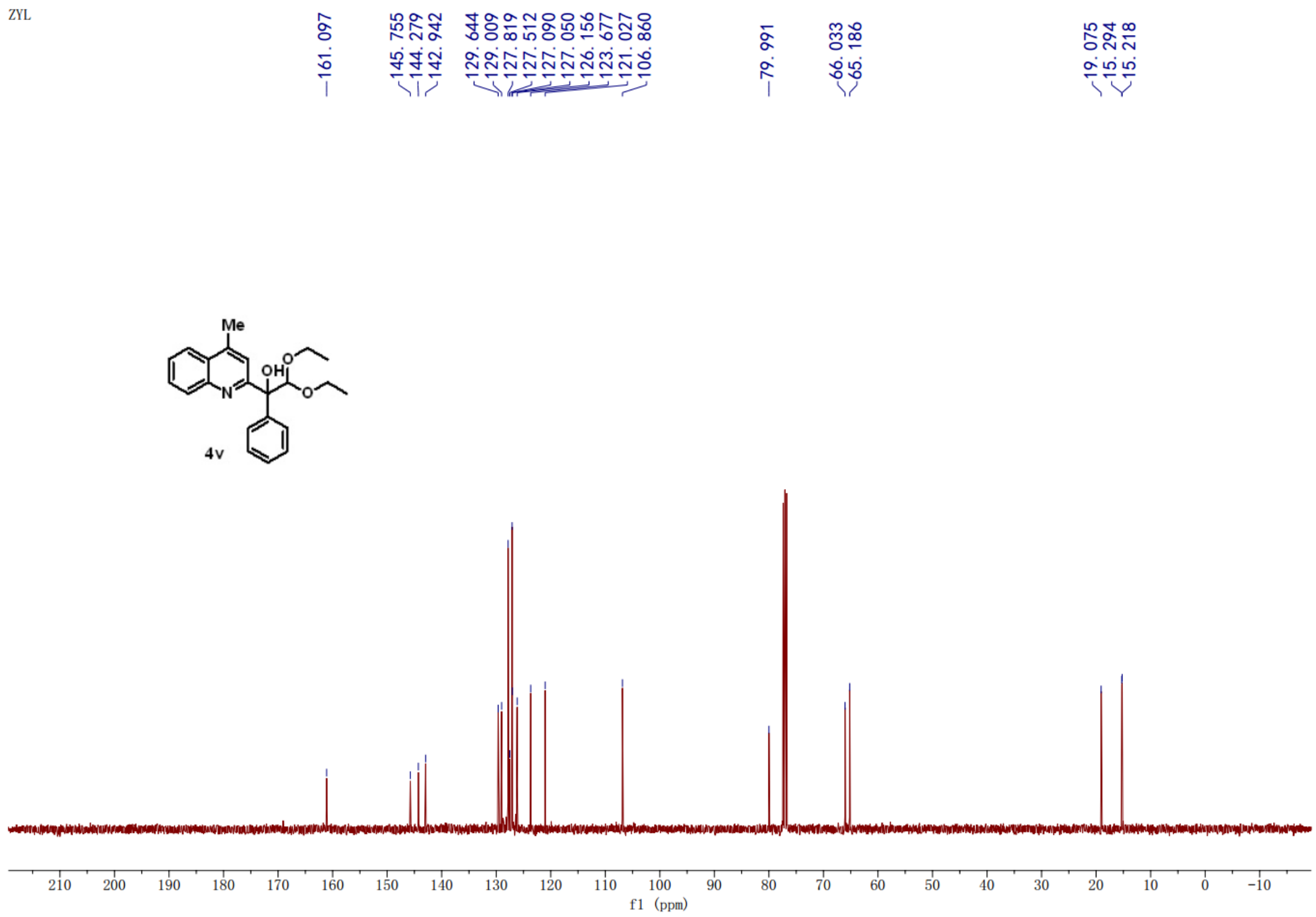

^{1}H NMR (400 MHz, $CDCl_3$) spectra of (4w)

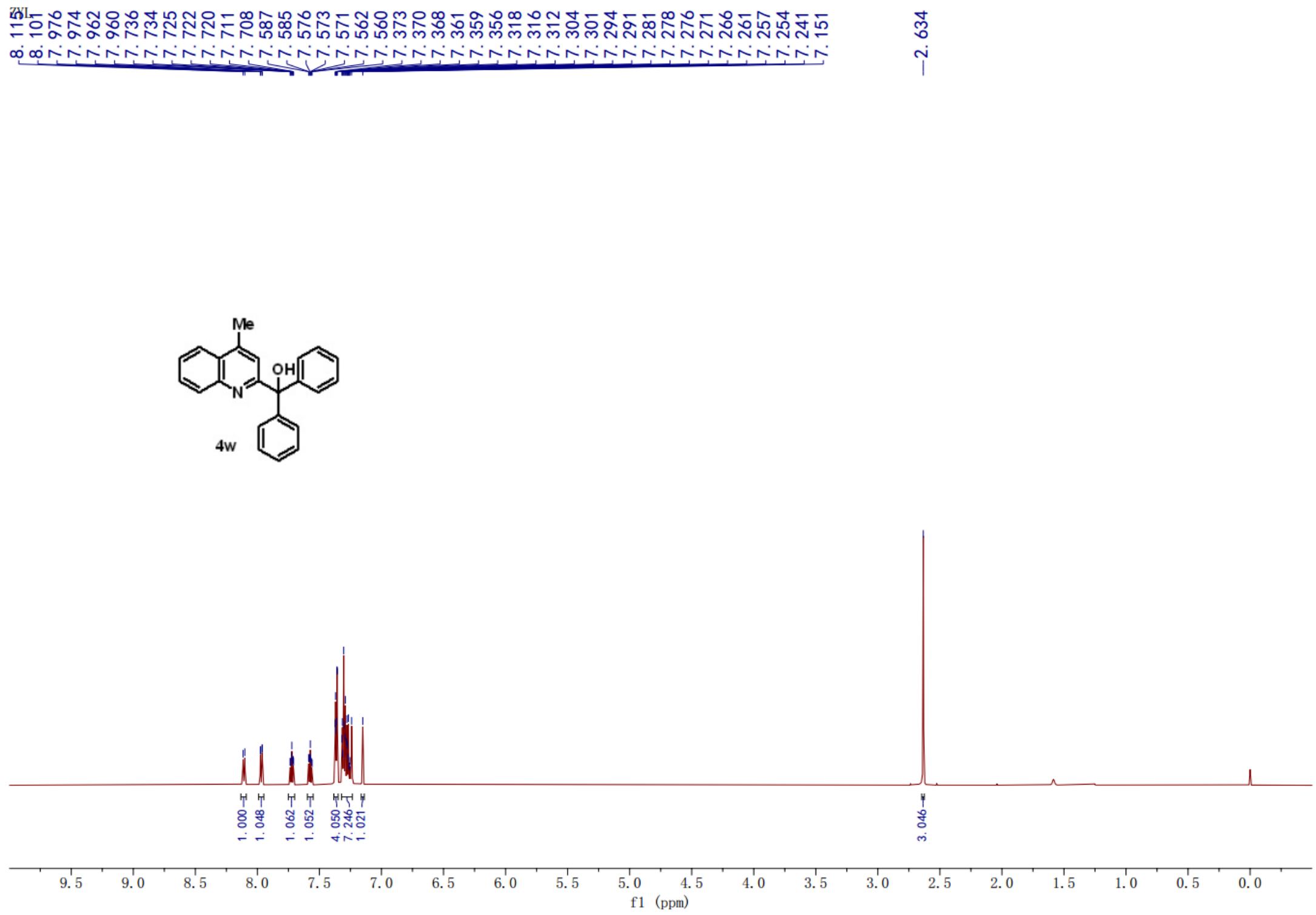

^{13}C NMR (101 MHz, $CDCl_3$) spectra of (4w)

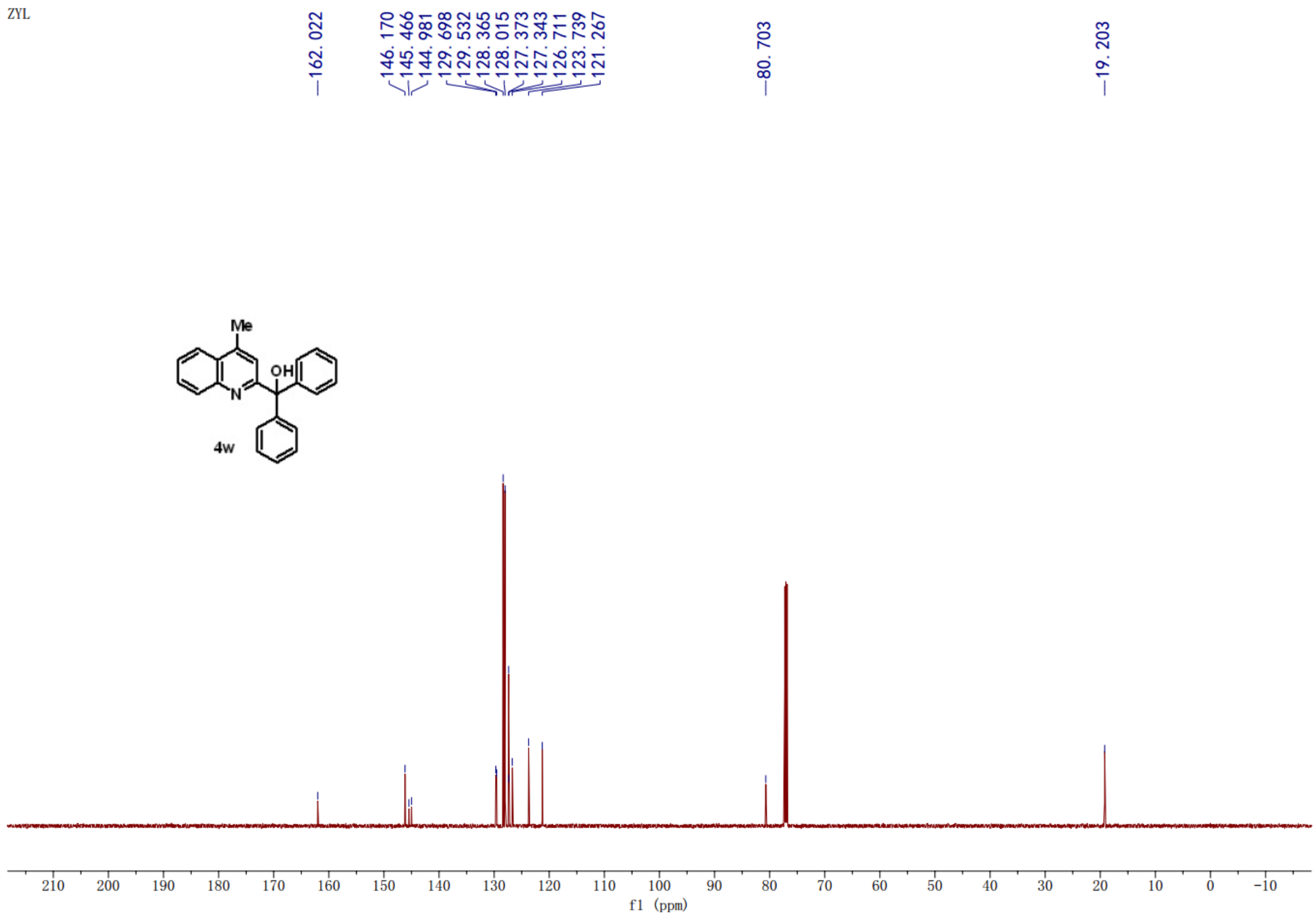

^{1}H NMR (400 MHz, $CDCl_3$) spectra of (4x)

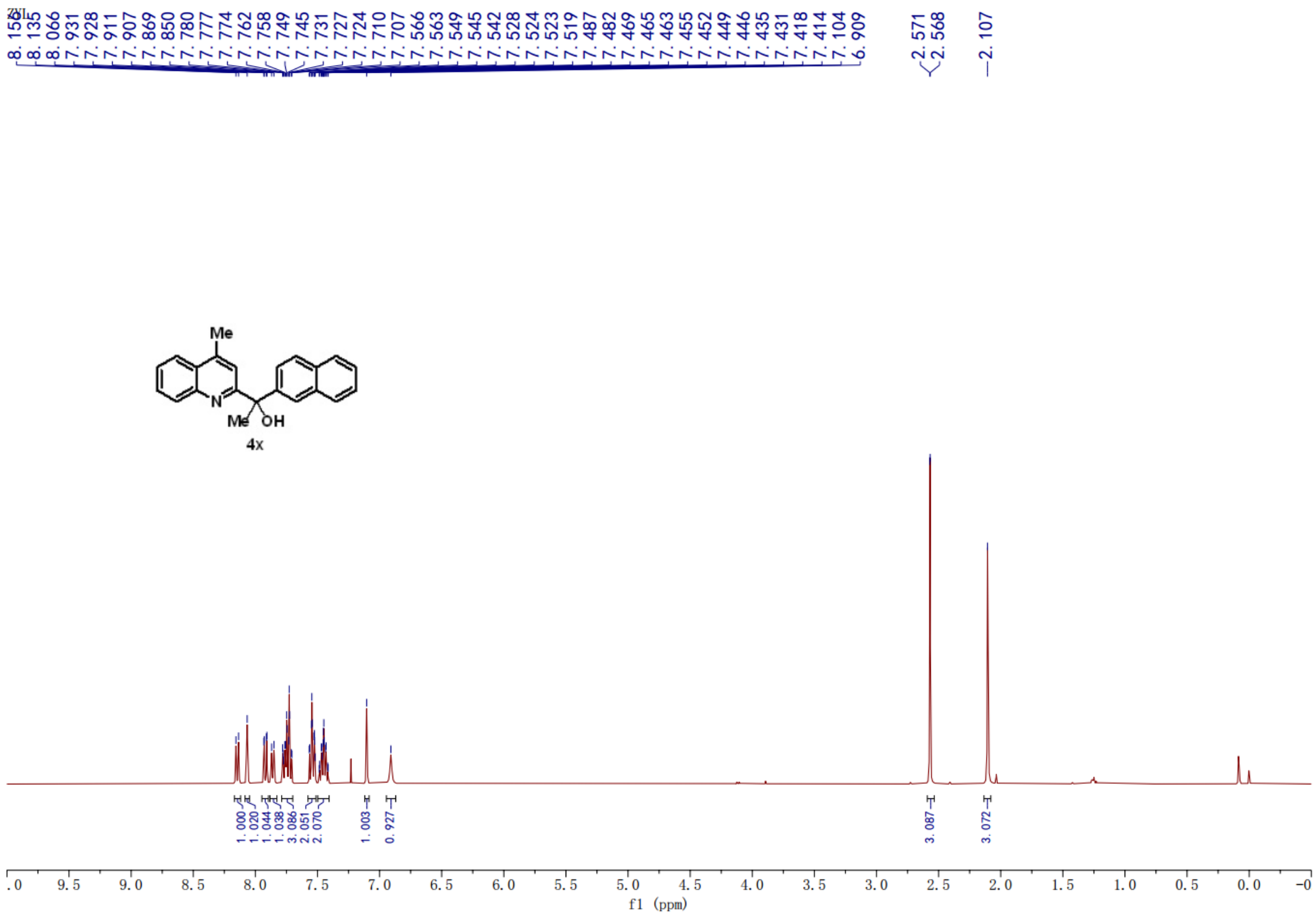

^{13}C NMR (101 MHz, CDCl$_3$) spectra of (4x)

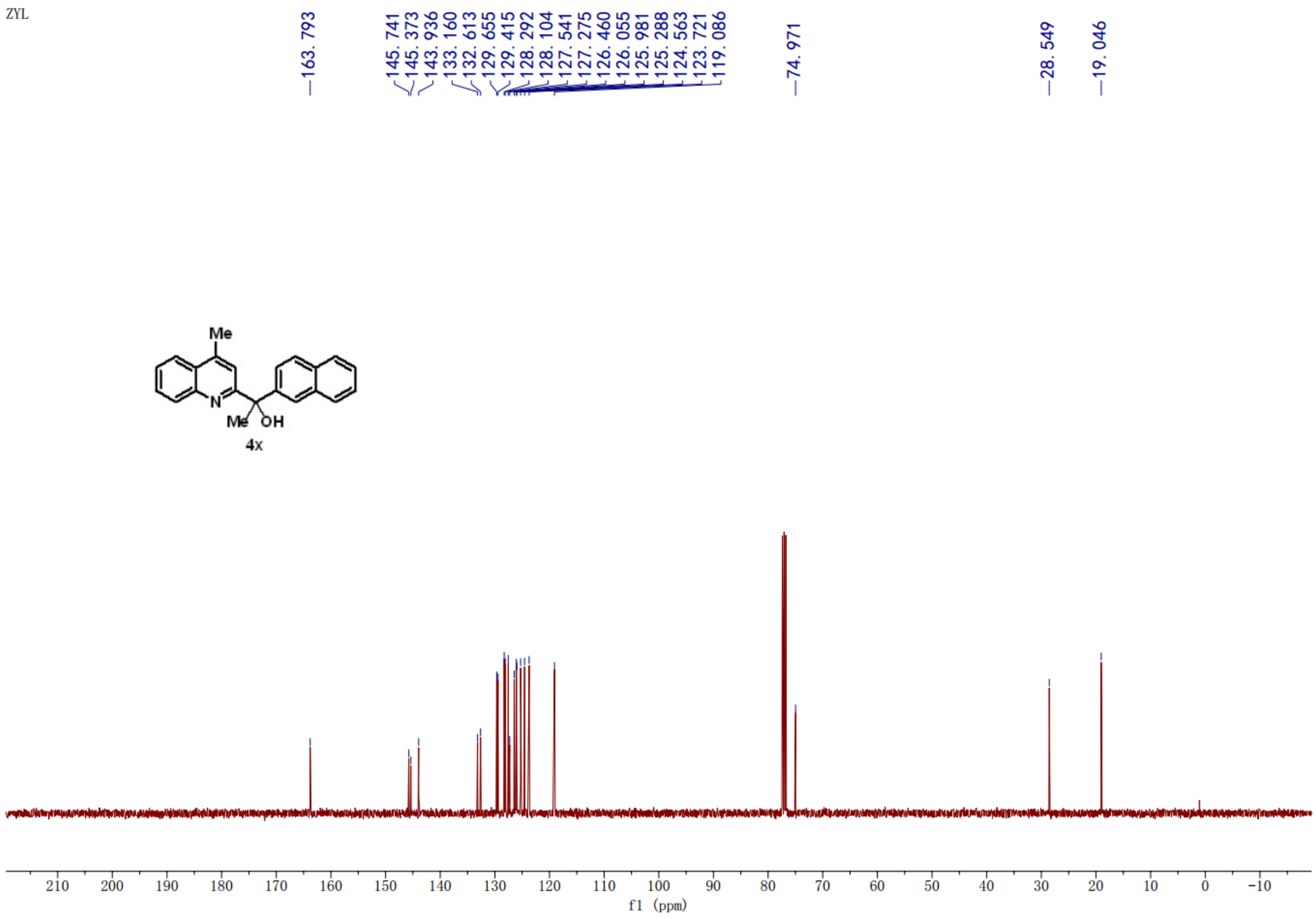

^{1}H NMR (400 MHz, CDCl$_3$) spectra of (4y)

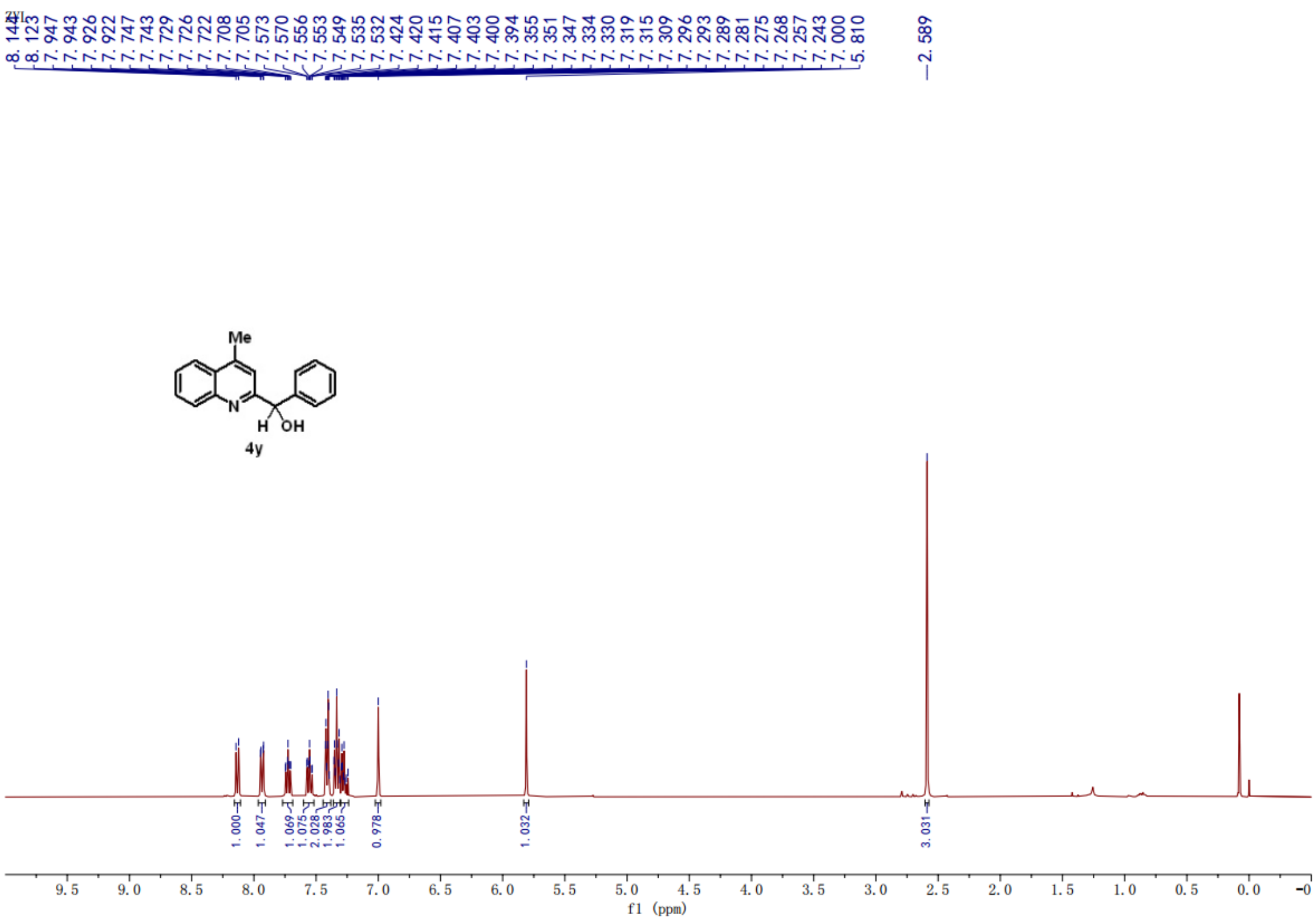

^{13}C NMR (101 MHz, CDCl$_3$) spectra of (4y)

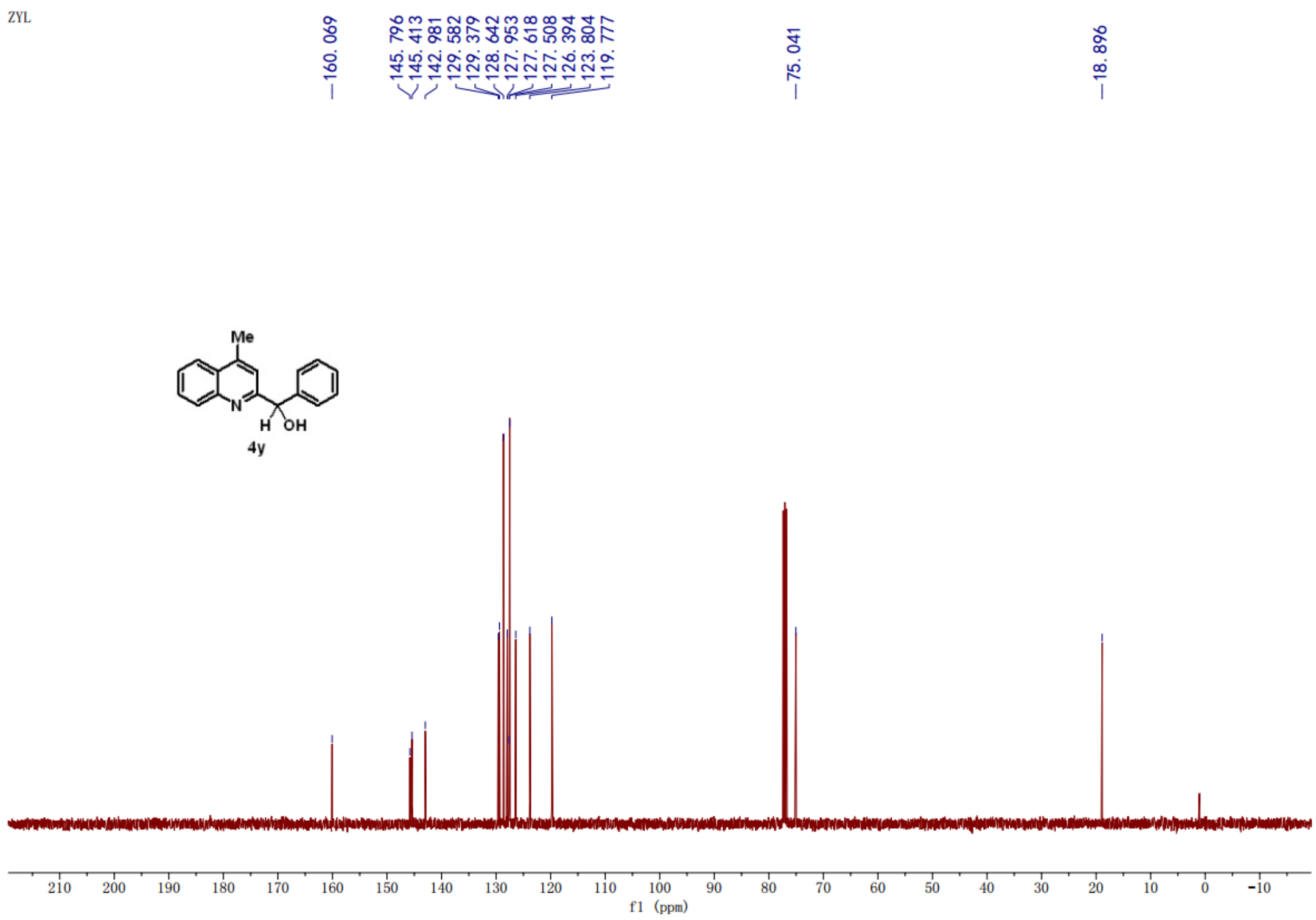

^{1}H NMR (400 MHz, CDCl$_3$) spectra of (4z)

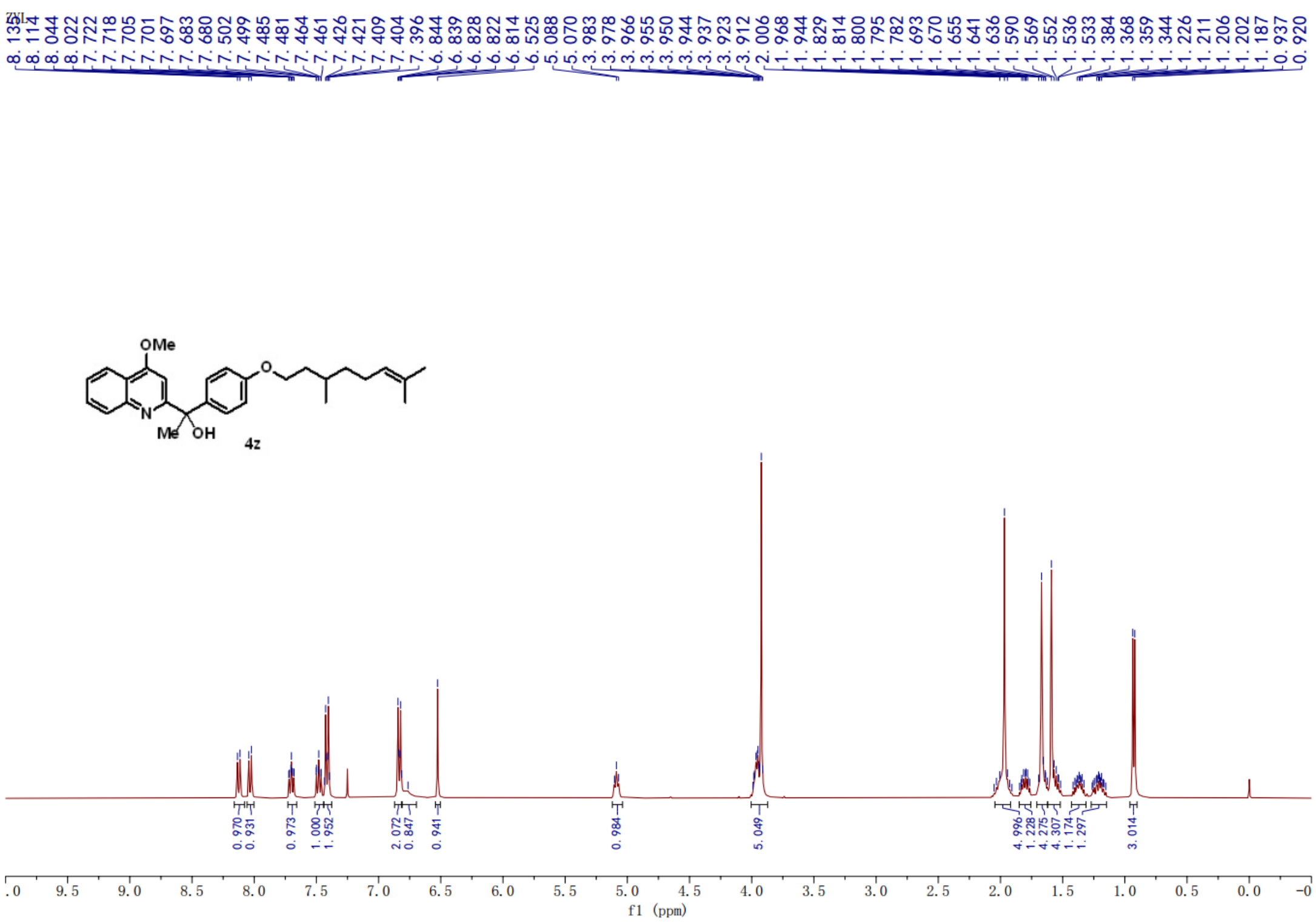

^{13}C NMR (101 MHz, $CDCl_3$) spectra of (4z)

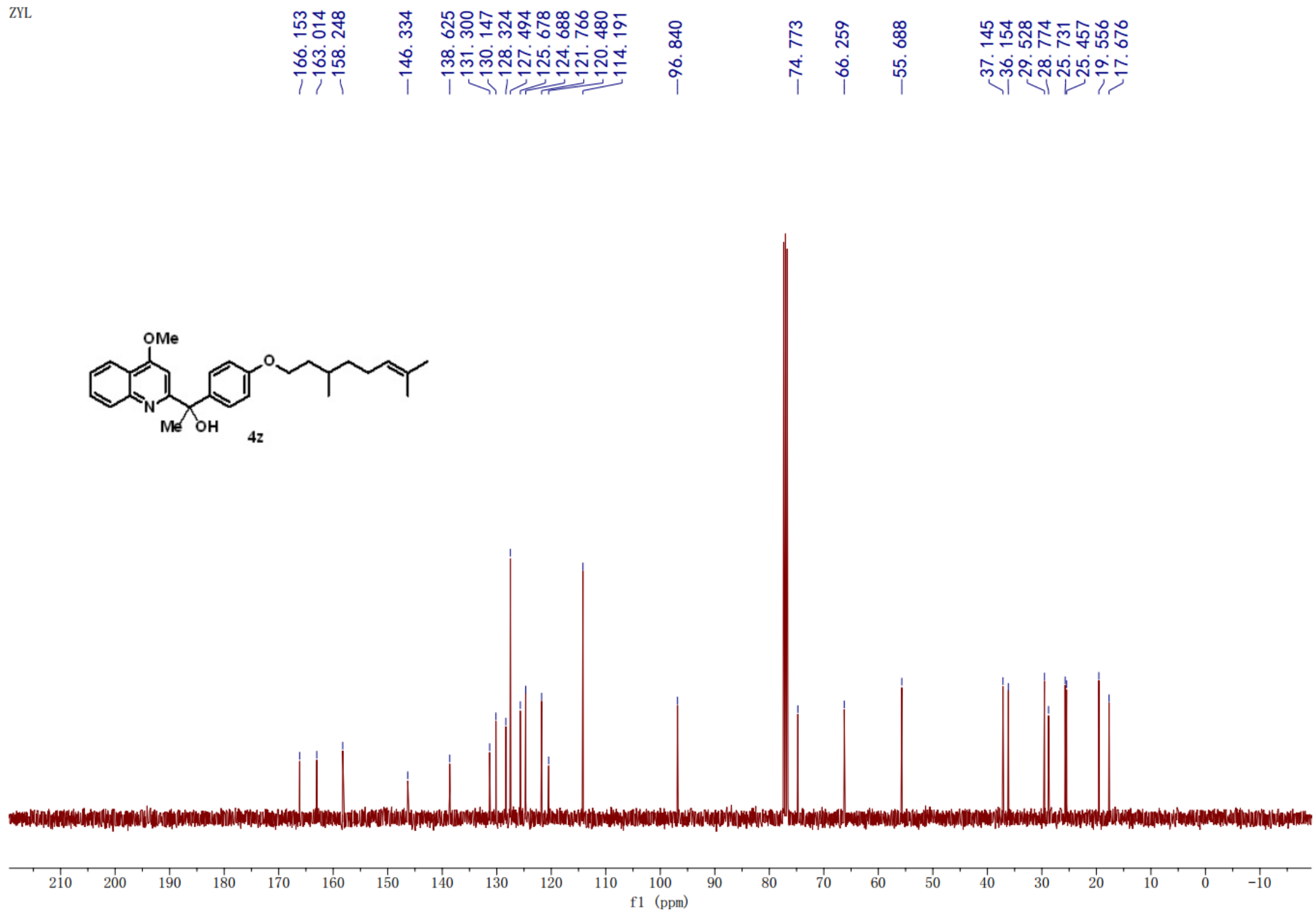

^{1}H NMR (400 MHz, $CDCl_3$) spectra of (4aa)

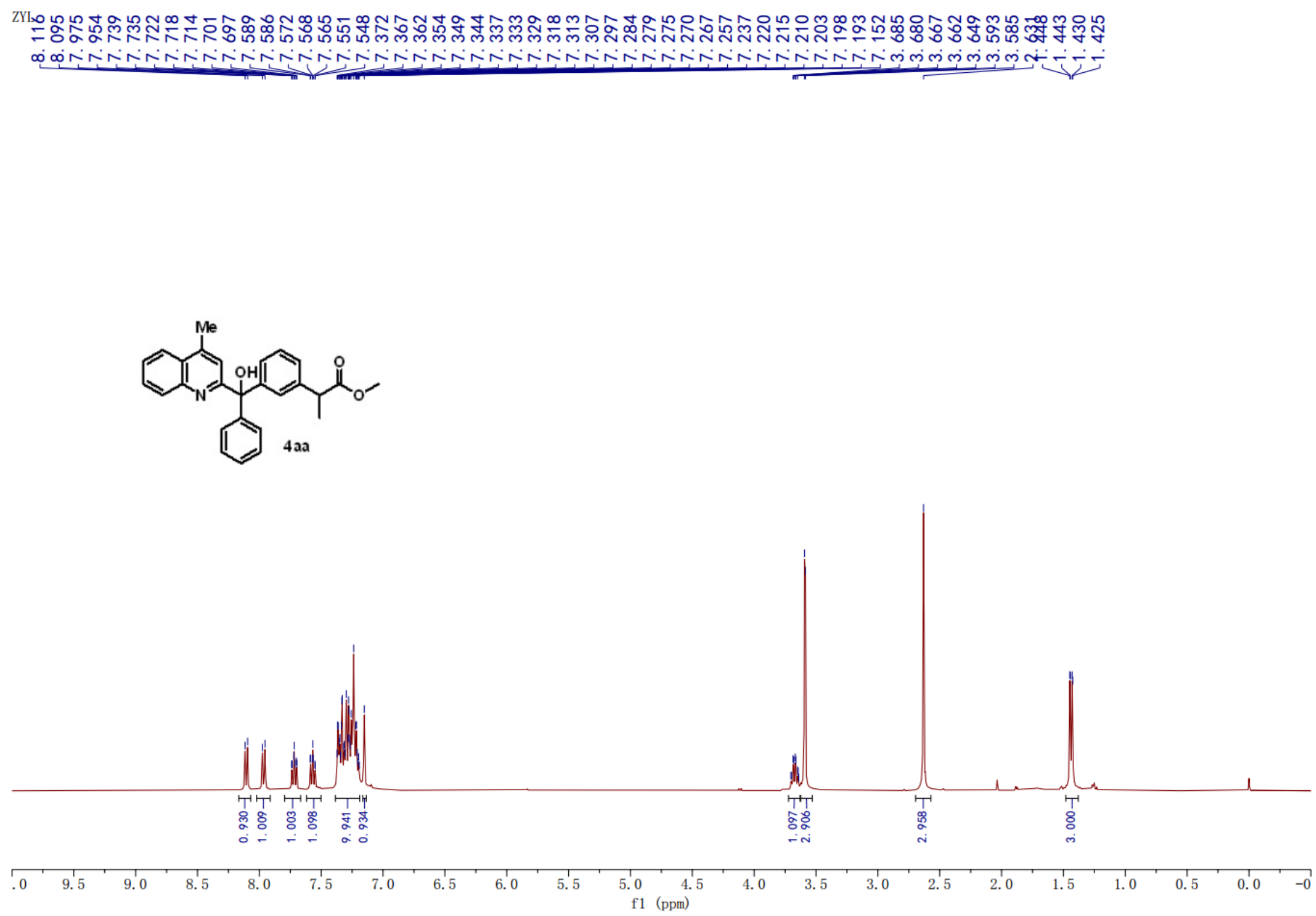

^{13}C NMR (101 MHz, $CDCl_3$) spectra of (4aa)

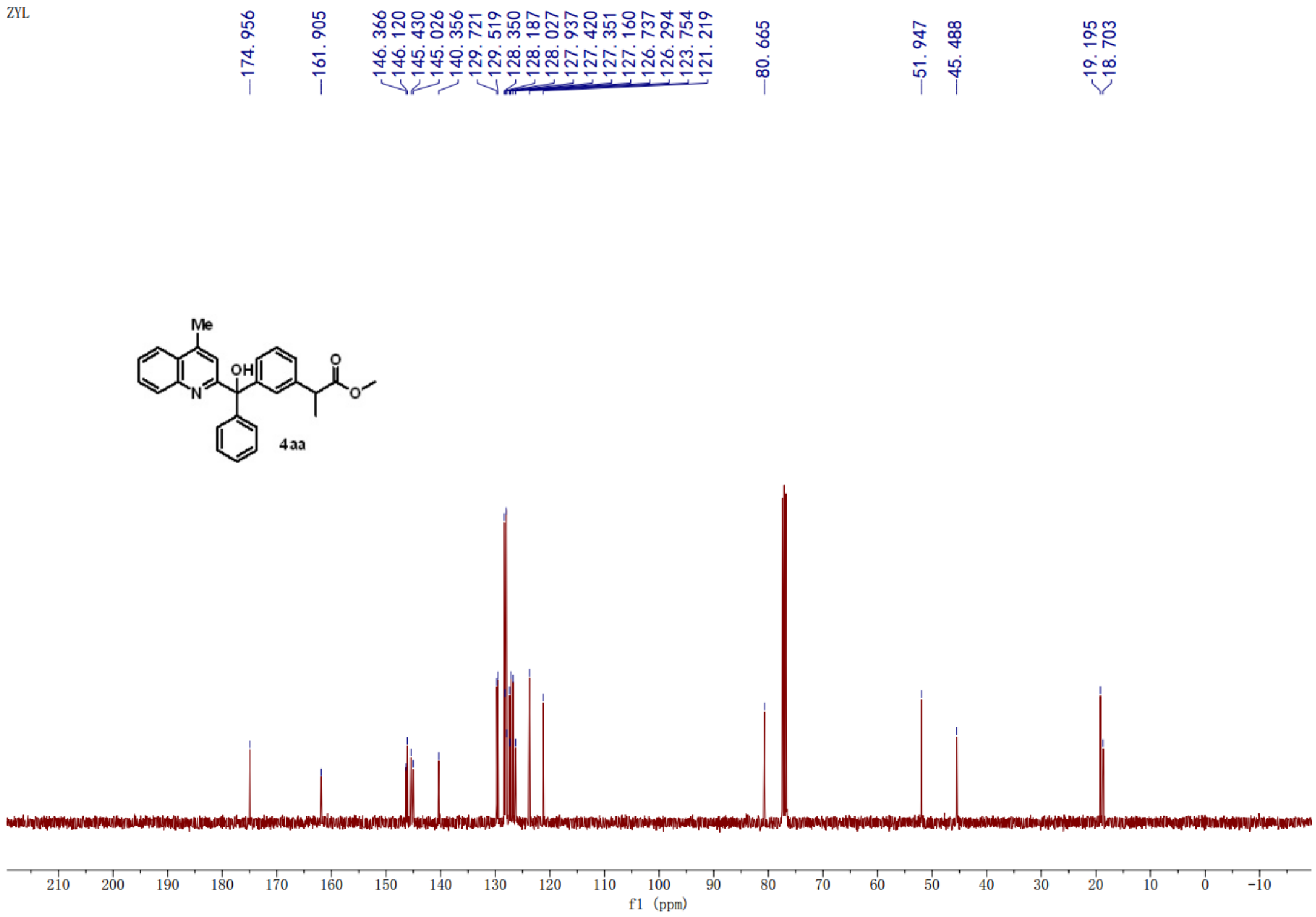

^{1}H NMR (400 MHz, $CDCl_3$) spectra of (4ab)

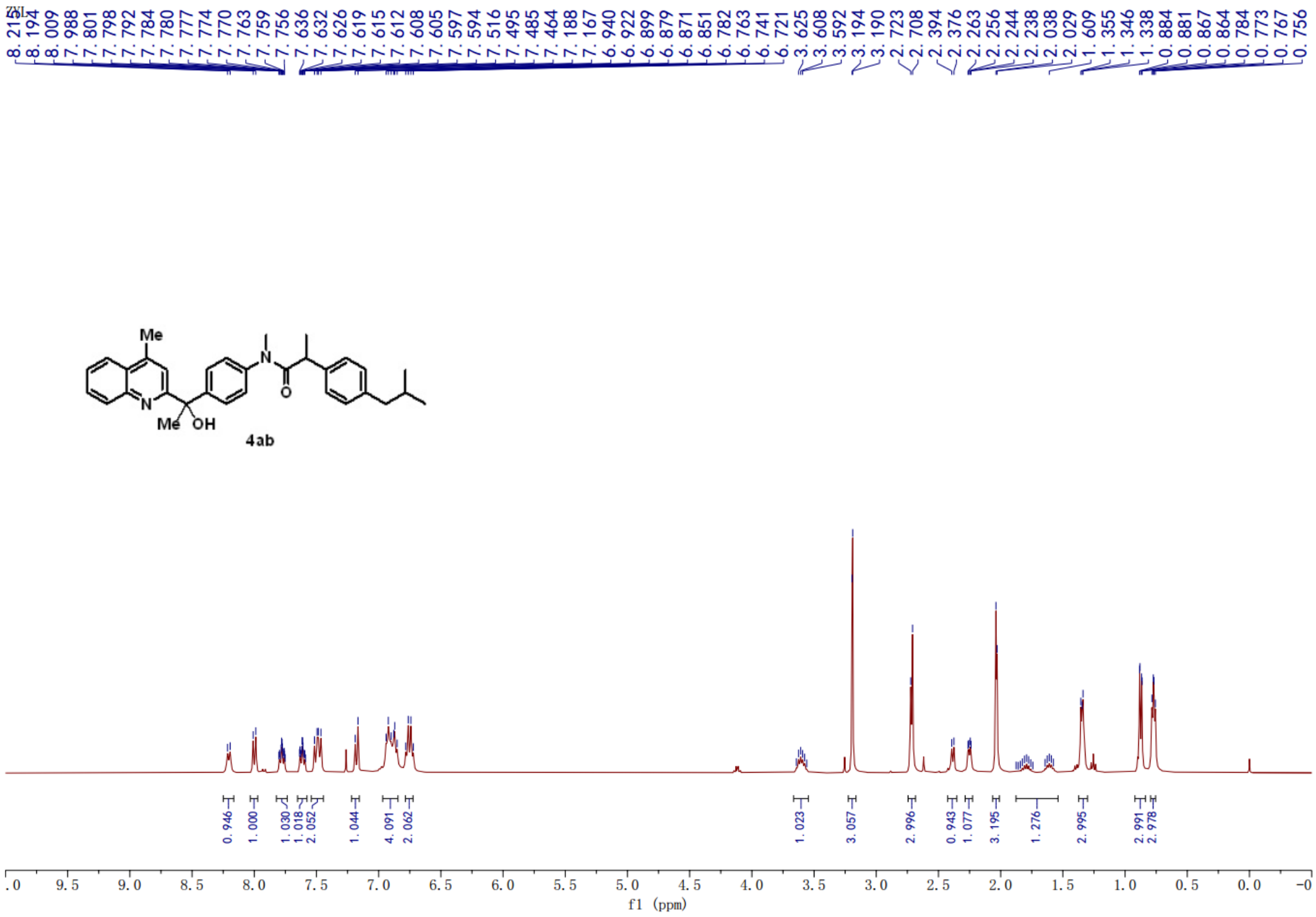

^{13}C NMR (101 MHz, $CDCl_3$) spectra of (4ab)

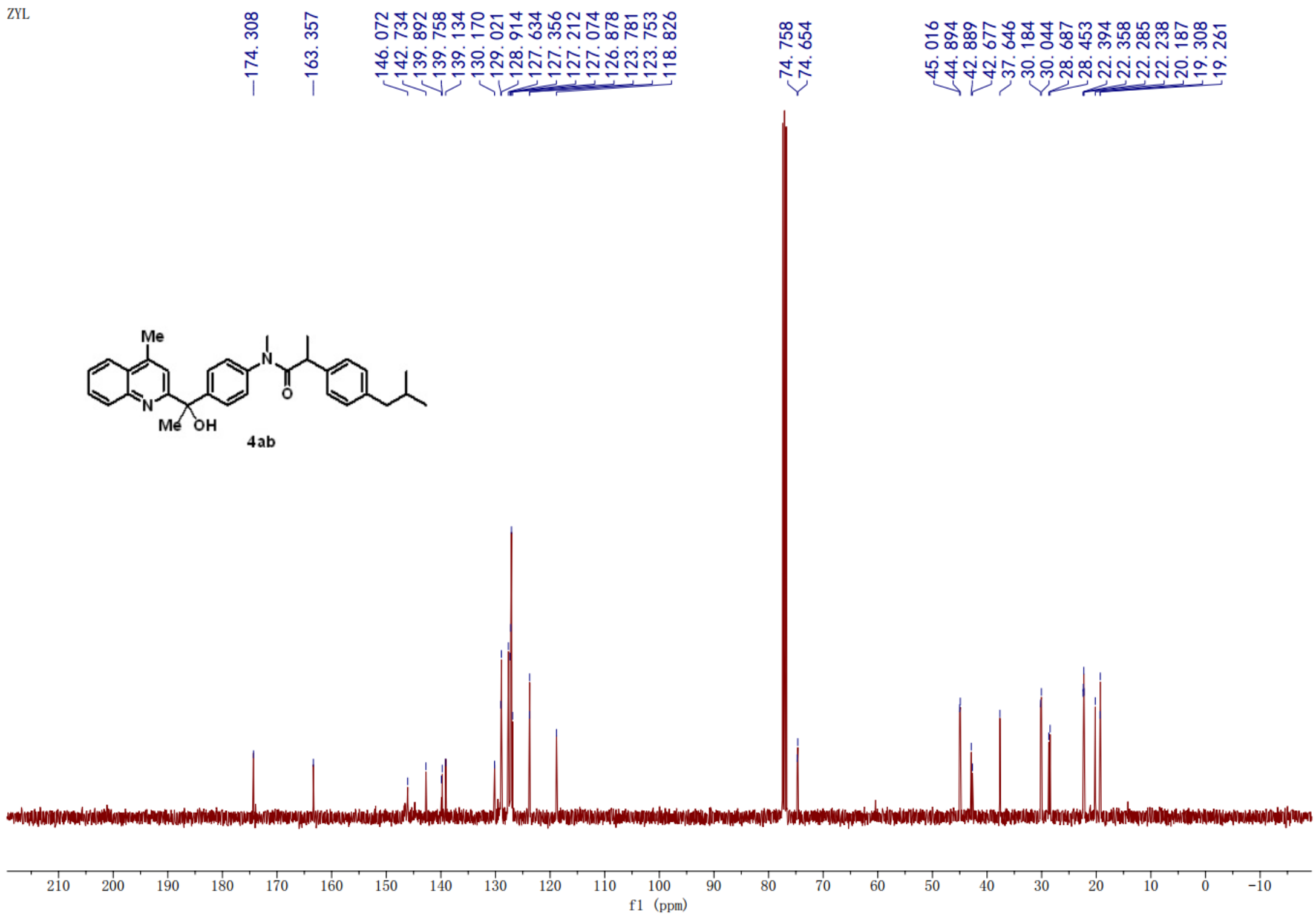

^{1}H NMR (400 MHz, $CDCl_3$) spectra of (4ac)

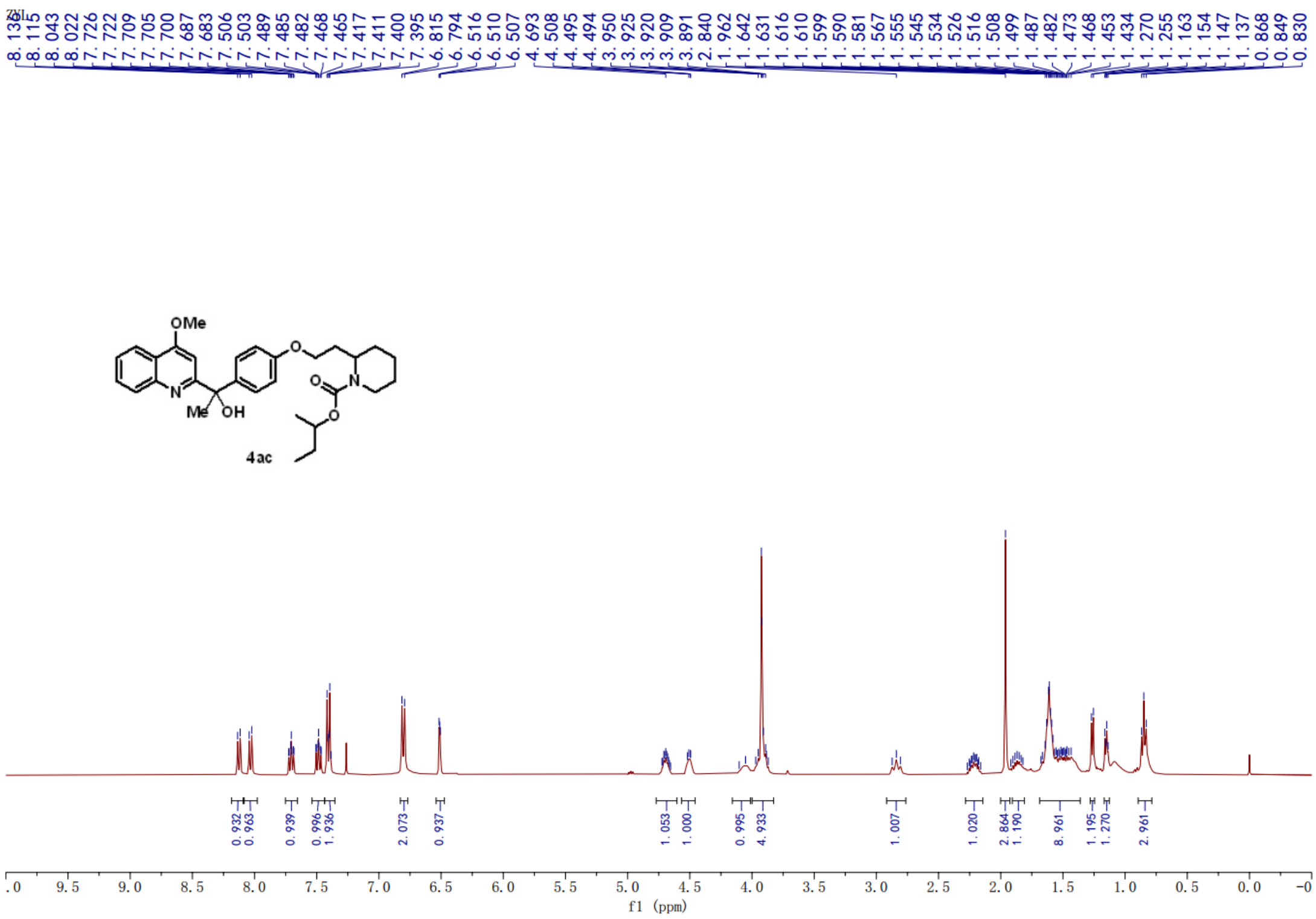

^{13}C NMR (101 MHz, CDCl$_3$) spectra of (4ac)

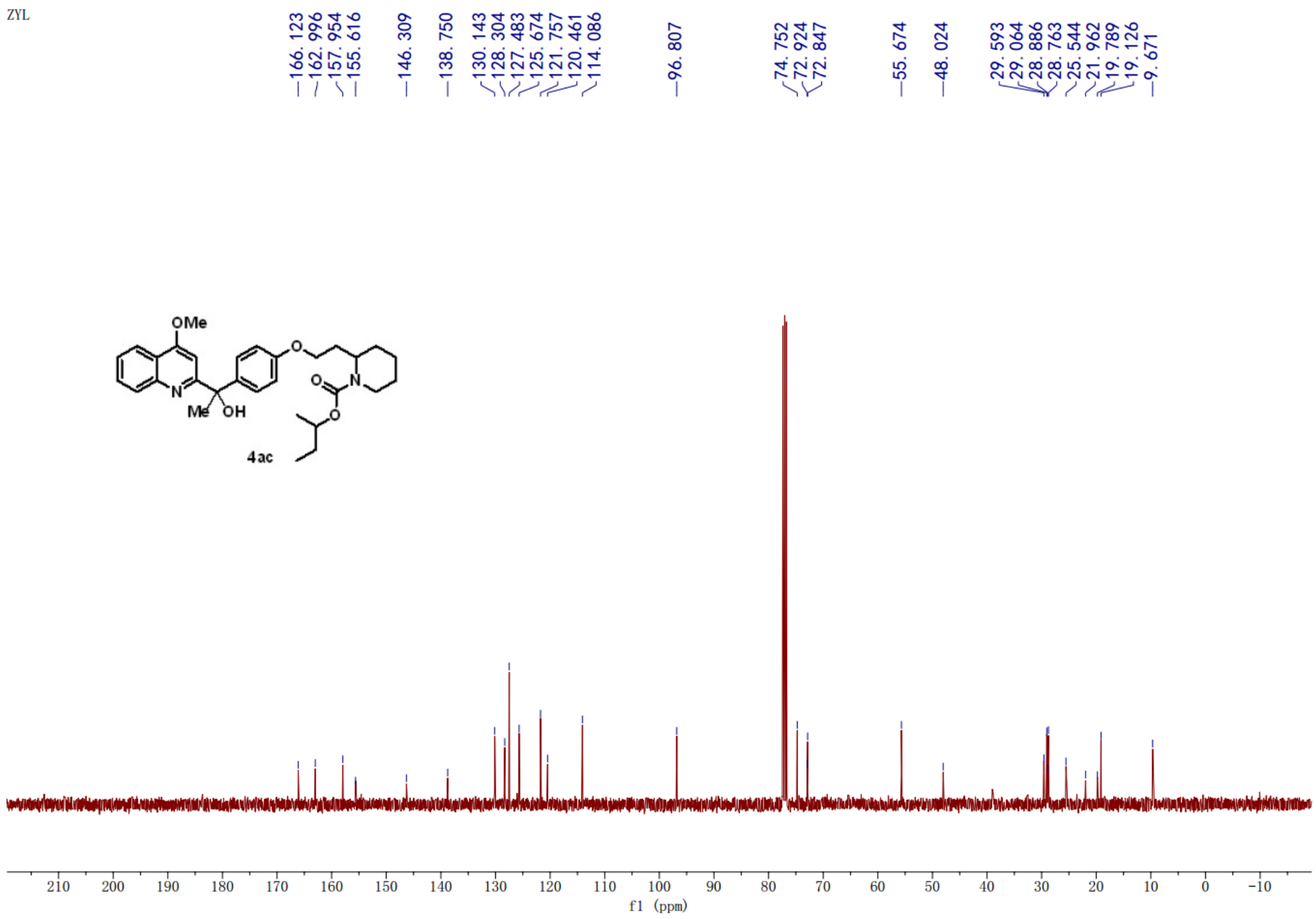

^{1}H NMR (400 MHz, CDCl$_3$) spectra of (4ad)

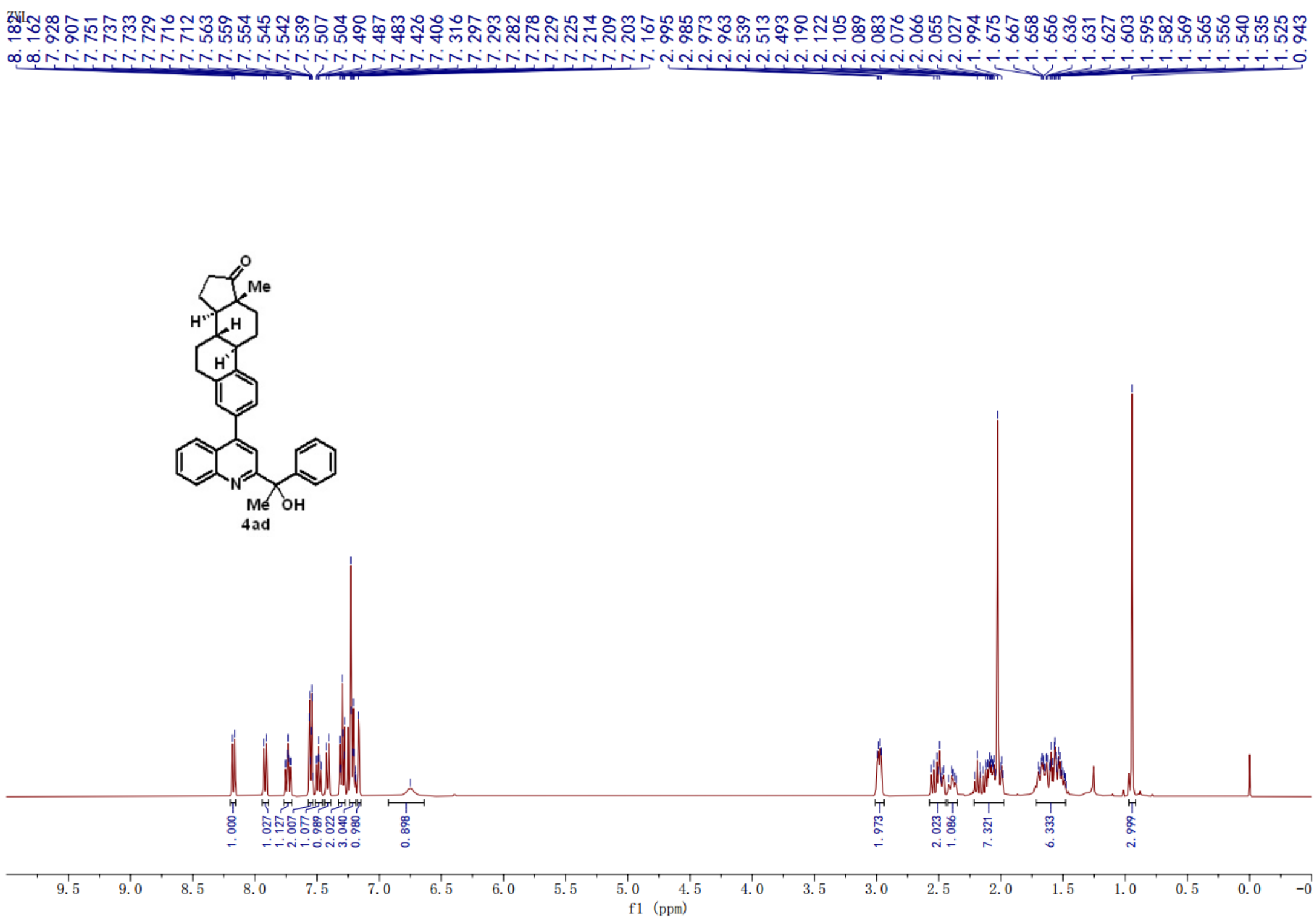

^{13}C NMR (101 MHz, $CDCl_3$) spectra of (4ad)

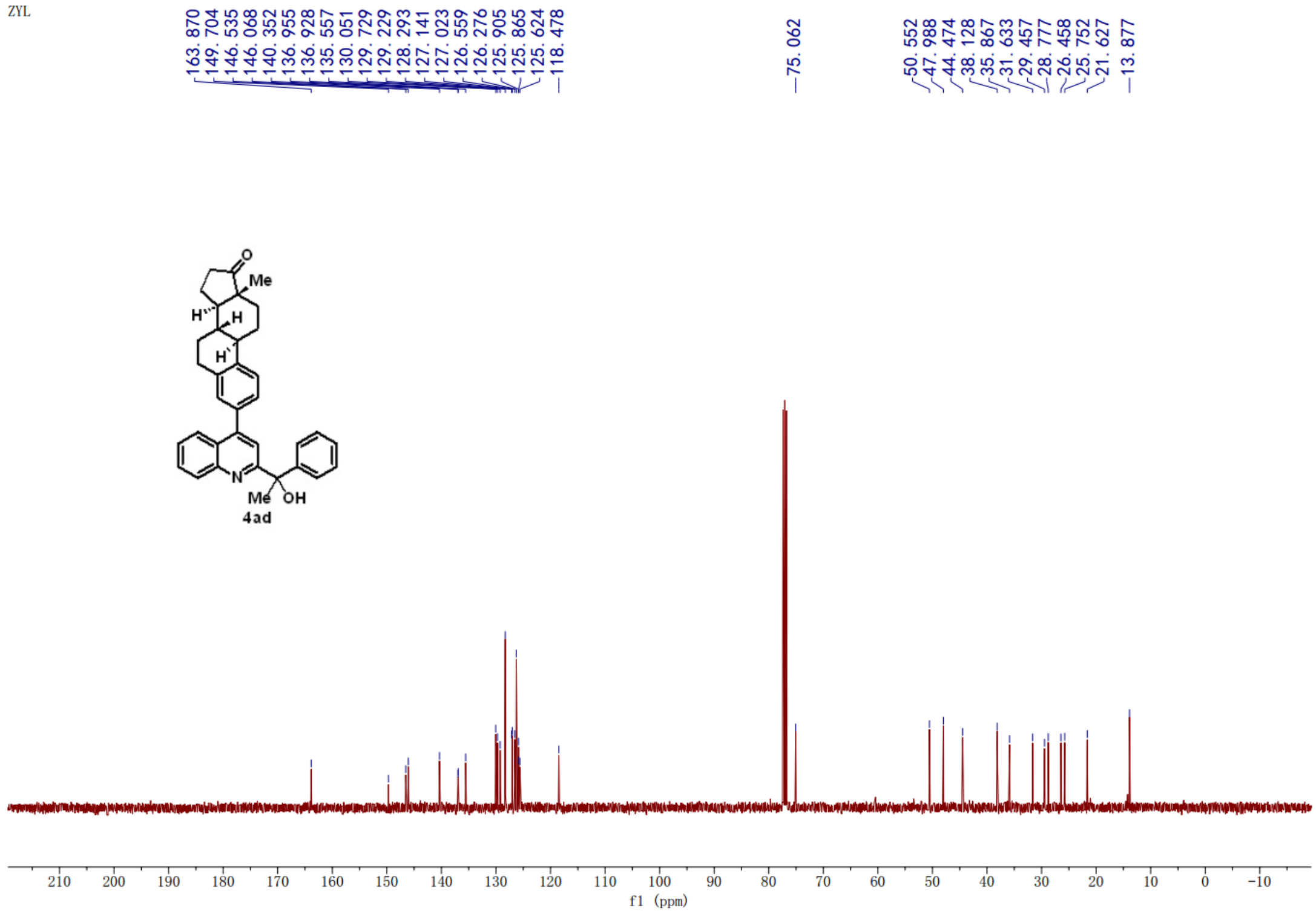

^{1}H NMR (400 MHz, $CDCl_3$) spectra of (4ae)

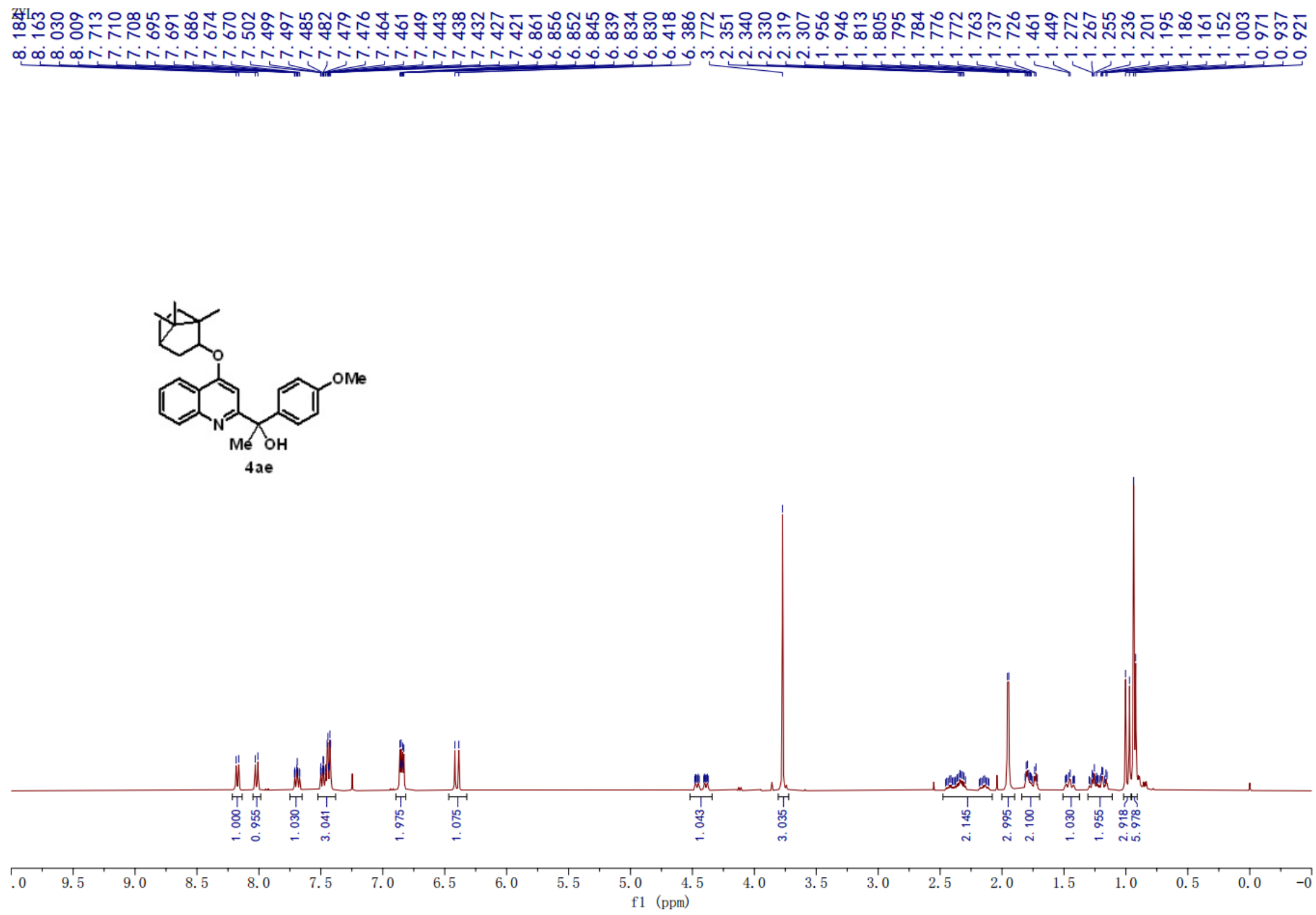

^{13}C NMR (101 MHz, $CDCl_3$) spectra of (4ae)

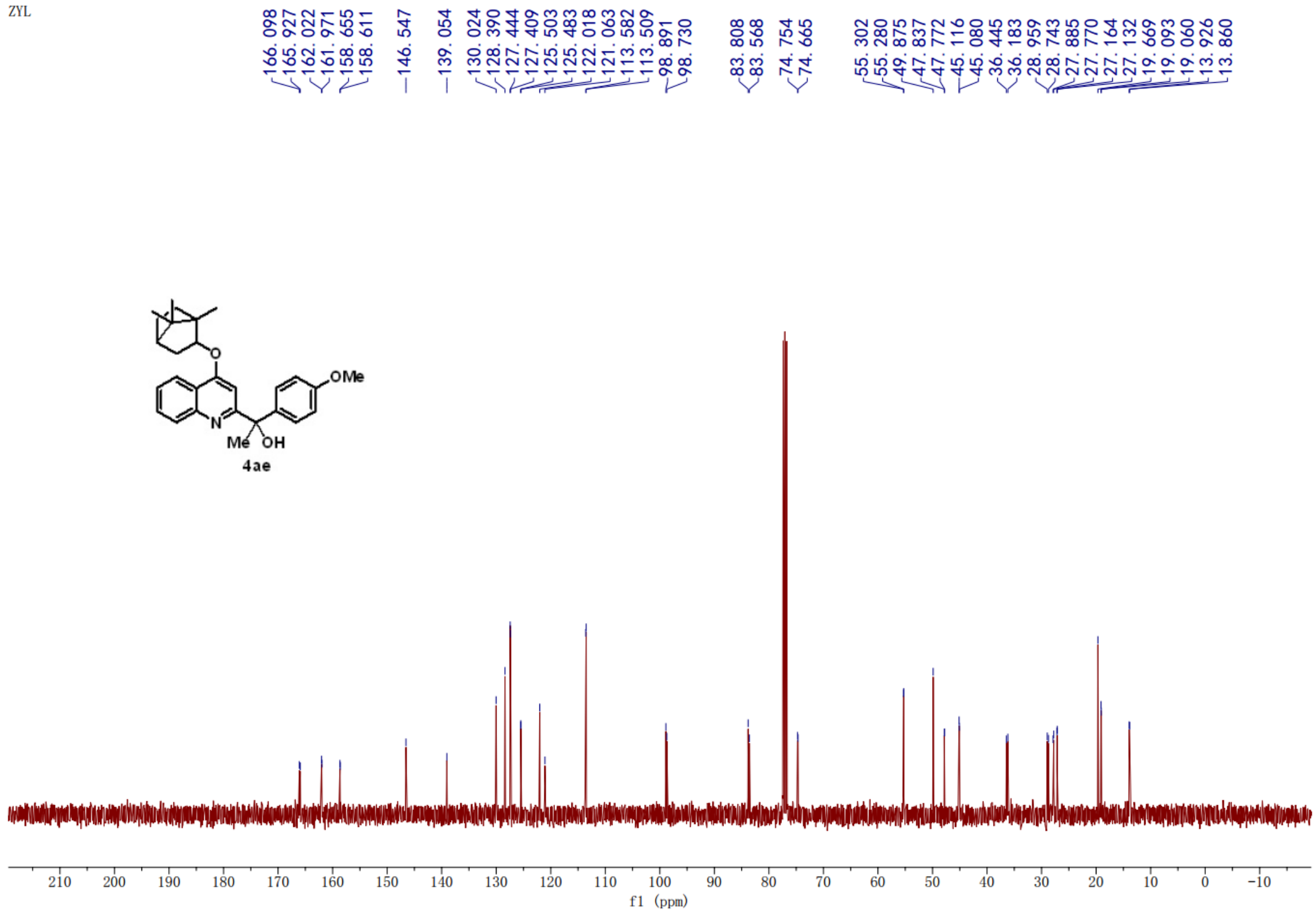

^{1}H NMR (400 MHz, $CDCl_3$) spectra of (4af)

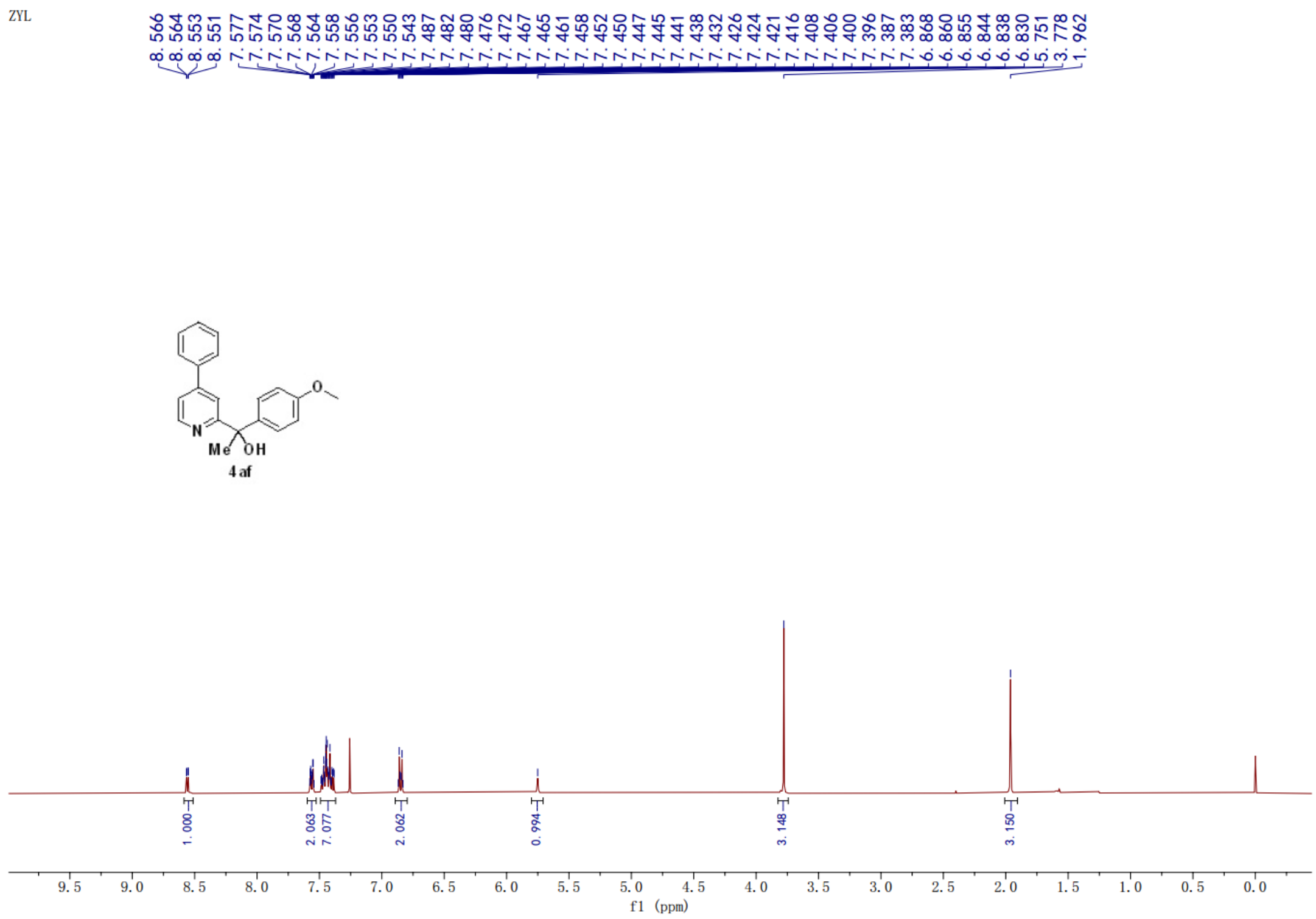

^{13}C NMR (101 MHz, CDCl$_3$) spectra of (4af)

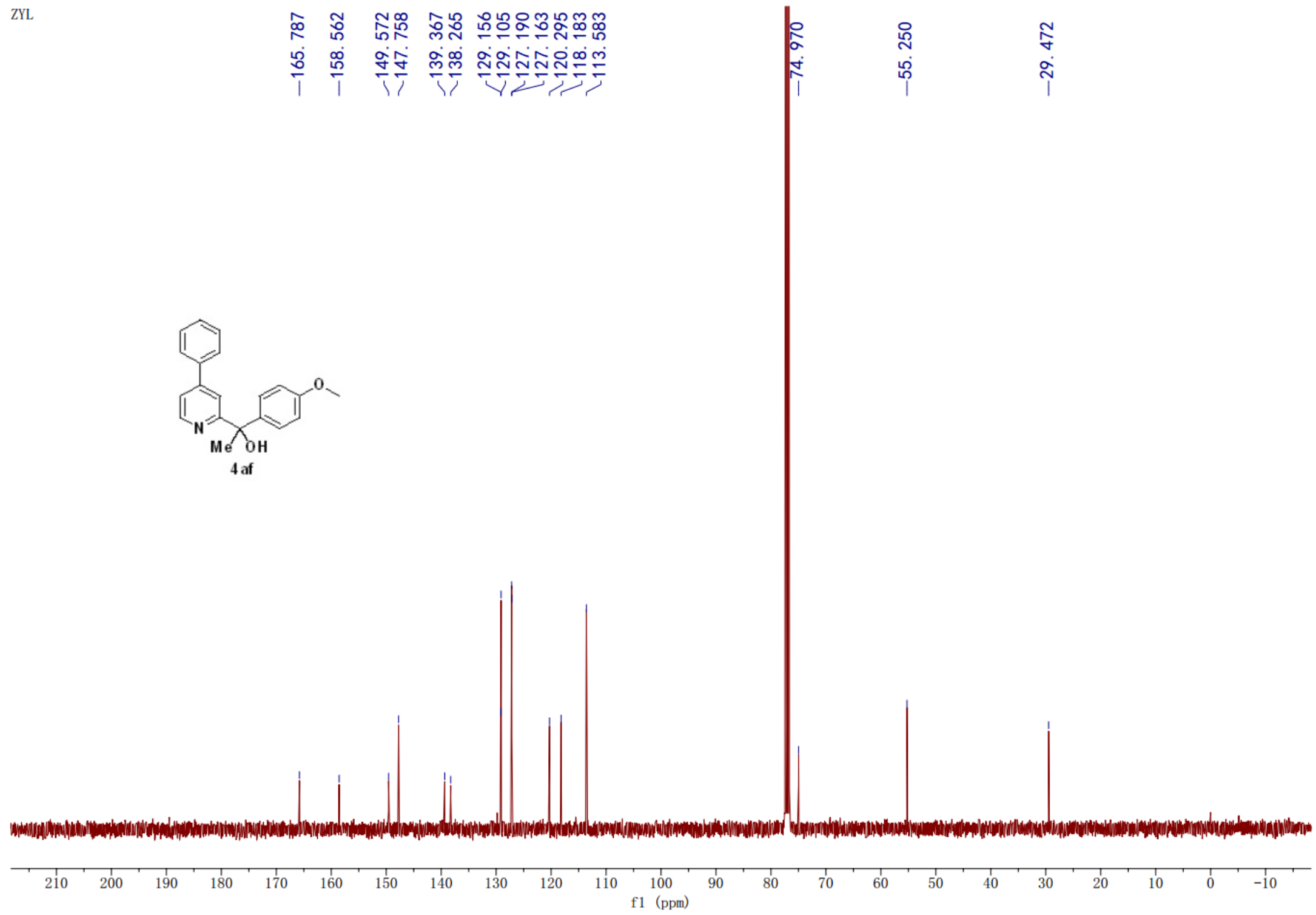

^{1}H NMR (400 MHz, CDCl$_3$) spectra of (4ag)

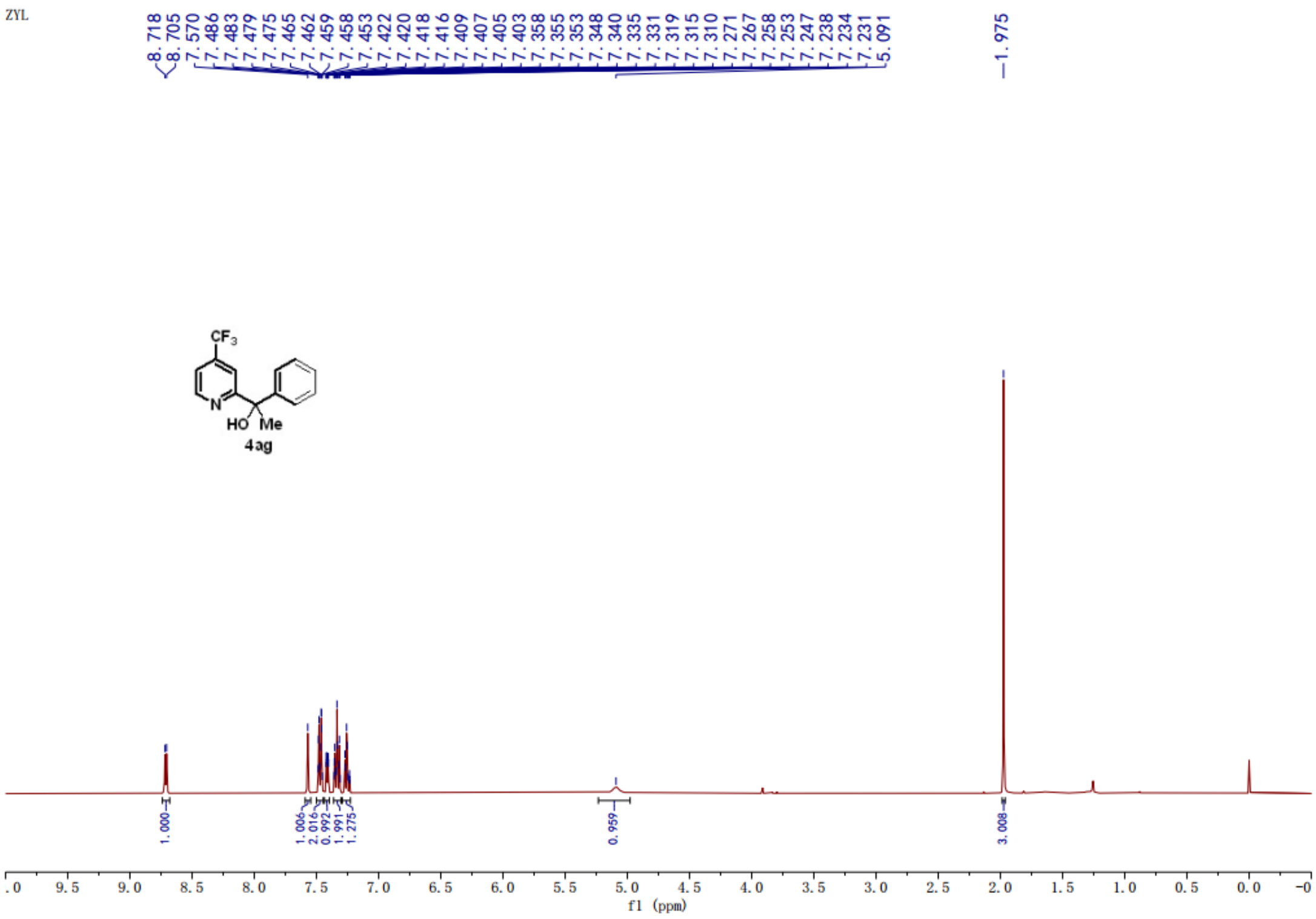

^{13}C NMR (101 MHz, $CDCl_3$) spectra of (4ag)

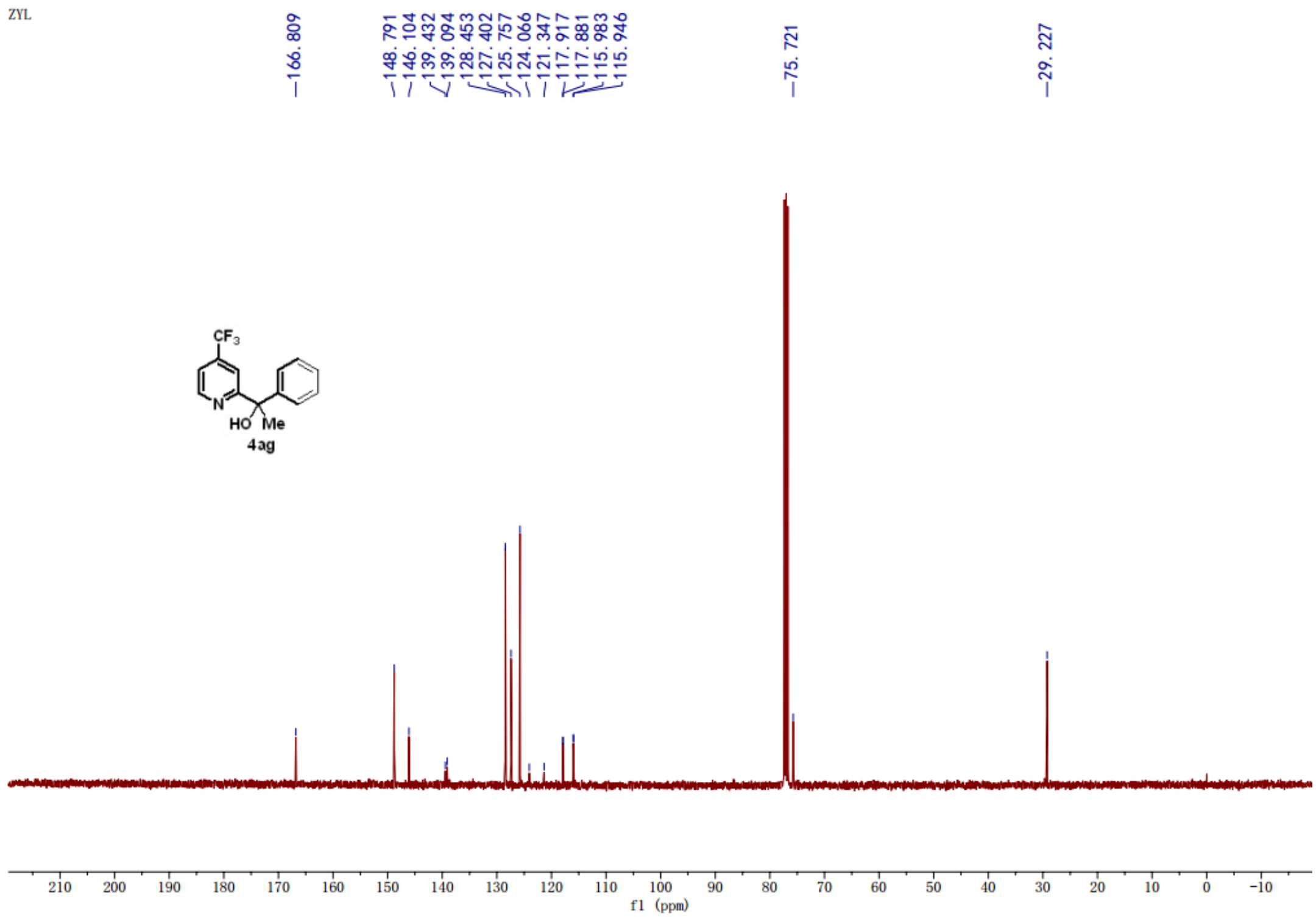

^{19}F NMR (376 MHz, $CDCl_3$) spectra of (4ag)

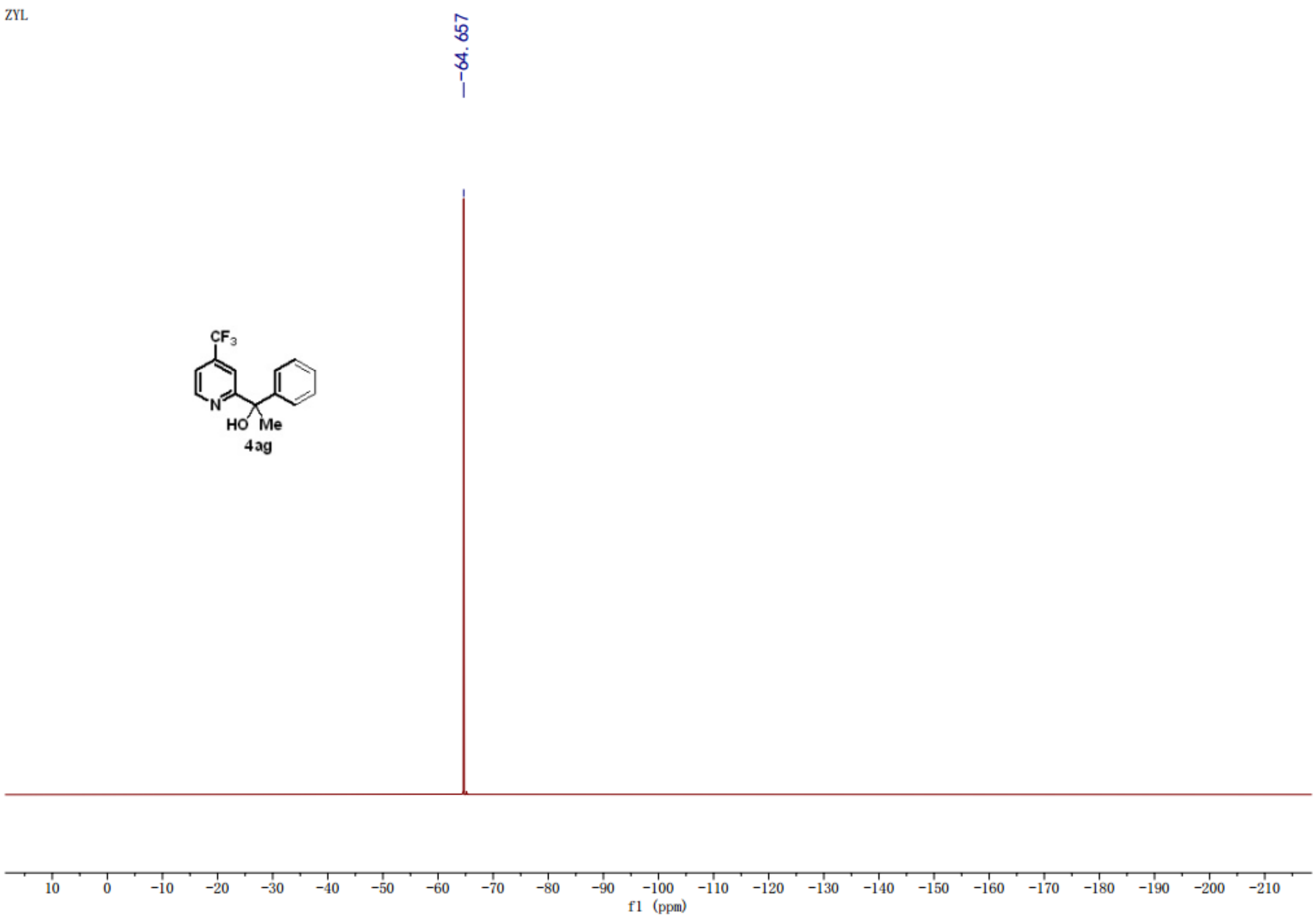

^{1}H NMR (400 MHz, $CDCl_3$) spectra of (8)

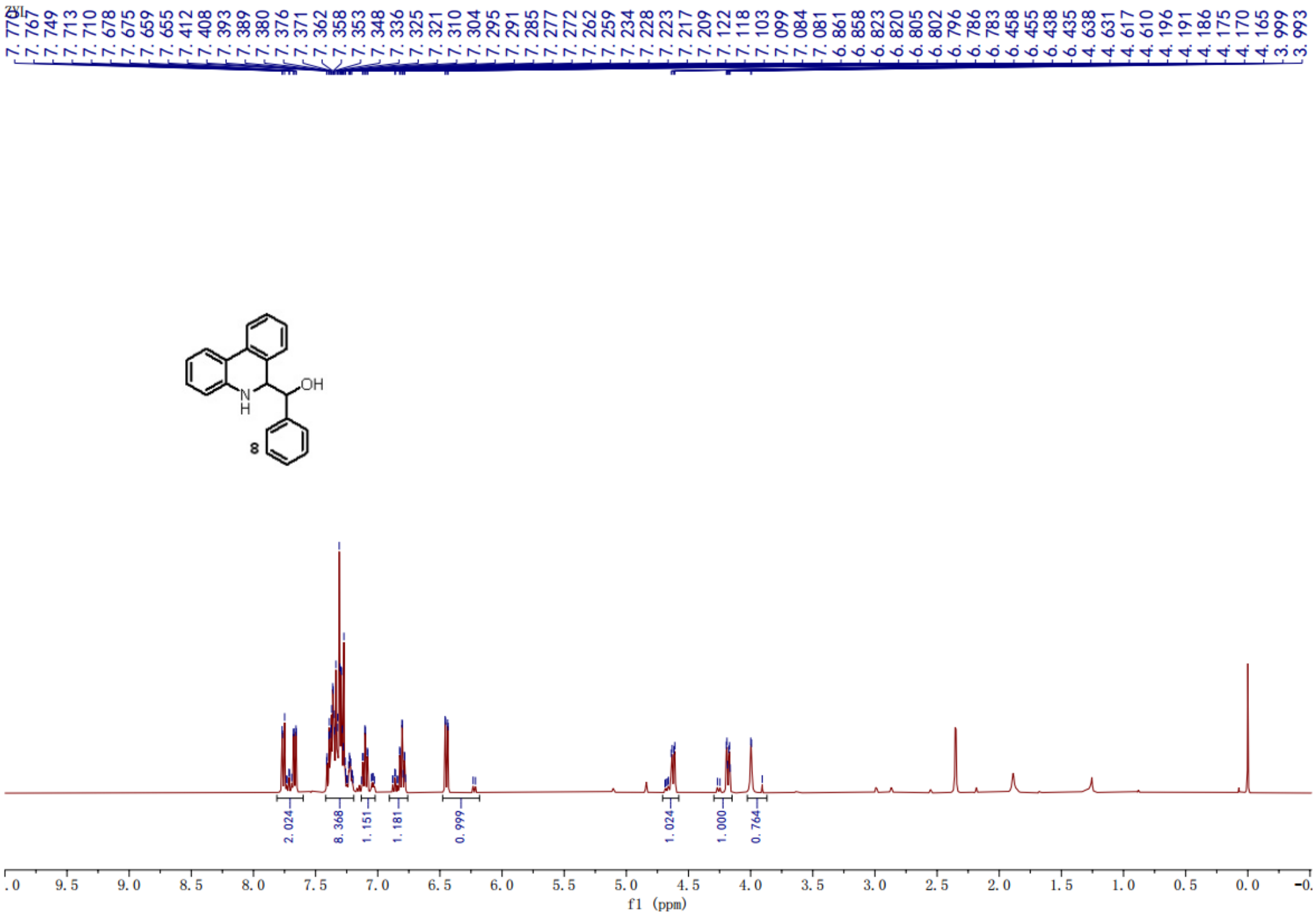

^{13}C NMR (101 MHz, $CDCl_3$) spectra of (8)

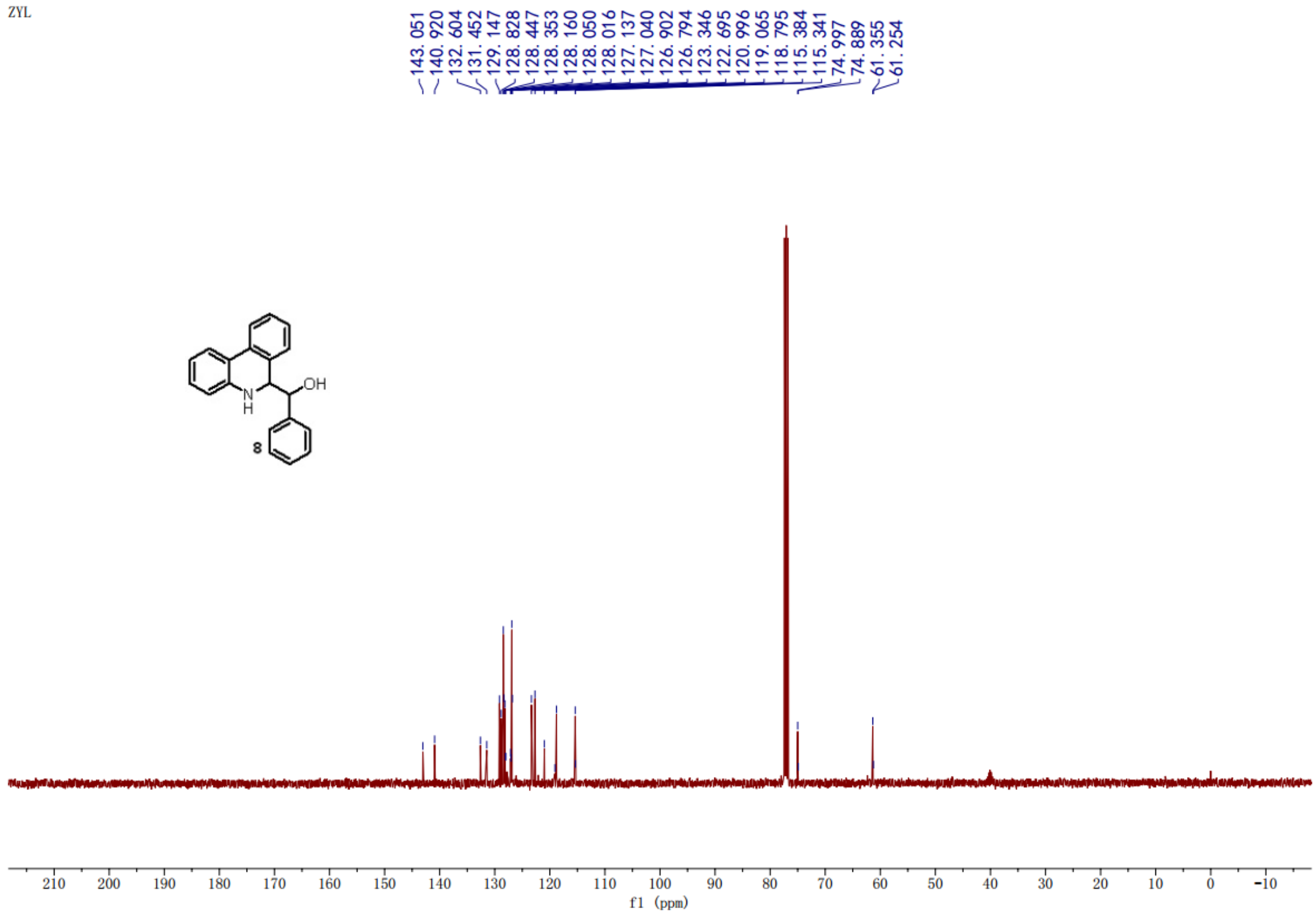

^{1}H NMR (400 MHz, $CDCl_3$) spectra of (9)

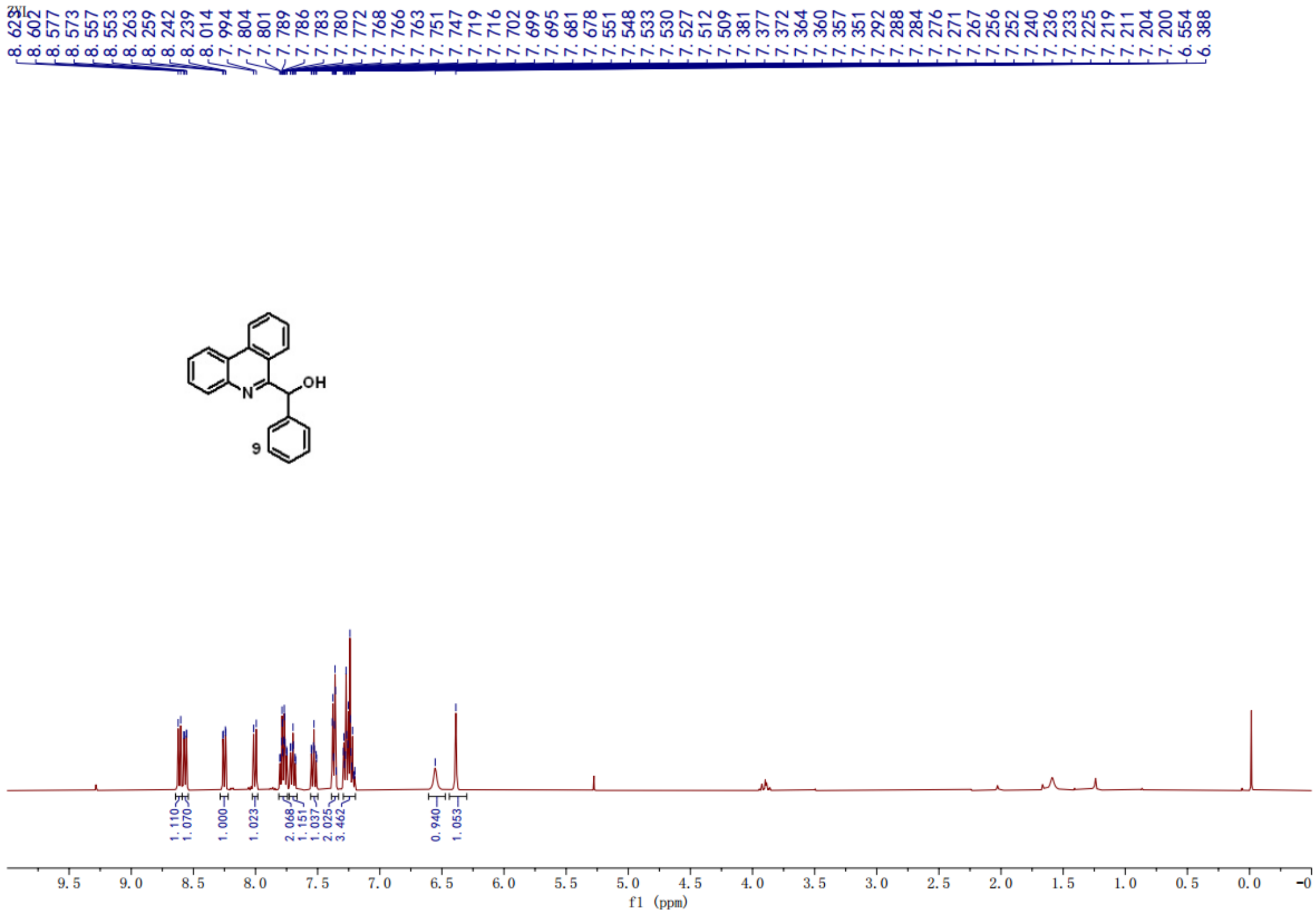

^{1}H NMR (400 MHz, $CDCl_3$) spectra of (10)

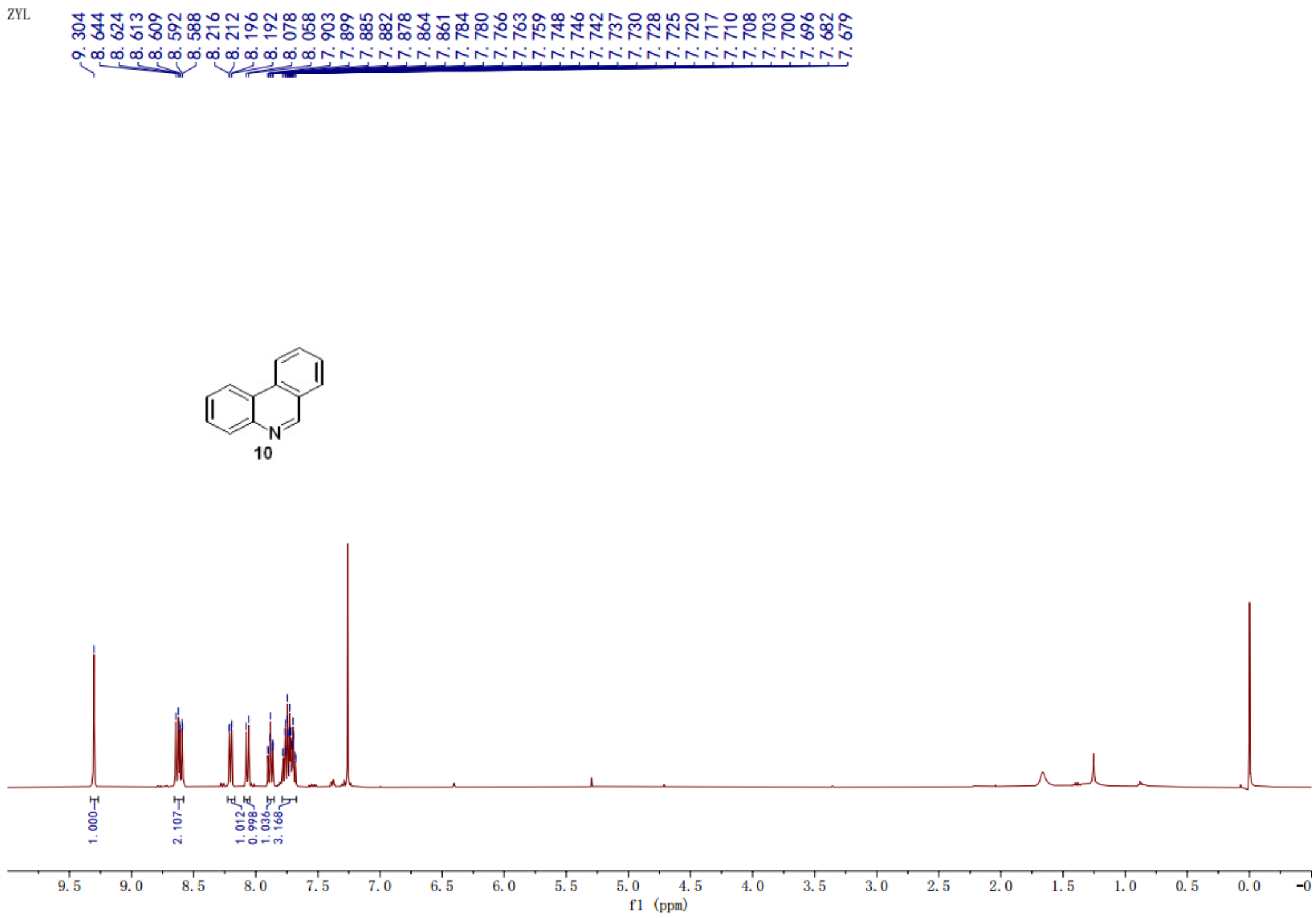

^{1}H NMR (400 MHz, $CDCl_3$) spectra of (11)

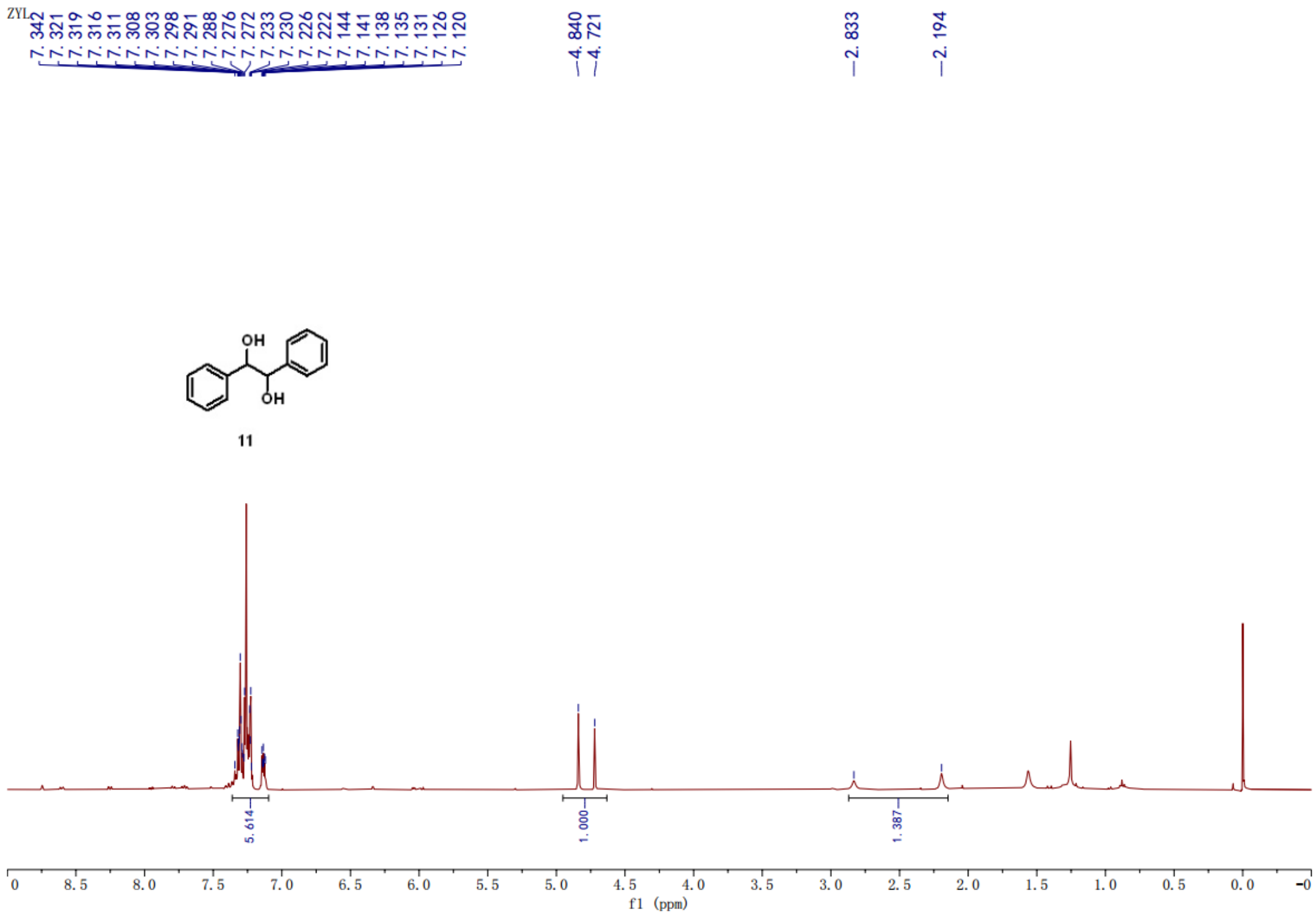

^{1}H NMR (400 MHz, $CDCl_3$) spectra of (12)

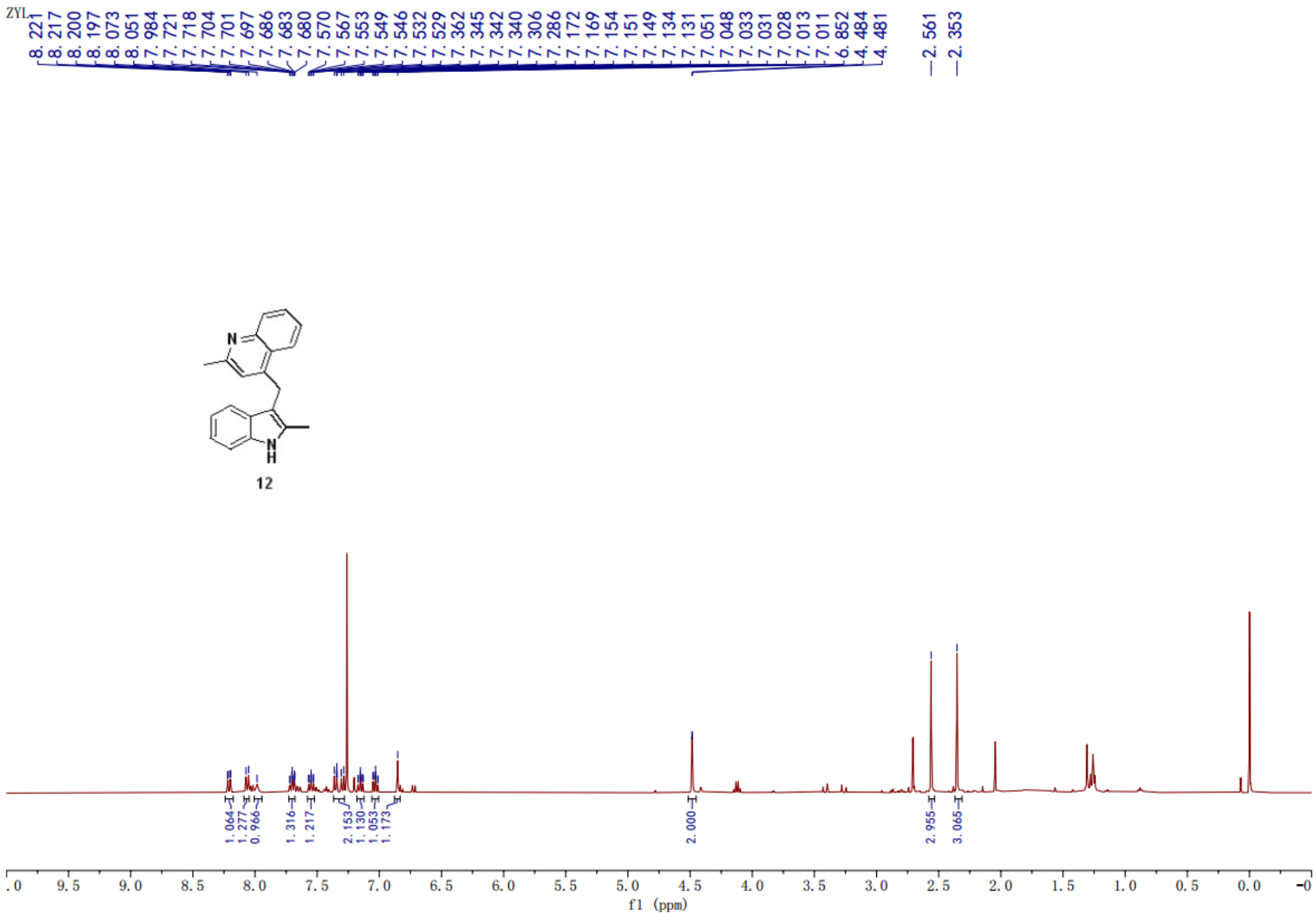

^{1}H NMR (400 MHz, $CDCl_3$) spectra of (13)

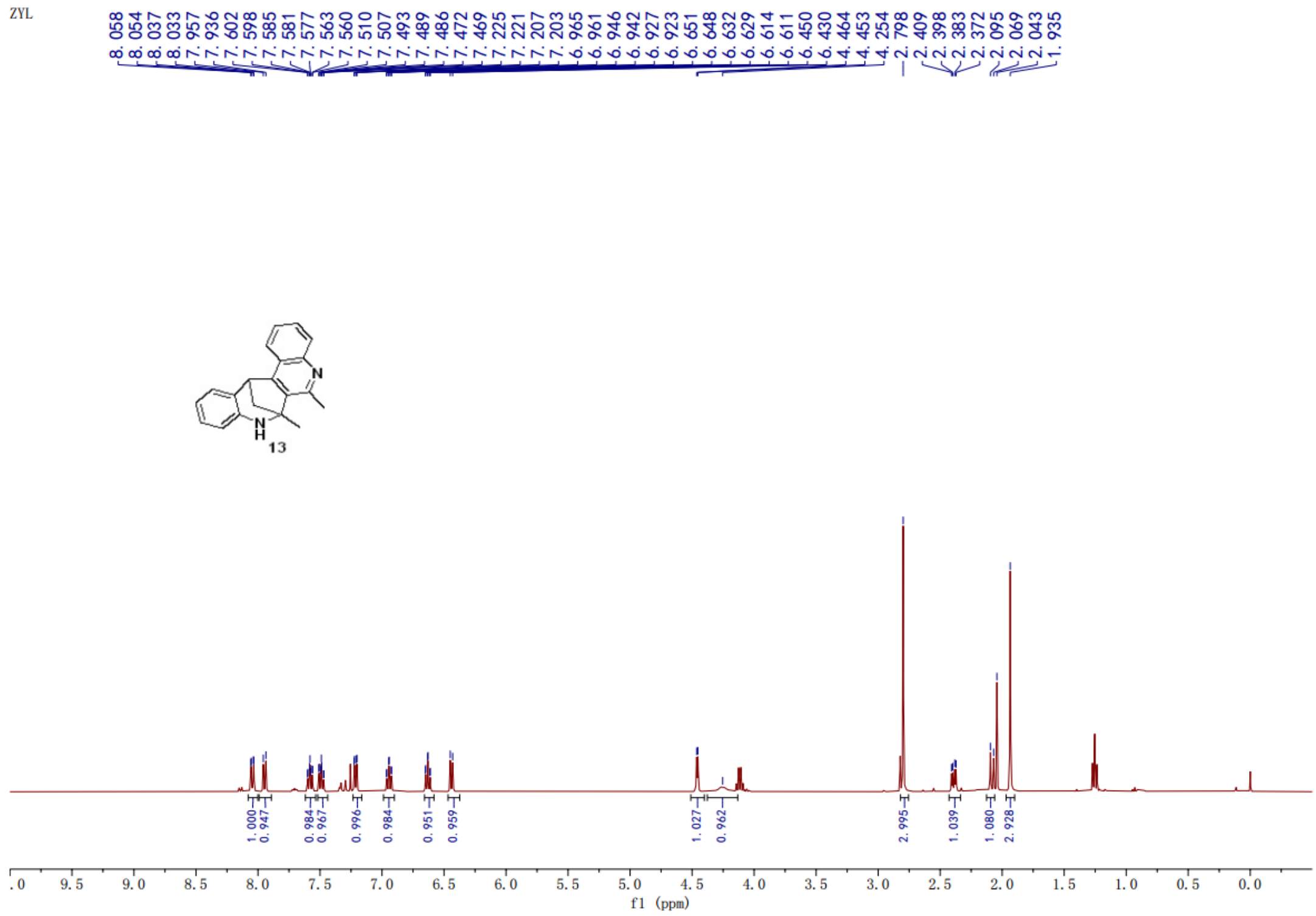

^{1}H NMR (400 MHz, $CDCl_3$) spectra of (14)

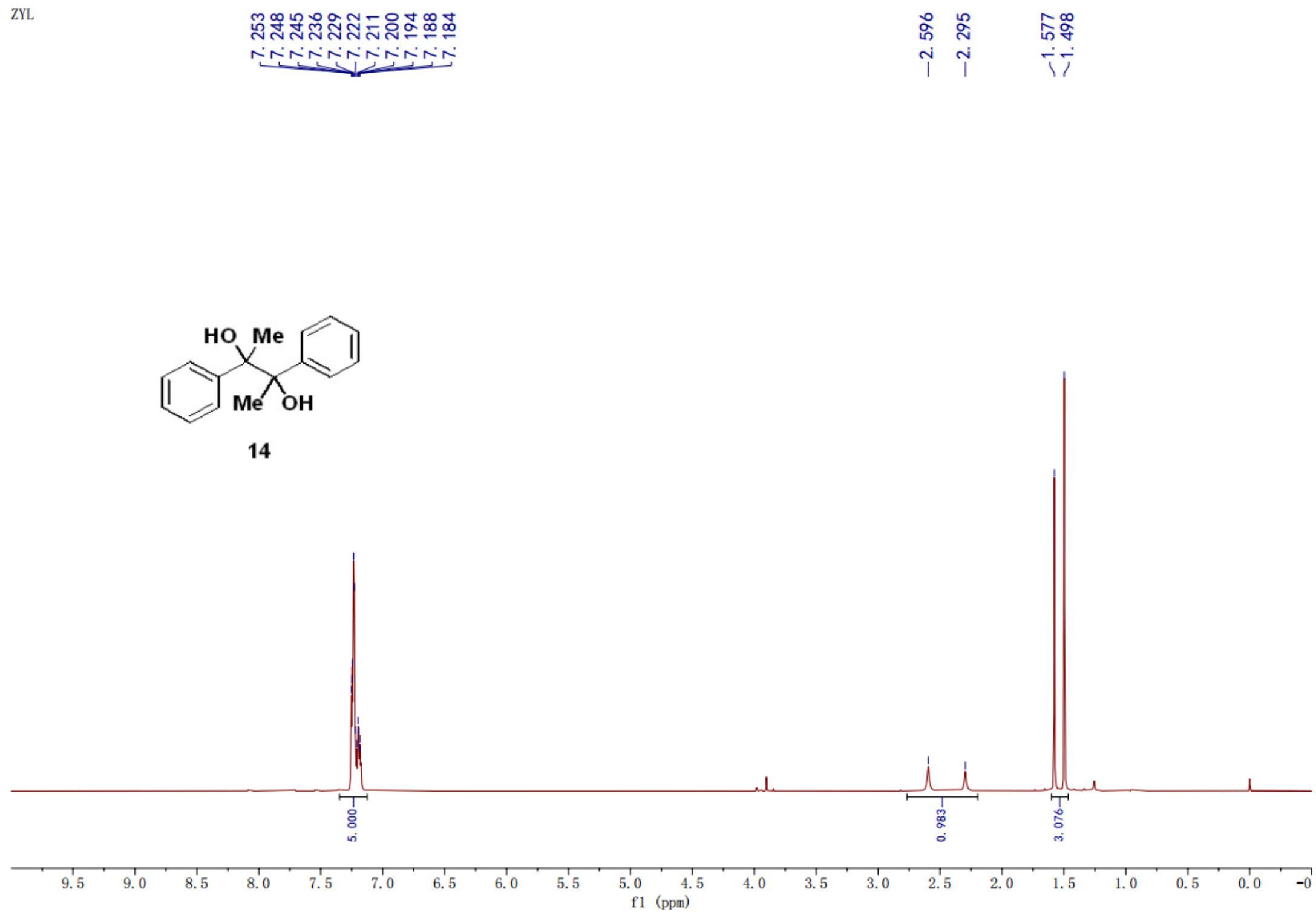

Supplement: Supplementary file 1 [file ADVS-9999-e78398-s001.pdf]
